# Synthesis and Fluorescence Profiling of Naphthylamine Analogs

**DOI:** 10.1002/cbic.70569

**Published:** 2026-10-09

**Authors:** Cole M. Friederichs, Jackson W. Berryman, Quentin M. R. Gibaut, Inga V. Leus, Chloe F. Cox, Helen I. Zgurskaya, Adam S. Duerfeldt

**Affiliations:** ^1^ Department of Medicinal Chemistry College of Pharmacy University of Minnesota Minneapolis Minnesota USA; ^2^ Department of Chemistry and Biochemistry University of Oklahoma Norman Oklahoma USA

**Keywords:** fluorescent probe, gram‐negative bacteria, internal conversion, *N*‐phenyl‐1‐naphthylamine, TD‐DFT

## Abstract

*N*‐phenyl‐1‐naphthylamine (NPN) has been widely used to probe the integrity of the outer membrane of gram‐negative bacteria. NPN fluoresces in the hydrophobic environment of lipid bilayers, whereas its fluorescence is quenched in the aqueous extracellular milieu. Because NPN does not intrinsically accumulate in intact gram‐negative bacteria, it has been broadly applied as a reporter of outer membrane permeabilization. Despite its widespread utility, systematic derivatization of this probe has remained largely unexplored. Here, we investigated the structure–fluorescence relationships of NPN through the synthesis of a diverse library featuring varied linkages to the fluorogenic naphthylamine core. Subsequent library expansion was informed by density functional theory and time‐dependent density functional theory studies, which suggested possible origins of fluorescence quenching in aqueous environments. The final resulting library comprised 95 compounds, of which 49 exhibited a fluorogenic profile similar to that of NPN. A selected subset of analogs was then evaluated in a fluorescence‐based accumulation assay using varied efflux and permeation strains of *Escherichia coli*, revealing that installed structural motifs influenced bacterial permeation. Collectively, these probes provide a foundation for further studies examining how small structural changes affect accumulation in gram‐negative bacteria.

## Graphical abstract

*N*‐phenyl‐1‐naphthylamine (NPN) has been historically used as a probe to monitor gram‐negative bacterial outer membrane permeabilization, fluorescing only in hydrophobic environments. Herein, a 95‐membered naphthylamine library was synthesized to explore structure–fluorescence relationships, informed by density functional theory studies. Forty‐nine analogs mimicked NPN’s fluorogenic behavior, and fluorescence‐based *Escherichia coli* accumulation assays revealed that specific structural motifs significantly influence bacterial accumulation and permeation.

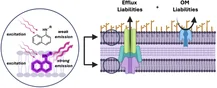

## 1 Introduction

The rapid rise of antimicrobial resistance (AMR) has greatly increased the need for novel, effective antibacterial agents [1]. Of particular need in the fight against gram‐negative AMR bacteria are chemical agents that overcome the bacterial defense mechanisms that limit intracellular drug accumulation. Specifically, effective antibacterial agents must permeate the amphiphilic cell envelope of gram‐negative bacteria (GNB) while avoiding efflux [2, 3]. Recent literature supports the hypothesis that small changes in chemical structure can significantly influence how compounds are recognized by various defense mechanisms, including the asymmetrical outer membrane (OM) and efflux pumps [4, 5, 6, 7, 8, 9]. Motif‐driven effects on uptake into GNB have been demonstrated across various scaffolds, including oxazolidinones, using mass spectrometry (MS)‐based quantification [10, 11]. While considered the field standard, MS quantification presents a significant bottleneck in the pipeline of screening for privileged motifs, as it is a time‐consuming and resource‐intensive technique. Given the rapid rate that bacteria can gain resistance to known antibiotics and the global toll of AMR on public health, there is a clear need for orthogonal approaches to understanding the effects of structural modifications on small‐molecule accumulation in GNB. One interest of ours is in the development of fluorescent small‐molecule libraries to enable the exploration of fluorescence‐based quantification as an alternative screening approach for motif‐driven structure–uptake relationships (SURs) in GNB.

Flurogenicity represents a promising photophysical property that could be exploited for rapid screening to establish SURs in GNB. Fluorogenic molecules can exhibit microenvironment‐dependent fluorescence profiles. A fluorogenic molecule that exhibits a unique fluorescence profile after accumulation in GNB would be a useful starting point for probe development so long as the scaffold is amenable to functionalization. Prior efforts to utilize fluorescence to track accumulation largely depend on the conjugation of known antibiotics to large fluorophores [12]. While useful for visualization, this approach dramatically changes the nature of the molecule being tracked. On the contrary, an inherently fluorescent library of small molecules that retain the same core but differ only in the motifs appended to the core could enable more efficient screening of motif‐driven effects compared to traditional time‐ and resource‐intensive MS‐based assays and is expected to (1) facilitate the identification of privileged, accumulation‐enhancing motifs, (2) enable the identification of accumulation hindering motifs, and (3) provide a tool chemical library to enable kinetic assessment of permeation, efflux, and accumulation in bacterial species of interest.

*N*‐phenyl‐1‐naphthylamine (NPN) is a fluorogenic probe that fluoresces only in the lipophilic inner membrane and in the inner phospholipid leaflet of the outer membrane of GNB but not in the extracellular aqueous environment [13, 14, 15]. NPN has been used to probe OM integrity in GNB as it can partition into and fluoresce within the hydrophobic cores of phospholipid membranes when the OM has been compromised by heat, salts, or chemical agents [16, 17, 18, 19]. Systematic derivatization of NPN to investigate the molecular mechanism(s) of fluorogenicity, as well as the structural perturbations that maintain or improve the fluorogenic nature of the probe, has not been reported. NPN is an attractive scaffold for diversification, as it contains the relatively small fluorogenic naphthylamine core. Notably, the aryl‐nitrogen of naphthylamine provides a synthetic handle to install a broad range of functionalities through a variety of chemical transformations. The development of such a collection of closely related fluorogenic probes would provide an opportunity to assess motif‐driven effects on OM penetration and efflux kinetics through the lens of fluorescence‐based quantification. We therefore sought to develop a diverse library of NPN derivatives with various linkages and motifs to determine how amenable the fluorogenic naphthylamine core is to functionalization.

Herein, we report the first round of structure‐fluorescence profiling of a library of 95 naphthylamine analogs. Our library explores the feasibility of amide, sulfonamide, urea, alkyl, and aryl linkages, with various motifs included in each series. Upon synthesis, the fluorogenic nature of each member was assessed in the presence and absence of *Escherichia coli* (*E. coli*) polar lipid extracts, through which we investigated the effects of small motifs and linker composition on the fluorogenic nature of the naphthylamine core. The roles of viscosity and solvent environment were also investigated computationally using density functional theory (DFT) analysis to provide insight into the origin of the fluorogenic behavior of functionalized naphthylamines and elucidate the preference of aryl‐substituted library members for desired fluorogenic behavior.

## 2 Library Design and Synthesis

This library was designed with a “shotgun” approach, appending a wide variety of functional groups and linkages to the naphthylamine core. While we speculated that both the naphthyl and distal phenyl groups must be conjugated, we tested a variety of linkers: aryl, alkyl, sulfonamide, amide, and urea (Figure 1). The amide series was synthesized through HATU coupling, with reactions yielding 33%–97%. The urea series was synthesized from their respective isocyanates in yields ranging from 40% to quantitative. We attribute low‐yielding representatives in the urea series to degraded isocyanate starting material. The aryl series was synthesized via microwave‐assisted Buchwald–Hartwig couplings, which enabled rapid access to the product but often generated complex mixtures of nonpolar byproducts, making purification difficult in some cases.

**FIGURE 1:**
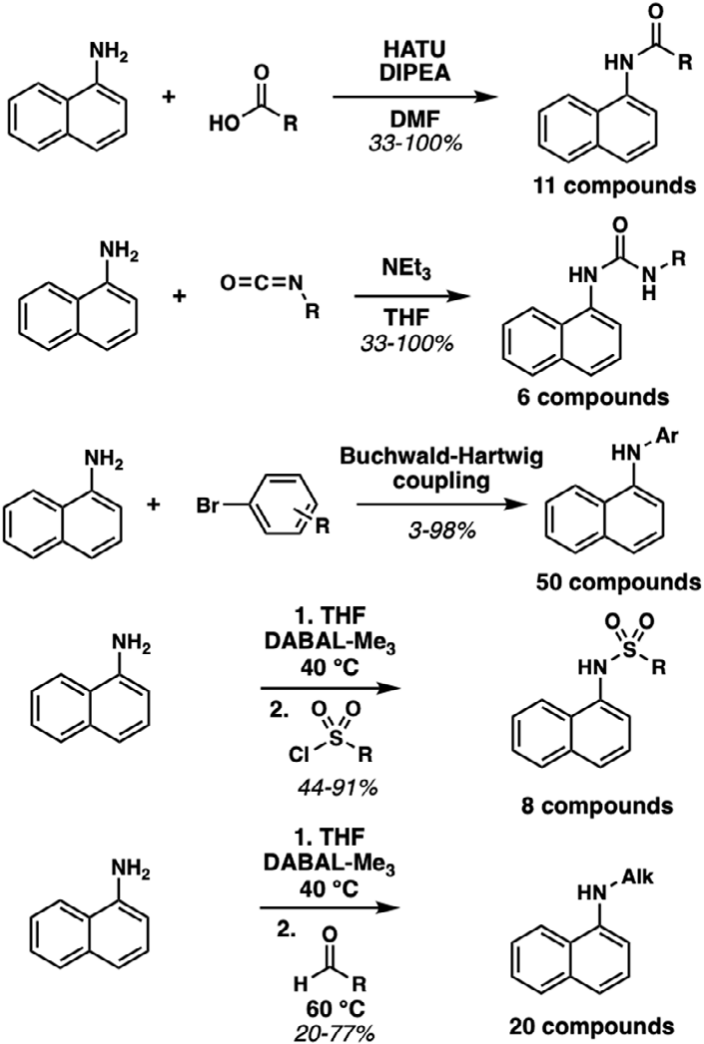
Overview of the most common synthetic routes used to access each linker class in the NPN analog library. Yield ranges for each series and the number of compounds prepared are shown.

Most notably, significant synthetic challenges were encountered in generating the alkyl series due to the poor nucleophilic character of naphthylamine. We hypothesized that the naphthylamine required activation for alkylation reactions to proceed. This was accomplished using DABAL‐Me_3_, a bench‐ and air‐stable AlMe_3_ equivalent, which increased the nucleophilicity and enabled efficient formation of the desired products [20, 21]. To our knowledge, this represents a new synthetic application of DABAL‐Me_3,_ which has traditionally been used to form amide bonds from amines and esters. Synthetic challenges due to low naphthylamine nucleophilicity persisted in the sulfonamide series, where naphthylamine exhibited low reactivity toward sulfonyl chlorides. Initial attempts to form sulfonamides under common conditions revealed no conversion; only under forceful conditions, using *n*‐BuLi at room temperature, was partial conversion accomplished. Once again, DABAL‐Me_3_ provided a significant improvement in yield (82% yield) for the test substrate (SA‐08). (Table SA) The use of DABAL‐Me_3_ also proved to be effective in generating analogs accessed through reductive aminations.

To assess the fluorescence and fluorogenic properties of the library, compounds were dissolved in biological‐grade DMSO and added to HMG buffer (50 mM HEPES‐KOH [pH 7.0], 1 mM magnesium sulfate, and 0.4 mM glucose), with or without *E. coli* polar lipid extract [8]. In solution, the lipid extract forms micelles that lipophilic probes partition into. Initial emission spectra were recorded under the same conditions as the parent probe, NPN. However, an excitation wavelength of 350 nm was suitable only for the aryl and alkyl series, while the optimal excitation wavelength for the amide, sulfonamide, and urea series was 320 nm. A cutoff of >5‐fold greater fluorescence in the presence of lipid extract, with a minimum intensity of 1000 RFU, was established to classify a given compound as fluorogenic. Notably, the strongest fluorogenic response often appeared not at the wavelength of maximum emission but at shorter wavelengths near the leading shoulder of the emission band, where the probe in HMG buffer alone showed little to no fluorescence.

Of the initial 60 analogs generated (Figures S2–S6), 19 exhibited sufficient fluorogenic properties (Figure 2 and Supporting Information). Unsurprisingly, the aryl series performed well, with 7/15 compounds meeting the requirements for classification as fluorogenic. The amide series provided no fluorogenic compounds. Both the urea and sulfonamide series contained a single compound that remained fluorescence. Unexpectedly, the unconjugated alkyl series contained 10 fluorogenic compounds out of the 20 synthesized. We anticipated that the naphthylamine unit would need to be conjugated via the linker to the installed motif and were pleased to observe that fluorogenic profiles were maintained in this series.

**FIGURE 2:**
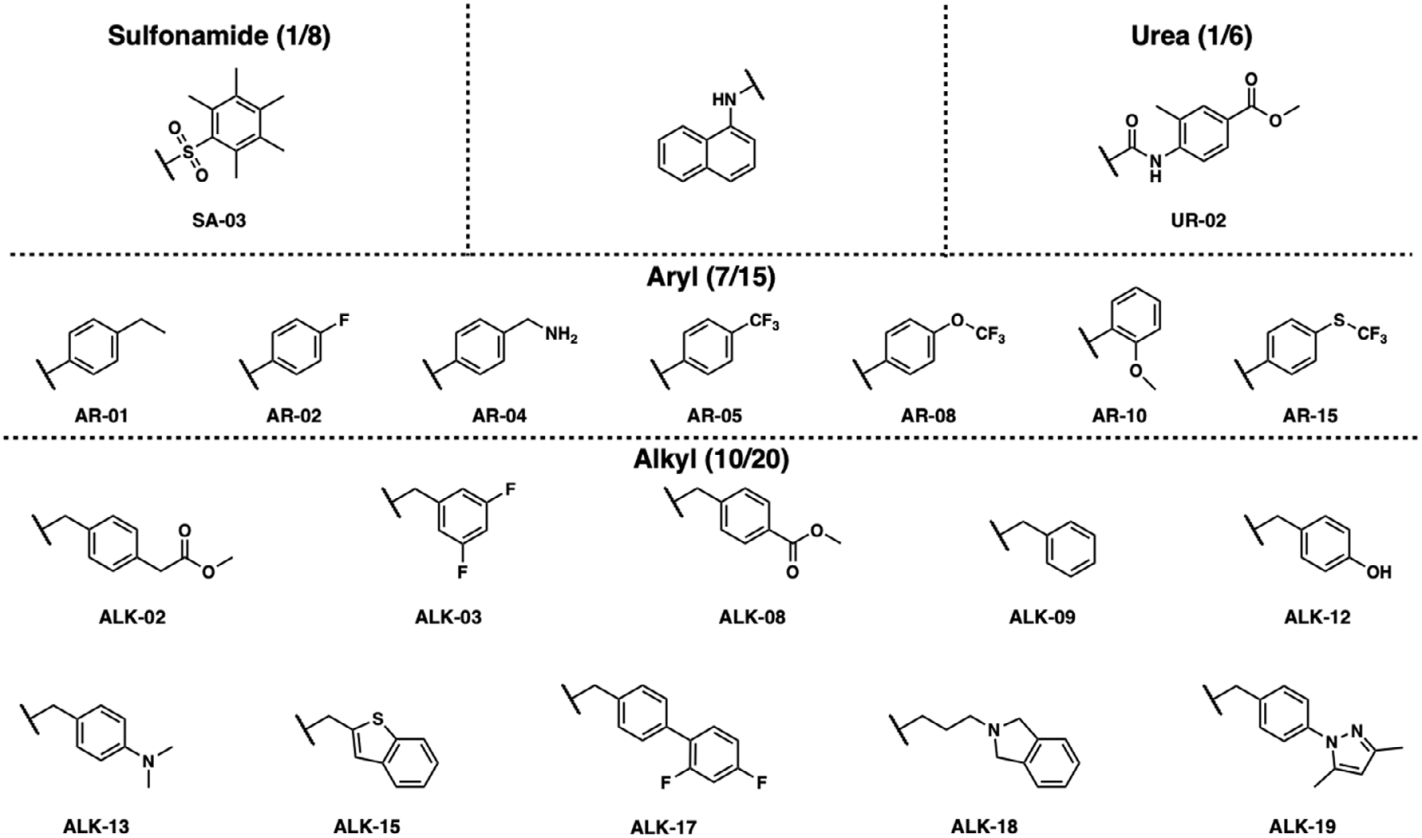
Structures of NPN analogs that retained fluorogenicity across the synthesized library. Compounds are grouped by linker class, with the number of fluorogenic analogs relative to the total number prepared shown in parentheses. No members of the amide series exhibited fluorogenic behavior.

### 2.1 Proposed Mechanism for Fluorogenicity

To investigate the fluorescence mechanism of this scaffold further, with the goal of enabling rational design of additional fluorogenic analogs, DFT and time‐dependent DFT (TD‐DFT) calculations were performed. The fluorogenic behavior of NPN has previously been attributed to microenvironment polarity or local microviscosity that prevent rotation around key bonds, breaking conjugation between the phenyl and naphthyl groups [15, 22, 23, 24, 25, 26, 27, 28]. In order to investigate this theory, NPN was optimized in the ground state at the *ω*B97X‐D4/def2‐TZVP level of theory, followed by optimization of the first excited singlet state in the gas phase in ORCA [29, 30]. A very minor difference between the S_0_ and S_1_ geometries was observed (Figure 3A,B), which is inconsistent with the characteristics of a twisted intramolecular charge‐transfer (TICT) probe [31]. Furthermore, the ground and excited states were also optimized using the CPCM solvation model in both water and hexane. Hexane was chosen as a low‐dielectric solvent model to best approximate the lipophilic lipid bilayer environment. The optimized geometries were similar across the gas, water, and hexane models. Additionally, the oscillator strengths, which provide insight into how likely a molecule is to absorb light or fluoresce, remained similar across all conditions. The DFT results across gas phase and CPCM solvation calculations suggested that variables outside solvent‐stabilization effects on the excited state contributed to the fluorogenic behavior.

**FIGURE 3:**
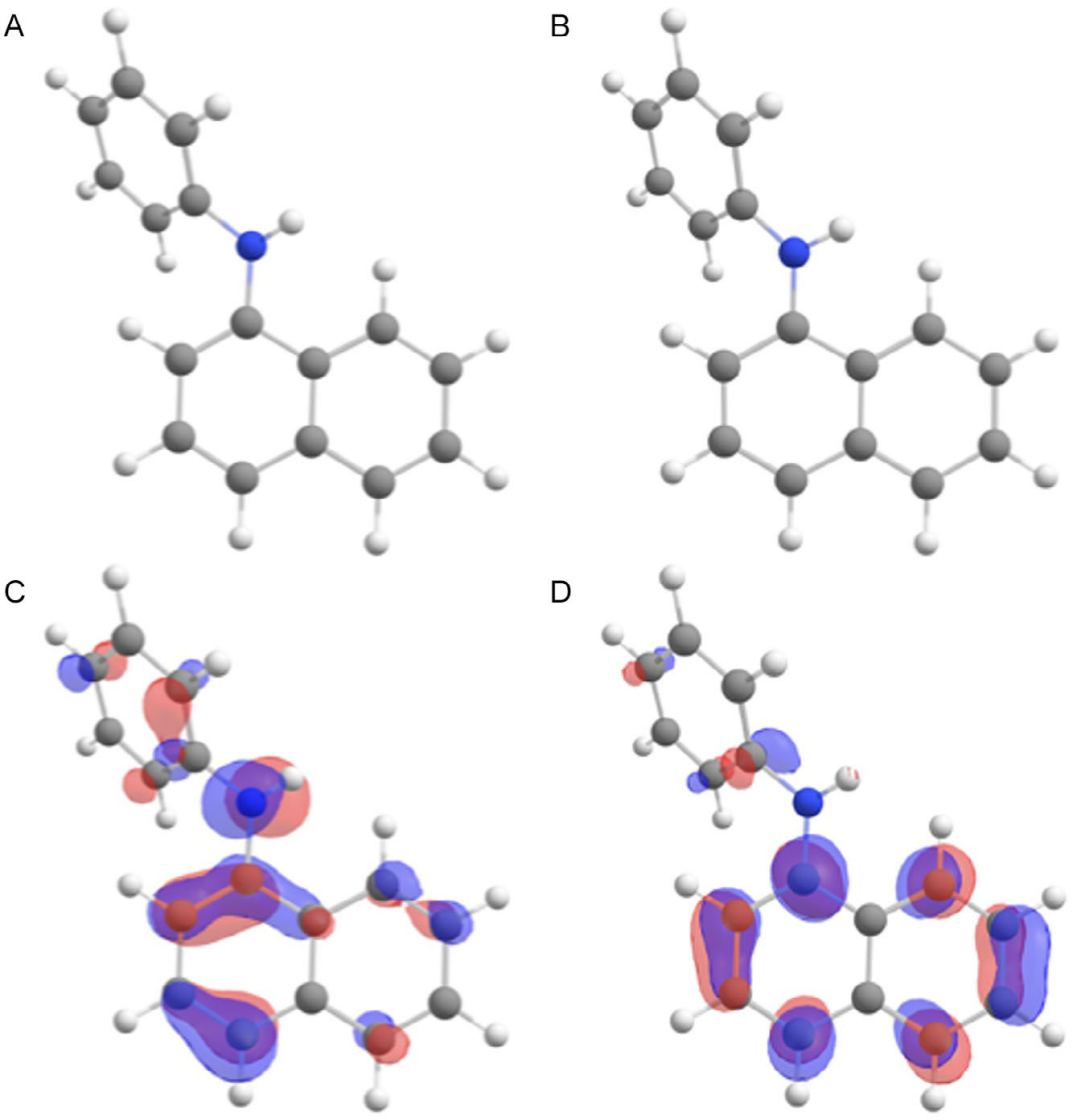
Computed geometries and natural transition orbitals associated with the S0 → S1 transition of NPN. (A) S0 optimized geometry. (B) S1 optimized geometry. (C) Particle NTO at the S0 geometry. (D) Hole NTO at the S1 geometry.

Prior to DFT studies, we considered that NPN might behave as a molecular rotor or a TICT probe due to the central nitrogen atom connecting the phenyl and naphthyl groups [32]. This hypothesis has also been proposed in earlier literature, prior to the term TICT being coined [33]. Natural transition orbitals indicated that the S_0_ to S_1_ transition involves the transfer of electron density from molecular orbitals primarily delocalized on the naphthyl ring to molecular orbitals that are primarily delocalized on the phenyl ring (Figure 3C,D). This suggests the possibility that rotation around the *N*‐aryl bonds could play a role in nonradiative quenching. To test this hypothesis, the fluorescence emission of NPN was experimentally measured in binary solutions of ethylene glycol and glycerol. This combination was chosen because both solvents are strongly polar and capable of hydrogen bonding, and different ratios yield different levels of viscosity. The ethylene glycol and glycerol solutions provided a useful system for assessing the relationship between fluorescence intensity and viscosity as a range of viscosities can be screened while maintaining a consistent, strongly polar environment. The resulting spectra suggest that both polarity and viscosity contribute to the emission profile (Figure 4A,B). A positive correlation was observed between fluorescence intensity and viscosity, with the 100% glycerol sample exhibiting greater fluorescence intensity than the 100% Ethylene glycol sample (Figure 4A,B). The maximum emission wavelength in these solutions (441 nm) was more redshifted than the maximum emission in lipid micelles (415 nm). In a Förster–Hoffmann analysis, a molecular rotor or TICT probe should have a linear response to viscosity. In our analysis, a correlation is observed between fluorescence and viscosity, although the curve appears to exhibit an exponential component. This suggests that rather than NPN acting entirely as a molecular rotor, other quenching mechanisms (e.g., polarity) are likely involved. In addition to these viscosity experiments, the fluorescence emission spectra for NPN were collected in a variety of organic solvents, which demonstrated that solvent dielectric alone is not capable of fluorescence enhancement or quenching (Figure S1 and Table SC).

**FIGURE 4:**
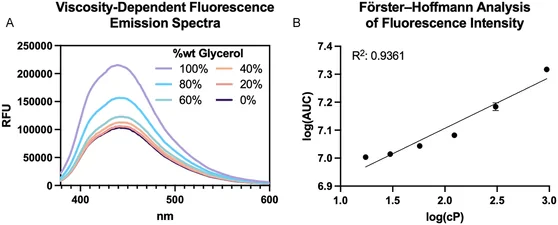
Fluorescence emission of NPN in binary ethylene glycol/glycerol solutions. (A) Emission spectra recorded in solvent solutions of varying viscosity, showing both viscosity‐dependent intensity changes. (B) Förster–Hoffmann analysis of viscosity emission spectra.

The viscosity experiment, combined with DFT calculations, prompted us to explore additional pathways that might be affected by local viscosity. Using ORCA’s excited‐state dynamics modules at the ωB97X‐D4/def2‐TZVP level of theory, the rates of internal conversion and intersystem crossing were examined. Initially, rates of fluorescence, internal conversion, and intersystem crossing from S_1_ and the nearest triplet state of NPN in the gas phase were calculated. These calculations revealed that the internal conversion rate was approximately 10‐fold higher than the emission rate, while intersystem crossing was about twofold greater (Table SG). With this insight, we then calculated the oscillator strengths and rates for all linkers with an unsubstituted phenyl group to simplify the analysis. The sulfonamide derivative SA‐03 exhibited a low oscillator strength (<0.05) for both absorption and emission transitions, indicating it would fluoresce weakly regardless of the environment; the other linkers had sufficient oscillator strengths (>0.20) (Table SE). Across all linkers, internal conversion occurred roughly 10‐fold faster than fluorescence emission (Table SE). Intersystem crossing rates varied more across the linkers but were generally slower than internal conversion rates. Given that the initial *N*‐aryl series accounted for a large portion of the fluorogenic compounds and were straightforward to synthesize, NPN rates were evaluated using CPCM to assess solvent dielectric effects. We calculated that emission was 3.4 times faster in hexanes than in water; however, internal conversion rates stayed roughly the same. Intersystem crossing was nearly 10‐fold faster in water than in hexane, but internal conversion quenching remained the fastest calculated nonradiative pathway.

Internal conversion is believed to be driven by molecular vibrations and their energetic overlap between S_0_ and S_1_ states. Both DFT calculations and fluorescence testing in ethylene glycol and glycerol solutions suggest that solvent dielectric effects alone are insufficient to explain nonradiative quenching and that viscosity contributes significantly to the fluorogenic properties of naphthylamine derivatives. In a more viscous environment, such as a lipid bilayer, reduced molecular motions may suppress the nonradiative quenching pathway, thus favoring radiative fluorescence.

Based on fluorescence profiles from the initial library, synthetic accessibility, and the DFT results, we anticipated that the aryl series would be the most expandable. Although the alkyl linker showed similar rates computationally, the flexibility of the linker and the more complex synthesis of derivatives led us to prioritize the *N*‐aryl scaffold for further derivatization. The aryl library was expanded by synthesizing an additional 35 compounds (Figure S7), of which 30 exhibited the desired fluorogenic behavior (Figure 5).

**FIGURE 5:**
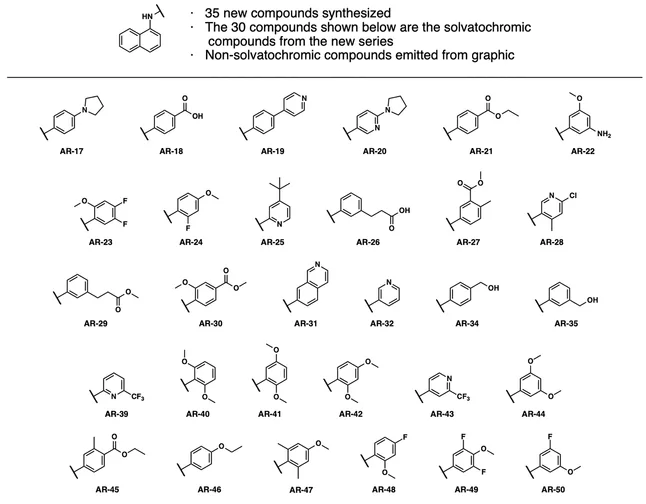
Fluorogenic members of the expanded *N*‐aryl naphthylamine library. Thirty compounds retained the desired fluorogenic profile and are shown here, with the shared NPN‐derived fluorophore core shown above.

### 2.2 Fluorescence‐Derived Cell‐Associated Accumulation Assay

To provide proof‐of‐concept that the library could be utilized to determine accumulation profiles and identify motif‐specific influence, a subset of four “well‐behaved” library members was selected for accumulation studies in varied efflux and permeation strains of *E. coli*. Cell association was measured in 4 different isogenic strains of *E. coli*: wild type (WT), hyperporinated (P), efflux‐deficient (E), and doubly compromised—hyperporinated and efflux‐deficient (PE) strains [8, 34, 35]. Testing in isogenic strains allows one to distinguish external partition into the outer membrane from the intracellular permeation and to decouple the effects of efflux and outer‐membrane exclusion for compounds of interest. The hyperporinated and efflux‐deficient (PE) strain is used as a benchmark to assess accumulation when the two most prevalent mechanisms preventing accumulation are absent. A ratio of the accumulation slope in PE strain to the slope of either hyperporinated (P) or efflux‐deficient (E) strains can be used as an indicator of each barrier’s impact on compound accumulation. Contrary to previous work, the inverse PE/P and PE/E rather than P/PE and E/PE ratios were utilized so that the trend of larger ratios corresponding to greater impact on intracellular accumulation remains true. To quantify relative accumulation, concentration‐dependent slopes were used.

Importantly, membrane association is required for the fluorescence of NPN. However, the exact membrane location(s) within the cellular envelope where NPN fluoresces remains unclear. Given that low fluorescence is observed upon treatment of WT bacteria, negligible fluorescence arises from probe aggregation outside bacterial membranes or from general cell association. On the other hand, much higher fluorescence is observed upon treatment of membrane‐compromised strains. This suggests that a significant majority of fluorescent signal is generated from probes that have at least penetrated the outermost layer of the cell envelope. The concentration of probe outside (as in WT) versus intramembrane versus cytosolic exist in an equilibrium primarily controlled by the individual partition coefficient of each probe, which can be calculated from molecular structure. Acknowledging this equilibrium, NPN probes that generate fluorescent signal(s) in membrane‐compromised strains also achieve intracellular accumulation in accordance with their respective partition coefficient.

In order to better understand the chronological order and kinetics of membrane association and eventual accumulation, kinetic experiments were performed (Figures S8–S12). In all bacterial strains tested, a fast‐binding step to the cell surface was observed. However, more drawn‐out time‐dependent changes in fluorescence were observed for membrane‐compromised cells. This suggests a slow binding step in these strains not present in WT, further supporting permeation and binding to internal membranes in these strains. Together, these data suggest that probes that generate a fluorescent signal have partitioned beyond simple membrane association.

In agreement with previous data, the accumulation of NPN is equally impacted by efflux (PE/P = 2.71) and OM permeation (PE/E = 3.08, Figure 6C) [36]. ALK‐03, however, containing a 3,5‐difluorobenzyl group on the naphthylamine, is more limited by OM penetration (PE/E = 3.74) than NPN and less so by efflux (PE/P = 2.02). ALK‐15, which contains a methylene‐linked benzothiophene, displays a slightly higher efflux liability than NPN (PE/P = 3.10) and significantly greater difficulty traversing the OM (PE/E = 7.92). The two new aryl analogs, AR‐01 and AR‐04, exhibit much more differentiated accumulation profiles than NPN. AR‐01 exhibits a PE/P ratio of 1.99, demonstrating a decrease in susceptibility when compared to NPN. However, AR‐01 is heavily limited by OM penetration (PE/E = 13.68), an effect ~4.5‐fold higher than that measured for NPN. AR‐04 contains a benzylamine motif, a functionality that should enable porin‐mediated accumulation in WT *E. coli*, as per the eNTRY rules [4]. Accumulation, however, in wild‐type *E. coli* is negligible. Interestingly, AR‐04 is less impacted than NPN in both efflux (PE/P = 1.53) and OM penetration (PE/E = 1.19); notably, this was the only efflux ratio statistically significantly different from NPN.

**FIGURE 6:**
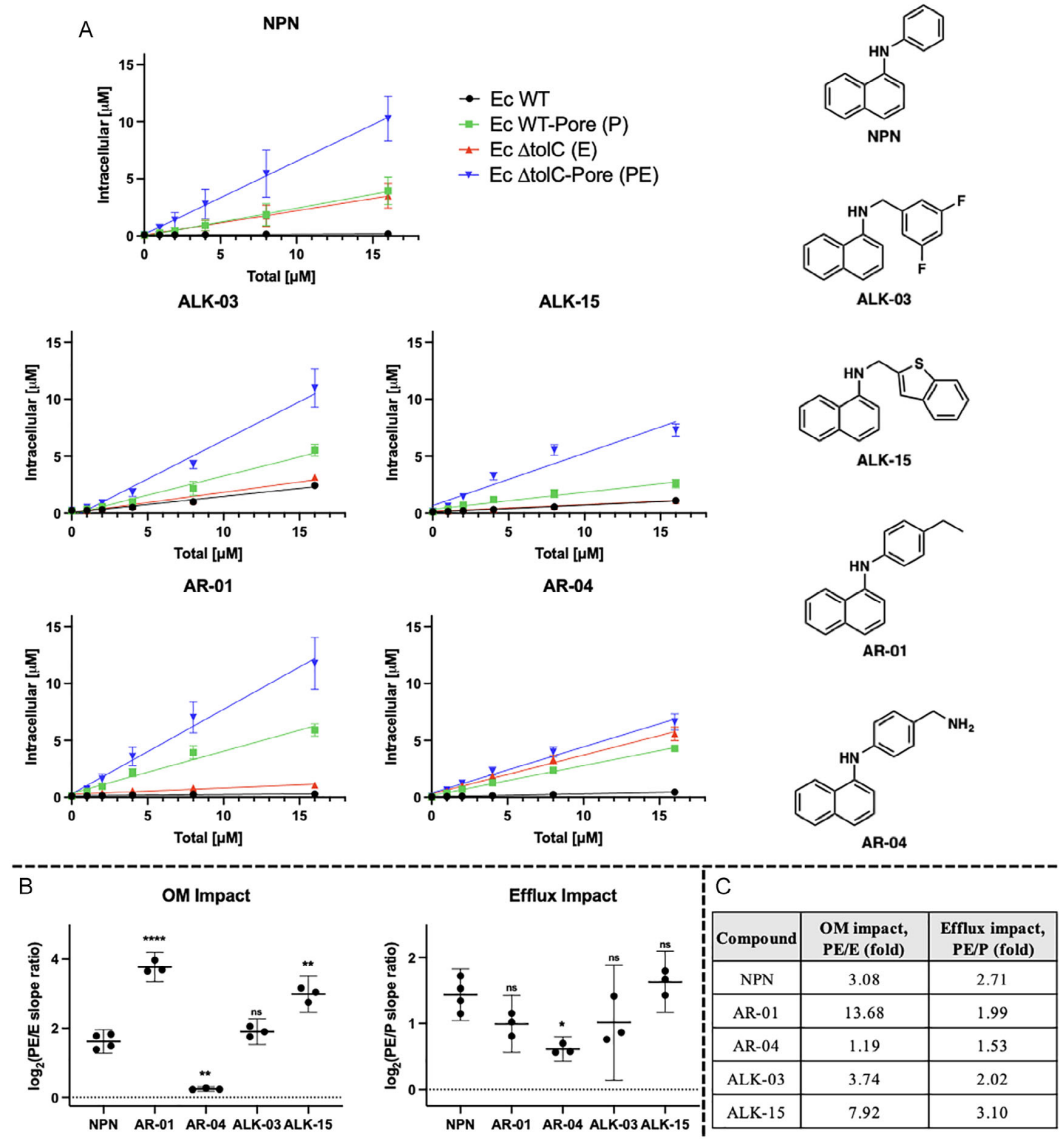
Accumulation behavior of NPN and selected analogs in isogenic *E. coli* strains: WT, hyperporinated (P), ΔtolC (E), and hyperporinated ΔtolC (PE). (A) Fluorescence derived accumulation curves. Error bars represent SD of three biological replicates. (NPN *n* = 4, other analogs *n* = 3). (B) Outermembrane and efflux impact ratios derived from the slopes. Ratios were log_2_‐transformed for statistical analysis. Each point represents one biological replicate with error bars indicating the 95% confidence interval. Dotted line at *y* = 0 corresponds to a ratio of 1. Each analog was compared to NPN using Brown–Forsythe and Welch one‐way ANOVA followed by Dunnett’s T3 multiple‐comparison test. ****p<0.0001; **p<0.01; *p<0.05; ns, not significantly different from NPN. (C) Geometric mean slope ratios obtained by back transforming the mean log_2_ ratios and expressed as fold differences.

## 3 Conclusion

In summary, this work demonstrates that the naphthylamine core is amenable to library expansion while retaining fluorogenic character. The presented computational studies provide new insight into potential mechanisms of nonradiative fluorescence decay, which contributed to the motivation to expand the library by 35 additional *N*‐aryl naphthylamine derivatives, of which 86% exhibited fluorogenic behavior. This represents a substantial improvement from the initial library, of which only 32% of the 60 compounds exhibited fluorogenic behavior.

As proof‐of‐concept, we demonstrate that this fluorogenic library can be utilized to assess accumulation through fluorescence‐based methods. Confirming previous observations with other chemotypes, small structural alterations can lead to meaningful changes in accumulation profiles. Future work will determine the accumulation profiles of the entire library to define motif‐driven effects on OM penetration and efflux avoidance. Additionally, this library now provides a compound collection that can be leveraged to interrogate accumulation kinetics across various bacterial species of interest using our varied efflux and permeation isogenic strain sets [8, 34, 35].

## Author Contributions

**Cole M. Friederichs**, **Jackson W. Berryman**, and **Quentin M.R. Gibaut** and **Chloe F. Cox** designed, synthesized, and structurally characterized all molecules. **Cole M. Friederichs** carried out all computational studies. **Cole M. Friederichs**, **Jackson W. Berryman,** and **Chloe F. Cox** carried out all fluorescence characterization. **Inga V. Leus** completed accumulation assays.**Cole M. Friederichs** wrote the original draft of the manuscript, which was then proofread and edited by all contributing authors. **Helen I. Zgurskaya** and **Adam S. Duerfeldt** contributed to conceptualization, study design, funding, resources, and supervision.

## Funding

This study was supported by National Institute of Allergy and Infectious Diseases .

## Conflicts of Interest

The authors declare no conflicts of interest.

## Supporting information

General experimental procedures, synthetic procedures, fluorescence parameters, library structures, characterization data, computational methods, NMR spectra, and spectral characterization of library members.
