# Supplementary material for: Synthesis and Fluorescence Profiling of Naphthylamine Analogs
Source: Chembiochem. 2026 Oct 9;27(19):e70569. doi: 10.1002/cbic.70569 (PMC13653121; doi:10.1002/cbic.70569)
Supplement: Supplementary file 1 [file CBIC-27-e70569-s001.pdf]

Supplementary Material

Synthesis and Fluorescence Profiling of Fluorescent N-phenylnaphthylamine Analogs

Cole Friederichs[a], Jackson W. Berryman[a], Quentin M.R. Gibaut[b], Inga V. Leus[b], Chloe F. Cox[a], Helen I. Zgurskaya[b], Adam S. Duerfeldt[a]

[a]*Department of Medicinal Chemistry, College of Pharmacy, University of Minnesota, Minneapolis, MN 55414*

[b]*Department of Chemistry and Biochemistry, University of Oklahoma, Norman OK, United States.*

Corresponding Author: Adam S. Duerfeldt – Department of Medicinal Chemistry, University of Minnesota, Minneapolis, MN 55454, United States; Phone: 612-624-6184; Email: aduerfel@umn.edu.

Table of Contents

Title	Page #
General experimental procedures	S2
Sulfonamide synthesis optimization (**Table A**)	S3
Fluorescence methodology and analysis (**Table B-E**) (**SI Figure 1**)	S4-6
Structures of all compounds by linker class (**SI Figures 2-7**)	S7-S11
Kinetic cell-association graphs (**SI Figures 8-12**)	S12-S14
General Synthetic methods	S15-S16
Characterization data	S17-S59
Computational methods, result, and coordinates (**Tables F and G**)	S60-S74
NMR Spectra	S75-S181
HPLC Traces of ALK-03, ALK-15, AR-01, and AR-04	S182-S183

General Experimental Procedures: Starting materials, ACS-grade methylene chloride (DCM), methanol (MeOH), hexanes, ethyl acetate (EtOAc), acetone, acetonitrile (CH_3CN), dimethyl formamide (DMF), dimethyl sulfoxide (DMSO), methylene chloride (DCM), tetrahydrofuran (THF), toluene, and trifluoroacetic acid (TFA) were purchased from Alfa Aesar, Fisher Scientific, or Sigma-Aldrich and used without further purification. Deionized water was used for all experimental procedures where "water" is indicated. All reactions requiring anhydrous conditions were run under a positive nitrogen atmosphere via balloon in flame dried glassware with anhydrous solvents unless stated otherwise. Flash column chromatography was carried out on silica gel (70–230 mesh, SiliCycle). Thin-layer chromatography (TLC) was performed on silica gel 60 F254 plates and visualized using a UV-lamp or with ninhydrin or basic $KMnO_4$ stain. NMR samples were prepared in 5 mm tubes with ~0.4 mL deuterated solvent ($CDCl_3$, CD_3CN, DMSO-d_6, acetone-d_6, and MeOD) and data was collected at 25 °C on a 400 MHz Bruker Avance, 600 MHz Bruker Avance NEO, or 850 MHz Bruker Avance III. Pulse sequences were used as supplied by Bruker Avance software. All NMR data were processed in MestreNova v14. Peak positions are reported after reference centering on deuterated solvent of relevance. High-resolution mass spectrometry was obtained on a Bruker BioTOF II ESI/TOF-MS and analyzed at the Mass Spectrometry Facility at the University of Minnesota.

Table A: Optimization conditions for the synthesis of sulfonamides

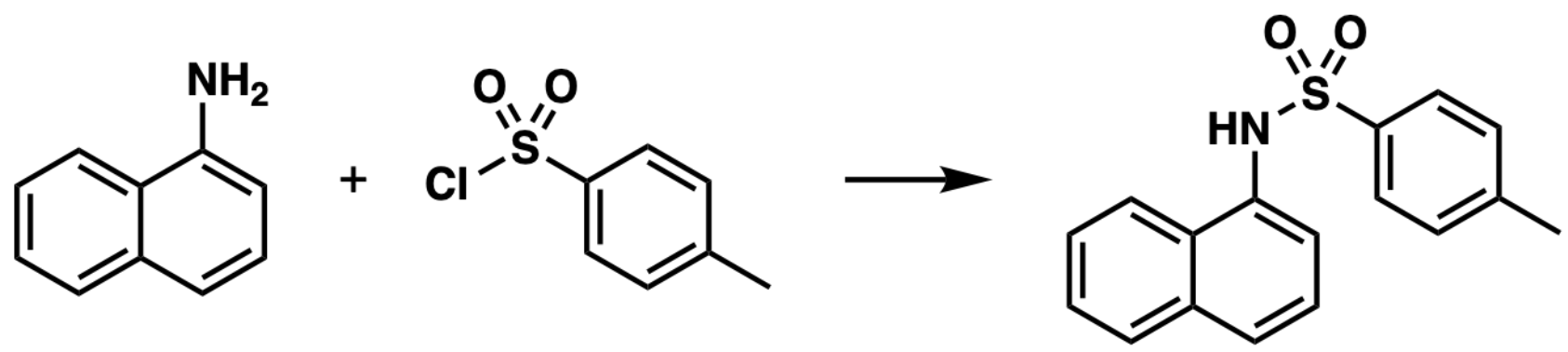

Entry	Base	Catalyst	Solvent	T (°C)	Yield
**1**	TEA (3 eq.)	-	THF	0 °C-rt	0
**2**	pyridine	DMAP (0.1eq)	pyridine	60 °C	0
**3**	K_2CO_3 (1 eq.)	-	solvent free	rt	0
**4**	Cs_2CO_3 (0.1 eq.)	-	MeCN	25 °C	0
**5**	KOtBu (1.5 eq.)	DMAP (0.2eq)	THF	reflux	0
**6**	-	CuO (0.05eq)	MeCN	rt	0
**7**	NaH (1 eq.)	-	DCM	rt	0
**8**	*n*-BuLi (1 eq.)	-	THF	rt	0
**9**	*n*-BuLi (1 eq.)	-	toluene	rt	37 %
**10**	*n*-BuLi (1 eq.)	-	DMF	rt	0
**11**	*n*-BuLi (1 eq.)	DMAP (0.5eq)	DMF	0 °C	0
**12**	*n*-BuLi (1 eq.)	-	THF	-78 °C	0
**13**	LDA (1.1 eq.)	-	THF	-78 °C	0
**14**	-	DABAL-Me_3	THF	40 °C-reflux	82%

Fluorescence Spectra: 5 mM stock solutions in DMSO of analog were prepared then diluted to 50 µM in HMG buffer. 100 µL of the 50 µM solution was added to 100 µL of 50 µg/mL solution of *E. coli* polar lipid extract (Avanti) in HMG buffer or 100 µL unaltered HMG buffer. Excitation and emission spectra were collected using a CLARIOstar plus plate reader in technical duplicate. The duplicates were averaged and then blank-corrected with the respective HMG buffer blank or lipid in buffer blank. Fold change was calculated at both the lipid spectrum max emission wavelength and the wavelength in which the greatest fold difference was observed. For RFU value lower than 0 and RFU of 5 was used in place to calculate the fold change. Compounds that exhibited fluorescence of less than 1000 RFU in the lipid sample were considered not fluorescent for further testing and were not included in the fluorogenic category. Parameters varied by linker (**TABLE B)**. Spectra were plotted in R.

Table B: Emission and excitation parameters for fluorescence spectra

Emission			
	AR	ALK	SA,UR,AM
Excitation	350 nm	350 nm	320 nm
Ex bandwidth	8 nm	8 nm	8 nm
Emission sweep	390-500 nm	390-500 nm	352-500 nm
Emission bandwidth	20 nm	20 nm	20 nm
Step Size	2 nm	2 nm	2 nm
Gain	1661	2000	2470
Flashes	40	40	40

Excitation			
	AR	ALK	SA,UR,AM
Emission	420 nm	420 nm	Too weak of signal
Em bandwidth	20 nm	20 nm	
Excitation sweep	320-370 nm	320-370 nm	
Excitation bandwidth	8 nm	8 nm	
Step Size	2 nm	2 nm	
Gain	2000	2000	
Flashes	40	40	

NPN Fluorescence in organic solvents: Solutions of 50 µM NPN were prepared in organic solvents from 50 mM DMSO stocks. Fluorescence spectra were collected on a Varian Cary Eclipse fluorescence spectrophotometer. Each sample contained 1 mL of solution measured at a gain of 400 eV.

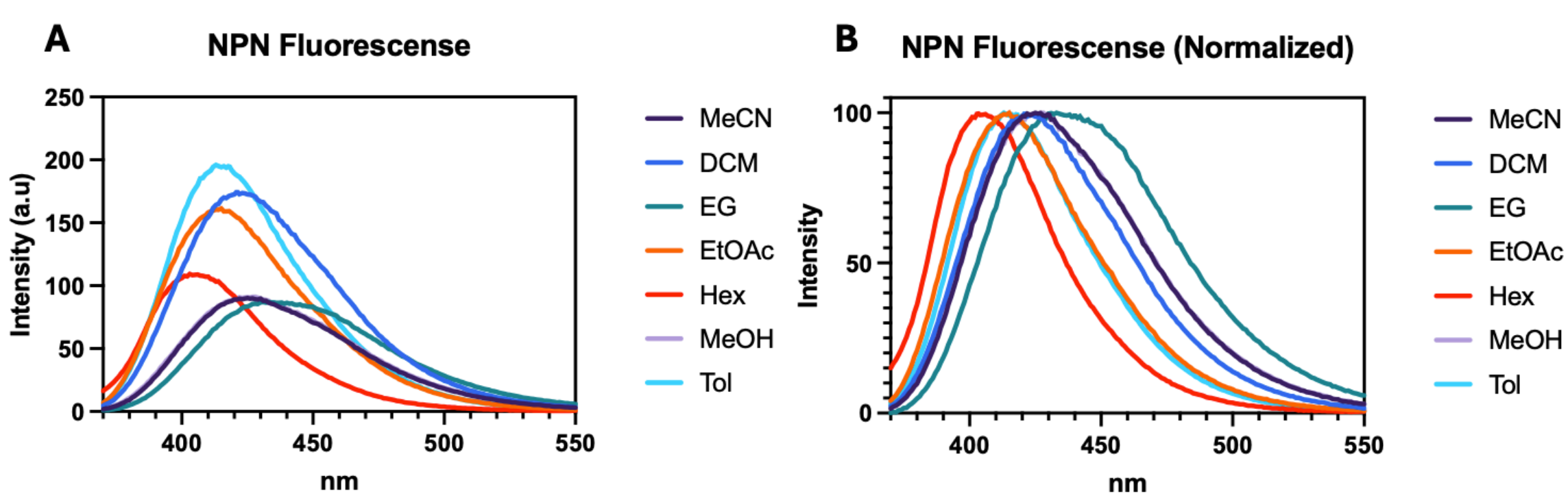

SI Figure 1: (A) Fluorescence emission spectra of NPN in organic solvents. (B) Normalized fluorescence emission spectra of NPN in organic solvents.

SI Table C: Max emission of NPN in organic solvents, relative signal intensity at max emission, and solvent properties

Solvent	λem (nm)	Relative Intensity (%)	Viscosity (cP)	Dielectric Constant
MeCN	425	45.9%	0.37	37.5
DCM	421	89.2%	0.42	9.0
EG	433	44.4%	16.10	37.7
EtOAc	415	82.3%	0.43	6.0
Hex	403	55.8%	0.29	1.9
MeOH	427	46.5%	0.54	33.0
Tol	413	100.0%	0.55	2.4

Viscosity Spectra and Correlation: Solutions of NPN (50 μM) were prepared in solutions of ethylene glycol and glycerol. The stock solutions were prepared by weight of solvent (5g total) in ratios of 0, 20, 40, 60, 80, and 100% glycerol. Solutions were heated to 80 °C and sonicated. 50 μL was removed, then 50 μL of 5 mM NPN in DMSO was added, and the solution was further mixed. 96-well plates were prepared for fluorescence testing by heating solutions to 80 °C and pipetting while hot to reduce inaccuracies when pipetting low-viscosity solutions. The plate was cooled to room temperature before measurements. Emission spectra were collected in triplicate on a CLARIOstar plate reader by exciting at 350 nm (8 nm bandwidth) and scanning emission 379 - 599 nm (20 nm bandwidth). Area under the curve was calculated for each replicate and then plotted on a Förster–Hoffmann plot using viscosities estimated from literature values.[1] Data was analyzed and plotted in GraphPad Prism.

Real time uptake. The experiment was carried out as described before for NPN[3] with small modifications. Briefly, overnight cells were subcultured (1:100) and grown for 1 hour in fresh LB medium, then induced with 0.1% arabinose and grown 3 more hours. Cells were collected by centrifugation, washed, and resuspended in 50 mM HEPES-KOH buffer (pH 7.0) containing 1 mM $MgSO_4$ and 0.4 mM glucose (HMG buffer) to an OD_{600} of ~1.0. The uptake assay was performed in a temperature-controlled multimode microplate reader (Tecan Spark 10M) equipped with a sample injector in fluorescence mode. The fluorescence of all compounds was monitored at λex-350 nm and λem-405 nm at a gain of 65 for 15 min. For each compound, the emission coefficients were determined using *E. coli* polar lipid fraction (Avanti Lipids) as described before. All measurements were collected in technical duplicate in biological triplicates with the exception of NPN for which 4 biological replicates were performed. This data was imported into MATLAB (MathWorks) to be fitted to a simple exponential equation in the form of $F = A1+A2(1-\exp(-kt))$ using emission coefficients specific for each compound.[4] The steady state data were plotted as a function of external concentration of compound.

SI Table D: *E. coli* strains used in uptake assay.

Strain	Relevant Genotype	Source
**GKCW101 (EC WT)**	BW25113 *att*Tn*7*::mini-Tn*7*T-Km^r -*araC* -P$_{araBAD}$ -MCS	[2]
**GKCW102 (EC WT-P)**	BW25113 *att*Tn*7*::mini-Tn*7*T-Km^r-*araC*-P$_{araBAD}$-*fhuA*Δ*C*/Δ*4L*	[2]
**GKCW103 (ECΔ)**	GD102 *att*Tn*7*::mini-Tn*7*T-Km^r -*araC* -P$_{araBAD}$ -MCS	[2]
**GKCW104 (ECΔ-P)**	GD102 *att*Tn*7*::mini-Tn*7*T-Km^r -*araC*-P$_{araBAD}$-*fhuA*Δ*C*/Δ*4L*	[2]

Uptake statistics and analysis: The slopes obtained from the fluorescence-based uptake experiment plotting intracellular accumulation vs total compound added. Four independent biological replicates were analyzed for NPN; related analogs were done in biological triplicates. Outer membrane and efflux impact ratios were calculated using the resulting slopes and $\log_2$ transformed for statistical analysis. $\log_2$ ratios were compared to 0 using two-tailed one-sample t tests with Holm–Šídák correction for multiple comparisons. To determine whether the analogs differed from NPN, OM-impact and efflux-impact $\log_2$ ratios were analyzed separately using Brown–Forsythe and Welch one-way ANOVA followed by Dunnett's T3 multiple-comparisons test. Statistical analyses were performed in GraphPad Prism.

SI Table E: Mean accumulation slopes and impact ratio statistics

Compound	n	Mean accumulation slope				$\log_2$(PE/E) outer membrane			$\log_2$(PE/P) TolC efflux		
		Ec WT	Ec WT-Pore	Ec ΔtolC	Ec ΔtolC-Pore	mean	p vs 0	p vs NPN	mean	p vs 0	p vs NPN
NPN	4	0.0065 ± 0.0023	0.2431 ± 0.0784	0.2135 ± 0.0680	0.6399 ± 0.1218	1.62 ± 0.22	0.0006	—	1.44 ± 0.25	0.0013	—
AR-01	3	0.0105 ± 0.0069	0.3695 ± 0.0344	0.0554 ± 0.0158	0.7436 ± 0.1411	3.77 ± 0.17	0.0007	<0.0001 ****	0.99 ± 0.17	0.0101	0.1223 ns
AR-04	3	0.0256 ± 0.0025	0.2644 ± 0.0165	0.3418 ± 0.0351	0.4056 ± 0.0419	0.25 ± 0.02	0.0032	0.0031 **	0.61 ± 0.08	0.0050	0.0103 *
ALK-03	3	0.1410 ± 0.0090	0.3357 ± 0.0366	0.1808 ± 0.0126	0.6792 ± 0.0971	1.90 ± 0.15	0.0020	0.2829 ns	1.01 ± 0.35	0.0380	0.4275 ns
ALK-15	3	0.0615 ± 0.0045	0.1495 ± 0.0217	0.0589 ± 0.0123	0.4602 ± 0.0278	2.98 ± 0.21	0.0017	0.0014 **	1.63 ± 0.19	0.0044	0.674 ns

References:

[1] M.-L. Ge, J.-L. Ma, B. Chu, "Densities and Viscosities of Propane-1,2,3-triol + Ethane-1,2-diol at T = (298.15 to 338.15) K" *J. Chem. Eng. Data* **2010**, *55*, 2649–2651.

[2] G. Krishnamoorthy, D. Wolloscheck, J. W. Weeks, C. Croft, V. V. Rybenkov, H. I. Zgurskaya, "Breaking the Permeability Barrier of Escherichia coli by Controlled Hyperporination of the Outer Membrane" *Antimicrob Agents Chemother* **2016**, *60*, 7372–7381.

[3] I. V. Leus, H. I. Zgurskaya, "No two are alike: on the role of *Klebsiella pneumoniae* permeability barriers in antibiotic susceptibility and persistence" *Antimicrob Agents Chemother* **2025**, *69*, e00085-25.

[4] D. A. Westfall, G. Krishnamoorthy, D. Wolloscheck, R. Sarkar, H. I. Zgurskaya, V. V. Rybenkov, "Bifurcation kinetics of drug uptake by Gram-negative bacteria" *PLoS ONE* **2017**, *12*, e0184671.

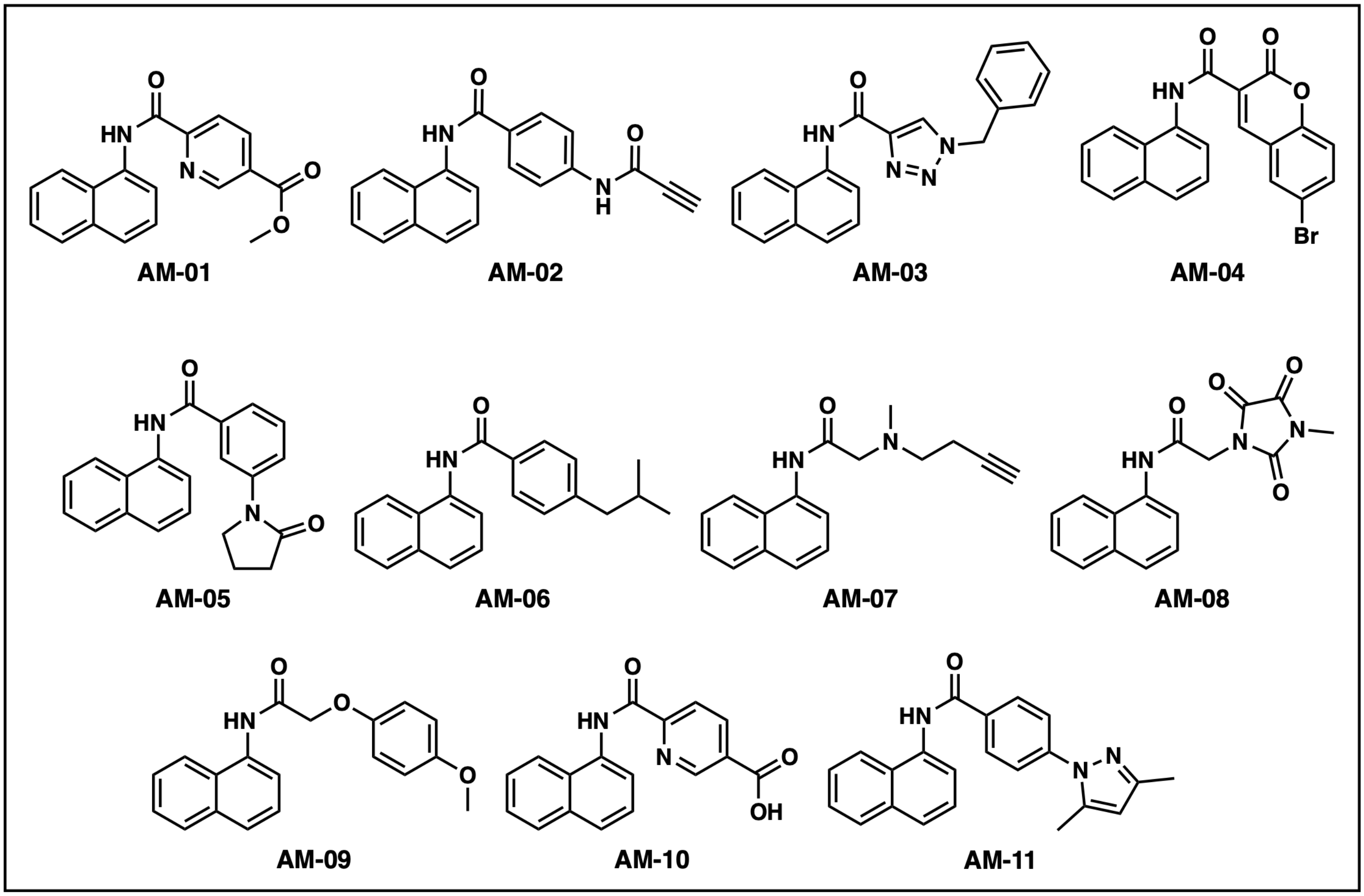

SI Figure 2: Amide series structures

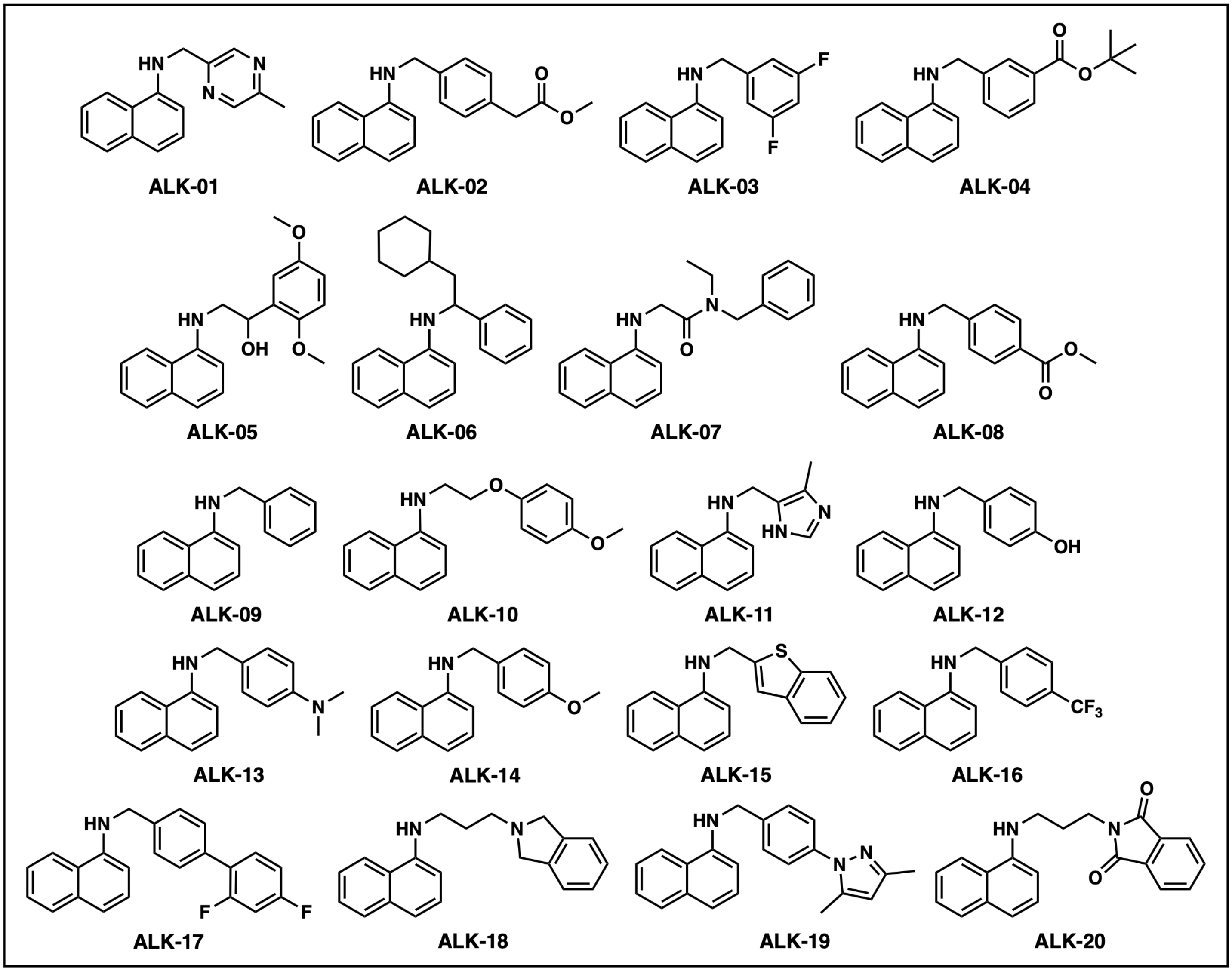

SI Figure 3: Alkyl series structures

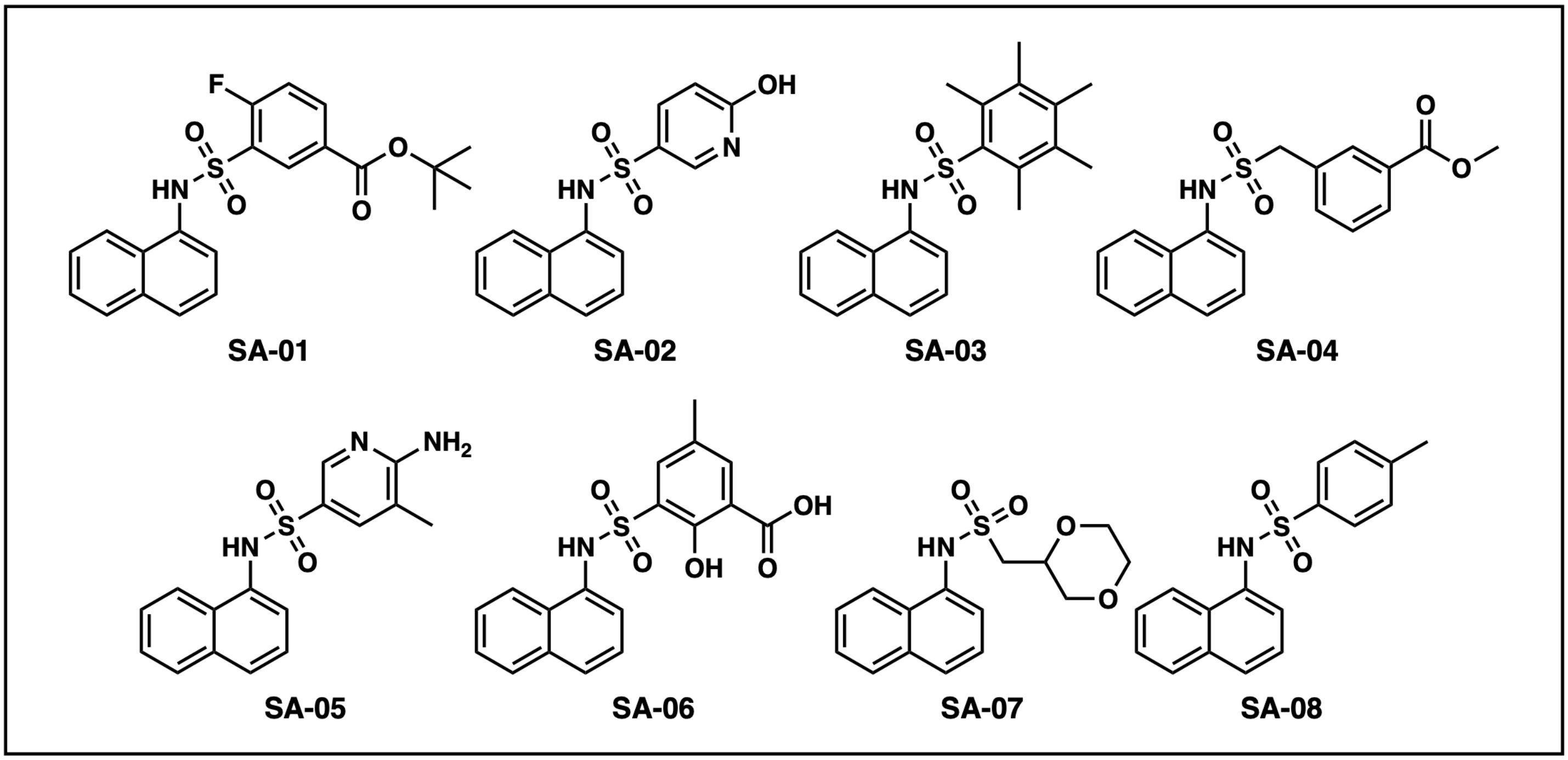

SI Figure 4: Sulfonamide series structures

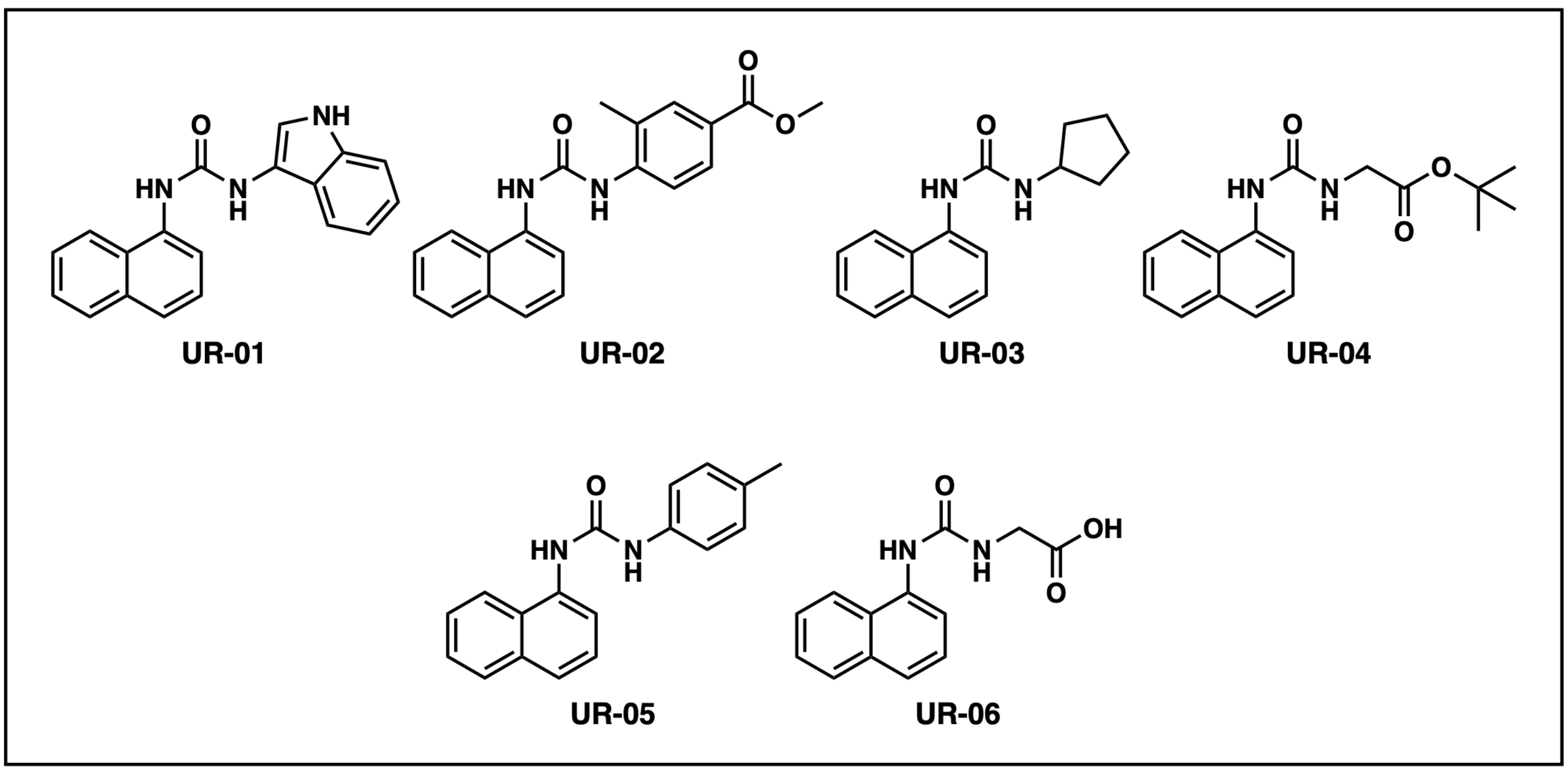

SI Figure 5: Urea series structures

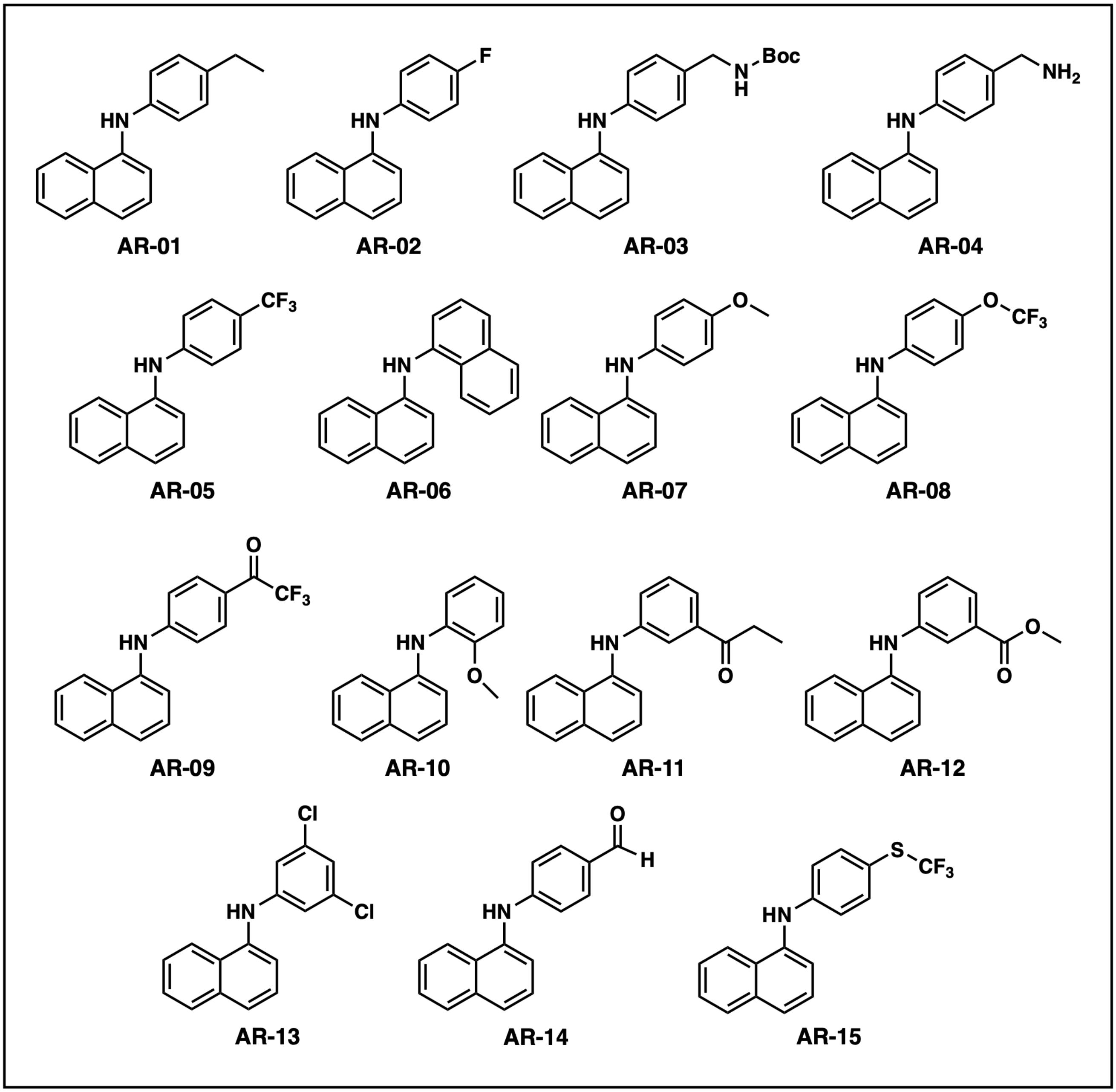

SI Figure 6: First generation Aryl series structures

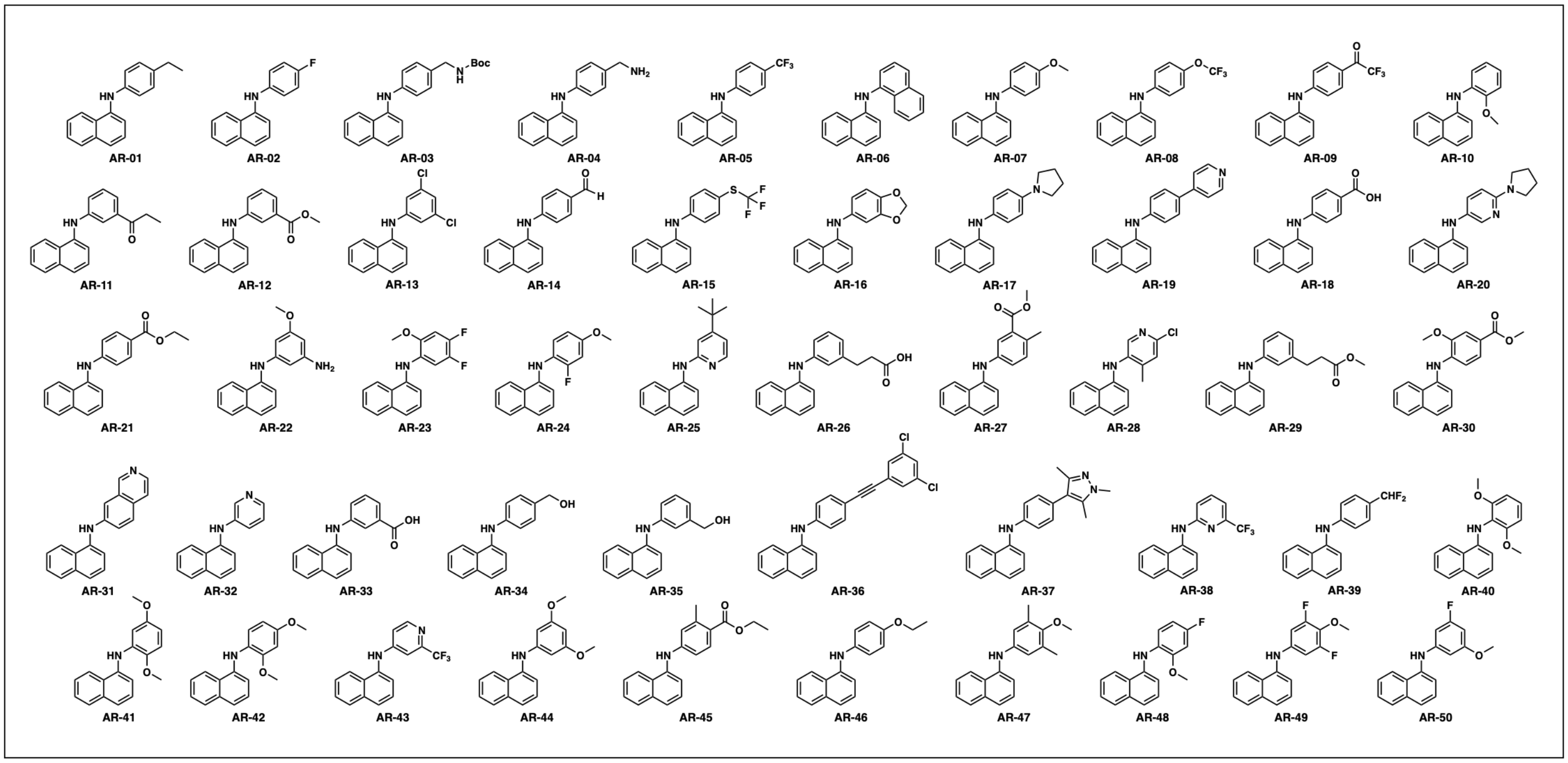

SI Figure 7: Full Aryl series structures

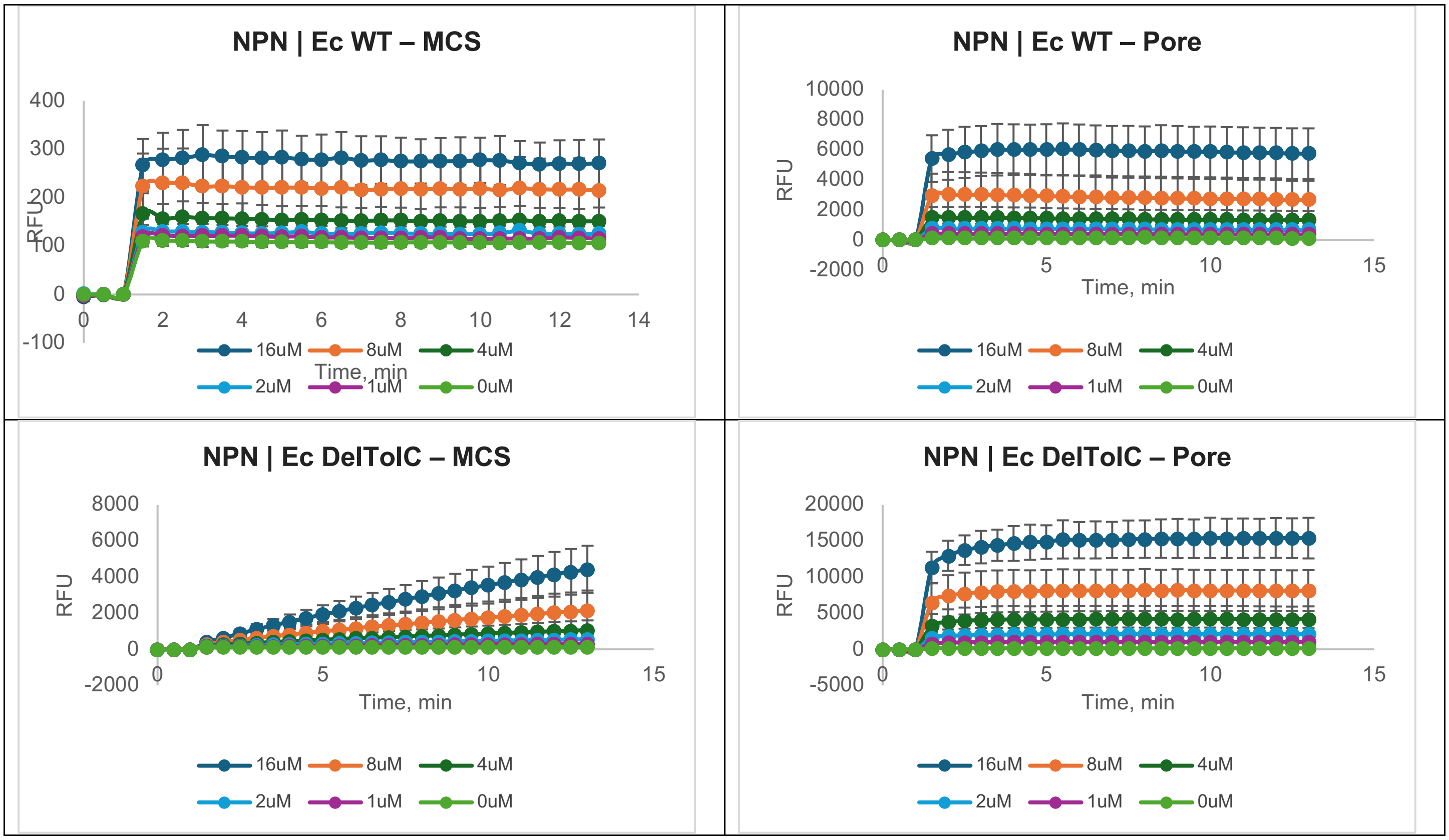

SI Figure 8: Kinetic cell-associated graphs for NPN

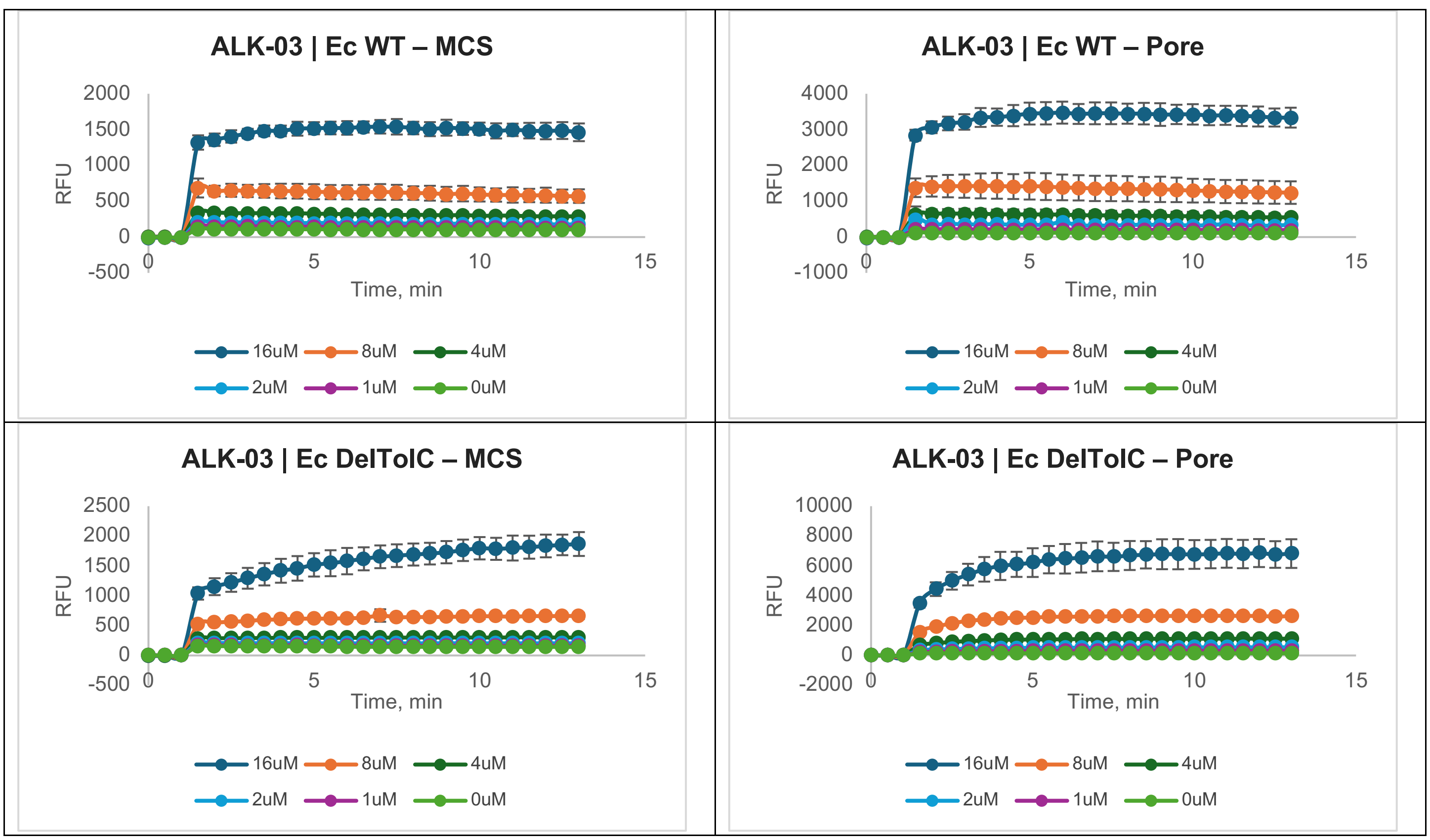

SI Figure 9: Kinetic cell-associated graphs for ALK-03

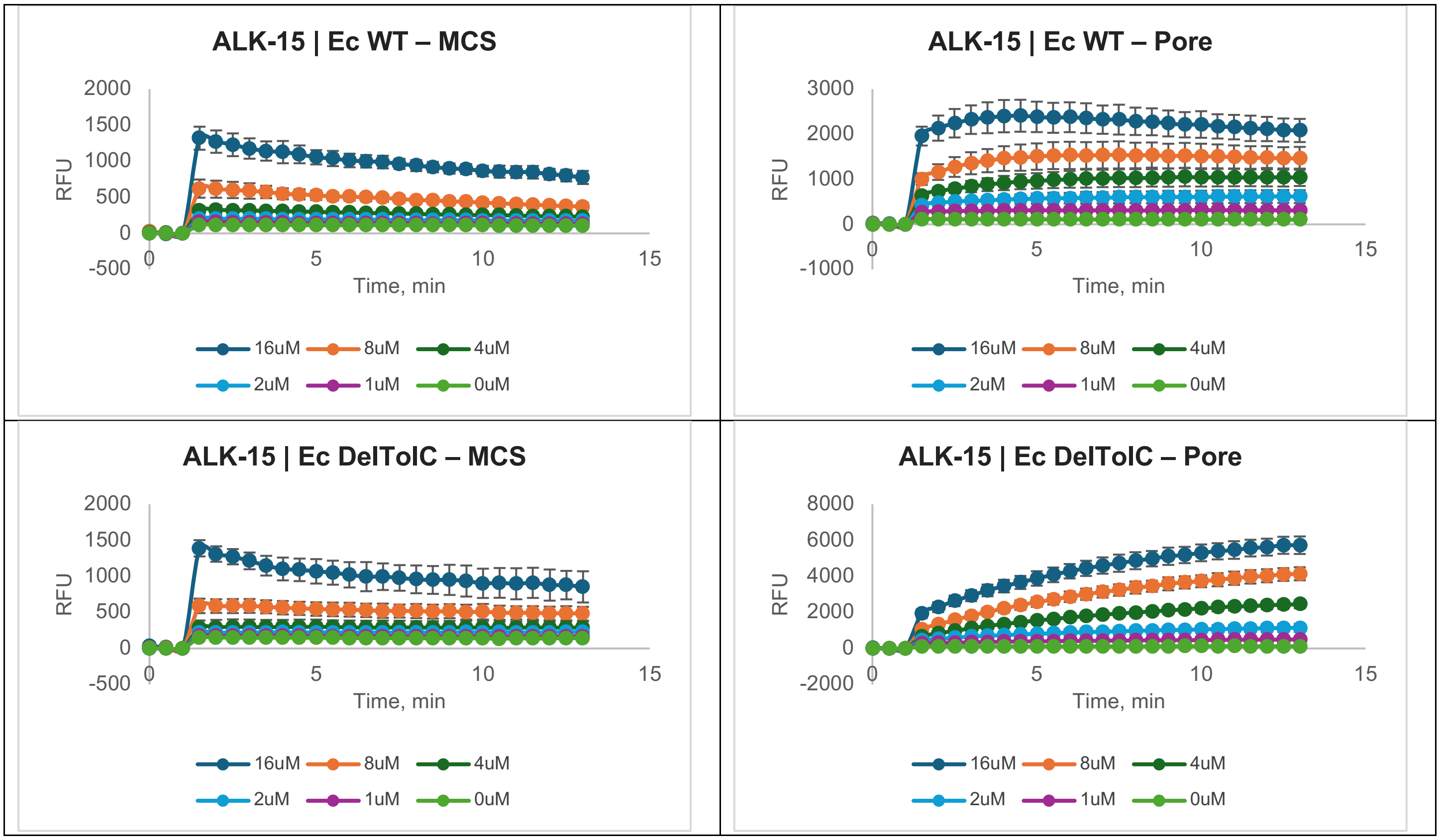

SI Figure 10: Kinetic cell-associated graphs for ALK-15

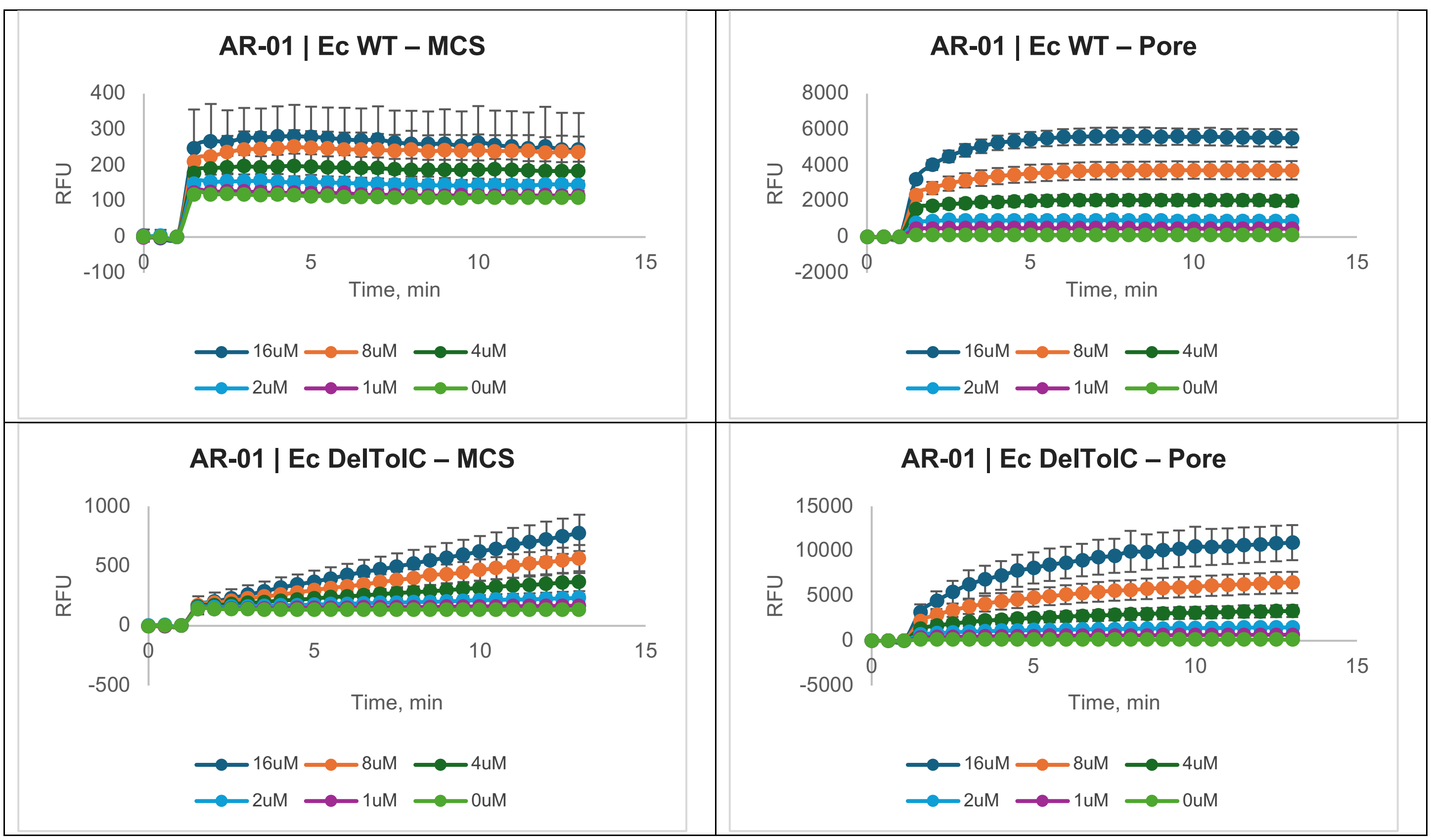

SI Figure 11: Kinetic cell-associated graphs for AR-01

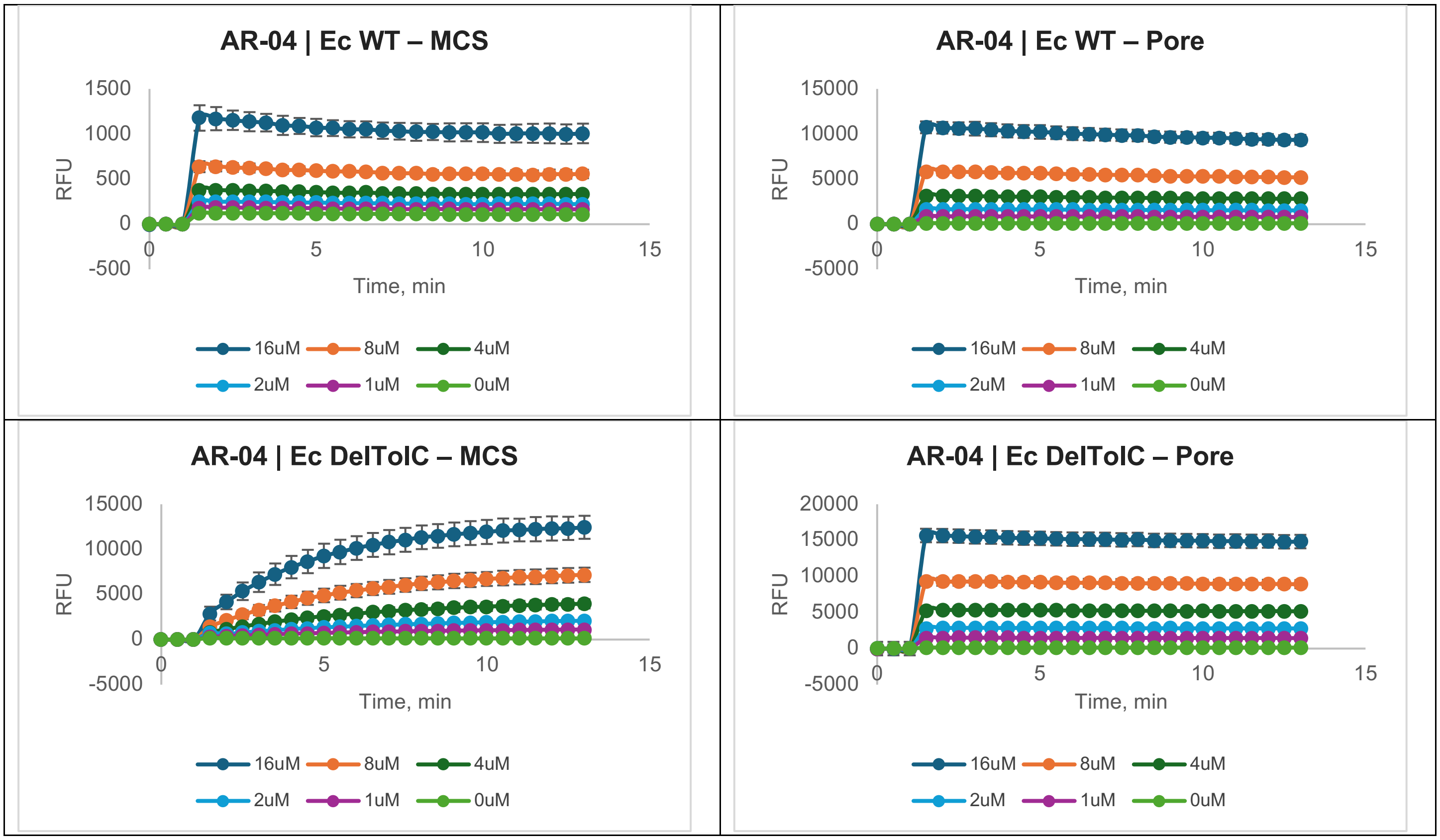

SI Figure 12: Kinetic cell-associated graphs for AR-04

General Procedures A: Amidation

To a solution of amine (1.0 eq.), carboxylic acid (1.5 eq.) and HATU (1.5 eq.) in dry DMF at 0 °C was added DIPEA (3.0 eq.). The reaction mixture was stirred for 4 - 12 h and then quenched with a saturated aqueous solution of $NaHCO_3$ and diluted with ethyl acetate. The aqueous layer was extracted with ethyl acetate (3 times) and the organic layers were combined and washed with H_2O (3 times) and then a saturated aqueous solution of NaCl before being dried over anhydrous Na_2SO_4 and filtering. Volatiles were removed by rotary evaporation and the crude product was purified by flash column chromatography (SiO_2), pTLC, or organic solvent washes (i.e., organic solvent was added into the vial and mixed with the solid before decanting).

General Procedures B: Saponification

The ester (1.0 eq.) was dissolved in a mixture of THF/MeOH/H_2O (3:1:2) and LiOH (7.0 eq.) was added. The reaction mixture was stirred at room temperature 12 h. The solvents were evaporated and the residue was acidified with 1 M aqueous HCl to pH 1 before being extracted with ethyl acetate, dried over anhydrous Na_2SO_4 and filtered. The organic solvent was evaporated and no purification was needed.

General Procedure C: Synthesis of Ureas

To a solution of amine (1.0 eq.) in dry THF at 0 °C was added TEA (3.0 eq.) and the reaction was stirred for 15 min before the isocyanate (1.0 eq.) was added. The reaction mixture was stirred for 4 – 12 h and then quenched with a saturated aqueous solution of $NaHCO_3$ and diluted with ethyl acetate. The aqueous layer was extracted with ethyl acetate (3 times) and the organic layers were combined and washed with H_2O (3 times) and then with a saturated aqueous solution of NaCl before being dried over anhydrous Na_2SO_4 and filtering. Volatiles were removed by rotary evaporation and the crude product was purified by flash column chromatography (SiO_2) or an organic solvent wash (the organic solvent was added into the vial and mixed with the solid before decanting).

General Procedure D: Synthesis of Sulfonamides

A solution of 1-naphthylamine (1.5 eq.) and DABAL-Me3 (1.5 eq.) in dry THF was allowed to stir for 1 h at 40 °C under nitrogen. Sulfonyl chloride (1.0 eq.) was then added and the reaction mixture was stirred overnight at reflux. The solution was cooled to room temperature, quenched with aqueous 1 M HCl and diluted with ethyl acetate. The aqueous layer was extracted with ethyl acetate (3 times) and the organic layers were combined and washed with H2O (3 times) and then with a saturated aqueous solution of NaCl before being dried over anhydrous Na2SO4 and filtering. Volatiles were removed by rotary evaporation and the crude product was purified by flash column chromatography (SiO2), pTLC, or organic solvent washes (the organic solvent was added into the vial and mixed with the solid before decanting).

General Procedure E: Reductive Amination

A solution of 1-naphthylamine (1.0 eq.) and DABAL-Me_3 (1.0 eq.) in dry THF was allowed to stir for 1 h at 40 °C under nitrogen. Aldehyde (1.0 eq.) was then added and the reaction mixture was stirred 2 – 12 h at reflux until imine formation is complete. The solution was cooled to room temperature and the reductive agent $LiAlH_4$ or $NaCNBH_3$ (1.5 eq.) was added. The solution was stirred again 2 – 12 h until the reaction is complete, then quenched dropwise with a saturated aqueous solution of $NaHCO_3$ and diluted with ethyl acetate. The aqueous layer was extracted with ethyl acetate (3 times) and the organic layers were combined and washed with H_2O (3 times) and then with a saturated aqueous solution of NaCl before being dried over anhydrous Na_2SO_4 and filtering. Volatiles were removed by rotary evaporation and the crude product was purified by flash column chromatography (SiO_2) or pTLC.

General Procedure F: S_N2 Amination

A solution of 1-naphthylamine (1.2 eq.) and Cs_2CO_3 (or Ag2CO3) (1.2 eq.) in DMF was stirred at 30 °C for 1 h and alkyl halide (1.0 eq.) was added dropwise. The reaction mixture was stirred 5 h at 60 °C and then quenched with a saturated aqueous solution of $NaHCO_3$ and diluted with ethyl acetate. The aqueous layer was extracted with ethyl acetate (3 times) and the organic layers were combined and washed with H_2O (3 times) and then with

a saturated aqueous solution of NaCl before being dried over anhydrous Na_2SO_4 and filtering. Volatiles were removed by rotary evaporation and the crude product was purified by flash column chromatography (SiO_2).

General Procedure G: Amide Reduction

To a solution of amide (1.0 eq.) in THF at room temperature was added $LiAlH_4$ (2 eq.). The reaction mixture was stirred 3 h and then quenched carefully with a saturated aqueous solution of $NaHCO_3$ and diluted with ethyl acetate. The aqueous layer was extracted with EtOAc (3 times) and the organic layers were combined and washed with H_2O (3 times) and then with a saturated aqueous solution of NaCl before being dried over anhydrous Na_2SO_4 and filtering. Volatiles were removed by rotary evaporation and the crude product was purified by flash column chromatography (SiO_2).

General Procedure H: Microwave Buchwald-Hartwig coupling

In a flame dried microwave vial, naphthalen-1-amine (1.0 Eq), aryl bromide (1.2 Eq), sodium tert-butoxide (1.2 Eq), were added, then the vial was capped The reagents were dissolved in anhydrous toluene (0.5 mmol) and N_2 was bubbled through the mixture for 30 minutes to degas solvent. BrettPhos Pd G4 (2.5 mol%) was added and the headspace was filled with N_2. The reaction was microwaved at 130 ºC for 10 minutes using a Biotage Initiator. The reaction was quenched with water and diluted with EtOAc. The crude product was filtered over celite and the organic layer was washed with brine. Purified compound was isolated by flash column chromatography (SiO_2).

General Procedure I: Buchwald-Hartwig coupling

In a flame dried microwave vial, naphthalen-1-amine (1.0 Eq), aryl bromide (1.25 Eq), sodium tert-butoxide (1.2 Eq), and Pd(allyl)(BrettPhos)Cl (1 mol%) were added, then the vial was sealed and headspace filled with N_2. After headspace had been flushed with N_2, reagents were dissolved in anhydrous toluene (0.2 mmol). The reaction was heated at 45 °C for two hours before being quenched with water and diluting with EtOAc. The reaction was filtered over celite and the organic layer was washed with brine. The crude product was purified by flash column chromatography (SiO_2) 0 to 40% EtOAc in Hexanes. The product was dissolved in minimal DCM and precipitated out as the hydrochloride salt with excess 1M HCl in Et_2O. The solvent was triturated off and product dried over vacuum.

Methyl 6-(naphthalen-1-ylcarbamoyl)nicotinate (AM-01)

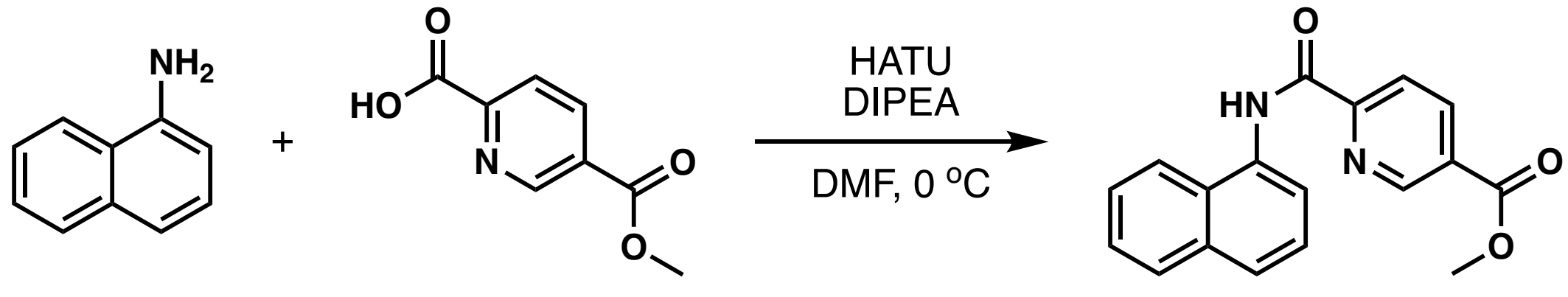

AM-01

General Procedure A: Reaction scale: 7.0 mg (0.049 mmol) of 1-naphthylamine. Purified by flash chromatography (SiO2, EtOAc/hexane, gradient from 40 to 70% in EtOAc) to afford AM-01 as a yellow solid (11.5 mg, 65%).

^{1}H NMR (400 MHz, DMSO-d_6) δ 11.03 (s, 1H), 9.25 (s, 1H), 8.57 (dd, J = 8.1, 2.1 Hz, 1H), 8.32 (d, J = 8.1 Hz, 1H), 8.03 – 7.94 (m, 2H), 7.86 (dd, J = 11.3, 7.8 Hz, 2H), 7.58 (dd, J = 9.3, 5.8 Hz, 3H), 3.96 (s, 3H).

^{13}C NMR (101 MHz, DMSO-d_6) δ 164.70, 153.03, 149.08, 139.03, 133.73, 132.96, 128.30, 128.05, 126.27, 126.23, 126.14, 125.67, 122.59, 122.40, 122.14, 52.84.

ESI-HRMS m/z: $[M+H]^+$ Calcd for $C_{18}H_{14}N_2O_3$ 307.1077; found 307.1071

***N*-(naphthalen-1-yl)-4-propiolamidobenzamide (AM-02)**

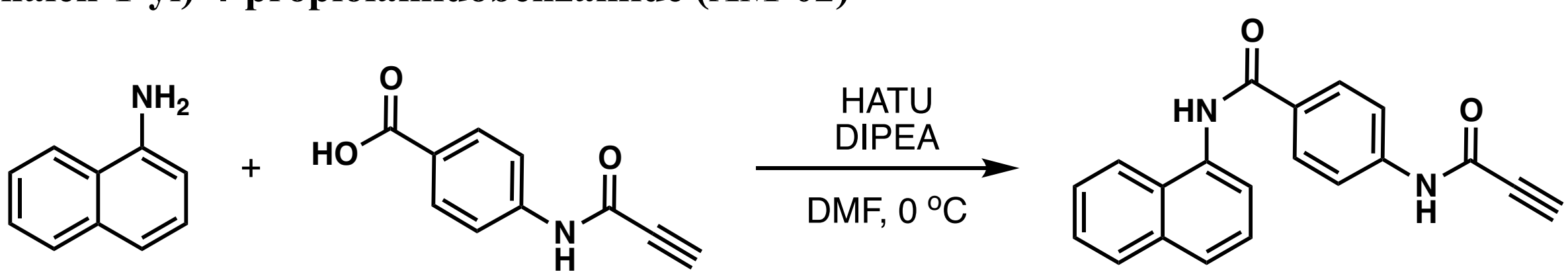

AM-02

General Procedure A: Reaction scale: 6.8 mg (0.048 mmol) of 1-naphthylamine. Purified by flash chromatography (SiO2, EtOAc/hexane, gradient from 20 to 90% in EtOAc) and hexane wash to afford AM-02 as a white/yellow solid (7.8 mg, 52%).

^{1}H NMR (500 MHz, Methanol-d_4) δ 8.06 (d, J = 8.4 Hz, 2H), 8.00 – 7.97 (m, 1H), 7.94 – 7.90 (m, 1H), 7.85 (d, J = 8.1 Hz, 1H), 7.79 (d, J = 8.5 Hz, 2H), 7.58 (d, J = 7.1 Hz, 1H), 7.55 – 7.50 (m, 3H), 2.81 (s, 1H).

^{13}C NMR (101 MHz, Methanol-d_4) δ 169.08, 152.41, 142.76, 135.81, 134.54, 131.33, 131.02, 129.87 (2C), 129.36, 128.28, 127.36, 127.22, 126.54, 125.28, 123.89, 120.71 (2C), 78.53, 77.01.

ESI-HRMS m/z: $[M+H]^+$ Calcd for $C_{20}H_{14}N_2O_2$ 315.1128; found 315.1123

1-benzyl-*N*-(naphthalen-1-yl)-1*H*-1,2,3-triazole-4-carboxamide (AM-03)

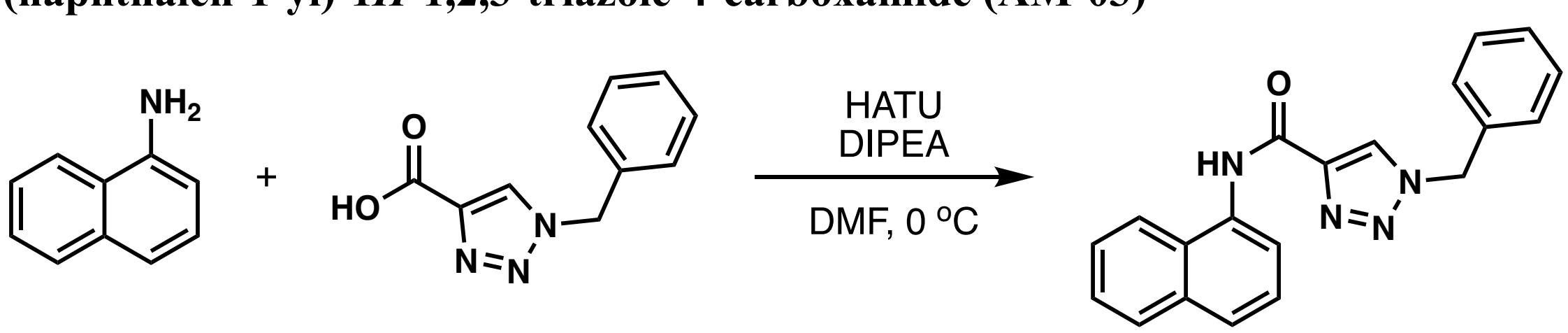

AM-03

General Procedure A: Reaction scale: 6.5 mg (0.046 mmol) of 1-naphthylamine. Crude solid was purified by methanol washes to afford AM-03 as a white solid (11.8 mg, 79%).

^{1}H NMR (400 MHz, DMSO-d_6) δ 10.58 (s, 1H), 8.87 (s, 1H), 7.96 (m, 2H), 7.86 (d, J = 8.2 Hz, 1H), 7.62 (d, J = 7.3 Hz, 1H), 7.55 (m, 3H), 7.42 (m, 4H), 7.37 (m, 1H), 5.73 (s, 2H), 2.81 (s, 1H) ppm.

^{13}C NMR (101 MHz, DMSO-d_6) δ 159.2, 142.9, 135.7, 133.8, 133.1, 129.4, 128.9 (2C), 128.6, 128.5, 128.2 (2C), 128.0, 127.7, 126.4, 126.2, 125.78, 123.2, 123.0, 53.3 ppm.

ESI-HRMS m/z: $[M+H]^+$ Calcd for $C_{20}H_{16}N_4O$ 329.1397; found 329.1392

6-bromo-*N*-(naphthalen-1-yl)-2-oxo-2*H*-chromene-3-carboxamide (AM-04)

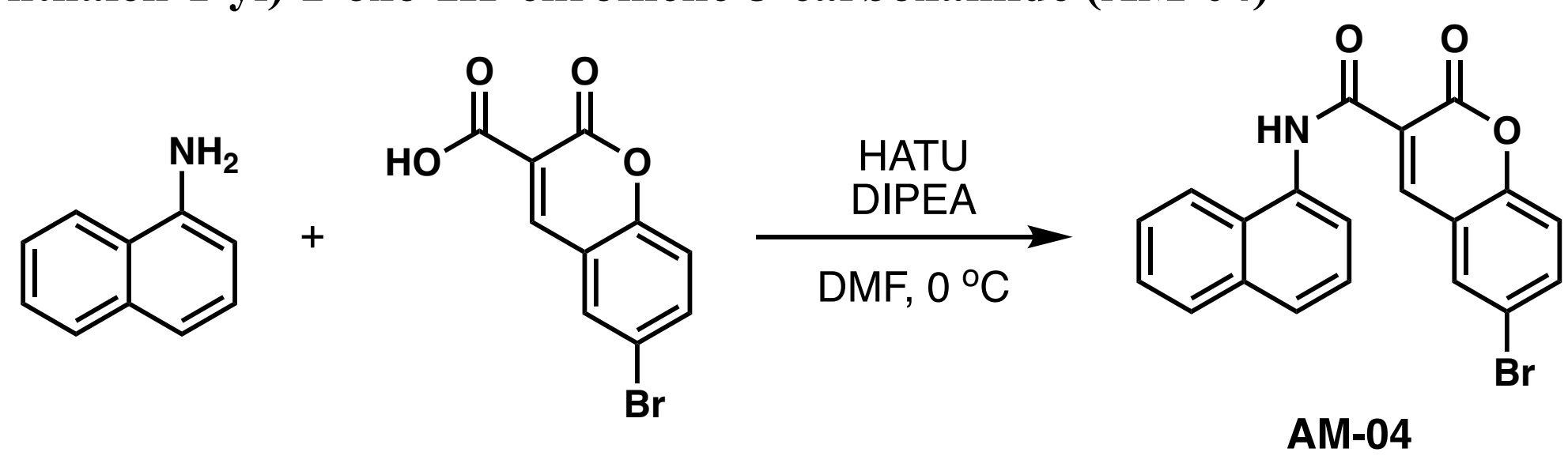

AM-04

General Procedure A: Reaction scale: 5.4 mg (0.038 mmol) of 1-naphthylamine. Crude solid was purified by methanol washes to afford AM-04 as a yellow solid (14.4 mg, 97%)

^{1}H NMR (400 MHz, DMSO-d_6) δ 11.21 (s, 1H), 9.01 (s, 1H), 8.34 (s, 1H), 8.27 (d, J = 7.5 Hz, 1H), 8.10 (d, J = 8.4 Hz, 1H), 8.01 (d, J = 8.1 Hz, 1H), 7.97 – 7.93 (m, 1H), 7.81 (d, J = 8.2 Hz, 1H), 7.68 (dd, J = 7.6, 7.6 Hz, 1H), 7.63 – 7.55 (m, 3H).

^{13}C NMR (101 MHz, DMSO-d_6) δ 161.01, 159.77, 152.98, 146.77, 136.65, 133.70, 132.64, 132.33, 128.76, 126.74, 126.43, 126.38, 125.93,125.90, 125.26, 120.70, 120.52, 118.94, 118.63, 116.96.

ESI-HRMS m/z: $[M+H]^+$ Calcd for $C_{20}H_{12}NO_3Br$ 394.0073; found 394.0070

***N*-(naphthalen-1-yl)-3-(2-oxopyrrolidin-1-yl)benzamide (AM-05)**

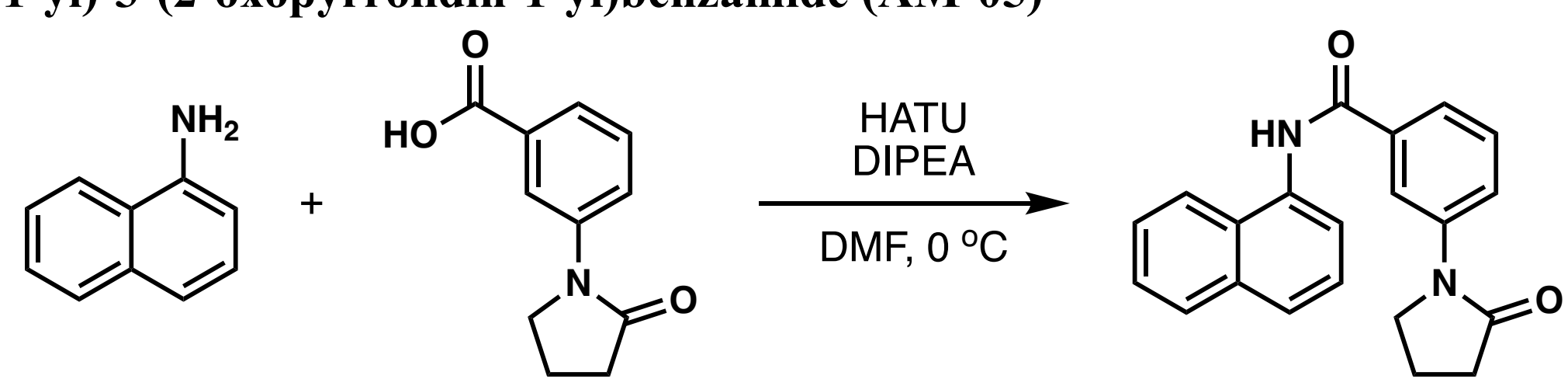

AM-05

General Procedure A: Reaction scale: 6.5 mg (0.045 mmol) of 1-naphthylamine. Crude solid was purified by methanol washes to afford AM-05 as a white solid (5.0 mg, 33%).

^{1}H NMR (400 MHz, DMSO-d_6) δ 10.48 (s, 1H), 8.22 (s, 1H), 8.04 – 7.94 (m, 3H), 7.91 – 7.83 (m, 2H), 7.63 – 7.51 (m, 5H), 3.94 (t, J = 7.0 Hz, 2H), 2.54 (t, J = 8.0 Hz, 2H), 2.10 (tt, J = 7.7, 7.6 Hz, 2H).

^{13}C NMR (101 MHz, DMSO-d_6) δ 174.15, 165.98, 139.79, 134.98, 133.81 (2C), 129.22, 128.82, 128.14, 126.40, 126.14, 126.05, 125.61, 123.99, 123.36, 123.07, 122.63, 118.68, 48.20, 32.37, 17.50.

ESI-HRMS m/z: $[M+H]^+$ Calcd for $C_{21}H_{18}N_2O_2$ 331.1441; found 331.1436

4-isobutyl-*N*-(naphthalen-1-yl)benzamide (AM-06)

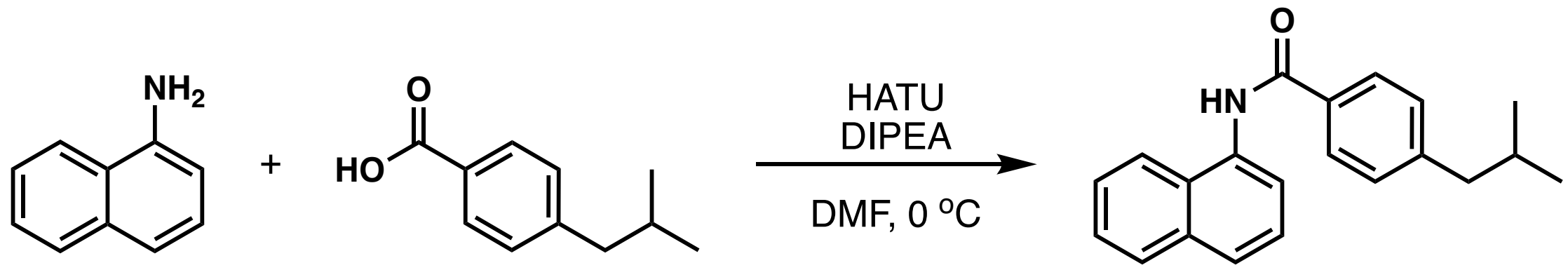

AM-06

General Procedure A: Reaction scale: 7.1 mg (0.049 mmol) of 1-naphthylamine. Purified by flash chromatography (SiO2, EtOAc/hexane, gradient from 20 to 50% in EtOAc) to afford AM-06 as a white solid (11.4 mg, 76%).

^{1}H NMR (400 MHz, Acetone-d_6) δ 9.60 (s, 1H), 8.16 – 8.11 (m, 1H), 8.08 (d, J = 8.2 Hz, 2H), 7.99 – 7.91 (m, 1H), 7.82 (d, J = 7.9 Hz, 2H), 7.58 – 7.49 (m, 3H), 7.36 (d, J = 8.2 Hz, 2H), 2.60 (d, J = 7.2 Hz, 2H), 1.94 (dq, J = 13.6, 6.9 Hz, 1H), 0.94 (d, J = 6.6 Hz, 6H).

^{13}C NMR (101 MHz, Acetone-d_6) δ 166.89, 146.60, 135.28, 135.06, 133.62, 130.11 (2C), 130.01, 129.18, 128.58 (2C), 126.89 (2C), 126.80, 126.46, 123.83, 123.77, 45.80, 31.05, 22.69 (2C).

ESI-HRMS m/z: $[M+H]^+$ Calcd for $C_{21}H_{21}NO$ 304.1696; found 304.1691

2-(but-3-yn-1-yl(methyl)amino)-N-(naphthalen-1-yl)acetamide (AM-07)

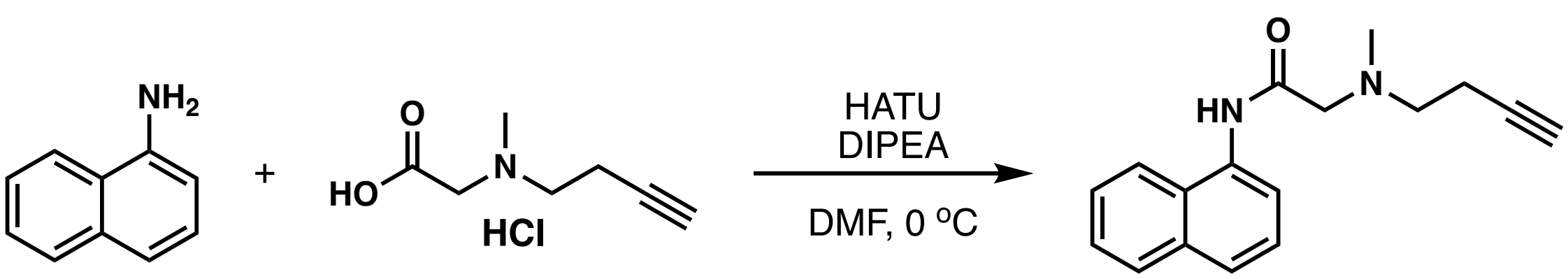

AM-07

General Procedure A: Reaction scale: 8.1 mg (0.056 mmol) of 1-naphthylamine. Purified by pTLC (EtOAc/hexane, 20:80) to afford AM-07 as a white/yellow solid (8.1 mg, 54%).

^{1}H NMR (400 MHz, Acetone-d_6) δ 8.26 (dd, J = 7.1, 5.1 Hz, 1H), 8.07 (d, J = 8.2 Hz, 1H), 7.94 (d, J = 7.8 Hz, 1H), 7.70 (d, J = 8.2 Hz, 1H), 7.56 (dt, J = 15.3, 6.8 Hz, 2H), 7.49 (t, J = 7.9 Hz, 1H), 3.32 (s, 2H), 2.86 (t, J = 6.8 Hz, 2H), 2.57 (td, J = 7.1, 3.6 Hz, 2H), 2.54 (s, 3H), 2.39 (t, J = 2.5 Hz, 1H).

^{13}C NMR (101 MHz, Acetone-d_6) δ 169.45, 135.02, 134.11, 129.43, 126.88, 126.79, 126.59, 125.03, 121.71, 118.64, 118.51, 83.51, 71.04, 62.56, 57.05, 43.05, 18.17.

ESI-HRMS m/z: $[M+H]^+$ Calcd for $C_{17}H_{18}N_2O$ 267.1492; found 267.1490

2-(3-methyl-2,4,5-trioxoimidazolidin-1-yl)-*N*-(naphthalen-1-yl)acetamide (AM-08)

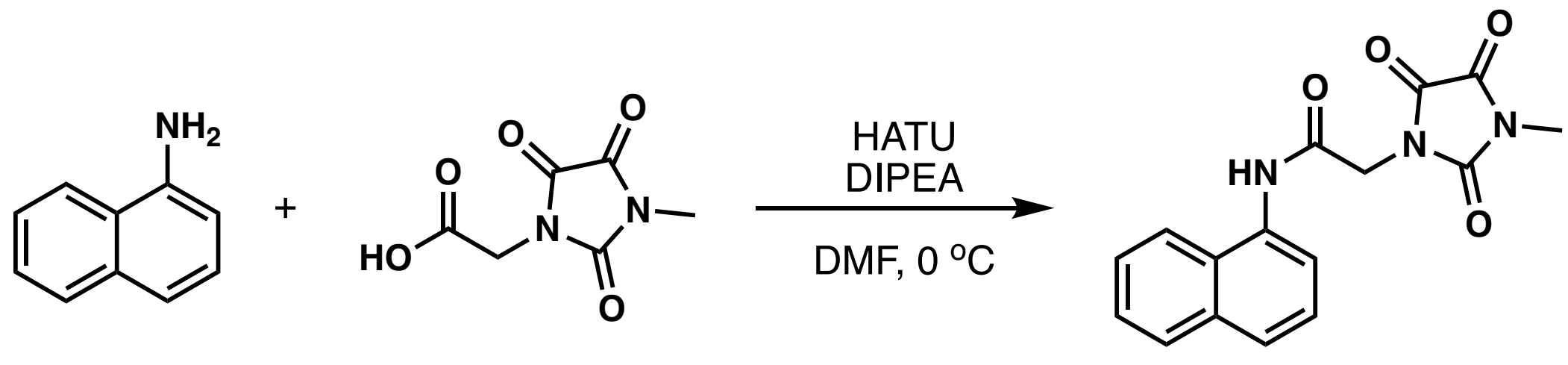

AM-08

General Procedure A: Reaction scale: 6.9 mg (0.048 mmol) of 1-naphthylamine. Purified by flash chromatography (SiO2, EtOAc/hexane, gradient from 40 to 90% in EtOAc) and hexane wash to afford AM-08 as a white/yellow solid (5.6 mg, 37%).

^{1}H NMR (400 MHz, Acetone-*d6*) δ 9.58 (s, 1H), 8.13 (m, 1H), 7.94 (m, 1H), 7.80 (m, 2H), 7.55 (t, J = 3.9 Hz, 2H), 7.50 (t, J = 7.9 Hz, 1H), 4.72 (s, 2H), 3.17 (s, 3H) ppm.

^{13}C NMR (101 MHz, Acetone-d_6) δ 165.41, 158.16, 158.13, 155.07, 135.12, 133.71, 129.20, 128.88, 126.95 (2C), 126.85, 126.36, 123.00, 122.58, 42.30, 25.01.

ESI-HRMS m/z: $[M+H]^+$ Calcd for $C_{16}H_{13}N_3O_4$ 312.0979; found 312.0975

2-(4-methoxyphenoxy)-*N*-(naphthalen-1-yl)acetamide (AM-09)

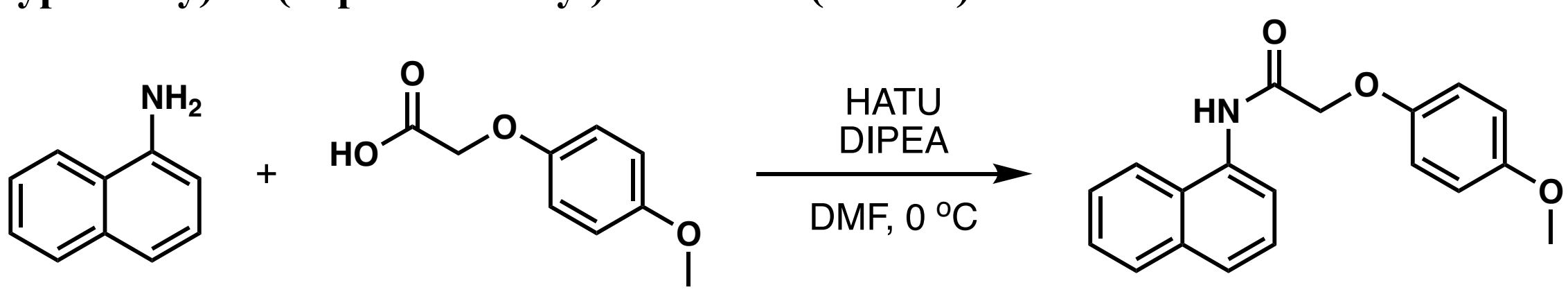

AM-09

General Procedure A: Reaction scale: 28.6 mg (0.200 mmol) of 1-naphthylamine. Purified by flash chromatography (SiO2, EtOAc/hexane, gradient from 30 to 60% in EtOAc) to afford AM-09 as a white solid (61.5 mg, 100%).

^{1}H NMR (300 MHz, Acetone-d_6) δ 9.41 (s, 1H), 7.92 (dd, J = 12.7, 5.8 Hz, 3H), 7.79 (d, J = 8.1 Hz, 1H), 7.51 (t, J = 7.4 Hz, 3H), 7.11 (d, J = 8.6 Hz, 2H), 6.94 (d, J = 8.7 Hz, 2H), 4.75 (s, 2H), 3.77 (s, 3H).

^{13}C NMR (75 MHz, Acetone-d_6) δ 167.98, 155.66, 152.75, 135.07, 133.61, 129.19, 128.79, 126.90, 126.85, 126.57, 126.36, 122.69, 122.28, 116.73 (2C), 115.53 (2C), 69.16, 55.83.

ESI-HRMS m/z: $[M+H]^+$ Calcd for $C_{19}H_{17}NO_3$ 308.1281; found 308.1277

6-(naphthalen-1-ylcarbamoyl)nicotinic acid (AM-10)

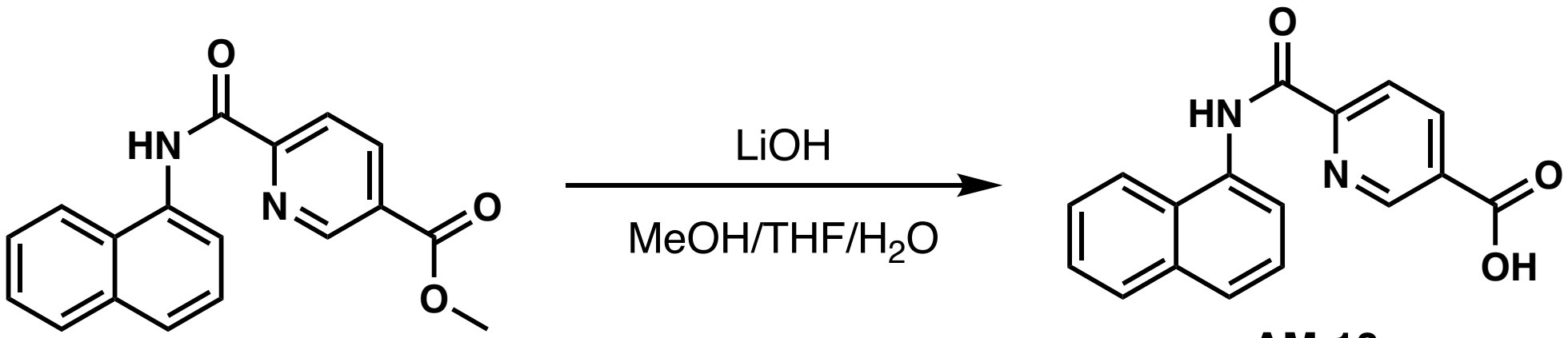

AM-10

General Procedures B: Reaction scale: 7.0 mg (0.023 mmol) of AM-01. No purification was needed and AM-10 was afforded as a white/yellow solid (2.7 mg, 40%).

^{1}H NMR (400 MHz, Methanol-d_4) δ 9.33 (s, 1H), 8.60 (d, *J* = 8.1 Hz, 1H), 8.35 (d, *J* = 8.0 Hz, 1H), 8.06 (d, *J* = 8.1 Hz, 1H), 8.02 (d, *J* = 7.4 Hz, 1H), 7.95 (d, *J* = 7.8 Hz, 1H), 7.82 (d, *J* = 8.3 Hz, 1H), 7.64 – 7.51 (m, 3H).

^{13}C NMR (101 MHz, Methanol-d_4) δ 166.80, 164.31, 154.78, 151.04, 140.30, 135.71, 133.57, 129.66, 129.62, 129.13, 127.57, 127.43, 127.32, 126.64, 123.22, 122.50, 122.20.

ESI-HRMS m/z: $[M+H]^+$ Calcd for $C_{17}H_{12}N_2O_3$ 293.0921; found 293.0922

4-(3,5-dimethyl-1H-pyrazol-1-yl)-N-(naphthalen-1-yl)benzamide (AM-11)

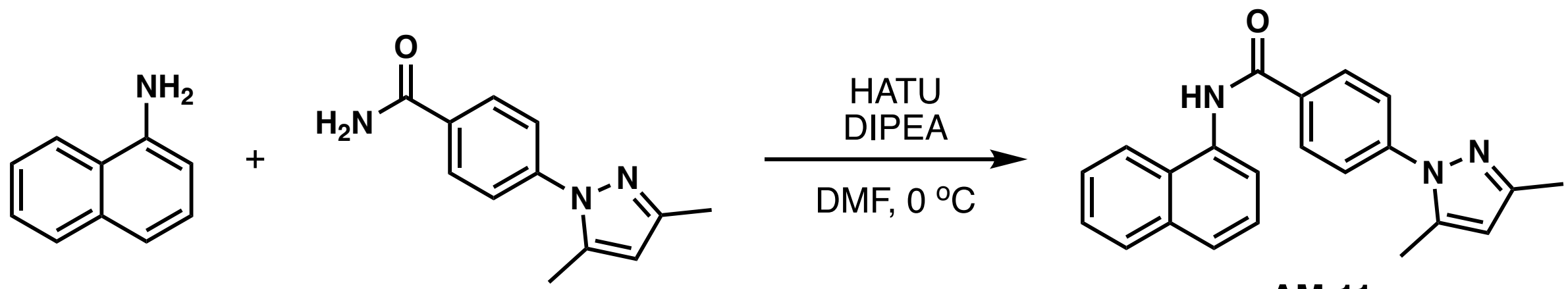

AM-11

General Procedure A: Reaction scale: 28.6 mg (0.200 mmol) of 1-naphthylamine. Purified by flash chromatography (SiO_2, EtOAc/hexane, gradient from 20 to 60% in EtOAc) to afford AM-11 as a white/brown solid (48.6 mg, 72%).

^{1}H NMR (500 MHz, Acetone-d_6) δ 9.74 (s, 1H), 8.26 (d, *J* = 8.5 Hz, 2H), 8.16 (dd, *J* = 6.3, 3.3 Hz, 1H), 7.96 (dd, *J* = 6.2, 3.2 Hz, 1H), 7.84 (d, *J* = 7.8 Hz, 2H), 7.72 (d, *J* = 8.5 Hz, 2H), 7.57 – 7.52 (m, 3H), 6.10 (s, 1H), 2.44 (s, 3H), 2.23 (s, 3H).

^{13}C NMR (126 MHz, Acetone-d_6) δ 166.23, 149.92, 143.80, 140.40, 135.22, 134.82, 133.81, 129.96, 129.48 (2C), 129.11, 127.00, 126.84, 126.78, 126.37, 124.18 (2C), 123.83, 123.73, 108.81, 13.62, 12.94.

ESI-HRMS m/z: $[M+H]^+$ Calcd for $C_{22}H_{19}N_3O$ 342.1601; found 342.1597

1-(1*H*-indol-3-yl)-3-(naphthalen-1-yl)urea (UR-01)

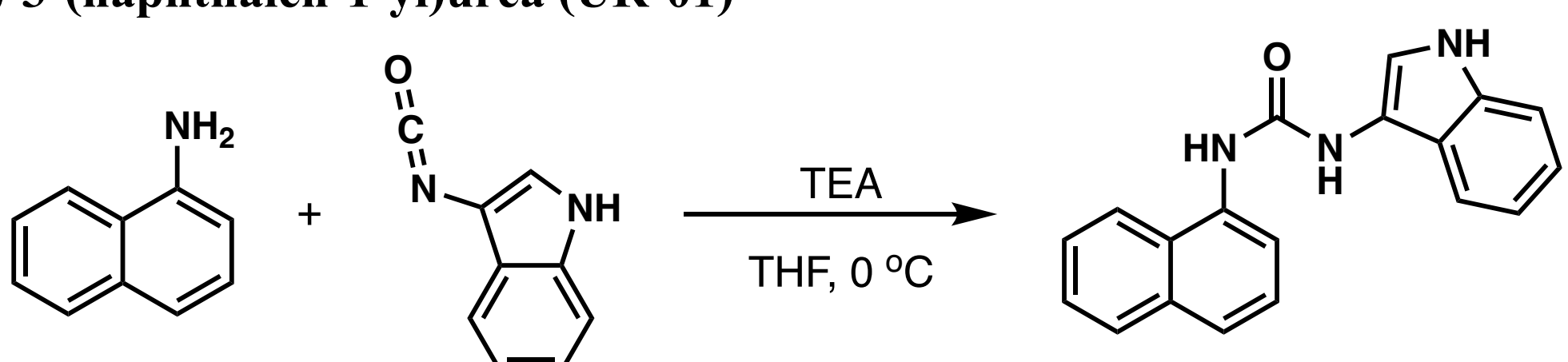

UR-01

General Procedure C: Reaction scale: 7.9 mg (0.050 mmol) of isocyanate. Crude solid was purified by DCM/EtOAc washes to afford UR-01 as a white solid (6.0 mg, 40%).

^{1}H NMR (400 MHz, Acetone-d_6) δ 9.98 (d, *J* = 25.5 Hz, 1H), 8.41 (d, *J* = 16.2 Hz, 1H), 8.21 (d, *J* = 6.7 Hz, 1H), 8.12 (s, 1H), 7.91 (d, *J* = 16.3 Hz, 1H), 7.73 (s, 1H), 7.68 – 7.53 (m, 2H), 7.47 (d, *J* = 15.4 Hz, 2H), 7.39 (t, *J* = 9.5 Hz, 1H), 7.12 (q, *J* = 8.3 Hz, 1H), 7.01 (dt, *J* = 13.6, 7.3 Hz, 1H).

^{13}C NMR (101 MHz, Acetone-*d6*) *δ* 154.2, 135.9, 135.3, 135.2, 129.4, 126.7, 126.5, 126.4, 125.1, 123.7, 122.6, 122.4, 121.9, 119.3, 118.3, 118.2, 117.8, 112.3, 112.2 ppm.

ESI-HRMS m/z: $[M+H]^+$ Calcd for $C_{19}H_{15}N_3O$ 302.1288; found 302.1283

Methyl 3-methyl-4-(3-(naphthalen-1-yl)ureido)benzoate (UR-02)

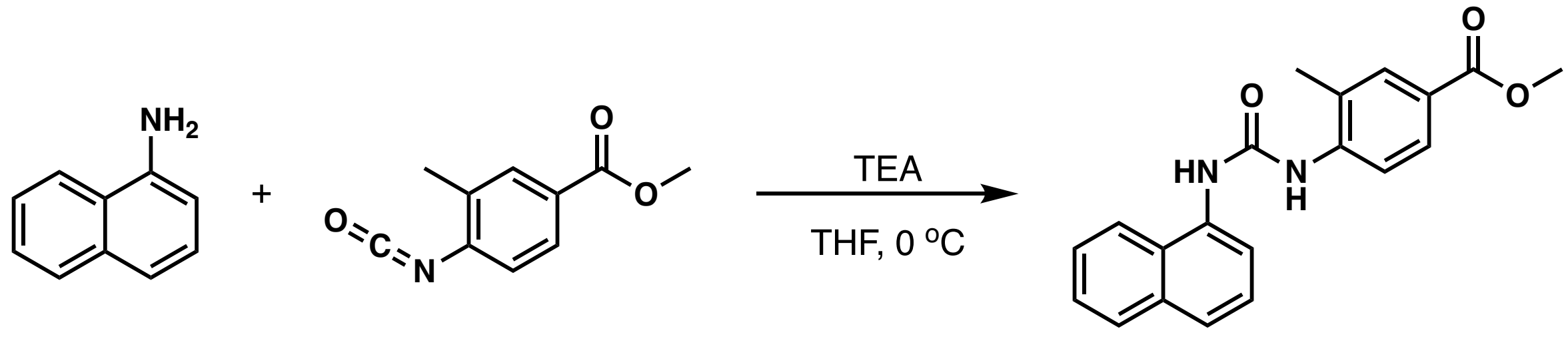

UR-02

General Procedure C: Reaction scale: 8.6 mg (0.045 mmol) of isocyanate. Crude solid was purified by acetone/MeOH washes to afford UR-02 as a white solid (10.4 mg, 70%).

^{1}H NMR (400 MHz, DMSO-d_6) δ 9.35 (s, 1H), 8.59 (s, 1H), 8.25 (d, J = 8.6 Hz, 1H), 8.18 (d, J = 8.5 Hz, 1H), 7.99 (d, J = 7.5 Hz, 1H), 7.95 (d, J = 8.0 Hz, 1H), 7.82 (s, 1H), 7.79 (d, J = 8.6 Hz, 1H), 7.68 (d, J = 8.2 Hz, 1H), 7.62 (dd, J = 7.4, 7.4 Hz, 1H), 7.56 (dd, J = 7.4, 7.4 Hz, 1H), 7.50 (dd, J = 7.9, 7.9 Hz, 1H), 3.82 (s, 3H), 2.39 (s, 3H).

^{13}C NMR (101 MHz, DMSO-d_6) δ 166.10, 152.72, 142.57, 133.96, 133.77, 131.33, 128.49, 127.92, 126.20, 126.05, 126.00, 125.90, 125.82, 123.39, 122.59, 121.61, 118.73, 118.18, 51.82, 18.06.

ESI-HRMS m/z: $[M+H]^+$ Calcd for $C_{20}H_{18}N_2O_3$ 335.1390; found 335.1387

1-cyclopentyl-3-(naphthalen-1-yl)urea (UR-03)

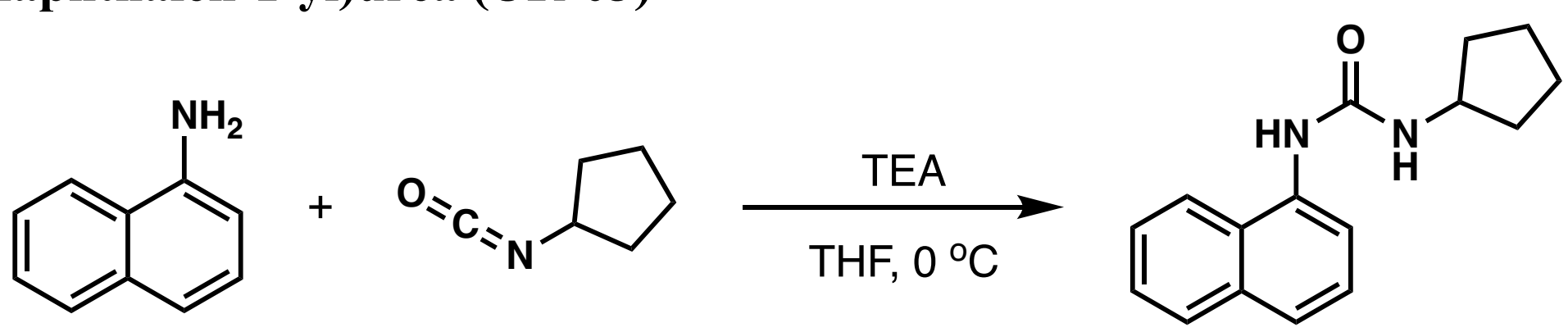

UR-03

General Procedure C: Reaction scale: 6.6 mg (0.059 mmol) of isocyanate. Crude solid was purified by MeOH washes to afford UR-03 as a white solid (4.9 mg, 33%).

^{1}H NMR (400 MHz, DMSO-d_6) δ 8.38 (s, 1H), 8.05 (dd, J = 8.4, 8.4 Hz, 2H), 7.88 (d, J = 8.0 Hz, 1H), 7.57 – 7.48 (m, 3H), 7.40 (dd, J = 7.9, 7.9 Hz, 1H), 6.64 (d, J = 7.1 Hz, 1H), 4.05 – 3.93 (m, 1H), 1.93 – 1.82 (m, 2H), 1.72 – 1.62 (m, 2H), 1.60 – 1.52 (m, 2H), 1.45 – 1.37 (m, 2H).

^{13}C NMR (101 MHz, DMSO-d_6) δ 155.04, 135.24, 133.71, 128.43, 125.98, 125.74, 125.38, 125.10, 121.68, 121.14, 115.71, 51.00, 32.95 (2C), 23.19 (2C).

ESI-HRMS m/z: $[M+H]^+$ Calcd for $C_{16}H_{18}N_2O$ 255.1492; found 255.1489

***Tert*-butyl (naphthalen-1-ylcarbamoyl)glycinate (UR-04)**

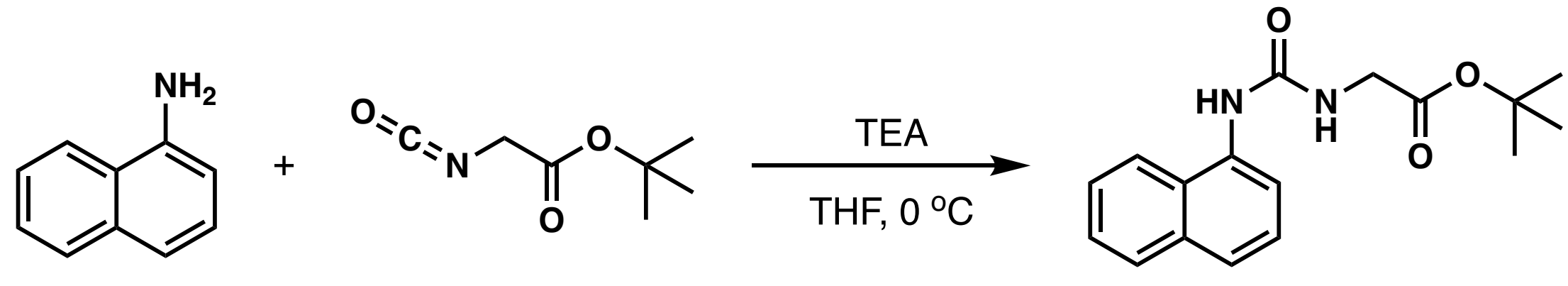

UR-04

General Procedure C: Reaction scale: 7.8 mg (0.050 mmol) of isocyanate. Purified by flash chromatography (SiO2, EtOAc/Hexane, gradient from 50 to 70% in EtOAc) to afford UR-04 as a white/brown solid (13.7 mg, 59%).

^{1}H NMR (400 MHz, Methanol-d_4) δ 8.05 (d, J = 7.5 Hz, 1H), 7.89 – 7.83 (m, 1H), 7.67 (d, J = 7.7 Hz, 2H), 7.53 – 7.47 (m, 2H), 7.44 (dd, J = 7.9, 7.9 Hz, 1H), 3.88 (s, 2H), 1.49 (s, 9H).

^{13}C NMR (101 MHz, Methanol-d_4) δ 171.66, 159.30, 135.79, 135.09, 129.78, 129.39, 127.02, 127.00, 126.68, 126.14, 122.98, 122.20, 82.70, 43.60, 28.33 (3C).

ESI-HRMS m/z: $[M+H]^+$ Calcd for $C_{17}H_{20}N_2O_3$ 301.1547; found 301.1545

1-(naphthalen-1-yl)-3-(*p*-tolyl)urea (UR-05)

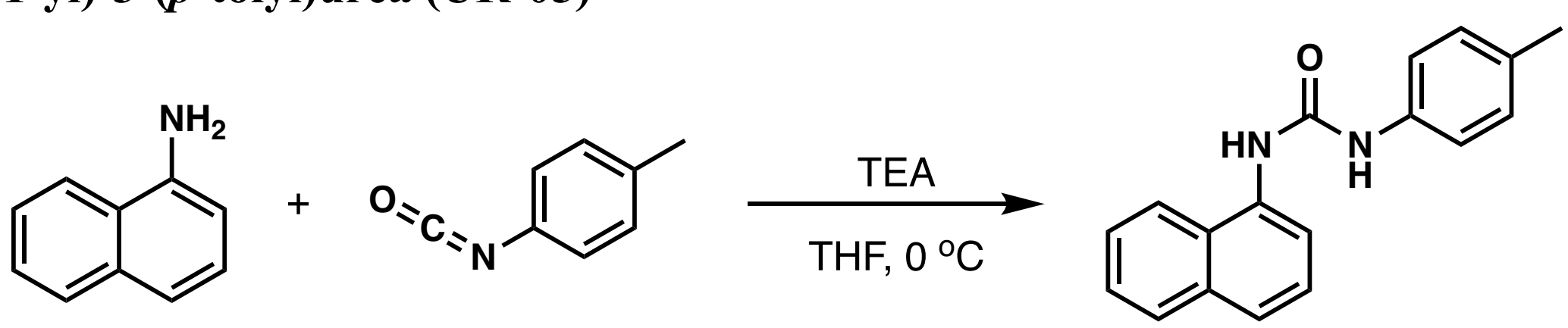

UR-05

General Procedure C: Reaction scale: 7.8 mg (0.054 mmol) of isocyanate. Crude solid was purified by MeOH washes to afford UR-05 as a white solid (11.1 mg, 74%).

^{1}H NMR (400 MHz, DMSO-d_6) δ 8.96 (s, 1H), 8.74 (s, 1H), 8.13 (d, J = 8.3 Hz, 1H), 8.03 (d, J = 7.5 Hz, 1H), 7.93 (d, J = 7.9 Hz, 1H), 7.62 (dd, J = 7.7, 7.7 Hz, 2H), 7.59 – 7.52 (m, 2H), 7.47 (dd, J = 7.9, 7.9 Hz, 1H), 7.40 (d, J = 8.2 Hz, 2H), 7.12 (d, J = 8.1 Hz, 2H), 2.26 (s, 3H).

^{13}C NMR (101 MHz, DMSO-d_6) δ 153.37, 137.65, 134.85, 134.16, 131.10, 129.72 (2C), 128.89, 126.34, 126.24, 126.13, 123.21, 121.73, 118.63 (2C), 117.58, 20.81.

ESI-HRMS m/z: $[M+H]^+$ Calcd for $C_{18}H_{16}N_2O$ 277.1335; found 277.1329

(Naphthalen-1-ylcarbamoyl)glycine (UR-06)

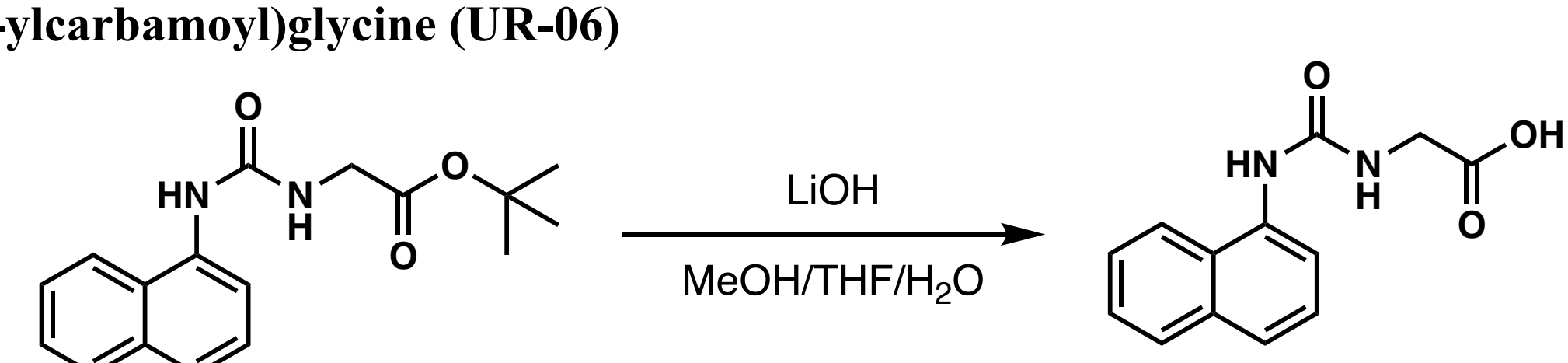

UR-06

General Procedure C: Reaction scale: 9.5 mg (0.032 mmol) of UR-04. No purification was needed and UR-06 was afforded as a white solid (8.1 mg, 100%).

^{1}H NMR (400 MHz, Methanol-*d*4) *δ* 8.06 (m, 1H), 7.86 (m, 1H), 7.68 (dd, *J* = 7.5, 7.5 Hz, 2H), 7.50 (m, 2H), 7.44 (t, *J* = 7.8 Hz, 1H), 3.98 (s, 2H) ppm.

^{13}C NMR (101 MHz, Methanol-d_4) *δ* 174.1, 159.3, 135.8, 135.1, 129.7, 129.4, 127.0, 127.0, 126.7, 126.0, 122.9, 122.0, 42.6 ppm.

ESI-HRMS m/z: $[M+H]^+$ Calcd for $C_{13}H_{12}N_2O_3$ 245.0921; found 245.0917

Tert-butyl 4-fluoro-3-(_N_-(naphthalen-1-yl)sulfamoyl)benzoate (SA-01)

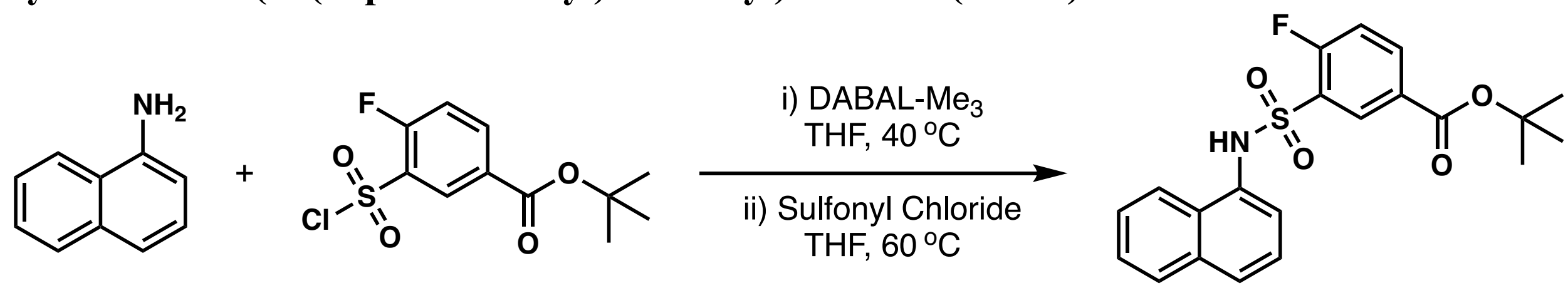

SA-01

General Procedure D: Reaction scale: 22.0 mg (0.075 mmol) of sulfonyl chloride. Purified by pTLC (EtOAc/hexane, 40:60) to afford SA-01 as a brown oil (23.1 mg, 77%).

^{1}H NMR (400 MHz, Acetone-*d*6) *δ* 8.29 (m, 2H), 8.16 (ddd, *J* = 8.2, 4.6, 2.1 Hz, 1H), 7.86 (m, 1H), 7.72 (d, *J* = 7.6 Hz, 1H), 7.47 (m, 2H), 7.39 (m, 3H), 1.52 (s, 9H) ppm.

^{13}C NMR (101 MHz, Acetone-d_6) δ 163.74, 162.22 (d, *J* = 260.9 Hz), 137.22 (d, *J* = 9.9 Hz), 135.31, 134.27, 132.49, 132.31, 130.93, 129.45 (d, *J* = 14.9 Hz), 129.43 (d, *J* = 3.5 Hz), 129.07, 128.50, 127.25 (d, *J* = 5.8 Hz), 126.32, 124.89, 123.46, 118.39 (d, *J* = 22.3 Hz), 82.65, 28.07 (3C).

^{19}F NMR (376 MHz, DMSO) δ -102.85.

ESI-HRMS m/z: $[M+H]^+$ Calcd for $C_{21}H_{20}NO_4FS$ 402.1170; found 402.1169

6-hydroxy-_N_-(naphthalen-1-yl)pyridine-3-sulfonamide (SA-02)

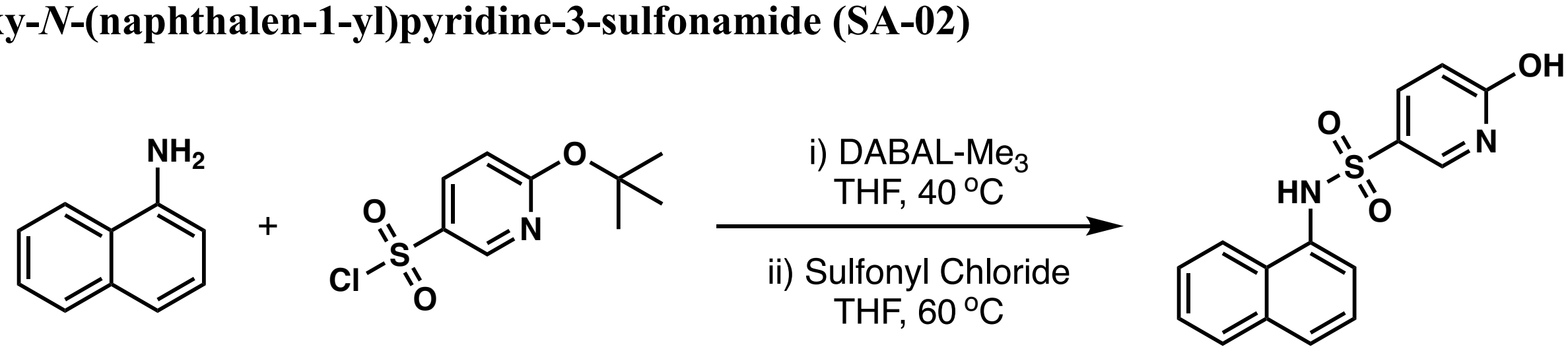

SA-02

General Procedure D: Reaction scale: 21.0 mg (0.084 mmol) of sulfonyl chloride. Crude solid was purified by DCM washes to afford SA-02 as a white solid (19.4 mg, 77%).

^{1}H NMR (400 MHz, Methanol-d_4) δ 8.01 (d, *J* = 7.1 Hz, 1H), 7.86 (d, *J* = 6.1 Hz, 1H), 7.80 (d, *J* = 7.9 Hz, 1H), 7.71 (d, *J* = 9.2 Hz, 1H), 7.59 (s, 1H), 7.53 – 7.45 (m, 2H), 7.42 (d, *J* = 8.0 Hz, 1H), 7.37 (d, *J* = 6.9 Hz, 1H), 6.46 (d, *J* = 9.6 Hz, 1H).

^{13}C NMR (101 MHz, Methanol-d_4) δ 164.74, 139.71, 139.07, 135.91, 133.25, 131.30, 129.32, 128.74, 127.45, 127.38, 126.47, 125.79, 123.87, 121.26, 121.02.

ESI-HRMS m/z: $[M+H]^+$ Calcd for $C_{15}H_{12}N_2O_3S$ 301.0641; found 301.0639

2,3,4,5,6-pentamethyl-*N*-(naphthalen-1-yl)benzenesulfonamide (SA-03)

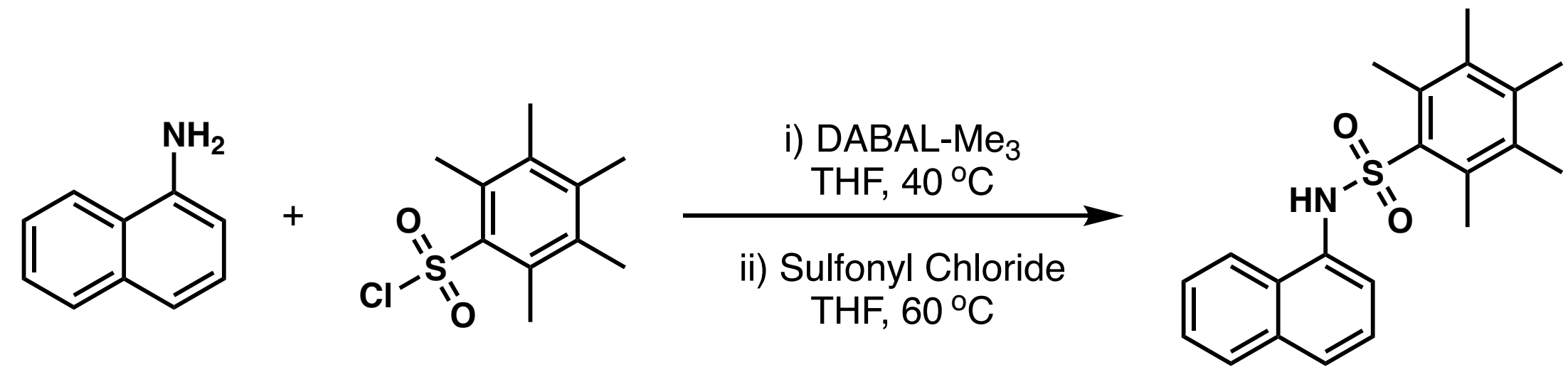

SA-03

General Procedure D: Reaction scale: 10.4 mg (0.042 mmol) of sulfonyl chloride. Purified by flash chromatography (SiO2, EtOAc/hexane, gradient from 10 to 50% in EtOAc) and Et2O wash to afford SA-03 as a white/pink solid (12.8 mg, 86%).

^{1}H NMR (400 MHz, Acetone-d_6) δ 8.87 (s, 1H), 8.34 – 8.27 (m, 1H), 7.92 – 7.87 (m, 1H), 7.77 (d, *J* = 8.2 Hz, 1H), 7.53 – 7.48 (m, 2H), 7.34 (dd, *J* = 7.8, 7.8 Hz, 1H), 7.18 (d, *J* = 7.4 Hz, 1H), 2.44 (s, 6H), 2.25 (s, 3H), 2.19 (s, 6H).

^{13}C NMR (101 MHz, Acetone-*d*6) *δ* 140.2, 135.5 (2C), 135.4, 135.3, 133.7, 131.1, 128.9 (2C), 127.6, 127.1, 126.9, 126.2, 124.1 (2C), 123.9, 19.5 (2C), 17.8, 17.1 (2C) ppm.

ESI-HRMS m/z: $[M+K]^+$ Calcd for $C_{21}H_{23}NO_2S$ 392.1081; found 392.1083

Methyl 3-((*N*-(naphthalen-1-yl)sulfamoyl)methyl)benzoate (SA-04)

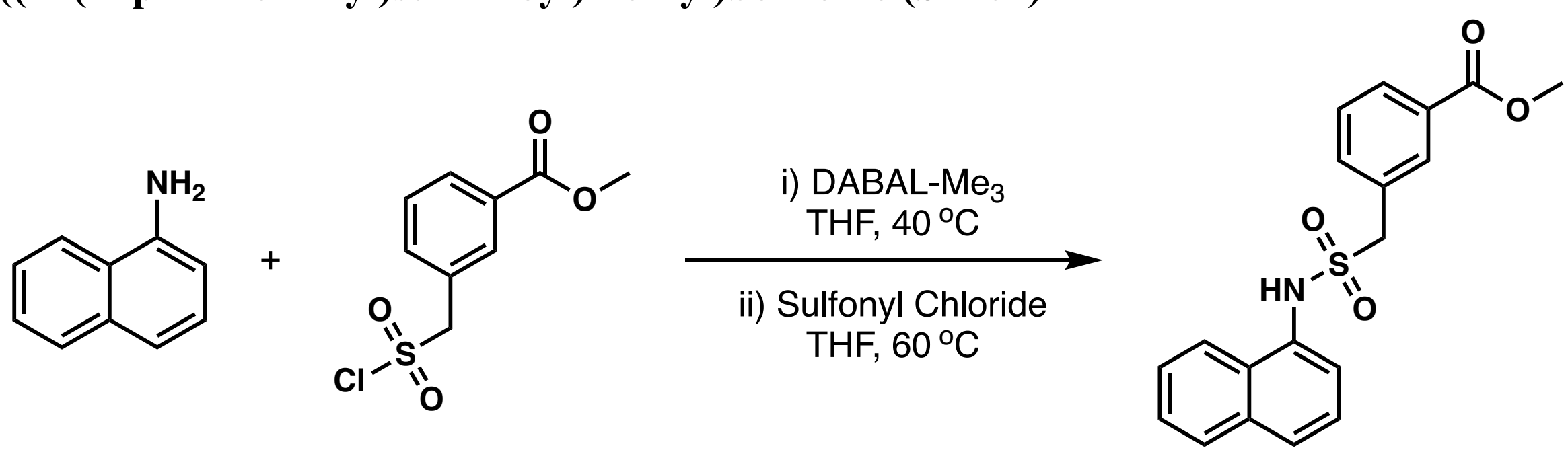

SA-04

General Procedure D: Reaction scale: 20.9 mg (0.084 mmol) of sulfonyl chloride. Purified by flash chromatography (SiO2, EtOAc/hexane, gradient from 20 to 70% in EtOAc) to afford SA-04 as a pink oil (19.4 mg, 65%).

^{1}H NMR (400 MHz, Acetone-d_6) δ 8.76 (s, 1H), 8.31 – 8.20 (m, 1H), 8.04 – 7.88 (m, 3H), 7.83 (d, *J* = 8.2 Hz, 1H), 7.70 (d, *J* = 7.3 Hz, 1H), 7.63 (d, *J* = 7.7 Hz, 1H), 7.59 – 7.48 (m, 3H), 7.45 (dd, *J* = 8.0, 8.0 Hz, 1H), 4.63 (s, 2H), 3.83 (s, 3H).

^{13}C NMR (101 MHz, Acetone-d_6) δ 166.73, 136.41, 135.40, 133.84, 132.76, 131.38, 131.26, 129.99, 129.79, 129.46, 129.10, 127.30, 127.13 (2C), 126.49, 123.63, 122.54, 58.35, 52.37.

ESI-HRMS m/z: $[M+H]^+$ Calcd for $C_{19}H_{17}NO_4S$ 356.0951; found 356.0943

6-amino-5-methyl-*N*-(naphthalen-1-yl)pyridine-3-sulfonamide) (SA-05)

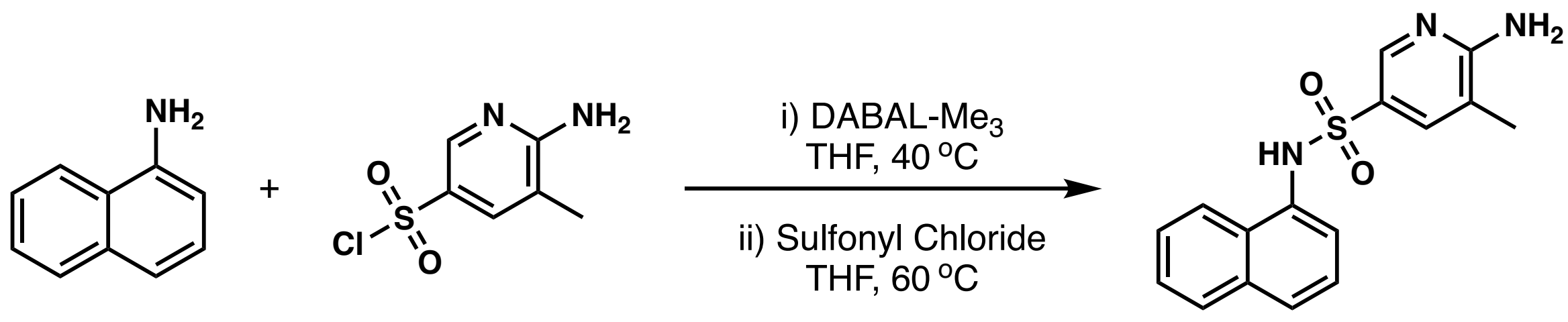

SA-05

General Procedure D: Reaction scale: 9.9 mg (0.048 mmol) of sulfonyl chloride. Purified by flash chromatography (SiO2, EtOAc/hexane, gradient from 50 to 100% in EtOAc) to afford SA-05 as an orange oil (13.7 mg, 91%).

^{1}H NMR (400 MHz, Acetone-d_6) δ 8.85 (s, 1H), 8.26 – 8.18 (m, 1H), 8.10 (s, 1H), 7.93 – 7.84 (m, 1H), 7.78 (d, J = 7.9 Hz, 1H), 7.50 (s, 1H), 7.50 – 7.33 (m, 4H), 6.05 (s, 2H), 2.07 (s, 3H).

^{13}C NMR (101 MHz, Acetone-d_6) δ 161.67, 146.91, 136.06, 135.34, 133.84, 130.43, 128.96, 127.48, 127.00, 126.85, 126.27, 125.31, 123.85, 123.70, 116.49, 17.05.

ESI-HRMS m/z: $[M+H]^+$ Calcd for $C_{16}H_{15}N_3O_2S$ 314.0958; found 314.0954

2-hydroxy-5-methyl-3-(*N*-(naphthalen-1-yl)sulfamoyl)benzoic acid (SA-06)

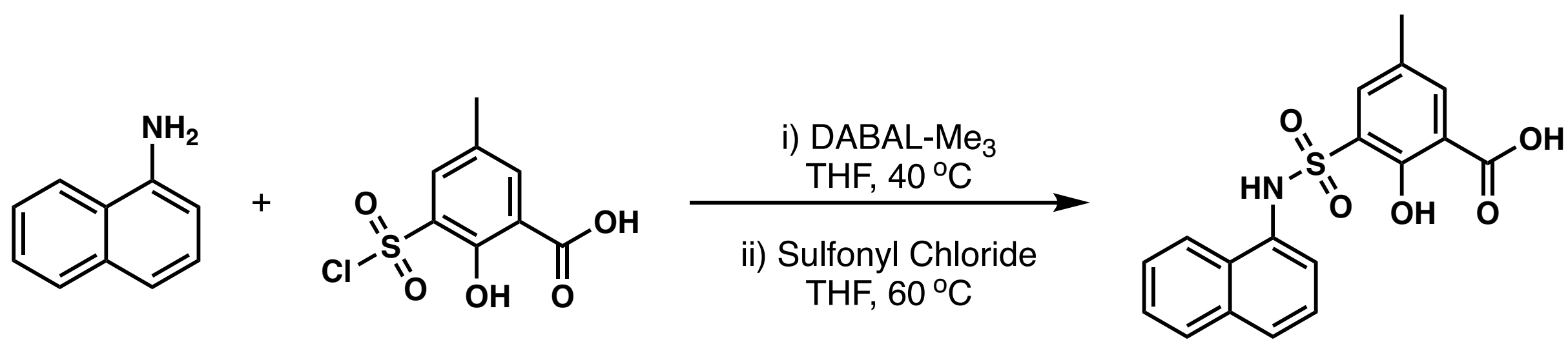

SA-06

General Procedure D: 10.5 mg (0.042 mmol) of sulfonyl chloride. Crude solid was purified by Et2O washes to afford SA-06 as a brown/pink solid (6.6 mg, 44%).

^{1}H NMR (400 MHz, Acetone-d_6) δ 8.52 – 8.39 (m, 1H), 7.90 (s, 1H), 7.88 – 7.84 (m, 1H), 7.74 (d, J = 7.6 Hz, 1H), 7.63 (s, 1H), 7.53 – 7.47 (m, 2H), 7.40 – 7.33 (m, 2H), 2.23 (s, 3H).

^{13}C NMR (101 MHz, Acetone-d_6) δ 172.21, 158.15, 136.81, 136.14, 135.28, 133.54, 130.78, 128.83, 128.76, 127.92, 127.65, 127.06, 126.87, 126.27, 124.15, 123.30, 115.03, 20.04.

ESI-HRMS m/z: $[M+H]^+$ Calcd for $C_{18}H_{15}NO_5S$ 358.0744; found 358.0742

1-(1,4-dioxan-2-yl)-*N*-(naphthalen-1-yl)methanesulfonamide (SA-07)

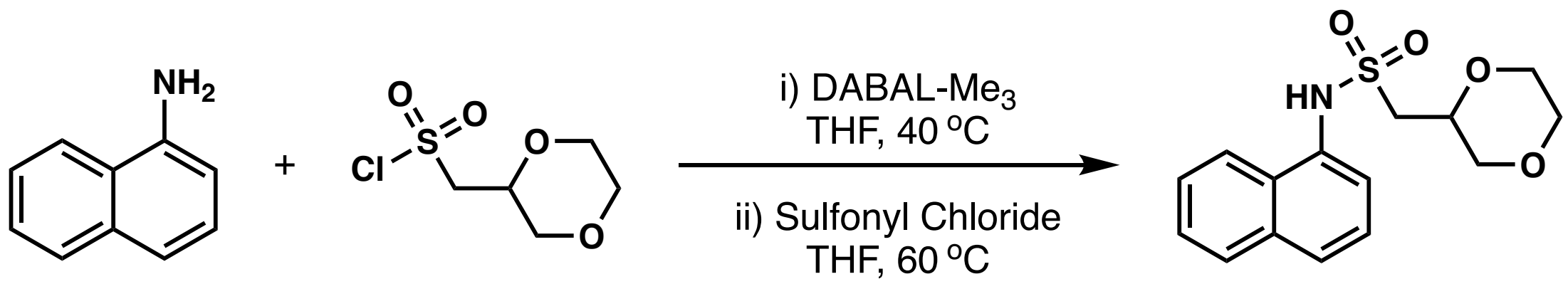

SA-07

General Procedure D: Reaction scale: 10.5 mg (0.042 mmol) of sulfonyl chloride. Purified by flash chromatography (SiO2, EtOAc/hexane, gradient from 20 to 100% in EtOAc) to afford SA-07 as an orange oil (13.8 mg, 92%).

^{1}H NMR (400 MHz, Acetone-d_6) δ 8.61 (s, 1H), 8.42 (d, J = 8.2 Hz, 1H), 7.95 (d, J = 7.4 Hz, 1H), 7.85 (d, J = 8.2 Hz, 1H), 7.72 (d, J = 7.2 Hz, 1H), 7.63 – 7.46 (m, 3H), 4.18 – 4.08 (m, 1H), 3.83 (dd, J = 11.5, 2.3 Hz, 1H), 3.75 (dd, J = 11.3, 2.2 Hz, 1H), 3.69 – 3.62 (m, 2H), 3.50 (td, J = 11.8, 2.8 Hz, 1H), 3.36 – 3.29 (m, 2H), 3.22 (dd, J = 14.7, 4.9 Hz, 1H).

^{13}C NMR (101 MHz, Acetone-d_6) δ 135.44, 133.98, 130.53, 128.98, 127.59, 127.14, 127.04, 126.44, 124.16, 123.30, 71.96, 70.39, 67.31, 66.78, 53.42.

ESI-HRMS m/z: $[M+H]^+$ Calcd for $C_{15}H_{17}NO_4S$ 308.0951; found 308.0950

4-methyl-*N*-(naphthalen-1-yl)benzenesulfonamide (SA-08)

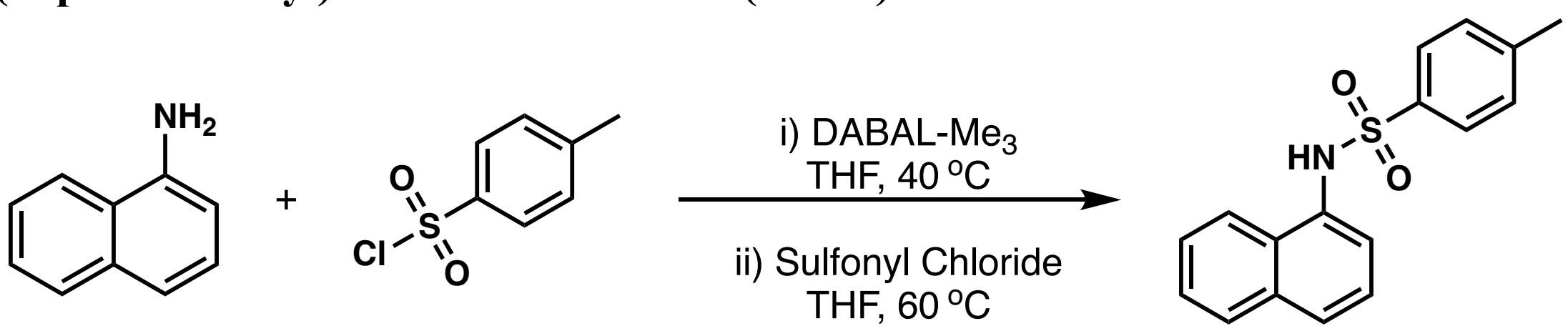

SA-08

General Procedure D: Reaction scale: 12.8 mg (0.067 mmol) of sulfonyl chloride. Purified by flash chromatography (SiO2, EtOAc/hexane, gradient from 40 to 50% in EtOAc) to afford SA-08 as a brown solid (16.5 mg, 82%).

^{1}H NMR (400 MHz, Acetone-d_6) δ 8.94 (s, 1H), 8.15 (d, J = 8.2 Hz, 1H), 7.88 (d, J = 7.7 Hz, 1H), 7.78 (d, J = 8.1 Hz, 1H), 7.62 (d, J = 8.2 Hz, 2H), 7.50 – 7.38 (m, 3H), 7.32 (d, J = 6.9 Hz, 1H), 7.27 (d, J = 8.0 Hz, 2H), 2.34 (s, 3H).

^{13}C NMR (101 MHz, Acetone-d_6) δ 144.24, 138.47, 135.32, 133.59, 130.51, 130.27 (2C), 128.94, 128.07 (2C), 127.65, 127.02, 126.89, 126.23, 123.90, 123.77, 21.34.

ESI-HRMS m/z: $[M+H]^+$ Calcd for $C_{17}H_{15}NO_2S$ 298.0896; found 298.0892

N-((5-methylpyrazin-2-yl)methyl)naphthalen-1-amine (ALK-01)

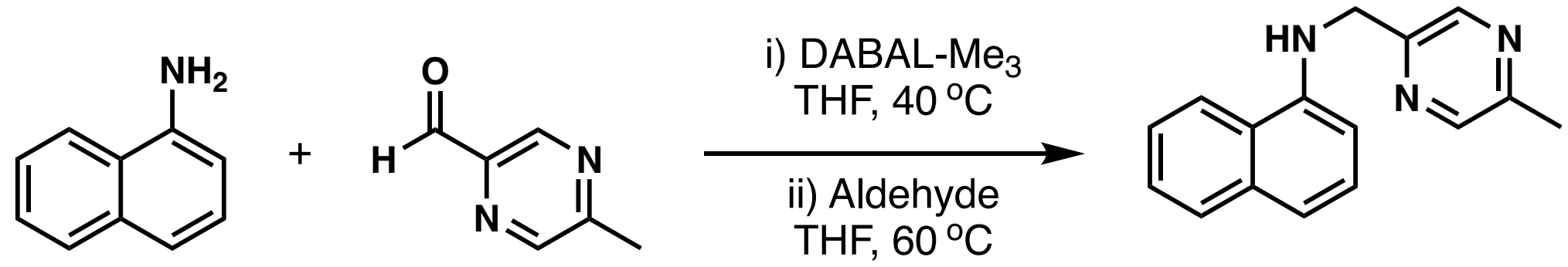

ALK-01

General Procedure E: Reaction scale: 21.5 mg (0.150 mmol) of 1-naphthylamine. Purified by flash chromatography (SiO2, EtOAc/hexane, gradient from 50 to 80% in EtOAc) to afford ALK-01 as a yellow oil (8.9 mg, 24%).

^{1}H NMR (300 MHz, Acetone-d_6) δ 8.59 (s, 1H), 8.48 (s, 1H), 8.21 – 8.13 (m, 1H), 7.85 – 7.75 (m, 1H), 7.51 – 7.40 (m, 2H), 7.29 – 7.15 (m, 2H), 6.56 (d, J = 7.2 Hz, 1H), 6.41 (s, 1H), 4.68 (d, J = 5.3 Hz, 2H), 2.49 (s, 3H).

^{13}C NMR (75 MHz, Acetone-d_6) δ 152.95, 152.40, 144.25, 144.18, 143.41, 135.46, 129.20, 127.43, 126.54, 125.32, 124.53, 121.61, 117.74, 105.10, 47.69, 21.14.

ESI-HRMS m/z: $[M+H]^+$ Calcd for $C_{16}H_{15}N_3$ 250.1339; found 250.1336

Methyl 2-(4-((naphthalen-1-ylamino)methyl)phenyl)acetate (ALK-02)

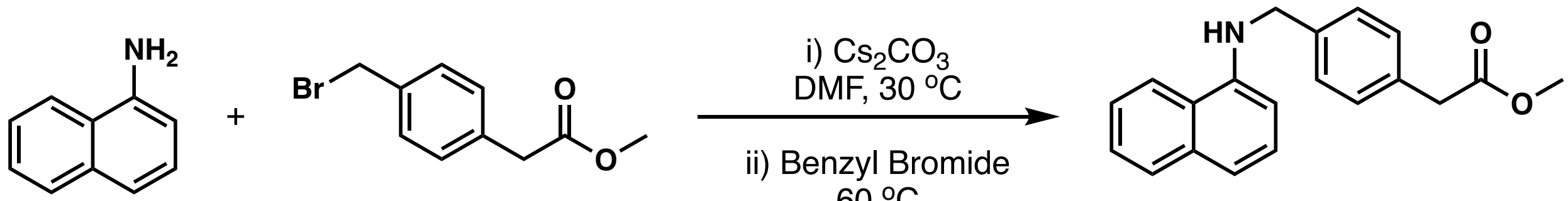

ALK-02

General Procedure F: Reaction scale: 11.9 mg (0.049 mmol) of alkyl halide. Purified by flash chromatography (SiO2, EtOAc/hexane, gradient from 20 to 50% in EtOAc) to afford ALK-02 as a yellow oil (6.2 mg, 42%).

^{1}H NMR (400 MHz, Methanol-d_4) δ 8.14 – 8.02 (m, 1H), 7.77 – 7.69 (m, 1H), 7.44 – 7.39 (m, 2H), 7.38 (d, J = 8.0 Hz, 2H), 7.22 (d, J = 8.0 Hz, 2H), 7.17 (dd, J = 7.8, 7.8 Hz, 1H), 7.11 (d, J = 8.1 Hz, 1H), 6.44 (d, J = 7.4 Hz, 1H), 4.52 (s, 2H), 3.66 (s, 3H), 3.62 (s, 2H).

^{13}C NMR (101 MHz, Methanol-d_4) δ 159.65, 141.80, 134.35, 134.05 (2C), 128.32 (2C), 127.49, 126.50, 125.31, 125.04, 121.91, 117.64, 105.61, 52.03, 48.30, 41.37.

ESI-HRMS m/z: $[M+H]^+$ Calcd for $C_{20}H_{19}NO_2$ 306.1489; found 306.1481

N-(3,5-difluorobenzyl)naphthalen-1-amine (ALK-03)

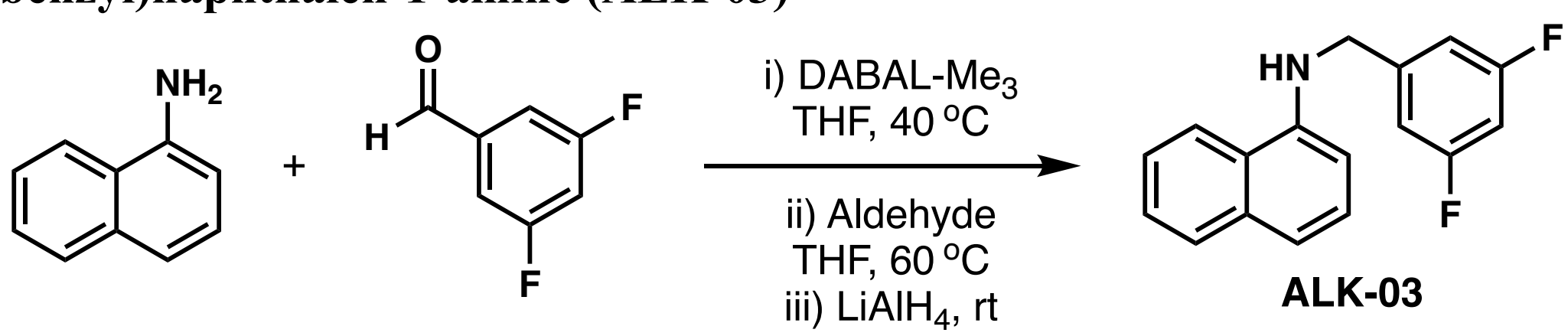

ALK-03

General Procedure E: Reaction scale: 15.9 mg (0.111 mmol) of 1-naphthylamine. Purified by flash chromatography (SiO2, EtOAc/hexane, gradient from 10 to 40% in EtOAc) to afford ALK-03 as a yellow oil (22.9 mg, 77%).

^{1}H NMR (400 MHz, Acetone-d_6) δ 8.20 (m, 1H), 7.79 (m, 1H), 7.46 (m, 2H), 7.21 (m, 2H), 7.12 (d, J = 7.3 Hz, 2H), 6.87 (t, J = 9.4 Hz, 1H), 6.43 (m, 2H), 4.65 (d, J = 5.8 Hz, 2H) ppm.

^{13}C NMR (101 MHz, Acetone-d_6) δ 164.08 (dd, J = 246.5, 12.9 Hz, 2C), 146.54 (dd, J = 8.3, 8.3 Hz), 144.08, 135.47, 129.19, 127.38, 126.52, 125.28, 124.48, 121.72, 117.72, 110.55 (dd, J = 18.6, 6.7 Hz, 2C), 105.15, 102.62 (dd, J = 25.9, 25.9 Hz), 47.40 (t, J = 2.1, 2.1 Hz).

^{19}F NMR (376 MHz, DMSO-d_6) δ -110.23.

ESI-HRMS m/z: $[M+H]^+$ Calcd for $C_{17}H_{13}NF_2$ 270.1089; found 270.1084

Tert-butyl 3-((naphthalen-1-ylamino)methyl)benzoate (ALK-04)

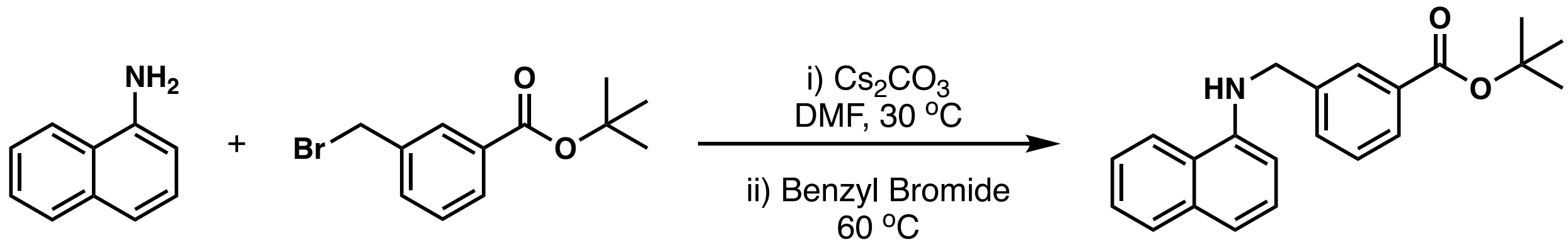

ALK-04

General Procedure F: Reaction scale: 12.2 mg (0.045 mmol) of alkyl halide. Purified by flash chromatography (SiO2, EtOAc/hexane, gradient from 20 to 40% in EtOAc) to afford ALK-04 as a white solid (12.2 mg, 81%).

^{1}H NMR (400 MHz, Acetone-d_6) δ 8.21 (m, 1H), 8.09 (s, 1H), 7.84 (d, J = 7.7 Hz, 1H), 7.78 (m, 1H), 7.68 (d, J = 7.4 Hz, 1H), 7.43 (m, 3H), 7.21 (dd, J = 7.8 Hz, 1H), 7.16 (d, J = 8.0 Hz, 1H), 6.50 (d, J = 7.3 Hz, 1H), 6.36 (s, 1H), 4.65 (d, J = 5.5 Hz, 2H), 1.55 (s, 9H) ppm.

^{13}C NMR (101 MHz, Acetone-d_6) δ 166.03, 144.47, 141.53, 135.48, 133.11, 132.22, 129.27, 129.14, 128.88, 128.55, 127.42, 126.43, 125.15, 124.53, 121.78, 117.40, 105.11, 81.24, 47.88, 28.25 (3C).

ESI-HRMS m/z: $[M+H]^+$ Calcd for $C_{22}H_{23}NO_2$ 334.1802; found 334.1800

1-(2,5-dimethoxyphenyl)-2-(naphthalen-1-ylamino)ethan-1-ol (ALK-05)

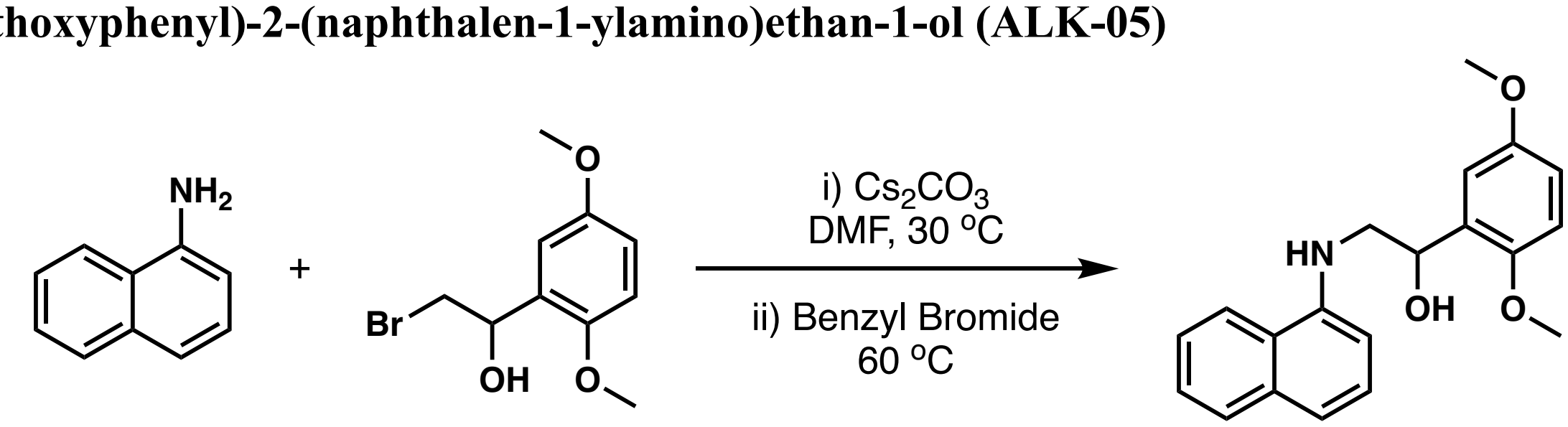

ALK-05

General Procedure F: Reaction scale: 12.0 mg (0.046 mmol) of alkyl halide. Purified by flash chromatography (SiO2, EtOAc/hexane, gradient from 20 to 60% in EtOAc) to afford ALK-05 as a yellow oil (3.8 mg, 26%).

^{1}H NMR (400 MHz, Acetone-d_6) δ 8.26 – 8.20 (m, 1H), 7.80 – 7.74 (m, 1H), 7.49 – 7.42 (m, 2H), 7.13 (s, 1H), 7.12 (s, 1H), 7.02 (d, J = 3.1 Hz, 1H), 6.97 (d, J = 8.9 Hz, 1H), 6.76 (dd, J = 8.9, 3.2 Hz, 1H), 6.36 – 6.28 (m, 1H), 5.88 (dd, 1H), 5.08 – 5.02 (m, 1H), 4.27 (s, 1H), 3.92 (s, 3H), 3.85 – 3.72 (m, 2H), 3.58 (s, 3H).

^{13}C NMR (101 MHz, Acetone-d_6) δ 154.84, 152.21, 144.00, 135.35, 130.70, 129.17, 127.35, 126.37, 125.21, 124.74, 121.57, 117.53, 114.63, 112.44, 112.32, 106.11, 65.78, 56.39, 55.54, 55.38.

ESI-HRMS m/z: $[M+H]^+$ Calcd for $C_{20}H_{21}NO_3$ 324.1594; found 324.1586

_N_-(2-cyclohexyl-1-phenylethyl)naphthalen-1-amine (ALK-06)

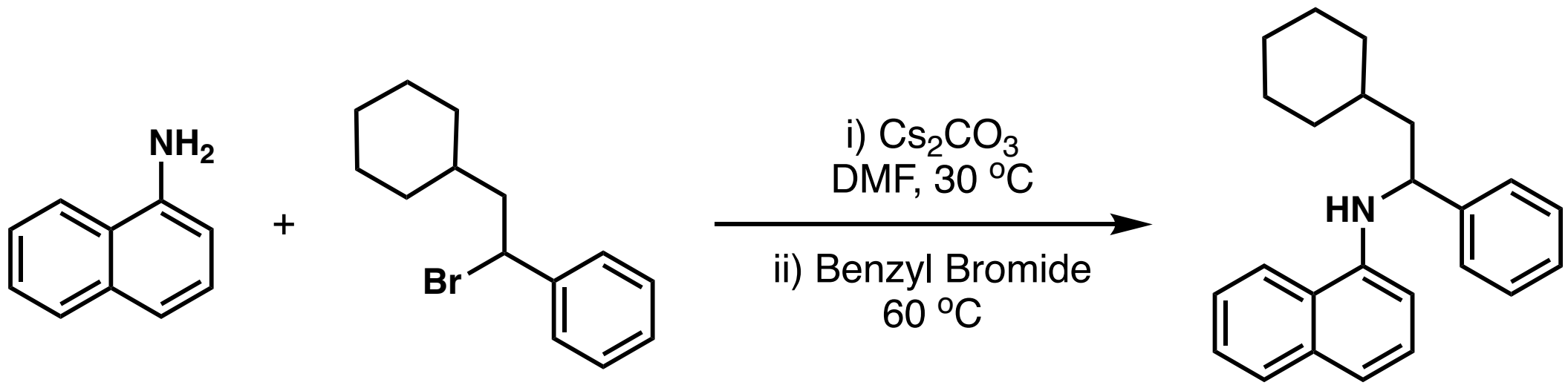

ALK-06

General Procedure F: Reaction scale: 12.3 mg (0.046 mmol) of alkyl halide. Purified by flash chromatography (SiO2, EtOAc/hexane, 10:90) to afford ALK-06 as a brown oil (12.2 mg, 81%).

^{1}H NMR (400 MHz, Methanol-d_4) δ 8.18 (d, J = 7.6 Hz, 1H), 7.70 (dd, J = 6.8, 1.7 Hz, 1H), 7.40 (m, 4H), 7.27 (dd, J = 7.5, 7.5 Hz, 2H), 7.16 (dd, J = 7.2, 7.2 Hz, 2H), 7.06 (m, 2H), 6.34 (d, J = 7.2 Hz, 1H), 4.60 (dd, J = 8.2, 6.1 Hz, 1H), 1.96 (m, 3H, 1.67 (m, 10H) ppm.

^{13}C NMR (101 MHz, Methanol-d_4) δ 143.55, 140.81, 132.67, 129.43 (2C), 129.28, 128.80, 127.65, 127.47 (2C), 127.40, 126.41, 125.24, 121.86, 117.33, 106.39, 56.65, 47.91, 35.99, 34.97, 34.10, 27.73, 27.47, 27.35.

Note: ^{13}C shows mixture of conformers

ESI-HRMS m/z: $[M+H]^+$ Calcd for $C_{24}H_{27}N$ 330.2216; found 330.2213

_N_-benzyl-_N_-ethyl-2-(naphthalen-1-ylamino)acetamide (ALK-07)

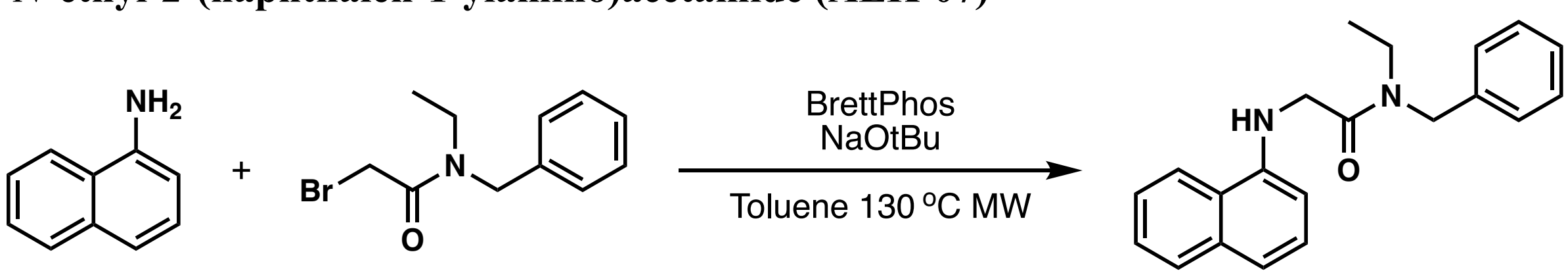

ALK-07

General Procedure H: Reaction scale: 80 mg (0.35 mmol) of alkyl halide. Purified by flash chromatography (SiO2, EtOAc/hexane, gradient from 0 to 60% in EtOAc) to afford ALK-07 as a brown oil (30 mg, 27%)

^{1}H NMR (400 MHz, Acetone-d_6) δ 8.00 (t, J = 7.7 Hz, 1H), 7.80 (t, J = 6.8 Hz, 1H), 7.53 – 7.44 (m, 2H), 7.43 – 7.15 (m, 7H), 6.57 (dd, J = 46.1, 7.4 Hz, 1H), 6.01 (s, 1H), 4.77 (d, J = 18.8 Hz, 2H), 4.20 (dd, J = 34.0, 3.7 Hz, 2H), 3.51 (p, J = 6.8 Hz, 2H), 1.25 (d, J = 7.1 Hz, 3H).

^{13}C NMR (101 MHz, acetone) δ 169.74, 169.66, 143.89, 143.81, 139.09, 138.38, 135.36, 135.33, 129.67, 129.30, 129.25, 128.74, 128.29, 127.97, 127.69, 127.61, 127.57, 126.55, 125.45, 124.17, 124.13, 121.10, 121.07, 117.42, 105.00, 104.95, 50.02, 48.52, 46.05, 45.70, 41.80, 41.27, 13.90, 12.90.

ESI-HRMS m/z: $[M+H]^+$ Calcd for $C_{21}H_{22}N_2O$ 319.1805; found 319.1798

Methyl 4-((naphthalen-1-ylamino)methyl)benzoate (ALK-08)

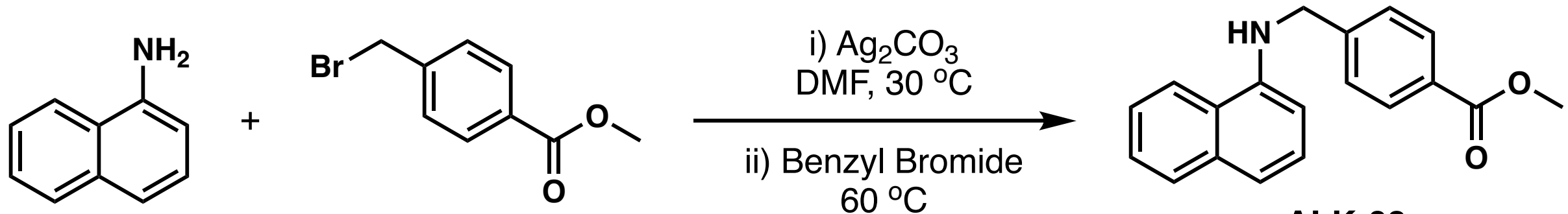

ALK-08

General Procedure E: Reaction scale: 23.6 mg (0.103 mmol) of alkyl halide. Purified by flash chromatography (SiO2, EtOAc/hexane, 20:80) to afford ALK-08 as a pink/orange oil (17.9 mg, 60%).

^{1}H NMR (300 MHz, Acetone-d_6) δ 8.25 – 8.18 (m, 1H), 7.97 (d, J = 8.1 Hz, 2H), 7.84 – 7.73 (m, 1H), 7.58 (d, J = 8.0 Hz, 2H), 7.51 – 7.38 (m, 2H), 7.26 – 7.09 (m, 2H), 6.54 – 6.29 (m, 2H), 4.68 (d, J = 5.6 Hz, 2H), 3.86 (s, 3H).

^{13}C NMR (75 MHz, Acetone-d_6) δ 167.11, 146.82, 144.36, 135.48, 130.34 (2C), 129.70, 129.17, 127.95 (2C), 127.39, 126.47, 125.19, 124.49, 121.76, 117.45, 105.12, 52.23, 47.90.

ESI-HRMS m/z: $[M+H]^+$ Calcd for $C_{19}H_{17}NO_2$ 292.1332; found 292.1331

***N*-benzylnaphthalen-1-amine (ALK-09)**

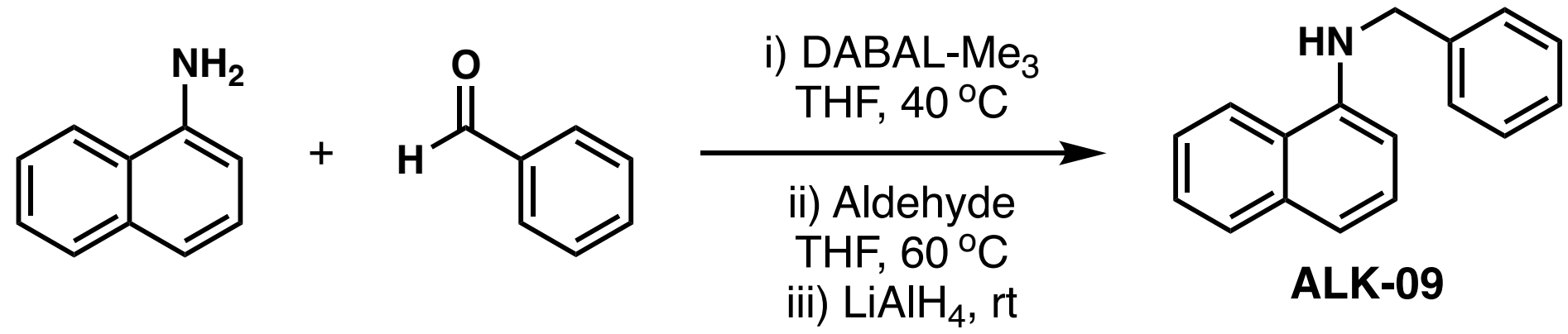

ALK-09

General Procedure E: Reaction scale: 250 mg (1.75 mmol) of 1-naphthylamine. Purified flash chromatography (SiO2, EtOAc/hexane, gradient from 0 to 60% in EtOAc) to afford ALK-09 as a white solid (192 mg, 47.1%).

^{1}H NMR (400 MHz, Acetone-d_6) δ 8.20 (d, J = 7.6 Hz, 1H), 7.83 – 7.72 (m, 1H), 7.53 – 7.38 (m, 4H), 7.38 – 7.27 (m, 2H), 7.27 – 7.18 (m, 2H), 7.15 (d, J = 8.1 Hz, 1H), 6.50 (d, J = 7.4 Hz, 1H), 6.23 (s, 1H), 4.58 (d, J = 5.3 Hz, 2H).

^{13}C NMR (101 MHz, Acetone-d_6) δ 143.78, 140.07, 134.58, 128.34 (2C), 128.24, 127.10 (2C), 126.70, 126.57, 125.50, 124.19, 123.61, 120.90, 116.31, 104.18, 47.30.

ESI-HRMS m/z: $[M+H]^+$ Calcd for $C_{17}H_{15}N$ 234.1277; found 234.1274

***N*-(2-(4-methoxyphenoxy)ethyl)naphthalen-1-amine (ALK-10)**

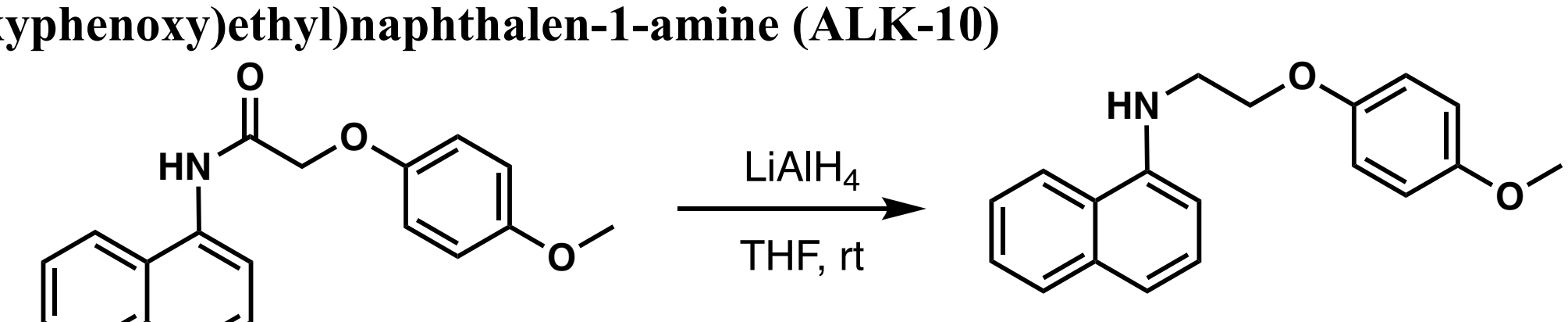

ALK-10

General Procedure G: Reaction scale: 21.5 mg (0.070 mmol) of AM-09. Purified by flash chromatography (SiO2, EtOAc/hexane, gradient from 10 to 20% in EtOAc) to afford ALK-10 as a yellow oil (3.2 mg, 16%).

^{1}H NMR (500 MHz, Acetone-d_6) δ 8.10 (d, J = 8.2 Hz, 1H), 7.78 (d, J = 7.9 Hz, 1H), 7.47 – 7.37 (m, 2H), 7.33 (dd, J = 7.9, 7.9 Hz, 1H), 7.19 (d, J = 8.1 Hz, 1H), 6.93 (d, J = 9.1 Hz, 2H), 6.86 (d, J = 9.1 Hz, 2H), 6.70 (d, J = 7.6 Hz, 1H), 5.68 (t, J = 5.9 Hz, 1H), 4.29 (t, J = 5.6 Hz, 2H), 3.73 (s, 3H), 3.70 (dt, J = 5.6, 5.6 Hz, 2H).

^{13}C NMR (126 MHz, Acetone-d_6) δ 154.10, 153.04, 143.90, 134.62, 128.25, 126.69, 125.57, 124.25, 123.67, 120.79, 116.57, 115.46 (2C), 114.52 (2C), 103.63, 66.70, 54.93, 43.25.

ESI-HRMS m/z: $[M+H]^+$ Calcd for $C_{19}H_{19}NO_2$ 294.1489; found 294.1485

_N_-((4-methyl-1_H_-imidazol-5-yl)methyl)naphthalen-1-amine (ALK-11)

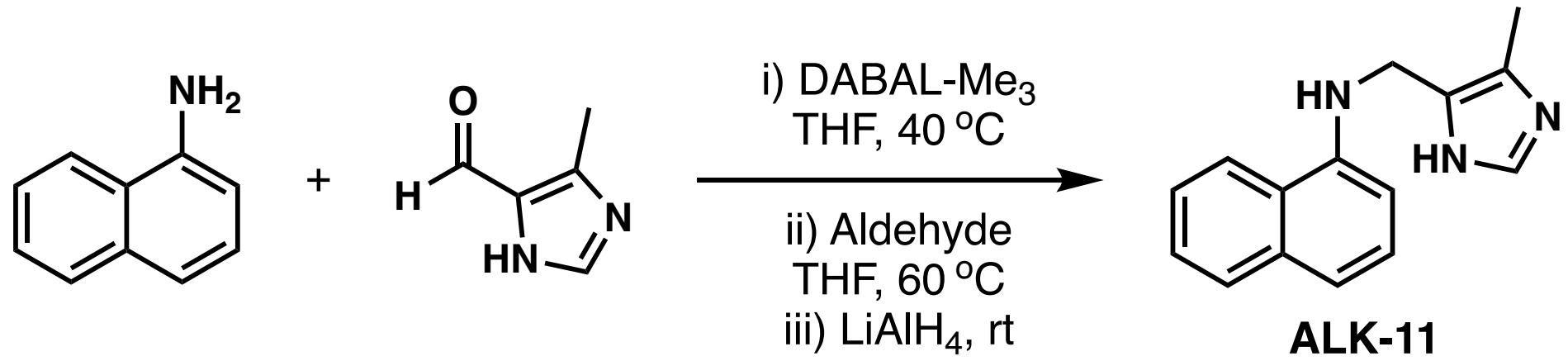

General Procedure E: Reaction scale: 21.5 mg (0.150 mmol) of 1-naphthylamine. Crude solid was purified by acetone washes to afford ALK-11 as a white/brown solid (18.6 mg, 52%).

^{1}H NMR (850 MHz, Chloroform-d) δ 7.83 (d, J = 8.4 Hz, 1H), 7.79 (d, J = 8.1 Hz, 1H), 7.54 (s, 1H), 7.46 – 7.42 (m, 1H), 7.43 – 7.39 (m, 1H), 7.36 (dd, J = 7.8, 7.8 Hz, 1H), 7.27 (d, J = 7.6 Hz, 1H), 6.69 (d, J = 7.5 Hz, 1H), 4.37 (s, 2H), 2.31 (s, 3H).

^{13}C NMR (214 MHz, Chloroform-d) δ 143.55, 134.41, 133.53, 128.72, 126.68, 125.92, 124.89, 123.78, 120.31, 117.96, 104.80, 40.52, 10.56.

ESI-HRMS m/z: $[M+H]^+$ Calcd for $C_{15}H_{15}N_3$ 238.1339; found 238.1336

4-((naphthalen-1-ylamino)methyl)phenol (ALK-12)

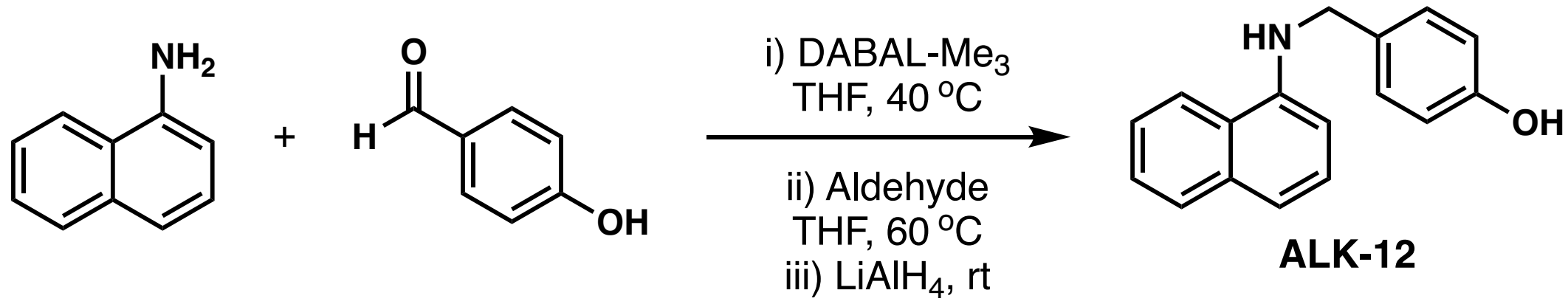

General Procedure E: Reaction scale: 21.5 mg (0.150 mmol) of 1-naphthylamine. Purified by flash chromatography (SiO2, EtOAc/hexane, gradient from 30 to 70% in EtOAc) to afford ALK-12 as a brown oil (28.7 mg, 77%).

^{1}H NMR (500 MHz, Acetone-d_6) δ 8.26 (s, 1H), 8.12 (d, J = 7.1 Hz, 1H), 7.73 (d, J = 7.6 Hz, 1H), 7.43 – 7.33 (m, 2H), 7.26 (d, J = 8.3 Hz, 2H), 7.20 (dd, J = 7.9, 7.9 Hz, 1H), 7.11 (d, J = 8.1 Hz, 1H), 6.78 (d, J = 8.4 Hz, 2H), 6.51 (d, J = 7.6 Hz, 1H), 5.96 (s, 1H), 4.41 (d, J = 3.7 Hz, 2H).

^{13}C NMR (126 MHz, Acetone-d_6) δ 157.24, 144.76, 135.42, 131.33, 129.39 (2C), 129.08, 127.48, 126.31, 124.99, 124.47, 121.75, 117.07, 116.02 (2C), 105.02, 47.87.

ESI-HRMS m/z: $[M+H]^+$ Calcd for $C_{17}H_{15}NO$ 250.1226; found 250.1224

***N*-(4-(dimethylamino)benzyl)naphthalen-1-amine (ALK-13)**

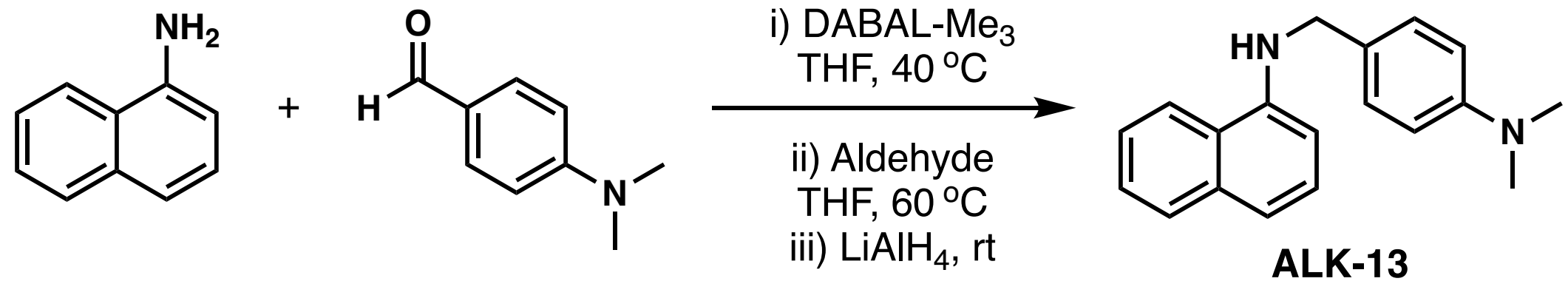

ALK-13

General Procedure E: Reaction scale: 21.5 mg (0.150 mmol) of 1-naphthylamine. Purified by pTLC (EtOAc/hexane, 20:80) to afford ALK-13 as a yellow oil (22.3 mg, 54%).

^{1}H NMR (500 MHz, Acetone-d_6) δ 8.15 (d, J = 8.2 Hz, 1H), 7.77 (d, J = 7.7 Hz, 1H), 7.45 – 7.36 (m, 2H), 7.29 (d, J = 8.5 Hz, 2H), 7.24 (dd, J = 7.9, 7.9 Hz, 1H), 7.14 (d, J = 8.1 Hz, 1H), 6.72 (d, J = 8.6 Hz, 2H), 6.57 (d, J = 7.6 Hz, 1H), 5.92 (s, 1H), 4.42 (d, J = 5.4 Hz, 2H), 2.89 (s, 6H).

^{13}C NMR (126 MHz, Acetone-d_6) δ 150.88, 144.87, 135.42, 129.08, 129.06 (2C), 128.12, 127.51, 126.29, 124.96, 124.48, 121.76, 116.97, 113.47 (2C), 105.01, 47.96, 40.75 (2C).

ESI-HRMS m/z: $[M+H]^+$ Calcd for $C_{19}H_{20}N_2$ 277.1699; found 277.1699

***N*-(4-methoxybenzyl)naphthalen-1-amine (ALK-14)**

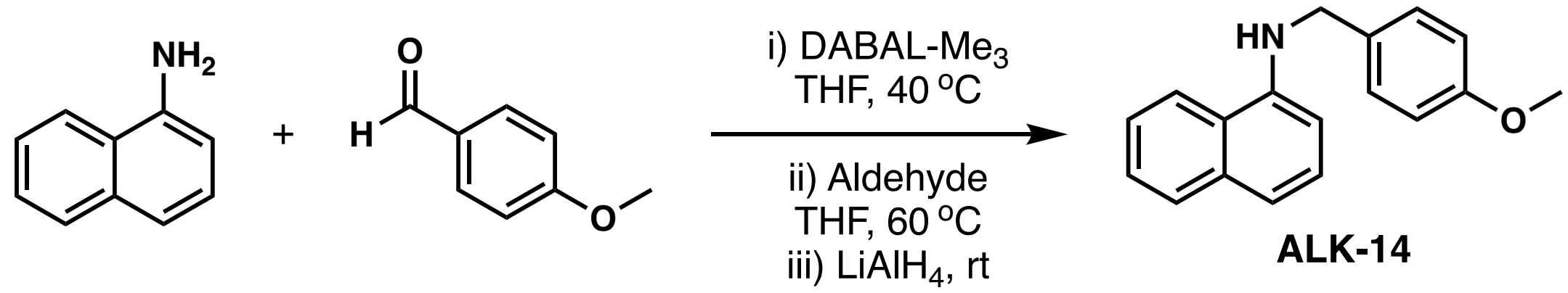

ALK-14

General Procedure E: Reaction scale: 21.5 mg (0.150 mmol) of 1-naphthylamine. Purified by flash chromatography (SiO2, EtOAc/hexane, gradient from 5 to 10% in EtOAc) to afford ALK-14 as a yellow oil (24.7 mg, 62%).

^{1}H NMR (300 MHz, Acetone-d_6) δ 8.20 – 8.13 (m, 1H), 7.85 – 7.69 (m, 1H), 7.54 – 7.30 (m, 4H), 7.23 (dd, J = 7.8, 7.8 Hz, 1H), 7.15 (d, J = 8.0 Hz, 1H), 6.88 (d, J = 8.4 Hz, 2H), 6.53 (d, J = 7.4 Hz, 1H), 6.07 (s, 1H), 4.49 (d, J = 5.3 Hz, 2H), 3.76 (s, 3H).

^{13}C NMR (75 MHz, Acetone-d_6) δ 159.66, 144.69, 135.44, 132.58, 129.27 (2C), 129.11, 127.47, 126.34, 125.03, 124.49, 121.75, 117.14, 114.58 (2C), 105.07, 55.41, 47.72.

ESI-HRMS m/z: $[M+H]^+$ Calcd for $C_{18}H_{17}NO$ 264.1383; found 264.1379

***N*-(benzo[*b*]thiophen-2-ylmethyl)naphthalen-1-amine (ALK-15)**

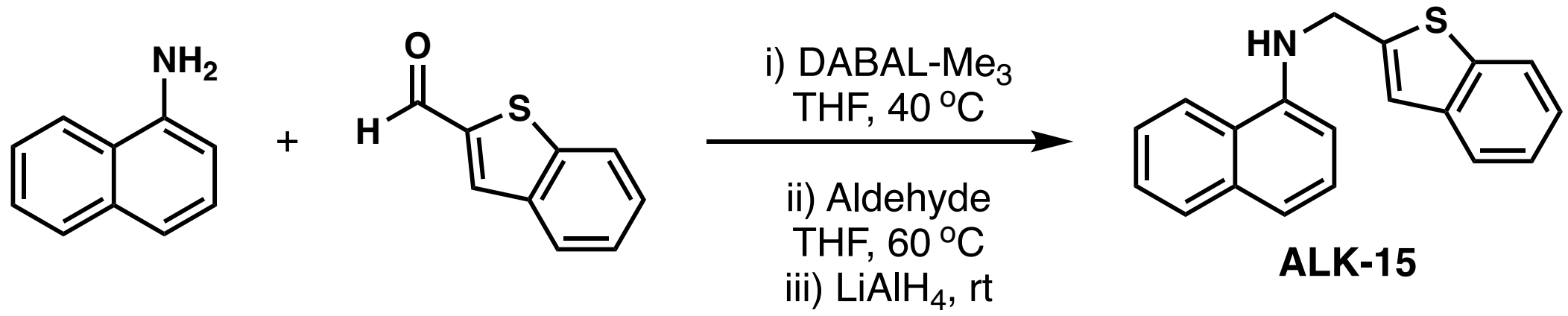

ALK-15

General Procedure E: Reaction scale: 21.5 mg (0.150 mmol) of 1-naphthylamine. Purified by flash chromatography (SiO2, EtOAc/hexane, gradient from 5 to 10% in EtOAc) to afford ALK-15 as a yellow/white solid (28.4 mg, 65%).

^{1}H NMR (400 MHz, Acetone-d_6) δ 8.19 (d, J = 7.8 Hz, 1H), 8.04 (m, 1H), 7.96 (m, 1H), 7.79 (d, J = 7.4 Hz, 1H), 7.56 (s, 1H), 7.42 (m, 4H), 7.27 (dd, J = 7.8, 7.8 Hz, 1H), 7.19 (d, J = 8.0 Hz, 1H), 6.69 (d, J = 7.4 Hz, 1H), 6.13 (s, 1H), 4.82 (d, J = 5.1 Hz, 2H) ppm.

^{13}C NMR (75 MHz, Acetone-d_6) δ 144.73, 141.65, 139.27, 135.47, 135.09, 129.13, 127.51, 126.41, 125.29, 125.13, 124.93, 124.59, 124.22, 123.67, 122.88, 121.85, 117.46, 105.05, 43.11.

ESI-HRMS m/z: $[M+H]^+$ Calcd for $C_{19}H_{15}NS$ 290.0998; found 290.0995

***N*-(4-(trifluoromethyl)benzyl)naphthalen-1-amine (ALK-16)**

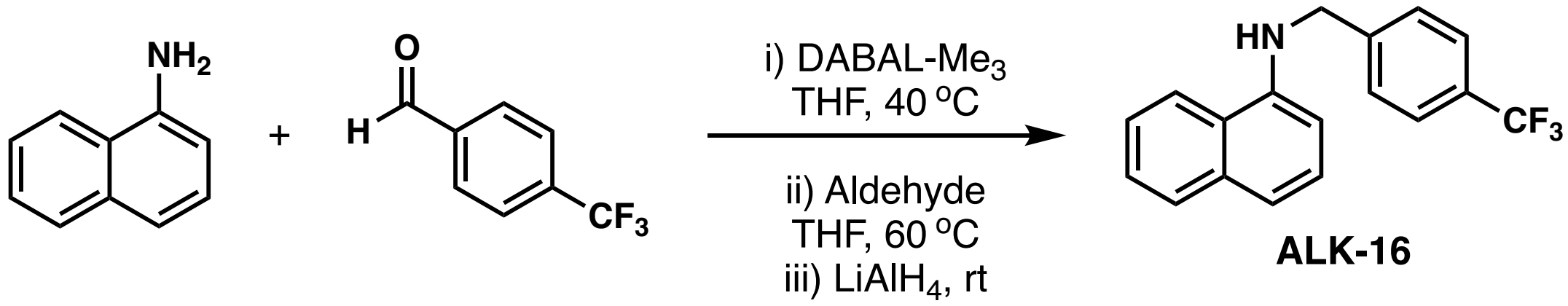

General Procedure E: Reaction scale: 21.5 mg (0.150 mmol) of 1-naphthylamine. Purified by flash chromatography (SiO2, EtOAc/hexane, gradient from 5 to 10% in EtOAc) to afford ALK-16 as a yellow oil (34.8 mg, 77%).

^{1}H NMR (300 MHz, Acetone-d_6) δ 8.22 – 8.14 (m, 1H), 7.84 – 7.71 (m, 1H), 7.63 (s, 4H), 7.48 – 7.35 (m, 2H), 7.24 – 7.07 (m, 2H), 6.48 – 6.31 (m, 2H), 4.67 (d, J = 5.5 Hz, 2H).

^{13}C NMR (214 MHz, Chloroform-d) δ 143.49, 142.82, 134.46, 129.76 (q, J = 32.4 Hz), 128.95, 127.77, 126.63, 126.03, 125.11, 124.33 (q, J = 271.7 Hz), 123.53, 119.89, 118.25, 105.14, 48.17.

^{19}F NMR (376 MHz, Chloroform-d) δ -62.40.

ESI-HRMS m/z: $[M+H]^+$ Calcd for $C_{18}H_{14}NF_3$ 302.1151; found 302.1149

***N*-((2',4'-difluoro-[1,1'-biphenyl]-4-yl)methyl)naphthalen-1-amine (ALK-17)**

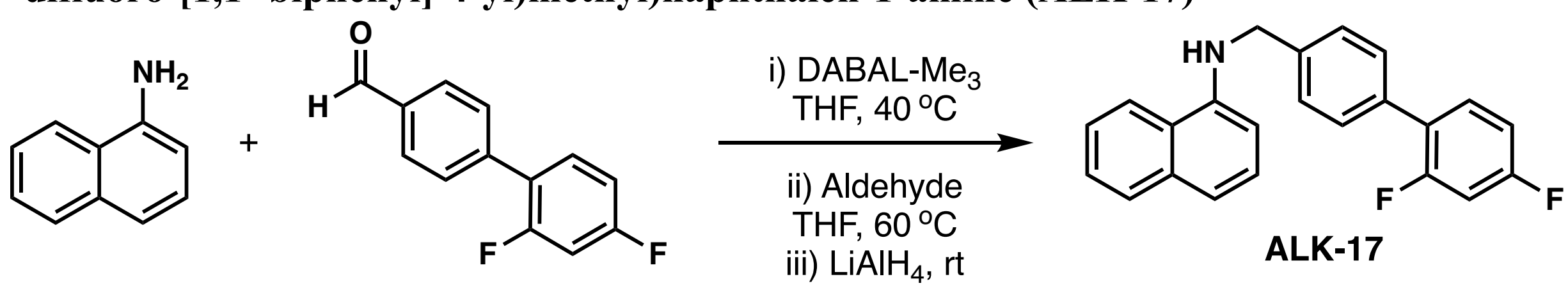

General Procedure E: Reaction scale: 21.5 mg (0.150 mmol) of 1-naphthylamine. Purified by flash chromatography (SiO2, EtOAc/hexane, gradient from 5 to 10% in EtOAc) to afford ALK-17 as a yellow oil (34.5 mg, 66%).

^{1}H NMR (300 MHz, Acetone-d_6) δ 8.19 (m, 1H), 7.75 (m, 1H), 7.48 (m, 7H), 7.11 (m, 4H), 6.51 (d, J = 7.2 Hz, 1H), 6.27 (s, 1H), 4.61 (d, J = 5.5 Hz, 2H) ppm.

^{19}F NMR (376 MHz, Chloroform-d) δ -111.38 (d, J = 7.4 Hz), -113.49 (d, J = 7.4 Hz).

ESI-HRMS m/z: $[M+H]^+$ Calcd for $C_{23}H_{17}NF_2$ 346.1402; found 346.1399

N-(3-(isoindolin-2-yl)propyl)naphthalen-1-amine (ALK-18)

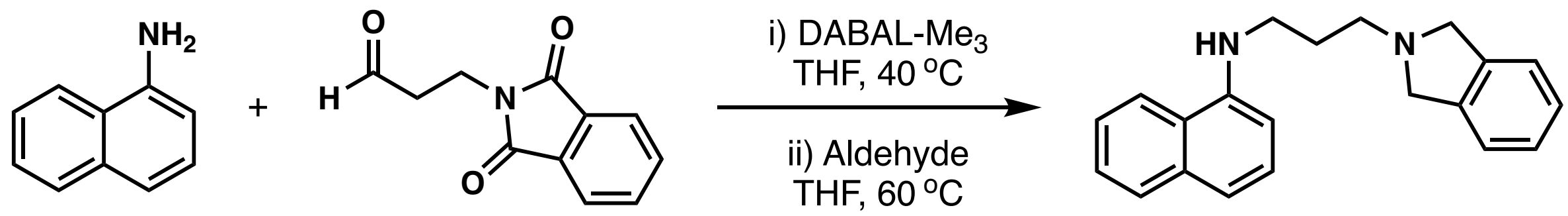

ALK-18

General Procedure E: Reaction scale: 12.5 mg (0.088 mmol) of 1-naphthylamine. Purified by flash chromatography (SiO2, EtOAc/hexane, gradient from 10 to 60% in EtOAc) to afford ALK-18 as a brown oil (5.2 mg, 20%).

^{1}H NMR (500 MHz, Acetone-d_6) δ 7.70 (d, J = 8.2 Hz, 1H), 7.67 (d, J = 8.6 Hz, 1H), 7.35 – 7.28 (m, 2H), 7.31 – 7.21 (m, 5H), 7.09 (d, J = 8.2 Hz, 1H), 6.99 (dd, J = 7.7, 7.7 Hz, 1H), 6.68 (s, 1H), 6.53 (d, J = 7.6 Hz, 1H), 4.01 (s, 4H), 3.42 (t, J = 6.3 Hz, 2H), 3.02 (t, J = 6.2 Hz, 2H), 2.08 (t, J = 6.2 Hz, 2H).

^{13}C NMR (125 MHz, Acetone-d_6) δ 145.8, 141.3 (2C), 135.4, 129.0, 127.7, 127.5 (2C), 126.2, 124.7, 124.5, 123.1 (2C), 121.8, 116.5, 103.7, 59.6 (2C), 55.5, 44.5, 27.7 ppm

ESI-HRMS m/z: $[M+H]^+$ Calcd for $C_{21}H_{22}N_2$ 303.1856; found 303.1850

N-(4-(3,5-dimethyl-1H-pyrazol-1-yl)benzyl)naphthalen-1-amine (ALK-19)

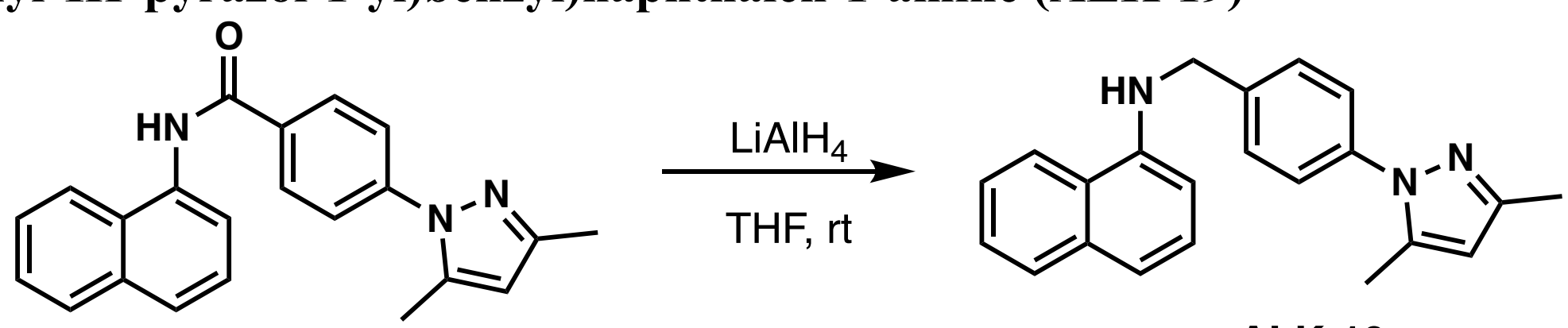

ALK-19

General Procedure G: Reaction scale: 20.0 mg (0.059 mmol) of starting material. Purified by flash chromatography (SiO2, EtOAc/hexane, gradient from 20 to 60% in EtOAc) to afford ALK-19 as a white/yellow solid (4.5 mg, 23%).

^{1}H NMR (500 MHz, Acetone-d_6) 8.22 (d, J = 7.7 Hz, 1H), 7.79 (d, J = 7.7 Hz, 1H), 7.57 (d, J = 8.0 Hz, 2H), 7.45 (d, J = 7.6 Hz, 4H), 7.23 (dd, J = 7.8, 7.8 Hz, 1H), 7.17 (d, J = 8.1 Hz, 1H), 6.54 (d, J = 7.5 Hz, 1H), 6.30 (s, 1H), 6.00 (s, 1H), 4.65 (d, J = 5.4 Hz, 2H), 2.30 (s, 3H), 2.18 (s, 3H) ppm.

^{13}C NMR (125 MHz, Acetone-d_6) δ 148.9, 144.6, 139.8, 139.7 (2C), 135.5, 129.2, 128.5 (2C), 127.5, 126.4, 125.2, 125.1 (2C), 124.6, 121.8, 117.4, 107.7, 105.1, 47.8, 13.6, 12.6 ppm.

ESI-HRMS m/z: $[M+H]^+$ Calcd for $C_{22}H_{21}N_3$ 328.1808; found 328.1803

2-(3-(naphthalen-1-ylamino)propyl)isoindoline-1,3-dione (ALK-20)

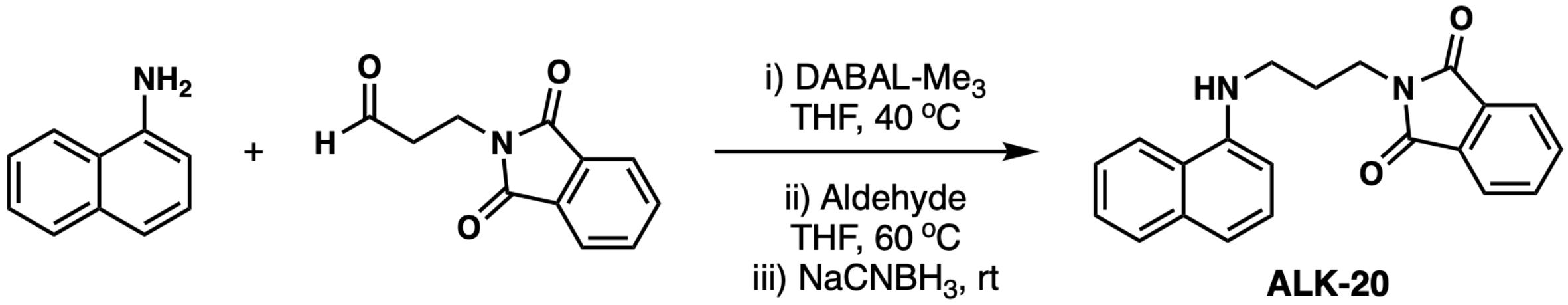

General Procedure E: Modified to use $NaCNBH_3$ as the reductant. Reaction scale: 12.5 mg (0.088 mmol) of 1-naphthylamine. Purified by flash chromatography (SiO2, EtOAc/hexane, gradient from 10 to 60% in EtOAc) to afford ALK-20 as a yellow oil (16.3 mg, 57%).

^{1}H NMR (500 MHz, Acetone-d_6) δ 8.08 (d, J = 8.1 Hz, 1H), 7.92 – 7.79 (m, 4H), 7.75 (d, J = 7.8 Hz, 1H), 7.40 (p, J = 6.5 Hz, 2H), 7.28 (dd, J = 7.9, 7.9 Hz, 1H), 7.14 (d, J = 8.1 Hz, 1H), 6.60 (d, J = 7.6 Hz, 1H), 5.59 (s, 1H), 3.86 (t, J = 6.7 Hz, 2H), 3.41 (q, J = 6.4 Hz, 2H), 2.14 (p, J = 6.7 Hz, 2H).

^{13}C NMR (126 MHz, Acetone-d_6) δ 169.09, 144.75, 135.52, 134.96, 133.18, 129.09, 127.57, 126.34, 125.00, 124.56, 123.73, 121.64, 117.10, 104.37, 41.75, 36.47, 28.33.

ESI-HRMS m/z: $[M+H]^+$ Calcd for $C_{21}H_{18}N_2O_2$ 331.1441; found 331.1436

***N*-(4-ethylphenyl)naphthalen-1-amine (AR-01)**

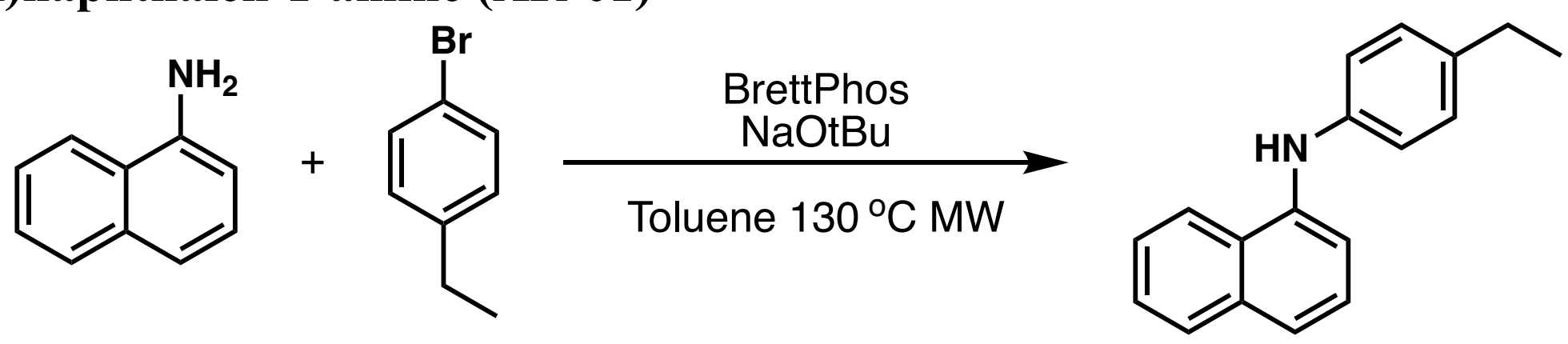

AR-01

General Procedure H: Reaction scale: 257 mg (1.80 mmol) of naphthalen-1-amine. Yields AR-01 as red oil (213 mg, 48%).

^{1}H NMR (500 MHz, Acetone-d_6) δ 8.19 (d, J = 8.3 Hz, 1H), 7.87 (d, J = 7.9 Hz, 1H), 7.47 (m, 3H), 7.38 (m, 2H), 7.31 (d, J = 7.4 Hz, 1H), 7.11 (d, J = 8.3 Hz, 2H), 7.05 (d, J = 8.3 Hz, 2H), 2.58 (q, J = 7.6 Hz, 2H), 1.20 (t, J = 7.6 Hz, 3H) ppm.

^{13}C NMR (125 MHz, Acetone-d_6) δ 143.5, 141.3, 137.0, 135.8, 129.3 (2C), 129.1, 128.0, 127.0, 126.8, 125.8, 123.2, 122.0, 119.2 (2C), 114.0, 28.7, 16.4 ppm.

ESI-HRMS m/z: $[M+H]^+$ Calcd for $C_{18}H_{17}N$ 248.1434; found 248.1431

***N*-(4-fluorophenyl)naphthalen-1-amine (AR-02)**

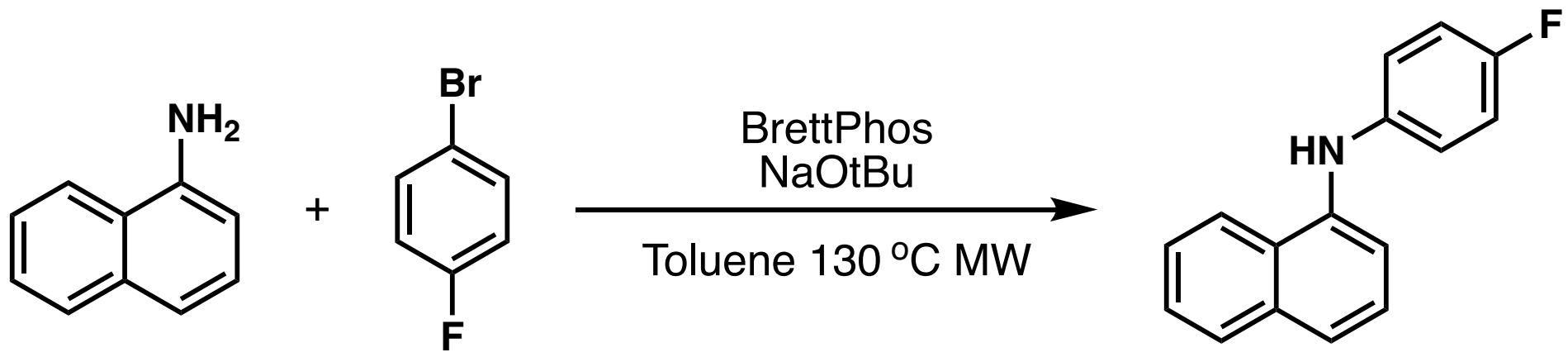

AR-02

General Procedure H: Reaction scale: 200 mg (1.40 mmol) of naphthalen-1-amine. Yields AR-02 as White solid (191 mg, 58%).

^{1}H NMR (400 MHz, Acetone-d_6) δ 8.17 (d, J = 8.2 Hz, 1H), 7.89 (dd, J = 7.7, 1.7 Hz, 1H), 7.52 (m, 2H), 7.47 (m, 2H), 7.39 (t, J = 7.8, 7.8 Hz, 1H), 7.27 (d, J = 7.5 Hz, 1H), 7.11 (m, 2H), 7.03 (dd, J = 9.7, 7.8 Hz, 2H).

^{13}C NMR (214 MHz, Acetone-d_6) δ 158.39 (d, J = 236.7 Hz), 142.62 (d, J = 18.6 Hz), 141.31 (d, J = 16.4 Hz), 136.01, 129.26, 128.40 (d, J = 4.6 Hz), 127.03, 126.93, 126.11, 123.25 (d, J = 3.1 Hz), 122.78, 120.64, 120.59 (d, J = 7.7 Hz), 116.44 (d, J = 22.6 Hz), 114.95 (d, J = 12.6 Hz).

^{19}F NMR (376 MHz, $CDCl_3$) δ -122.75.

ESI-HRMS m/z: $[M+H]^+$ Calcd for $C_{16}H_{12}FN$ 238.1027; found 238.1024

***tert*-butyl (4-(naphthalen-1-ylamino)benzyl)carbamate (AR-03)**

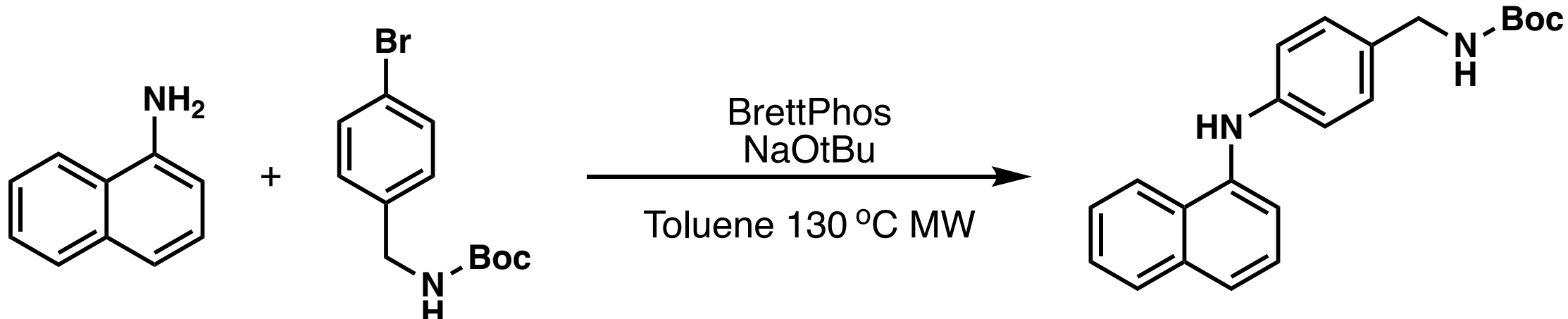

AR-03

General Procedure H: Reaction scale: 200 mg (1.40 mmol) of naphthalen-1-amine. Yields AR-03 as White solid (230 mg, 47%).

^{1}H NMR (400 MHz, Acetone-d_6) δ 8.17 (dd, J = 8.3, 1.6 Hz, 1H), 7.88 (dd, J = 7.9, 1.6 Hz, 1H), 7.54 (d, J = 8.0 Hz, 1H), 7.48 (m, 2H), 7.40 (t, J = 7.7, 7.7 Hz, 1H), 7.35 (dt, J = 7.5, 1.4, 1.4 Hz, 1H), 7.19 (d, J = 8.5 Hz, 2H), 7.04 (m, 2H), 6.35 (s, 1H), 4.20 (d, J = 6.2 Hz, 2H), 1.42 (s, 9H).

^{13}C NMR (214 MHz, Acetone) δ 156.89, 145.26, 140.90, 136.03, 132.94, 129.29 (2C), 129.24, 128.75, 127.01, 126.90, 126.08, 123.45, 122.94, 118.53 (2C), 115.72, 78.80, 44.70, 28.80 (3C).

ESI-HRMS m/z: $[M+H]^+$ Calcd for $C_{22}H_{24}N_2O_2$ 349.1911; found 349.1907

***N*-(4-(aminomethyl)phenyl)naphthalen-1-amine (AR-04)**

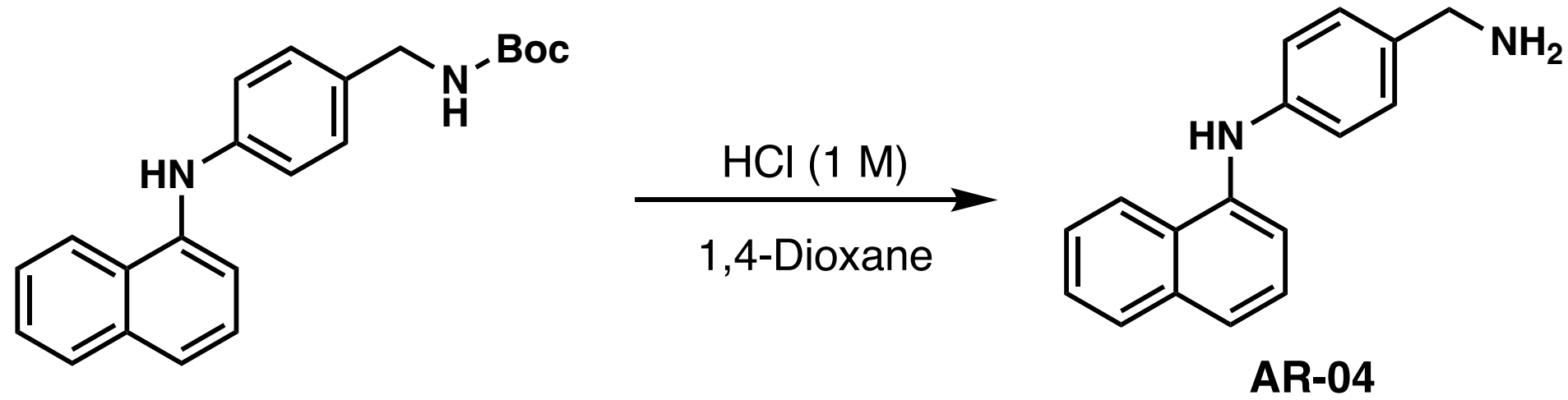

AR-04

Procedure*: tert*-butyl (4-(naphthalen-1-ylamino)benzyl)carbamate (.050 g, 1 Eq, 0.14 mmol) was dissolved in 1,4-Dioxane (3 mL)followed by 4M HCl in 1,4-Dioxane (1 mL) reaction was stirred at room temperature for 4 hours. Reaction was diluted with EtOAc and quenched with sat. $NaHCO_3$. The aqueous layer was extracted 3 times with EtOAc. The organic layers were combined, dried with Na_2SO_4, and concentrated under vacuum to yield N-(4-(aminomethyl)phenyl)naphthalen-1-amine (.020 g, 81 μmol, 56 %) as a yellow oil.

^{1}H NMR (850 MHz, Chloroform-*d*) δ 8.02 (d, *J* = 8.4 Hz, 1H), 7.87 (d, *J* = 8.2 Hz, 1H), 7.56 (d, *J* = 8.2 Hz, 1H), 7.49 (m, 3H), 7.41 – 7.37 (m, 1H), 7.35 (d, *J* = 7.5 Hz, 1H), 7.21 (d, *J* = 8.2 Hz, 2H), 6.99 (d, *J* = 8.0 Hz, 2H), 5.97 (s, 1H), 3.81 (s, 2H).

^{13}C NMR (214 MHz, Chloroform-*d*) δ 143.37, 138.95, 135.67, 134.68, 128.67, 128.37 (2C), 127.43, 126.26, 126.17, 125.79, 122.78, 121.77, 117.81 (2C), 115.19, 46.16.

ESI-HRMS m/z: $[M-NH_2]^+$ Calcd for $C_{17}H_{14}N$ 232.1121; found 232.1117

***N*-(4-(trifluoromethyl)phenyl)naphthalen-1-amine (AR-05)**

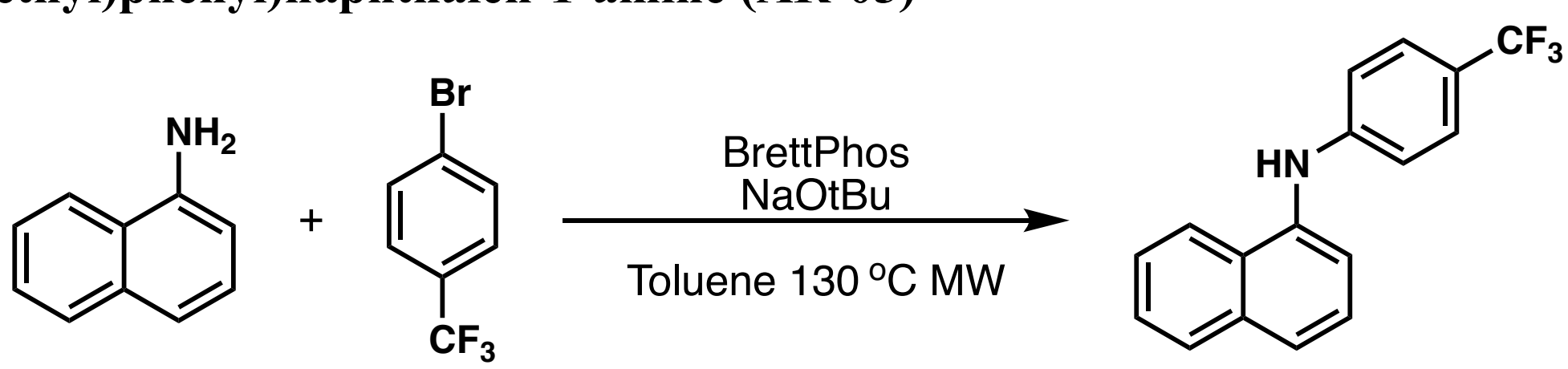

AR-05

General Procedure H: Reaction scale: 125 mg (0.87 mmol) of naphthalen-1-amine. Yields AR-05 as White solid (73 mg, 29%).

^{1}H NMR (400 MHz, Acetone-*d*$_6$) δ 8.10 (dd, *J* = 8.2, 1.6 Hz, 1H), 8.00 (s, 1H), 7.96 (dd, *J* = 7.7, 1.5 Hz, 1H), 7.76 (p, *J* = 3.8, 3.8, 3.8, 3.8 Hz, 1H), 7.51 (m, 6H), 7.05 (dd, *J* = 9.0, 2.9 Hz, 2H).

^{13}C NMR (101 MHz, $CDCl_3$) δ 148.85, 136.87, 134.90, 129.21, 128.76, 126.81 (q, J = 3.8 Hz, 2C), 126.55, 126.39, 126.07, 125.47, 124.85 (q, J = 270.8 Hz), 122.34, 121.20 (q, J = 32.7 Hz, 2C), 120.05, 114.94.

^{19}F NMR (376 MHz, DMSO) δ -59.35.

ESI-HRMS m/z: $[M+H]^+$ Calcd for $C_{17}H_{12}F_3N$ 288.0995; found 288.0996

Di(naphthalen-1-yl)amine (AR-06)

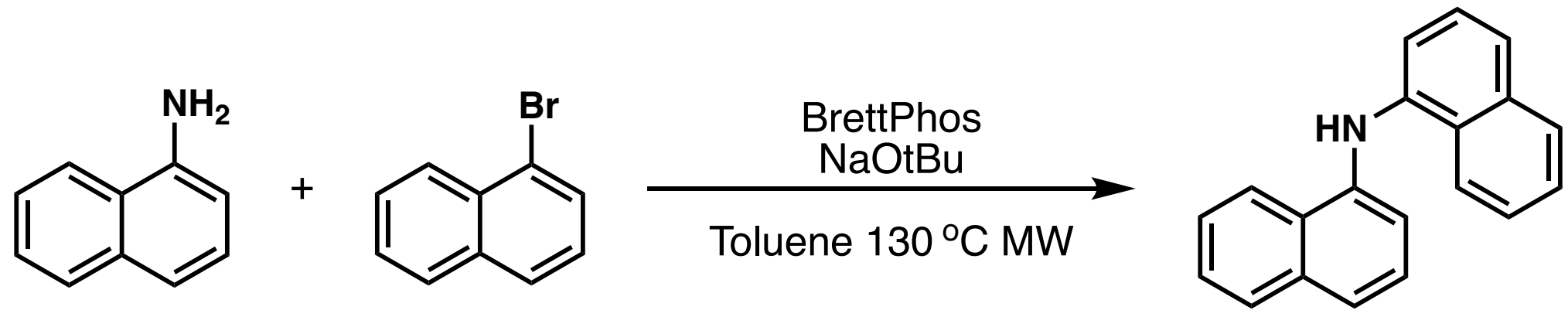

AR-06

General Procedure H: Reaction scale: 200 mg (1.40 mmol) of naphthalen-1-amine. Yields AR-06 as red solid (137 mg, 36%).

^{1}H NMR (400 MHz, Acetone-d_6) δ 8.24 (dd, J = 8.6, 1.2 Hz, 2H), 7.92 (dd, J = 8.1, 1.4 Hz, 2H), 7.67 (s, 1H), 7.56 (d, J = 8.2 Hz, 2H), 7.49 (m, 4H), 7.35 (dd, J = 7.8, 7.8 Hz, 2H), 6.95 (d, J = 7.5 Hz, 2H).

^{13}C NMR (214 MHz, Acetone) δ 142.40, 142.32, 135.95 (2C), 129.28 (2C), 128.37 (2C), 127.11 (2C), 126.89 (2C), 126.15 (2C), 123.64, 123.63, 122.89 (2C), 116.53, 116.50.

ESI-HRMS m/z: $[M+H]^+$ Calcd for $C_{20}H_{15}N$ 270.1277; found 270.1275

***N*-(4-methoxyphenyl)naphthalen-1-amine (AR-07)**

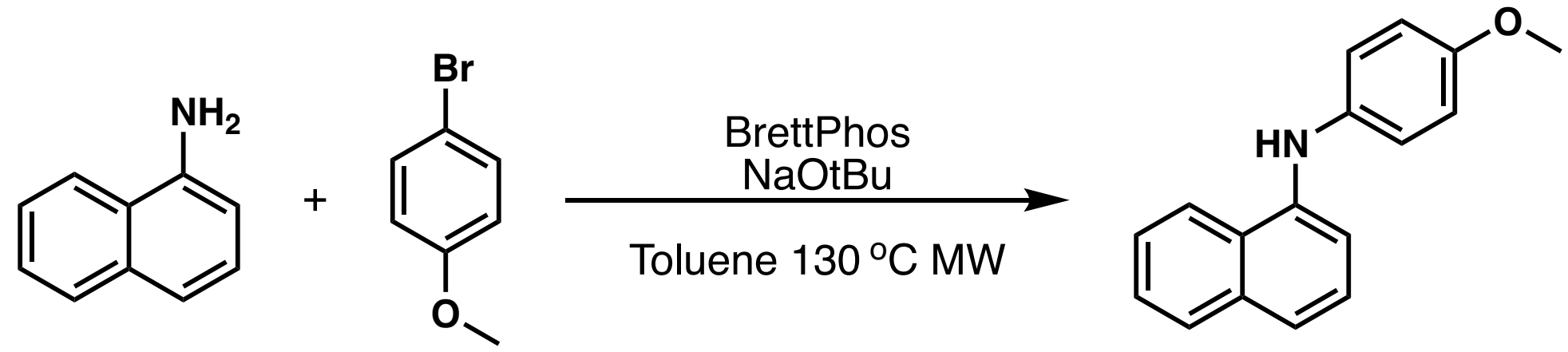

AR-07

General Procedure H: Reaction scale: 200 mg (1.40 mmol) of naphthalen-1-amine. Yields AR-07 as red oil (151 mg, 43%).

^{1}H NMR (400 MHz, Chloroform-d) δ 8.04 – 7.97 (m, 1H), 7.84 (d, J = 7.2 Hz, 1H), 7.55 – 7.42 (m, 3H), 7.33 (dd, J = 8.0, 8.0 Hz, 1H), 7.11 (d, J = 7.1 Hz, 1H), 7.06 (d, J = 8.4 Hz, 2H), 6.89 (d, J = 8.3 Hz, 2H), 5.88 (s, 1H), 3.81 (s, 3H).

^{13}C NMR (101 MHz, Chloroform-d) δ 155.28, 140.95, 136.99, 134.77, 128.74, 126.30, 126.16, 126.10, 125.52, 122.00 (2C), 121.21, 121.13, 114.92 (2C), 111.94, 55.77.

ESI-HRMS m/z: $[M+H]^+$ Calcd for $C_{17}H_{15}NO$ 250.1226; found 250.1224

***N*-(4-(trifluoromethoxy)phenyl)naphthalen-1-amine (AR-08)**

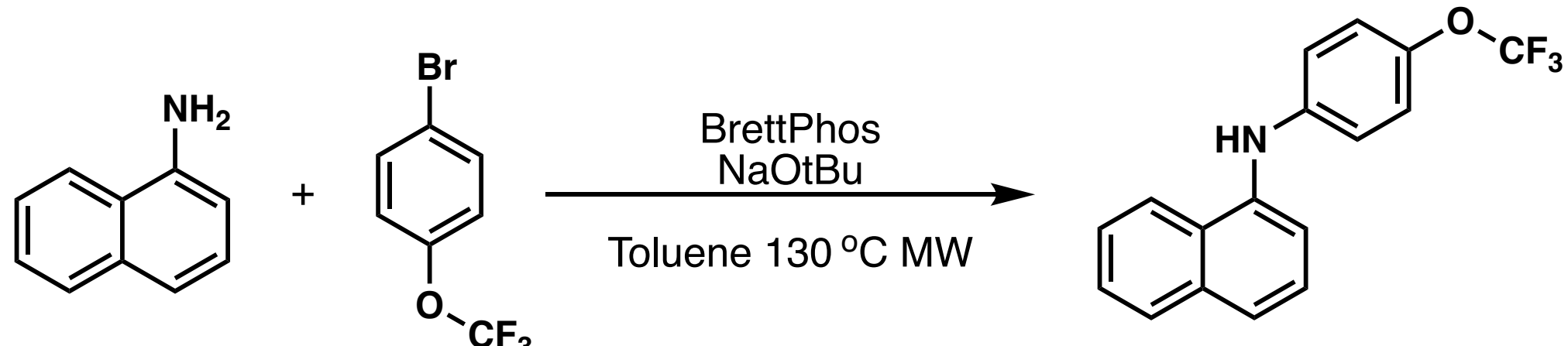

AR-08

General Procedure H: Reaction scale: 200 mg (1.40 mmol) of naphthalen-1-amine. Yields AR-08 as red oil (327 mg, 77%).

^{1}H NMR (850 MHz, DMSO) δ 8.43 (s, 1H), 8.17 (dd, *J* = 7.9, 1.9 Hz, 1H), 7.92 – 7.88 (m, 1H), 7.59 (d, *J* = 8.1 Hz, 1H), 7.51 (pd, *J* = 6.7, 1.6 Hz, 2H), 7.44 – 7.40 (m, 1H), 7.37 (d, *J* = 7.4 Hz, 1H), 7.19 (d, *J* = 8.7 Hz, 2H), 7.10 – 7.06 (m, 2H).

^{13}C NMR (214 MHz, DMSO) δ 144.84, 140.64, 138.66, 134.45, 128.15, 127.51, 126.12, 126.08, 125.29, 122.78, 122.45, 122.08 (2C), 120.34 (q, *J* = 254.7 Hz), 116.95 (2C), 115.69.

^{19}F NMR (376 MHz, DMSO) δ -57.31.

ESI-HRMS m/z: $[M+H]^+$ Calcd for $C_{17}H_{12}NOF_3$ 304.0944; found 304.0941

2,2,2-trifluoro-1-(4-(naphthalen-1-ylamino)phenyl)ethan-1-one (AR-09)

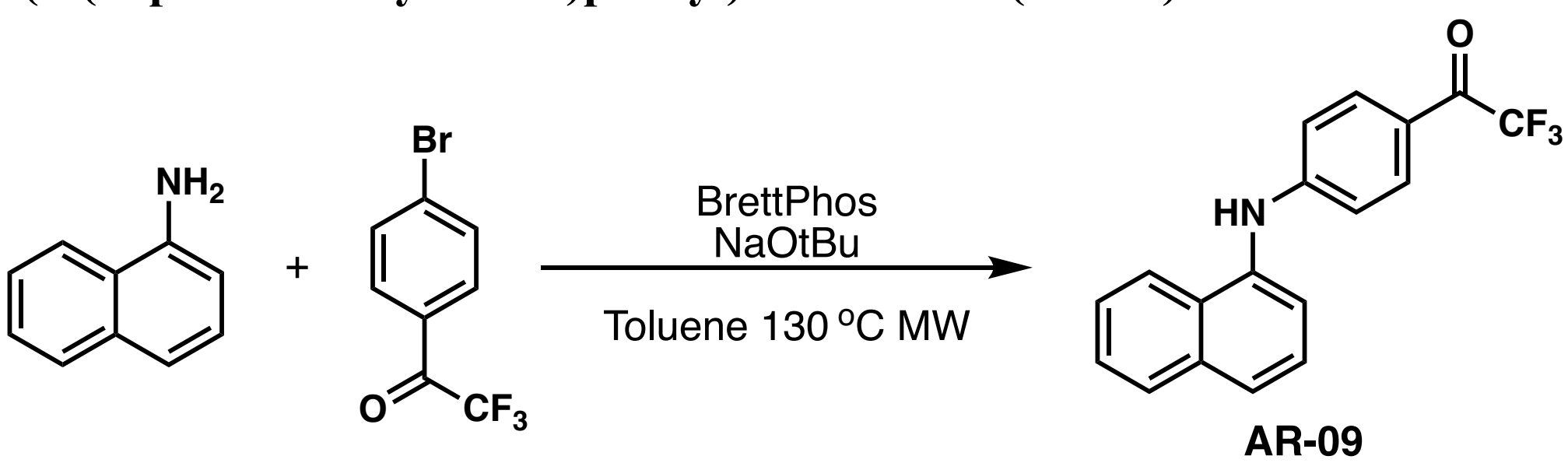

AR-09

General Procedure H: Reaction scale: 200 mg (1.40 mmol) of naphthalen-1-amine. Yields AR-09 as yellow solid (13 mg, 3%).

^{1}H NMR (850 MHz, DMSO) δ 9.50 (s, 1H), 8.02 – 7.99 (m, 1H), 7.99 – 7.96 (m, 1H), 7.89 – 7.85 (m, 3H), 7.60 – 7.54 (m, 3H), 7.52 (d, *J* = 7.2 Hz, 1H), 6.95 – 6.91 (m, 2H).

^{13}C NMR (214 MHz, DMSO) δ 176.45 (q, *J* = 33.0 Hz), 153.88, 135.28, 134.35, 132.65 (2C), 128.88, 128.39, 126.51, 126.30, 126.06, 125.92, 122.72, 121.74, 117.96, 117.12 (q, *J* = 291.9 Hz), 113.57 (2C).

^{19}F NMR (376 MHz, DMSO) δ -69.52.

ESI-HRMS m/z: $[M+H]^+$ Calcd for $C_{18}H_{12}NOF_3$ 316.0944; found 316.0934

***N*-(2-methoxyphenyl)naphthalen-1-amine (AR-10)**

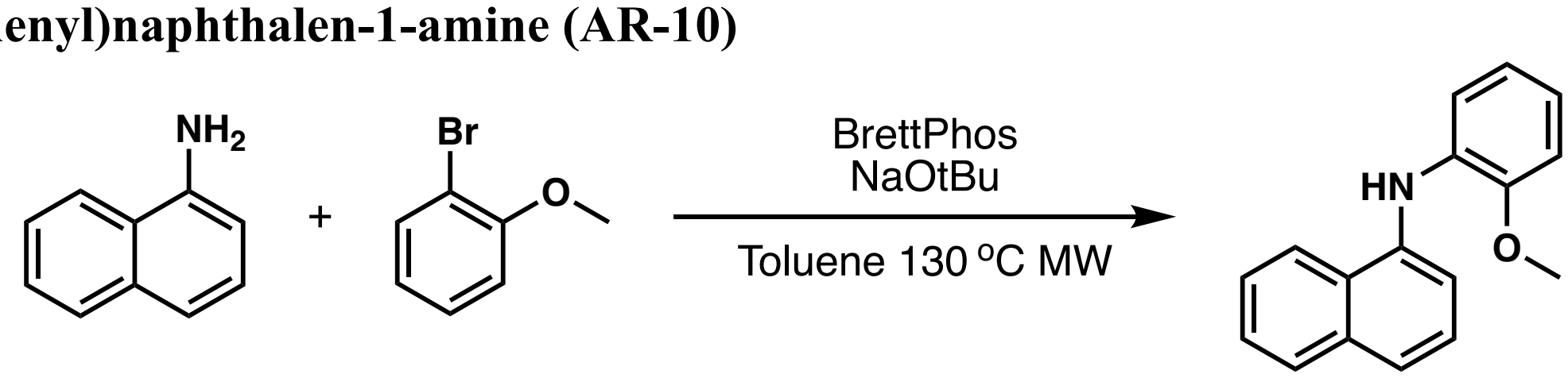

AR-10

General Procedure H: Reaction scale: 200 mg (1.40 mmol) of naphthalen-1-amine. Yields AR-10 as red solid (334 mg, 96%).

^{1}H NMR (400 MHz, DMSO) δ 8.13 – 8.07 (m, 1H), 7.92 – 7.85 (m, 1H), 7.54 – 7.42 (m, 3H), 7.41 – 7.31 (m, 2H), 7.04 (dd, *J* = 8.1, 1.3 Hz, 1H), 7.01 (d, *J* = 7.4 Hz, 1H), 6.95 – 6.86 (m, 1H), 6.87 – 6.76 (m, 2H), 3.84 (s, 3H).

^{13}C NMR (101 MHz, $CDCl_3$) δ 148.17, 138.55, 134.85, 134.60, 128.59, 128.35, 126.18, 126.12, 125.75, 123.04, 122.17, 121.04, 119.58, 116.29, 114.89, 110.55, 55.81.

ESI-HRMS m/z: $[M+H]^+$ Calcd for $C_{17}H_{15}NO$ 250.1226; found 250.1224

1-(3-(naphthalen-1-ylamino)phenyl)propan-1-one (AR-11)

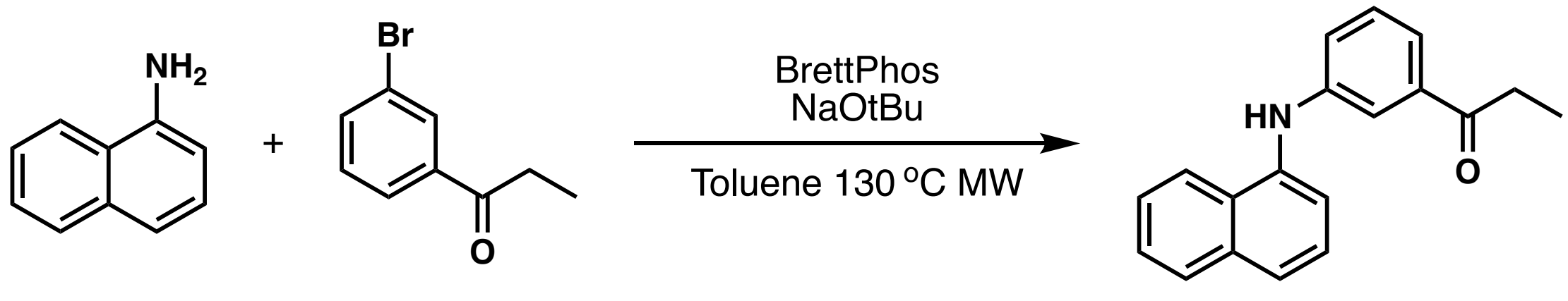

AR-11

General Procedure H: Reaction scale: 200 mg (1.40 mmol) of naphthalen-1-amine. Yields AR-11 as tan solid (223 mg, 58%).

^{1}H NMR (400 MHz, DMSO) δ 8.45 (s, 1H), 8.17 – 8.10 (m, 1H), 7.96 – 7.89 (m, 1H), 7.62 – 7.32 (m, 8H), 7.23 (m, 1H), 2.97 (q, J = 7.2 Hz, 2H), 1.06 (t, J = 7.2 Hz, 3H).

^{13}C NMR (101 MHz, DMSO) δ 200.93, 146.24, 139.07, 138.03, 134.91, 129.90, 128.68, 127.84, 126.67, 126.63, 125.81, 123.22, 122.83, 121.07, 119.34, 115.84, 115.44, 31.70, 8.67.

ESI-HRMS m/z: $[M+H]^+$ Calcd for $C_{19}H_{17}NO$ 276.1383; found 276.1378

Methyl 3-(naphthalen-1-ylamino)benzoate (AR-12)

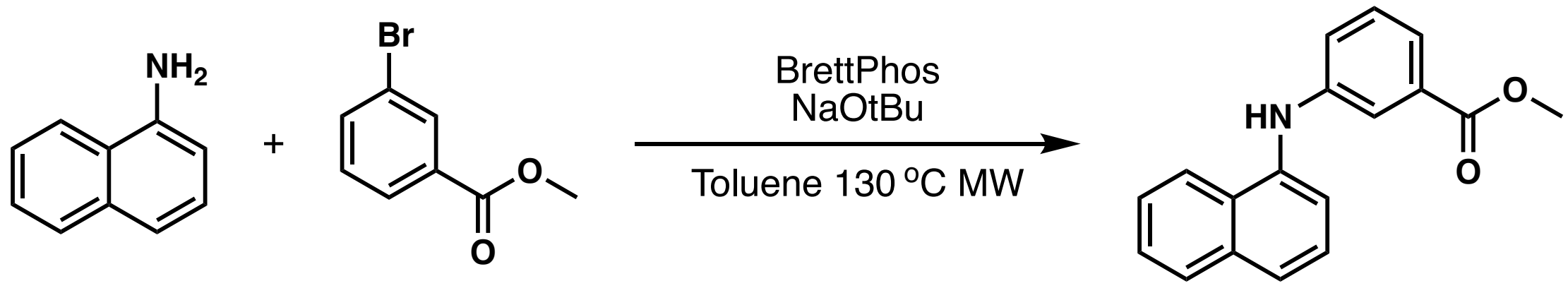

AR-12

General Procedure H: Reaction scale: 200 mg (1.40 mmol) of naphthalen-1-amine. Yields AR-12 as tan solid (194 mg, 50%).

^{1}H NMR (400 MHz, DMSO) δ 8.47 (s, 1H), 8.14 – 8.09 (m, 1H), 7.95 – 7.91 (m, 1H), 7.65 – 7.58 (m, 2H), 7.56 – 7.50 (m, 2H), 7.45 (t, J = 7.8 Hz, 1H), 7.39 – 7.33 (m, 3H), 7.23 (m, 1H), 3.80 (s, 3H).

^{13}C NMR (101 MHz, DMSO) δ 166.44, 146.00, 138.41, 134.46, 130.50, 129.51, 128.24, 127.60, 126.26, 126.17, 125.46, 122.79, 122.71, 120.50, 119.57, 116.33, 116.02, 52.08.

ESI-HRMS m/z: $[M+H]^+$ Calcd for $C_{18}H_{15}NO_2$ 278.1176; found 278.1172

_N_-(3,5-dichlorophenyl)naphthalen-1-amine (AR-13)

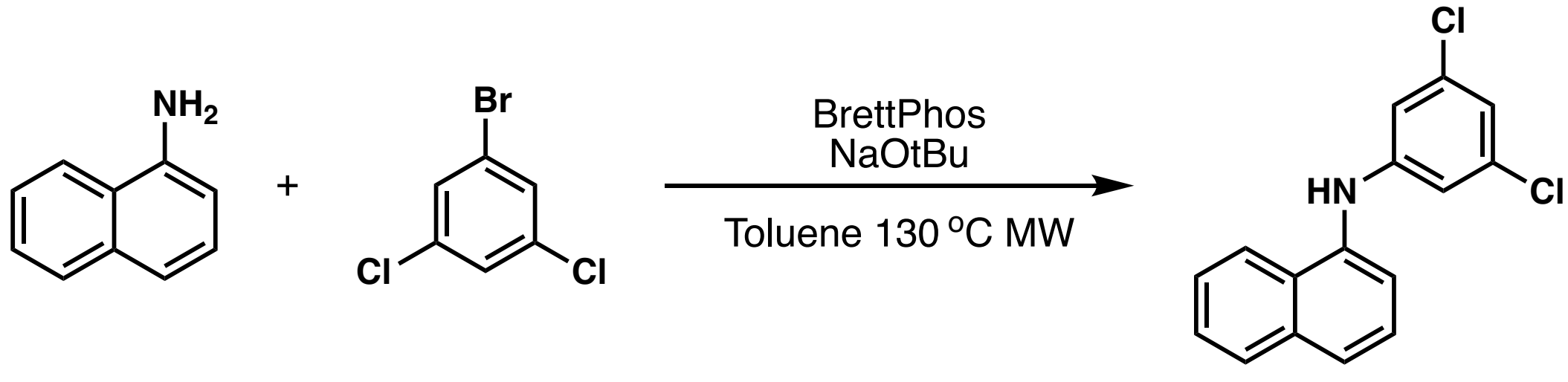

AR-13

General Procedure H: Reaction scale: 200 mg (1.40 mmol) of naphthalen-1-amine. Yields AR-13 as white solid (214 mg, 53%).

^{1}H NMR (400 MHz, $CDCl_3$) δ 7.73 (d, J = 7.5 Hz, 2H), 7.53 (dd, J = 8.3, 1.2 Hz, 1H), 7.37 – 7.26 (m, 3H), 7.18 (dd, J = 7.4, 1.2 Hz, 1H), 6.65 (dd, J = 1.8, 1.8 Hz, 1H), 6.51 (d, J = 1.8 Hz, 2H), 5.60 (s, 1H).

^{13}C NMR (101 MHz, $CDCl_3$) δ 147.84, 136.41, 135.63 (2C), 134.80, 129.02, 128.72, 126.55, 126.43, 126.00, 125.57, 122.04, 120.14, 119.31, 113.76 (2C).

ESI-HRMS m/z: $[M+H]^+$ Calcd for $C_{16}H_{11}NCl_2$ 288.0341; found 288.0335

4-(naphthalen-1-ylamino)benzaldehyde (AR-14)

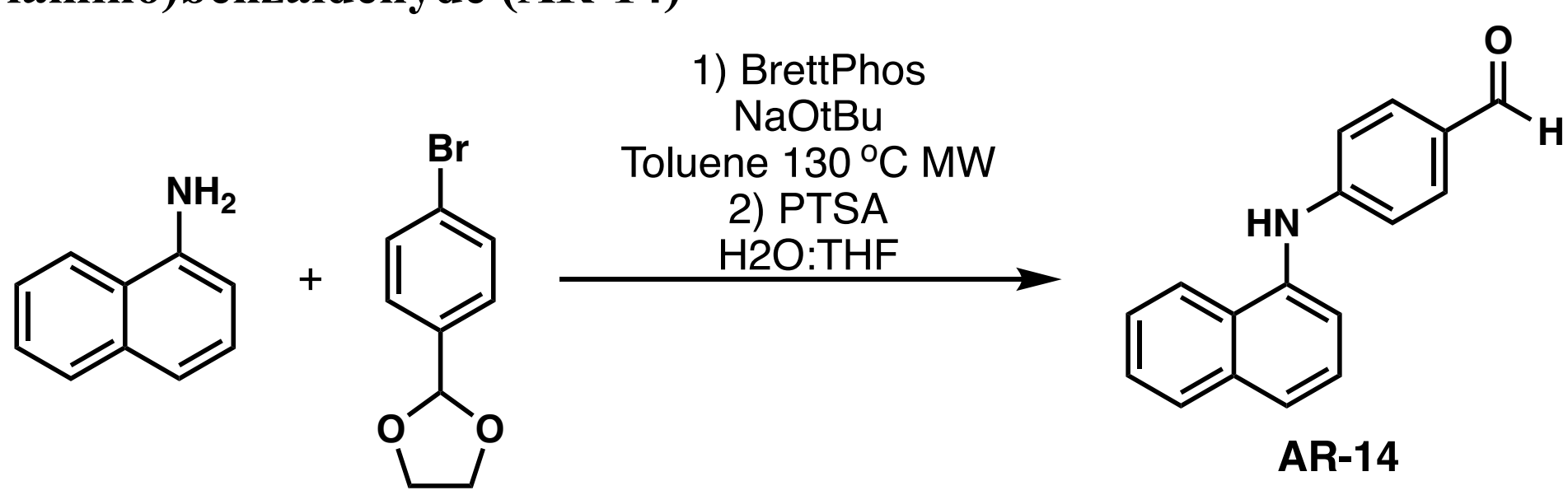

AR-14

Procedure: Following general procedure H: Reaction scale: 200 mg (1.40 mmol) of naphthalen-1-amine. The crude intermediate was dissolved in 0.86 mL of THF and 0.15 mL dI water. PTSA (3.3 mg, 10 mol %) was added. The reaction was stirred for 4 hours at room temp. Reaction was quenched with $NaHCO_3$. An extraction was performed with EthOAc (3x15ml) and the organic phase was washed with brine 3 times. The mixture was dried over sodium sulfate, filtered, and evaporated. The reaction was purified using flash column chromatography. Yields AR-14 as white solid (214 mg, 53%) over 2 steps.

^{1}H NMR (400 MHz, $CDCl_3$) δ 9.78 (s, 1H), 8.02 – 7.94 (m, 1H), 7.92 (dd, J = 8.1, 1.6 Hz, 1H), 7.82 – 7.74 (m, 1H), 7.72 (d, J = 8.6 Hz, 2H), 7.58 – 7.46 (m, 4H), 6.86 (d, J = 8.7 Hz, 2H), 6.40 (s, 1H).

^{13}C NMR (101 MHz, $CDCl_3$) δ 190.52, 151.91, 135.83, 134.88, 132.29 (2C), 129.59, 128.77, 128.29, 126.64, 126.60, 126.38, 126.01, 122.42, 121.70, 114.33 (2C).

ESI-HRMS m/z: $[M+H]^+$ Calcd for $C_{17}H_{13}NO$ 248.1070; found 248.1067

***N*-(4-((trifluoromethyl)thio)phenyl)naphthalen-1-amine (AR-15)**

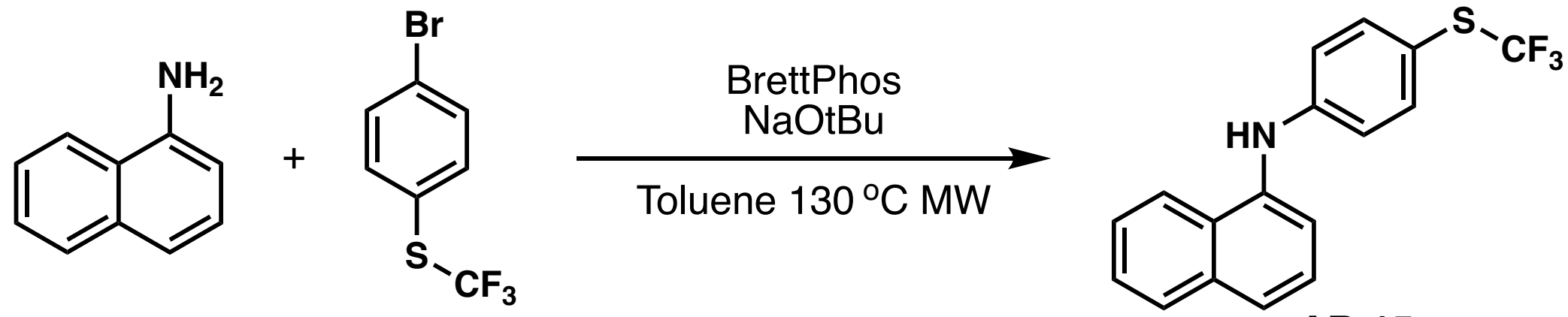

AR-15

General Procedure H: Reaction scale: 200 mg (1.40 mmol) of naphthalen-1-amine. Yields AR-15 as red solid (384 mg, 86%).

^{1}H NMR (850 MHz, DMSO) δ 8.77 (s, 1H), 8.12 – 8.08 (m, 1H), 7.94 – 7.91 (m, 1H), 7.70 (d, *J* = 7.8 Hz, 1H), 7.54 – 7.48 (m, 2H), 7.50 – 7.44 (m, 4H), 7.04 – 7.00 (m, 2H).

^{13}C NMR (214 MHz, DMSO) δ 149.28, 137.90, 137.10 (2C), 134.42, 129.70 (q, *J* = 308.3 Hz), 128.40, 128.22, 126.22, 126.01, 125.65, 124.03, 122.86, 118.81, 115.52 (2C), 108.96.

^{19}F NMR (376 MHz, DMSO) δ -43.74.

ESI-HRMS m/z: $[M+H]^+$ Calcd for $C_{17}H_{12}NF_3S$ 320.0715; found 320.0707

***N*-(naphthalen-1-yl)benzo[*d*][1,3]dioxol-5-amine (AR-16)**

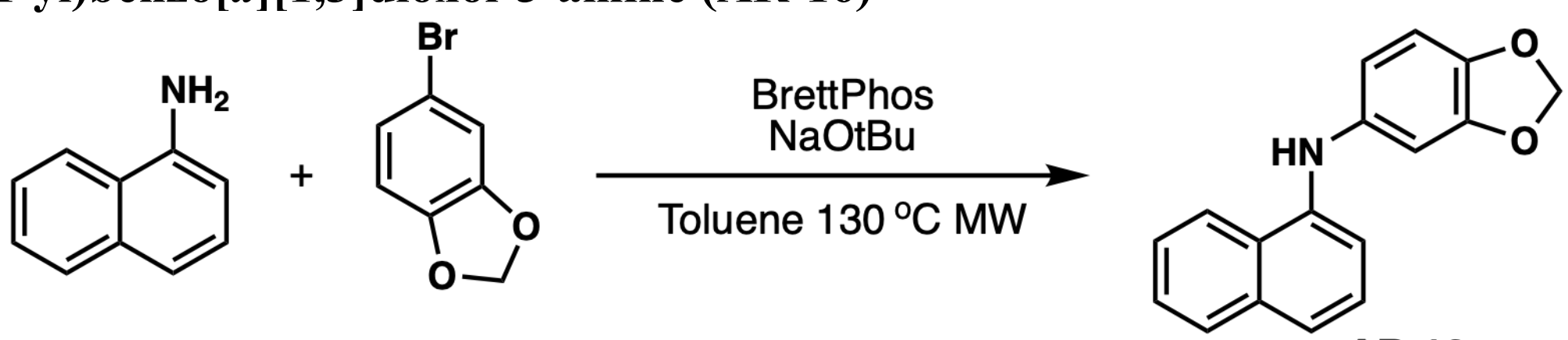

AR-16

General Procedure H: Reaction scale: 200 mg (1.40 mmol) of naphthalen-1-amine. Yields AR-16 as a red oil (345 mg, 94%).

^{1}H NMR (400 MHz, Chloroform-*d*) δ 8.02 – 7.97 (m, 1H), 7.88 – 7.83 (m, 1H), 7.53 – 7.45 (m, 3H), 7.35 (dd, *J* = 7.9, 7.9 Hz, 1H), 7.17 (dd, *J* = 7.5, 1.1 Hz, 1H), 6.76 (d, *J* = 8.2 Hz, 1H), 6.66 (d, *J* = 2.3 Hz, 1H), 6.51 (dd, *J* = 8.2, 2.3 Hz, 1H), 5.94 (s, 2H), 5.84 (s, 1H).

^{13}C NMR (101 MHz, Chloroform-*d*) δ 148.44, 142.74, 140.45, 138.89, 134.77, 128.72, 126.58, 126.26, 126.20, 125.63, 121.77, 121.36, 113.28, 112.41, 108.79, 102.28, 101.20.

ESI-HRMS m/z: $[M+H]^+$ Calcd for $C_{17}H_{13}NO_2$ 264.1019; found 264.1014

***N*-(4-(pyrrolidin-1-yl)phenyl)naphthalen-1-amine (AR-17)**

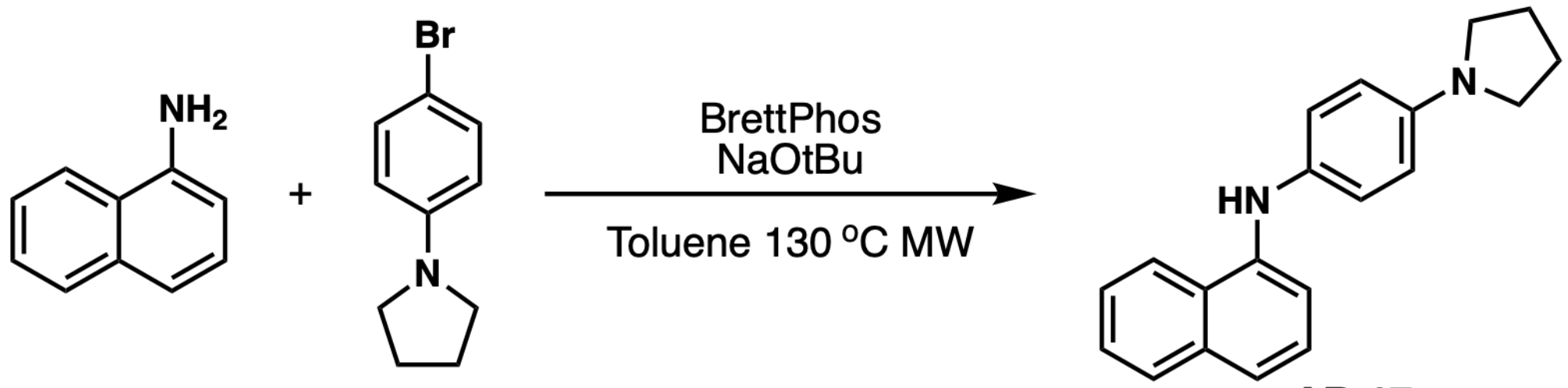

AR-17

General Procedure H: Reaction scale: 200 mg (1.40 mmol) of naphthalen-1-amine. Yields AR-17 as a green solid (300 mg, 75%).

^{1}H NMR (400 MHz, DMSO-d_6) δ 8.30 – 8.25 (m, 1H), 7.82 – 7.76 (m, 2H), 7.49 – 7.41 (m, 2H), 7.22 (d, *J* = 4.5 Hz, 2H), 7.06 (d, *J* = 8.7 Hz, 2H), 6.79 (dd, *J* = 4.4, 4.4 Hz, 1H), 6.57 (d, *J* = 8.8 Hz, 2H), 3.25 – 3.20 (m, 4H), 2.00 – 1.94 (m, 4H).

^{13}C NMR (101 MHz, DMSO-d_6) δ 144.67, 143.61, 134.81, 131.82, 128.41, 126.87, 126.26, 124.75, 124.69, 124.52 (2C), 122.64, 117.85, 112.79 (2C), 107.49, 48.06, 25.42.

ESI-HRMS m/z: $[M+H]^+$ Calcd for $C_{20}H_{20}N_2$ 289.1699; found 289.1692

4-(naphthalen-1-ylamino)benzoic acid (AR-18)

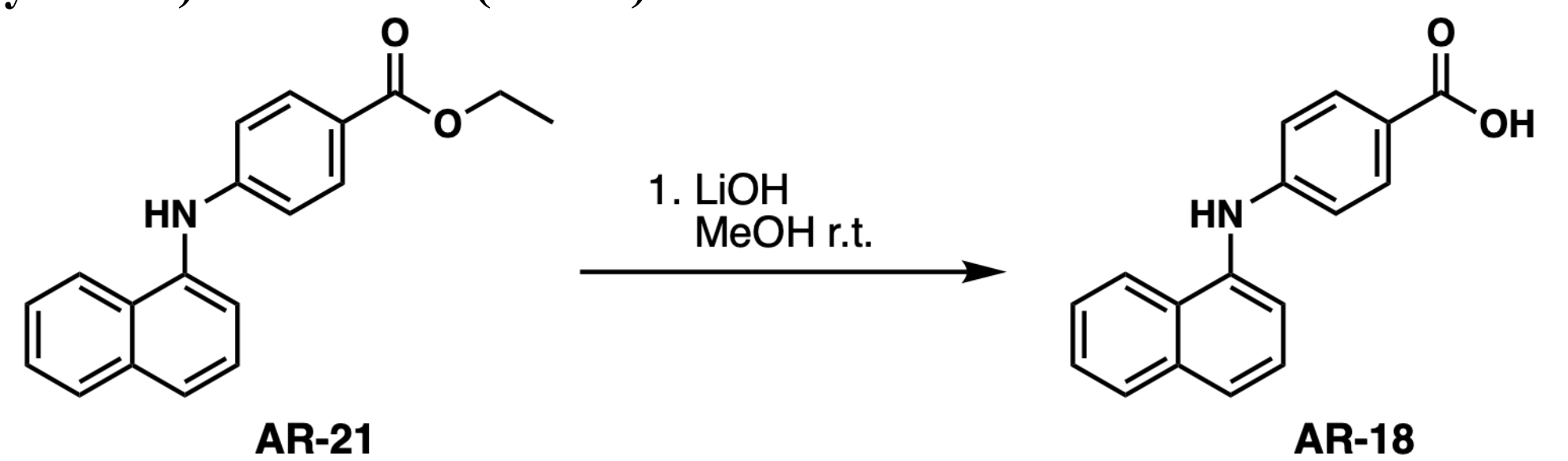

General Procedures B: Reaction scale: 215 mg (0.738 mmol) of AR-21. Yields AR-18 as a red solid (194 mg, 74% yield)

^{1}H NMR (400 MHz, Methanol-d_4) δ 8.03 (d, *J* = 8.2 Hz, 1H), 7.89 (d, *J* = 7.9 Hz, 1H), 7.82 (d, *J* = 8.4 Hz, 2H), 7.70 (d, *J* = 7.4 Hz, 1H), 7.52 – 7.43 (m, 4H), 6.86 (d, *J* = 8.4 Hz, 2H).

^{13}C NMR (101 MHz, Methanol-d_4) δ 152.76, 138.66, 136.35, 132.62(2C), 130.71, 129.40, 127.24, 126.93, 126.82, 125.90(2C), 123.99, 121.18, 114.78.

ESI-HRMS m/z: $[M+H]^+$ Calcd for $C_{17}H_{13}NO_2$ 264.1019; found 264.1018

***N*-(4-(pyridin-4-yl)phenyl)naphthalen-1-amine (AR-19)**

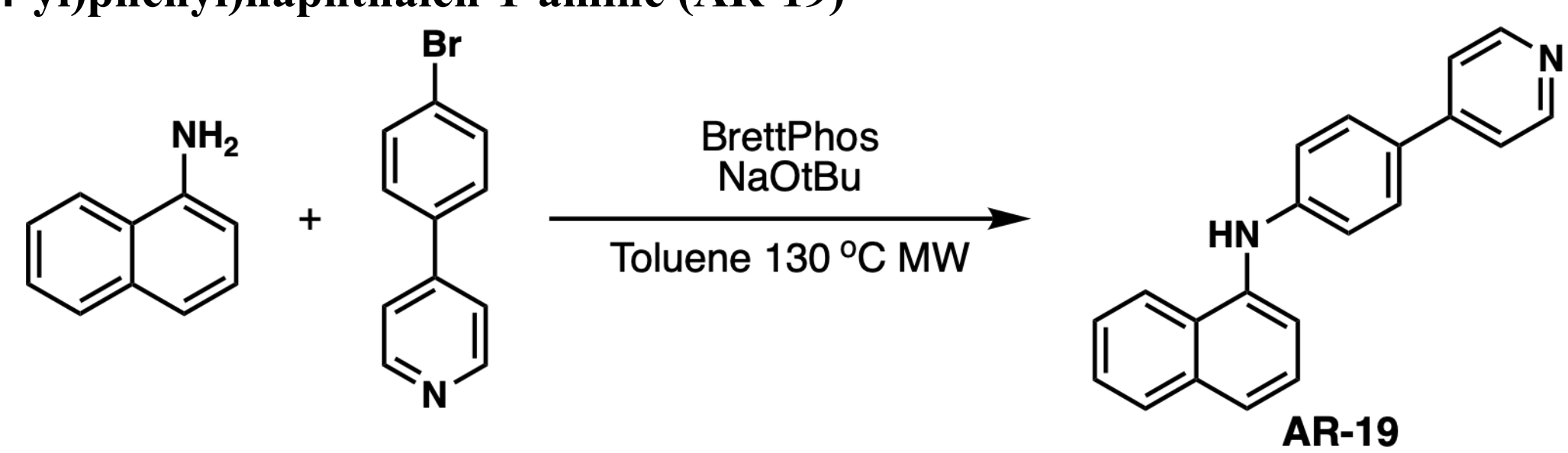

General Procedure H: Reaction scale: 200 mg (1.40 mmol) of naphthalen-1-amine. Yields AR-19 as a tan solid (153 mg, 37%).

^{1}H NMR (400 MHz, Chloroform-*d*) δ 8.64 – 8.55 (m, 2H), 8.06 – 8.00 (m, 1H), 7.95 – 7.86 (m, 1H), 7.74 – 7.64 (m, 1H), 7.56 (d, *J* = 8.8 Hz, 2H), 7.54 – 7.44 (m, 6H), 7.02 (d, *J* = 8.7 Hz, 2H), 6.12 (s, 1H).

^{13}C NMR (101 MHz, Chloroform-*d*) δ 149.86, 148.39, 146.84, 137.57, 134.91, 128.93, 128.77, 128.74, 128.18, 126.47, 126.21, 126.12, 124.64, 122.20, 120.85, 118.61, 116.58.

ESI-HRMS m/z: $[M+H]^+$ Calcd for $C_{21}H_{16}N_2$ 297.1386; found 297.1382

***N*-(naphthalen-1-yl)-6-(pyrrolidin-1-yl)pyridin-3-amine (AR-20)**

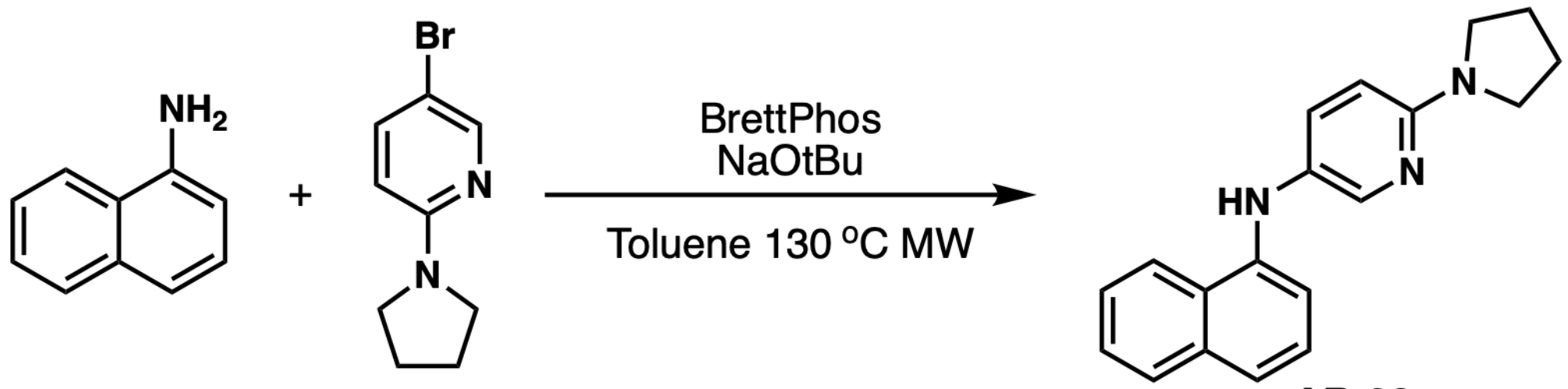

AR-20

General Procedure H: Reaction scale: 200 mg (1.40 mmol) of naphthalen-1-amine. Yields AR-20 as a brown solid(235 mg, 58%).

^{1}H NMR (400 MHz, Chloroform-*d*) δ 8.02 (d, *J* = 2.7 Hz, 1H), 7.93 – 7.89 (m, 1H), 7.76 – 7.71 (m, 1H), 7.42 – 7.37 (m, 2H), 7.30 (dd, *J* = 8.8, 2.7 Hz, 1H), 7.25 (d, *J* = 8.1 Hz, 1H), 7.21 – 7.14 (m, 2H), 6.68 (dd, *J* = 7.4, 1.2 Hz, 1H), 6.32 (d, *J* = 8.8 Hz, 1H), 5.71 (s, 1H), 3.43 – 3.37 (m, 4H), 1.98 – 1.91 (m, 4H).

^{13}C NMR (101 MHz, Chloroform-*d*) δ 155.10, 144.39, 142.75, 134.65, 134.58, 128.79, 127.68, 126.41, 126.09, 125.29, 124.52, 120.50, 119.47, 108.63, 106.89, 47.08 (2C), 25.77 (2C).

ESI-HRMS m/z: $[M+H]^+$ Calcd for $C_{19}H_{19}N_3$ 290.1652; found 290.1647

Ethyl 4-(naphthalen-1-ylamino)benzoate (AR-21)

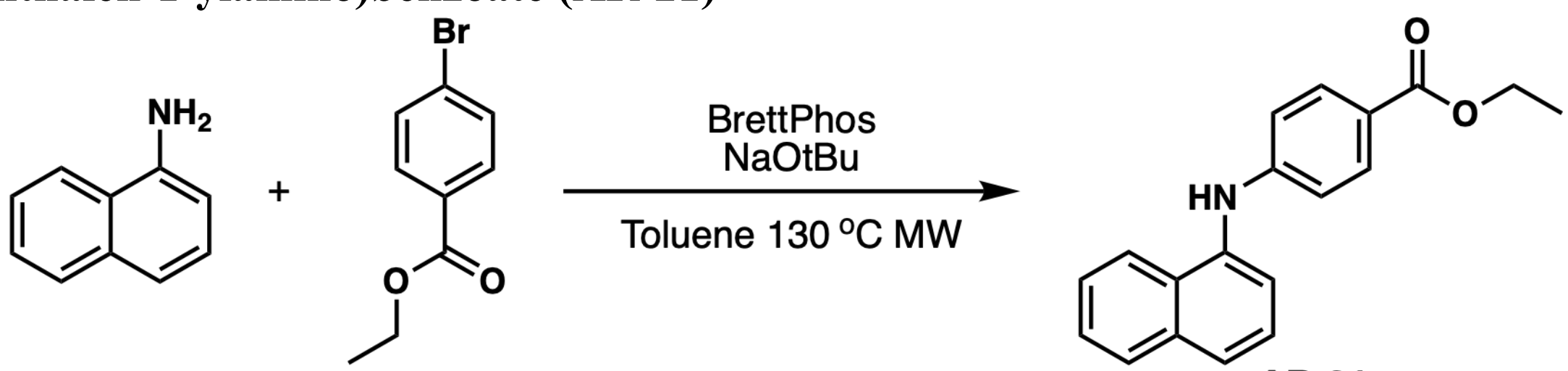

AR-21

General Procedure H: Reaction scale: 200 mg (1.40 mmol) of naphthalen-1-amine. Yields AR-21 as a tan solid (215 mg, 53%).

^{1}H NMR (400 MHz, Chloroform-*d*) δ 7.98 (d, *J* = 8.4 Hz, 1H), 7.91 (d, *J* = 8.6 Hz, 3H), 7.74 – 7.69 (m, 1H), 7.55 – 7.44 (m, 4H), 6.84 (d, *J* = 8.7 Hz, 2H), 6.20 (s, 1H), 4.33 (q, *J* = 7.1 Hz, 2H), 1.37 (t, *J* = 7.1 Hz, 3H).

^{13}C NMR (101 MHz, Chloroform-*d*) δ 166.73, 150.01, 136.66, 134.87, 131.57 (2C), 129.23, 128.72, 126.51, 126.35, 126.04, 125.50, 122.38, 121.13, 120.28, 114.40 (2C), 60.53, 14.57.

ESI-HRMS m/z: $[M+H]^+$ Calcd for $C_{19}H_{17}NO_2$ 292.1332; found 292.1325

5-methoxy-*N*[1]-(naphthalen-1-yl)benzene-1,3-diaminium chloride (AR-22)

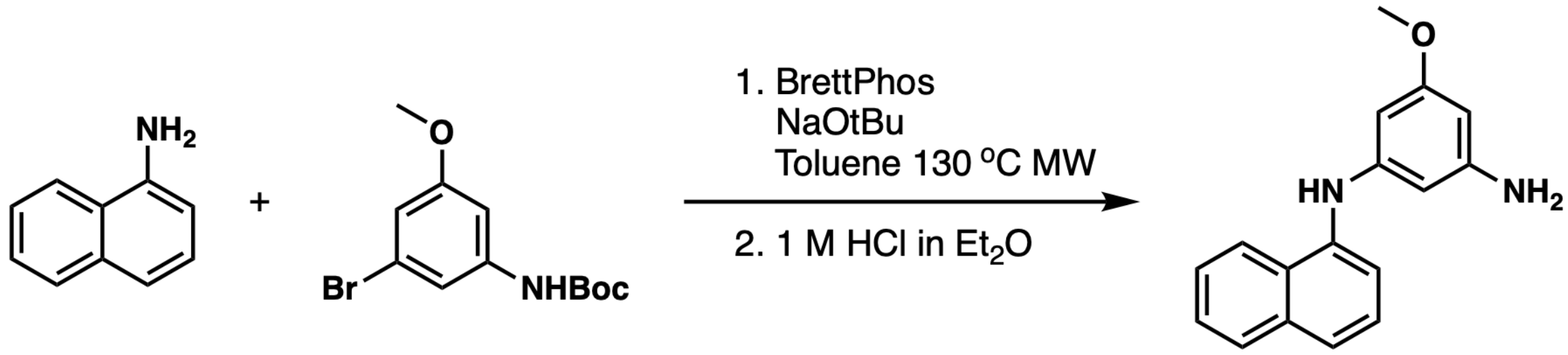

AR-22

General Procedure H Reaction scale: 54 mg (0.38 mmol) of naphthalen-1-amine. After extraction dissolve crude solid in 2 mL of 1 M HCl and let react for 4 hours. Dilute with 10 mL EtOAc and wash with saturated $NaHCO_3$. Purify by flash column chromatography (SiO_2) 0 to 40% EtOAc in Hexanes. Yields AR-22 as white solid (38 mg, 46%).

^{1}H NMR (400 MHz, DMSO-d_6) δ 9.92 (s, 3H), 8.55 (s, 1H), 8.08 (d, J = 7.9 Hz, 1H), 7.93 (d, J = 7.7 Hz, 1H), 7.67 (d, J = 8.1 Hz, 1H), 7.52 (ddd, J = 7.5, 4.9, 2.1 Hz, 2H), 7.47 (t, J = 8.2 Hz, 1H), 7.40 (d, J = 7.4 Hz, 1H), 6.48 (dt, J = 7.2, 2.1 Hz, 2H), 6.29 (t, J = 2.0 Hz, 1H), 3.69 (s, 3H).

^{13}C NMR (101 MHz, DMSO-d_6) δ 160.77, 148.19, 137.78, 134.41, 134.21, 128.22, 128.05, 126.26, 126.08, 125.56, 123.36, 122.87, 117.80, 101.80, 99.55, 98.87, 55.14.

ESI-HRMS m/z: $[M+H]^+$ Calcd for $C_{17}H_{16}N_2O$ 265.1335; found 265.1332

***N*-(4,5-difluoro-2-methoxyphenyl)naphthalen-1-amine (AR-23)**

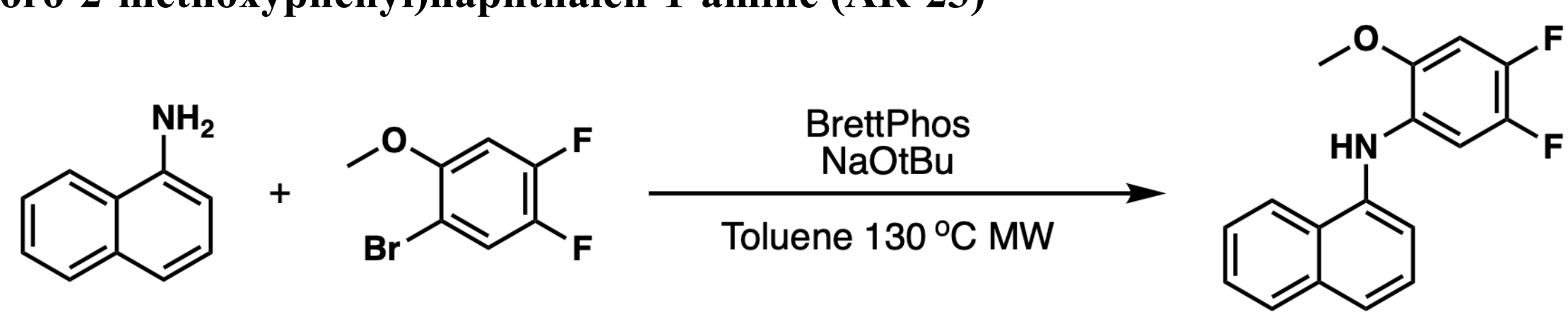

AR-23

General Procedure H: Reaction scale: 150 mg (1.05 mmol) of naphthalen-1-amine. Yields AR-23 as white solid (81 mg, 27%).

^{1}H NMR (850 MHz, DMSO-d_6) δ 8.06 (d, J = 8.3 Hz, 1H), 7.90 (d, J = 8.0 Hz, 1H), 7.58 (d, J = 8.1 Hz, 1H), 7.53 – 7.45 (m, 1H), 7.40 (d, J = 7.8 Hz, 1H), 7.38 (s, 1H), 7.16 (dd, J = 12.4, 7.6 Hz, 1H), 7.08 (d, J = 7.4 Hz, 1H), 6.67 (dd, J = 12.3, 8.3 Hz, 1H), 3.86 (s, 3H).

^{19}F NMR (376 MHz, DMSO-d_6) δ -147.31 (d, J = 23.9 Hz, 1F), -148.40 (d, J = 23.9 Hz, 1F).

ESI-HRMS m/z: $[M+H]^+$ Calcd for $C_{17}H_{13}F_2NO$ 286.1038; found 286.1036

_N_-(2-fluoro-4-methoxyphenyl)naphthalen-1-amine (AR-24)

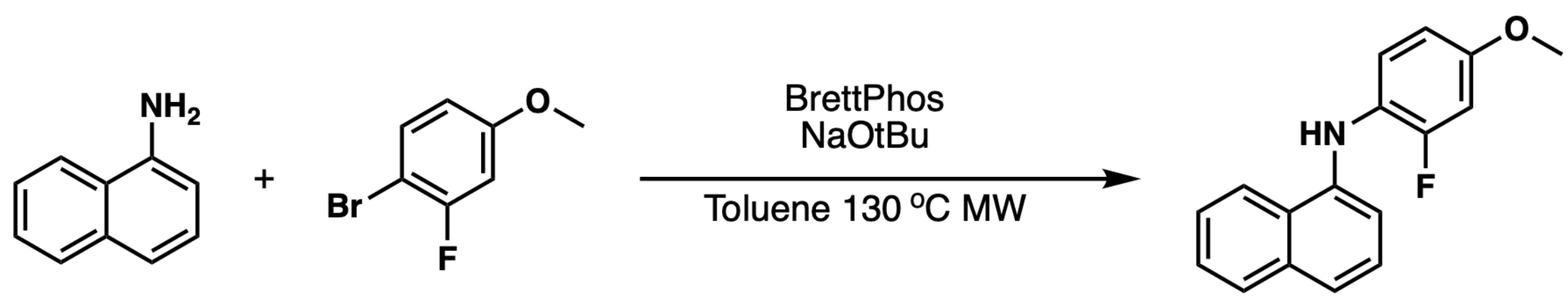

AR-24

General Procedure H: Reaction scale: 100 mg (0.70 mmol) of naphthalen-1-amine. Yields AR-24 as white solid (150 mg, 71%).

^{1}H NMR (400 MHz, DMSO-d_6) δ 8.33 – 8.24 (m, 1H), 7.87 – 7.79 (m, 2H), 7.54 – 7.43 (m, 2H), 7.32 (d, J = 8.1 Hz, 1H), 7.27 (dd, J = 7.7 Hz, 1H), 7.18 (dd, J = 9.2, 9.2 Hz, 1H), 6.96 (dd, J = 12.7, 2.8 Hz, 1H), 6.83 – 6.76 (m, 1H), 6.56 – 6.49 (m, 1H), 3.78 (d, J = 0.7 Hz, 3H).

^{13}C NMR (101 MHz, DMSO-d_6) δ 156.61 (d, J = 245.5 Hz), 156.53 (d, J = 10.1 Hz), 141.95, 134.14, 128.00, 126.64, 126.29, 125.87, 124.64, 124.42, 123.29 (d, J = 12.4 Hz), 122.32, 118.70, 110.43, 108.37, 102.58 (d, J = 17.9 Hz), 55.65 (d, J = 7.4 Hz).

^{19}F NMR (376 MHz, DMSO-d_6) δ -119.51 (s, 1F).

ESI-HRMS m/z: $[M+H]^+$ Calcd for $C_{17}H_{14}FNO$ 268.1132; found 268.1129

4-(_Tert_-butyl)-_N_-(naphthalen-1-yl)pyridin-2-amine (AR-25)

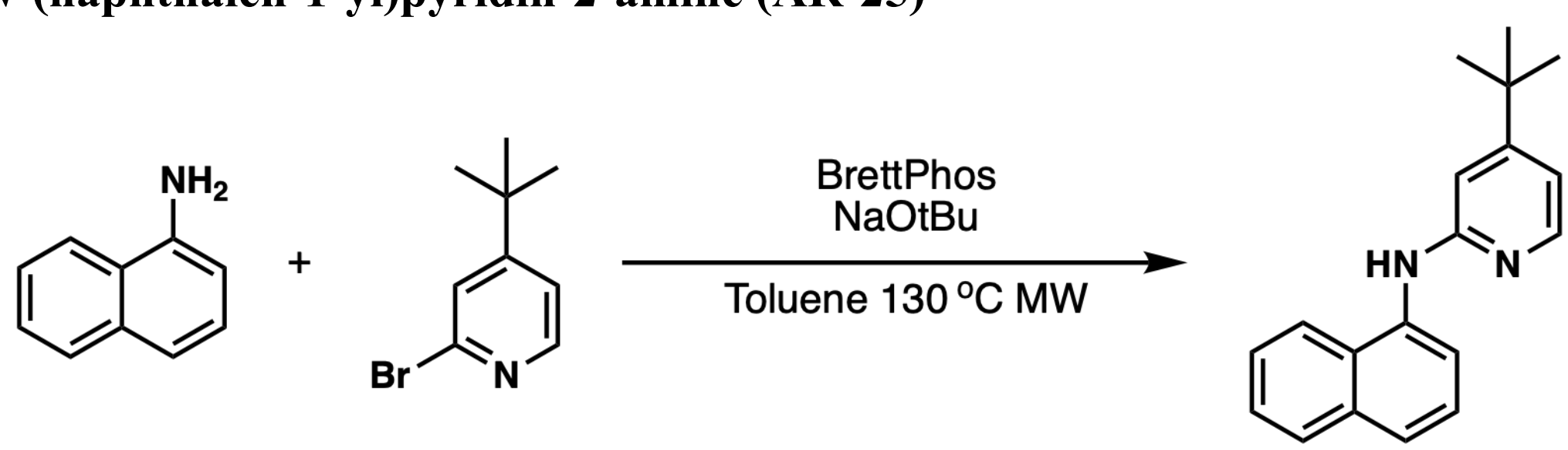

AR-25

General Procedure H: Reaction scale: 150 mg (1.05 mmol) of naphthalen-1-amine. Yields AR-25 as white solid (222 mg, 77%).

^{1}H NMR (400 MHz, Chloroform-d) δ 8.14 – 8.04 (m, 2H), 7.92 – 7.86 (m, 1H), 7.67 (d, J = 1.1 Hz, 1H), 7.61 (dd, J = 7.5, 1.2 Hz, 1H), 7.56 – 7.43 (m, 3H), 7.07 (s, 1H), 6.75 (dq, J = 3.2, 1.7 Hz, 2H), 1.20 (s, 9H).

^{13}C NMR (101 MHz, Chloroform-d) δ 162.02, 157.64, 148.29 (d, J = 2.7 Hz), 136.62, 134.82, 128.80, 128.62, 126.35, 126.07, 126.02, 124.62, 122.34, 119.05, 112.86 (d, J = 4.4 Hz), 105.02 (d, J = 5.6 Hz), 34.85, 30.53 (3C, d, J = 4.1 Hz).

ESI-HRMS m/z: $[M+H]^+$ Calcd for $C_{19}H_{20}N_2$ 277.1699; found 277.1694

N-(3-(2-carboxyethyl)phenyl)naphthalen-1-aminium (AR-26)

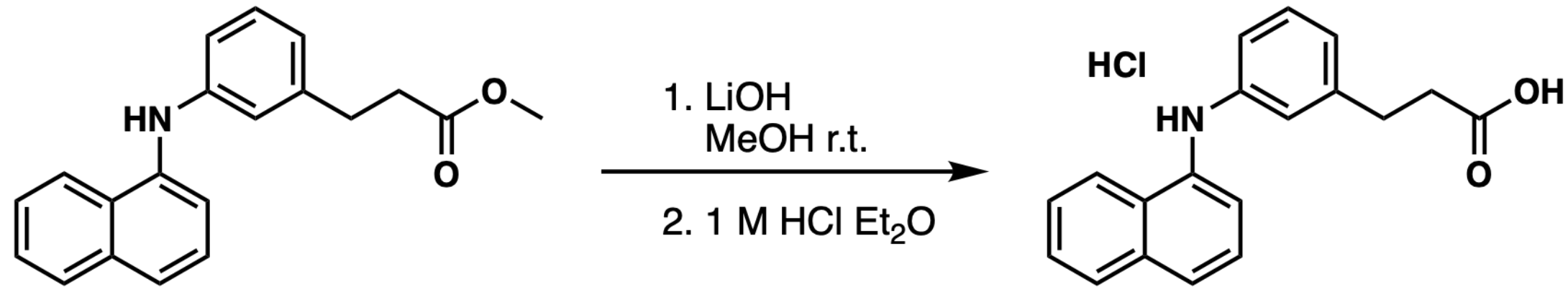

AR-29 AR-26

Procedure: Dissolve AR-29 (.100 g, 1 Eq, 327 μmol) in MeOH (3.27 mL) then slowly add LiOH (78.4 mg, 10 Eq, 3.27 mmol). Let react for 18 hours before concentrating in vacuo. Dissolve in a minimal amount of DCM and precipitate out the product as the hydrochloride salt with excess 1M HCl in Et_2O. Triturate off solvent and dry over vacuum to yield AR-26 (.089 g, 0.31 mmol, 93 %) as a blue solid.

^{1}H NMR (400 MHz, DMSO-d_6) δ 8.20 – 8.13 (m, 1H), 7.88 (dd, J = 8.0, 1.6 Hz, 1H), 7.54 – 7.45 (m, 3H), 7.39 (t, J = 7.8 Hz, 1H), 7.31 (dd, J = 7.5, 1.2 Hz, 1H), 7.12 (dd, J = 7.8, 7.8 Hz, 1H), 6.95 (t, J = 2.0 Hz, 1H), 6.87 (dd, J = 7.7, 1.9 Hz, 1H), 6.69 (d, J = 7.5 Hz, 1H), 2.75 (t, J = 7.6 Hz, 2H), 2.55 – 2.46 (m, 2H).

^{13}C NMR (101 MHz, DMSO-d_6) δ 173.77, 144.88, 141.74, 139.41, 134.41, 129.01, 128.09, 126.85, 126.18, 126.04, 124.98, 122.78, 121.18, 119.55, 117.05, 114.88, 113.79, 35.23, 30.52.

ESI-HRMS m/z: $[M+H]^+$ Calcd for $C_{19}H_{17}NO_2$ 292.1332; found 292.1329

Methyl 2-methyl-5-(naphthalen-1-ylamino)benzoate (AR-27)

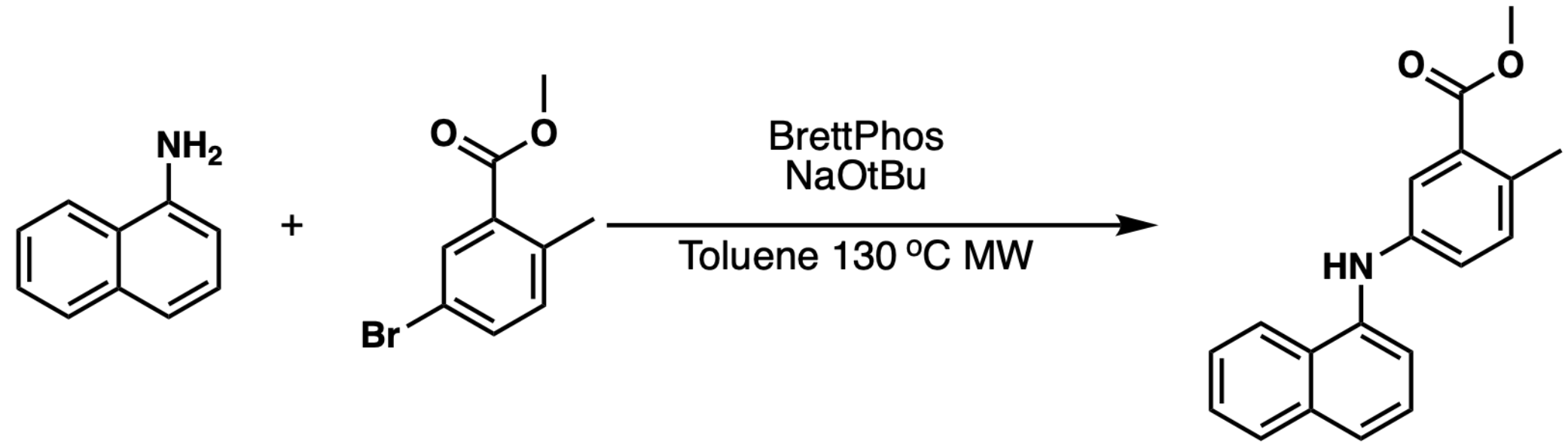

AR-27

General Procedure H: Reaction scale: 50 mg (0.35 mmol) of naphthalen-1-amine. Yields AR-27 as white solid (80 mg, 79%).

^{1}H NMR (400 MHz, DMSO-d_6) δ 8.18 – 8.11 (m, 1H), 7.89 (dd, J = 7.4, 2.0 Hz, 1H), 7.60 – 7.44 (m, 4H), 7.40 (t, J = 7.8 Hz, 1H), 7.27 (dd, J = 7.5, 1.1 Hz, 1H), 7.21 – 7.10 (m, 2H), 3.78 (s, 3H), 2.42 (s, 3H).

^{13}C NMR (101 MHz, DMSO-d_6) δ 167.87, 143.53, 139.65, 134.89, 132.80, 130.25, 130.09, 128.61, 127.42, 126.63, 126.61, 125.65, 123.16, 122.13, 121.16, 118.97, 114.55, 52.26, 20.83.

ESI-HRMS m/z: $[M+H]^+$ Calcd for $C_{19}H_{17}NO_2$ 292.1332; found 292.1329

6-chloro-4-methyl-*N*-(naphthalen-1-yl)pyridin-3-amine (AR-28)

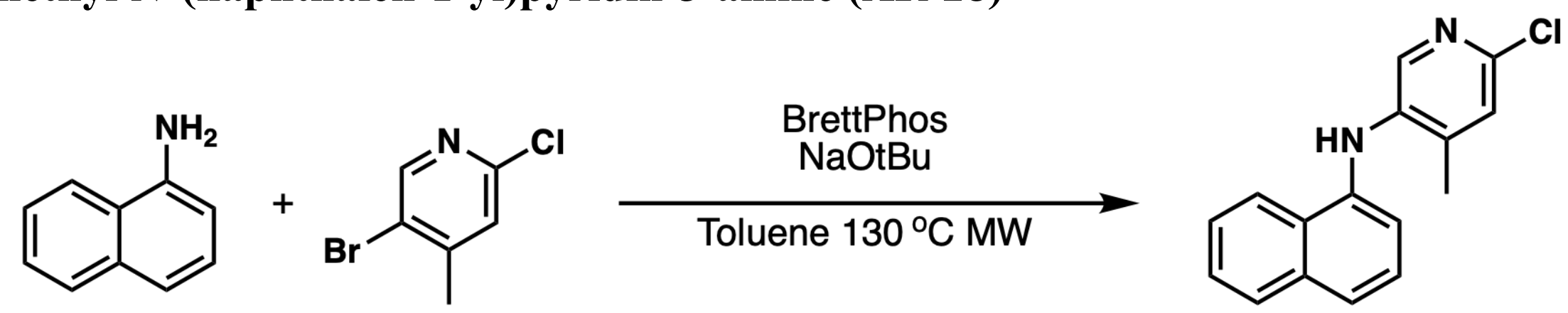

AR-28

General Procedure H: Reaction scale: 250 mg (1.75 mmol) of naphthalen-1-amine. Yields AR-28 as an off white solid (221 mg, 47%).

^{1}H NMR (400 MHz, DMSO-d_6) δ 8.12 (d, J = 7.9 Hz, 1H), 7.90 (d, J = 7.7 Hz, 1H), 7.76 (s, 1H), 7.57 – 7.45 (m, 3H), 7.39 (s, 1H), 7.36 (t, J = 7.8 Hz, 1H), 6.77 (d, J = 7.4 Hz, 1H), 2.22 (s, 3H).

^{13}C NMR (101 MHz, DMSO-d_6) δ 142.76, 142.70, 141.23, 140.19, 140.12, 134.75, 128.68, 126.85, 126.69, 126.64, 125.83, 125.77, 123.25, 122.30, 114.73, 17.86.

ESI-HRMS m/z: $[M+H]^+$ Calcd for $C_{16}H_{13}ClN_2$ 269.0840; found 269.0836

***N*-(3-(3-methoxy-3-oxopropyl)phenyl)naphthalen-1-aminium chloride (AR-29)**

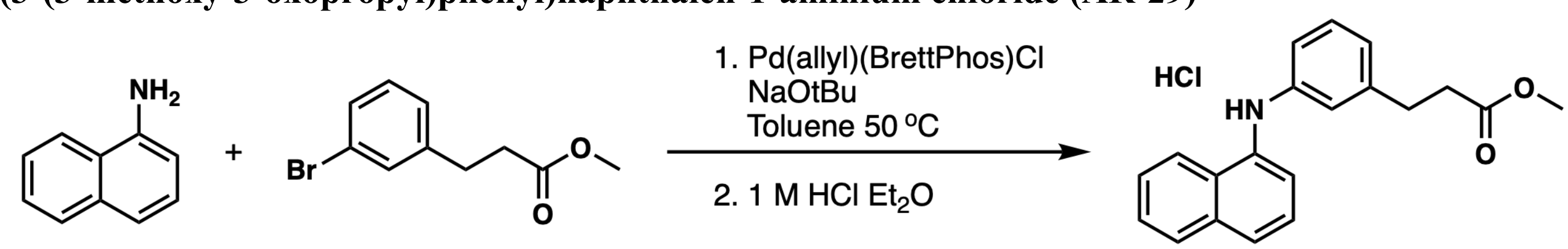

AR-29

General Procedure I: Reaction scale: 125 mg (0.87 mmol) of naphthalen-1-amine. Yields AR-29 as white solid (202 mg, 76%).

^{1}H NMR (400 MHz, Chloroform-d) δ 8.25 – 8.16 (m, 1H), 7.92 – 7.86 (m, 2H), 7.81 (d, J = 8.3 Hz, 1H), 7.57 – 7.49 (m, 2H), 7.45 (dd, J = 7.8, 7.8 Hz, 1H), 7.32 – 7.25 (m, 2H), 7.21 (dd, J = 9.0, 7.3 Hz, 1H), 7.03 (d, J = 7.5 Hz, 1H), 3.63 (s, 3H), 2.85 (t, J = 7.8 Hz, 2H), 2.54 (t, J = 7.8 Hz, 2H).

^{13}C NMR (101 MHz, Chloroform-d) δ 172.93, 142.44, 139.86, 134.66, 133.52, 129.76, 128.56, 127.87, 127.14, 126.99, 126.80, 126.13, 125.34, 122.27, 120.98, 120.70, 118.94, 51.62, 35.21, 30.62.

ESI-HRMS m/z: $[M+H]^+$ Calcd for $C_{20}H_{19}NO_2$ 306.1489; found 306.1484

Methyl 3-methoxy-4-(naphthalen-1-ylamino)benzoate (AR-30)

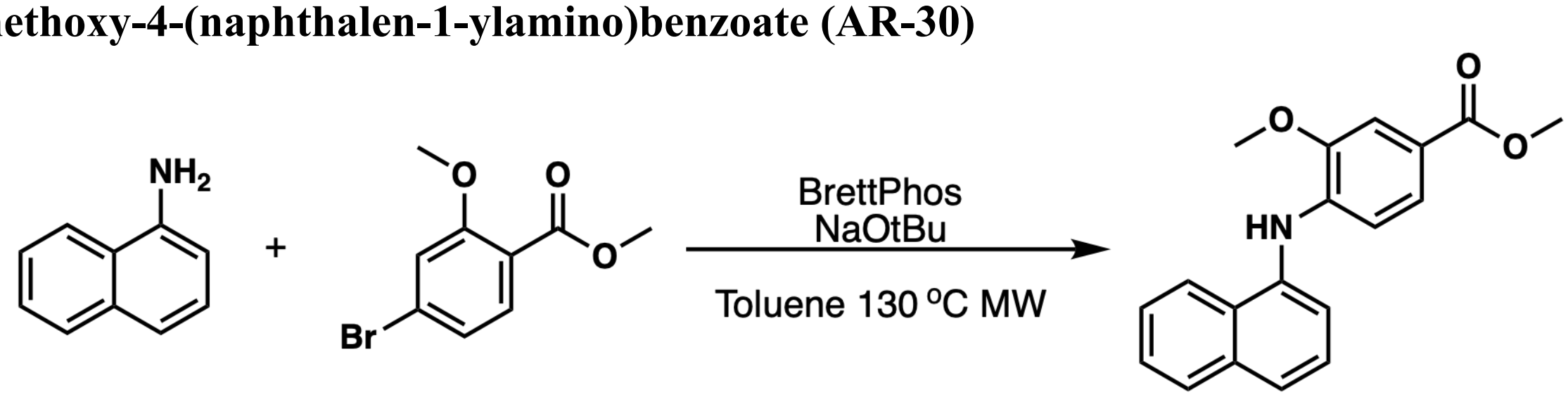

AR-30

General Procedure H: Reaction scale: 200 mg (1.40 mmol) of naphthalen-1-amine. Yields AR-30 as brown oil (119 mg, 28%).

^{1}H NMR (400 MHz, Chloroform-*d*) δ 8.03 – 7.99 (m, 1H), 7.92 – 7.88 (m, 1H), 7.70 (d, *J* = 7.8 Hz, 1H), 7.59 (d, *J* = 1.8 Hz, 1H), 7.55 – 7.46 (m, 5H), 6.82 (d, *J* = 8.4 Hz, 1H), 4.04 (s, 3H), 3.88 (s, 3H).

^{13}C NMR (101 MHz, Chloroform-*d*) δ 167.38, 146.47, 140.07, 136.47, 134.85, 129.29, 128.66, 126.45, 126.26, 125.99, 125.18, 124.09, 122.39, 120.09, 119.74, 111.40, 110.96, 56.06, 51.93.

ESI-HRMS m/z: $[M+H]^+$ Calcd for $C_{19}H_{17}NO_3$ 308.1281; found 308.1275

_N_-(naphthalen-1-yl)isoquinolin-7-amine (AR-31)

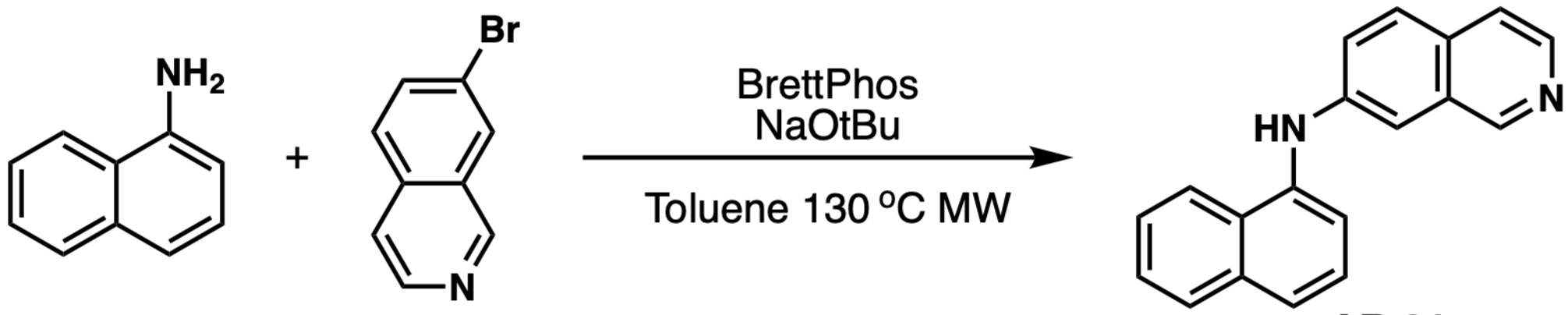

AR-31

General Procedure H: Reaction scale: 200 mg (1.40 mmol) of naphthalen-1-amine. Yields AR-31 as tan solid (113 mg, 30%).

^{1}H NMR (400 MHz, Chloroform-*d*) δ 8.97 (d, *J* = 1.0 Hz, 1H), 8.34 (d, *J* = 5.6 Hz, 1H), 8.06 – 8.02 (m, 1H), 7.94 – 7.90 (m, 1H), 7.75 – 7.69 (m, 2H), 7.56 – 7.41 (m, 6H), 7.21 (d, *J* = 2.4 Hz, 1H), 6.19 (s, 1H).

^{13}C NMR (101 MHz, $CDCl_3$) δ 151.03, 144.53, 140.79, 137.69, 134.96, 131.02, 130.30, 128.81, 128.72, 127.94, 126.54, 126.29, 126.17, 124.85, 123.57, 122.20, 120.34, 118.63, 108.98.

ESI-HRMS m/z: $[M+H]^+$ Calcd for $C_{19}H_{14}N_2$ 271.1230; found 271.1224

_N_-(naphthalen-1-yl)pyridin-3-amine (AR-32)

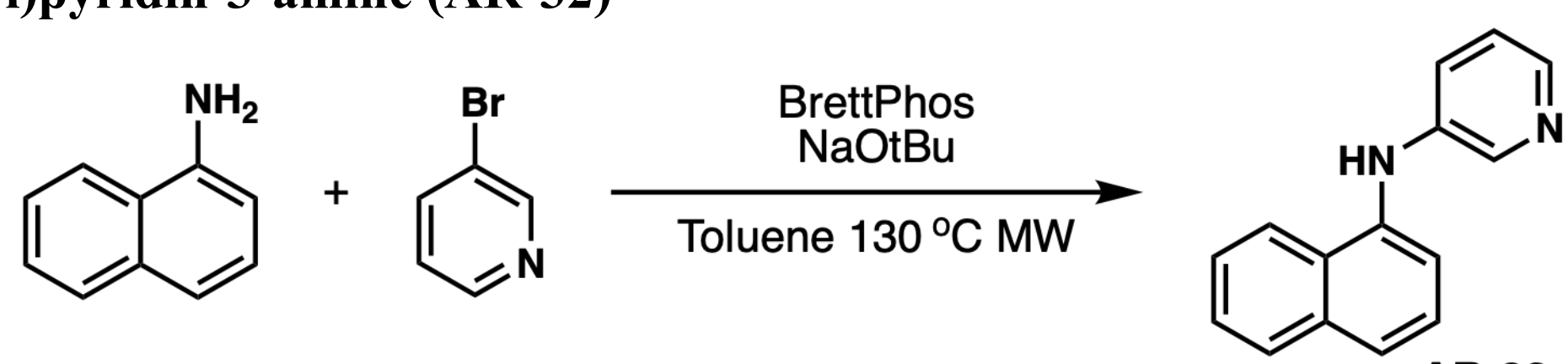

AR-32

General Procedure H: Reaction scale: 200 mg (1.40 mmol) of naphthalen-1-amine. Yields AR-32 as tan solid (175 mg, 57%).

^{1}H NMR (400 MHz, Chloroform-*d*) δ 8.36 (d, *J* = 2.7 Hz, 1H), 8.15 (dd, *J* = 4.6, 1.5 Hz, 1H), 8.02 – 7.97 (m, 1H), 7.92 – 7.87 (m, 1H), 7.65 (d, *J* = 8.1 Hz, 1H), 7.56 – 7.48 (m, 2H), 7.43 (t, *J* = 7.8 Hz, 1H), 7.36 (d, *J* = 6.9 Hz, 1H), 7.20 (ddd, *J* = 8.3, 2.9, 1.5 Hz, 1H), 7.13 (dd, *J* = 8.2, 4.6 Hz, 1H), 5.94 (s, 1H).

^{13}C NMR (101 MHz, Chloroform-*d*) δ 141.69, 141.61, 139.67, 137.61, 134.88, 128.80, 128.27, 126.51, 126.24, 126.08, 124.42, 123.86, 122.99, 121.92, 117.31.

ESI-HRMS m/z: $[M+H]^+$ Calcd for $C_{15}H_{12}N_2$ 221.1073; found 221.1071

3-(naphthalen-1-ylamino)benzoic acid (AR-33)

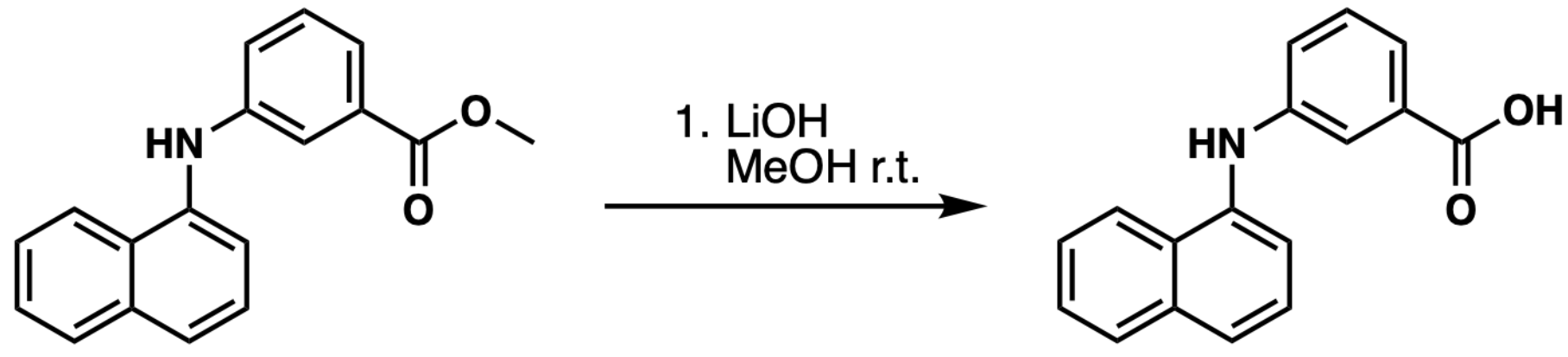

AR-12 **AR-33**

General Procedures B: Reaction scale: 168 mg (0.606 mmol) of AR-21. Yields AR-33 as brown solid (90 mg, 54% yield)

^{1}H NMR (400 MHz, Methanol-d_4) δ 8.09 (d, J = 8.1 Hz, 1H), 7.86 (d, J = 7.9 Hz, 1H), 7.62 (s, 1H), 7.59 (d, J = 8.0 Hz, 1H), 7.52 – 7.32 (m, 5H), 7.27 (dd, J = 7.9, 7.9 Hz, 1H), 7.16 (d, J = 8.3 Hz, 1H).

^{13}C NMR (101 MHz, MeOD) δ 170.25, 147.77, 140.22, 136.37, 132.70, 130.11, 129.78, 129.34, 127.08, 126.99, 126.45, 124.26, 123.78, 121.82, 121.51, 118.34, 117.85.

ESI-HRMS m/z: $[M+H]^+$ Calcd for $C_{17}H_{13}NO_2$ 264.1019; found 264.1018

(4-(naphthalen-1-ylamino)phenyl)methanol (AR-34)

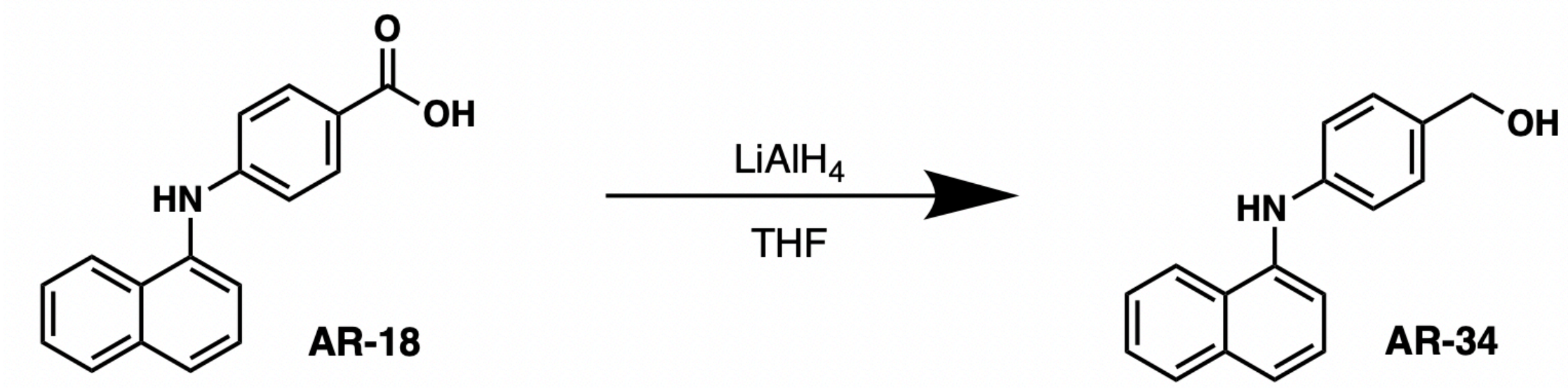

General Procedure: Reaction scale: 20 mg (.076 mmol) of AR-18 was dissolved in 0.5 mL THF. Added 23 mg (8 eq., 605μL) of $LiAlH_4$. Allowed to stir for 24 hours at RT. Added 2 mL saturated sodium potassium tartrate solution. Extracted into ethyl acetate (3x15 mL). Washed with water 3 times, brine 1 time. Dried over sodium sulfate, filtered, and concentrated in vacuo. Purified using flash chromatography (0-10% MeOH in DCM). Yields AR-34 as a tan solid (10 mg, 53% yield).

^{1}H NMR (400 MHz, Methanol-d_4) δ 8.11 (d, J = 7.9 Hz, 1H), 7.81 (d, J = 8.1 Hz, 1H), 7.51 – 7.39 (m, 3H), 7.32 (dd, J = 8.1, 8.1 Hz, 1H), 7.22 (d, J = 7.3 Hz, 1H), 7.04 (d, J = 8.1 Hz, 2H), 6.94 (d, J = 8.3 Hz, 2H), 2.27 (s, 2H).

^{13}C NMR (101 MHz, Methanol-d_4) δ 143.96, 141.88, 136.29, 130.70, 130.57, 129.23, 128.60, 127.01, 126.85, 125.96, 123.50, 122.28, 119.49, 114.52, 54.89.

ESI-HRMS m/z: $[M+H]^+$ Calcd for $C_{17}H_{15}NO$ 250.1226; found 250.1224

(3-(naphthalen-1-ylamino)phenyl)methanol (AR-35)

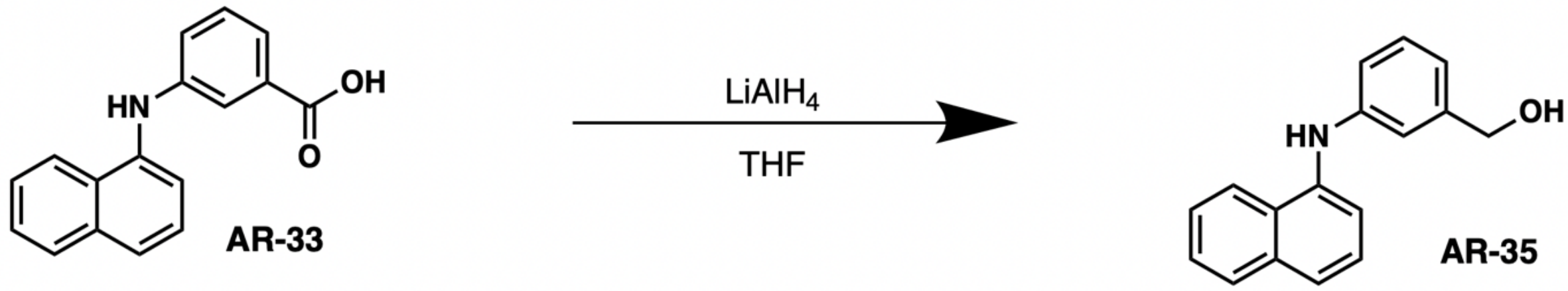

General Procedure: Reaction scale: 35 mg (.113 mmol) of AR-33 was dissolved in 0.5 mL THF. Added 27 mg (8 eq., 700µL) of $LiAlH_4$. Allowed to stir for 24 hours at RT. Added 2 mL saturated sodium potassium tartrate solution. Extracted into ethyl acetate (3x15 mL). Washed with water 3 times, brine 1 time. Dried over sodium sulfate, filtered, and concentrated in vacuo. Purified using flash chromatography (0-10% MeOH in DCM). Yields AR-35 as a tan solid (10 mg, 48% yield).

^{1}H NMR (400 MHz, Methanol-d_4) δ 8.13 – 8.08 (m, 1H), 7.86 – 7.81 (m, 1H), 7.51 (d, J = 7.8 Hz, 1H), 7.49 – 7.39 (m, 2H), 7.39 – 7.31 (m, 2H), 7.17 (t, J = 7.8 Hz, 1H), 7.02 (d, J = 2.0 Hz, 1H), 6.90 (dd, J = 7.9, 2.4 Hz, 1H), 6.82 (d, J = 7.5 Hz, 1H), 4.52 (s, 2H).

^{13}C NMR (101 MHz, MeOD) δ 147.21, 143.66, 140.99, 136.33, 130.09, 129.39, 129.26, 127.00, 126.94, 126.18, 123.71, 123.34, 119.40, 117.18, 116.73, 116.67, 65.40.

ESI-HRMS m/z: $[M+H]^+$ Calcd for $C_{17}H_{15}NO$ 250.1226; found 250.1224

***N*-(4-((3,5-dichlorophenyl)ethynyl)phenyl)naphthalen-1-amine (AR-36)**

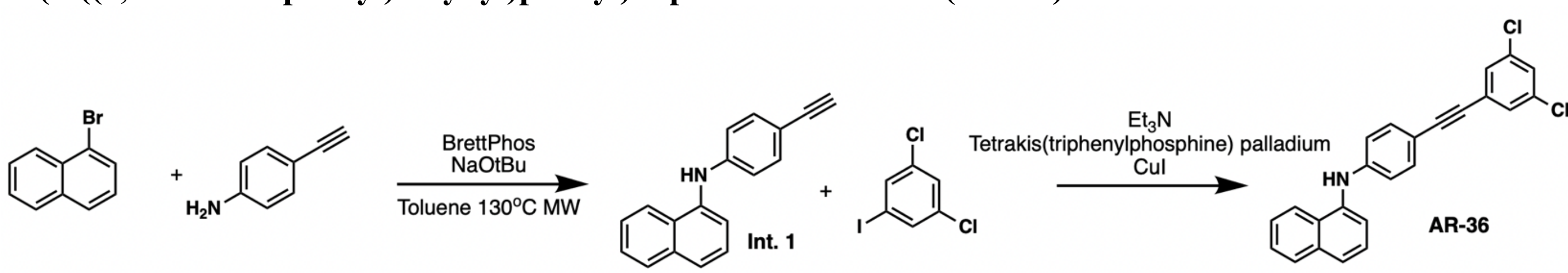

Step #1: General Procedure H: Reaction scale: 200 mg (0.971 mmol) of 1-bromonaphthalene. Yields Int. 1 as a white solide (38 mg, 16% yield). **Step #2: General Procedure:** Reaction scale: 38 mg (0.156 mmol) of Int. 1 was dissolved in 2 mL of Et_3N. Dichloro-iodobenzene (1eq, 33mg), tetrakis(triphenylphosphine) palladium (20 mol%, 3.6 mg) and copper iodide (10 mol%, 3 mg) were added to the reaction vessel. Degassed solution by bubbling nitrogen through for 30 minutes. Heated to 50 °C and allowed to stir overnight for ~18 hours. Filtered reaction through celite and washed with DCM. Purified with flash chromatography (20% EtOAc in Hexanes). Yields AR-36 as a brown solid (50 mg, 83% yield).

^{1}H NMR (400 MHz, Methanol-d_4) δ 8.09 – 8.02 (m, 1H), 7.88 (dd, J = 7.6, 1.8 Hz, 1H), 7.64 (d, J = 7.5 Hz, 1H), 7.53 – 7.36 (m, 6H), 7.38 – 7.31 (m, 2H), 6.93 – 6.85 (m, 2H).

^{13}C NMR (214 MHz, Acetone) δ 148.23, 138.58, 135.89, 135.65, 133.94, 130.14, 129.71, 129.67, 129.28, 128.51, 128.12, 127.10, 126.89, 126.55, 124.98, 124.95, 123.58, 119.42, 119.31, 116.25, 116.23, 112.40, 94.19, 85.60.

ESI-HRMS m/z: $[M+H]^+$ Calcd for $C_{24}H_{15}Cl_2N$ 388.0654; found 388.0649

N-(4-(1,3,5-trimethyl-1H-pyrazol-4-yl)phenyl)naphthalen-1-amine (AR-37)

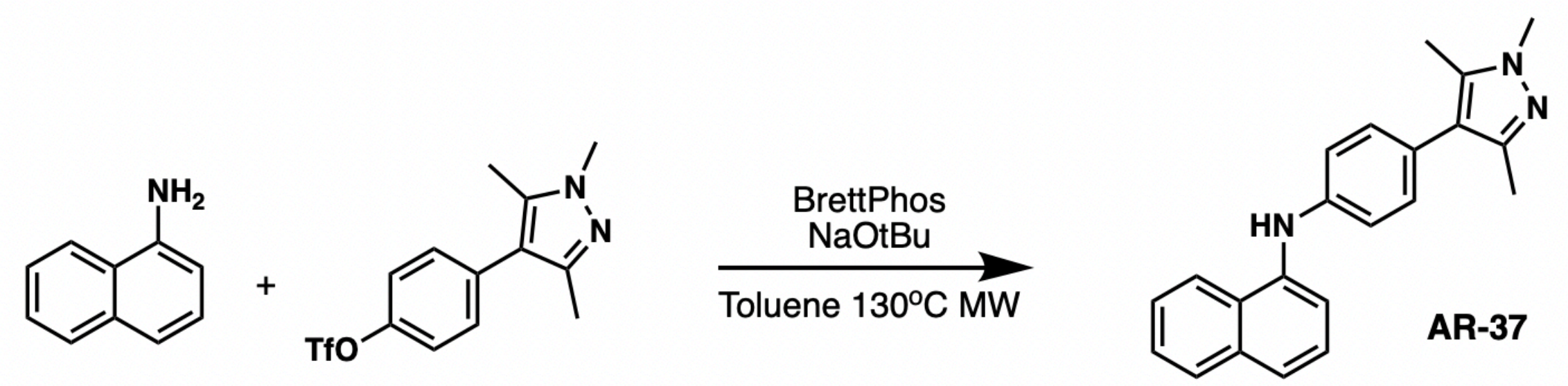

General Procedure H: Reaction scale: 56 mg (0.391 mmol) of naphthalen-1-amine. Yields AR-37 as white solid (50 mg, 39% yield).

^{1}H NMR (400 MHz, Methanol-d_4) δ 8.13 (d, J = 7.9 Hz, 1H), 7.83 (d, J = 7.7 Hz, 1H), 7.56 – 7.34 (m, 5H), 7.11 – 7.01 (m, 4H), 3.74 (s, 3H), 2.23 (s, 4H), 2.18 (s, 3H).

^{13}C NMR (101 MHz, MeOD) δ 145.76, 140.89, 138.58, 136.34, 131.31, 129.37, 129.29, 126.99, 126.24, 125.76, 123.70, 123.40, 120.69, 118.14, 116.62, 35.89, 35.80, 12.18, 12.13, 10.13, 10.07.

ESI-HRMS m/z: $[M+H]^+$ Calcd for $C_{22}H_{21}N_3$ 328.1808; found 328.1800

2-(naphthalen-1-ylammonio)-6-(trifluoromethyl)pyridin-1-ium chloride (AR-38)

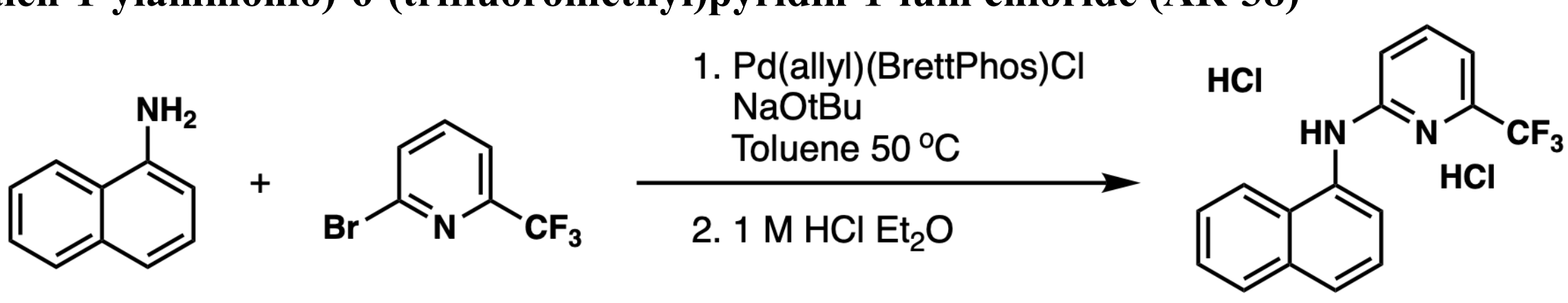

General Procedure I: Reaction scale: 125 mg (0.87 mmol) of naphthalen-1-amine. Yields AR-38 as white solid (202 mg, 76%).

^{1}H NMR (400 MHz, DMSO-d_6) δ 9.86 (s, 2H), 9.44 (s, 1H), 8.19 – 8.09 (m, 1H), 7.98 – 7.91 (m, 1H), 7.84 (dd, J = 7.6, 1.1 Hz, 1H), 7.78 – 7.69 (m, 2H), 7.58 – 7.46 (m, 3H), 7.15 (d, J = 7.3 Hz, 1H), 7.05 (d, J = 8.5 Hz, 1H).

^{13}C NMR (101 MHz, DMSO-d_6) δ 157.50, 144.54 (d, J = 33.2 Hz), 138.88, 135.50, 134.11, 128.25, 127.89, 126.08, 125.90, 125.68, 124.16, 122.78, 121.72 (d, J = 273.9 Hz), 119.55, 113.30, 110.26.

ESI-HRMS m/z: $[M+H]^+$ Calcd for $C_{16}H_{11}F_3N_2$ 289.0947; found 289.0945

N-(4-(difluoromethoxy)phenyl)naphthalen-1-aminium chloride (AR-39)

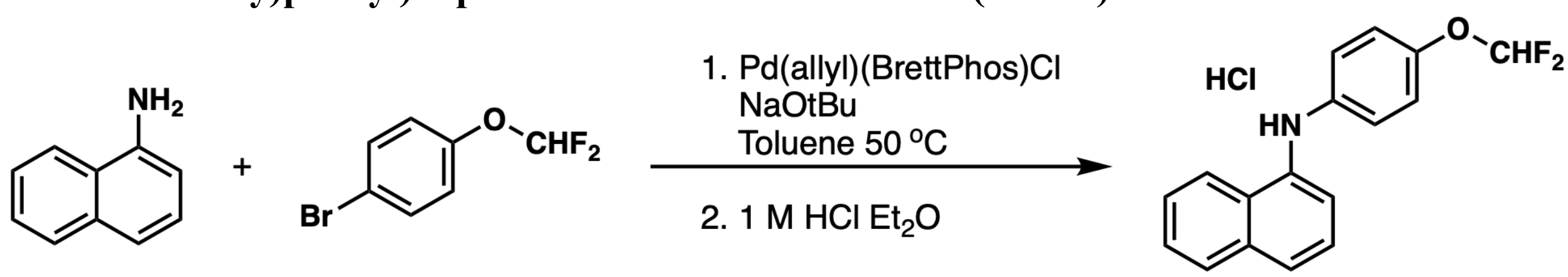

General Procedure I: Reaction scale: 100 mg (0.70 mmol) of naphthalen-1-amine. Yields AR-39 as white solid (132 mg, 59%).

^{1}H NMR (400 MHz, DMSO-d_6) δ 9.81 (s, 2H), 8.18 (d, J = 7.8 Hz, 1H), 7.87 (d, J = 7.5 Hz, 1H), 7.55 – 7.43 (m, 3H), 7.38 (t, J = 7.8 Hz, 1H), 7.31 – 6.84 (m, 6H).

^{13}C NMR (101 MHz, DMSO-d_6) δ 143.69, 142.76, 139.53, 134.49, 128.19, 126.95, 126.23, 126.16, 125.18, 122.83, 121.57, 120.53, 118.22, 115.52 (d, J = 256.9 Hz), 113.96.

^{19}F NMR (376 MHz, DMSO-d_6) δ -80.87 (d, J = 74.9 Hz, 1F).

ESI-HRMS m/z: $[M+H]^+$ Calcd for $C_{17}H_{13}F_2NO$ 286.1038; found 286.1050

***N*-(2,6-dimethoxyphenyl)naphthalen-1-aminium chloride (AR-40)**

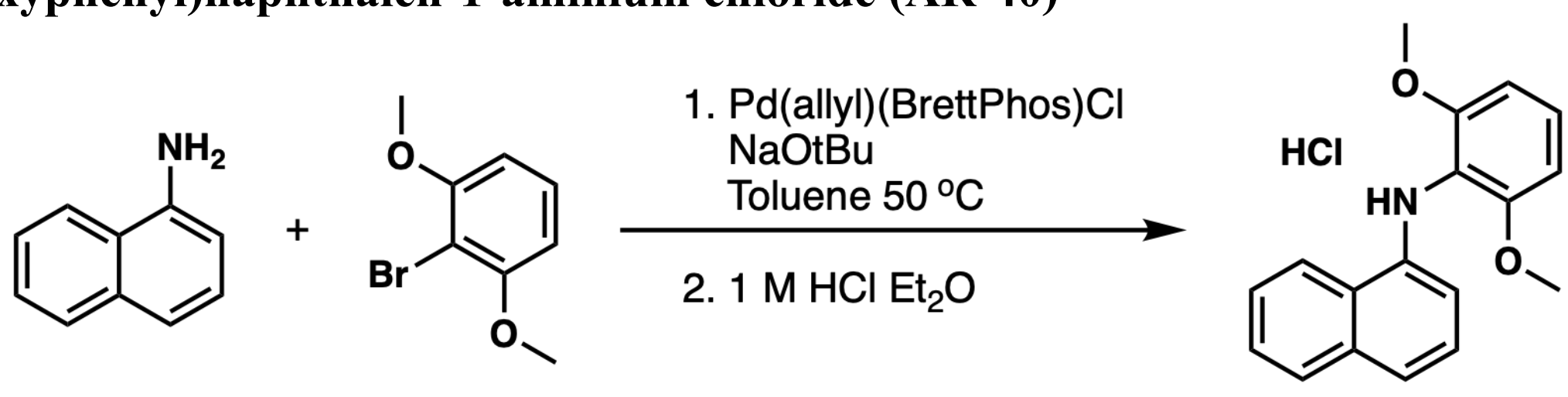

AR-40

General Procedure I: Reaction scale: 100 mg (0.70 mmol) of naphthalen-1-amine. Yields AR-40 as white solid (143 mg, 65%).

^{1}H NMR (400 MHz, DMSO-d_6) δ 10.13 (s, 2H), 8.42 – 8.33 (m, 1H), 7.84 – 7.74 (m, 1H), 7.50 – 7.40 (m, 2H), 7.26 – 7.11 (m, 3H), 6.77 (d, J = 8.4 Hz, 2H), 6.11 (dd, J = 7.1, 1.7 Hz, 1H), 3.70 (s, 6H).

^{13}C NMR (101 MHz, DMSO-d_6) δ 155.86, 142.64, 134.48, 128.39, 126.79, 126.21, 125.98, 124.70, 124.14, 122.66, 119.16, 117.41, 106.71, 105.34, 56.13.

ESI-HRMS m/z: $[M+H]^+$ Calcd for $C_{18}H_{17}NO_2$ 280.1332; found 280.1329

***N*-(2,5-dimethoxyphenyl)naphthalen-1-aminium chloride (AR-41)**

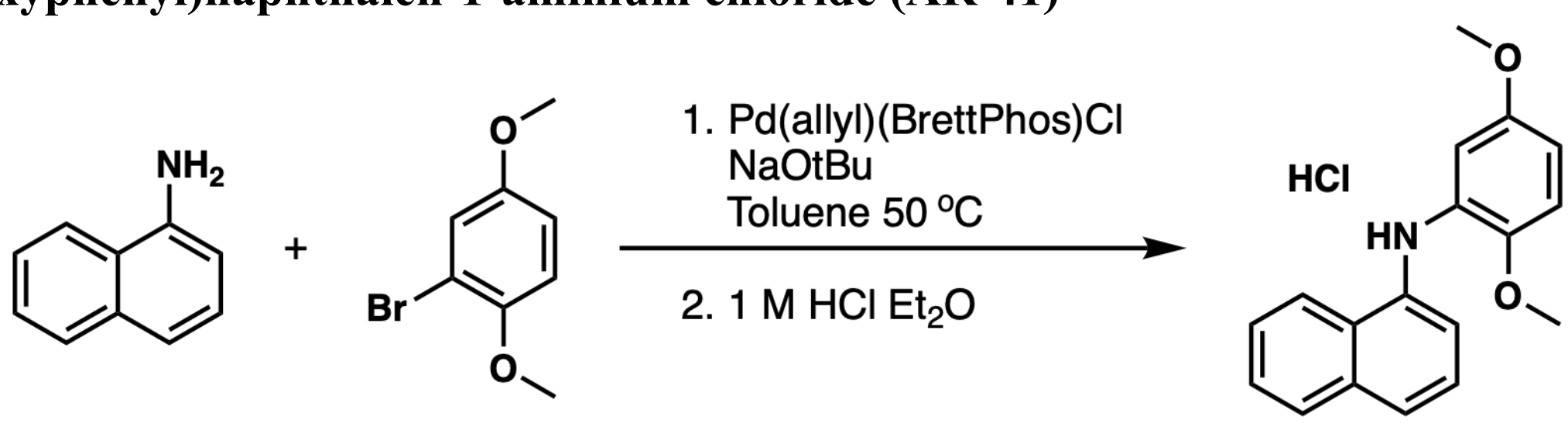

AR-41

General Procedure I: Reaction scale: 100 mg (0.70 mmol) of naphthalen-1-amine. Yields AR-41 as white solid (142 mg, 64%).

^{1}H NMR (400 MHz, DMSO-d_6) δ 9.08 (s, 2H), 8.08 – 8.01 (m, 1H), 7.89 (dd, J = 8.1, 1.5 Hz, 1H), 7.56 (d, J = 8.2 Hz, 1H), 7.48 (m, 2H), 7.40 (dd, J = 7.8, 7.8 Hz, 1H), 7.12 (dd, J = 7.5, 1.1 Hz, 1H), 6.93 (d, J = 8.8 Hz, 1H), 6.40 (dd, J = 8.8, 3.0 Hz, 1H), 6.30 (d, J = 2.9 Hz, 1H), 3.79 (s, 3H), 3.57 (s, 3H).

^{13}C NMR (101 MHz, DMSO-d_6) δ 154.08, 144.14, 139.59, 135.58, 134.68, 128.62, 127.73, 126.65, 126.43, 125.70, 123.24, 122.48, 116.40, 112.64, 104.46, 104.16, 56.58, 55.55.

ESI-HRMS m/z: $[M+H]^+$ Calcd for $C_{18}H_{17}NO_2$ 280.1332; found 280.1324

***N*-(2,4-dimethoxyphenyl)naphthalen-1-aminium chloride (AR-42)**

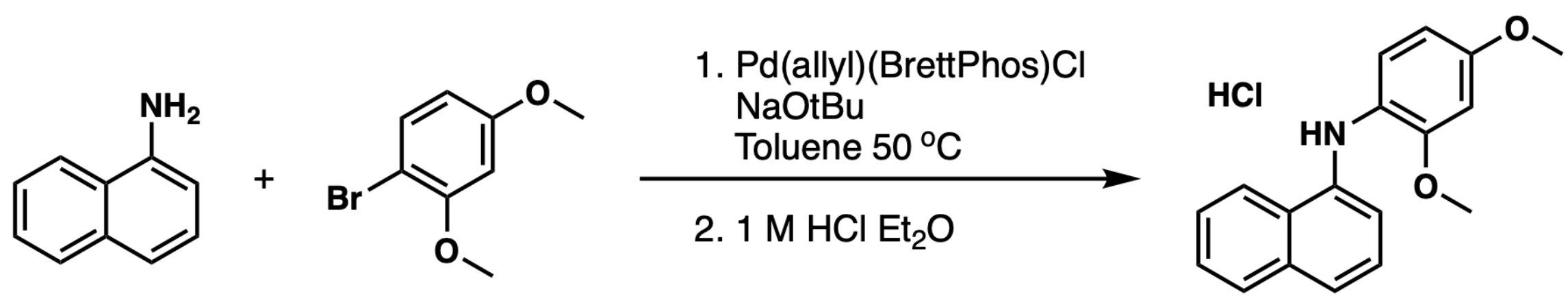

AR-42

General Procedure I: Reaction scale: 100 mg (0.70 mmol) of naphthalen-1-amine. Yields AR-42 as white solid (149 mg, 68%).

^{1}H NMR (400 MHz, DMSO-d_6) δ 9.76 (s, 2H), 8.29 – 8.21 (m, 1H), 7.85 – 7.77 (m, 1H), 7.51 – 7.39 (m, 2H), 7.29 – 7.17 (m, 2H), 7.02 (d, J = 8.5 Hz, 1H), 6.69 (d, J = 2.7 Hz, 1H), 6.55 – 6.47 (m, 2H), 3.77 (s, 3H), 3.74 (s, 3H).

^{13}C NMR (101 MHz, DMSO) δ 157.17, 154.36, 142.99, 134.63, 128.44, 126.82, 126.18, 125.53, 124.99, 124.90, 124.87, 122.72, 118.45, 108.68, 105.17, 100.11, 55.96, 55.80.

ESI-HRMS m/z: $[M+H]^+$ Calcd for $C_{18}H_{17}NO_2$ 280.1332; found 280.1324

4-(naphthalen-1-ylammonio)-2-(trifluoromethyl)pyridin-1-ium dichloride (AR-43)

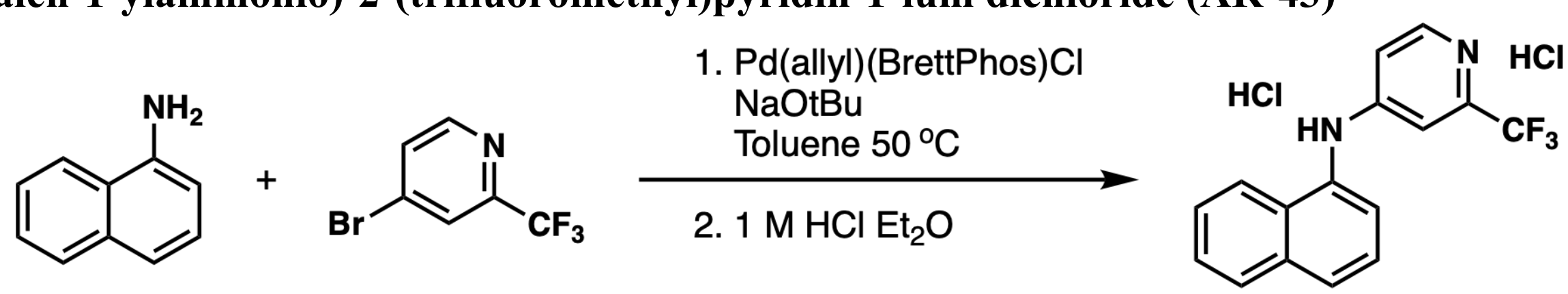

AR-43

General Procedure I: Reaction scale: 100 mg (0.70 mmol) of naphthalen-1-amine. Yields AR-43 as white solid (120 mg, 48%).

^{1}H NMR (400 MHz, DMSO-d_6) δ 10.74 (s, 1H), 8.28 (d, J = 6.3 Hz, 1H), 8.01 (ddd, J = 12.2, 7.9, 1.9 Hz, 2H), 7.94 (d, J = 7.9 Hz, 1H), 7.65 – 7.52 (m, 4H), 7.43 (d, J = 2.4 Hz, 1H), 7.29 (s, 2H), 6.93 (dd, J = 6.4, 2.4 Hz, 1H).

^{13}C NMR (101 MHz, DMSO-d_6) δ 156.48, 147.14, 143.05 (d, J = 34.8 Hz), 134.80, 134.11, 129.05, 128.96, 127.58, 127.25, 127.19, 126.52, 123.10, 122.68, 121.15 (d, J = 274.7 Hz), 110.28, 107.05.

^{19}F NMR (376 MHz, DMSO-d_6) δ -66.27 (s, 1F).

ESI-HRMS m/z: $[M+H]^+$ Calcd for $C_{16}H_{11}F_3N_2$ 289.0947; found 289.0940

N-(3,5-dimethoxyphenyl)naphthalen-1-aminium *chloride* (AR-44)

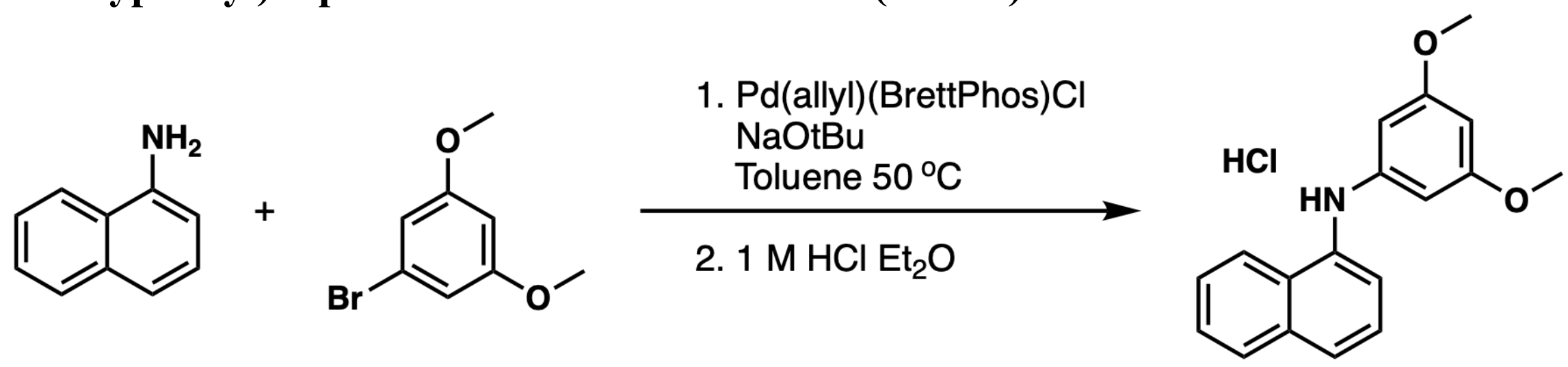

AR-44

General Procedure I: Reaction scale: 100 mg (0.70 mmol) of naphthalen-1-amine. Yields AR-44 as white solid (217 mg, 98%).

^{1}H NMR (400 MHz, DMSO-d_6) δ 10.06 (s, 2H), 8.20 – 8.10 (m, 1H), 7.91 – 7.84 (m, 1H), 7.57 – 7.53 (m, 1H), 7.52 – 7.44 (m, 2H), 7.44 – 7.34 (m, 2H), 6.21 (d, *J* = 2.1 Hz, 2H), 5.98 (t, *J* = 2.2 Hz, 1H), 3.65 (s, 6H).

^{13}C NMR (101 MHz, DMSO-d_6) δ 161.14, 147.28, 138.86, 134.44, 128.15, 127.54, 126.13, 126.12, 125.19, 123.01, 122.11, 116.01, 94.97, 91.52, 54.95.

ESI-HRMS m/z: $[M+H]^+$ Calcd for $C_{18}H_{17}NO_2$ 280.1332; found 280.1324

N-(4-(ethoxycarbonyl)-3-methylphenyl)naphthalen-1-aminium chloride (AR-45)

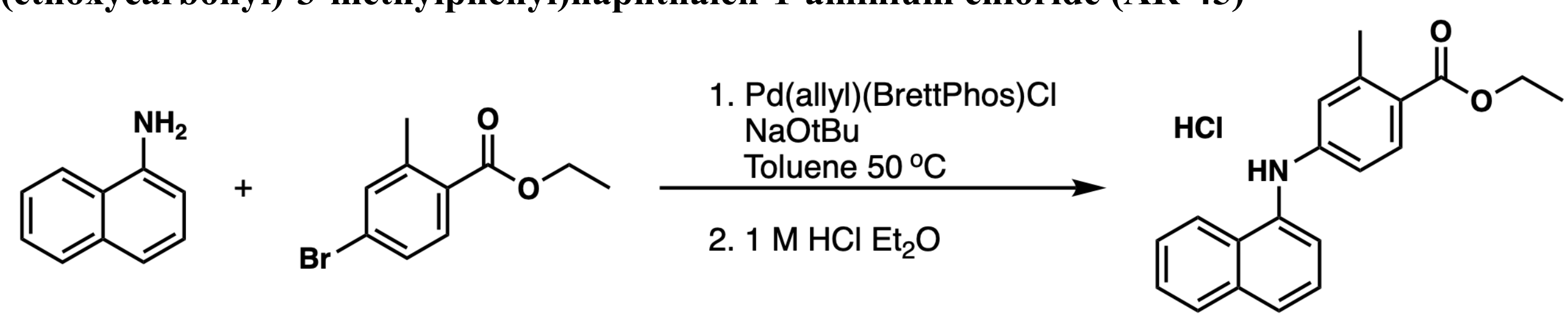

AR-45

General Procedure I: Reaction scale: 100 mg (0.70 mmol) of naphthalen-1-amine. Yields AR-45 as white solid (176 mg, 74%).

^{1}H NMR (400 MHz, DMSO-d_6) δ 8.70 (s, 2H), 8.06 (d, *J* = 7.9 Hz, 1H), 7.94 (d, *J* = 7.7 Hz, 1H), 7.74 (d, *J* = 8.5 Hz, 1H), 7.70 (d, *J* = 7.9 Hz, 1H), 7.57 – 7.41 (m, 4H), 6.81 – 6.72 (m, 2H), 4.20 (q, *J* = 7.1 Hz, 2H), 2.44 (s, 3H), 1.27 (t, *J* = 7.1 Hz, 3H).

^{13}C NMR (101 MHz, DMSO-d_6) δ 166.74, 150.09, 142.11, 137.57, 134.87, 132.93, 128.83, 128.71, 126.74, 126.56, 126.13, 124.41, 123.39, 119.39, 118.71, 117.43, 112.07, 60.02, 22.61, 14.76.

ESI-HRMS m/z: $[M+H]^+$ Calcd for $C_{20}H_{19}NO_2$ 306.1489; found 306.1484

_N_-(4-ethoxyphenyl)naphthalen-1-aminium chloride (AR-46)

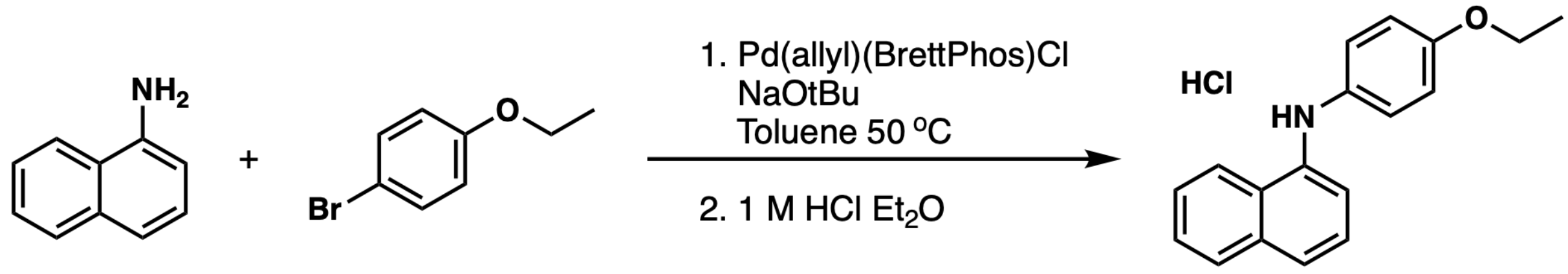

AR-46

General Procedure I: Reaction scale: 100 mg (0.70 mmol) of naphthalen-1-amine. Yields AR-46 as white solid (162 mg, 77%).

^{1}H NMR (850 MHz, DMSO-d_6) δ 9.84 (s, 2H), 8.23 (ddt, J = 8.3, 1.5, 0.8 Hz, 1H), 7.84 – 7.81 (m, 1H), 7.46 (dddd, J = 25.5, 8.2, 6.7, 1.3 Hz, 2H), 7.36 (d, J = 8.1 Hz, 1H), 7.30 (t, J = 7.8 Hz, 1H), 7.07 (d, J = 8.2 Hz, 2H), 7.03 (d, J = 7.5 Hz, 1H), 6.91 – 6.84 (m, 2H), 3.99 (q, J = 6.9 Hz, 2H), 1.32 (t, J = 6.9 Hz, 3H).

^{13}C NMR (101 MHz, DMSO-d_6) δ 153.83, 141.95, 137.31, 134.85, 128.48, 126.73, 126.40, 125.81, 125.10, 122.93, 121.95, 119.65, 115.57, 110.15, 63.64, 15.24.

ESI-HRMS m/z: $[M+H]^+$ Calcd for $C_{18}H_{17}NO$ 264.1383; found 264.1379

_N_-(4-methoxy-2,6-dimethylphenyl)naphthalen-1-aminium chloride (AR-47)

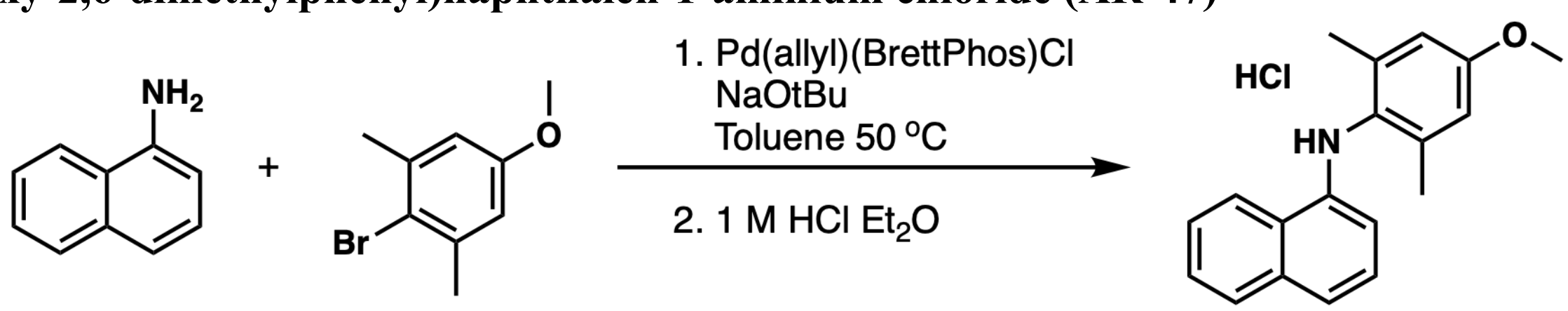

General Procedure I: Reaction scale: 100 mg (0.70 mmol) of naphthalen-1-amine. Yields AR-47 as white solid (95 mg, 43%).

^{1}H NMR (850 MHz, DMSO-d_6) δ 8.43 – 8.41 (m, 1H), 7.79 – 7.78 (m, 1H), 7.48 – 7.43 (m, 3H), 7.13 – 7.10 (m, 2H), 6.77 (s, 2H), 5.88 (dd, J = 6.6, 2.0 Hz, 1H), 3.76 (s, 3H), 2.10 (s, 6H).

^{13}C NMR (214 MHz, DMSO-d_6) δ 156.95, 143.21, 137.71, 134.34, 131.39, 127.99, 126.71, 125.69, 124.11, 122.82, 121.91, 115.92, 113.45, 103.78, 55.03, 18.00.

ESI-HRMS m/z: $[M+H]^+$ Calcd for $C_{19}H_{19}NO$ 278.1540; found 278.1534

_N_-(4-fluoro-2-methoxyphenyl)naphthalen-1-aminium chloride (AR-48)

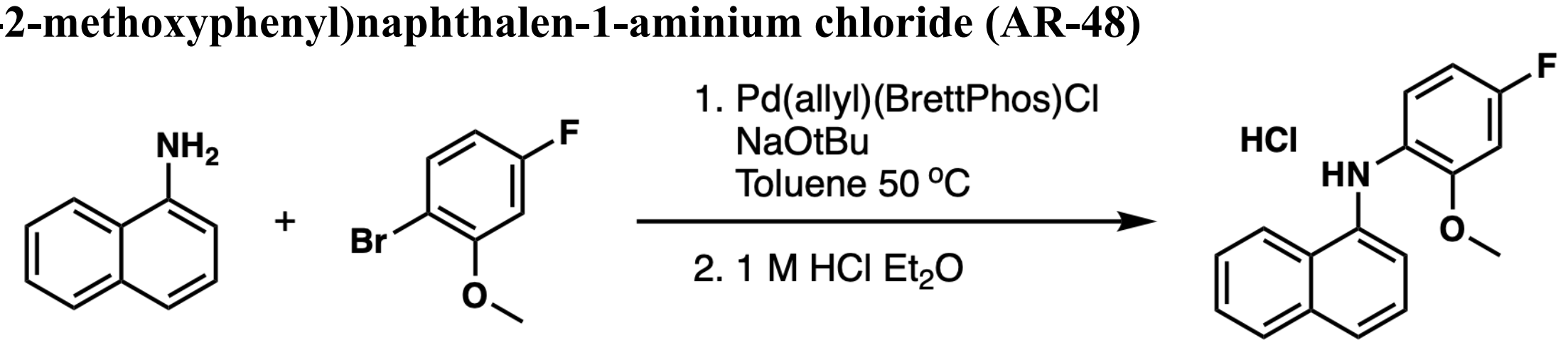

AR-48

General Procedure I: Reaction scale: 100 mg (0.70 mmol) of naphthalen-1-amine. Yields AR-48 as white solid (150 mg, 71%).

^{1}H NMR (400 MHz, DMSO-d_6) δ 9.19 (s, 2H), 8.24 – 8.12 (m, 1H), 7.89 – 7.80 (m, 1H), 7.52 – 7.40 (m, 2H), 7.39 (d, J = 8.1 Hz, 1H), 7.29 (t, J = 7.8 Hz, 1H), 7.00 (dd, J = 10.9, 2.9 Hz, 1H), 6.94 (dd, J = 8.7, 6.2 Hz, 1H), 6.74 (dd, J = 7.5, 1.1 Hz, 1H), 6.69 (td, J = 8.5, 2.8 Hz, 1H), 3.80 (s, 3H).

^{13}C NMR (214 MHz, DMSO-d_6) δ 158.25 (d, J = 237.8 Hz), 152.40 (d, J = 10.0 Hz), 140.97, 134.22, 129.06, 128.08, 126.26, 125.86, 125.67, 124.85, 122.53, 121.72 (d, J = 9.6 Hz), 119.92, 111.48, 106.31 (d, J = 21.7 Hz), 100.21 (d, J = 26.6 Hz), 55.91.

^{19}F NMR (376 MHz, DMSO-d_6) δ -118.69 (ddd, J = 11.0, 8.7, 6.6 Hz, 1F).

ESI-HRMS m/z: $[M+H]^+$ Calcd for $C_{17}H_{14}FNO$ 268.1132; found 268.1129

***N*-(3,5-difluoro-4-methoxyphenyl)naphthalen-1-aminium chloride (AR-49)**

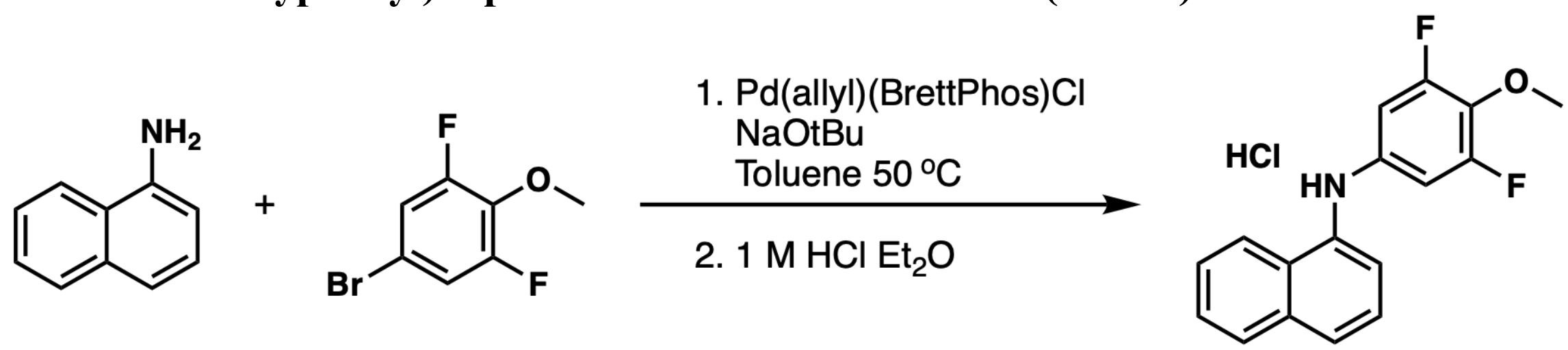

AR-49

General Procedure I: Reaction scale: 100 mg (0.70 mmol) of naphthalen-1-amine. Yields AR-49 as white solid (153 mg, 68%).

^{1}H NMR (400 MHz, DMSO-d_6) δ 8.86 (s, 2H), 8.16 – 8.06 (m, 1H), 7.95 – 7.86 (m, 1H), 7.63 (d, J = 8.0 Hz, 1H), 7.56 – 7.47 (m, 2H), 7.44 (dd, J = 7.7, 7.7 Hz, 1H), 7.39 (d, J = 7.5 Hz, 1H), 6.66 (d, J = 10.7 Hz, 2H), 3.80 (s, 3H).

^{13}C NMR (214 MHz, DMSO-d_6) δ 156.07 (dd, J = 243.4, 8.2 Hz), 142.21 (dd, J = 12.4, 12.4 Hz), 137.78, 134.44, 128.24, 127.68 (dd, J = 15.6, 15.6 Hz), 127.67, 126.26, 126.13, 125.57, 123.17, 122.70, 116.84, 99.28 (dd, J = 21.5, 4.3 Hz), 62.03.

^{19}F NMR (376 MHz, DMSO-d_6) δ -128.83 (s, 1F).

ESI-HRMS m/z: $[M+H]^+$ Calcd for $C_{17}H_{13}F_2NO$ 286.1038; found 286.1032

***N*-(3-fluoro-5-methoxyphenyl)naphthalen-1-aminium chloride (AR-50)**

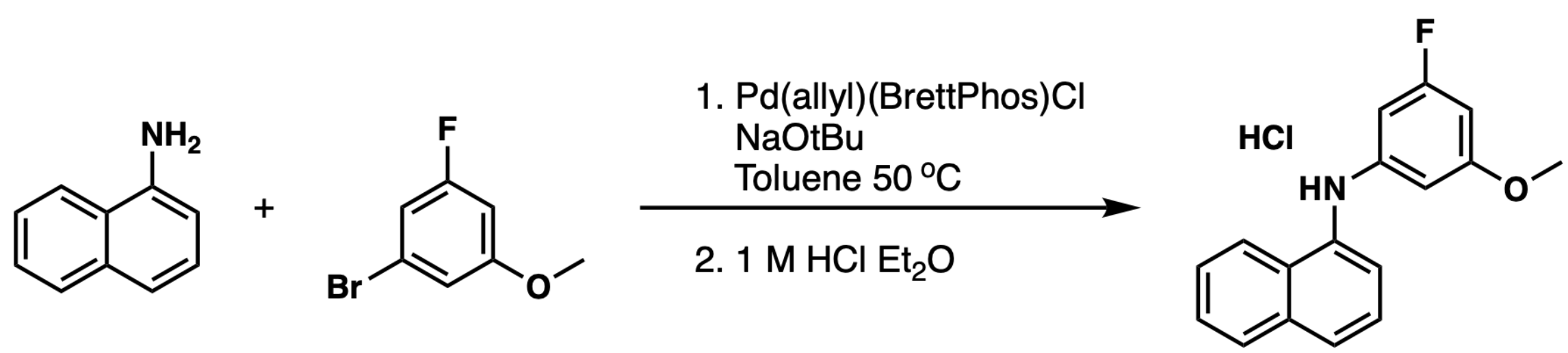

General Procedure I: Reaction scale: 100 mg (0.70 mmol) of naphthalen-1-amine. Yields AR-50 as white solid (172 mg, 81%).

^{1}H NMR (850 MHz, DMSO-d_6) δ 8.13 – 8.08 (m, 1H), 7.91 – 7.88 (m, 1H), 7.62 (d, J = 8.0 Hz, 1H), 7.53 – 7.46 (m, 2H), 7.45 (dd, J = 7.7, 7.7 Hz, 1H), 7.41 (d, J = 7.4 Hz, 1H), 6.38 (t, J = 2.2 Hz, 1H), 6.32 (ddd, J = 11.4, 2.2, 2.2 Hz, 1H), 6.19 (ddd, J = 11.0, 2.3, 2.3 Hz, 1H), 3.68 (s, 3H).

^{13}C NMR (214 MHz, DMSO-d_6) δ 163.90 (d, *J* = 238.8 Hz), 161.35 (d, *J* = 13.9 Hz), 148.28 (d, *J* = 13.8 Hz), 137.94, 134.45, 128.23, 127.99, 126.23, 126.13, 125.50, 123.21, 122.97, 117.58, 97.28, 94.74 (d, *J* = 25.9 Hz), 91.81 (d, *J* = 25.7 Hz), 55.29.

^{19}F NMR (376 MHz, DMSO-d_6) δ -111.87 (s, 1F).

ESI-HRMS m/z: $[M+H]^+$ Calcd for $C_{17}H_{14}FNO$ 268.1132; found 268.1129

DFT Results:

DFT Calculations: All computational results were done in ORCA. Structures were optimized using the ωB97X-D4/def2-TZVP level of theory. Calculations utilized the DefGrid3 and VeryTightSCF keywords. Numerical frequencies were calculated for use in excited state dynamic (ESD) calculations as well as to confirm a true geometry minima had been reached. Where applicable, the CPCM solvation model was used. Outputs were visualized in ChemCraft 1.8.

Table F: Oscillator strengths of unsubstituted derivatives with varied linkers

	S_0 (*f*)	S_1 (*f*)
ALK	0.25	0.27
AM	0.38	0.46
SULF	0.00	0.01
UR	0.22	0.37
NPN	0.35	0.38
NPN CPCM solvation		
Hexane	0.46	0.61
Water	0.62	0.63

Table G: Rates calculated through ORCA ESD module

	Fluorescense (s^{-1})	IC (s^{-1})	ISC (s^{-1})
ALK	2.28E+08	2.09E+09	2.14E+08
AM	2.52E+08	9.02E+09	1.78E+09
SULF	1.57E+07	1.83E+11	9.46E+07
UR	2.22E+09	1.53E+10	2.45E+08
NPN	1.55E+08	6.42E+09	3.49E+08
NPN CPCM solvation			
Hexane	5.01E+08	6.29E+09	3.37E+08
Water	1.47E+08	8.80E+09	1.89E+09

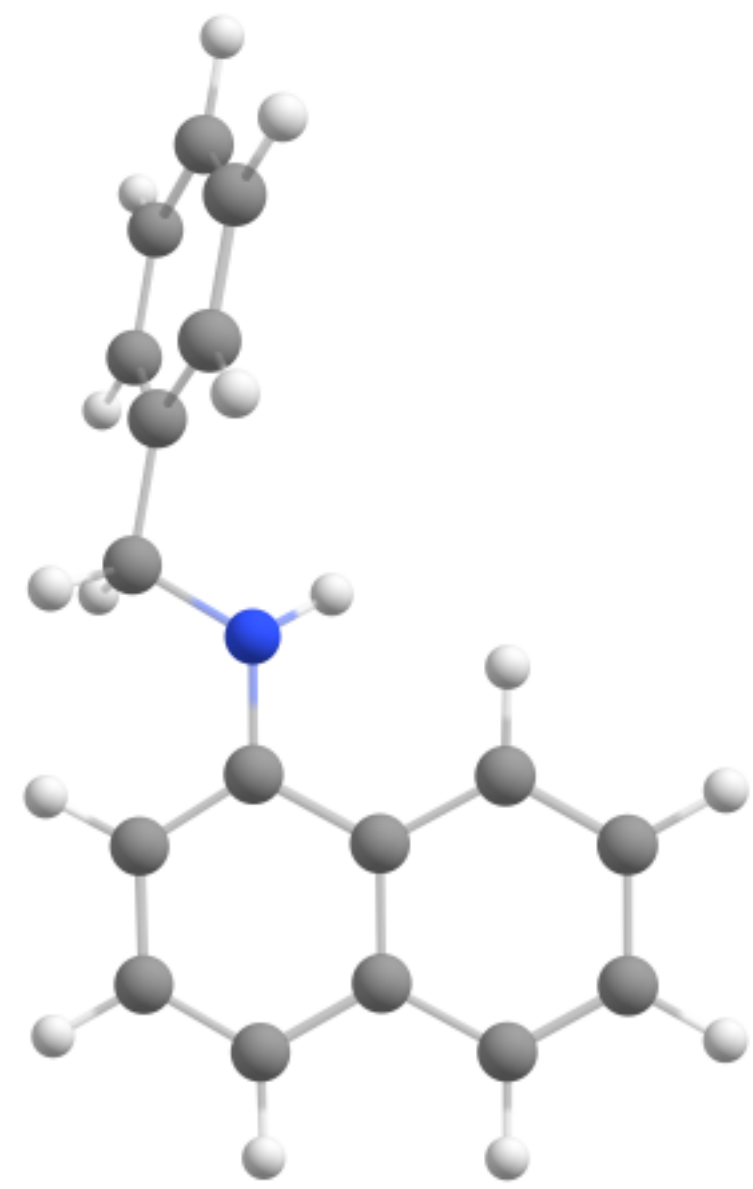

ALK Ground State (Gas)

C	-2.857135000	0.030897000	-0.068428000
C	-2.115479000	1.236862000	-0.136748000
C	-2.819522000	2.461708000	-0.200883000
C	-4.236624000	2.437797000	-0.231647000
C	-4.926261000	1.259284000	-0.186852000
C	-4.224888000	0.038618000	-0.095056000
C	-0.677173000	1.256818000	-0.131432000
C	-0.023037000	2.466460000	-0.146621000
C	-0.745369000	3.680839000	-0.181339000
C	-2.106320000	3.690251000	-0.220439000
N	0.004084000	0.046330000	-0.076479000
C	1.446504000	0.013392000	-0.228901000
C	1.942228000	-1.411494000	-0.200426000
C	2.699870000	-1.923511000	-1.247024000
C	3.160326000	-3.235470000	-1.213514000
C	2.859658000	-4.049140000	-0.130948000
C	2.097575000	-3.545763000	0.918484000
C	1.643336000	-2.235983000	0.883693000
H	-2.344137000	-0.917823000	0.036524000
H	-4.767011000	3.383233000	-0.288461000
H	-6.010678000	1.258507000	-0.210287000
H	-4.772946000	-0.895442000	-0.036012000
H	1.058357000	2.504405000	-0.144104000
H	-0.193500000	4.615014000	-0.191637000
H	-2.660326000	4.621743000	-0.261890000
H	-0.455574000	-0.711559000	-0.554427000
H	1.774239000	0.500356000	-1.158807000
H	1.894581000	0.572957000	0.599577000
H	2.932377000	-1.290660000	-2.098097000
H	3.751284000	-3.621204000	-2.037291000
H	3.215940000	-5.073185000	-0.102981000
H	1.860518000	-4.176932000	1.768365000
H	1.047199000	-1.841749000	1.700302000

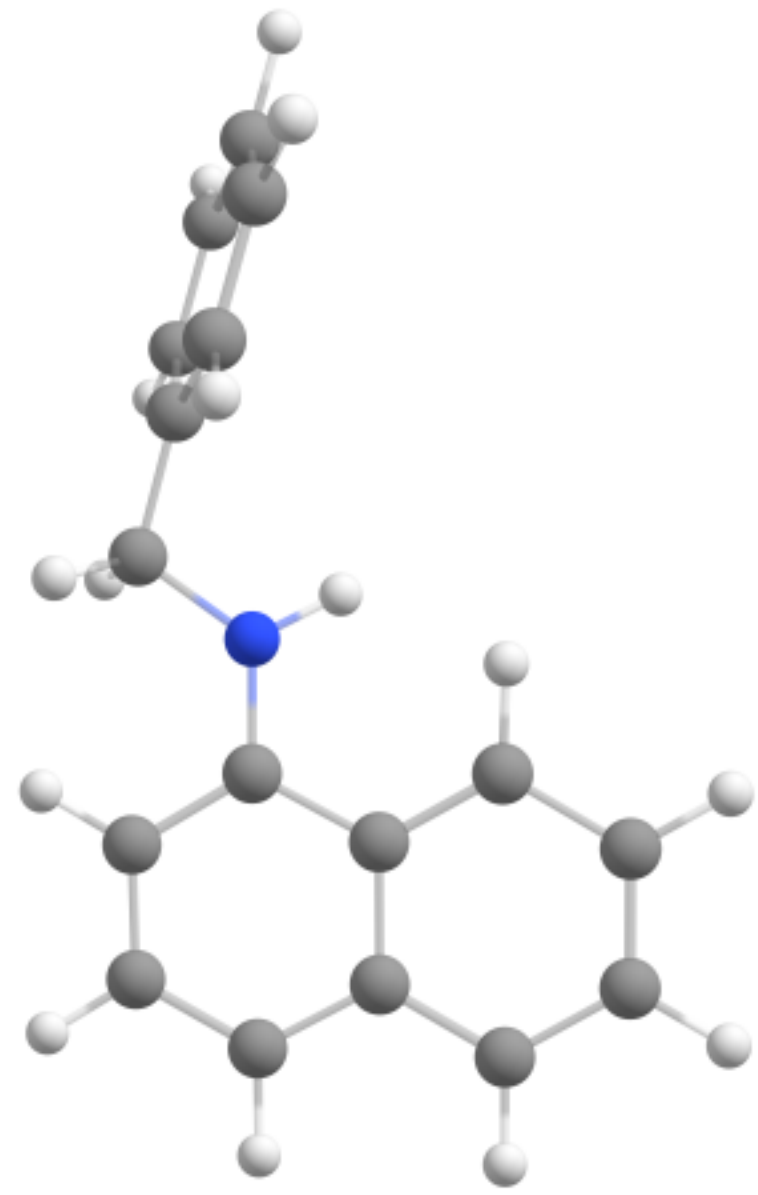

ALK S_1 (Gas)

C	-2.736903000	-0.018792000	-0.091079000
C	-2.066792000	1.209482000	-0.141159000
C	-2.815397000	2.424151000	-0.200467000
C	-4.226546000	2.364061000	-0.258355000
C	-4.878740000	1.116290000	-0.246815000
C	-4.158295000	-0.051690000	-0.160584000
C	-0.631781000	1.314972000	-0.147440000
C	0.031025000	2.578840000	-0.087035000
C	-0.714128000	3.726392000	-0.104653000
C	-2.122247000	3.646738000	-0.173969000
N	0.085078000	0.174852000	-0.227863000
C	1.534354000	0.082210000	-0.255340000
C	1.953272000	-1.365378000	-0.211782000
C	2.586440000	-1.957448000	-1.297618000
C	2.954726000	-3.297499000	-1.253430000
C	2.684257000	-4.054522000	-0.122427000
C	2.046233000	-3.469158000	0.966099000
C	1.684616000	-2.130966000	0.921616000
H	-2.206644000	-0.952256000	0.055869000
H	-4.793832000	3.285990000	-0.313831000
H	-5.961659000	1.079194000	-0.302071000
H	-4.667528000	-1.008944000	-0.143316000
H	1.111841000	2.620477000	-0.056326000
H	-0.229718000	4.694948000	-0.069124000
H	-2.699518000	4.565806000	-0.190217000
H	-0.420649000	-0.664661000	-0.464218000
H	1.936492000	0.563298000	-1.155285000
H	1.939143000	0.615975000	0.610668000
H	2.793598000	-1.367427000	-2.185038000
H	3.449731000	-3.749518000	-2.105909000
H	2.969284000	-5.100264000	-0.086954000
H	1.835011000	-4.056693000	1.852857000
H	1.187370000	-1.672906000	1.771412000

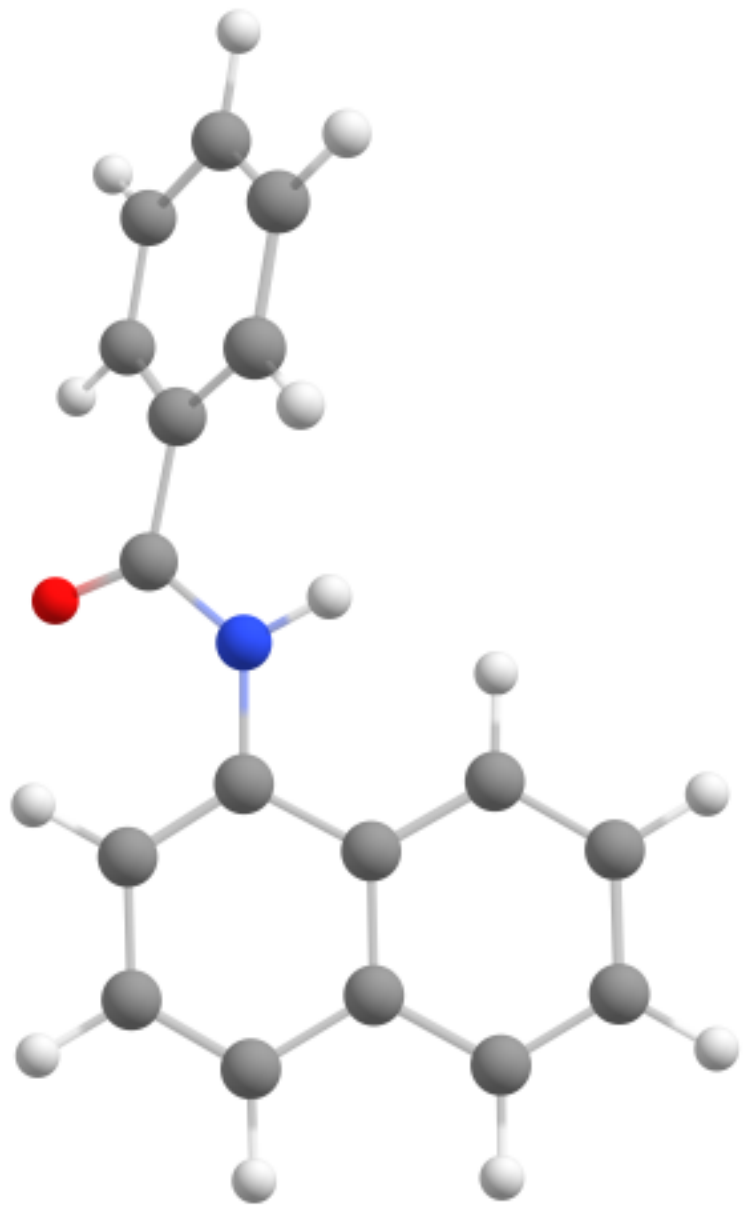

AM Ground State (Gas)

C	-0.046421000	-2.482588000	-0.166895000
C	-0.707589000	-1.231440000	-0.046247000
C	-2.121943000	-1.224834000	0.022275000
C	-2.821280000	-2.458648000	0.003481000
C	-2.156754000	-3.647024000	-0.091784000
C	-0.748255000	-3.655263000	-0.187693000
C	-0.015871000	0.021846000	0.000786000
C	-0.716124000	1.197833000	0.063363000
C	-2.127037000	1.182964000	0.102278000
C	-2.817864000	0.008146000	0.094795000
N	1.392282000	-0.009564000	0.012128000
C	2.255657000	0.980984000	-0.376516000
C	3.710616000	0.637783000	-0.207370000
C	4.616878000	1.253802000	-1.065753000
C	5.972368000	0.983215000	-0.960468000
C	6.434543000	0.107941000	0.016127000
C	5.537017000	-0.492700000	0.888677000
C	4.177720000	-0.231006000	0.775861000
O	1.905320000	2.047714000	-0.839729000
H	1.030676000	-2.531320000	-0.279751000
H	-3.904808000	-2.437002000	0.063464000
H	-2.704453000	-4.582829000	-0.106064000
H	-0.220906000	-4.597582000	-0.288330000
H	-0.183983000	2.136774000	0.080088000
H	-2.656547000	2.128177000	0.150859000
H	-3.901811000	-0.002849000	0.137675000
H	1.814983000	-0.880185000	0.274910000
H	4.238241000	1.945819000	-1.808536000
H	6.672329000	1.459076000	-1.638452000
H	7.495740000	-0.098886000	0.102609000
H	5.895157000	-1.159826000	1.665006000
H	3.493499000	-0.680900000	1.488265000

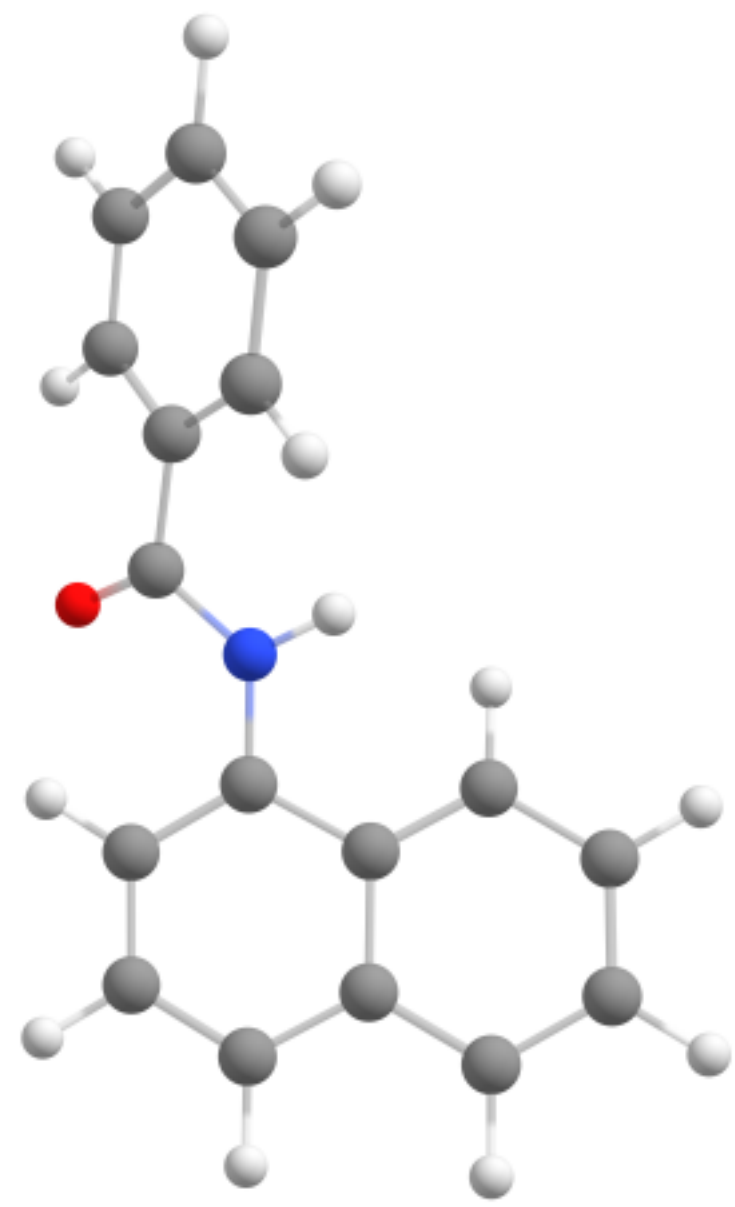

AM S_1 (Gas)

C	-0.102531000	-2.516383000	-0.444791000
C	-0.697475000	-1.282893000	-0.187125000
C	-2.086285000	-1.234414000	0.157243000
C	-2.789384000	-2.446371000	0.310737000
C	-2.153535000	-3.674135000	0.105613000
C	-0.827898000	-3.716714000	-0.278006000
C	0.017352000	-0.032557000	-0.245332000
C	-0.662855000	1.223811000	-0.145136000
C	-2.005285000	1.227038000	0.126743000
C	-2.707689000	0.018349000	0.301857000
N	1.373190000	-0.091440000	-0.289041000
C	2.276983000	0.923152000	-0.645388000
C	3.686301000	0.637053000	-0.294440000
C	4.674000000	1.374478000	-0.956440000
C	6.012014000	1.151607000	-0.686485000
C	6.387365000	0.195062000	0.253964000
C	5.413222000	-0.530707000	0.927523000
C	4.069553000	-0.311803000	0.660126000
O	1.912981000	1.922530000	-1.241137000
H	0.910749000	-2.577309000	-0.827142000
H	-3.836763000	-2.412463000	0.590697000
H	-2.711149000	-4.595136000	0.235700000
H	-0.338589000	-4.666087000	-0.462645000
H	-0.098416000	2.136297000	-0.262196000
H	-2.531147000	2.169355000	0.226559000
H	-3.767767000	0.047342000	0.533083000
H	1.776261000	-0.991310000	-0.068358000
H	4.362938000	2.115539000	-1.682823000
H	6.770049000	1.724003000	-1.209859000
H	7.436790000	0.023006000	0.466624000
H	5.699498000	-1.263031000	1.674418000
H	3.328904000	-0.858238000	1.234517000

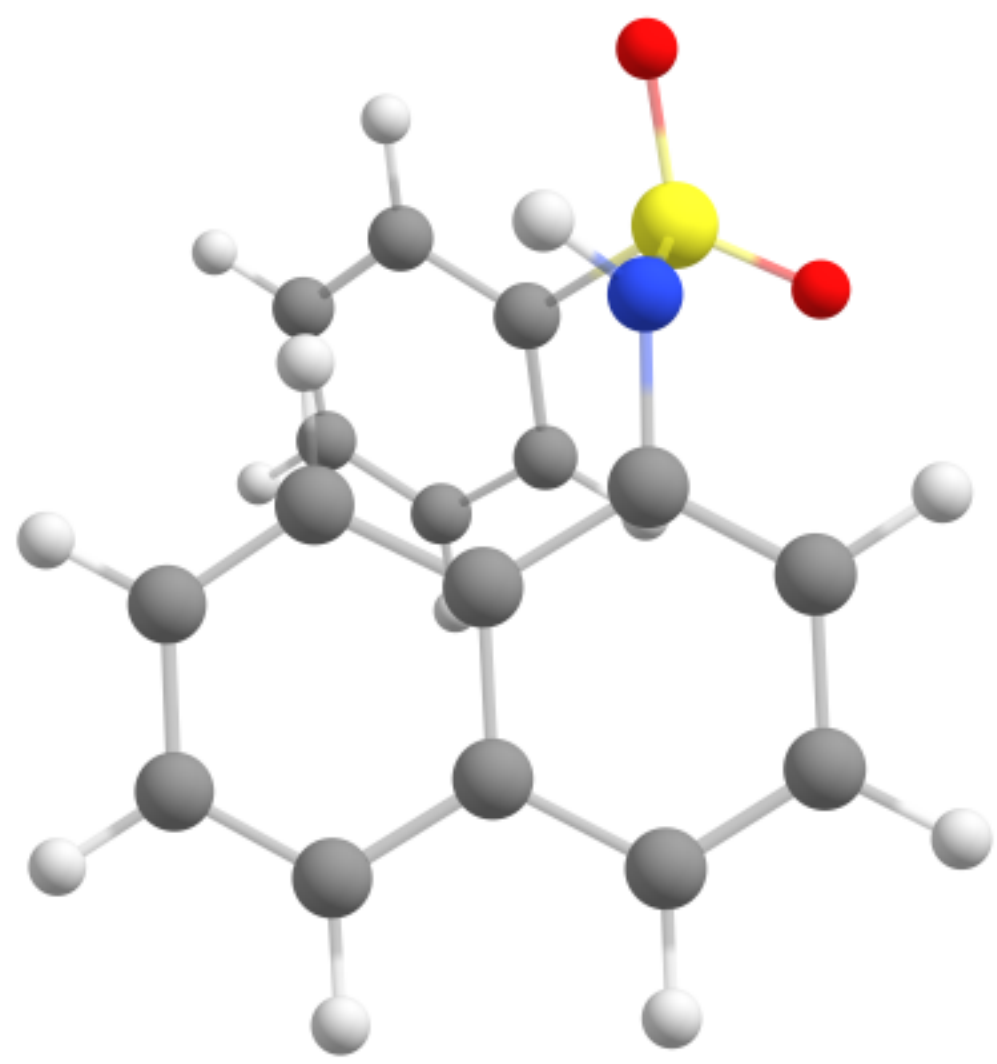

SA Ground State (Gas)

C	0.336254000	2.032405000	1.750086000
C	-0.784297000	1.164271000	1.692700000
C	-1.867610000	1.508198000	0.848379000
C	-1.808796000	2.717036000	0.107706000
C	-0.720763000	3.536939000	0.187939000
C	0.368507000	3.183664000	1.016631000
C	-0.863850000	-0.055116000	2.428688000
C	-1.944456000	-0.881895000	2.303353000
C	-3.007595000	-0.540166000	1.440424000
C	-2.975406000	0.629946000	0.738624000
N	0.205126000	-0.451985000	3.285082000
S	1.281078000	-1.591079000	2.762778000
C	1.986151000	-0.885914000	1.308263000
C	1.278298000	-0.950812000	0.114947000
C	1.776855000	-0.292127000	-0.999233000
C	2.973426000	0.409834000	-0.916073000
C	3.682702000	0.450613000	0.278603000
C	3.186997000	-0.195755000	1.401887000
O	2.287467000	-1.647181000	3.779145000
O	0.529308000	-2.741815000	2.372882000
H	1.192345000	1.773076000	2.360055000
H	-2.648151000	2.975109000	-0.530245000
H	-0.684826000	4.456678000	-0.385463000
H	1.237980000	3.829997000	1.065272000
H	-1.964800000	-1.804731000	2.868790000
H	-3.854757000	-1.210828000	1.351103000
H	-3.795109000	0.903979000	0.082341000
H	0.671655000	0.283512000	3.796193000
H	0.351933000	-1.509592000	0.067053000
H	1.230545000	-0.328380000	-1.934860000
H	3.360044000	0.921755000	-1.790649000
H	4.624254000	0.985340000	0.335404000
H	3.721631000	-0.178767000	2.344061000

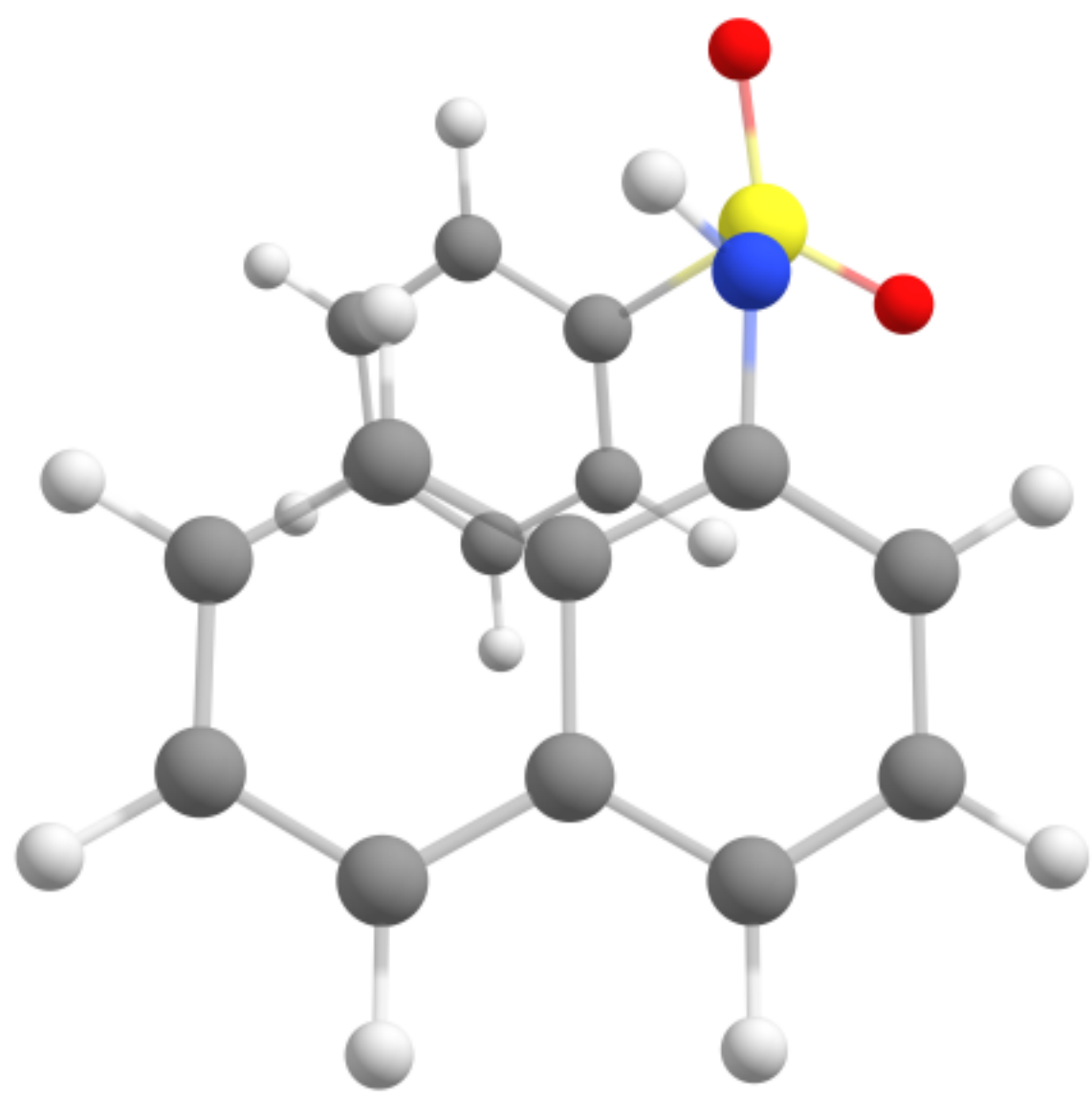

SA S_1 (Gas)

C	0.324491000	2.109819000	1.788201000
C	-0.761670000	1.218634000	1.713296000
C	-1.903377000	1.572846000	0.837729000
C	-1.881851000	2.822223000	0.188845000
C	-0.806110000	3.692896000	0.324265000
C	0.312396000	3.327044000	1.113947000
C	-0.820548000	-0.024062000	2.387208000
C	-1.877149000	-0.916862000	2.186539000
C	-2.931751000	-0.591340000	1.305105000
C	-2.942446000	0.643132000	0.657798000
N	0.212794000	-0.394735000	3.279380000
S	1.300884000	-1.562007000	2.825342000
C	1.990651000	-0.925398000	1.334061000
C	1.277030000	-1.060515000	0.149605000
C	1.770024000	-0.465994000	-1.002489000
C	2.965483000	0.242112000	-0.964587000
C	3.677930000	0.356066000	0.223320000
C	3.188096000	-0.224821000	1.384460000
O	2.304956000	-1.550896000	3.844774000
O	0.557992000	-2.739146000	2.504504000
H	1.205013000	1.845172000	2.360939000
H	-2.722846000	3.094665000	-0.440408000
H	-0.815803000	4.647774000	-0.187674000
H	1.161517000	3.994791000	1.192068000
H	-1.869639000	-1.855464000	2.723670000
H	-3.741770000	-1.294399000	1.157318000
H	-3.763532000	0.902979000	-0.002157000
H	0.684809000	0.369050000	3.744399000
H	0.353891000	-1.626551000	0.136439000
H	1.221126000	-0.558728000	-1.932681000
H	3.347993000	0.702577000	-1.869013000
H	4.617380000	0.896838000	0.245703000
H	3.726176000	-0.151489000	2.321957000

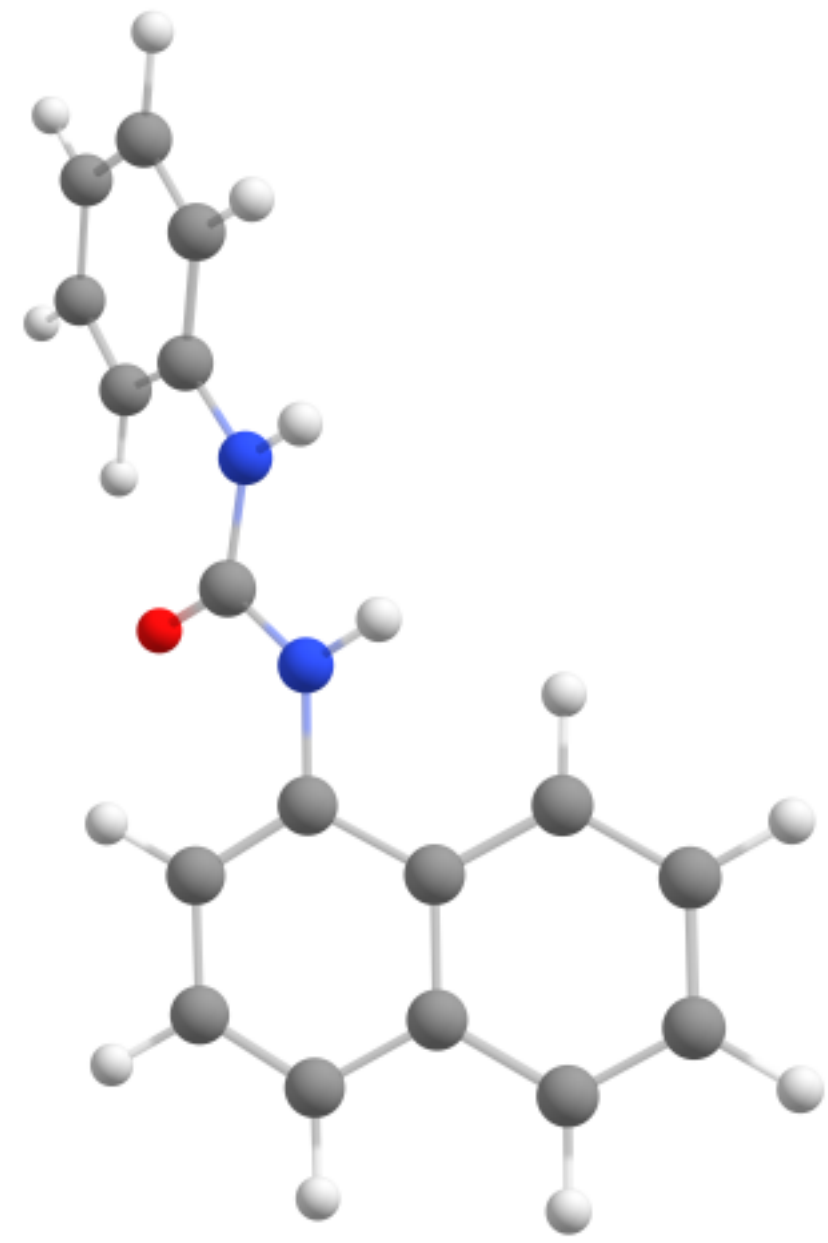

UR Ground State (Gas)

C	0.000186000	-2.384104000	-0.384244000
C	-0.692231000	-1.192843000	-0.040545000
C	-2.098868000	-1.252894000	0.112184000
C	-2.757177000	-2.500123000	-0.041339000
C	-2.061596000	-3.633879000	-0.347384000
C	-0.662849000	-3.569873000	-0.532136000
C	-0.037545000	0.064661000	0.149173000
C	-0.767210000	1.190815000	0.421470000
C	-2.172120000	1.118753000	0.530991000
C	-2.826032000	-0.070281000	0.396878000
N	1.370402000	0.091005000	0.083183000
C	2.140717000	1.145349000	-0.372924000
N	3.492896000	0.876318000	-0.277121000
O	1.678964000	2.176790000	-0.810140000
C	4.563880000	1.718147000	-0.635373000
C	5.834830000	1.348708000	-0.190874000
C	6.945372000	2.107513000	-0.521319000
C	6.805155000	3.250600000	-1.297888000
C	5.540231000	3.614696000	-1.737662000
C	4.417292000	2.861500000	-1.418923000
H	1.067418000	-2.361988000	-0.572708000
H	-3.834521000	-2.533059000	0.086645000
H	-2.578327000	-4.580216000	-0.463027000
H	-0.114565000	-4.465188000	-0.804278000
H	-0.262544000	2.137434000	0.547987000
H	-2.726480000	2.026620000	0.742194000
H	-3.904487000	-0.128747000	0.499025000
H	1.838259000	-0.766235000	0.314159000
H	3.762700000	0.054481000	0.235489000
H	5.952637000	0.458370000	0.421014000
H	7.923981000	1.803269000	-0.165955000
H	7.671363000	3.848860000	-1.556344000
H	5.415381000	4.504107000	-2.346222000
H	3.438386000	3.156086000	-1.763533000

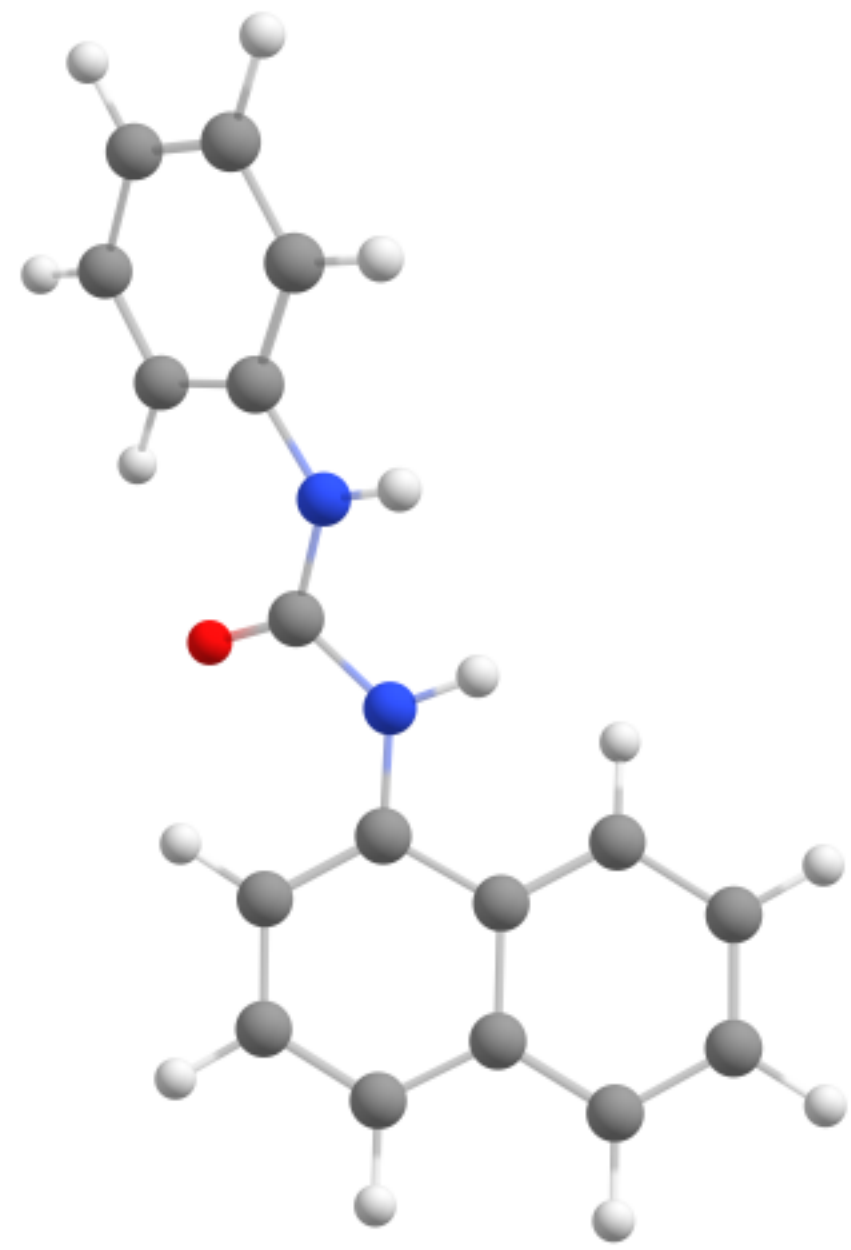

UR S_1 (Gas)

C	-0.048438000	-2.360694000	-0.634549000
C	-0.705787000	-1.202192000	-0.208063000
C	-2.065151000	-1.291507000	0.236806000
C	-2.678618000	-2.557286000	0.310507000
C	-1.975425000	-3.716315000	-0.064795000
C	-0.686492000	-3.625591000	-0.537131000
C	-0.082256000	0.086559000	-0.171367000
C	-0.816738000	1.281010000	0.114581000
C	-2.130241000	1.165565000	0.476740000
C	-2.744651000	-0.104432000	0.555939000
N	1.279334000	0.108390000	-0.309255000
C	2.095046000	1.205825000	-0.613538000
N	3.418588000	0.924287000	-0.373639000
O	1.665044000	2.249614000	-1.053189000
C	4.535123000	1.745196000	-0.627857000
C	5.769062000	1.292025000	-0.155976000
C	6.918836000	2.030272000	-0.377370000
C	6.855373000	3.231695000	-1.072914000
C	5.627104000	3.677277000	-1.540463000
C	4.463625000	2.948873000	-1.327293000
H	0.920618000	-2.307897000	-1.118793000
H	-3.700988000	-2.626348000	0.665257000
H	-2.459941000	-4.683655000	0.009797000
H	-0.150720000	-4.515844000	-0.846598000
H	-0.318544000	2.236469000	0.056671000
H	-2.705677000	2.052652000	0.714224000
H	-3.787660000	-0.169019000	0.849751000
H	1.725043000	-0.793217000	-0.197168000
H	3.623198000	0.081784000	0.136803000
H	5.825946000	0.353482000	0.388602000
H	7.868375000	1.663598000	-0.003092000
H	7.753682000	3.812699000	-1.247117000
H	5.563548000	4.613253000	-2.085037000
H	3.513284000	3.308123000	-1.690022000

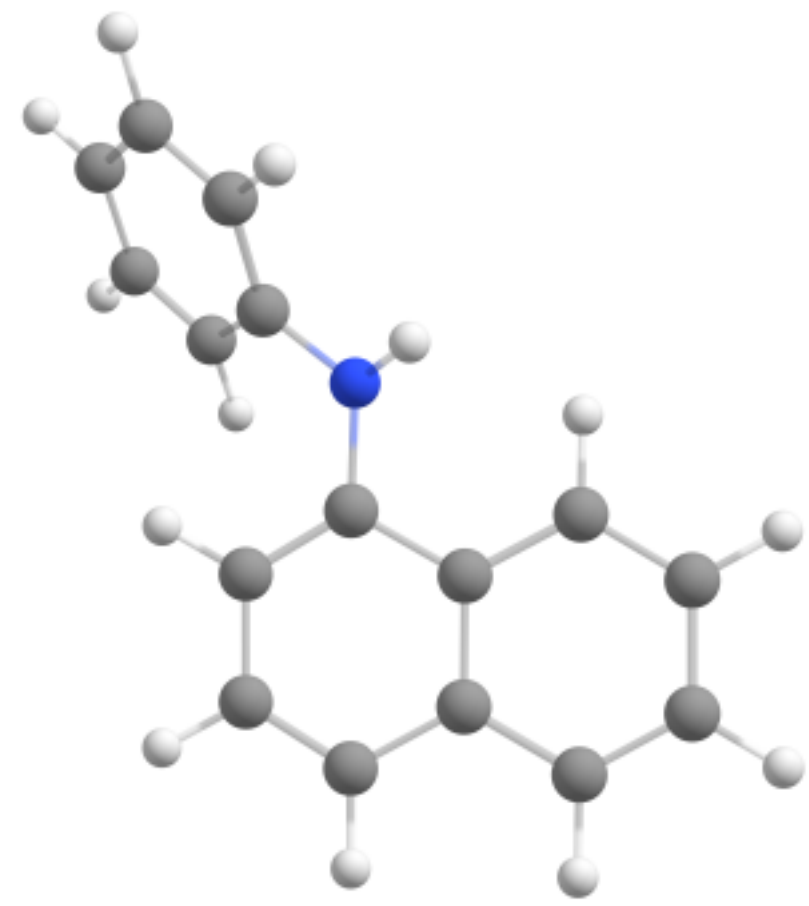

AR Ground State (Gas)

C	-0.034494000	-2.550214000	-0.026137000
C	-0.728041000	-1.316458000	-0.119811000
C	-2.128830000	-1.307182000	0.080762000
C	-2.783457000	-2.522235000	0.407523000
C	-2.087177000	-3.691934000	0.516601000
C	-0.693990000	-3.706465000	0.285734000
C	-0.061276000	-0.083322000	-0.417345000
C	-0.792855000	1.069892000	-0.543390000
C	-2.194828000	1.056041000	-0.378041000
C	-2.852747000	-0.095500000	-0.061888000
N	1.336664000	-0.113774000	-0.547539000
C	2.114173000	0.904815000	-1.113761000
C	3.317349000	1.269771000	-0.510178000
C	4.138521000	2.223679000	-1.094874000
C	3.765338000	2.842327000	-2.279831000
C	2.564850000	2.480803000	-2.881552000
C	1.747303000	1.516293000	-2.314462000
H	1.027717000	-2.584187000	-0.236569000
H	-3.856911000	-2.503775000	0.567621000
H	-2.601681000	-4.613350000	0.766962000
H	-0.148573000	-4.642094000	0.344765000
H	-0.291061000	2.006491000	-0.750126000
H	-2.743133000	1.985960000	-0.485372000
H	-3.927827000	-0.103522000	0.082461000
H	1.807097000	-0.639804000	0.170018000
H	3.608406000	0.801807000	0.425727000
H	5.072075000	2.491044000	-0.611226000
H	4.402213000	3.593691000	-2.732296000
H	2.265475000	2.946009000	-3.814909000
H	0.827359000	1.222044000	-2.805116000

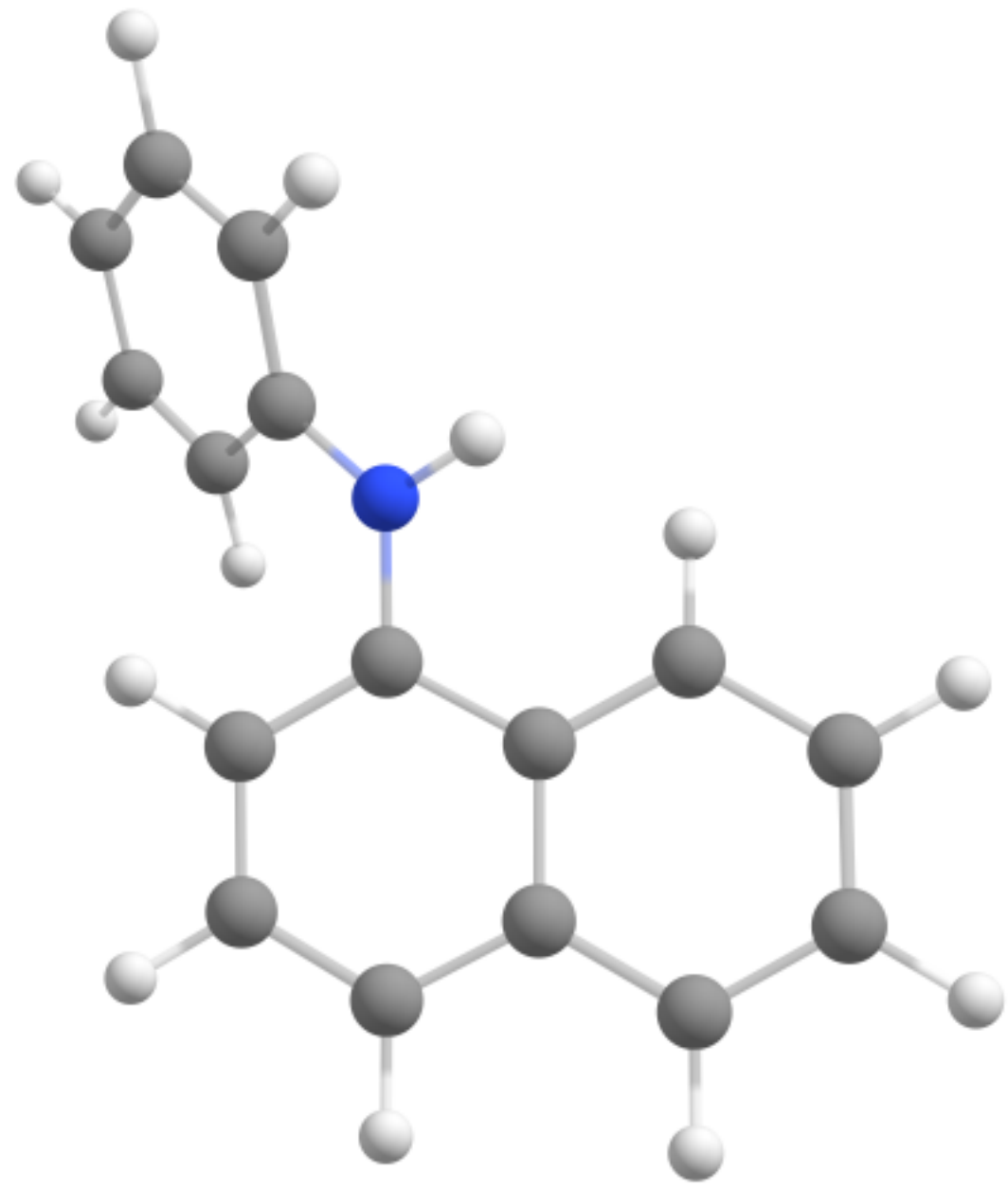

AR S_1 (Gas)

C	0.014459000	-2.484486000	-0.027219000
C	-0.707347000	-1.288748000	-0.124711000
C	-2.130844000	-1.314006000	0.035237000
C	-2.758558000	-2.534780000	0.336322000
C	-2.010340000	-3.709994000	0.477112000
C	-0.642124000	-3.689555000	0.294408000
C	-0.090458000	-0.024184000	-0.382924000
C	-0.851200000	1.177367000	-0.532728000
C	-2.217757000	1.108638000	-0.409909000
C	-2.851280000	-0.111938000	-0.140543000
N	1.275363000	0.042018000	-0.342669000
C	2.086252000	0.942211000	-1.010128000
C	3.361801000	1.228104000	-0.496349000
C	4.187274000	2.115531000	-1.155628000
C	3.761203000	2.738836000	-2.330466000
C	2.505886000	2.442250000	-2.847878000
C	1.666078000	1.544663000	-2.208797000
H	1.073631000	-2.512408000	-0.257065000
H	-3.836908000	-2.554881000	0.452493000
H	-2.511871000	-4.640801000	0.718606000
H	-0.064503000	-4.602767000	0.386157000
H	-0.338960000	2.122849000	-0.649106000
H	-2.811789000	2.012171000	-0.490638000
H	-3.932024000	-0.139866000	-0.043181000
H	1.737782000	-0.638360000	0.251436000
H	3.681840000	0.759924000	0.429273000
H	5.169490000	2.333434000	-0.751089000
H	4.411476000	3.438617000	-2.842467000
H	2.181031000	2.902243000	-3.774875000
H	0.710056000	1.274768000	-2.638926000

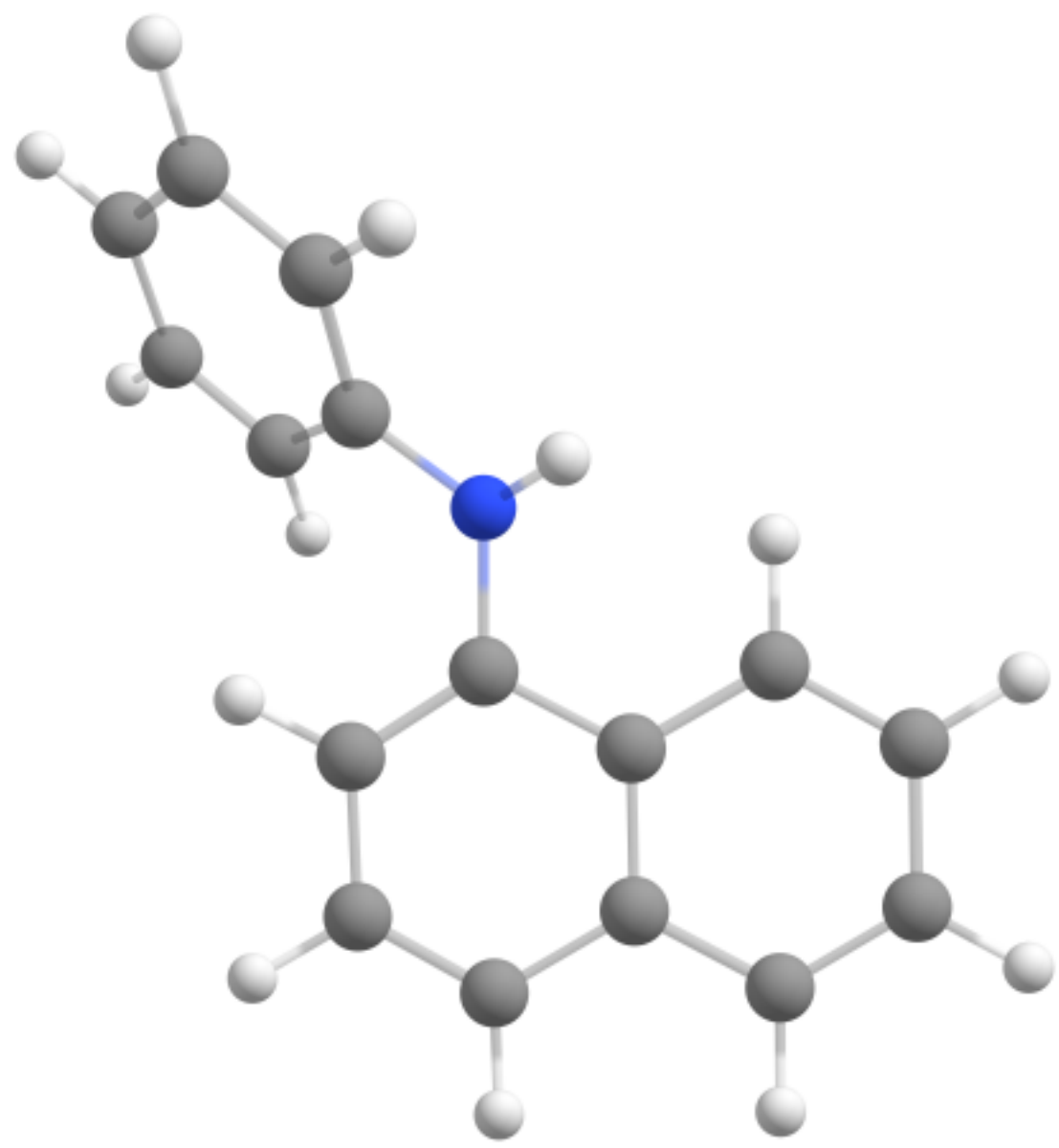

AR Ground State (CPCM: Hexane)

C	-0.033233000	-2.551332000	-0.016049000
C	-0.727758000	-1.318212000	-0.119123000
C	-2.129220000	-1.308987000	0.079921000
C	-2.784867000	-2.523201000	0.410531000
C	-2.087974000	-3.692583000	0.525728000
C	-0.693375000	-3.706887000	0.299566000
C	-0.061052000	-0.085709000	-0.424788000
C	-0.794885000	1.066608000	-0.553893000
C	-2.197099000	1.052941000	-0.386279000
C	-2.855113000	-0.098275000	-0.066773000
N	1.335276000	-0.117428000	-0.561744000
C	2.112564000	0.905344000	-1.121338000
C	3.320450000	1.258083000	-0.518045000
C	4.143327000	2.215876000	-1.095149000
C	3.768256000	2.849726000	-2.272520000
C	2.563964000	2.499262000	-2.874452000
C	1.744052000	1.531486000	-2.314544000
H	1.031065000	-2.586305000	-0.215387000
H	-3.858693000	-2.503613000	0.568510000
H	-2.602378000	-4.613431000	0.778862000
H	-0.147027000	-4.641519000	0.367014000
H	-0.296502000	2.003577000	-0.766825000
H	-2.745326000	1.982716000	-0.496607000
H	-3.930272000	-0.107536000	0.077638000
H	1.809647000	-0.650725000	0.148744000
H	3.613094000	0.775533000	0.409792000
H	5.080021000	2.473662000	-0.612033000
H	4.406565000	3.603804000	-2.718873000
H	2.262784000	2.976002000	-3.801532000
H	0.821370000	1.247973000	-2.806603000

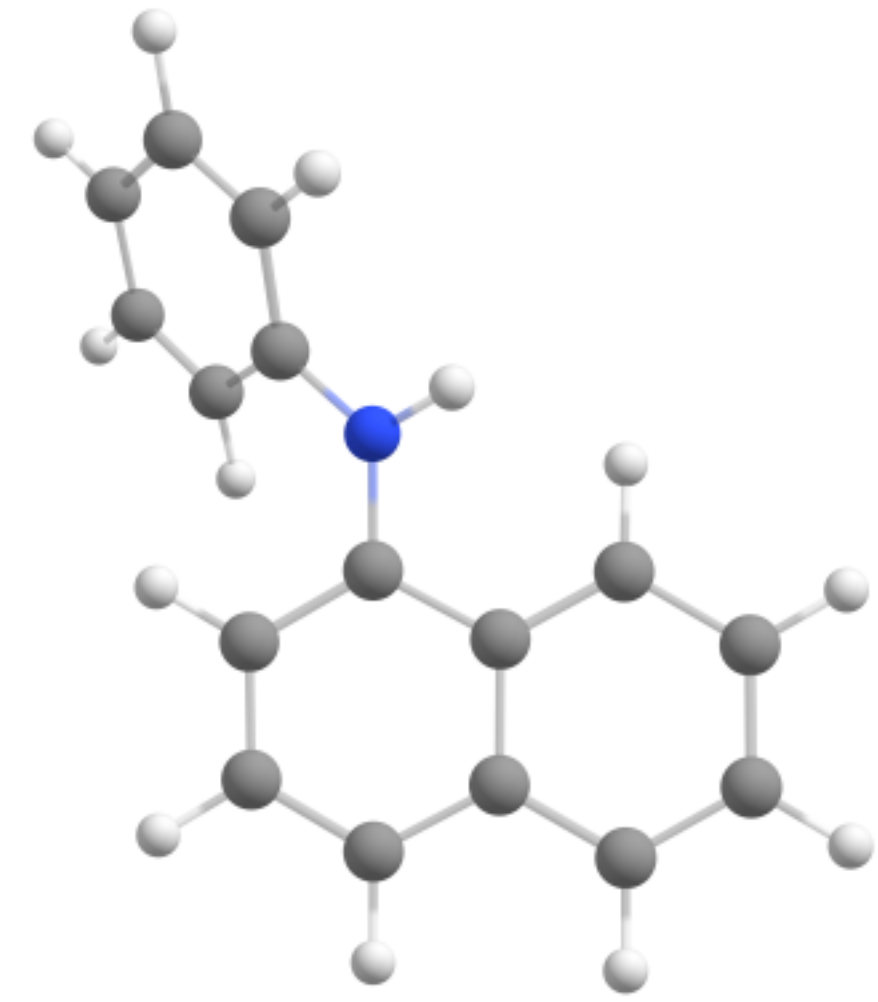

AR S_1 (CPCM: Hexane)

C	0.009410000	-2.489350000	-0.021338000
C	-0.707907000	-1.294249000	-0.120890000
C	-2.132689000	-1.315863000	0.040328000
C	-2.760861000	-2.537081000	0.345359000
C	-2.014711000	-3.714443000	0.486874000
C	-0.647007000	-3.696368000	0.303530000
C	-0.086882000	-0.029049000	-0.390237000
C	-0.855003000	1.171461000	-0.544779000
C	-2.218010000	1.105595000	-0.415876000
C	-2.855168000	-0.116118000	-0.136607000
N	1.275537000	0.037201000	-0.359991000
C	2.085768000	0.944662000	-1.020914000
C	3.362224000	1.222394000	-0.502465000
C	4.190184000	2.115264000	-1.152155000
C	3.767601000	2.750494000	-2.322610000
C	2.511788000	2.460196000	-2.845269000
C	1.669061000	1.558896000	-2.214970000
H	1.071252000	-2.516918000	-0.238762000
H	-3.839302000	-2.554960000	0.463831000
H	-2.518637000	-4.643645000	0.730905000
H	-0.068522000	-4.608939000	0.397537000
H	-0.345907000	2.116803000	-0.675045000
H	-2.811051000	2.009672000	-0.499101000
H	-3.935610000	-0.142949000	-0.034719000
H	1.747128000	-0.644840000	0.224846000
H	3.680193000	0.741751000	0.417386000
H	5.172343000	2.327183000	-0.743861000
H	4.419966000	3.454048000	-2.827082000
H	2.189013000	2.928746000	-3.768867000
H	0.713460000	1.297255000	-2.651305000

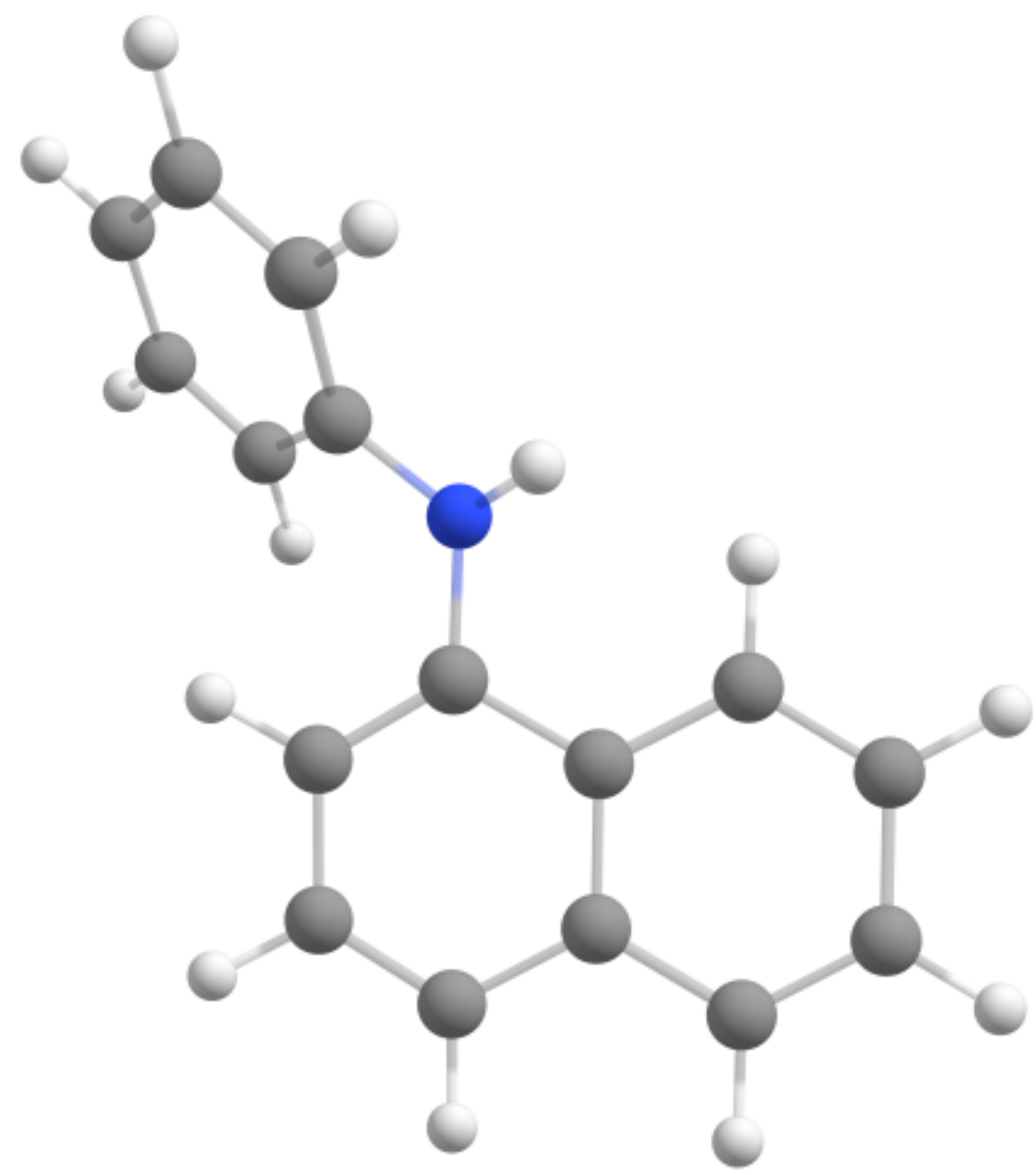

AR Ground State (CPCM: Water)

C	-0.031135000	-2.552269000	-0.001575000
C	-0.727377000	-1.320423000	-0.118036000
C	-2.129949000	-1.311703000	0.078180000
C	-2.786695000	-2.524935000	0.413790000
C	-2.088543000	-3.693489000	0.537964000
C	-0.692010000	-3.706934000	0.319015000
C	-0.060920000	-0.088539000	-0.434417000
C	-0.798272000	1.062306000	-0.568025000
C	-2.200895000	1.048436000	-0.398207000
C	-2.858784000	-0.102515000	-0.074496000
N	1.333073000	-0.121566000	-0.580306000
C	2.110217000	0.906417000	-1.131389000
C	3.325250000	1.241139000	-0.529741000
C	4.150663000	2.203572000	-1.096735000
C	3.772621000	2.858982000	-2.262799000
C	2.562466000	2.525058000	-2.863795000
C	1.739014000	1.553304000	-2.313422000
H	1.036018000	-2.588064000	-0.184959000
H	-3.861142000	-2.504326000	0.568492000
H	-2.602560000	-4.613673000	0.795005000
H	-0.143980000	-4.639748000	0.398948000
H	-0.304728000	1.999816000	-0.789219000
H	-2.749291000	1.977824000	-0.512975000
H	-3.934184000	-0.113755000	0.069389000
H	1.813297000	-0.664212000	0.120584000
H	3.620183000	0.737992000	0.386245000
H	5.092214000	2.447194000	-0.615242000
H	4.413121000	3.616450000	-2.700879000
H	2.258259000	3.018387000	-3.781392000
H	0.811728000	1.286124000	-2.806252000

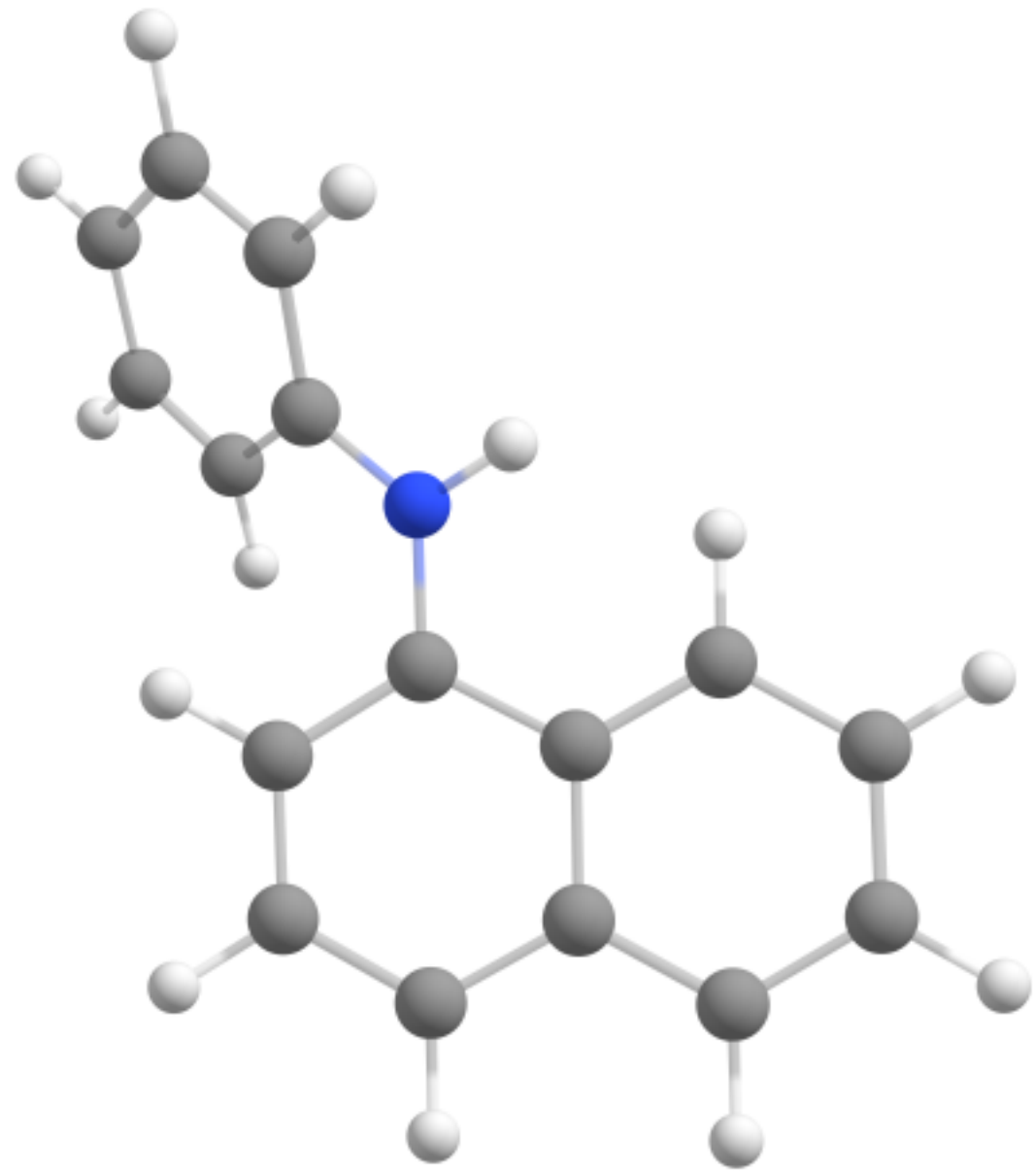

AR S_1 (CPCM: Water)

C	0.005009000	-2.493308000	-0.005923000
C	-0.708319000	-1.300179000	-0.113741000
C	-2.135878000	-1.319224000	0.043067000
C	-2.764190000	-2.542033000	0.351174000
C	-2.019152000	-3.720837000	0.498287000
C	-0.651326000	-3.703208000	0.320768000
C	-0.082059000	-0.033596000	-0.397070000
C	-0.860859000	1.164854000	-0.557818000
C	-2.219345000	1.101388000	-0.426118000
C	-2.861908000	-0.123437000	-0.138450000
N	1.275567000	0.033844000	-0.379452000
C	2.084421000	0.949369000	-1.033636000
C	3.365496000	1.209717000	-0.514953000
C	4.197016000	2.108137000	-1.153638000
C	3.775835000	2.763464000	-2.314278000
C	2.515979000	2.487673000	-2.838551000
C	1.668932000	1.583320000	-2.217896000
H	1.071203000	-2.519109000	-0.201524000
H	-3.843443000	-2.558862000	0.467459000
H	-2.525592000	-4.648708000	0.744090000
H	-0.070204000	-4.613501000	0.422124000
H	-0.356423000	2.110757000	-0.701128000
H	-2.811383000	2.006035000	-0.512741000
H	-3.942523000	-0.149741000	-0.034888000
H	1.760335000	-0.650083000	0.192969000
H	3.683030000	0.708039000	0.393635000
H	5.181964000	2.306930000	-0.744881000
H	4.431277000	3.470629000	-2.810203000
H	2.193404000	2.970919000	-3.754859000
H	0.710794000	1.337601000	-2.658075000

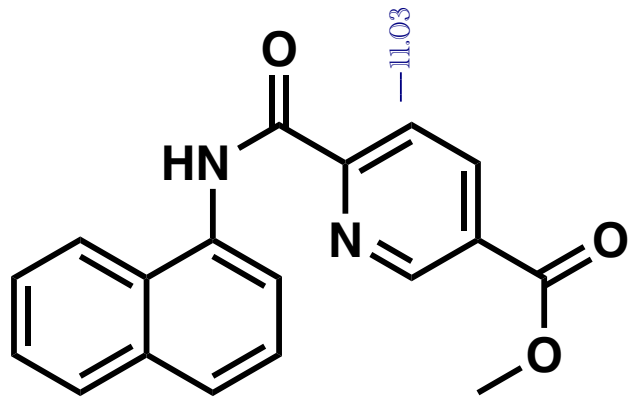

AM-01

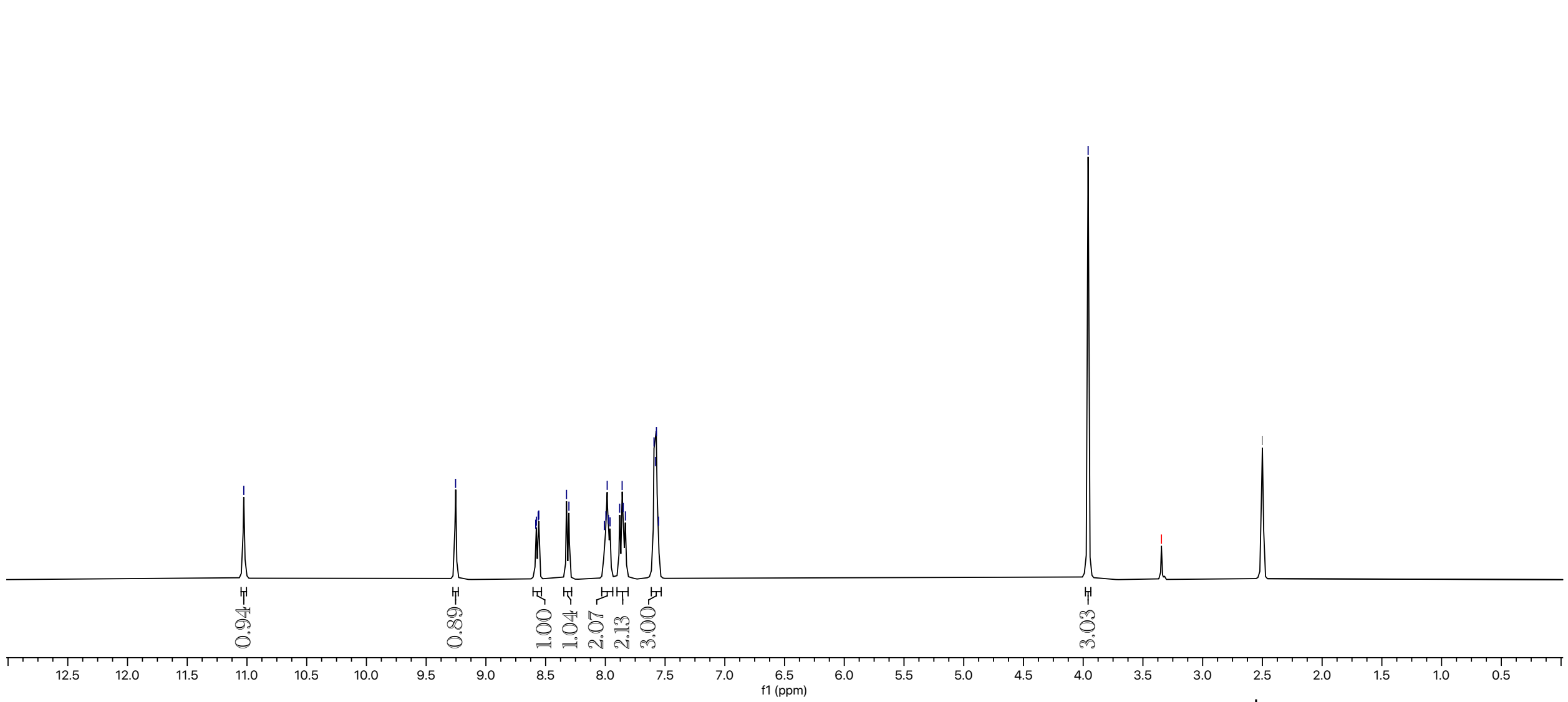

11.03
9.25
8.58
8.58
8.56
8.56
8.33
8.30
8.01
8.00
7.98
7.97
7.96
7.88
7.86
7.85
7.83
7.59
7.58
7.57
7.55
3.96
3.35 H2O
2.50 DMSO d6
0.94
0.89
1.00
1.04
2.07
2.13
3.00
3.03
f1 (ppm)

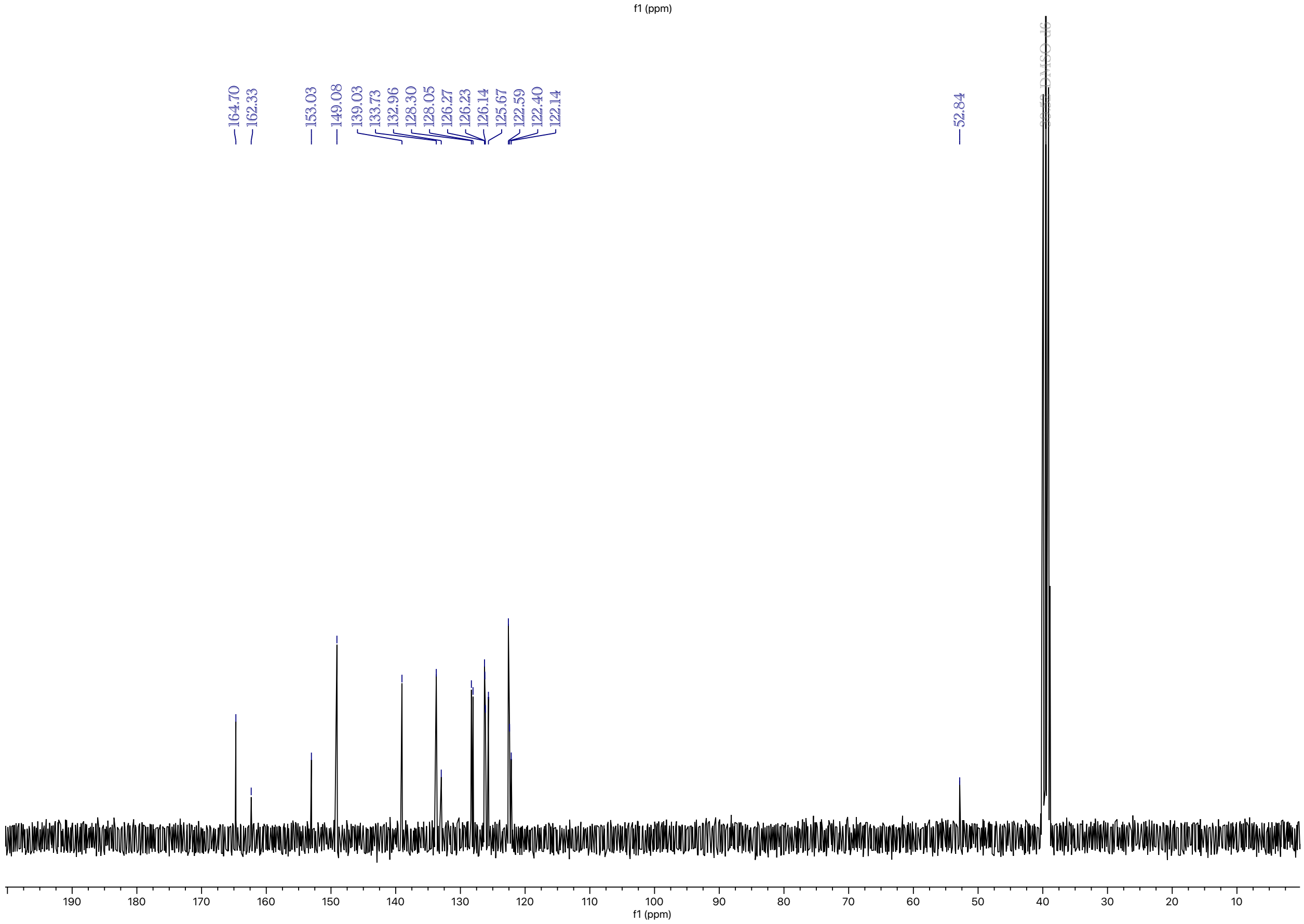

164.70
162.33
153.03
149.08
139.03
133.73
132.96
128.30
128.05
126.27
126.23
126.14
125.67
122.59
122.40
122.14
52.84
DMSO d6
f1 (ppm)

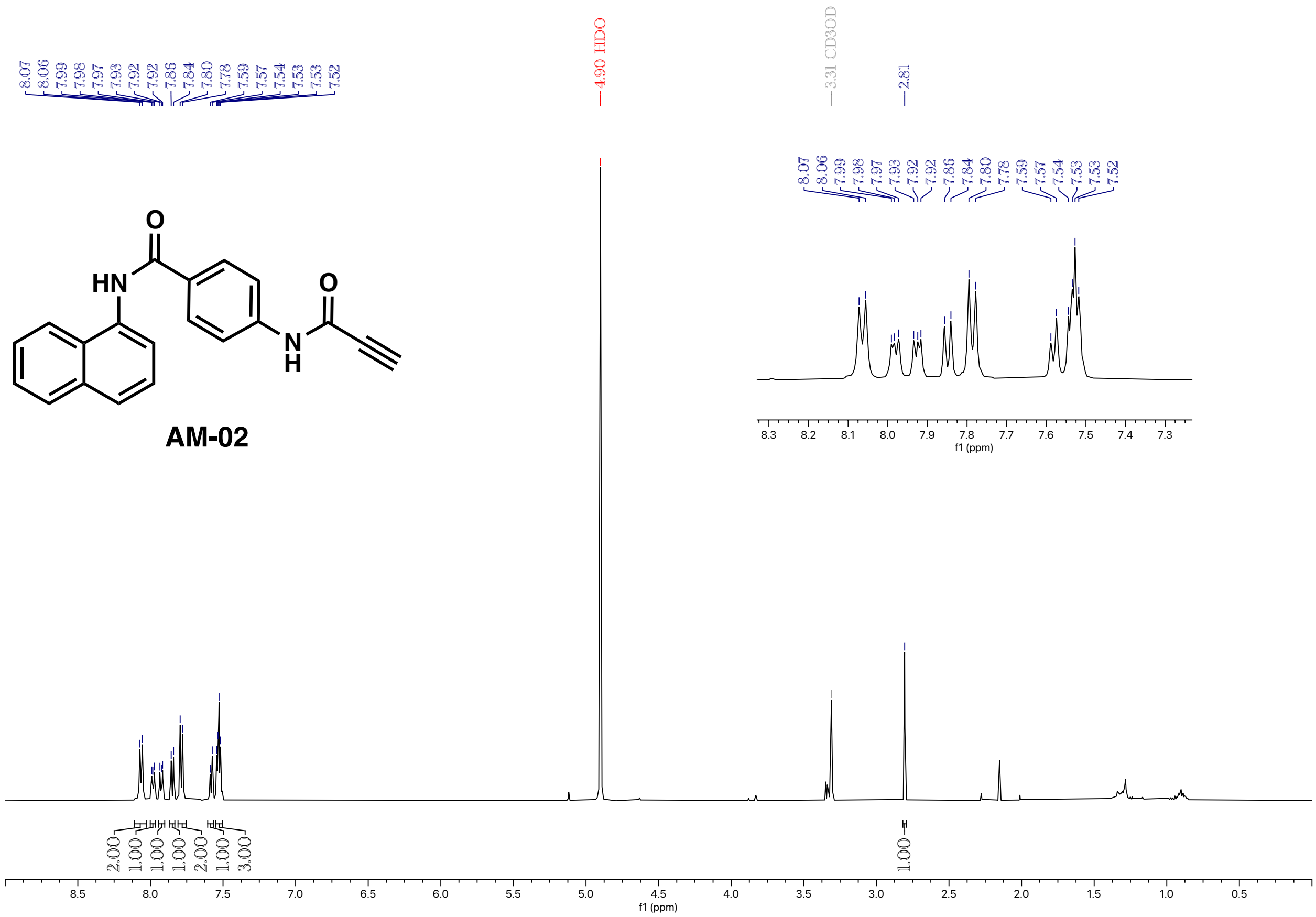
O
HN
O
N
H
8.07 8.06 7.99 7.98 7.97 7.93 7.92 7.92 7.86 7.84 7.80 7.78 7.59 7.57 7.54 7.53 7.53 7.52
4.90 HDO
3.31 CD3OD
2.81
2.00 1.00 1.00 1.00 2.00 1.00 3.00
1.00
f1 (ppm)

AM-02

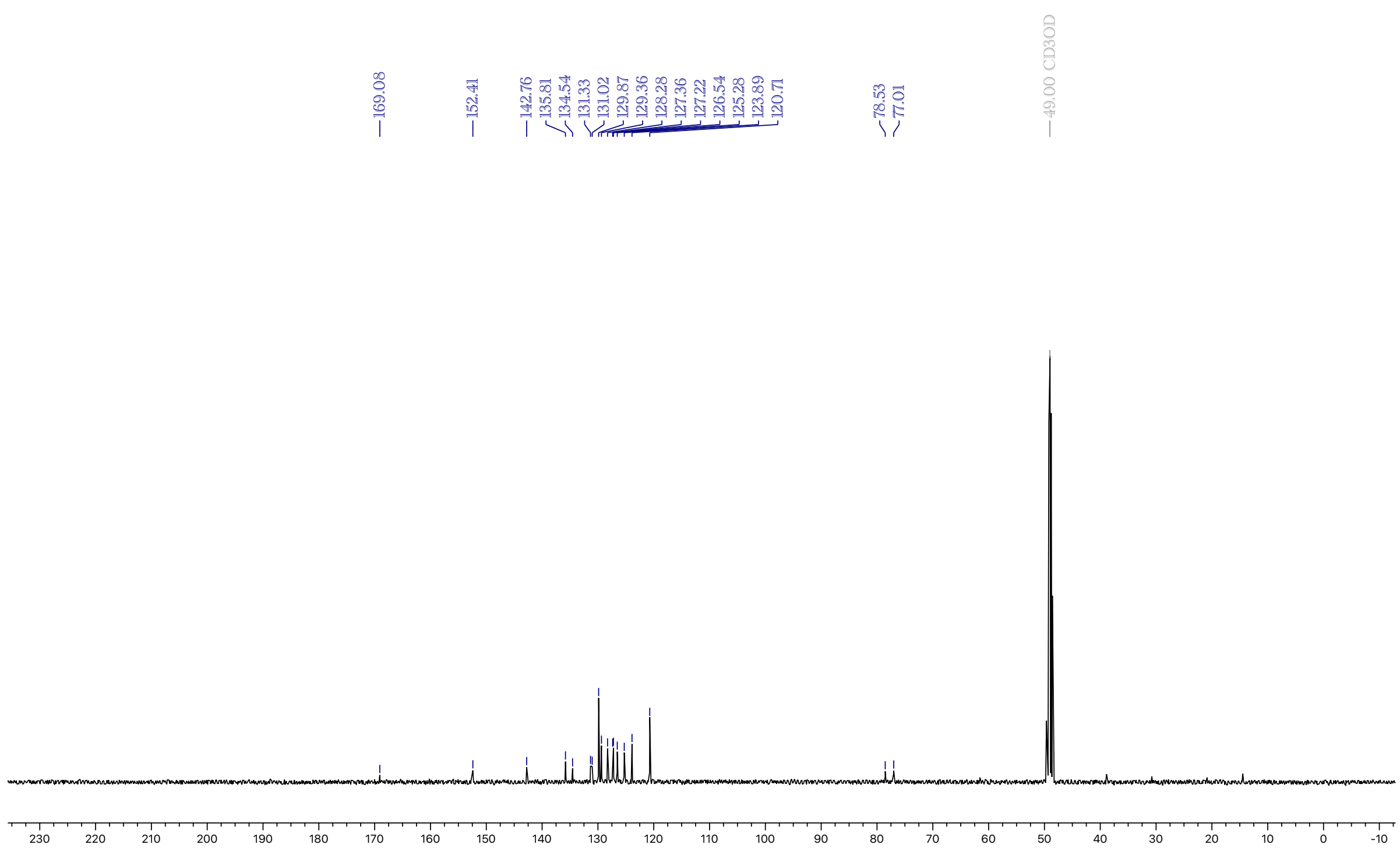
169.08
152.41
142.76 135.81 134.54 131.33 131.02 129.87 129.36 128.28 127.36 127.22 126.54 125.28 123.89 120.71
78.53 77.01
49.00 CD3OD
f1 (ppm)

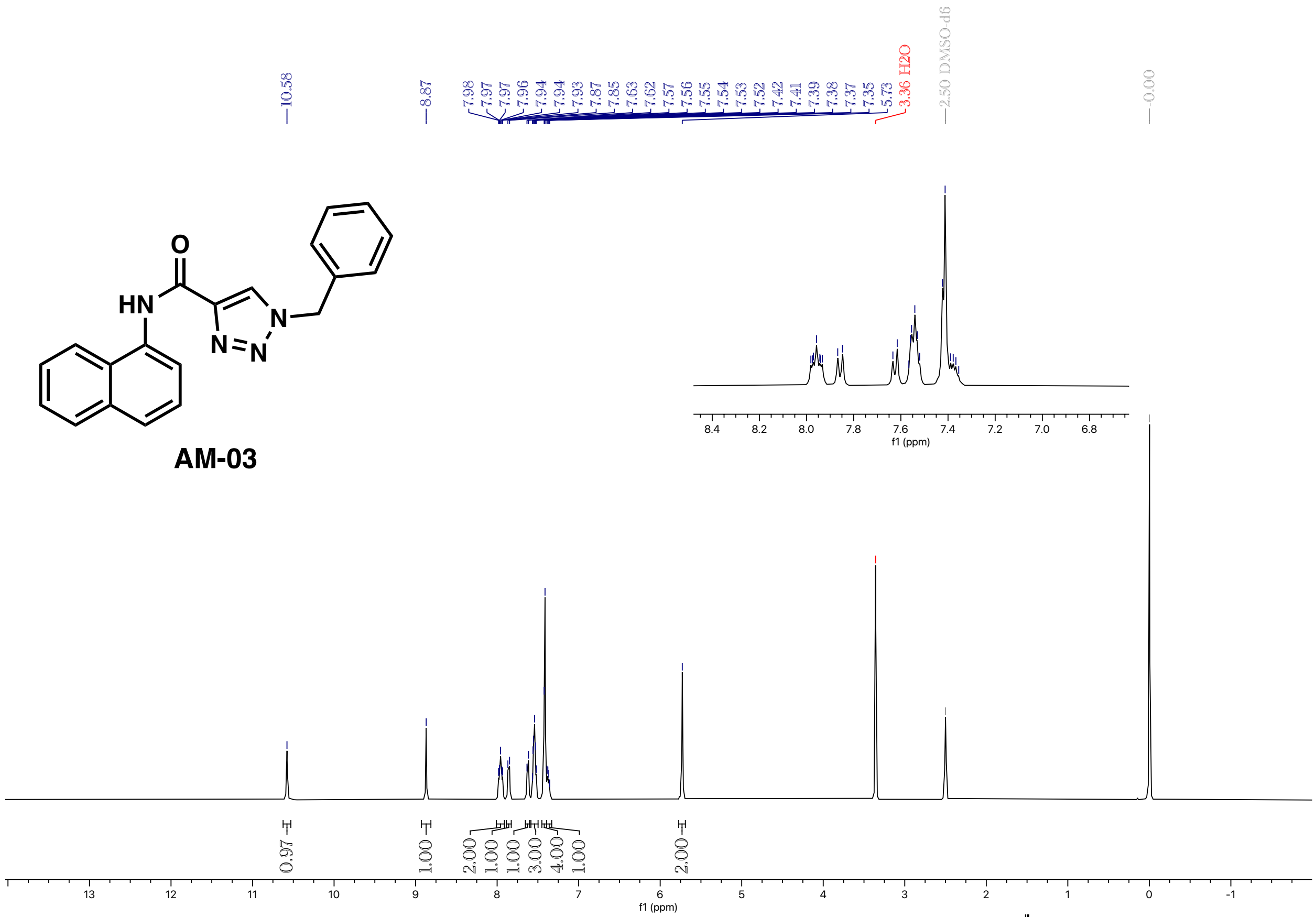
O
HN
N
N
N
AM-03
10.58
8.87
7.98
7.97
7.97
7.96
7.94
7.94
7.93
7.87
7.85
7.63
7.62
7.57
7.56
7.55
7.54
7.53
7.52
7.42
7.41
7.39
7.38
7.37
7.35
5.73
3.36 H2O
2.50 DMSO d6
0.00
0.97
1.00
2.00
1.00
1.00
3.00
4.00
1.00
2.00
f1 (ppm)

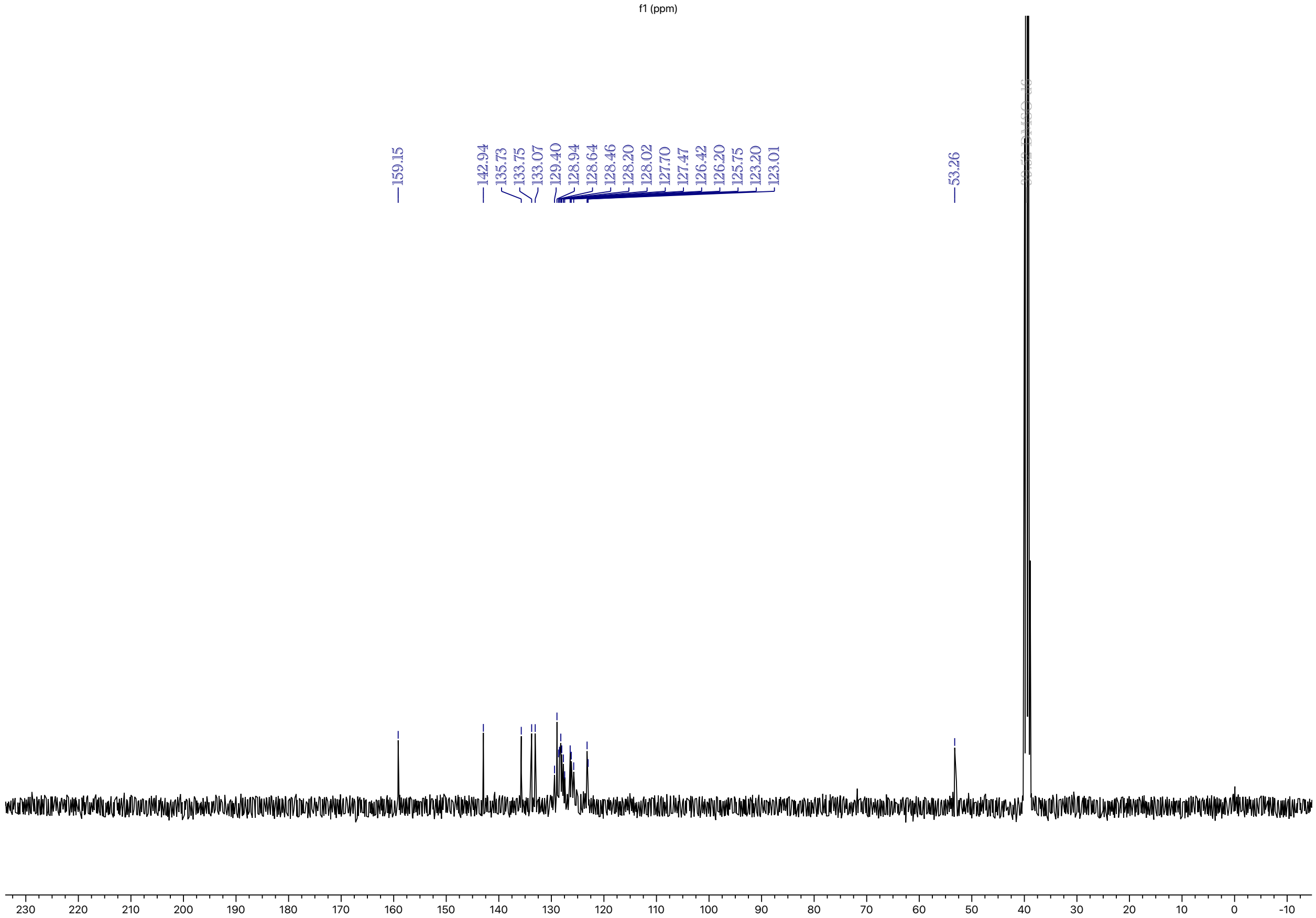
159.15
142.94
135.73
133.75
133.07
129.40
128.94
128.64
128.46
128.20
128.02
127.70
127.47
126.42
126.20
125.75
123.20
123.01
53.26
f1 (ppm)

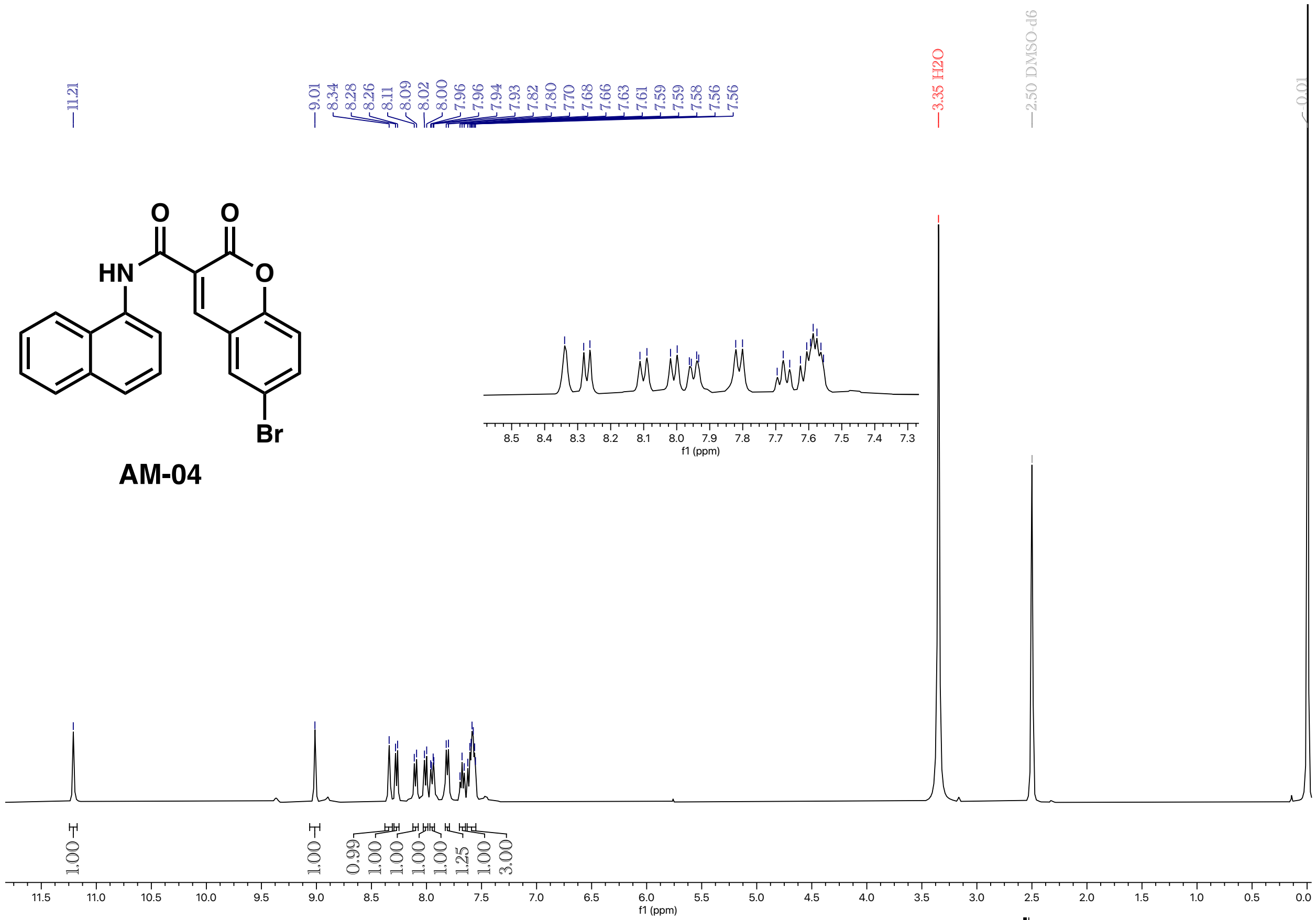

AM-04

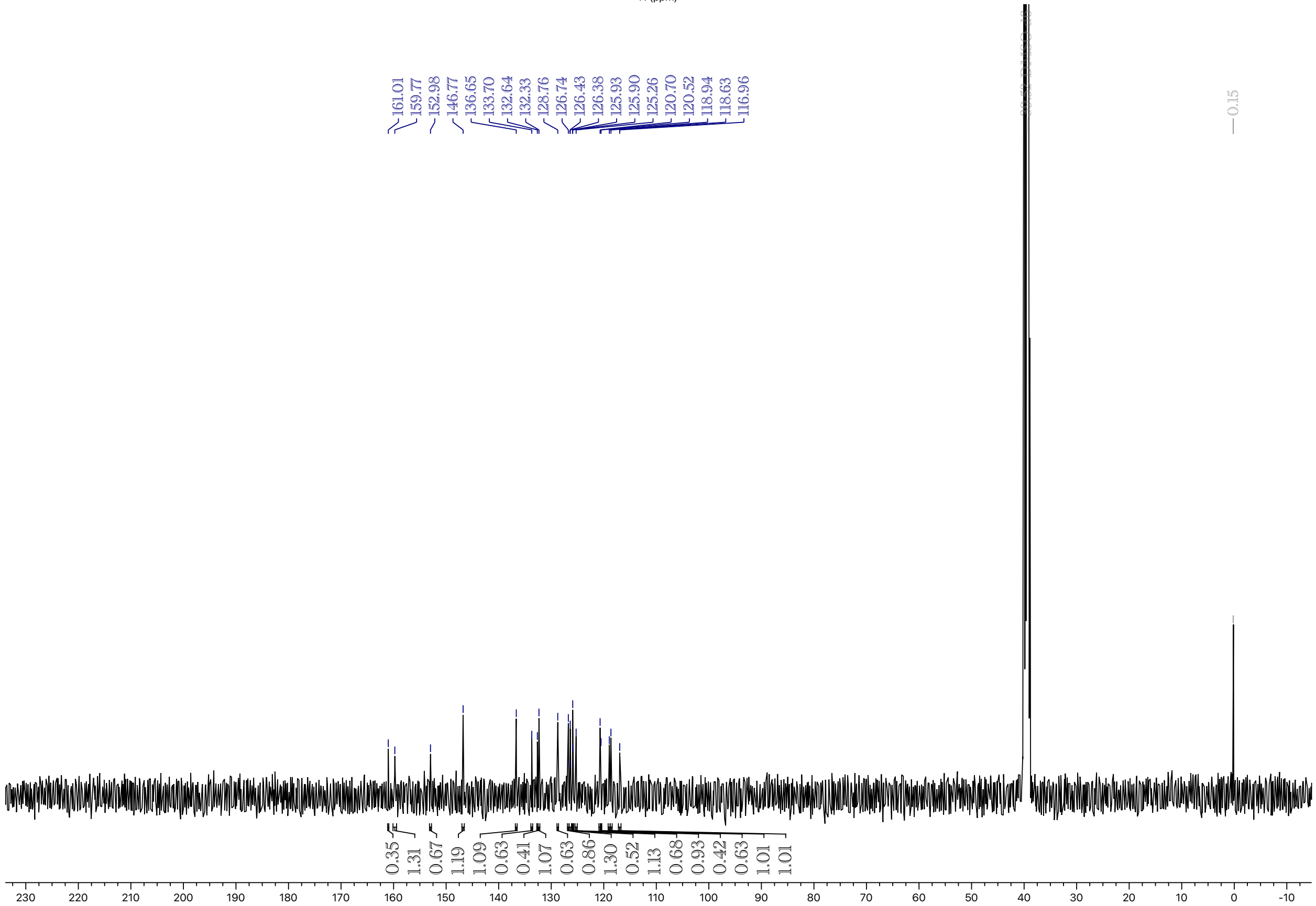

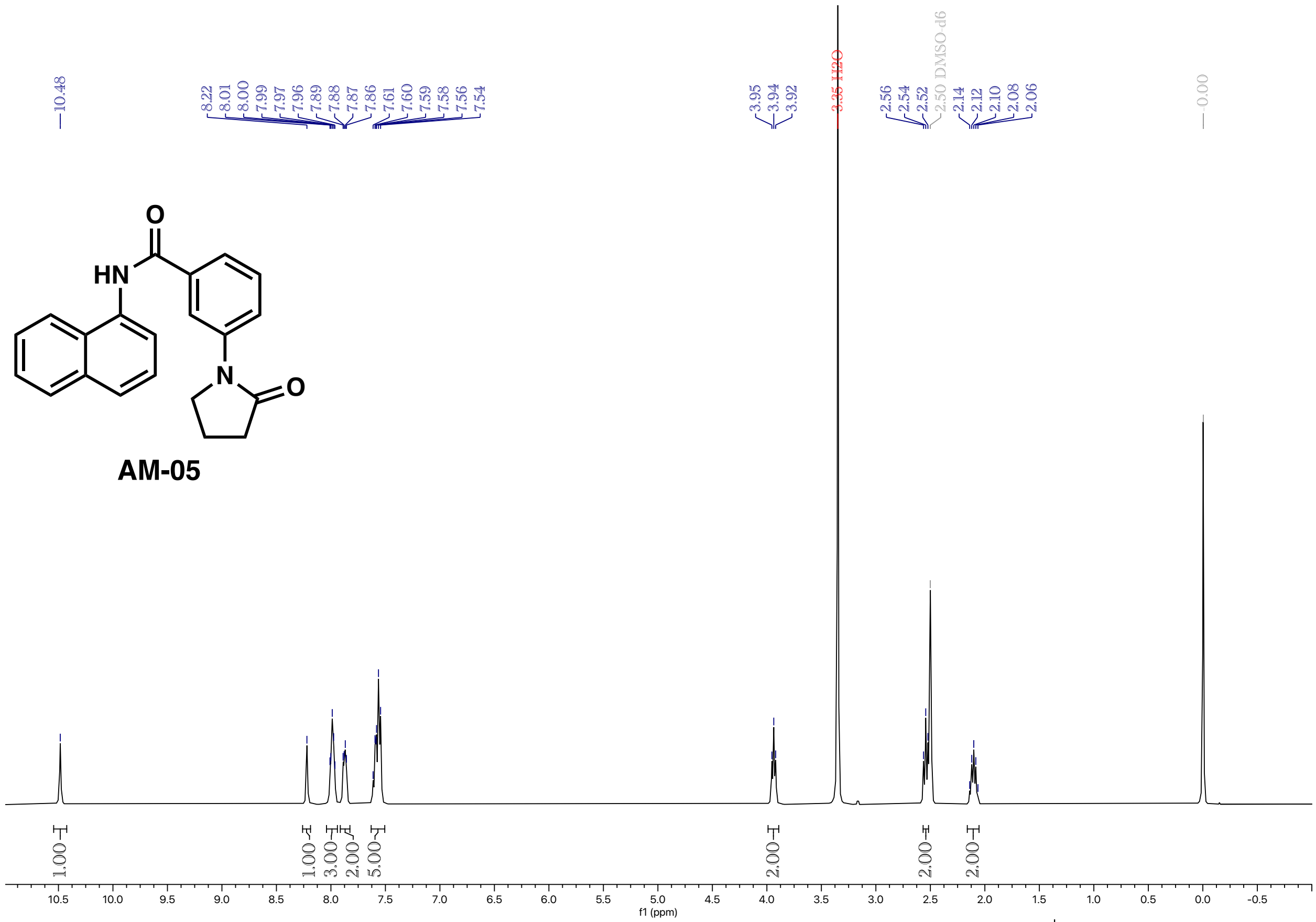

AM-05

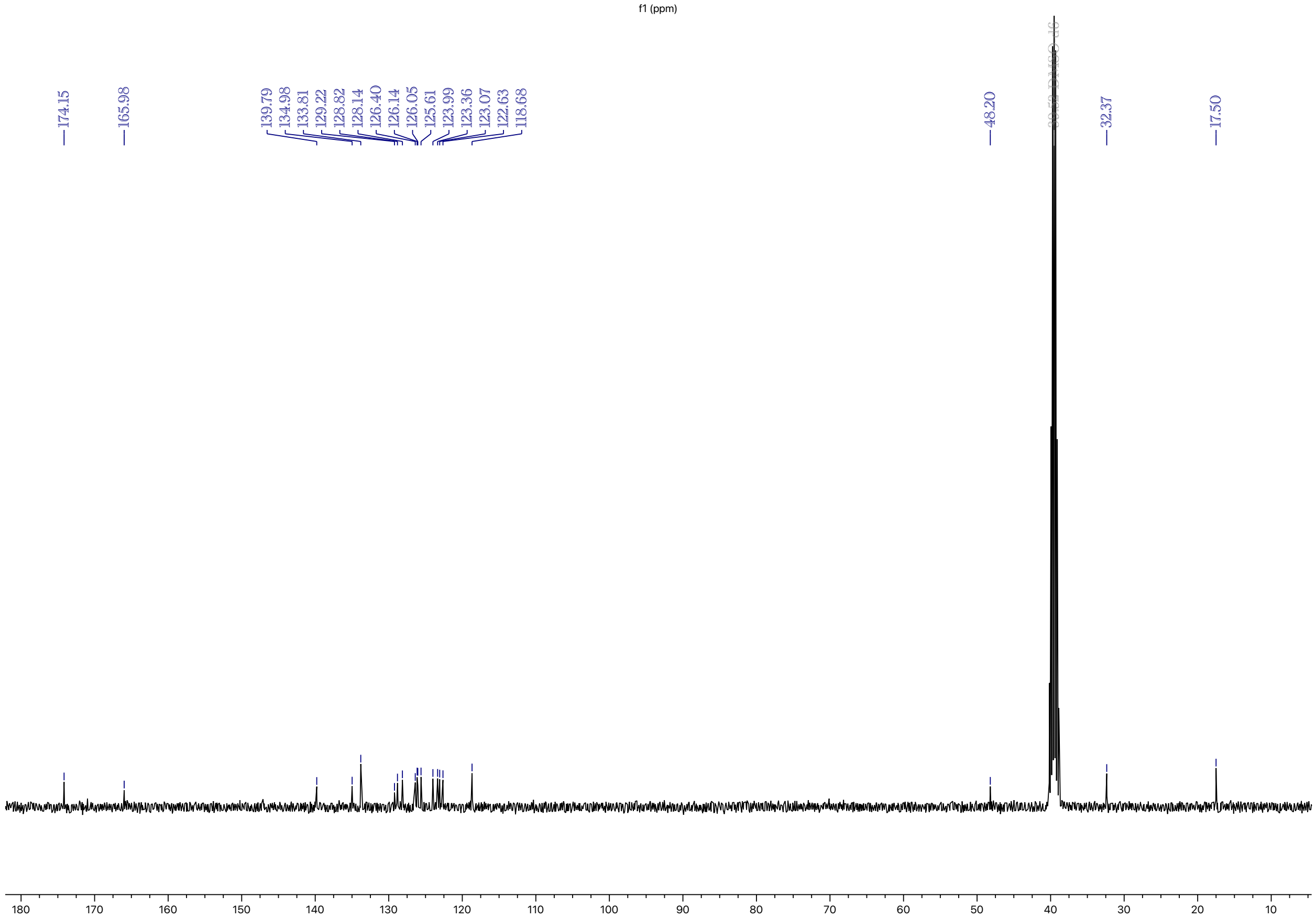

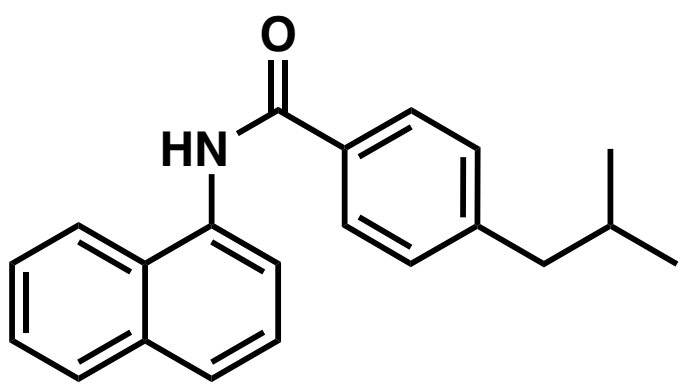
O
HN

AM-06

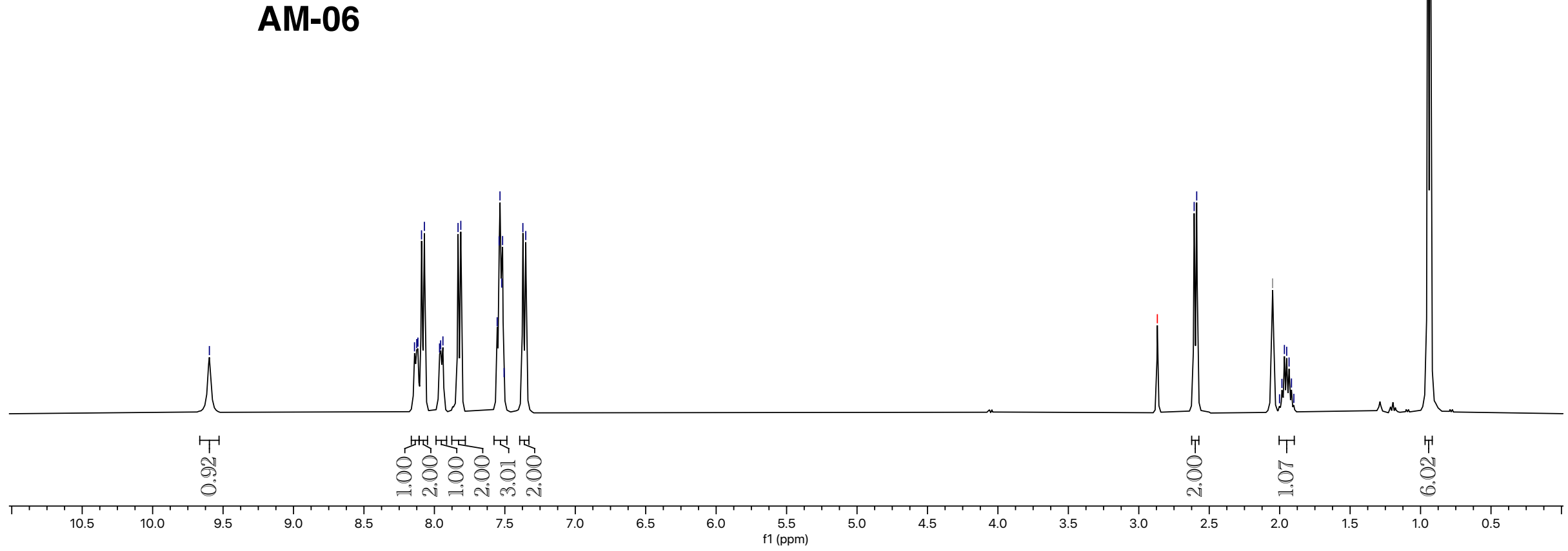
9.60
8.14 8.13 8.12 8.09 8.07 7.96 7.96 7.94 7.83 7.81 7.55 7.54 7.53 7.52 7.52 7.51 7.37 7.35
2.87 H2O
2.61 2.59
2.05 (CD3)2CO
2.00 1.98 1.97 1.95 1.93 1.92 1.90
0.95 0.93
0.92 1.00 2.00 1.00 2.00 3.01 2.00 2.00 1.07 6.02
f1 (ppm)

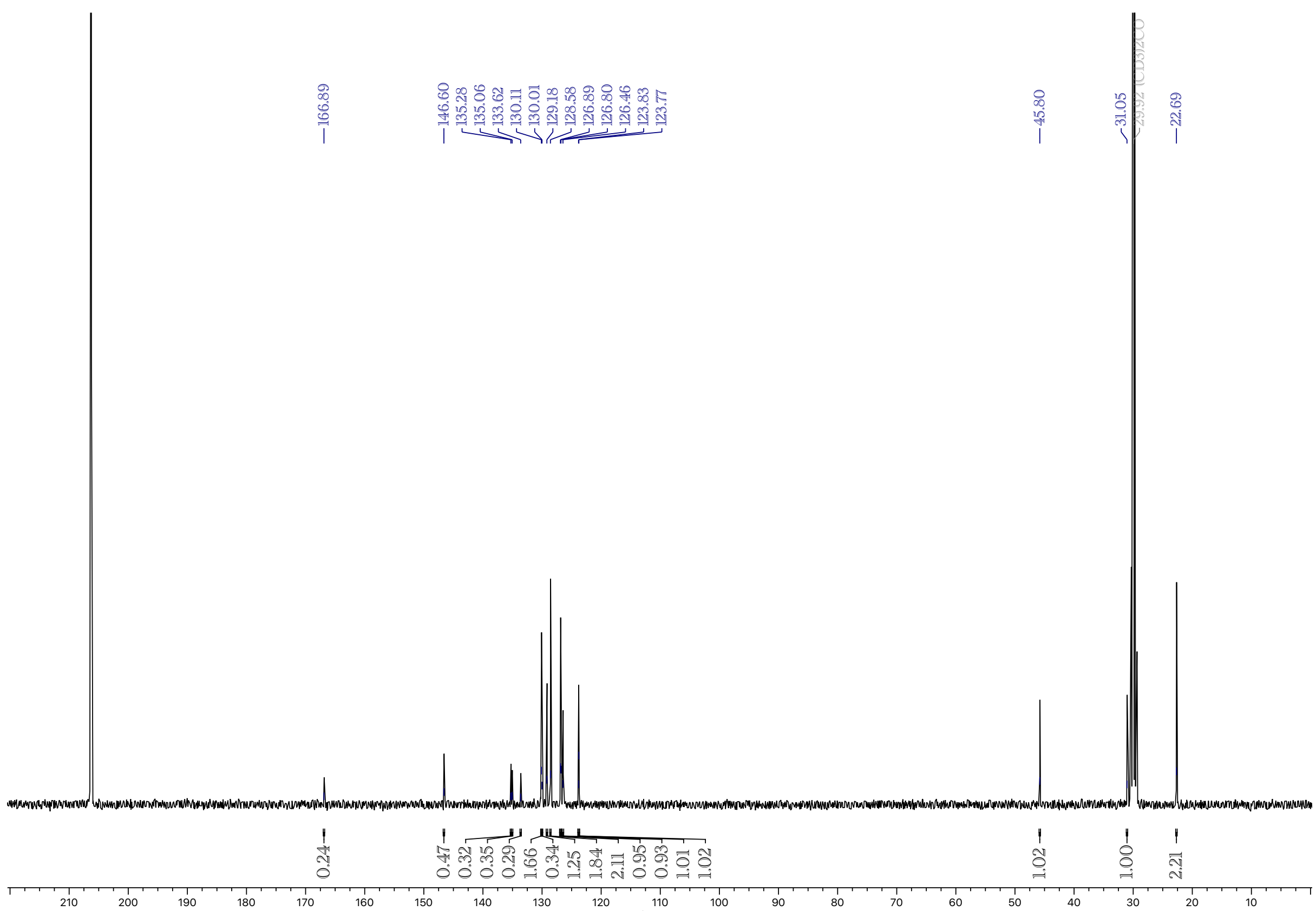
166.89
146.60 135.28 135.06 133.62 130.11 130.01 129.18 128.58 126.89 126.80 126.46 123.83 123.77
45.80
31.05
29.92 (CD3)2CO
22.69
0.24 0.47 0.32 0.35 0.29 1.66 0.34 1.25 1.84 2.11 0.95 0.93 1.01 1.02 1.02 1.00 2.21
f1 (ppm)

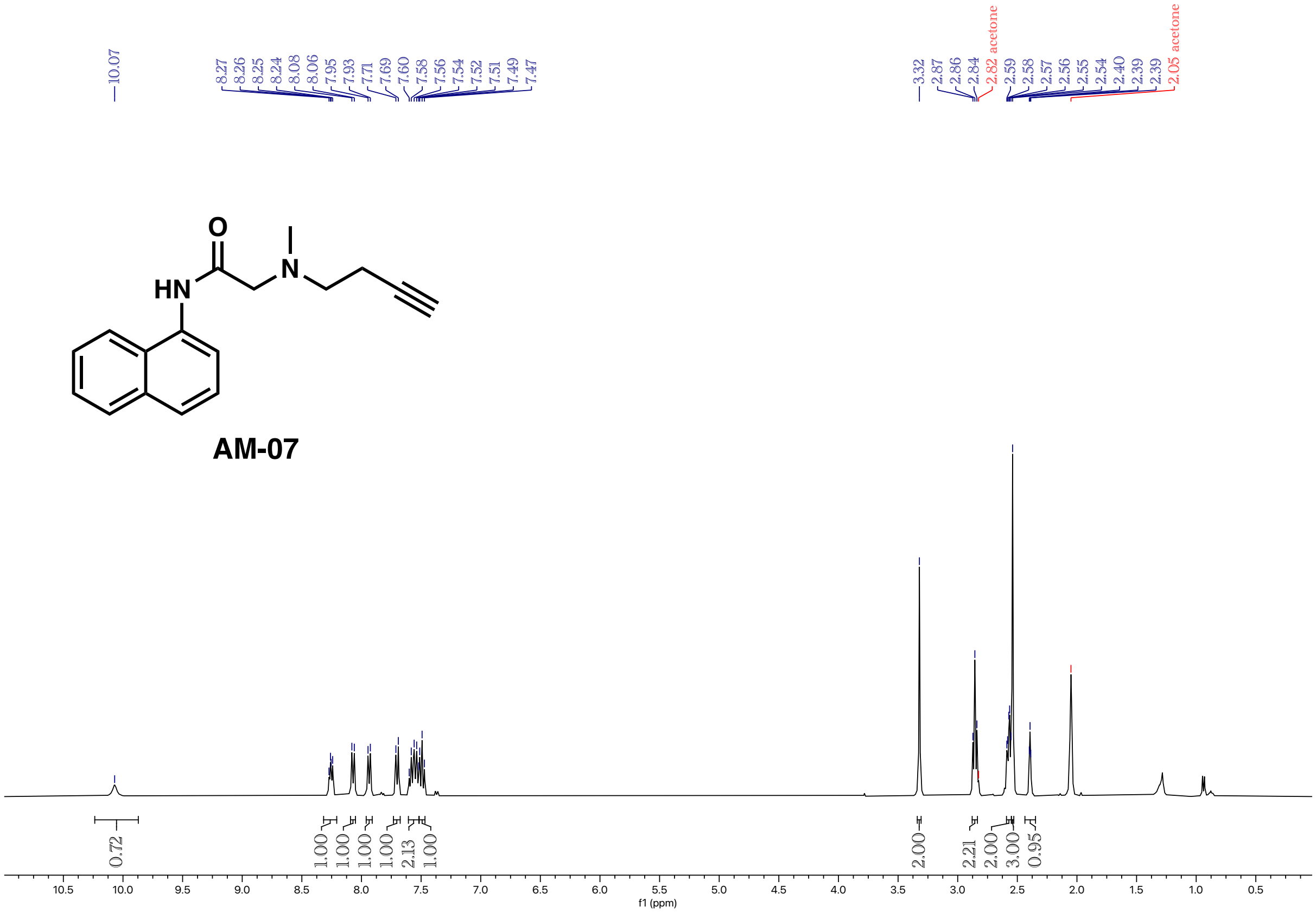
AM-07
10.07
8.27 8.26 8.25 8.24 8.08 8.06 7.95 7.93 7.71 7.69 7.60 7.58 7.56 7.54 7.52 7.51 7.49 7.47
3.32 2.87 2.86 2.84
2.82 acetone
2.59 2.58 2.57 2.56 2.55 2.54 2.40 2.39 2.39
2.05 acetone
0.72 1.00 1.00 1.00 1.00 2.13 1.00 2.00 2.21 2.00 3.00 0.95
10.5 10.0 9.5 9.0 8.5 8.0 7.5 7.0 6.5 6.0 5.5 5.0 4.5 4.0 3.5 3.0 2.5 2.0 1.5 1.0 0.5
f1 (ppm)

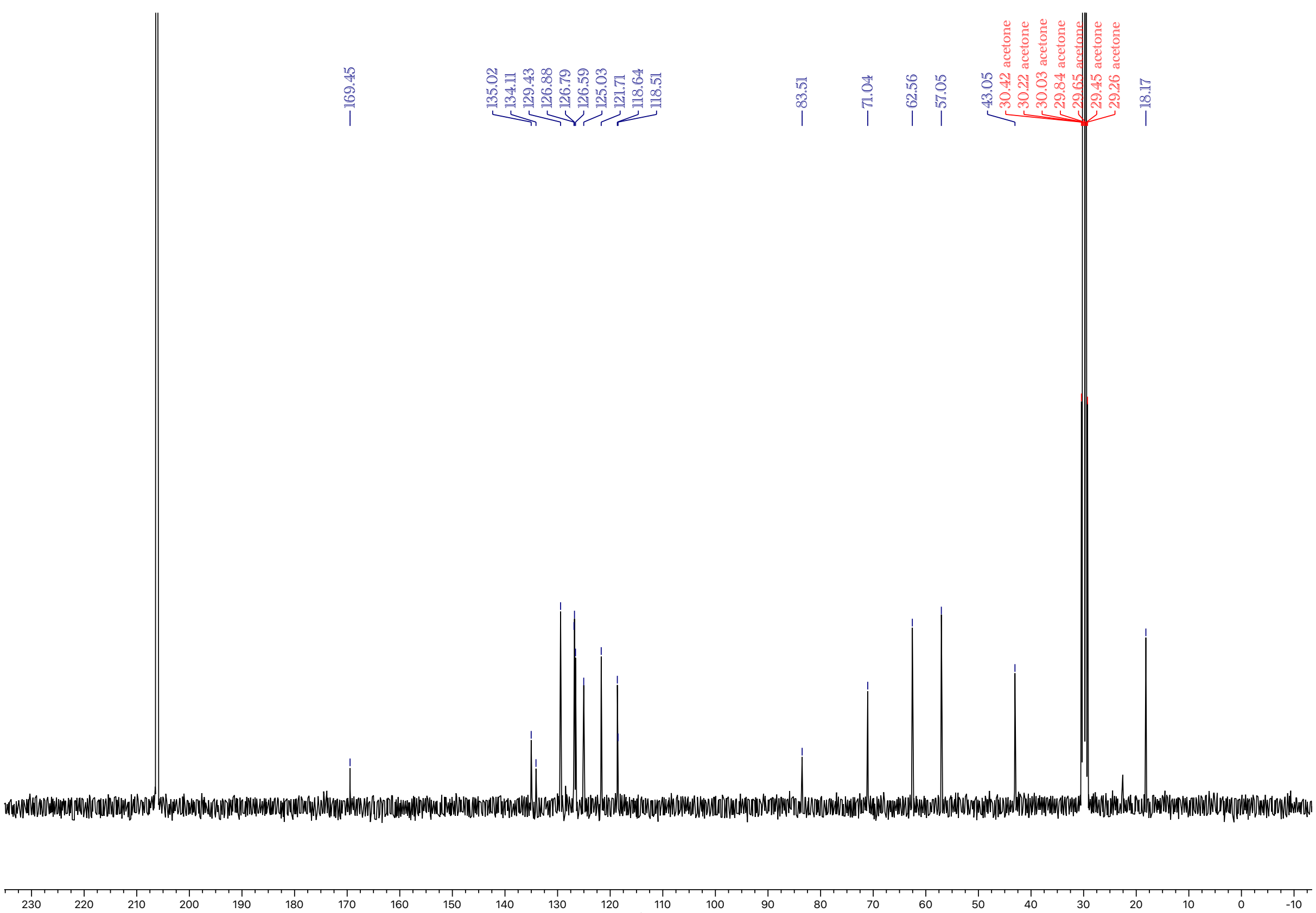
169.45
135.02 134.11 129.43 126.88 126.79 126.59 125.03 121.71 118.64 118.51
83.51
71.04
62.56
57.05
43.05
30.42 acetone
30.22 acetone
30.03 acetone
29.84 acetone
29.65 acetone
29.45 acetone
29.26 acetone
18.17
230 220 210 200 190 180 170 160 150 140 130 120 110 100 90 80 70 60 50 40 30 20 10 0 -10
f1 (ppm)

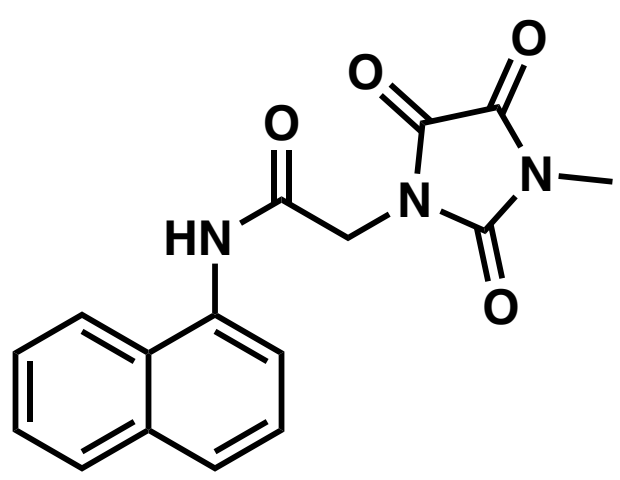

AM-08

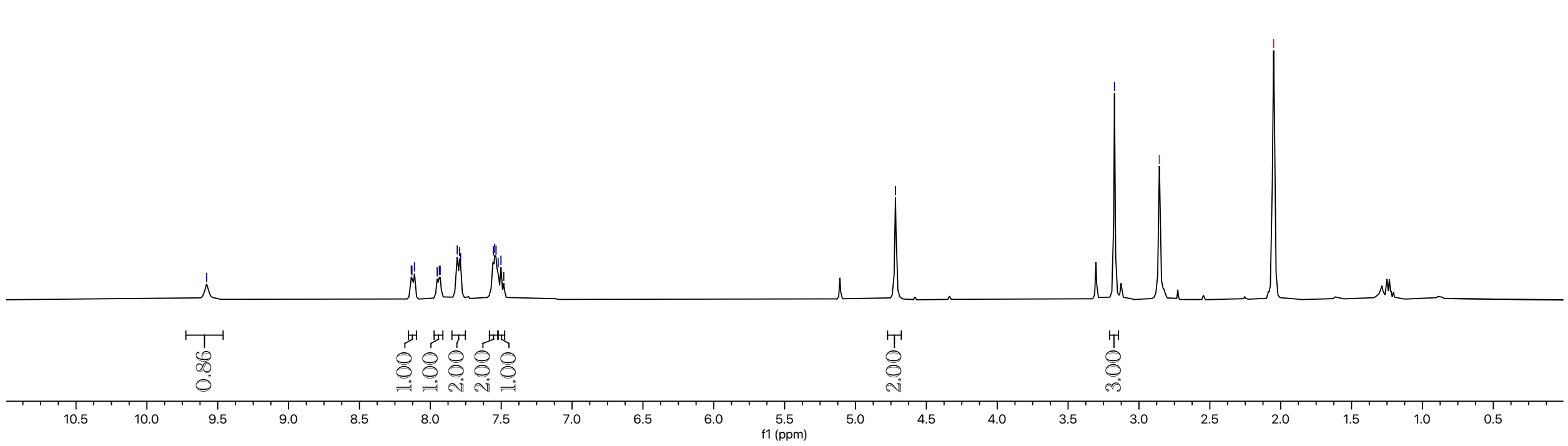

9.58
8.13
8.13
8.11
7.95
7.94
7.93
7.81
7.79
7.79
7.56
7.55
7.54
7.52
7.50
7.48
4.72
3.17
2.86 H2O
2.05 acetone
0.86
1.00
1.00
2.00
2.00
1.00
2.00
3.00
f1 (ppm)

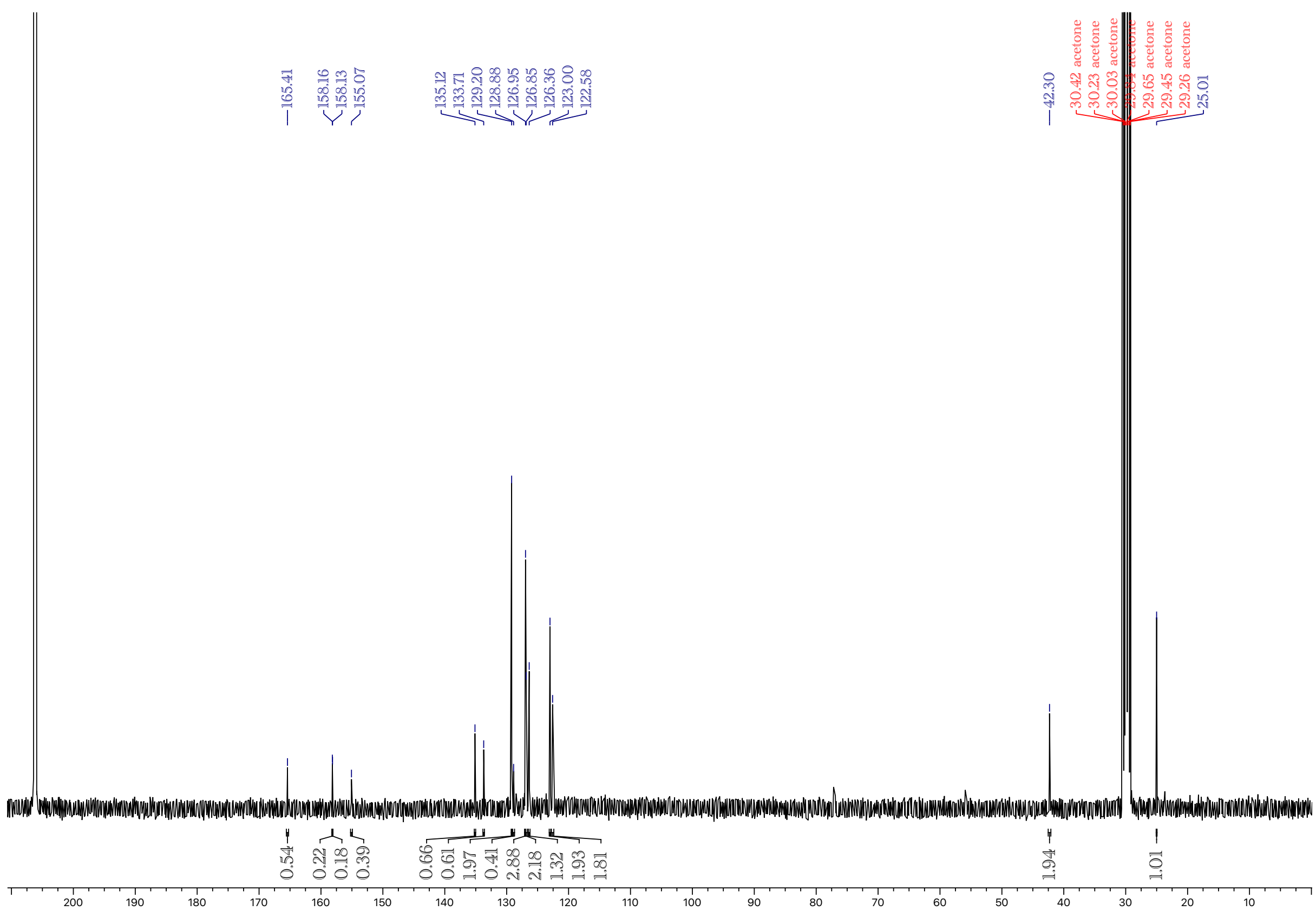

165.41
158.16
158.13
155.07
135.12
133.71
129.20
128.88
126.95
126.85
126.36
123.00
122.58
42.30
30.42 acetone
30.23 acetone
30.03 acetone
29.84 acetone
29.65 acetone
29.45 acetone
29.26 acetone
25.01
0.54
0.22
0.18
0.39
0.66
0.61
1.97
0.41
2.88
2.18
1.32
1.93
1.81
1.94
1.01
f1 (ppm)

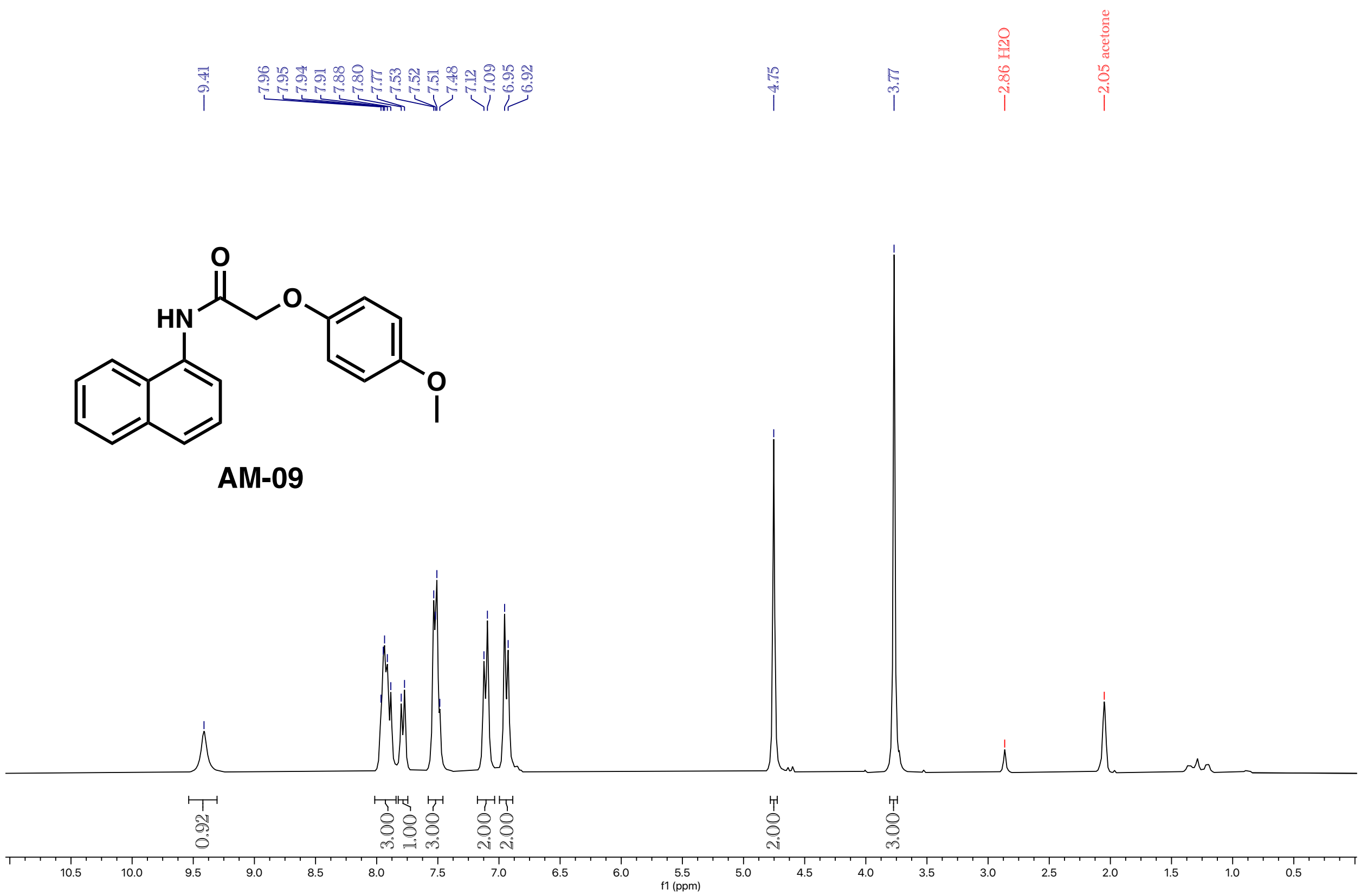

AM-09

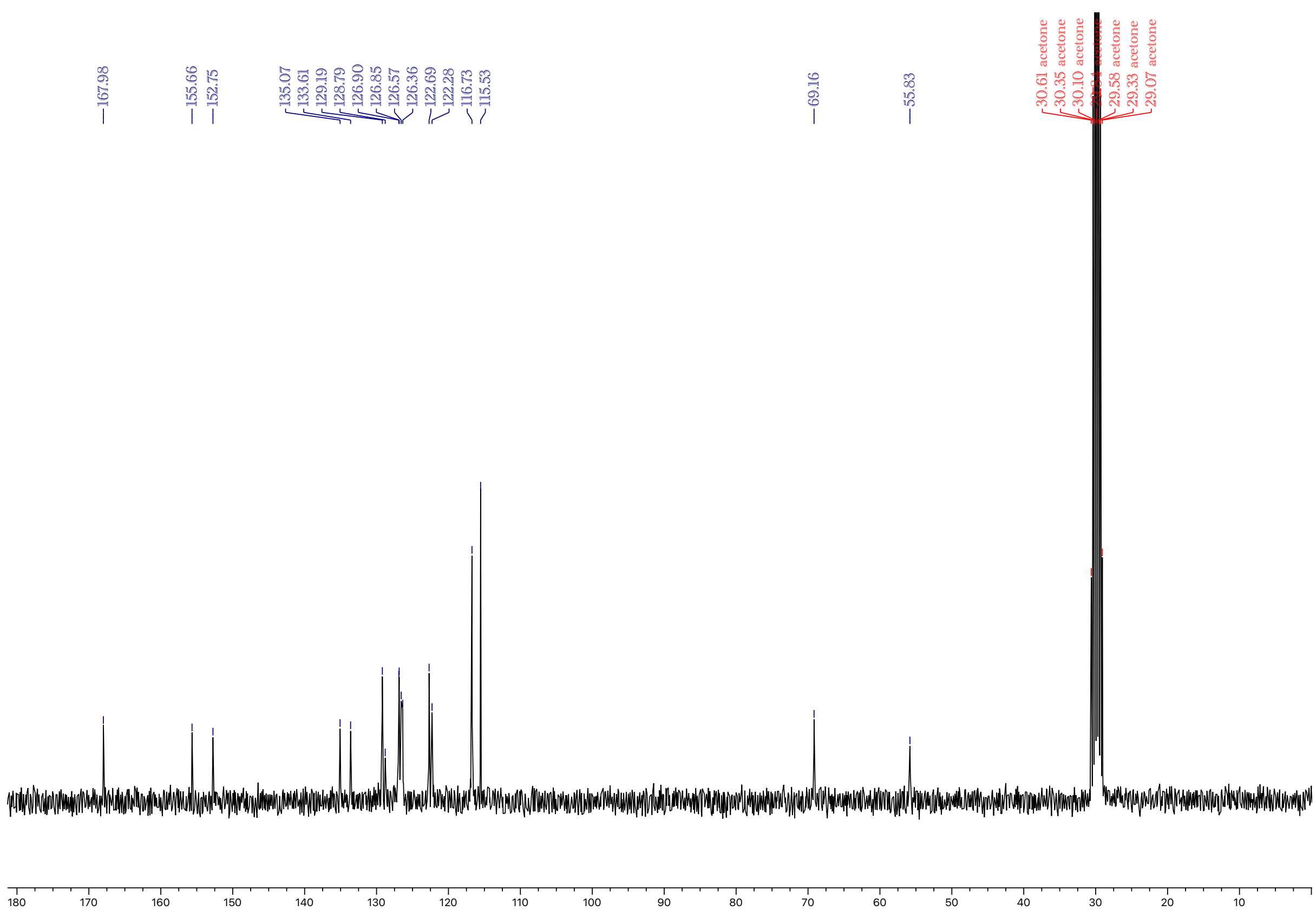

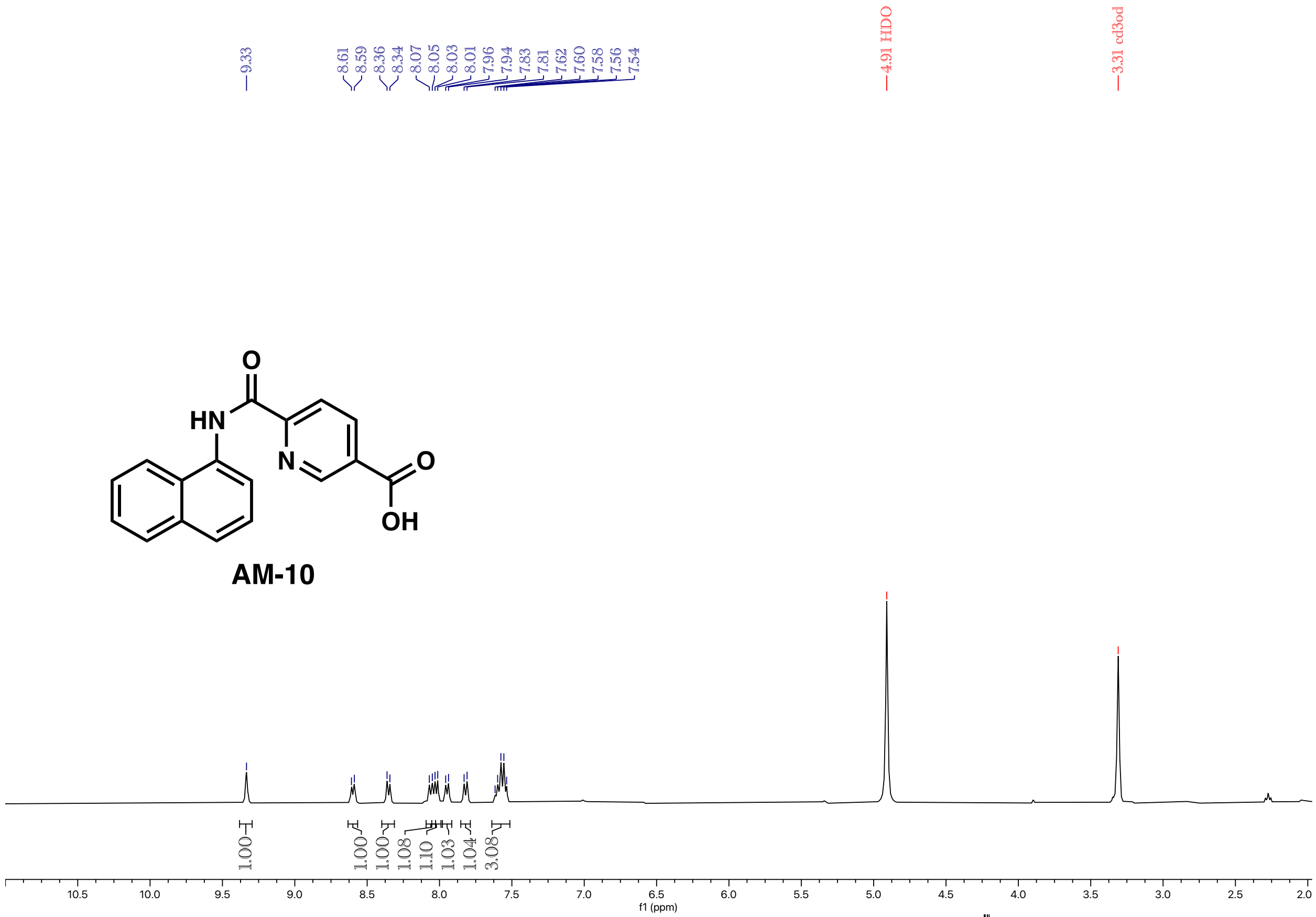
9.33
8.61
8.59
8.36
8.34
8.07
8.05
8.03
8.01
7.96
7.94
7.83
7.81
7.62
7.60
7.58
7.56
7.54
4.91 HDO
3.31 cd3od
O
HN
N
O
OH
AM-10
1.00
1.00
1.00
1.08
1.10
1.03
1.04
3.08
f1 (ppm)

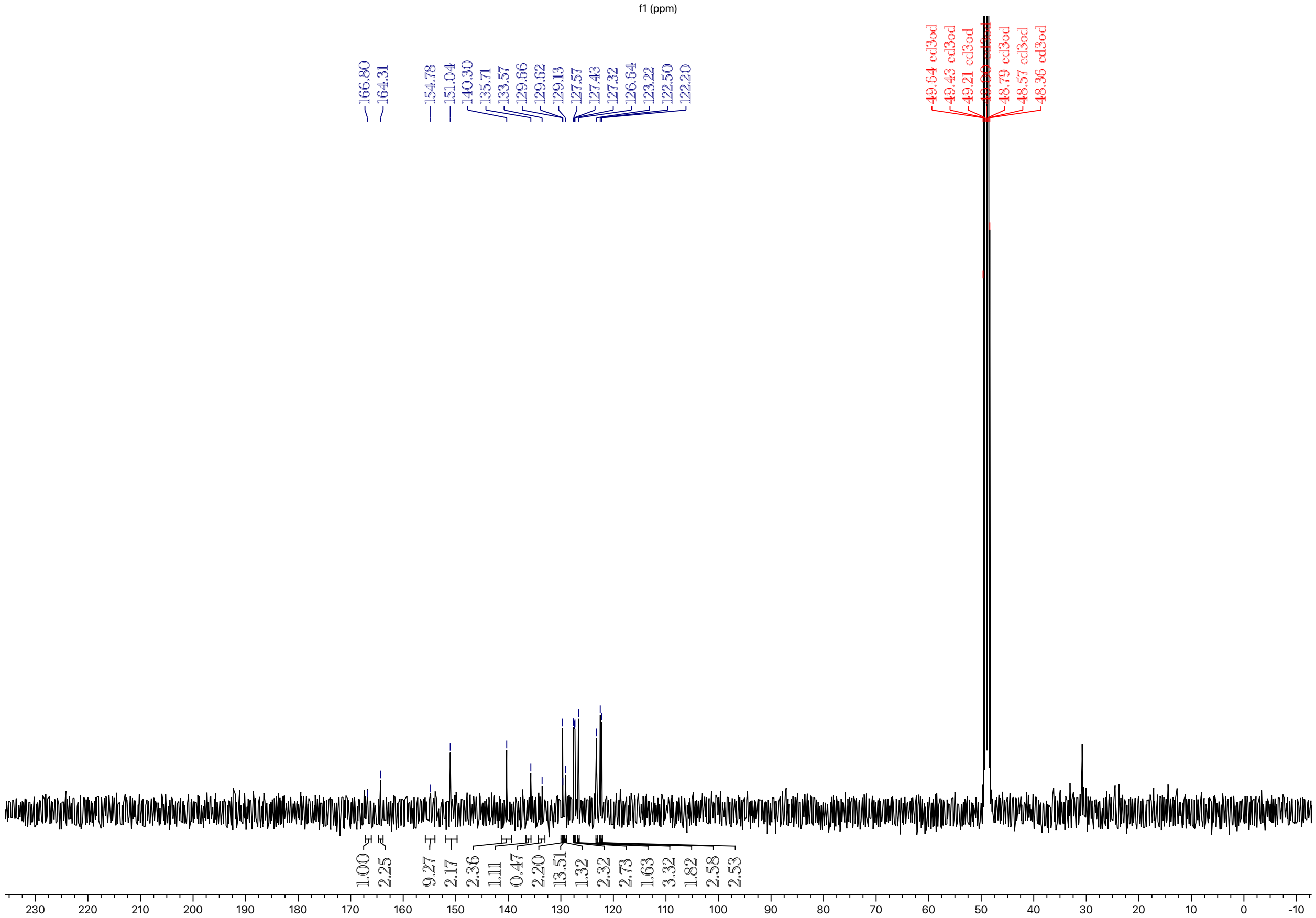
166.80
164.31
154.78
151.04
140.30
135.71
133.57
129.66
129.62
129.13
127.57
127.43
127.32
126.64
123.22
122.50
122.20
49.64 cd3od
49.43 cd3od
49.21 cd3od
48.79 cd3od
48.57 cd3od
48.36 cd3od
1.00
2.25
9.27
2.17
2.36
1.11
0.47
2.20
13.51
1.32
2.32
2.73
1.63
3.32
1.82
2.58
2.53
f1 (ppm)

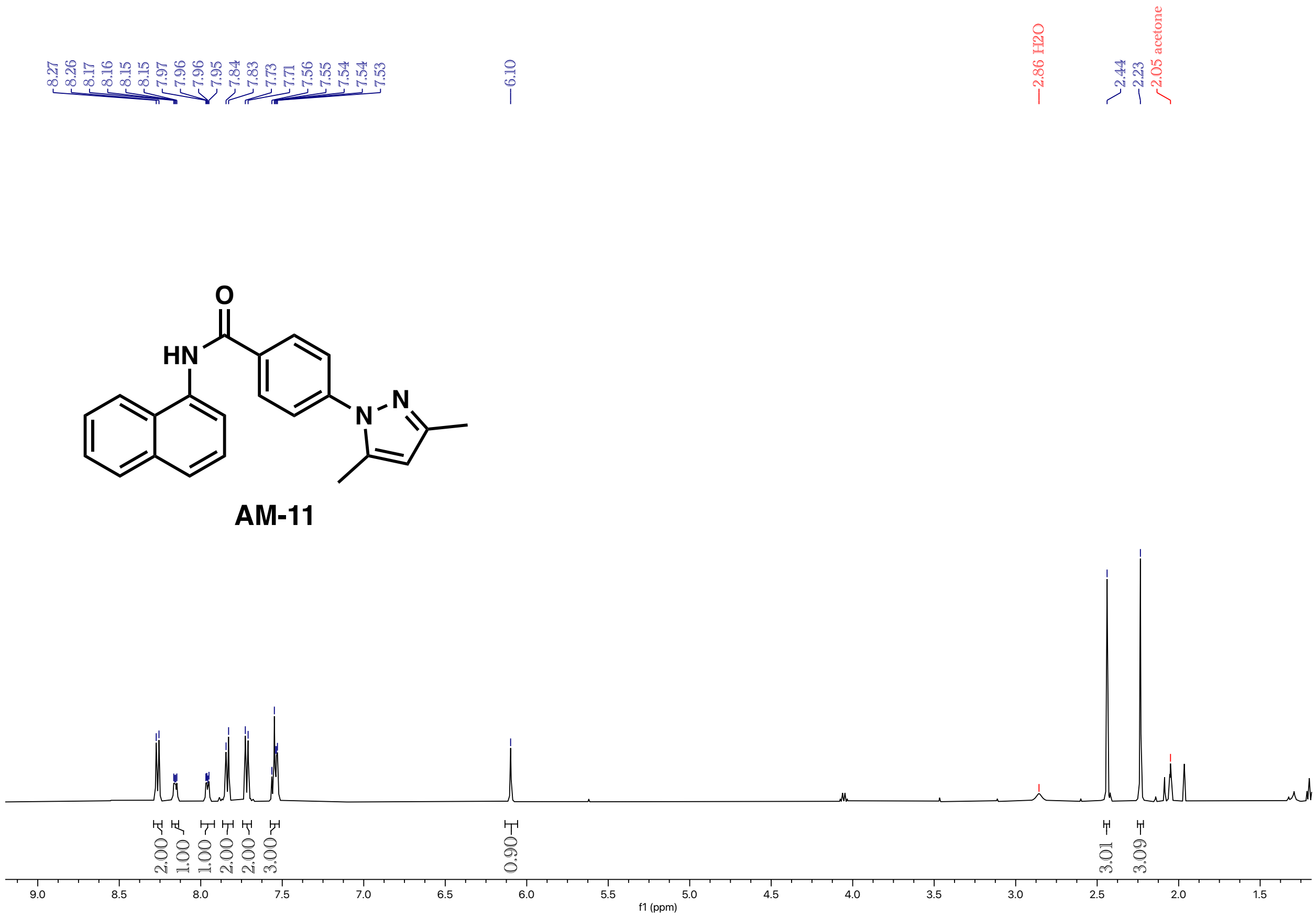

8.27
8.26
8.17
8.16
8.15
8.15
7.97
7.96
7.96
7.95
7.84
7.83
7.73
7.71
7.56
7.55
7.54
7.54
7.53
6.10
2.86 H2O
2.44
2.23
2.05 acetone
2.00
1.00
1.00
2.00
2.00
3.00
0.90
3.01
3.09
f1 (ppm)
AM-11

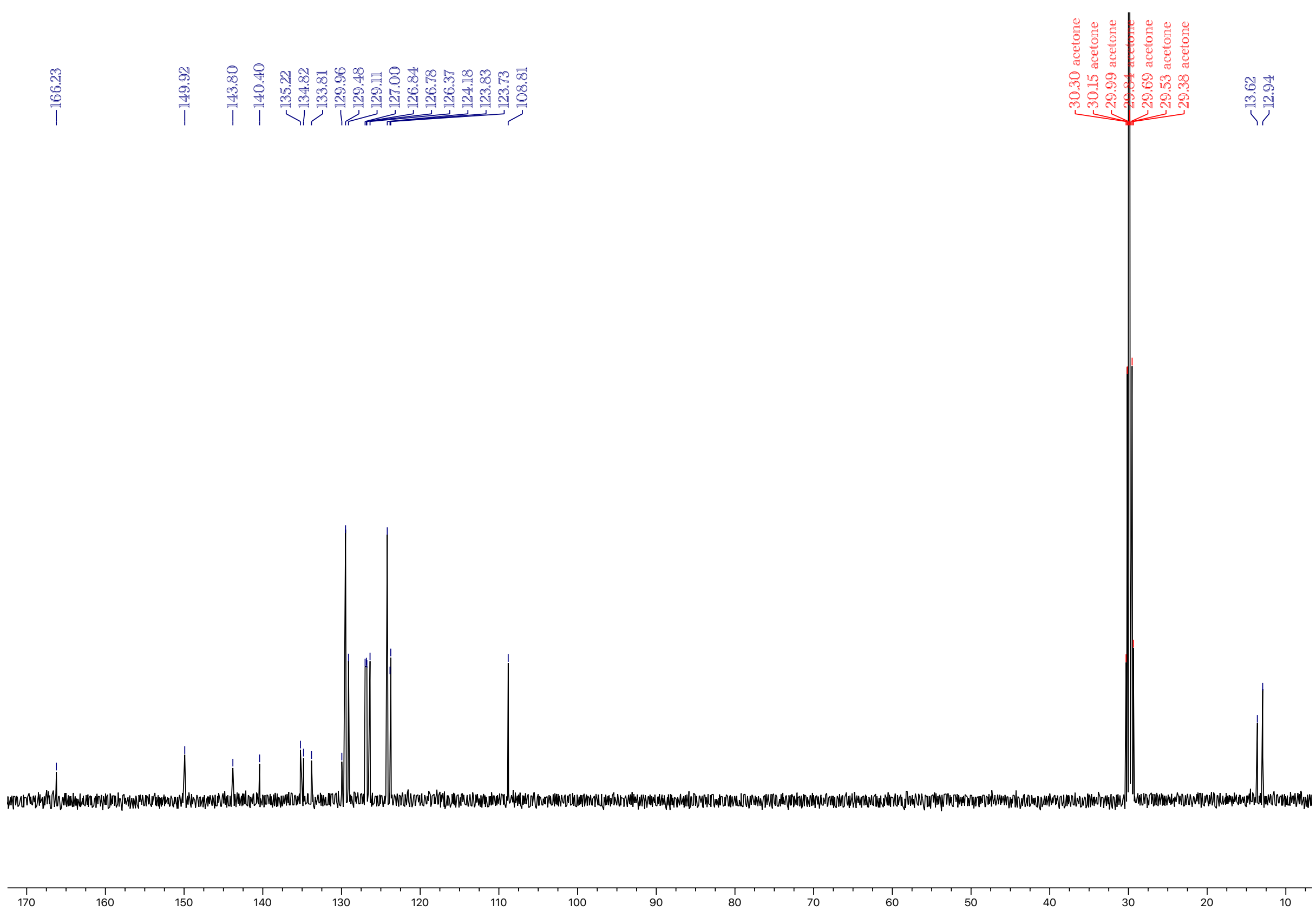

166.23
149.92
143.80
140.40
135.22
134.82
133.81
129.96
129.48
129.11
127.00
126.84
126.78
126.37
124.18
123.83
123.73
108.81
30.30 acetone
30.15 acetone
29.99 acetone
29.84 acetone
29.69 acetone
29.53 acetone
29.38 acetone
13.62
12.94
f1 (ppm)

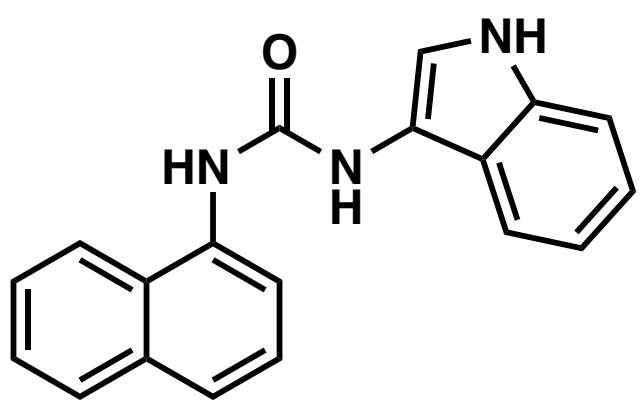

UR-01

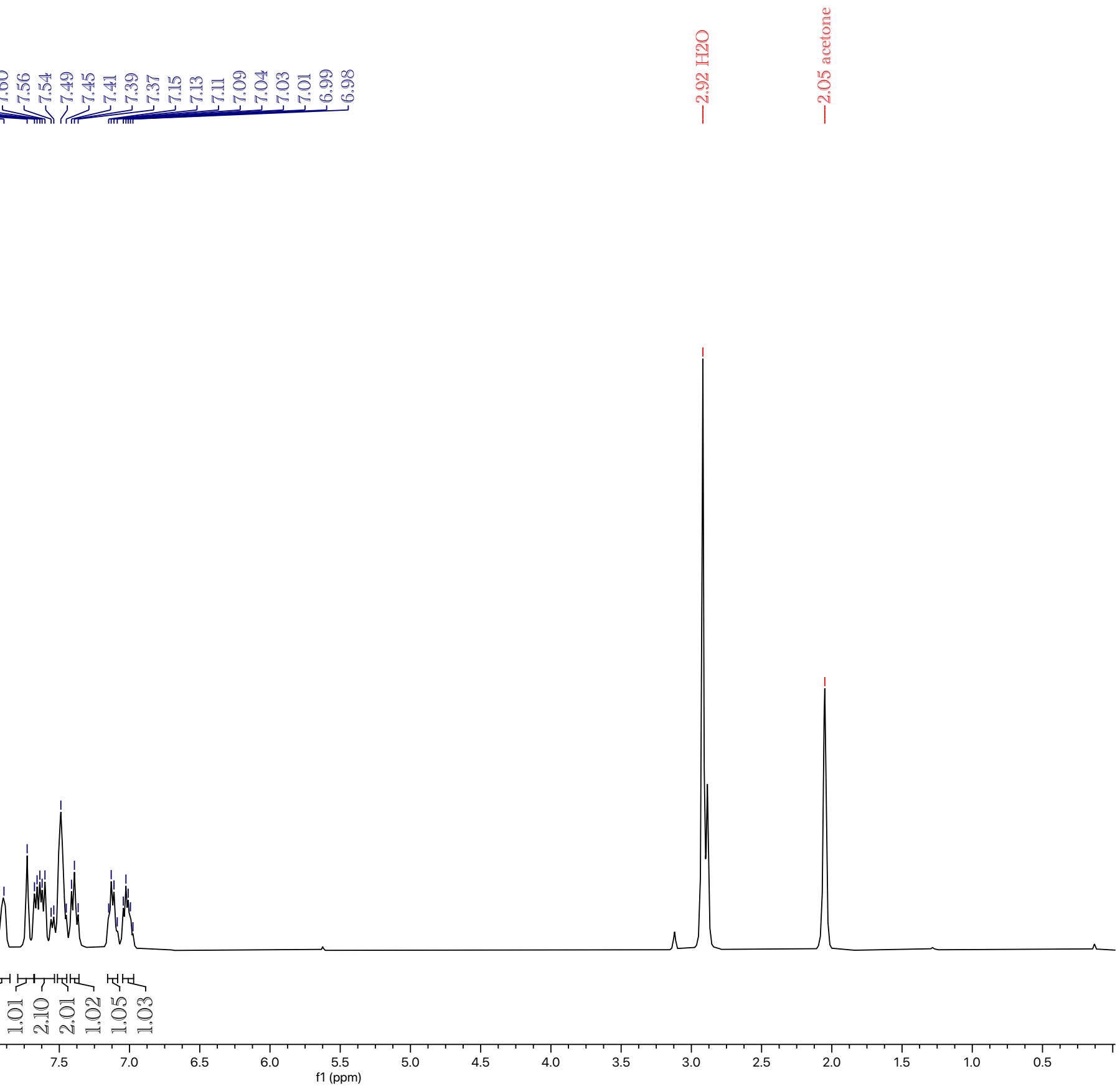

10.01
9.95
8.43
8.39
8.21
8.20
8.12
7.93
7.89
7.73
7.68
7.66
7.64
7.62
7.60
7.56
7.54
7.49
7.45
7.41
7.39
7.37
7.15
7.13
7.11
7.09
7.04
7.03
7.01
6.99
6.98
2.92 H2O
2.05 acetone
0.95
1.00
0.76
0.73
1.00
1.01
2.10
2.01
1.02
1.05
1.03
f1 (ppm)

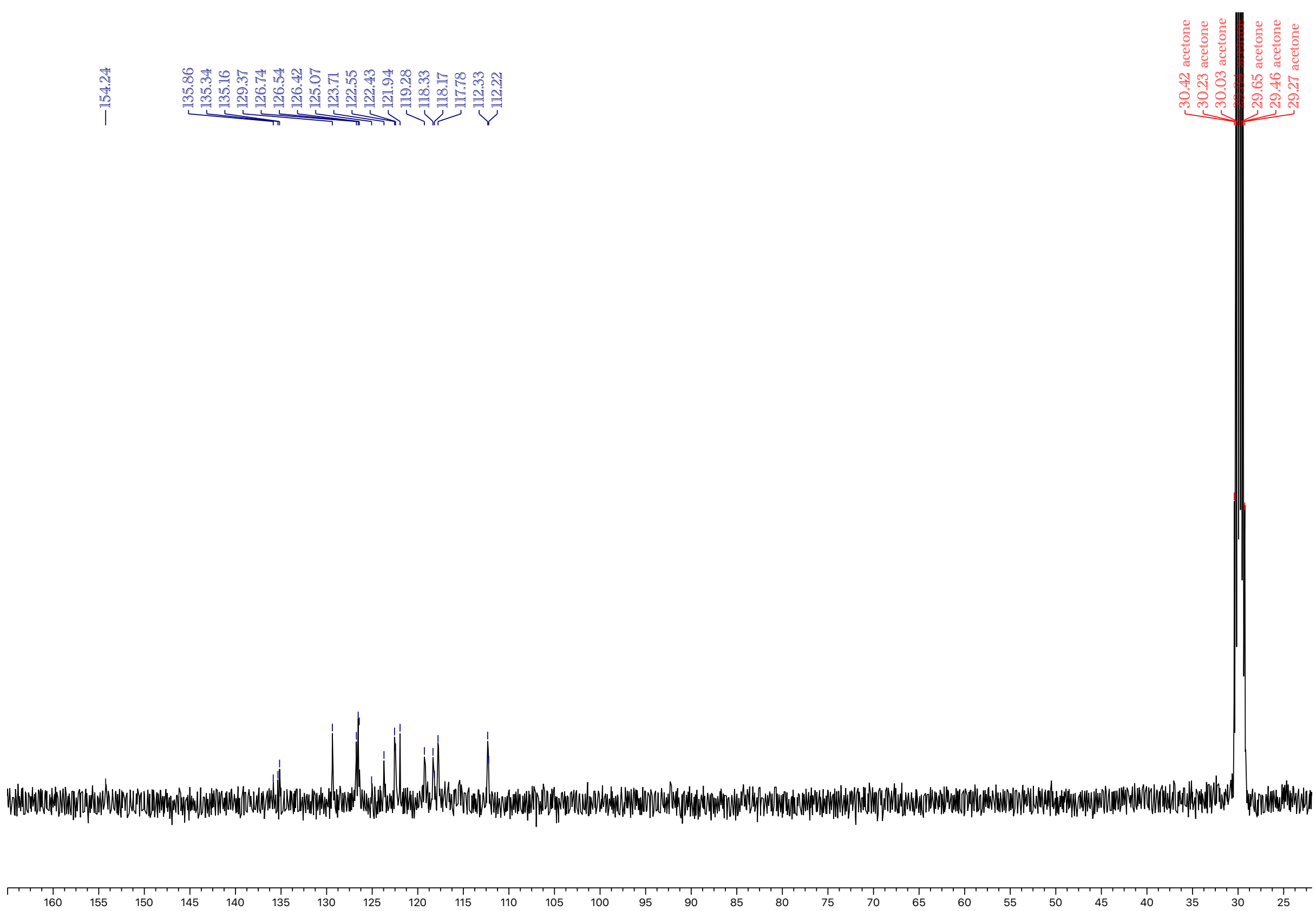

154.24
135.86
135.34
135.16
129.37
126.74
126.54
126.42
125.07
123.71
122.55
122.43
121.94
119.28
118.33
118.17
117.78
112.33
112.22
30.42 acetone
30.23 acetone
30.03 acetone
29.65 acetone
29.46 acetone
29.27 acetone
f1 (ppm)

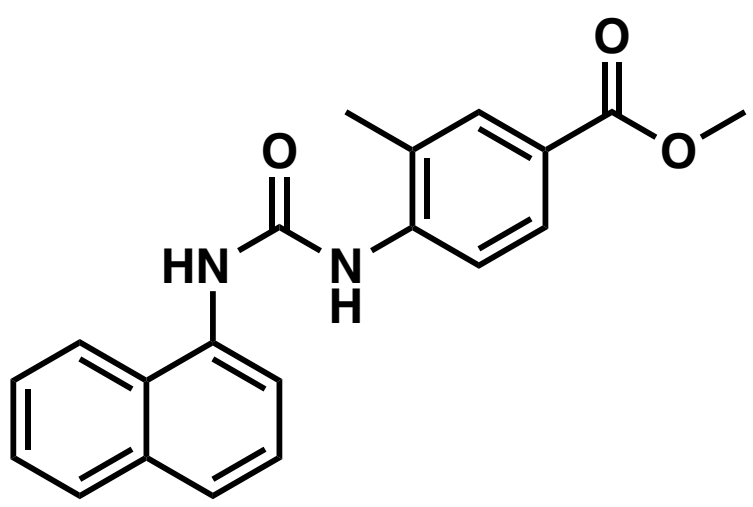

UR-02

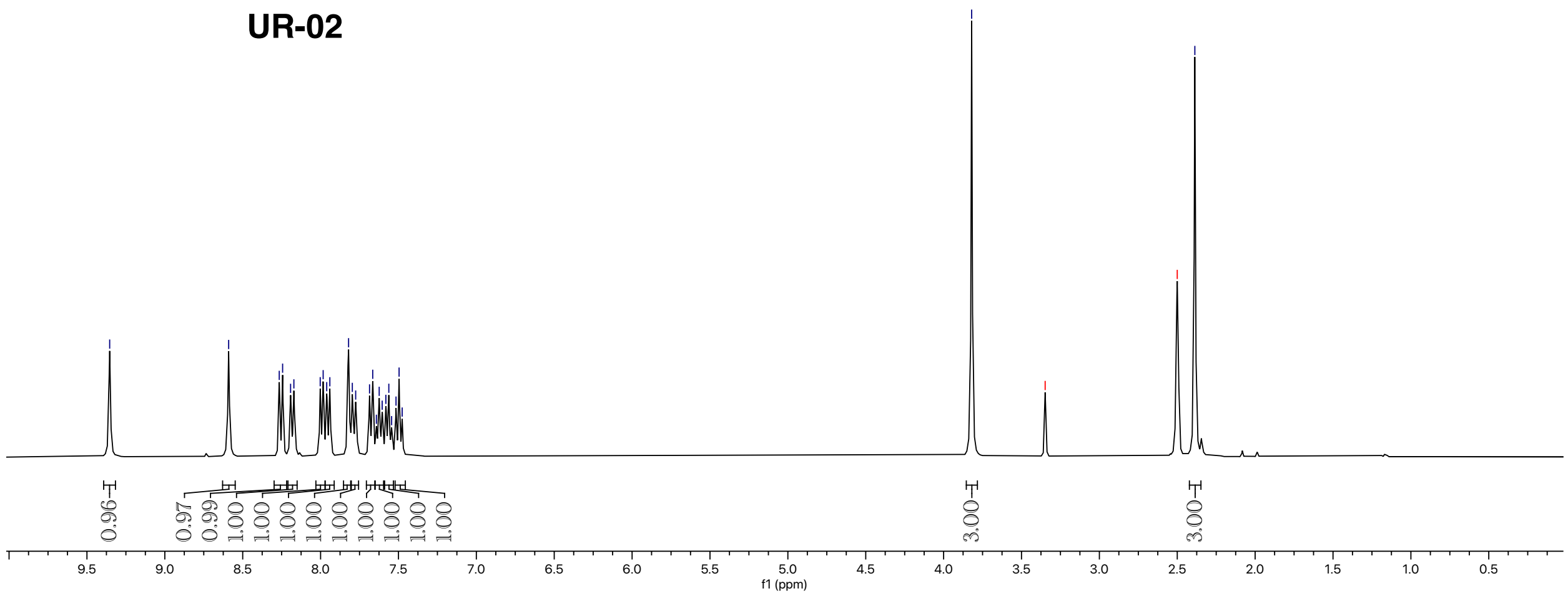

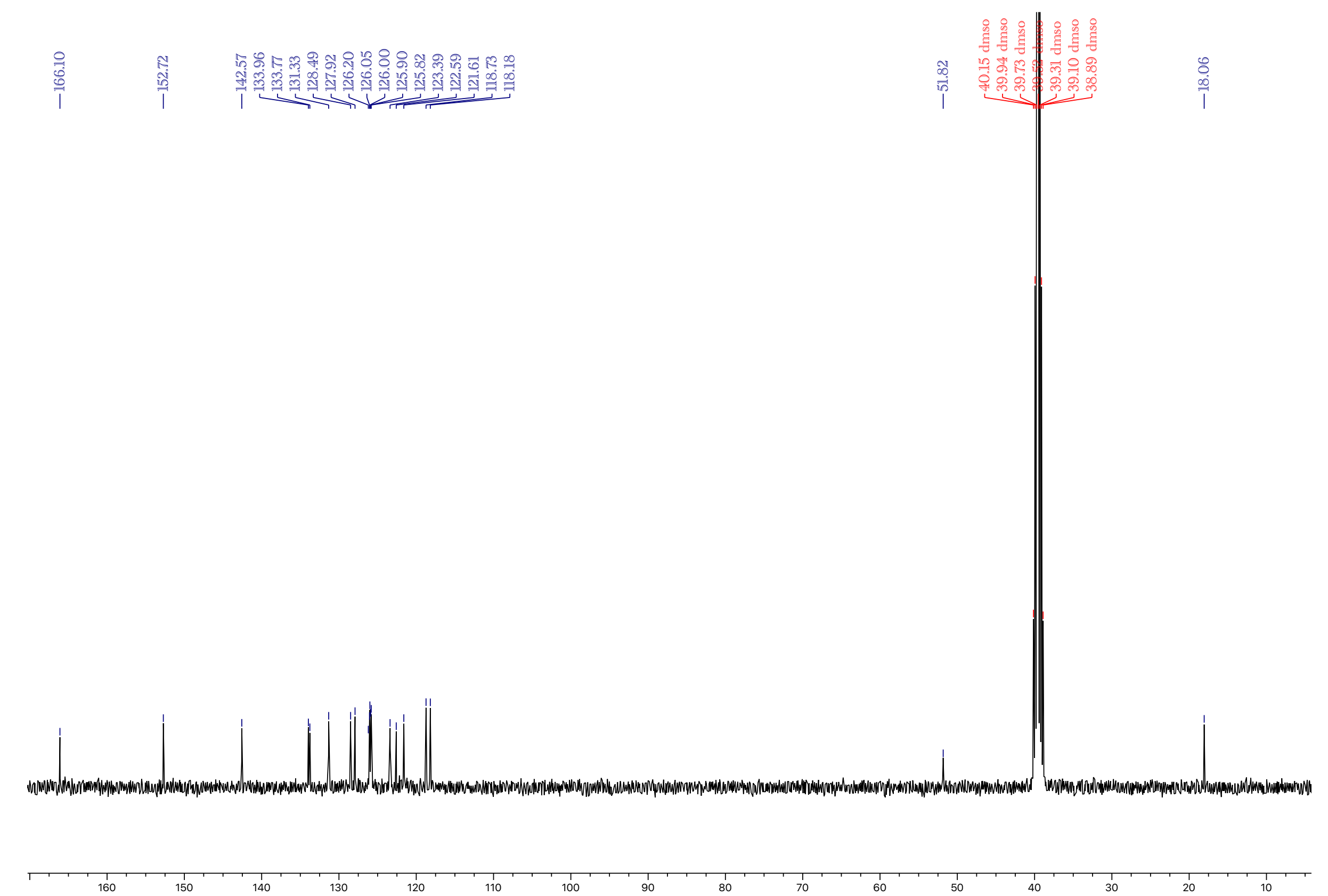

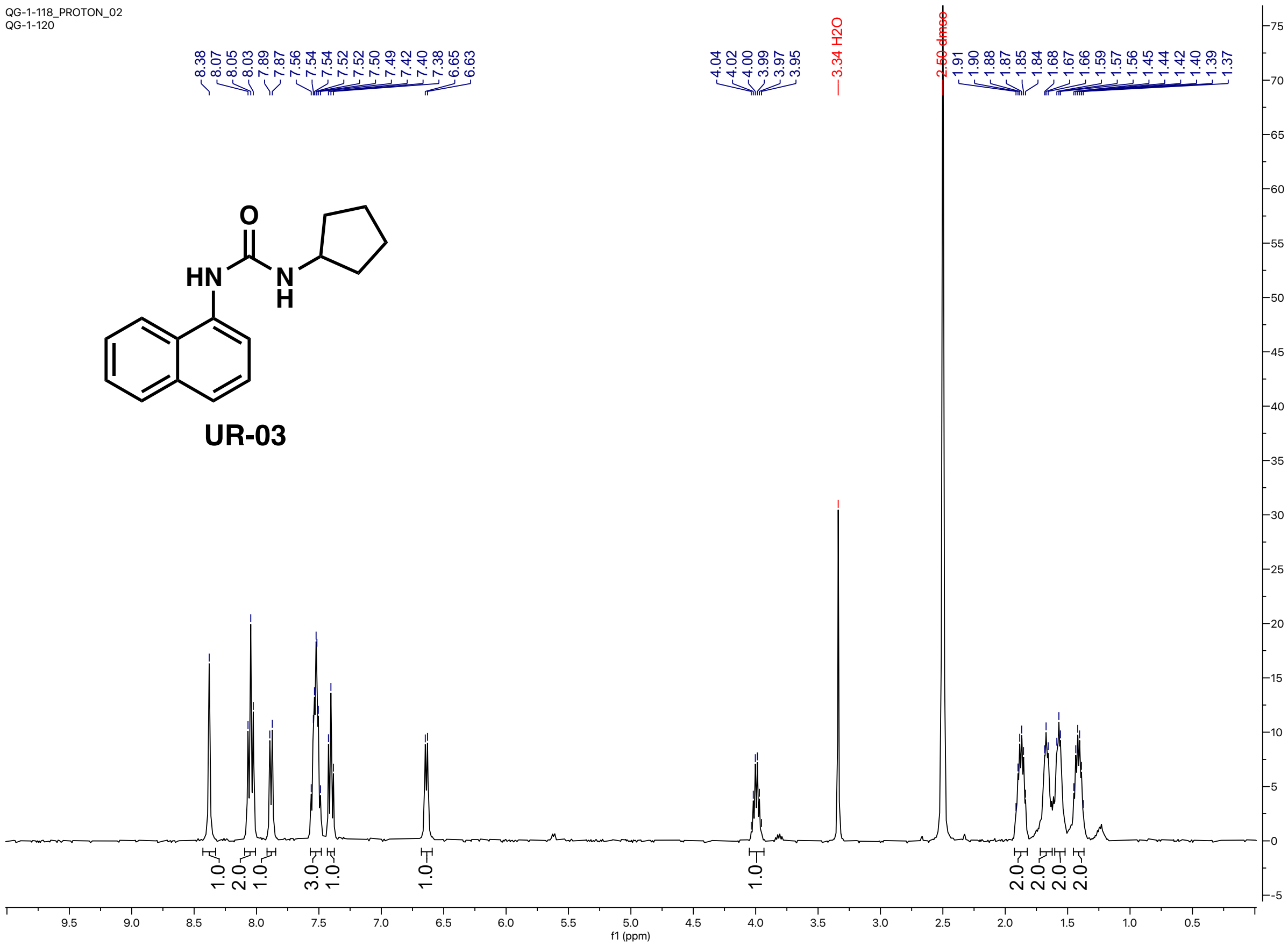
QG-1-118_PROTON_02
QG-1-120
8.38 8.07 8.05 8.03 7.89 7.87 7.56 7.54 7.54 7.52 7.52 7.50 7.49 7.42 7.40 7.38 6.65 6.63
4.04 4.02 4.00 3.99 3.97 3.95
3.34 H2O
2.50 dmso
1.91 1.90 1.88 1.87 1.85 1.84 1.68 1.67 1.66 1.59 1.57 1.56 1.45 1.44 1.42 1.40 1.39 1.37
HN
O
N
H
UR-03
1.0 2.0 1.0 3.0 1.0 1.0 1.0 2.0 2.0 2.0 2.0
f1 (ppm)

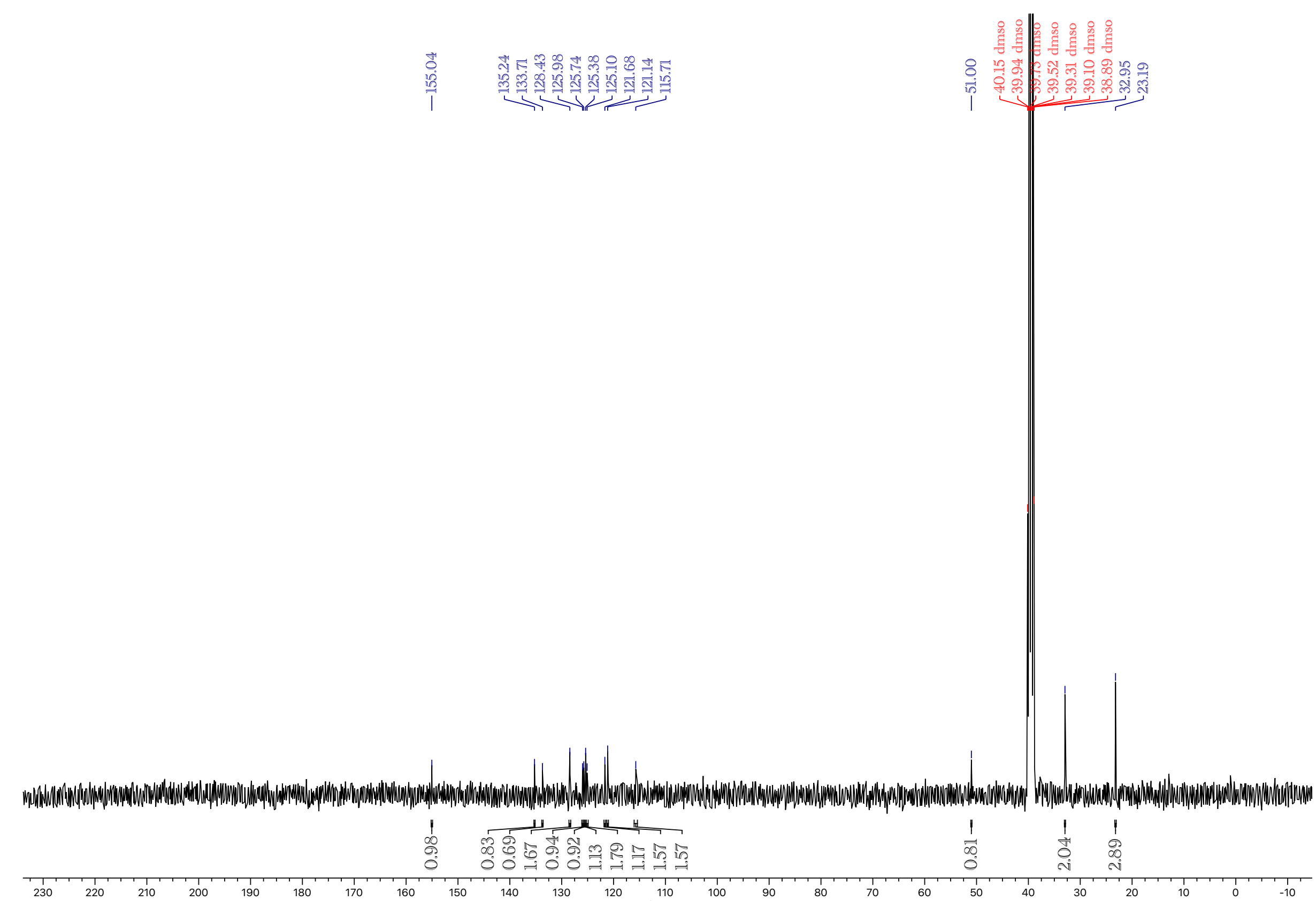
155.04
135.24 133.71 128.43 125.98 125.74 125.38 125.10 121.68 121.14 115.71
51.00
40.15 dmso 39.94 dmso 39.73 dmso 39.52 dmso 39.31 dmso 39.10 dmso 38.89 dmso
32.95 23.19
0.98 0.83 0.69 1.67 0.94 0.92 1.13 1.79 1.17 1.57 1.57 0.81 2.04 2.89
f1 (ppm)

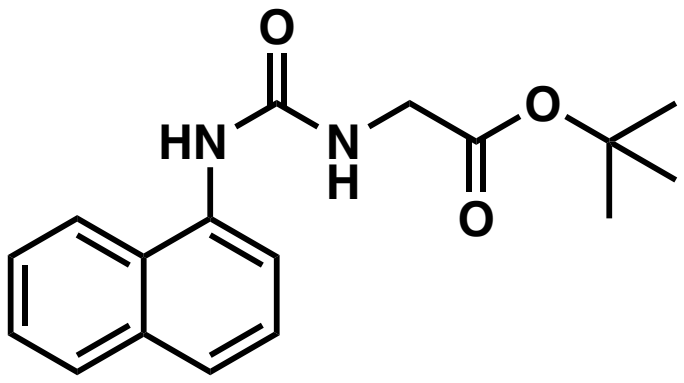

O
HN
N
H
O
O

UR-04

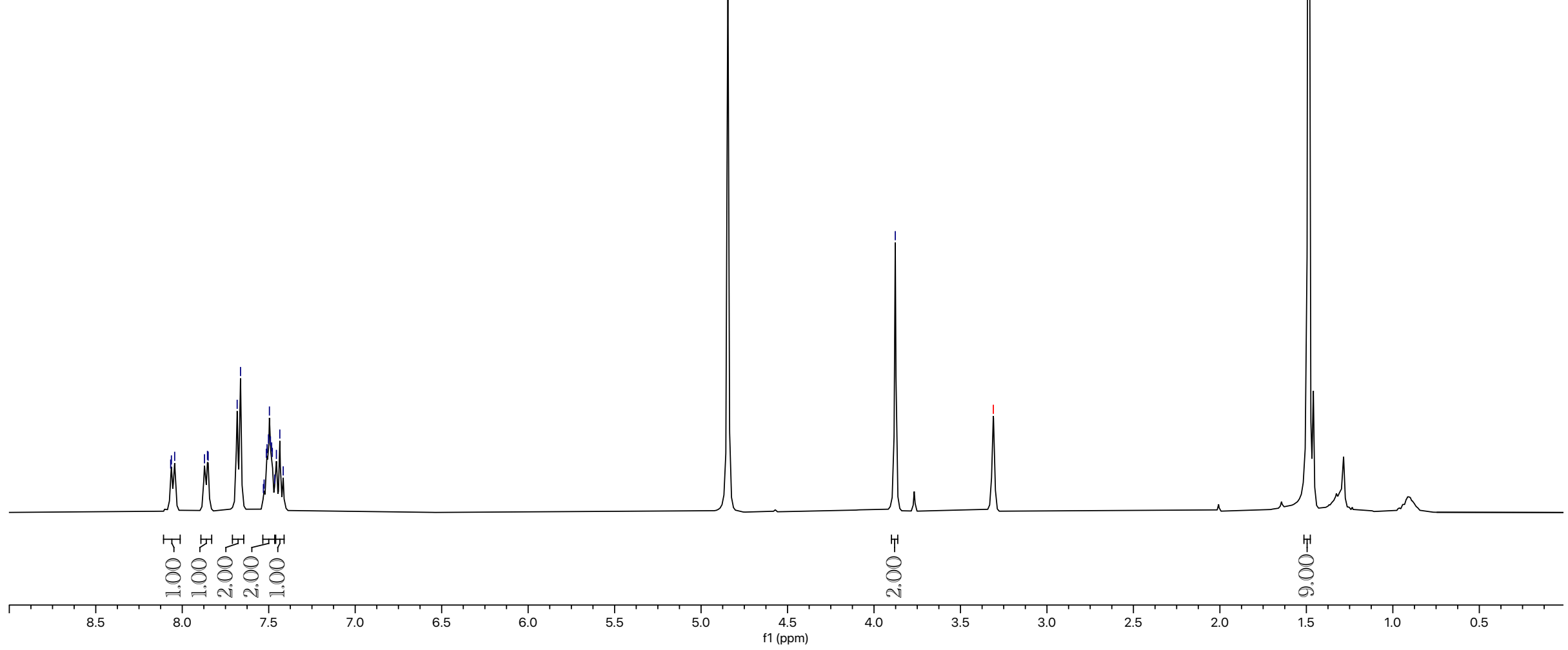

8.07
8.06
8.04
7.87
7.85
7.85
7.68
7.66
7.53
7.53
7.51
7.51
7.50
7.50
7.49
7.48
7.48
7.47
7.46
7.44
7.42
4.84 HDO
3.88
3.31 cd3od
1.49
1.00
1.00
2.00
2.00
1.00
2.00
9.00
8.5 8.0 7.5 7.0 6.5 6.0 5.5 5.0 4.5 4.0 3.5 3.0 2.5 2.0 1.5 1.0 0.5
f1 (ppm)

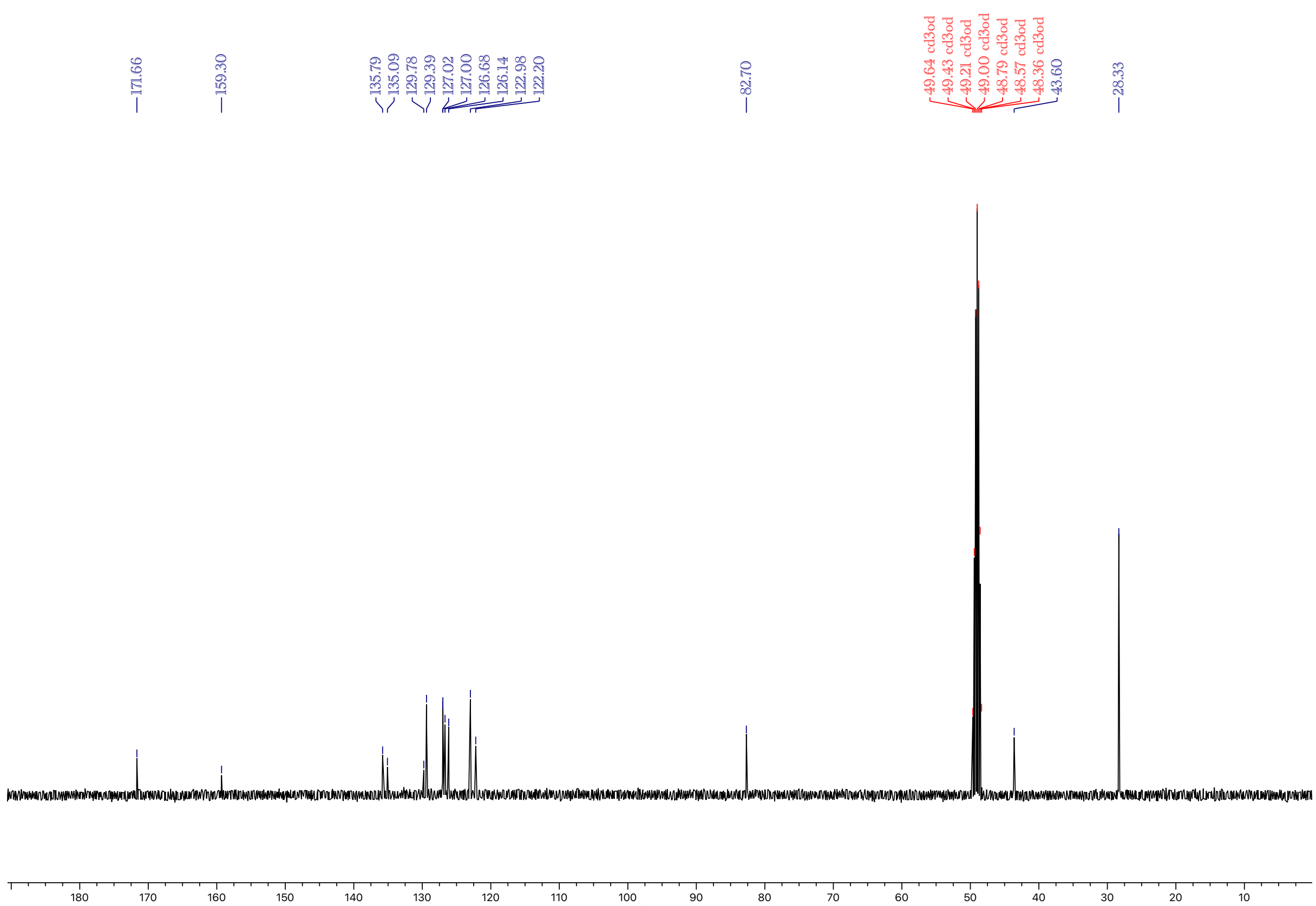

171.66
159.30
135.79
135.09
129.78
129.39
127.02
127.00
126.68
126.14
122.98
122.20
82.70
49.64 cd3od
49.43 cd3od
49.21 cd3od
49.00 cd3od
48.79 cd3od
48.57 cd3od
48.36 cd3od
43.60
28.33
180 170 160 150 140 130 120 110 100 90 80 70 60 50 40 30 20 10
f1 (ppm)

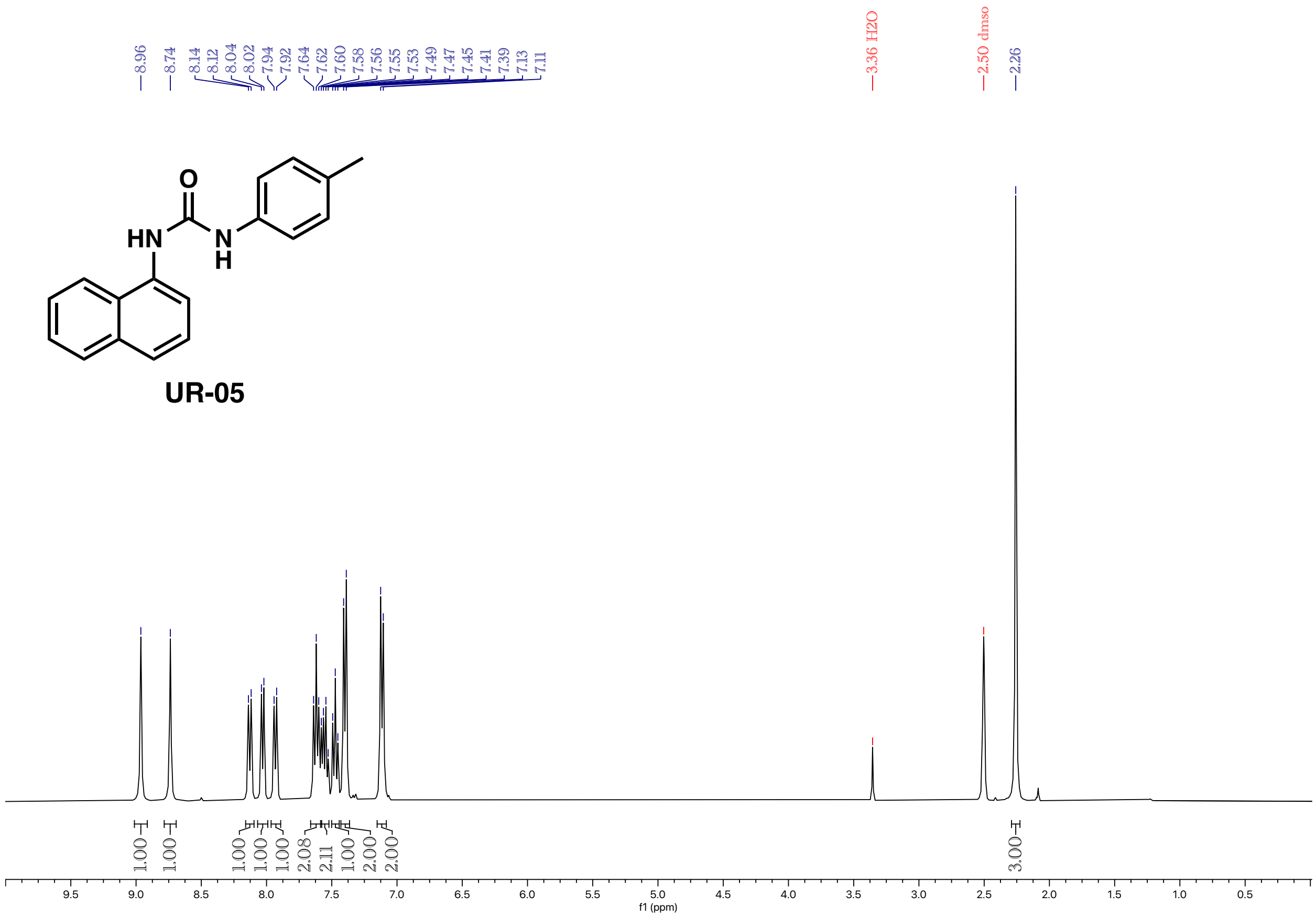

8.96
8.74
8.14
8.12
8.04
8.02
7.94
7.92
7.64
7.62
7.60
7.58
7.56
7.55
7.53
7.49
7.47
7.45
7.41
7.39
7.13
7.11
3.36 H2O
2.50 dmso
2.26
O
HN
N
H
UR-05
1.00
1.00
1.00
1.00
1.00
2.08
2.11
1.00
2.00
2.00
3.00
f1 (ppm)

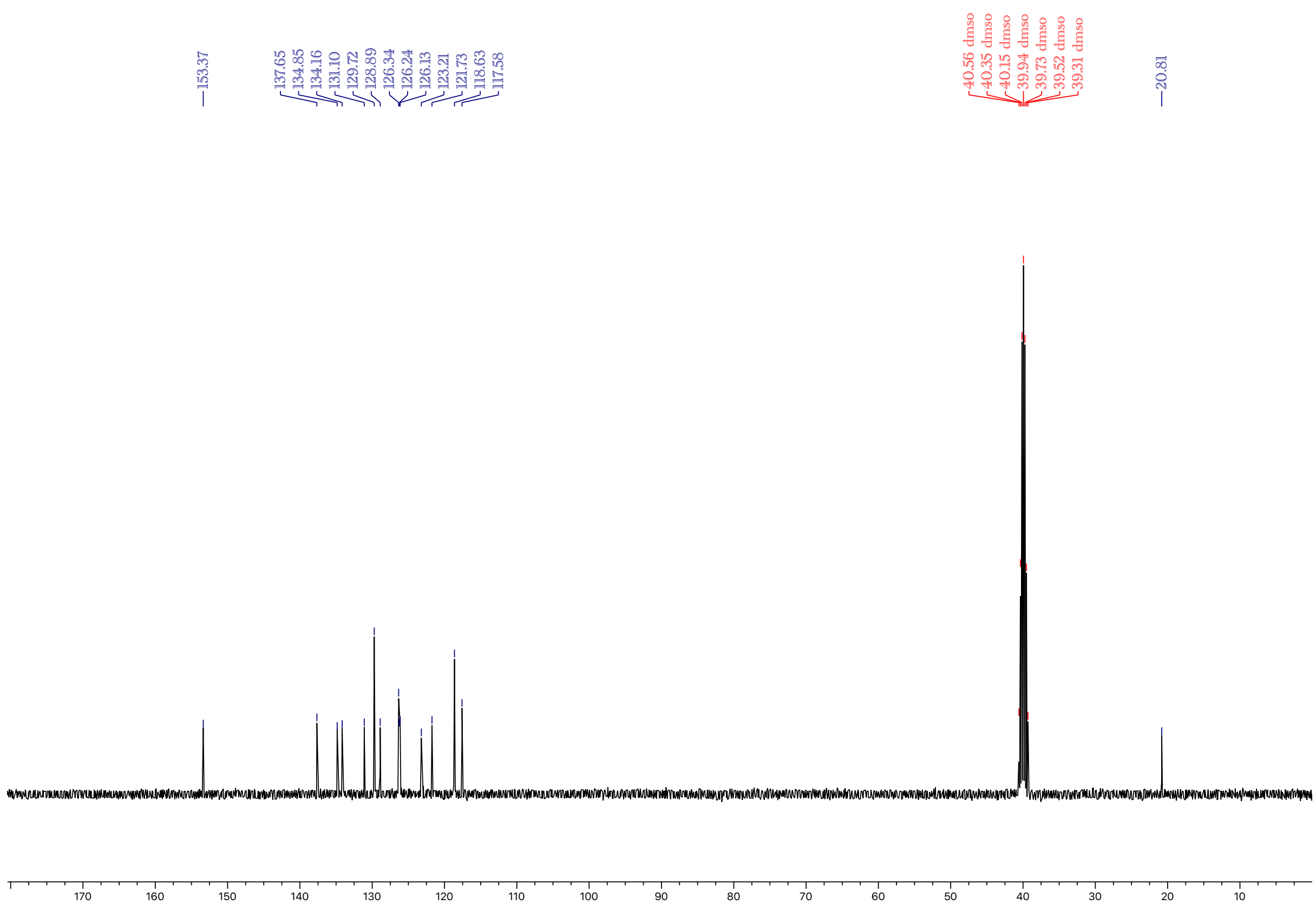

153.37
137.65
134.85
134.16
131.10
129.72
128.89
126.34
126.24
126.13
123.21
121.73
118.63
117.58
40.56 dmso
40.35 dmso
40.15 dmso
39.94 dmso
39.73 dmso
39.52 dmso
39.31 dmso
20.81
f1 (ppm)

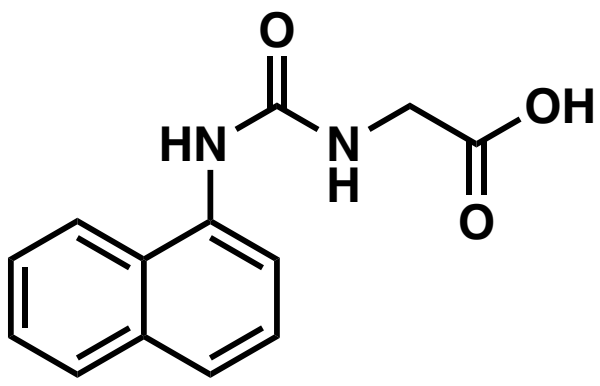

UR-06

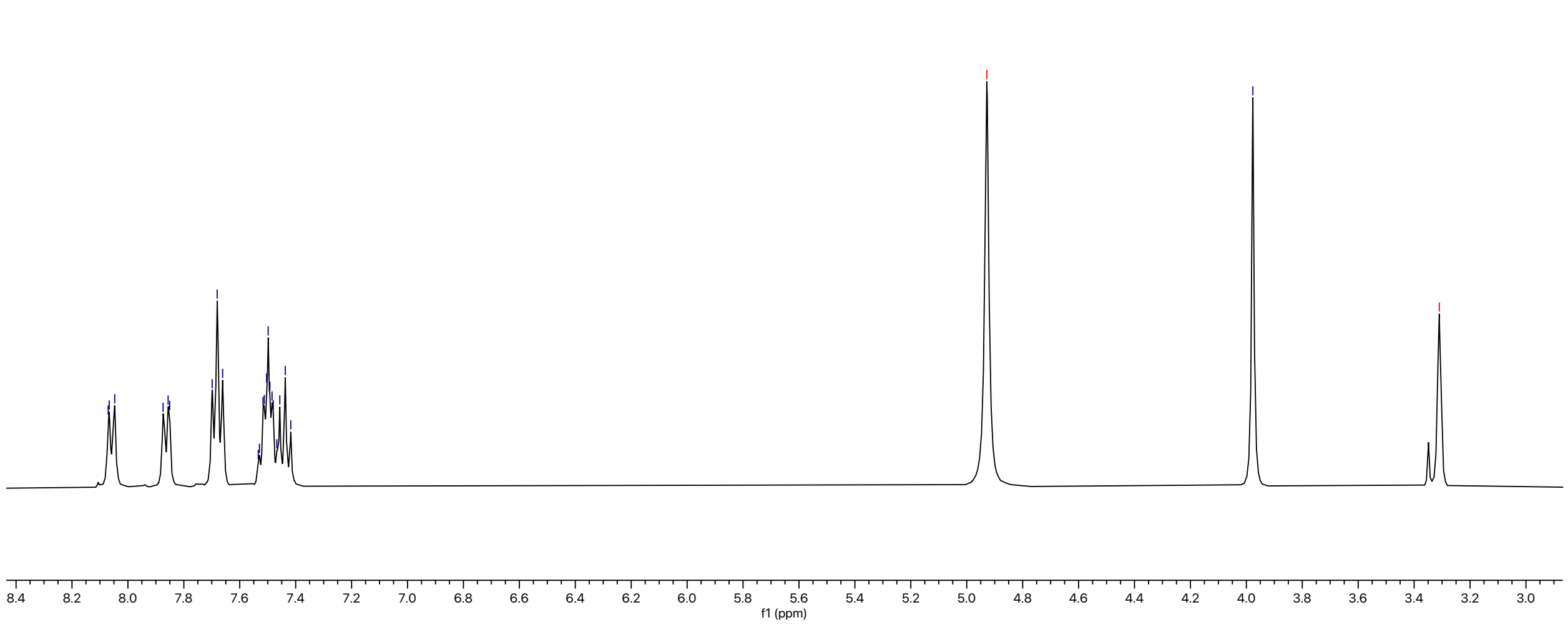

8.07
8.07
8.05
7.87
7.86
7.85
7.70
7.68
7.66
7.53
7.53
7.52
7.51
7.50
7.50
7.49
7.48
7.48
7.47
7.46
7.44
7.42
4.93 HDO
3.98
3.31 cd3od
f1 (ppm)

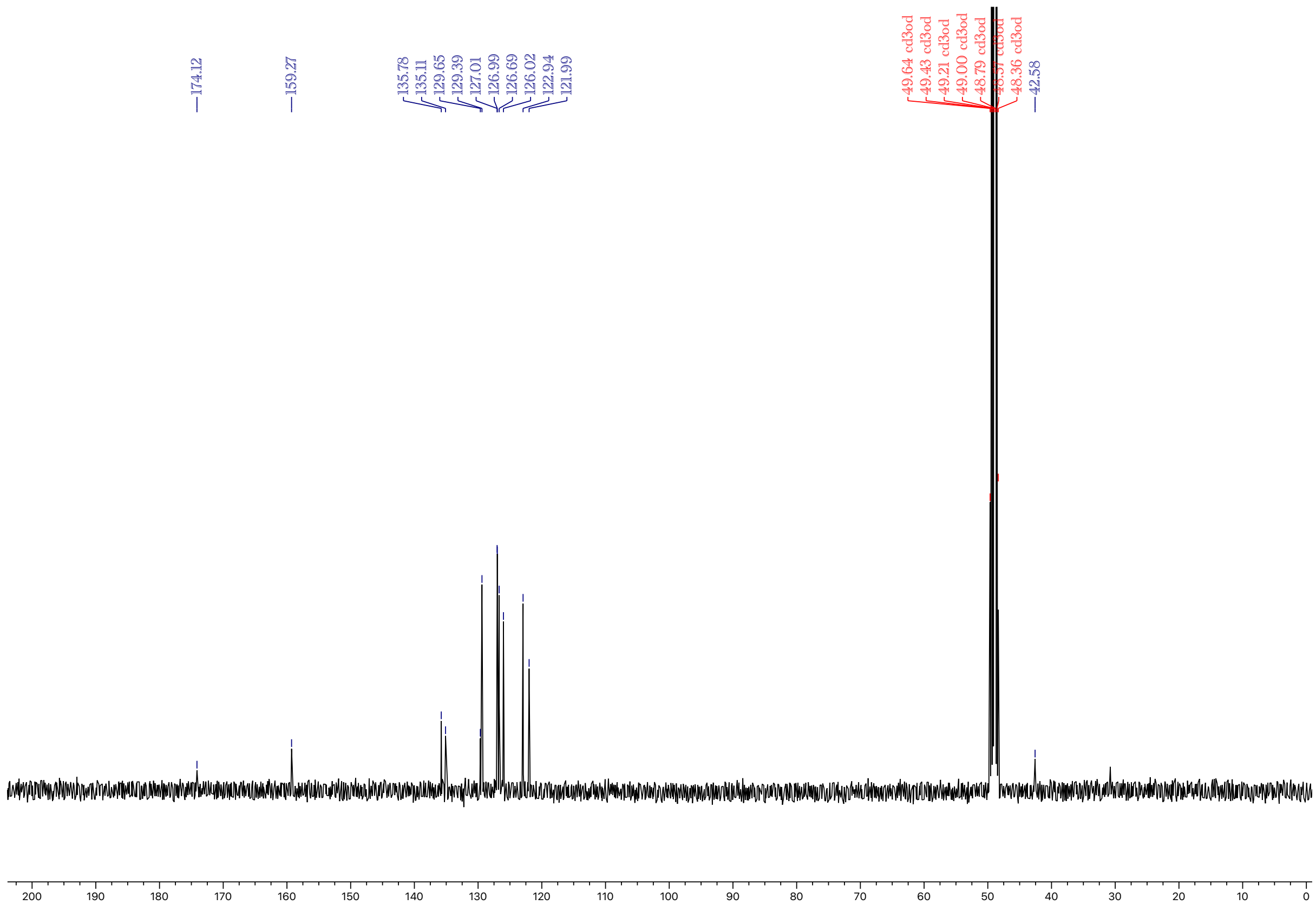

174.12
159.27
135.78
135.11
129.65
129.39
127.01
126.99
126.69
126.02
122.94
121.99
49.64 cd3od
49.43 cd3od
49.21 cd3od
49.00 cd3od
48.79 cd3od
48.57 cd3od
48.36 cd3od
42.58
f1 (ppm)

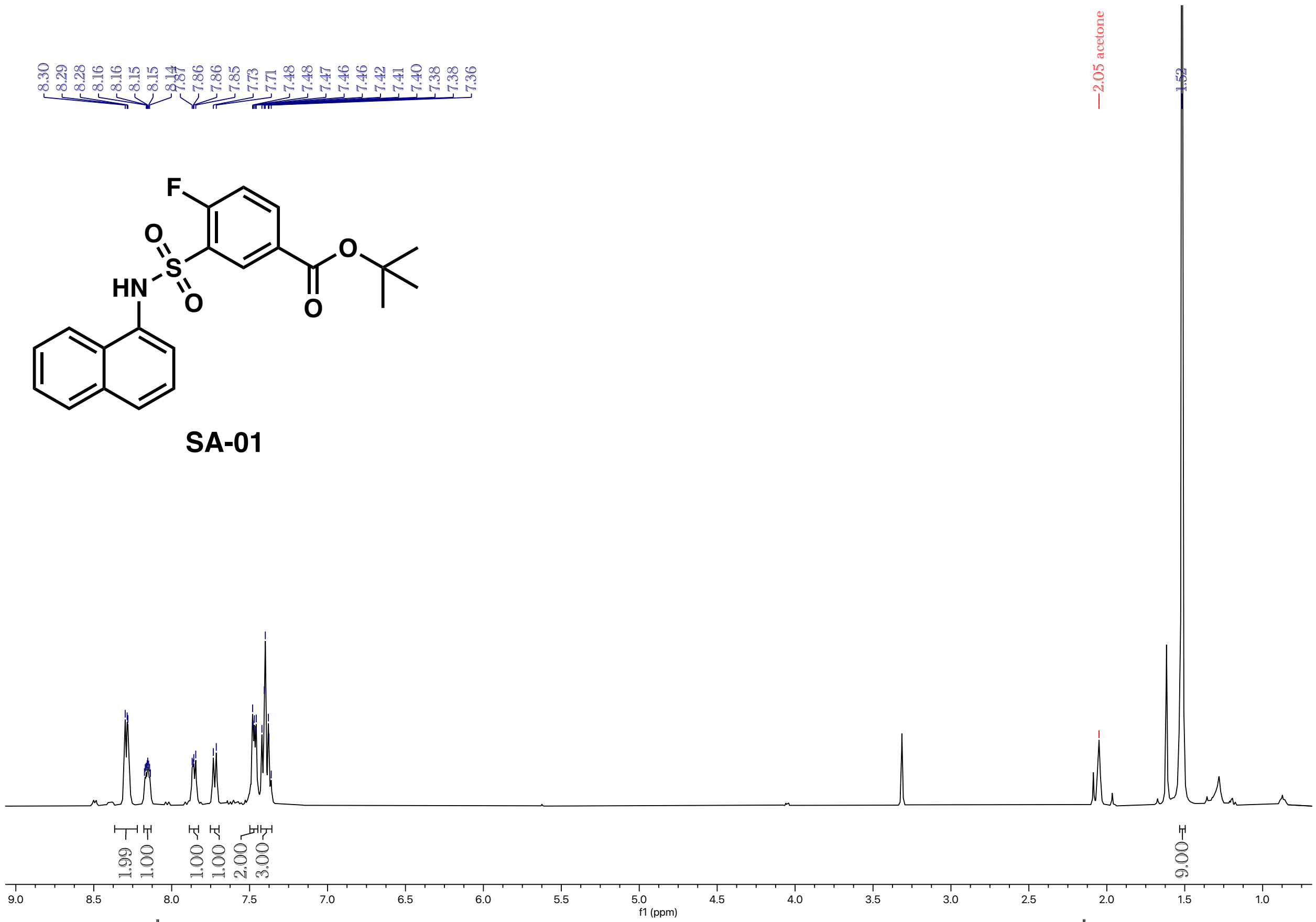
8.30
8.29
8.28
8.16
8.16
8.15
8.15
8.14
7.87
7.86
7.86
7.85
7.73
7.71
7.48
7.48
7.47
7.46
7.46
7.42
7.41
7.40
7.38
7.38
7.36
2.05 acetone
1.52
F
O
S
HN
O
O
O
O
SA-01
1.99
1.00
1.00
1.00
2.00
3.00
9.00
f1 (ppm)

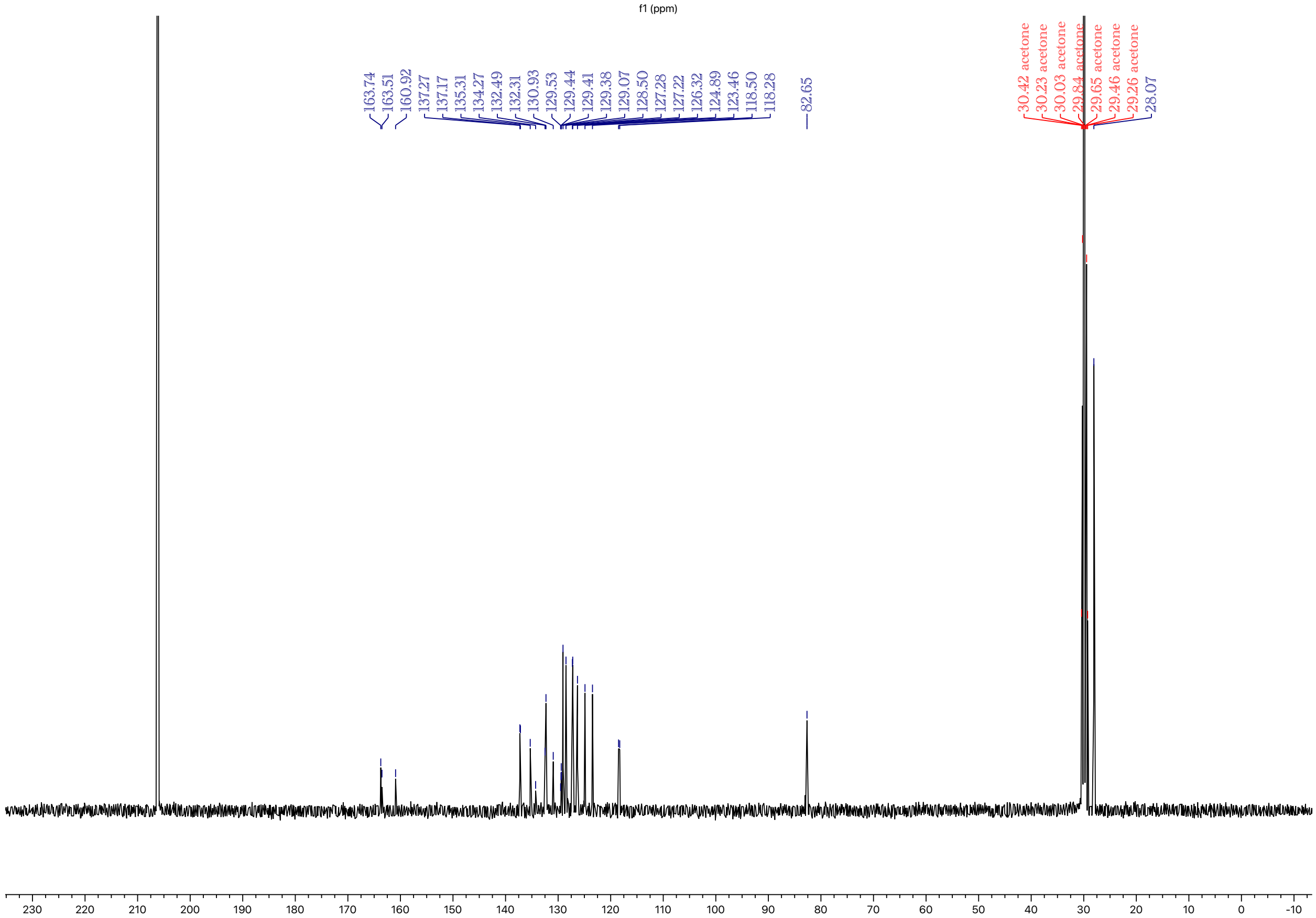
163.74
163.51
160.92
137.27
137.17
135.31
134.27
132.49
132.31
130.93
129.53
129.44
129.41
129.38
129.07
128.50
127.28
127.22
126.32
124.89
123.46
118.50
118.28
82.65
30.42 acetone
30.23 acetone
30.03 acetone
29.84 acetone
29.65 acetone
29.46 acetone
29.26 acetone
28.07
f1 (ppm)

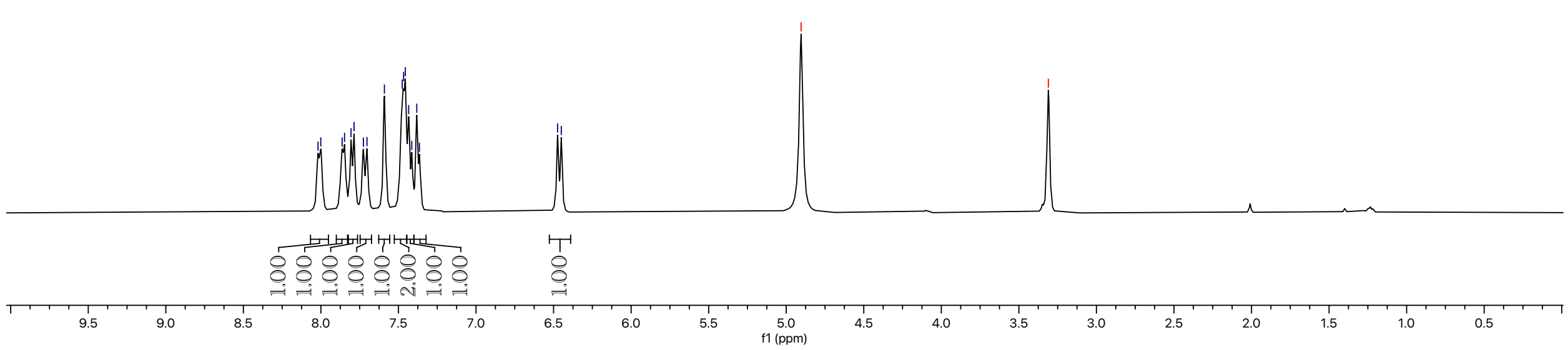
8.02
8.00
7.86
7.85
7.81
7.79
7.73
7.70
7.59
7.48
7.47
7.46
7.43
7.41
7.38
7.36
6.47
6.45
4.90 HDO
3.31 cd3od
1.00
1.00
1.00
1.00
1.00
2.00
1.00
1.00
1.00
f1 (ppm)

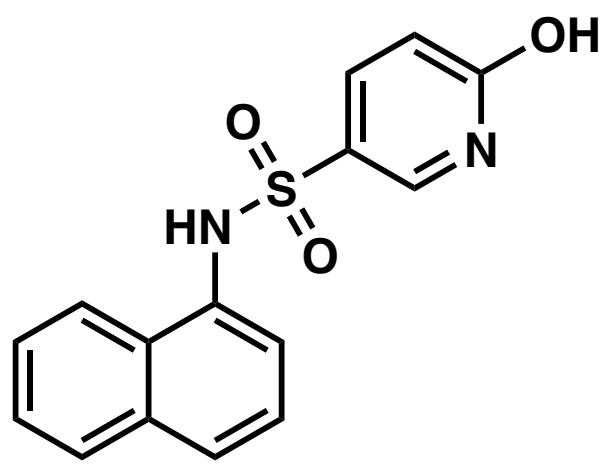
OH
N
O
S
HN
O

SA-02

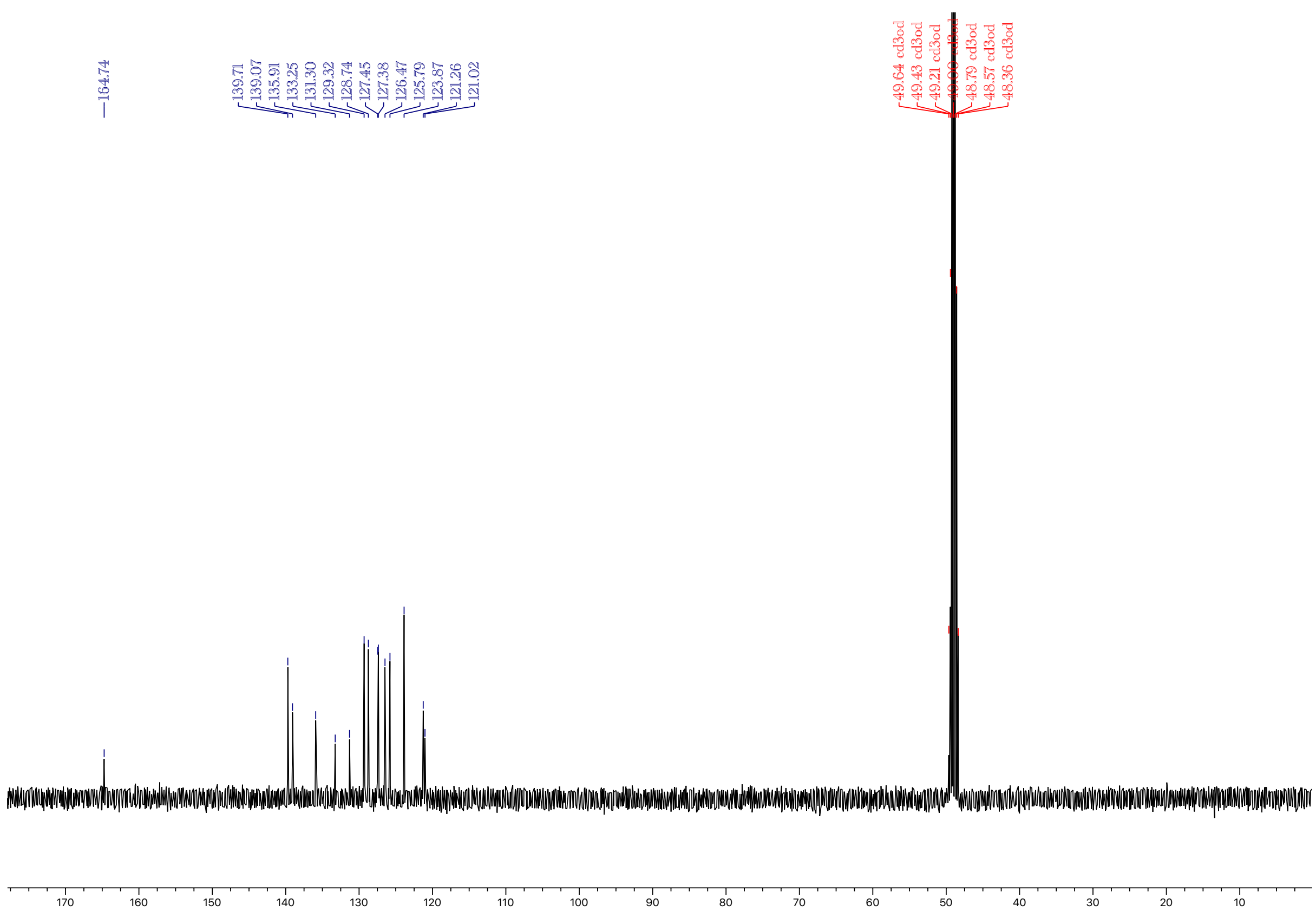
164.74
139.71
139.07
135.91
133.25
131.30
129.32
128.74
127.45
127.38
126.47
125.79
123.87
121.26
121.02
49.64 cd3od
49.43 cd3od
49.21 cd3od
48.79 cd3od
48.57 cd3od
48.36 cd3od
f1 (ppm)

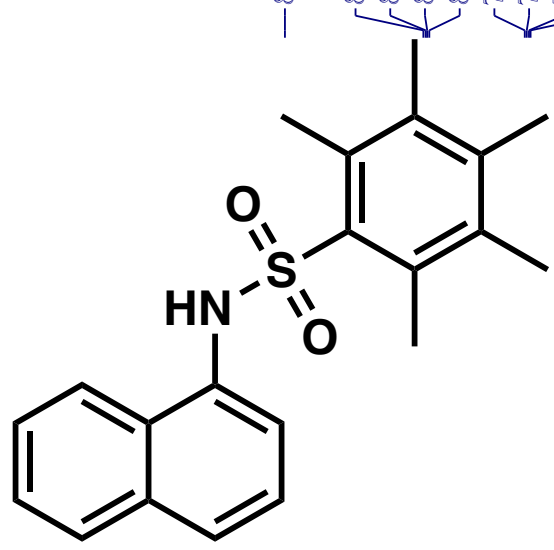

SA-03

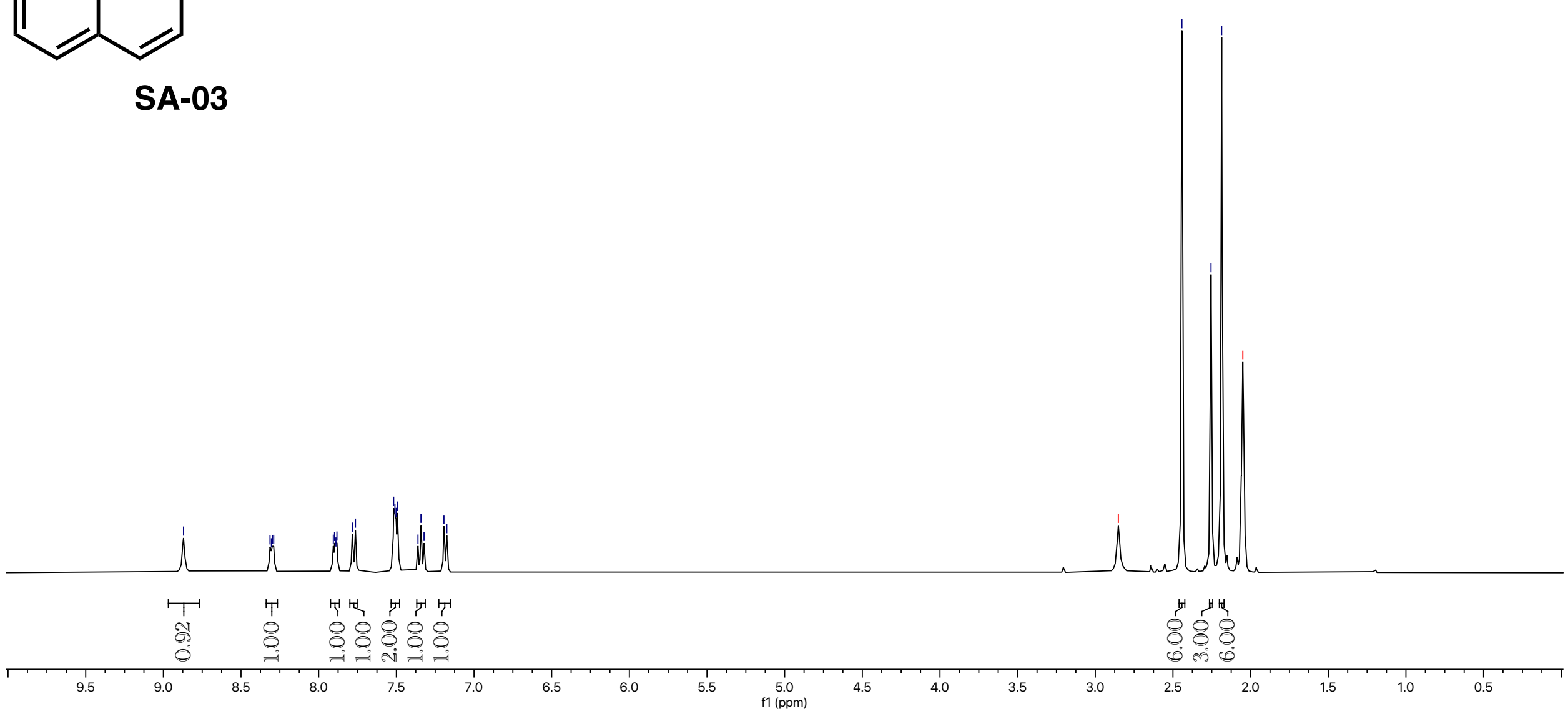
8.87
8.31
8.30
8.30
8.29
7.91
7.90
7.89
7.88
7.78
7.76
7.52
7.51
7.50
7.49
7.36
7.34
7.32
7.19
7.18
2.85 H2O
2.44
2.25
2.19
2.05 acetone
0.92
1.00
1.00
1.00
2.00
1.00
1.00
6.00
3.00
6.00
f1 (ppm)

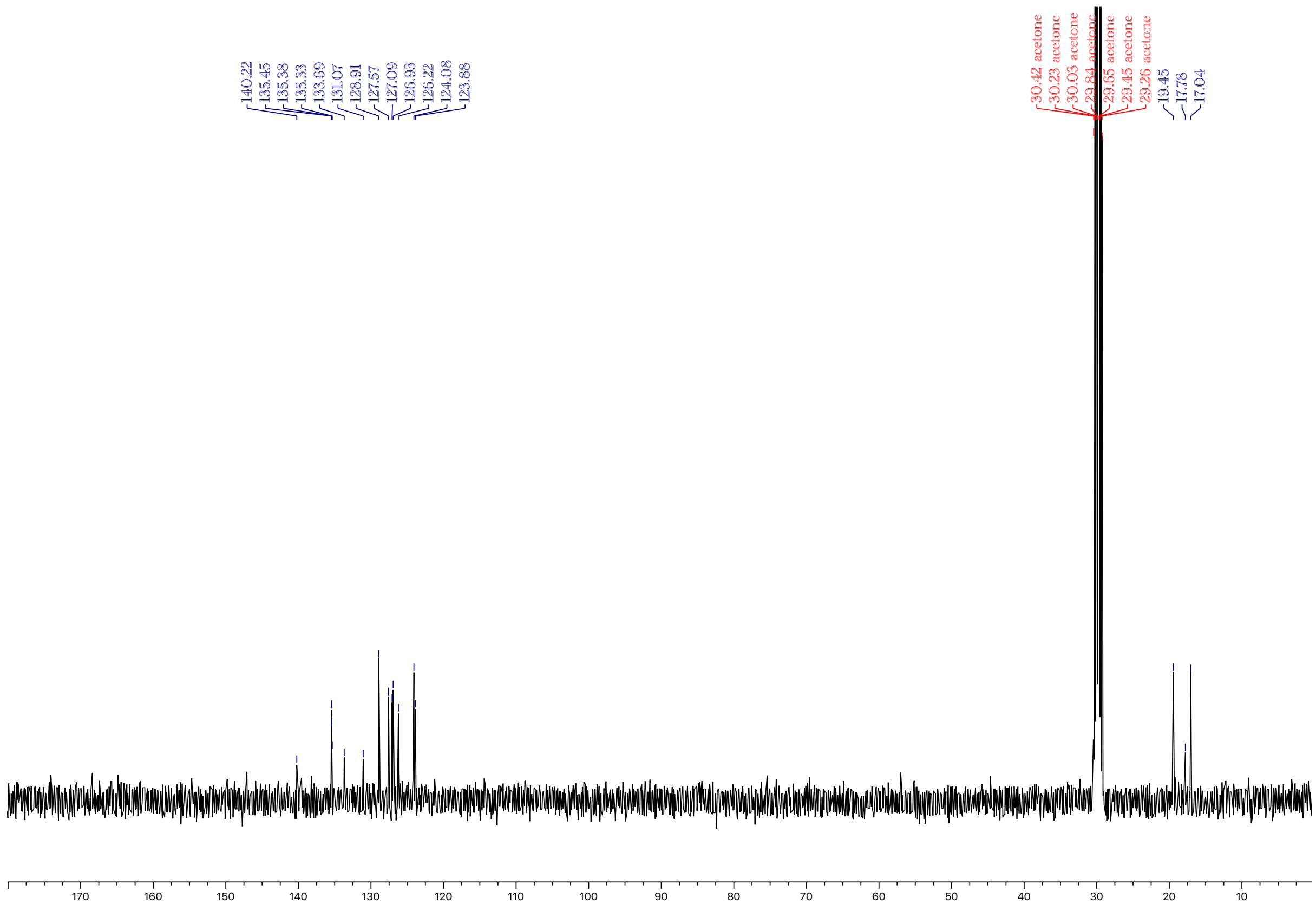
140.22
135.45
135.38
135.33
133.69
131.07
128.91
127.57
127.09
126.93
126.22
124.08
123.88
30.42 acetone
30.23 acetone
30.03 acetone
29.84 acetone
29.65 acetone
29.45 acetone
29.26 acetone
19.45
17.78
17.04
f1 (ppm)

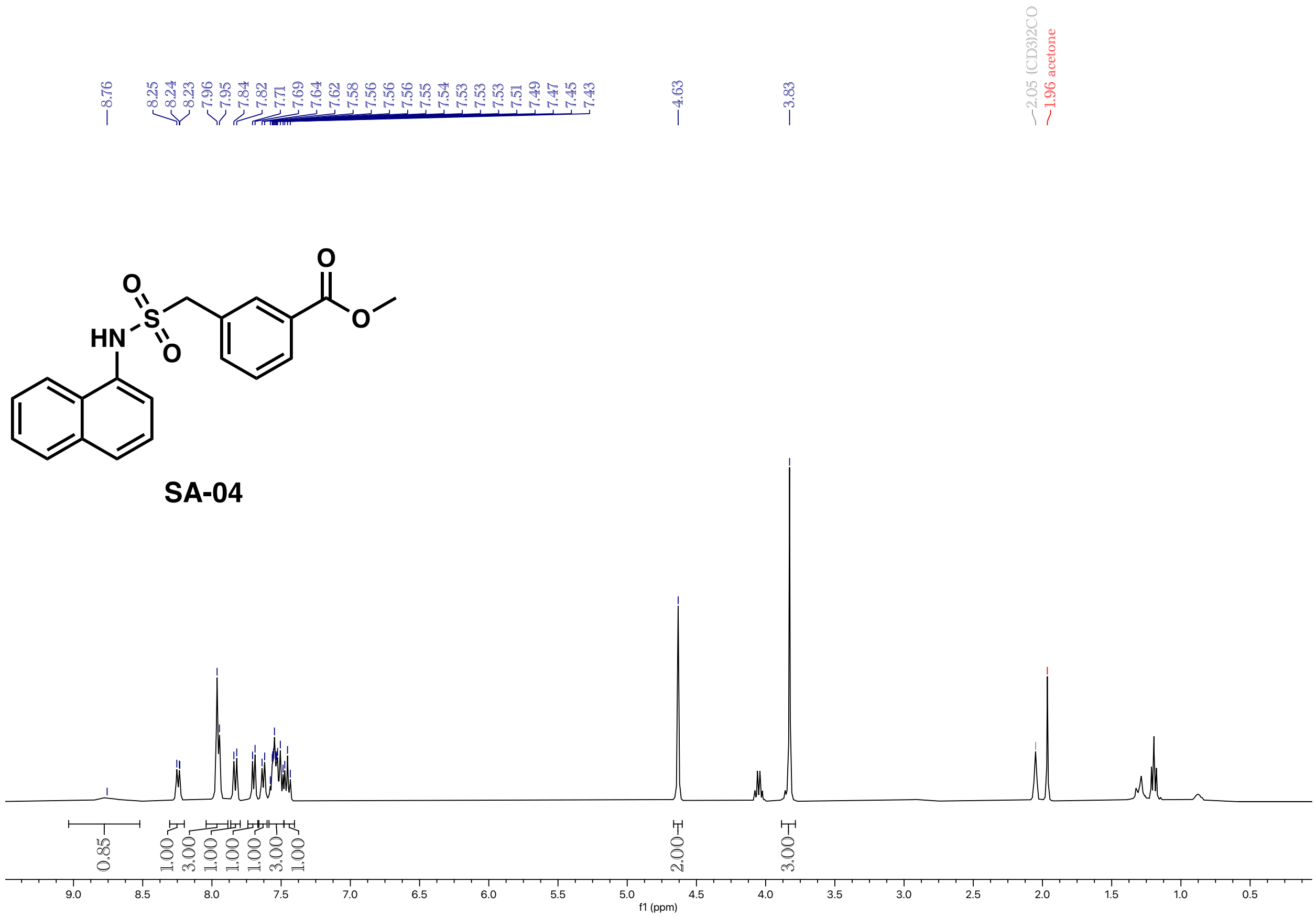

8.76
8.25 8.24 8.23 7.96 7.95 7.84 7.82 7.71 7.69 7.64 7.62 7.58 7.56 7.56 7.56 7.55 7.54 7.53 7.53 7.53 7.51 7.49 7.47 7.45 7.43
4.63
3.83
2.05 (CD3)2CO
1.96 acetone
SA-04
0.85 1.00 3.00 1.00 1.00 1.00 3.00 1.00 2.00 3.00
f1 (ppm)

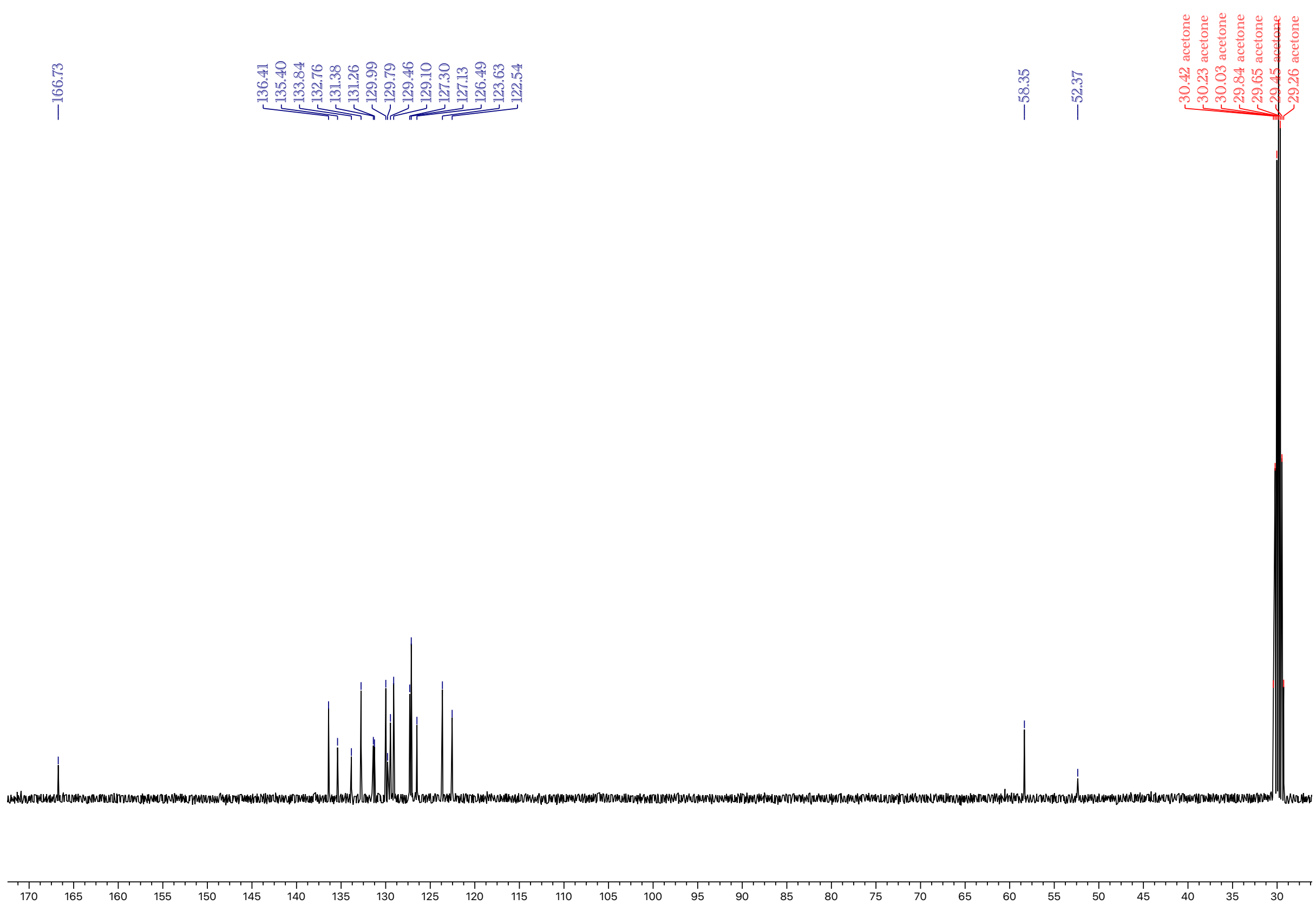

166.73
136.41 135.40 133.84 132.76 131.38 131.26 129.99 129.79 129.46 129.10 127.30 127.13 126.49 123.63 122.54
58.35
52.37
30.42 acetone
30.23 acetone
30.03 acetone
29.84 acetone
29.65 acetone
29.45 acetone
29.26 acetone
f1 (ppm)

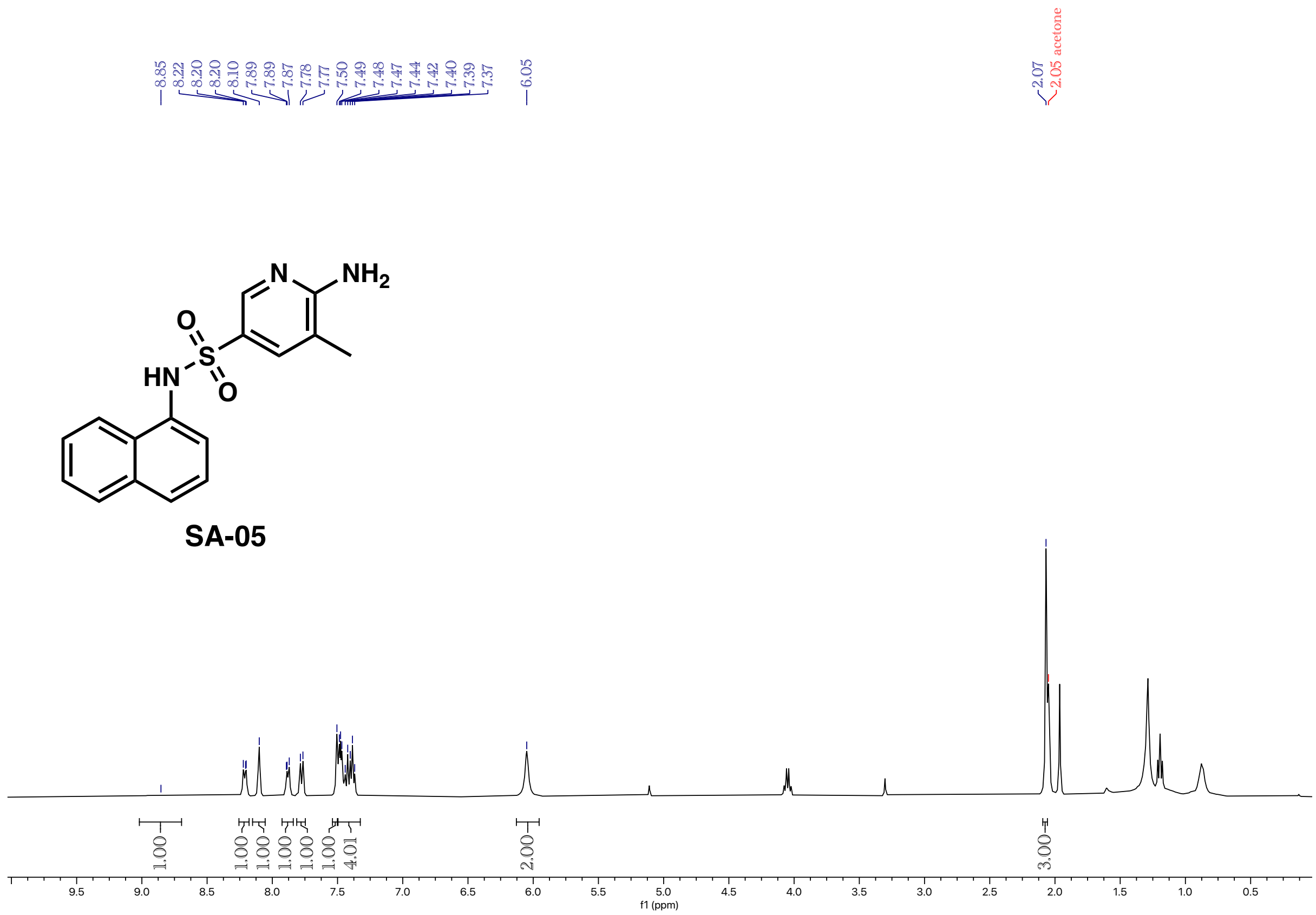
8.85 8.22 8.20 8.20 8.10 7.89 7.89 7.87 7.78 7.77 7.50 7.49 7.48 7.47 7.44 7.42 7.40 7.39 7.37
6.05
2.07
2.05 acetone
SA-05
1.00 1.00 1.00 1.00 1.00 4.01 2.00 3.00
f1 (ppm)

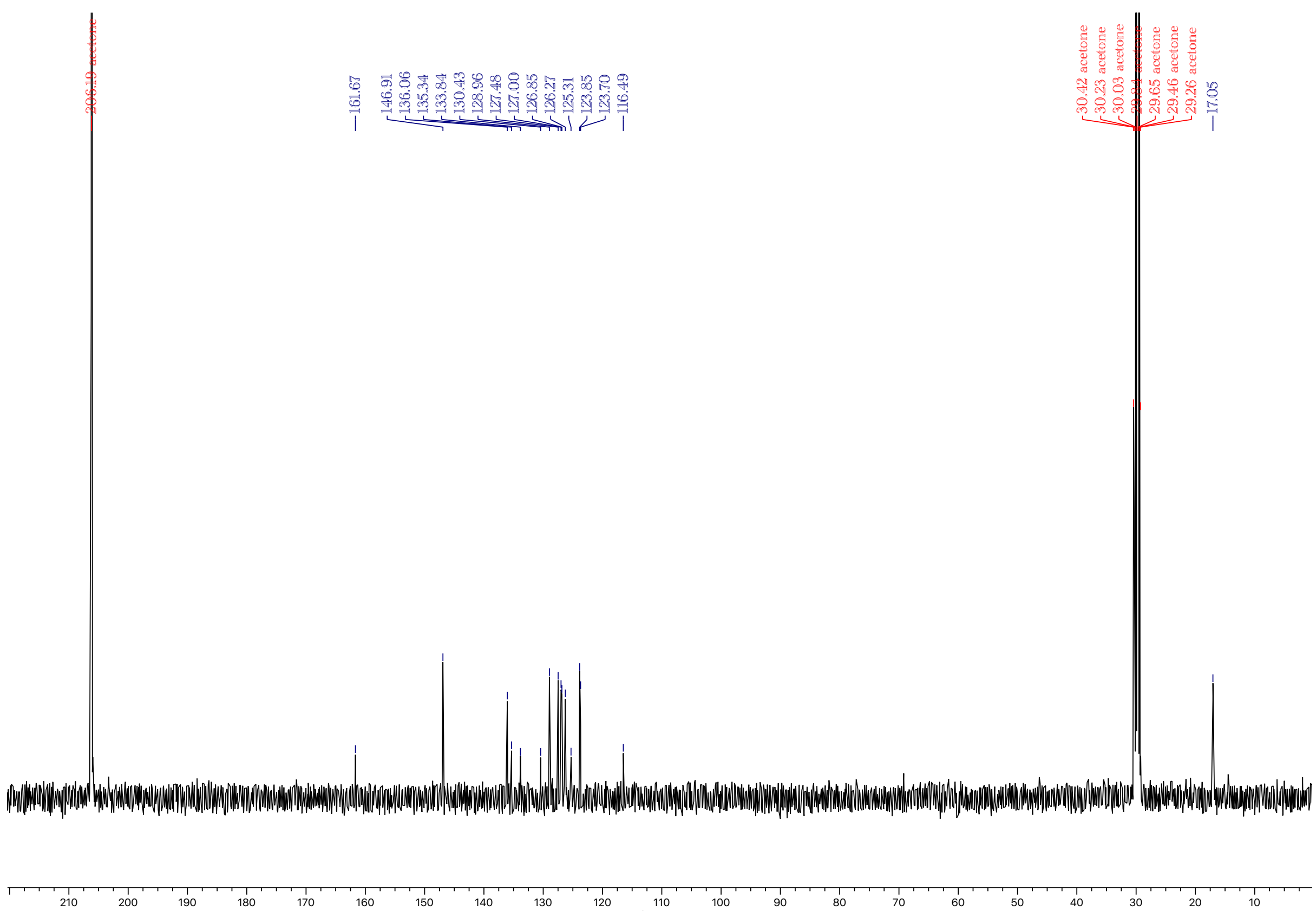
206.10 acetone
161.67
146.91 136.06 135.34 133.84 130.43 128.96 127.48 127.00 126.85 126.27 125.31 123.85 123.70 116.49
30.42 acetone
30.23 acetone
30.03 acetone
29.84 acetone
29.65 acetone
29.46 acetone
29.26 acetone
17.05
f1 (ppm)

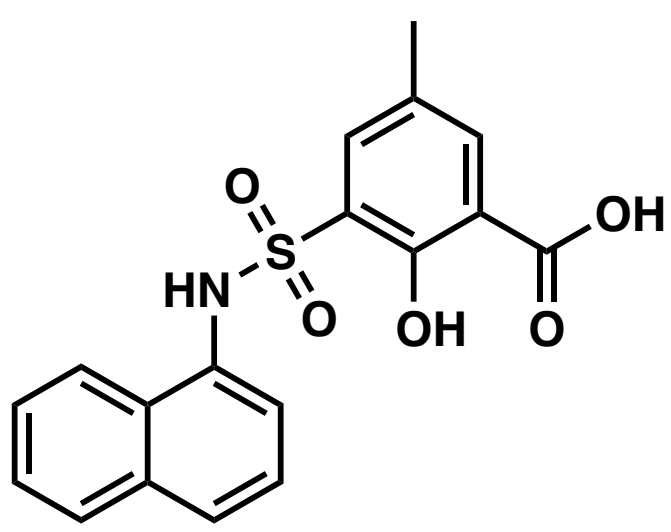
OH
O
HN
S
O
OH
O

SA-06

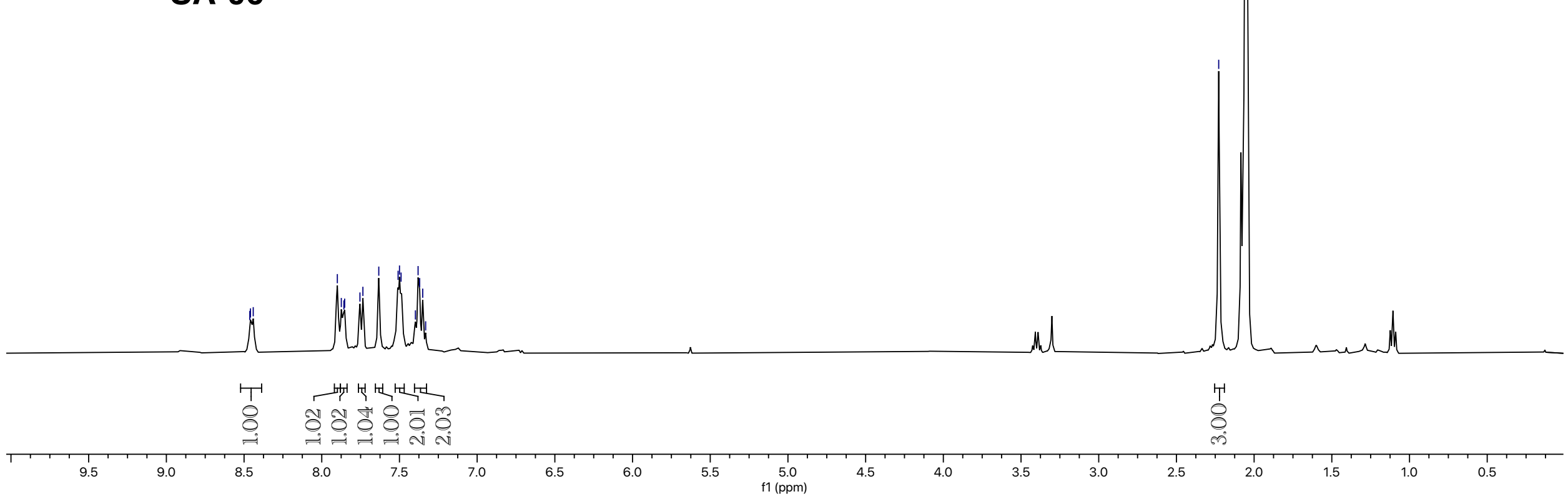
8.46
8.46
8.44
7.90
7.87
7.86
7.85
7.75
7.74
7.63
7.51
7.50
7.49
7.40
7.38
7.37
7.35
7.33
2.23
1.00
1.02
1.02
1.04
1.00
2.01
2.03
3.00
f1 (ppm)

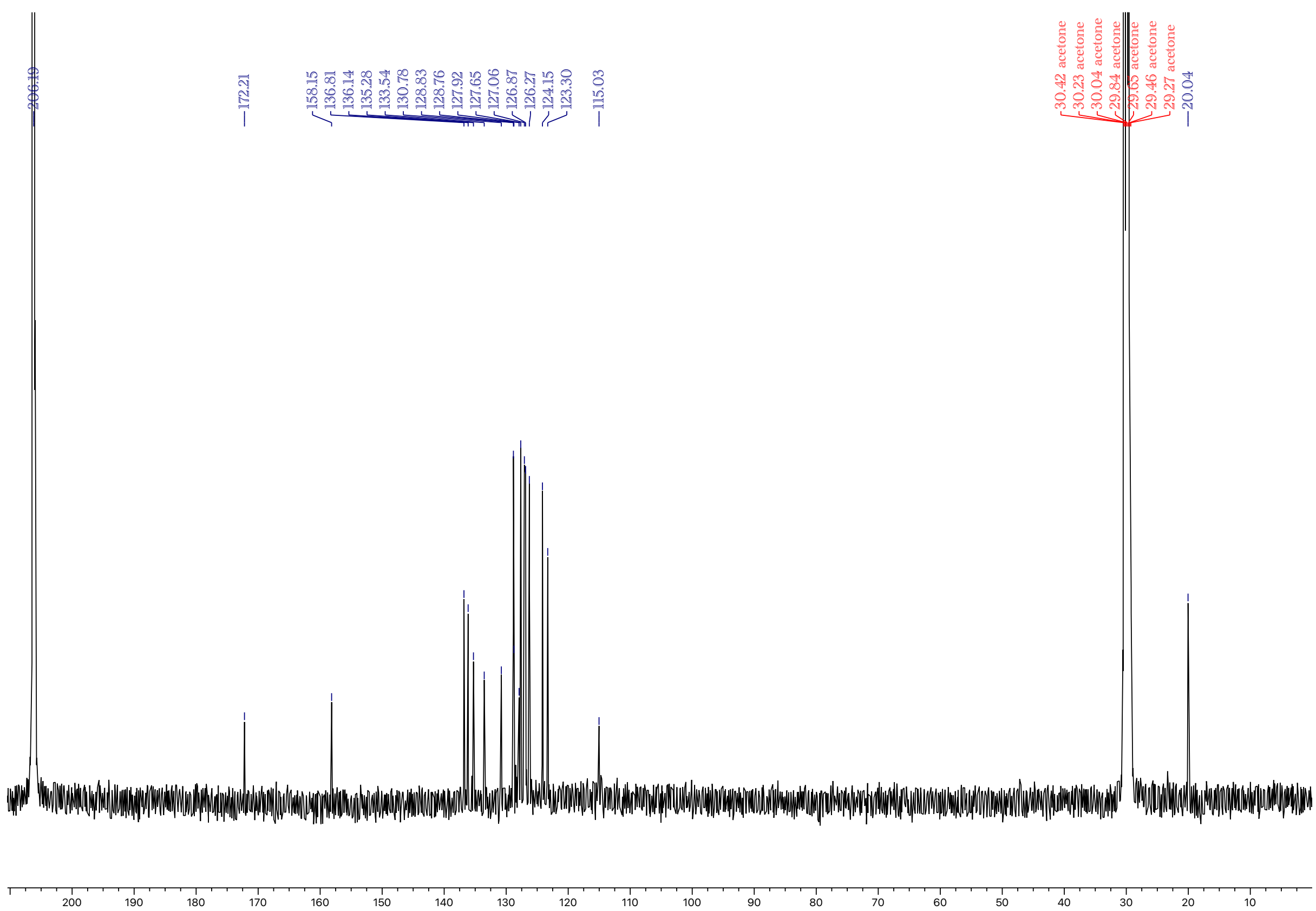
206.10
172.21
158.15
136.81
136.14
135.28
133.54
130.78
128.83
128.76
127.92
127.65
127.06
126.87
126.27
124.15
123.30
115.03
30.42 acetone
30.23 acetone
30.04 acetone
29.84 acetone
29.65 acetone
29.46 acetone
29.27 acetone
20.04
f1 (ppm)

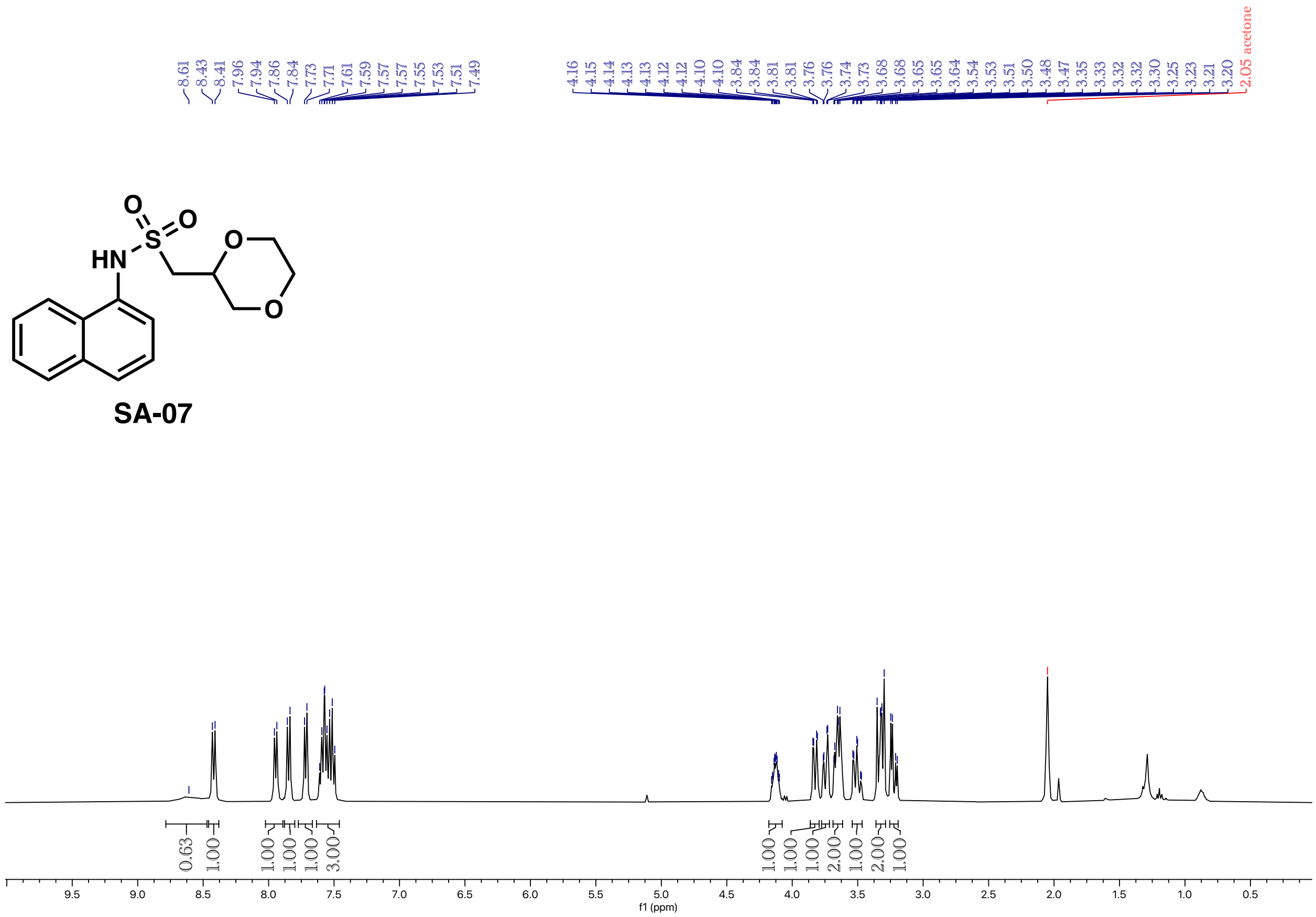

SA-07

8.61
8.43
8.41
7.96
7.94
7.86
7.84
7.73
7.71
7.61
7.59
7.57
7.57
7.55
7.53
7.51
7.49
4.16
4.15
4.14
4.13
4.13
4.12
4.12
4.12
4.10
4.10
3.84
3.84
3.81
3.81
3.76
3.76
3.74
3.73
3.68
3.68
3.65
3.65
3.64
3.54
3.53
3.51
3.50
3.48
3.47
3.35
3.33
3.32
3.32
3.30
3.25
3.23
3.21
3.20
2.05 acetone
0.63
1.00
1.00
1.00
1.00
3.00
1.00
1.00
2.00
1.00
2.00
1.00
f1 (ppm)

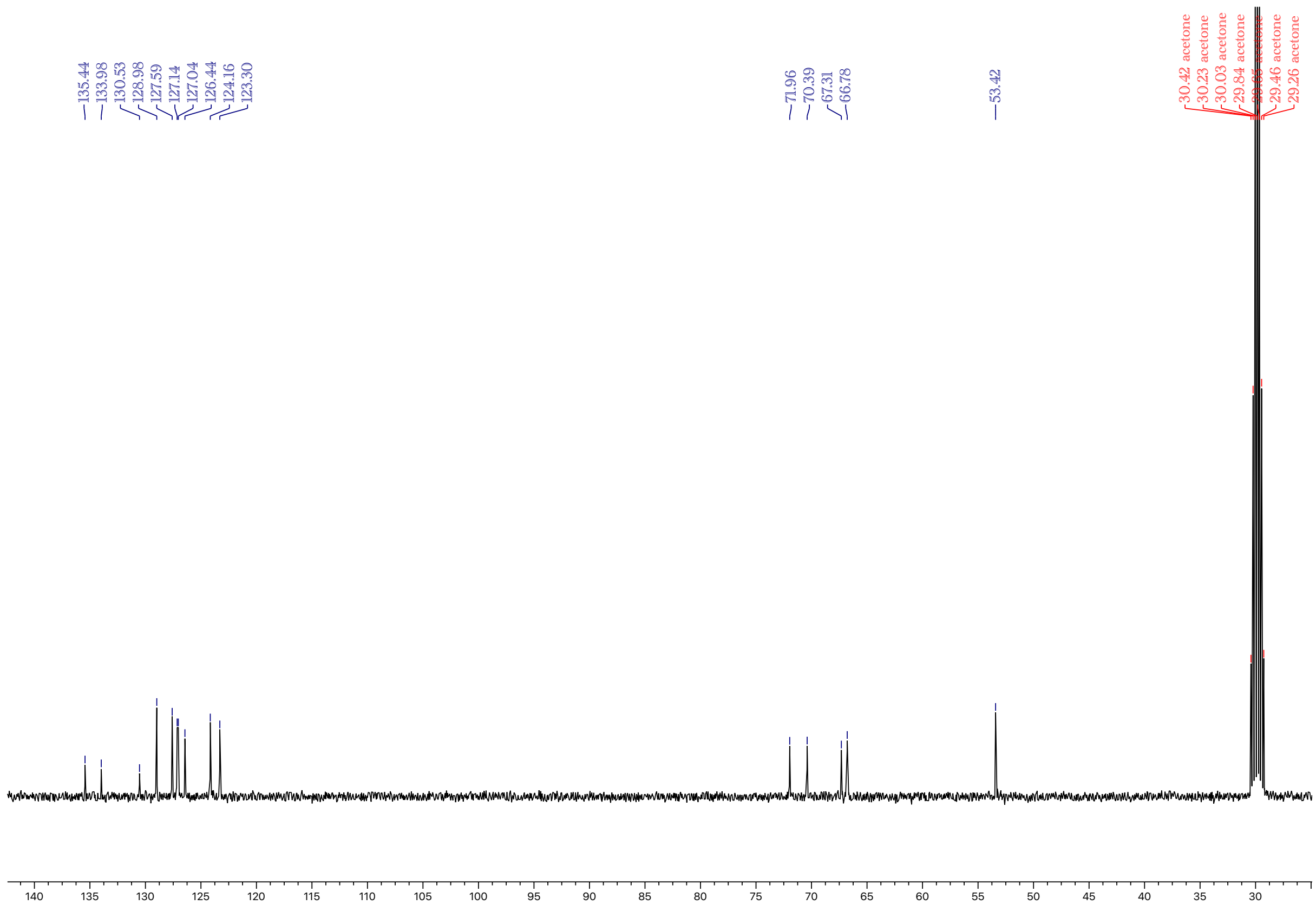
135.44
133.98
130.53
128.98
127.59
127.14
127.04
126.44
124.16
123.30
71.96
70.39
67.31
66.78
53.42
30.42 acetone
30.23 acetone
30.03 acetone
29.84 acetone
29.46 acetone
29.26 acetone
f1 (ppm)

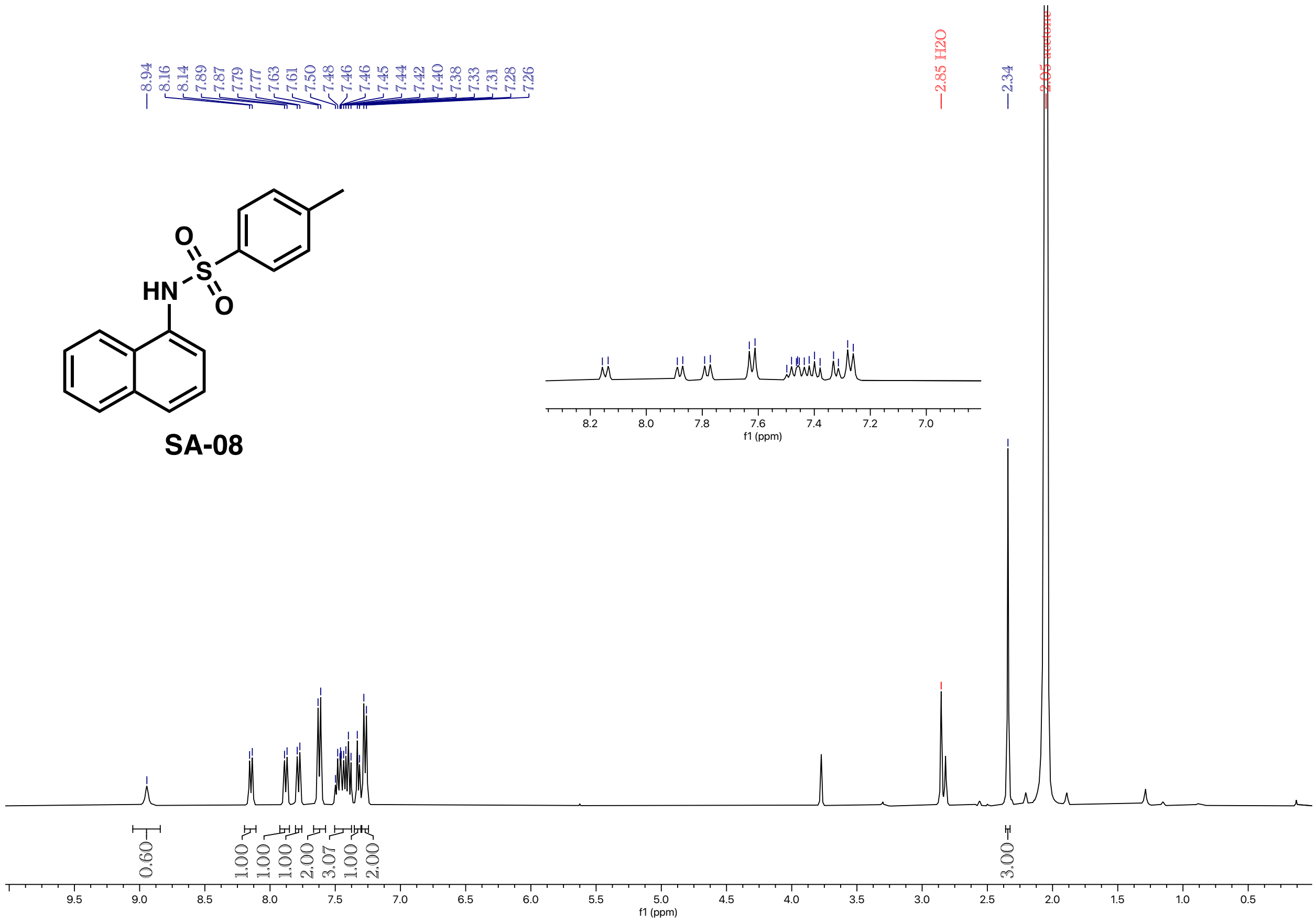

SA-08

8.94 8.16 8.14 7.89 7.87 7.79 7.77 7.63 7.61 7.50 7.48 7.46 7.46 7.45 7.44 7.42 7.40 7.38 7.33 7.31 7.28 7.26
2.85 H2O
2.34
2.05 acetone
0.60 1.00 1.00 1.00 2.00 3.07 1.00 2.00 3.00
f1 (ppm)

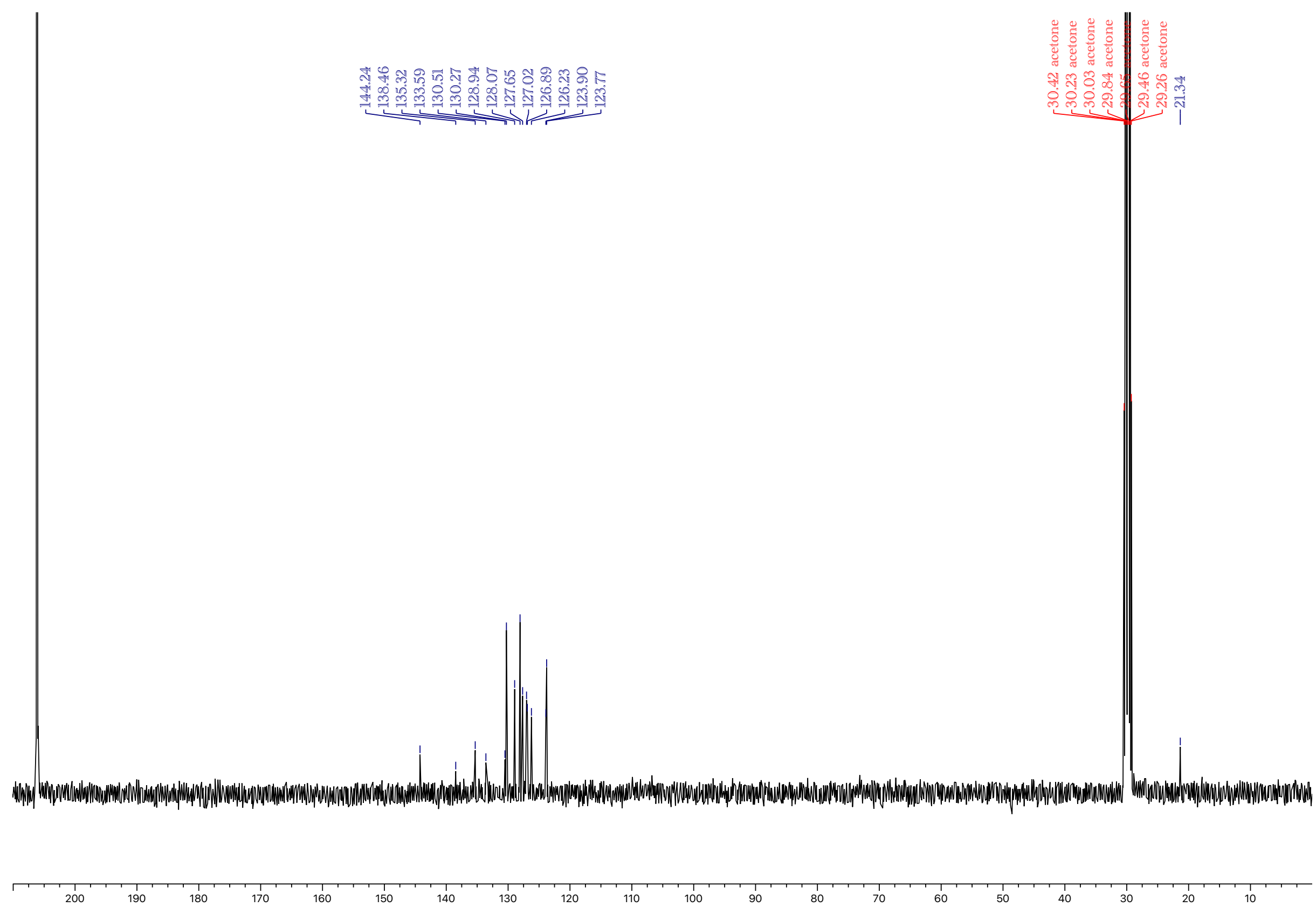
144.24 138.46 135.32 133.59 130.51 130.27 128.94 128.07 127.65 127.02 126.89 126.23 123.90 123.77
30.42 acetone
30.23 acetone
30.03 acetone
29.84 acetone
29.46 acetone
29.26 acetone
21.34
f1 (ppm)

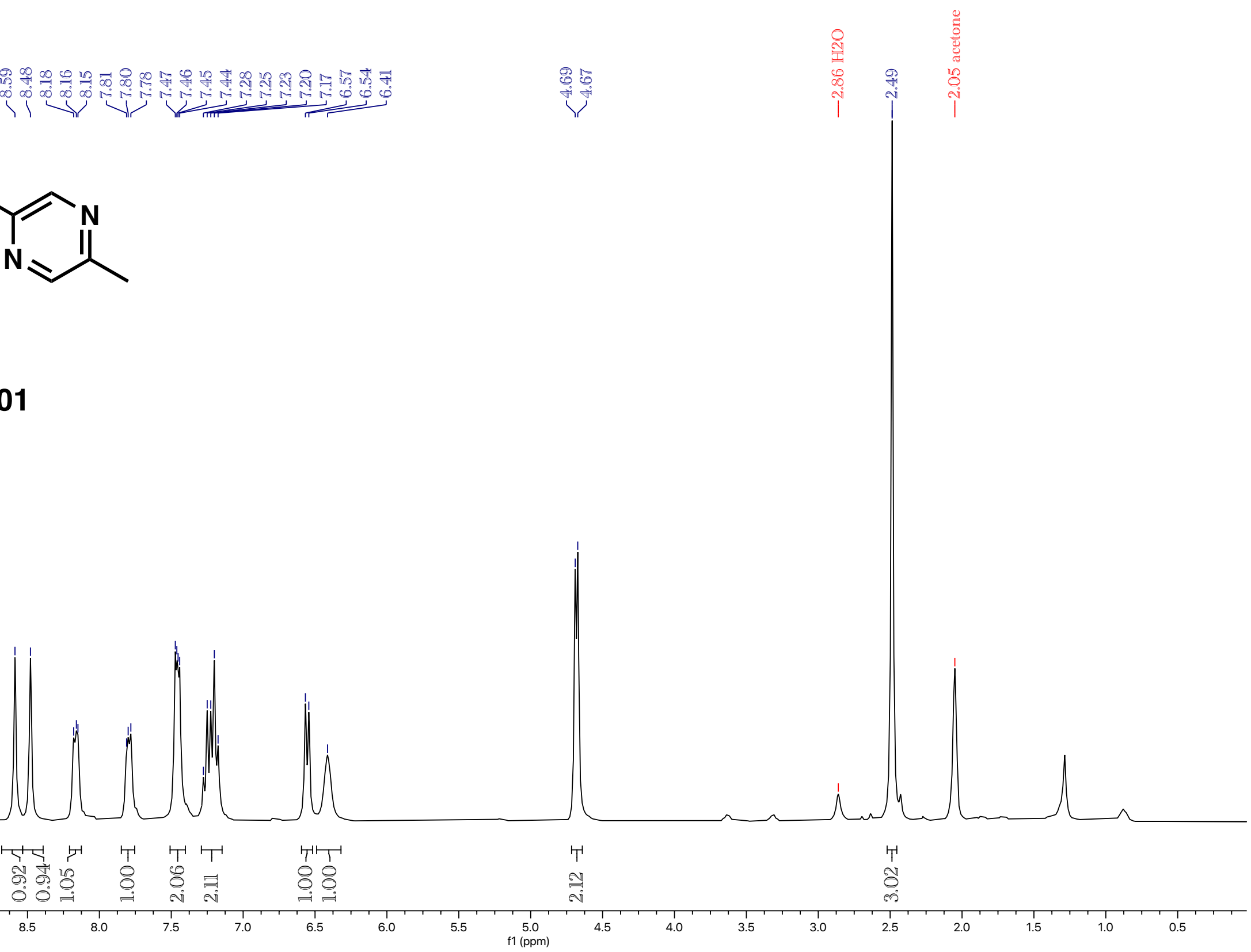

8.59
8.48
8.18
8.16
8.15
7.81
7.80
7.78
7.47
7.46
7.45
7.44
7.28
7.25
7.23
7.20
7.17
6.57
6.54
6.41
4.69
4.67
2.86 H2O
2.49
2.05 acetone
0.92
0.94
1.05
1.00
2.06
2.11
1.00
1.00
2.12
3.02
f1 (ppm)

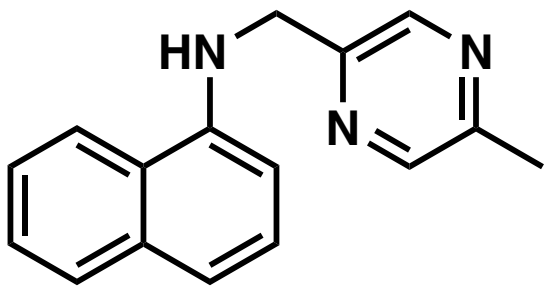

HN
N
N

ALK-01

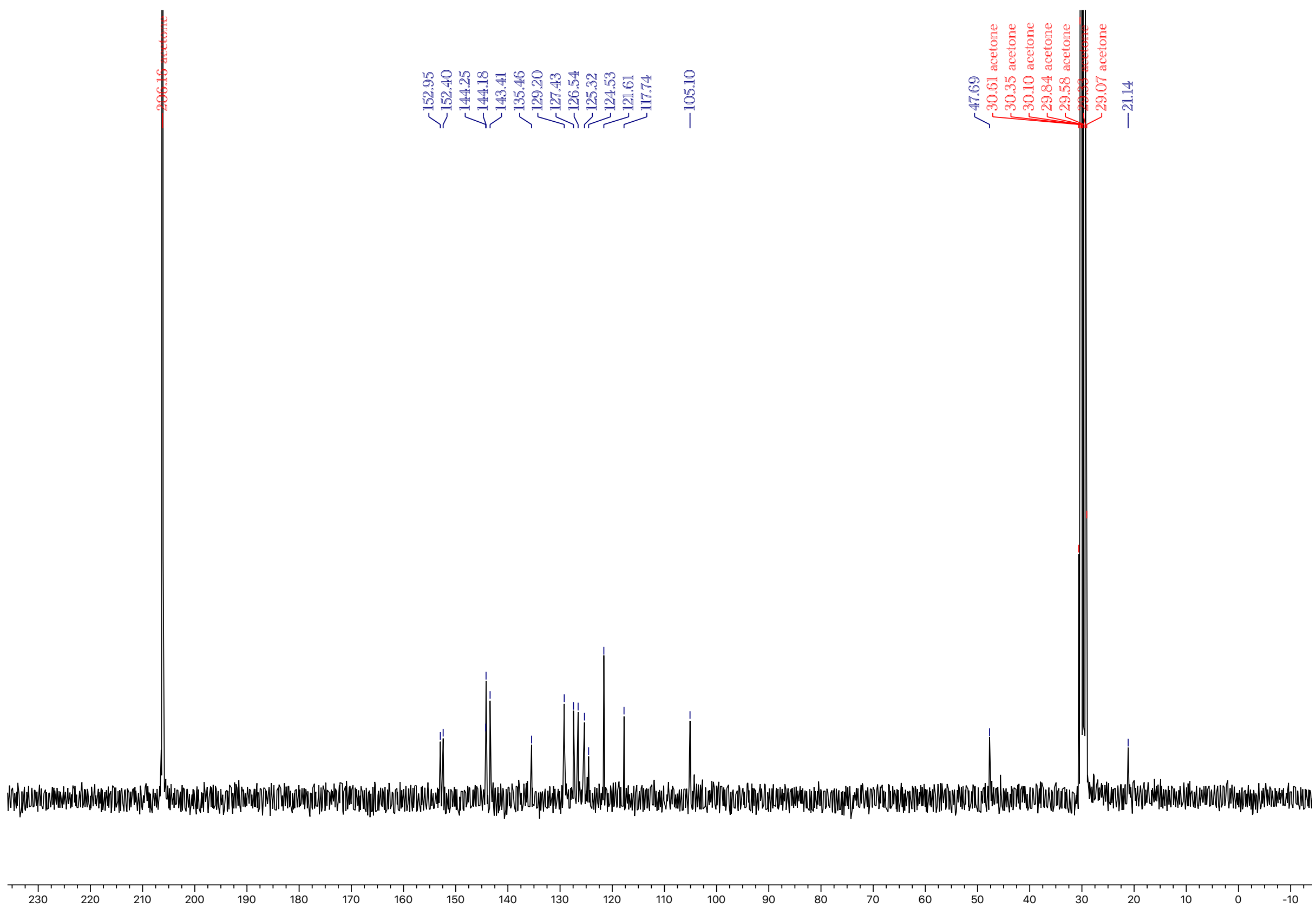

206.16 acetone
152.95
152.40
144.25
144.18
143.41
135.46
129.20
127.43
126.54
125.32
124.53
121.61
117.74
105.10
47.69
30.61 acetone
30.35 acetone
30.10 acetone
29.84 acetone
29.58 acetone
29.07 acetone
21.14
f1 (ppm)

ALK-02

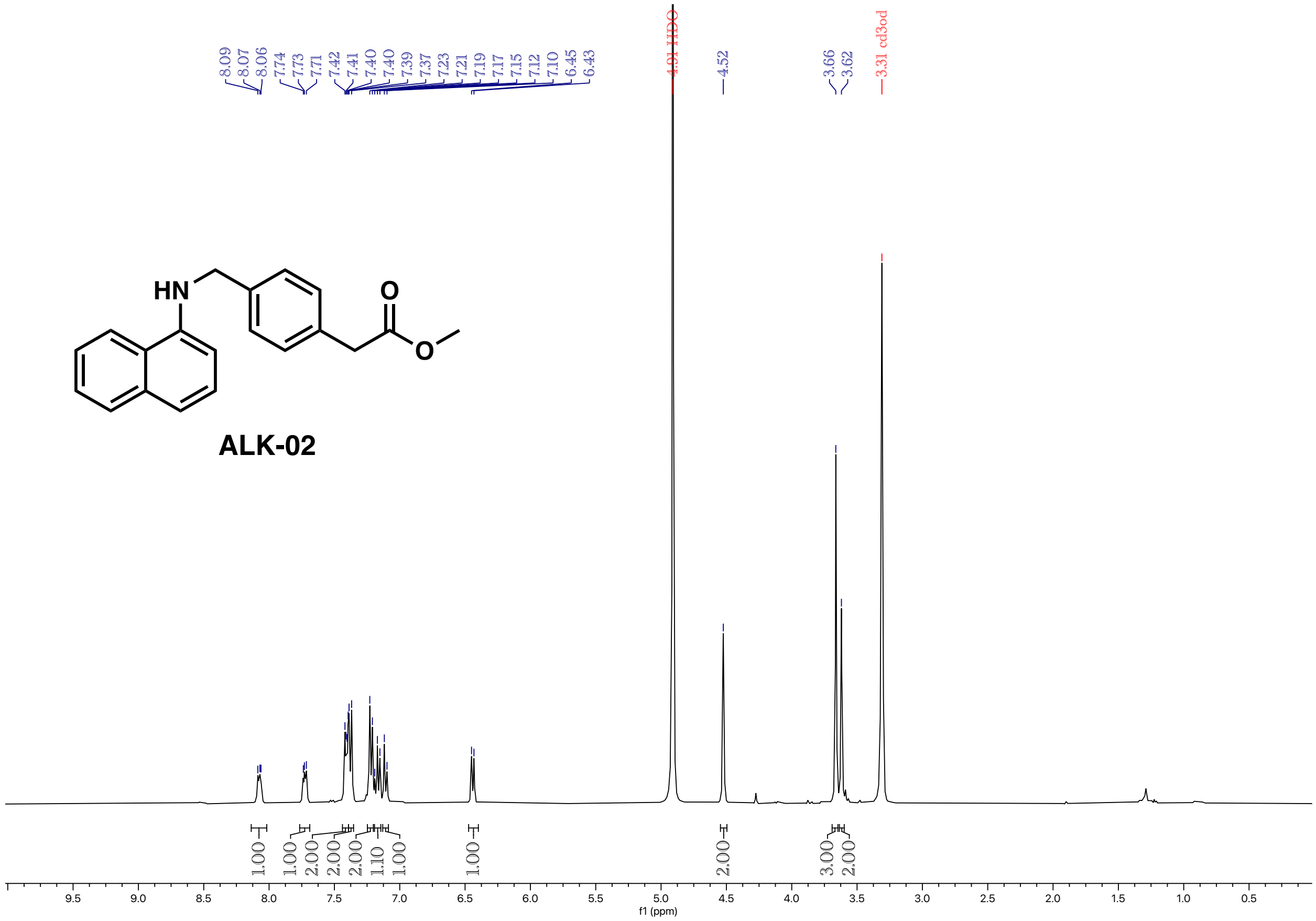

8.09 8.07 8.06 7.74 7.73 7.71 7.42 7.41 7.40 7.40 7.39 7.37 7.23 7.21 7.19 7.17 7.15 7.12 7.10 6.45 6.43
4.91 HDO
4.52
3.66 3.62
3.31 cd3od
1.00 1.00 2.00 2.00 2.00 1.10 1.00 1.00 2.00 3.00 2.00
f1 (ppm)

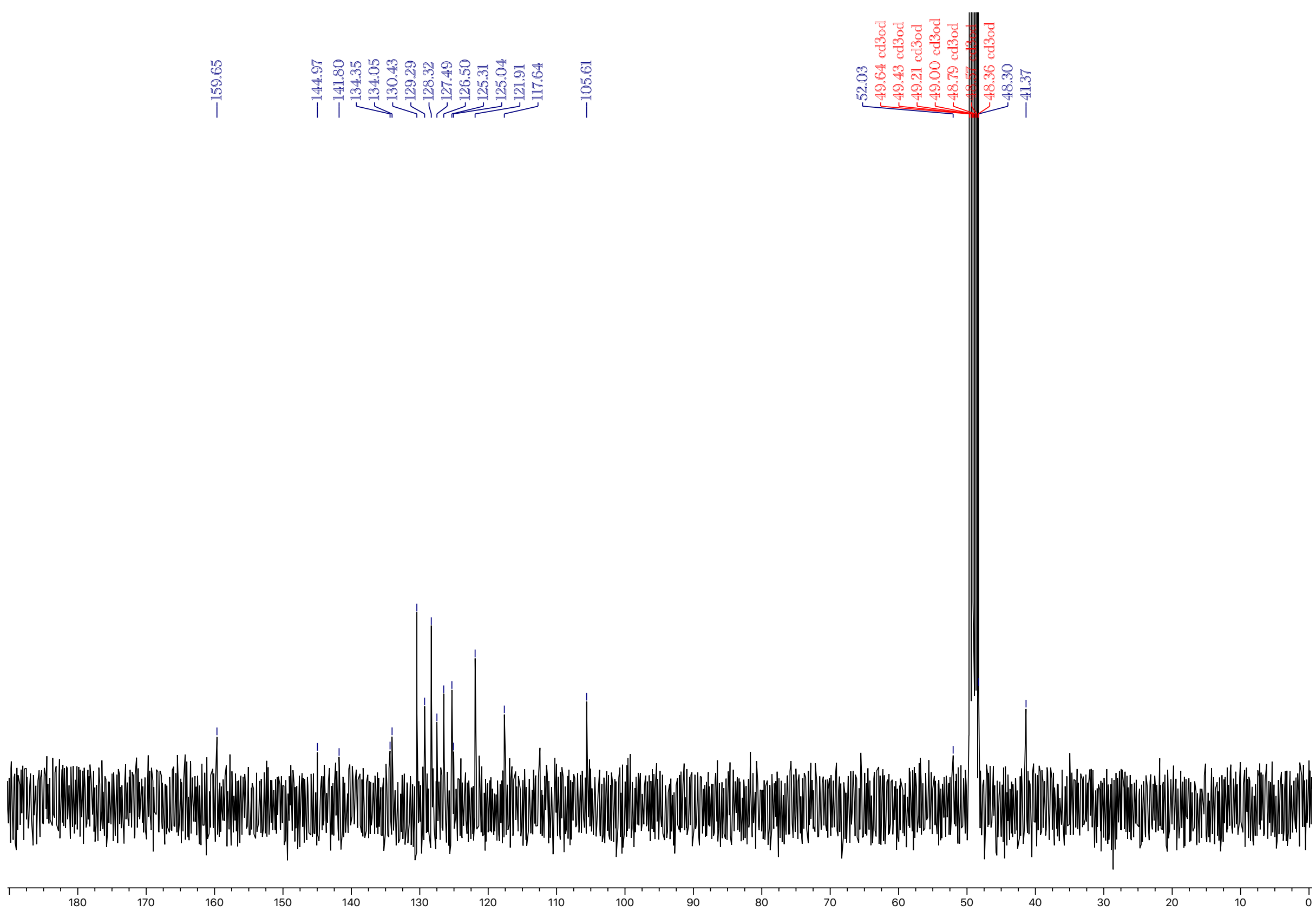

159.65
144.97 141.80 134.35 134.05 130.43 129.29 128.32 127.49 126.50 125.31 125.04 121.91 117.64
105.61
52.03 49.64 cd3od 49.43 cd3od 49.21 cd3od 49.00 cd3od 48.79 cd3od 48.57 cd3od 48.36 cd3od 48.30 41.37
f1 (ppm)

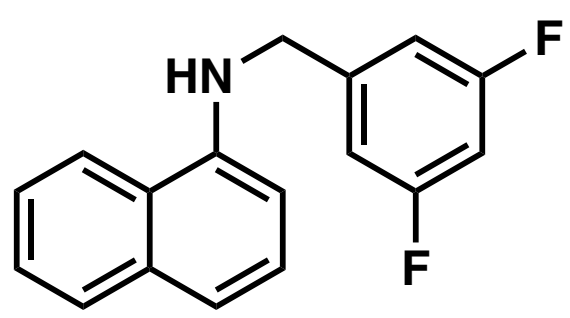
HN
F
F

ALK-03

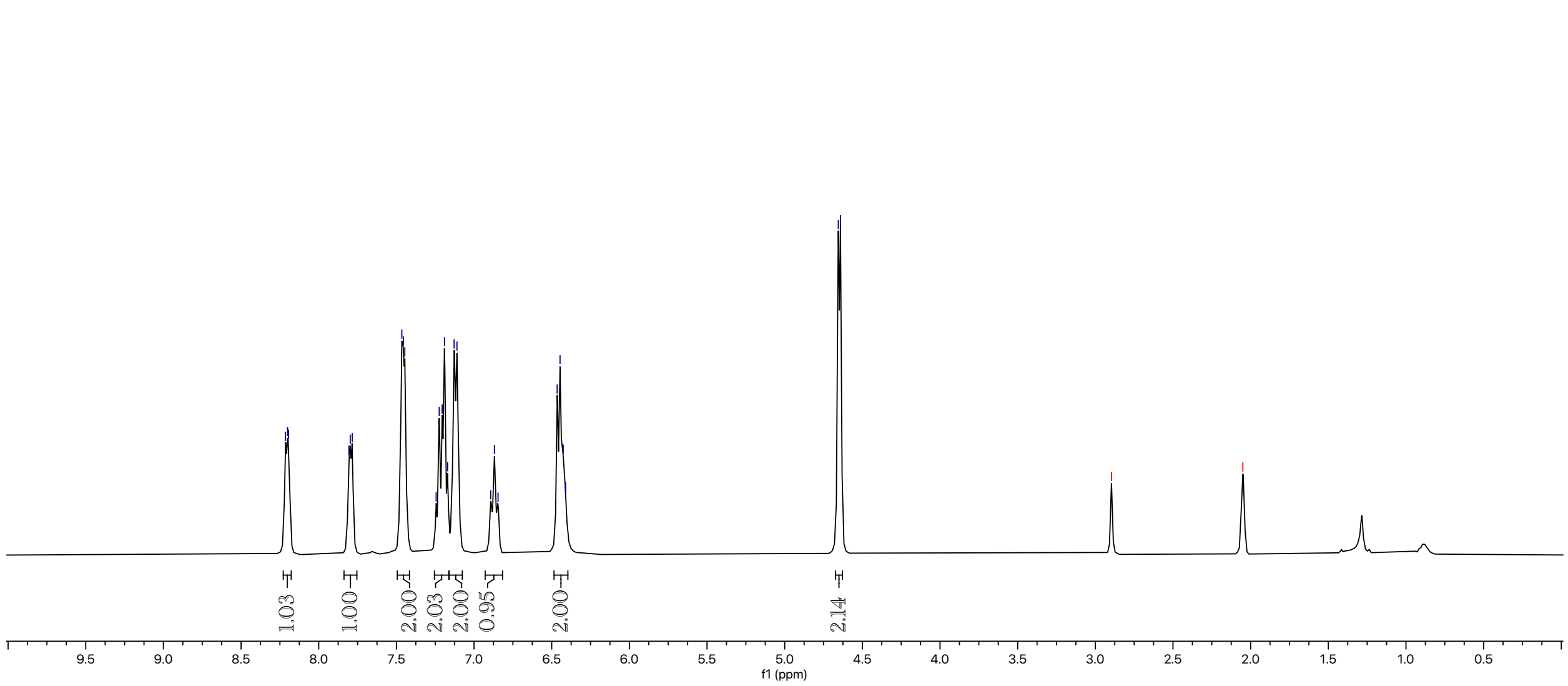
8.21
8.20
8.19
7.81
7.80
7.78
7.47
7.46
7.45
7.24
7.22
7.21
7.19
7.17
7.13
7.11
6.89
6.87
6.85
6.46
6.45
6.43
6.41
4.65
4.64
2.90 H2O
2.05 acetone
1.03
1.00
2.00
2.03
2.00
0.95
2.00
2.14
9.5
9.0
8.5
8.0
7.5
7.0
6.5
6.0
5.5
5.0
4.5
4.0
3.5
3.0
2.5
2.0
1.5
1.0
0.5
f1 (ppm)

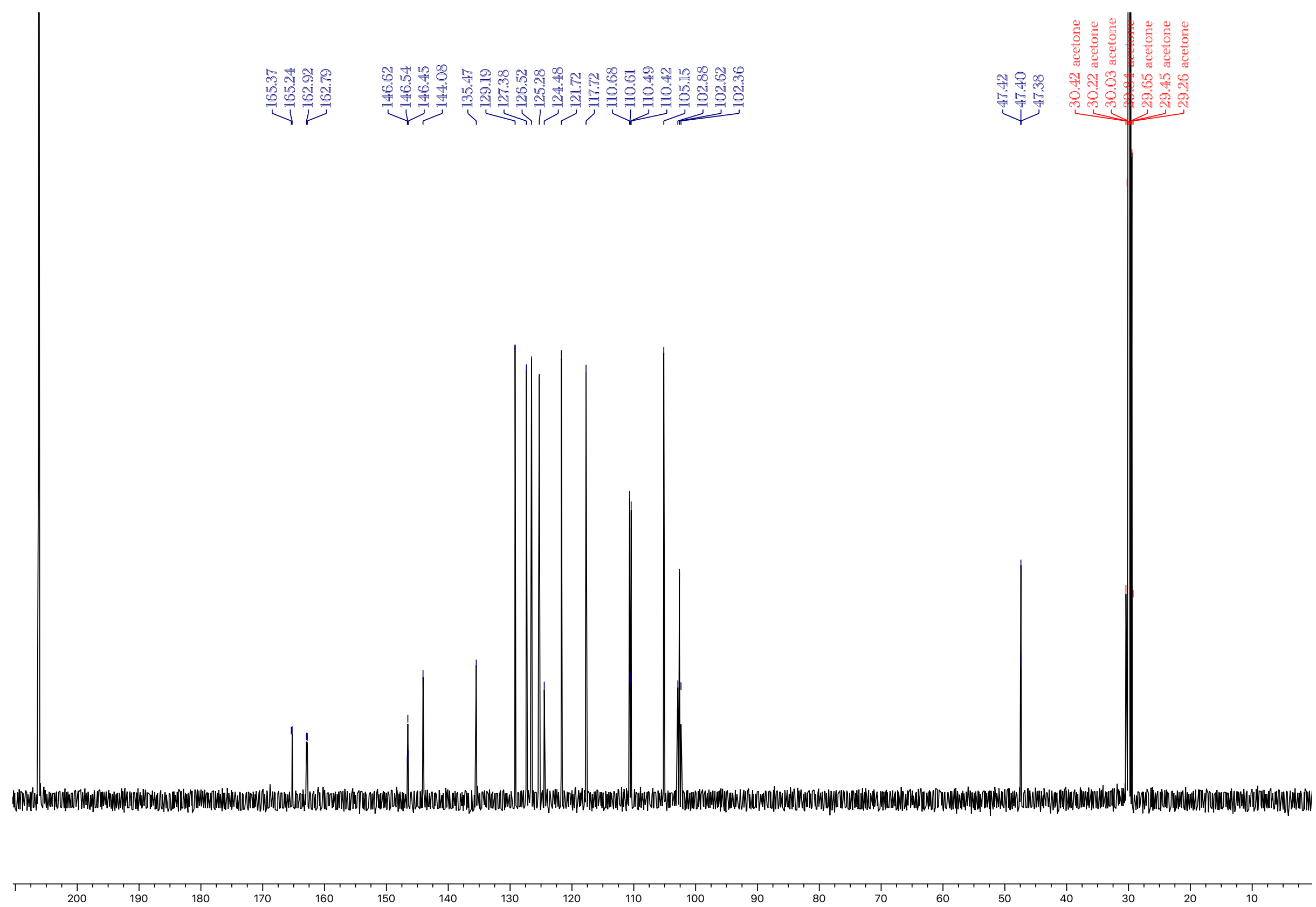
165.37
165.24
162.92
162.79
146.62
146.54
146.45
144.08
135.47
129.19
127.38
126.52
125.28
124.48
121.72
117.72
110.68
110.61
110.49
110.42
105.15
102.88
102.62
102.36
47.42
47.40
47.38
30.42 acetone
30.22 acetone
30.03 acetone
29.65 acetone
29.45 acetone
29.26 acetone
200
190
180
170
160
150
140
130
120
110
100
90
80
70
60
50
40
30
20
10
f1 (ppm)

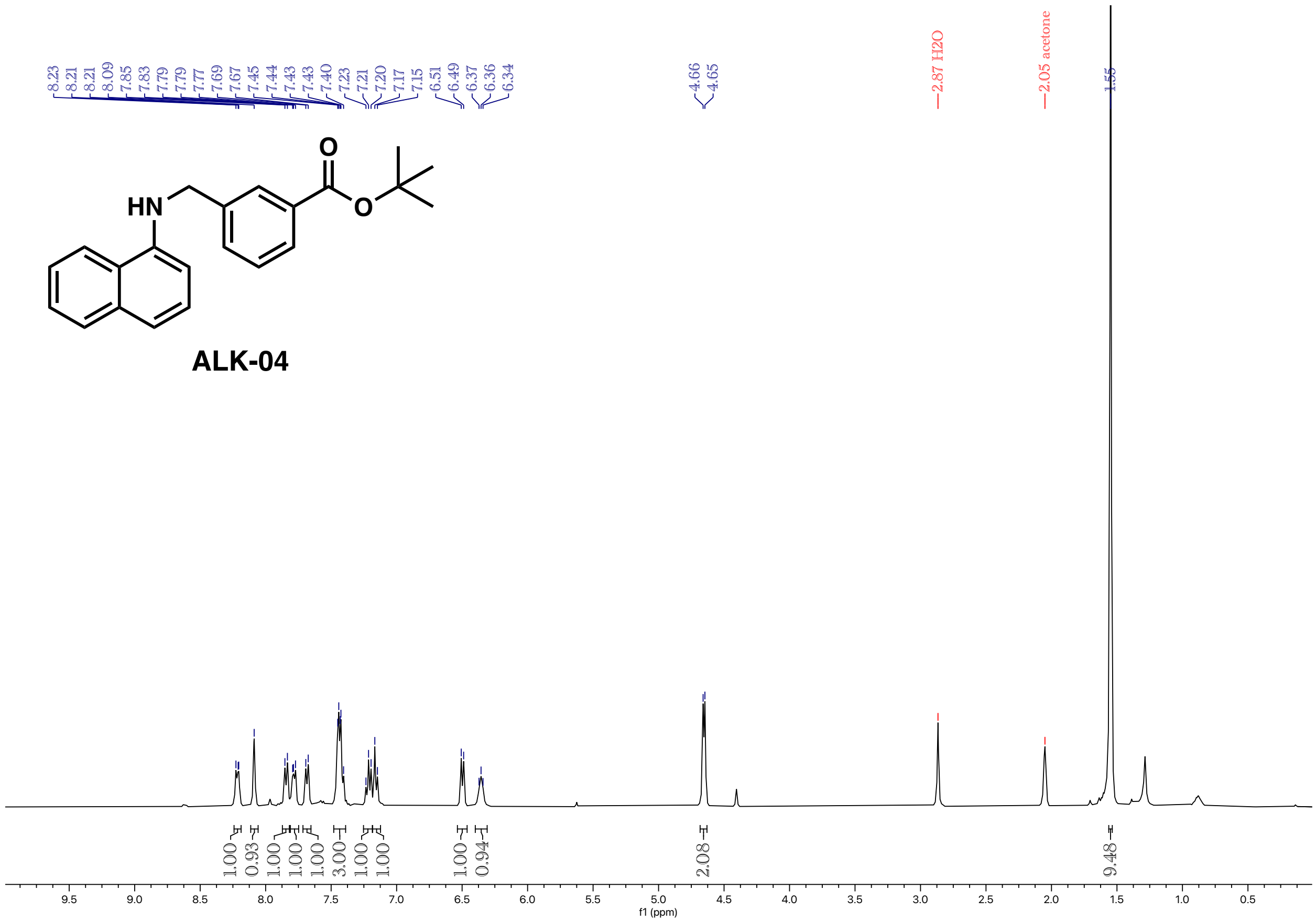
8.23
8.21
8.21
8.09
7.85
7.83
7.79
7.79
7.77
7.69
7.67
7.45
7.44
7.43
7.43
7.40
7.23
7.21
7.20
7.17
7.15
6.51
6.49
6.37
6.36
6.34
4.66
4.65
2.87 H2O
2.05 acetone
1.55
1.00
0.93
1.00
1.00
1.00
3.00
1.00
1.00
1.00
0.94
2.08
9.48
f1 (ppm)

ALK-04

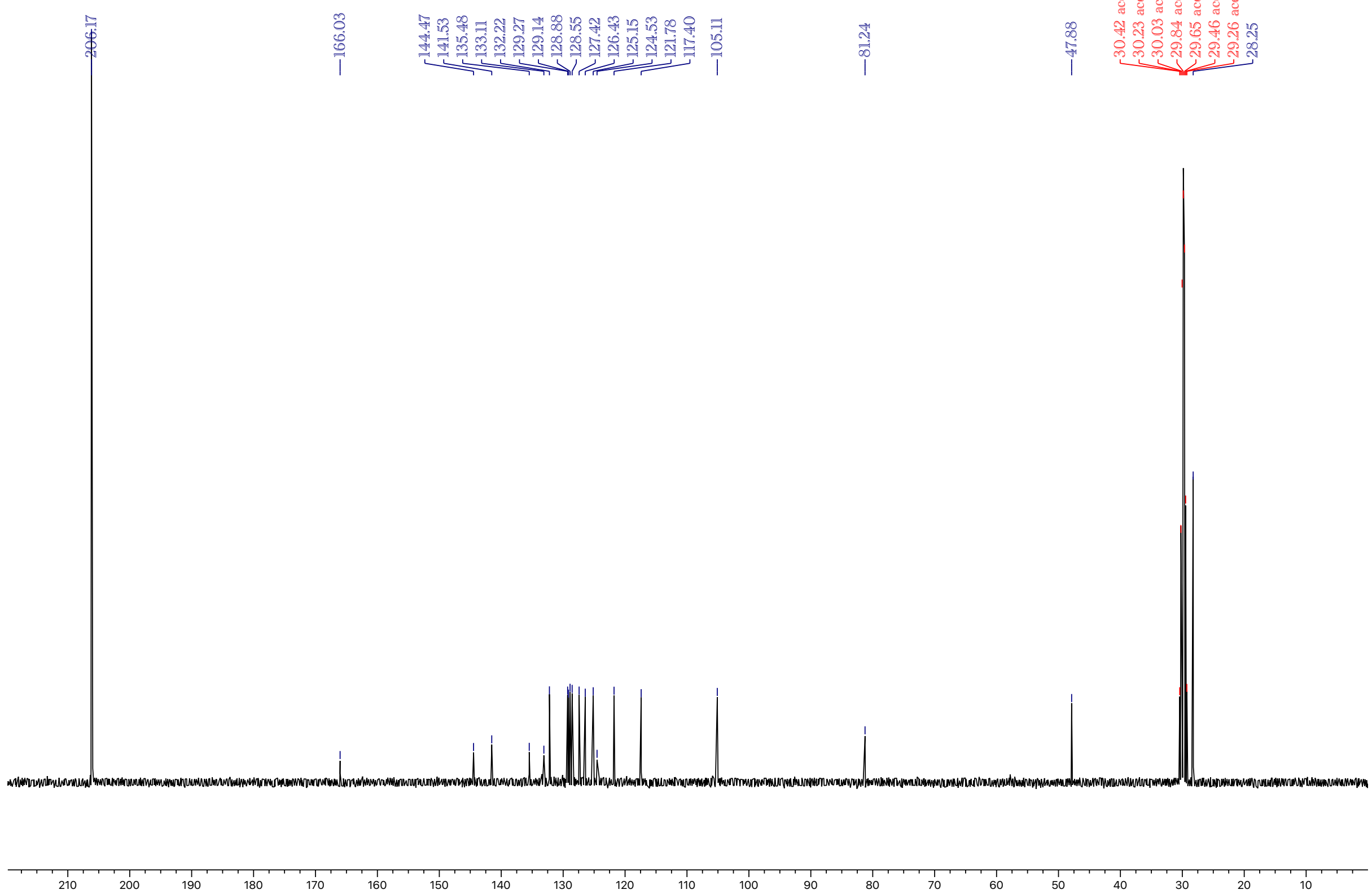
206.17
166.03
144.47
141.53
135.48
133.11
132.22
129.27
129.14
128.88
128.55
127.42
126.43
125.15
124.53
121.78
117.40
105.11
81.24
47.88
30.42 acetone
30.23 acetone
30.03 acetone
29.84 acetone
29.65 acetone
29.46 acetone
29.26 acetone
28.25
f1 (ppm)

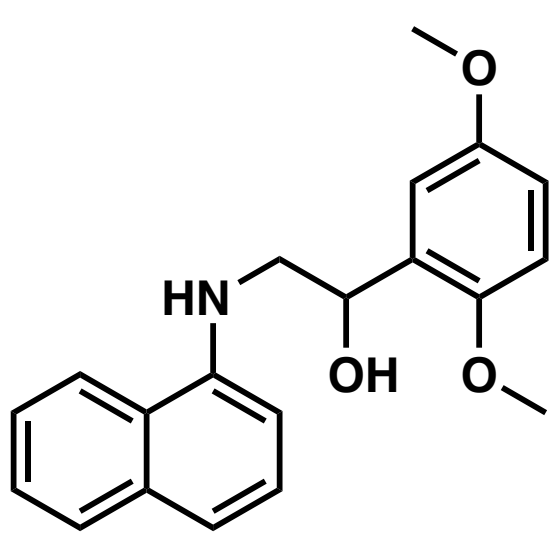

ALK-05

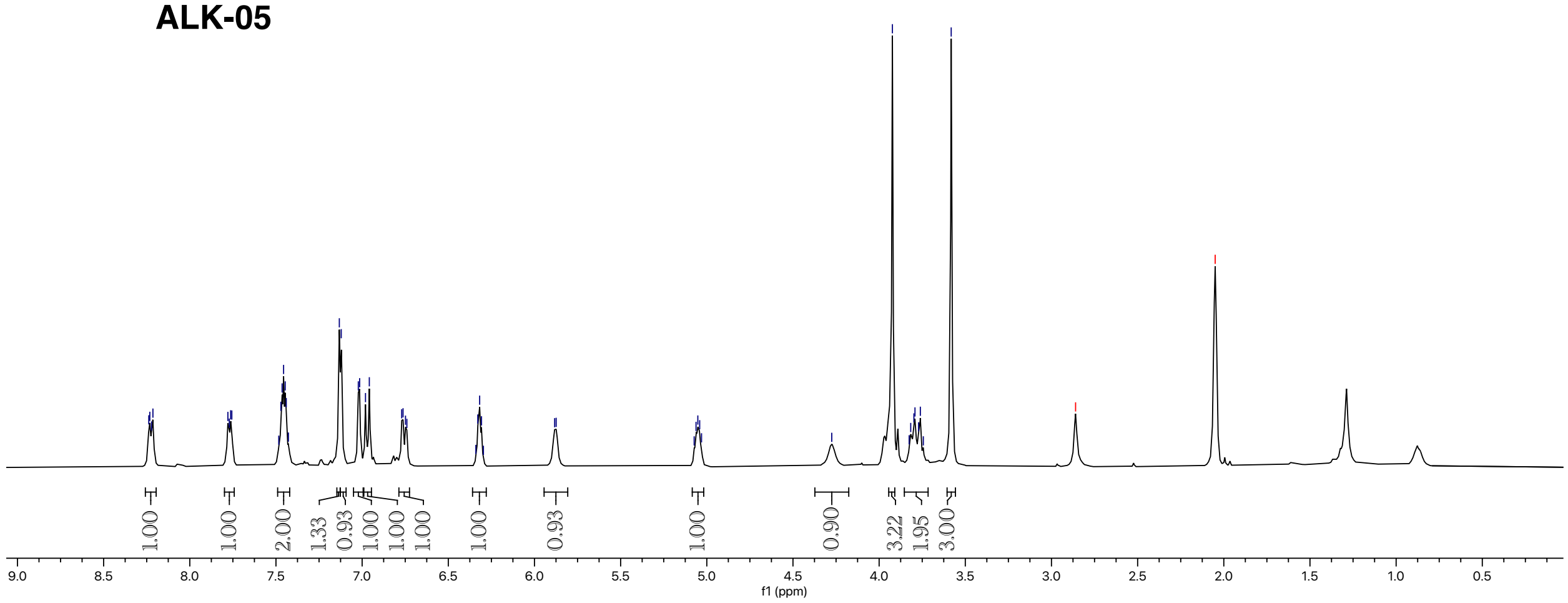

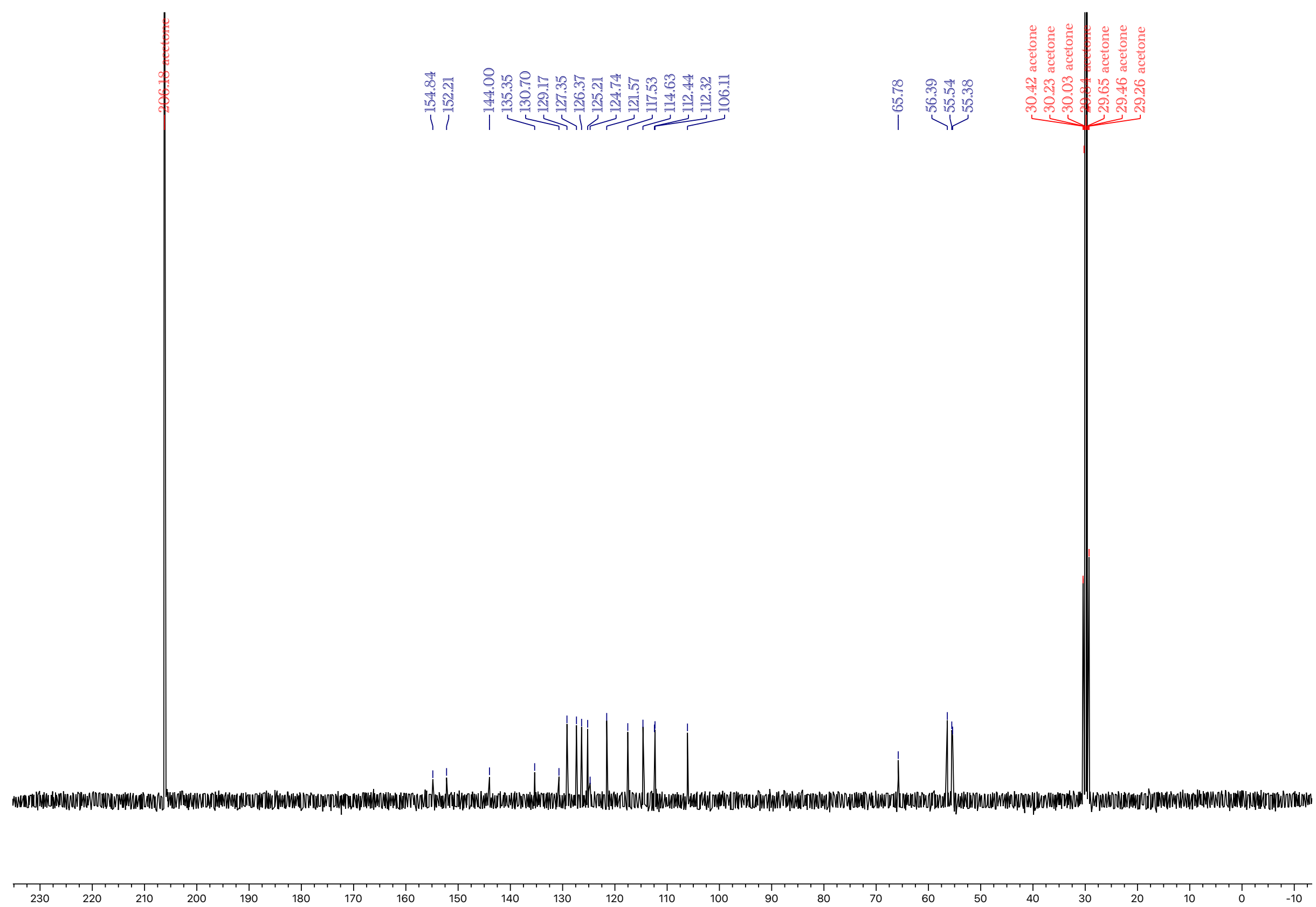

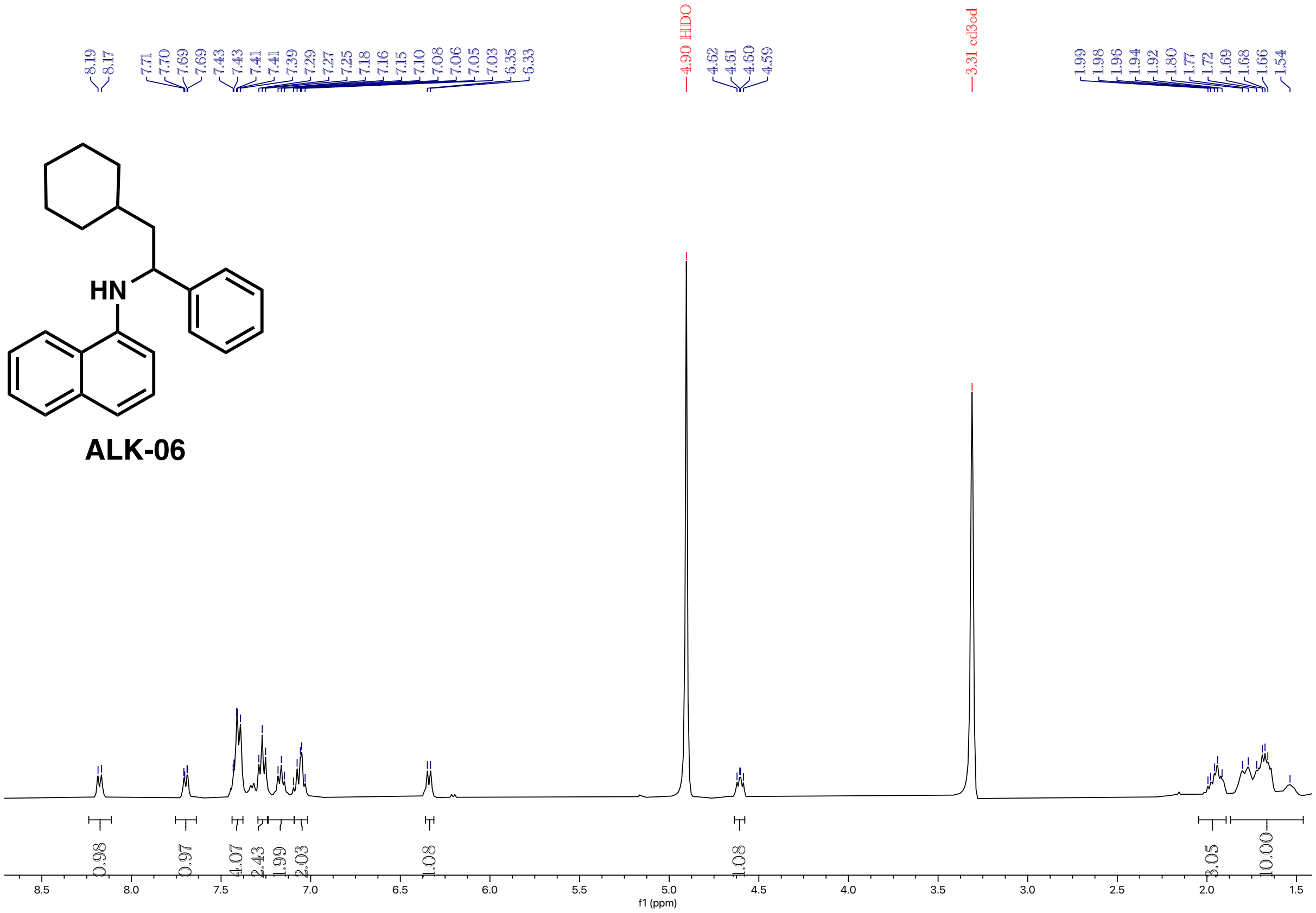

ALK-06

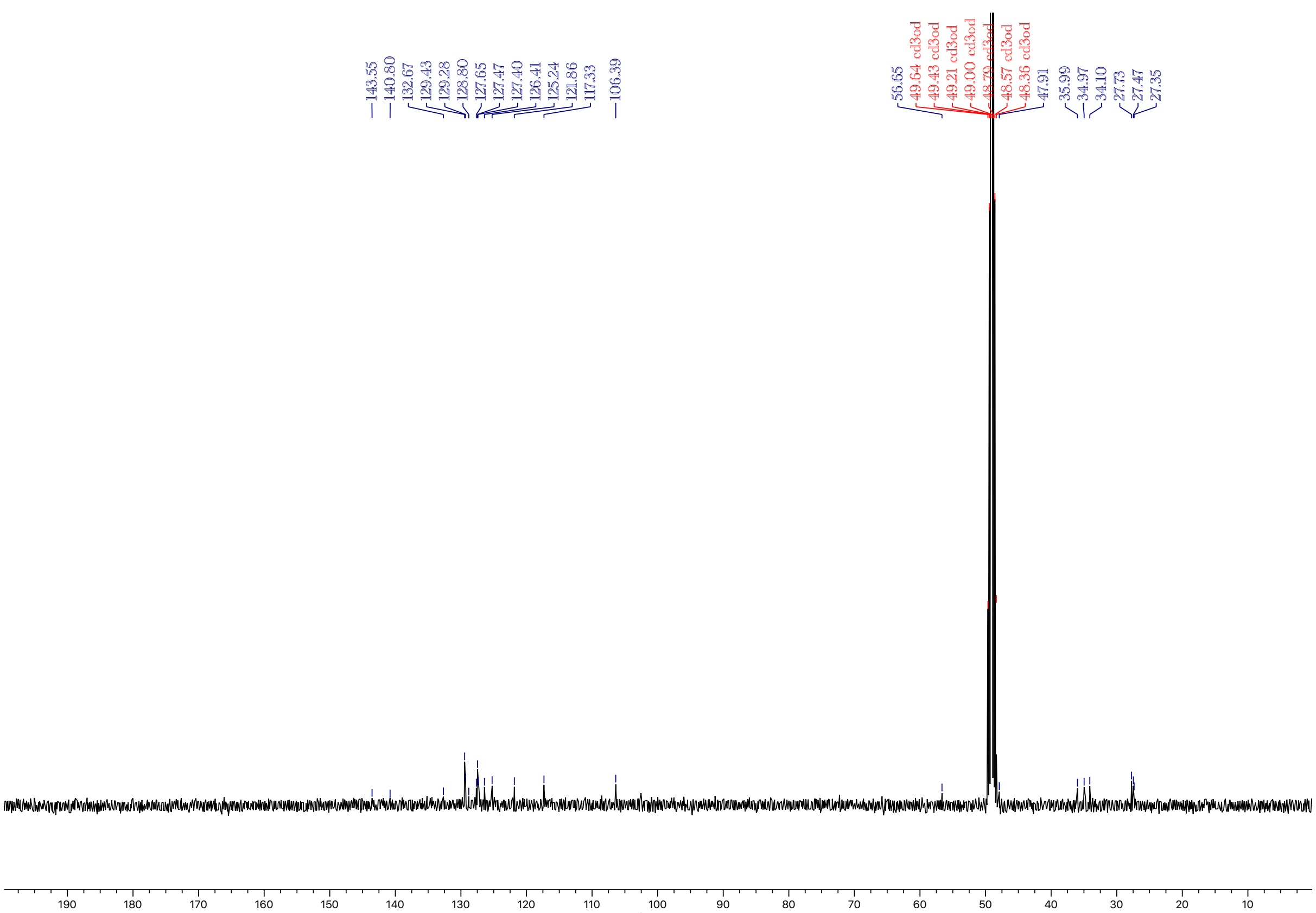

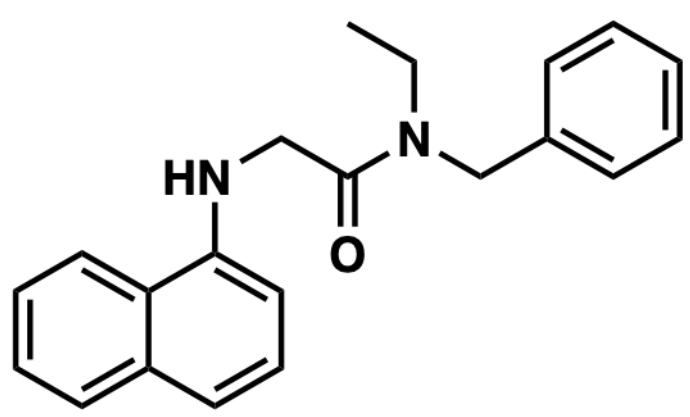
HN
N
O

ALK-07

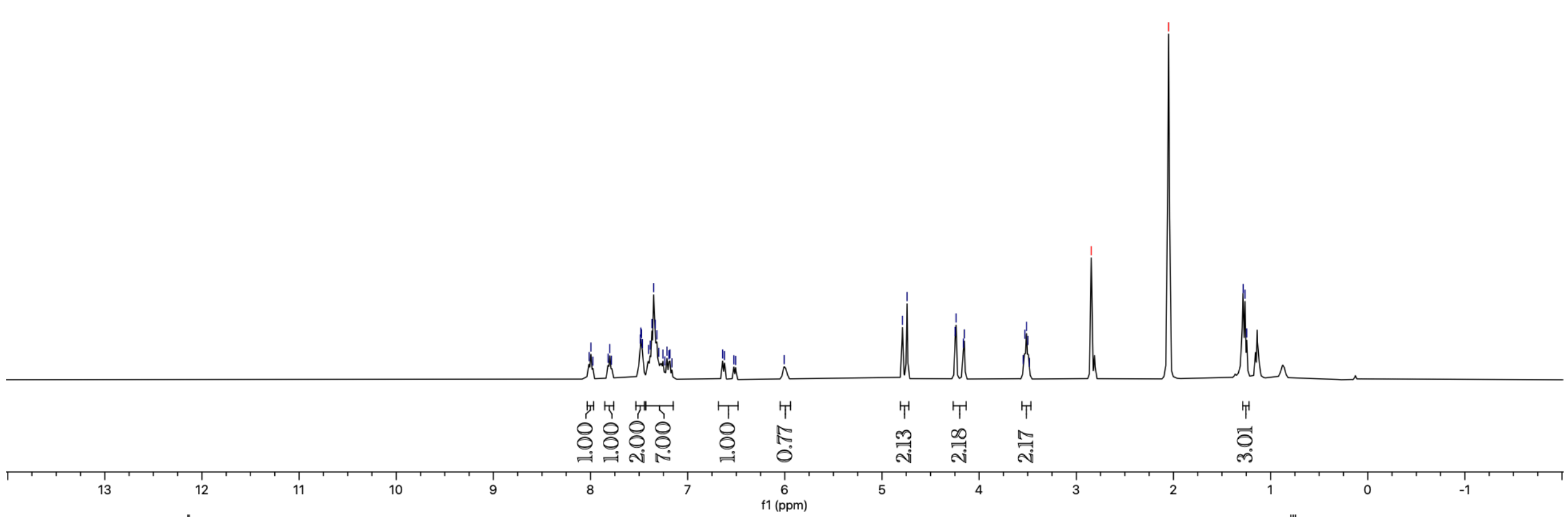
8.01 8.00 7.98 7.82 7.80 7.79 7.49 7.48 7.47 7.47 7.40 7.39 7.37 7.35 7.33 7.32 7.30 7.25 7.23 7.21 7.19 7.18 7.16 6.64 6.62 6.52 6.51 6.01
4.79 4.74 4.25 4.24 4.16 4.15 3.55 3.53 3.51 3.50 3.48
2.85 H2O
2.05 acetone
1.28 1.26 1.25
1.00 1.00 2.00 7.00 1.00 0.77 2.13 2.18 2.17 3.01
13 12 11 10 9 8 7 6 5 4 3 2 1 0 -1
f1 (ppm)

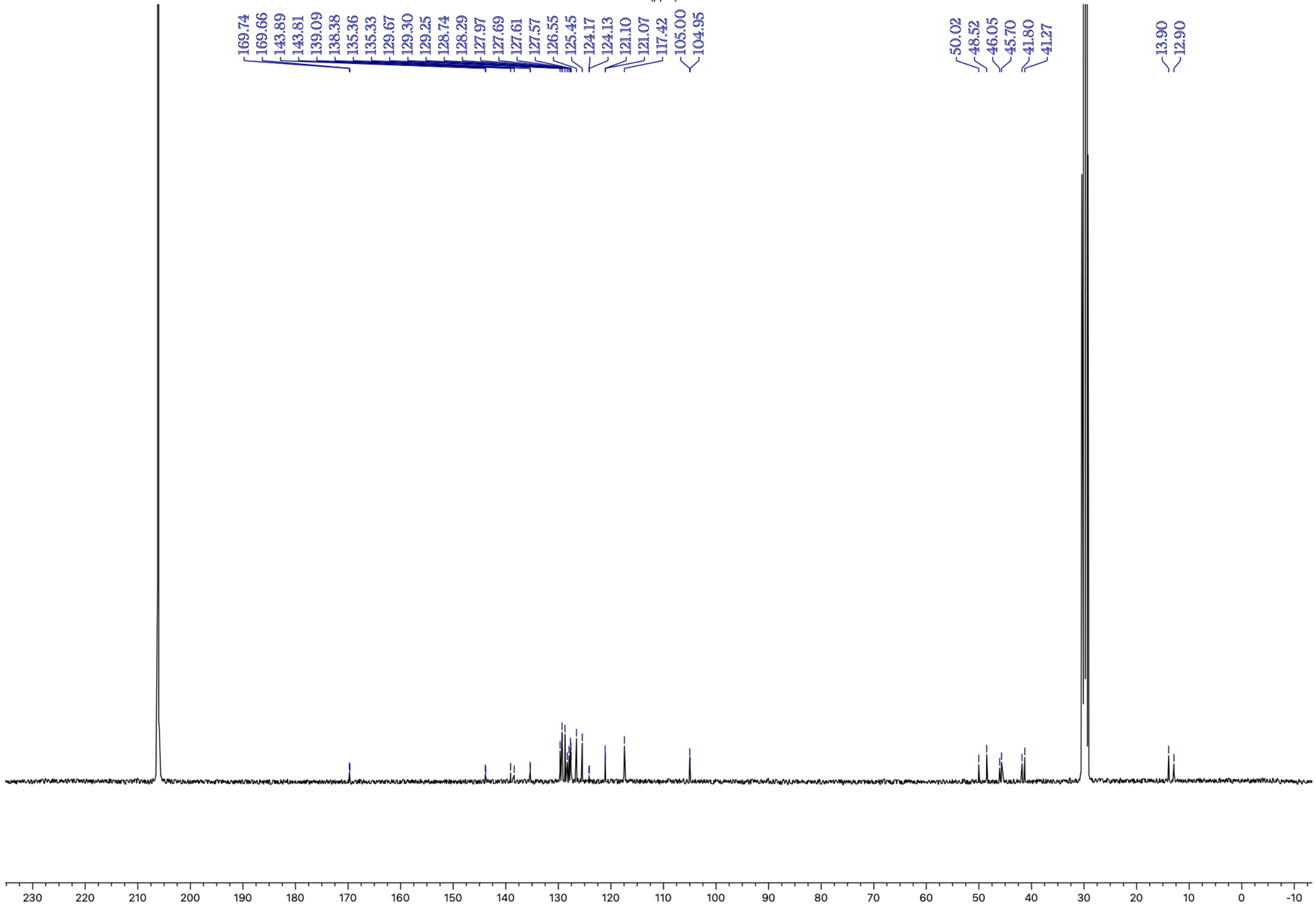
169.74 169.66 143.89 143.81 139.09 138.38 135.36 135.33 129.67 129.30 129.25 128.74 128.29 127.97 127.69 127.61 127.57 126.55 125.45 124.17 124.13 121.10 121.07 117.42 105.00 104.95
50.02 48.52 46.05 45.70 41.80 41.27
13.90 12.90
230 220 210 200 190 180 170 160 150 140 130 120 110 100 90 80 70 60 50 40 30 20 10 0 -10
f1 (ppm)

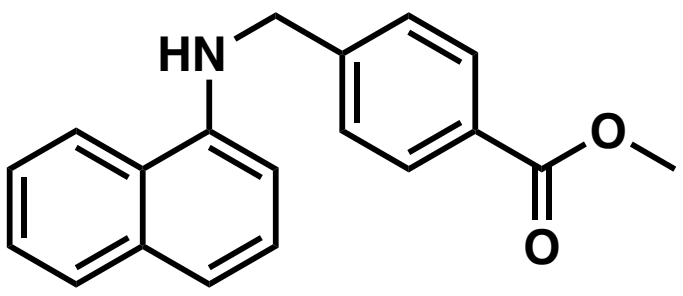
HN
O
O

ALK-08

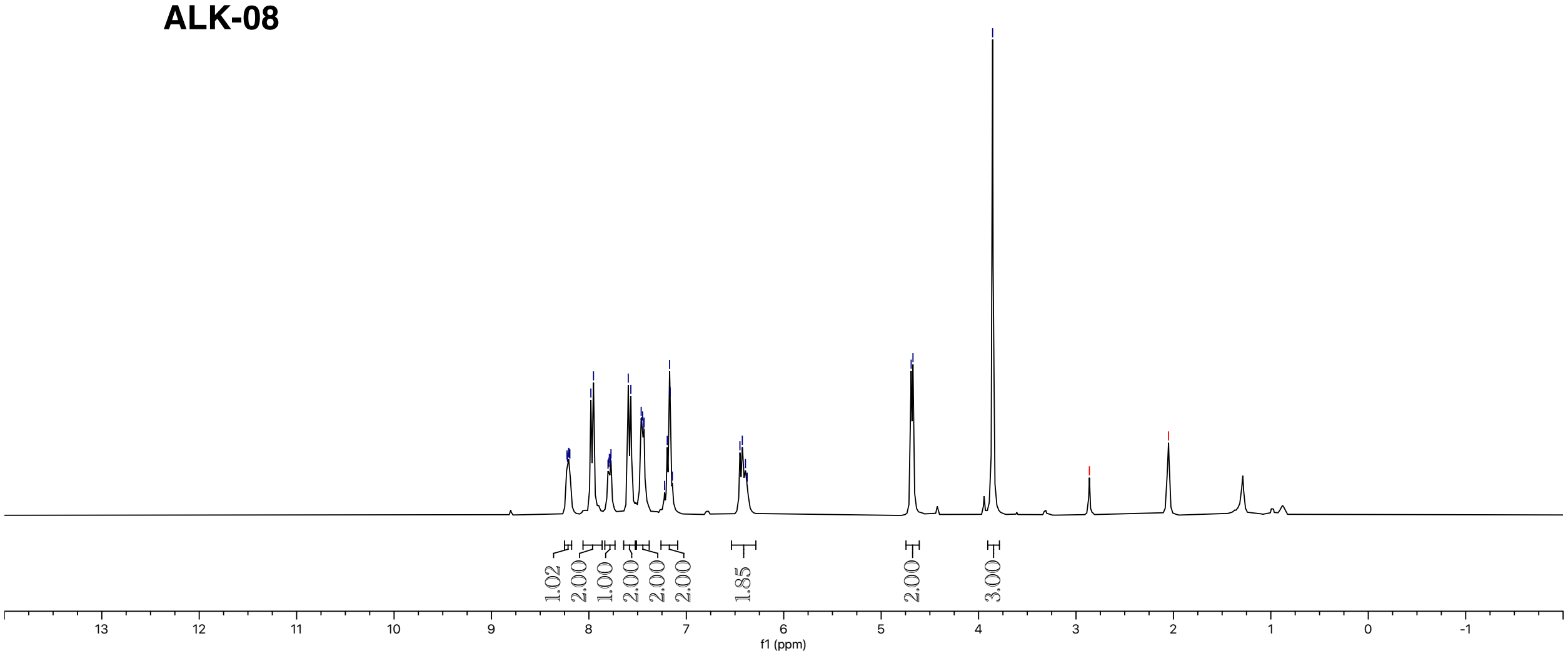
8.22
8.22
8.21
8.20
8.20
7.98
7.95
7.80
7.79
7.79
7.78
7.77
7.60
7.57
7.46
7.46
7.45
7.44
7.43
7.22
7.20
7.17
7.17
7.14
6.45
6.43
6.39
6.38
4.69
4.67
3.86
2.86 H2O
2.05 acetone
1.02
2.00
1.00
2.00
2.00
2.00
1.85
2.00
3.00
f1 (ppm)

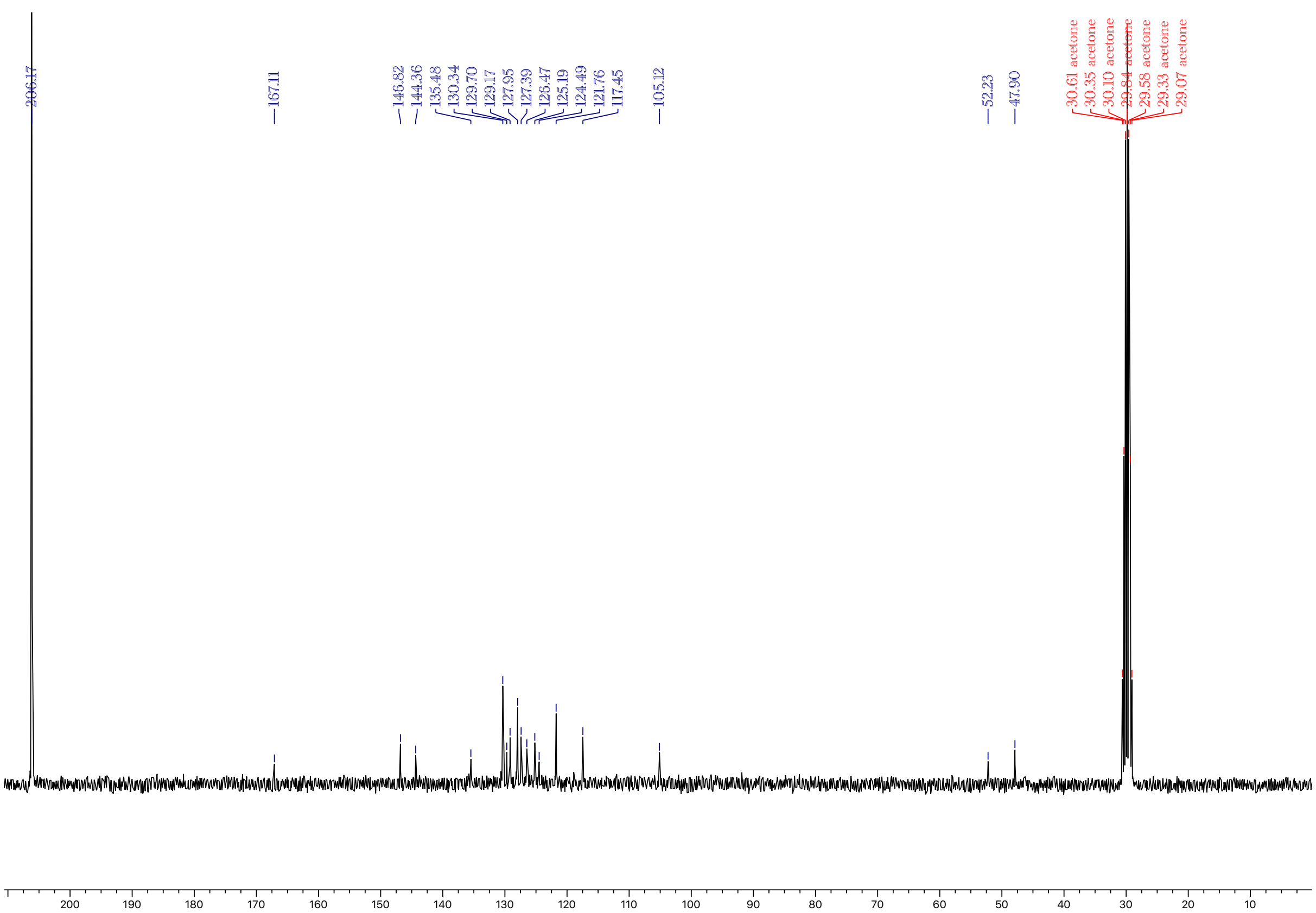
206.17
167.11
146.82
144.36
135.48
130.34
129.70
129.17
127.95
127.39
126.47
125.19
124.49
121.76
117.45
105.12
52.23
47.90
30.61 acetone
30.35 acetone
30.10 acetone
29.84 acetone
29.58 acetone
29.33 acetone
29.07 acetone
f1 (ppm)

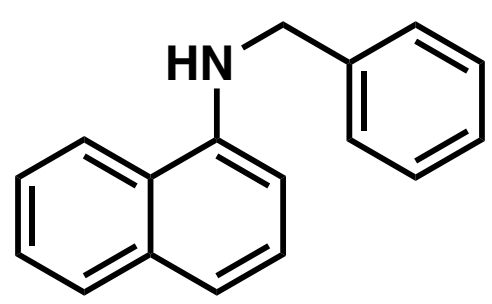

ALK-09

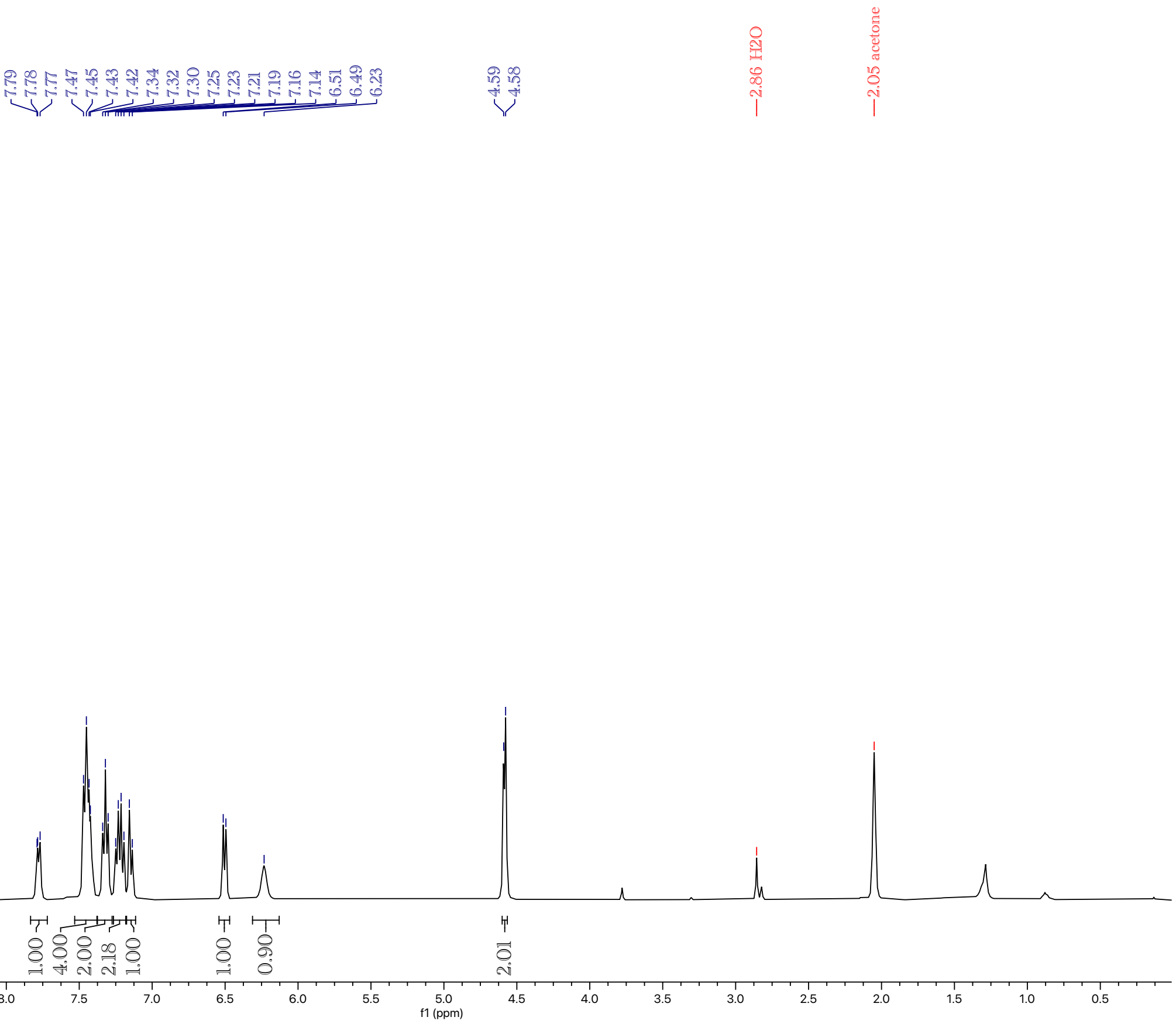

8.21
8.19
7.79
7.78
7.77
7.47
7.45
7.43
7.42
7.34
7.32
7.30
7.25
7.23
7.21
7.19
7.16
7.14
6.51
6.49
6.23
4.59
4.58
2.86 H2O
2.05 acetone
1.00
1.00
4.00
2.00
2.18
1.00
1.00
0.90
2.01
f1 (ppm)

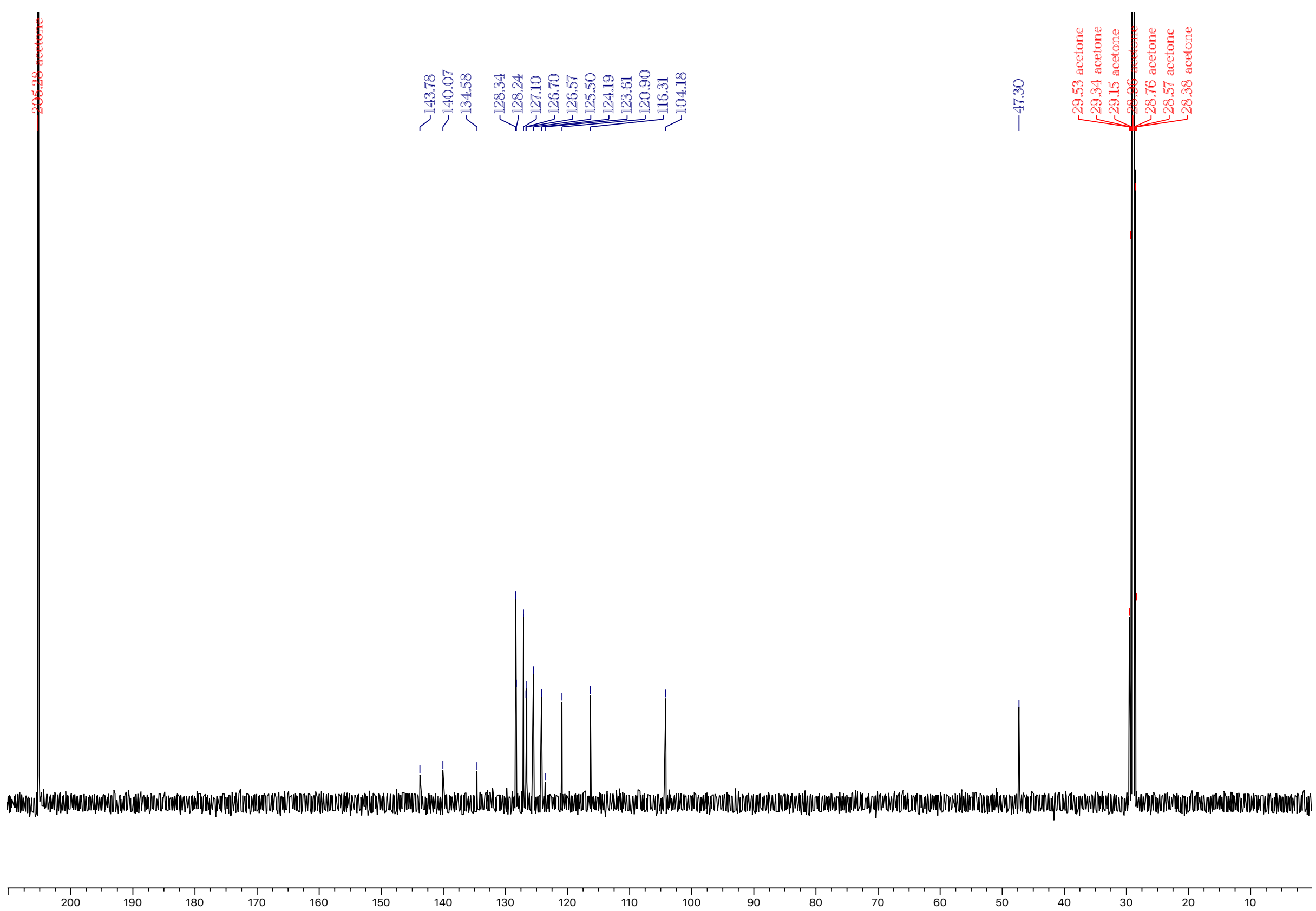

143.78
140.07
134.58
128.34
128.24
127.10
126.70
126.57
125.50
124.19
123.61
120.90
116.31
104.18
47.30
29.53 acetone
29.34 acetone
29.15 acetone
28.76 acetone
28.57 acetone
28.38 acetone
f1 (ppm)

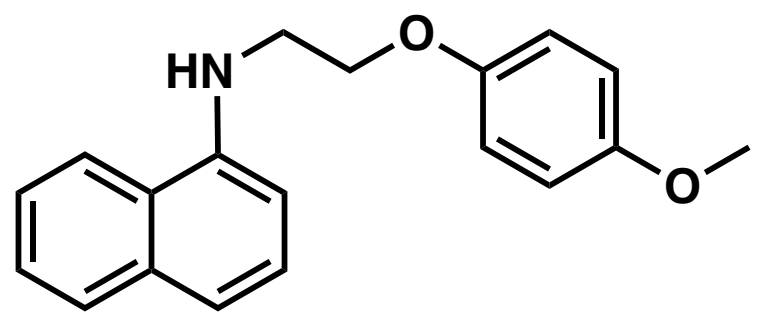
HN
O
O

ALK-10

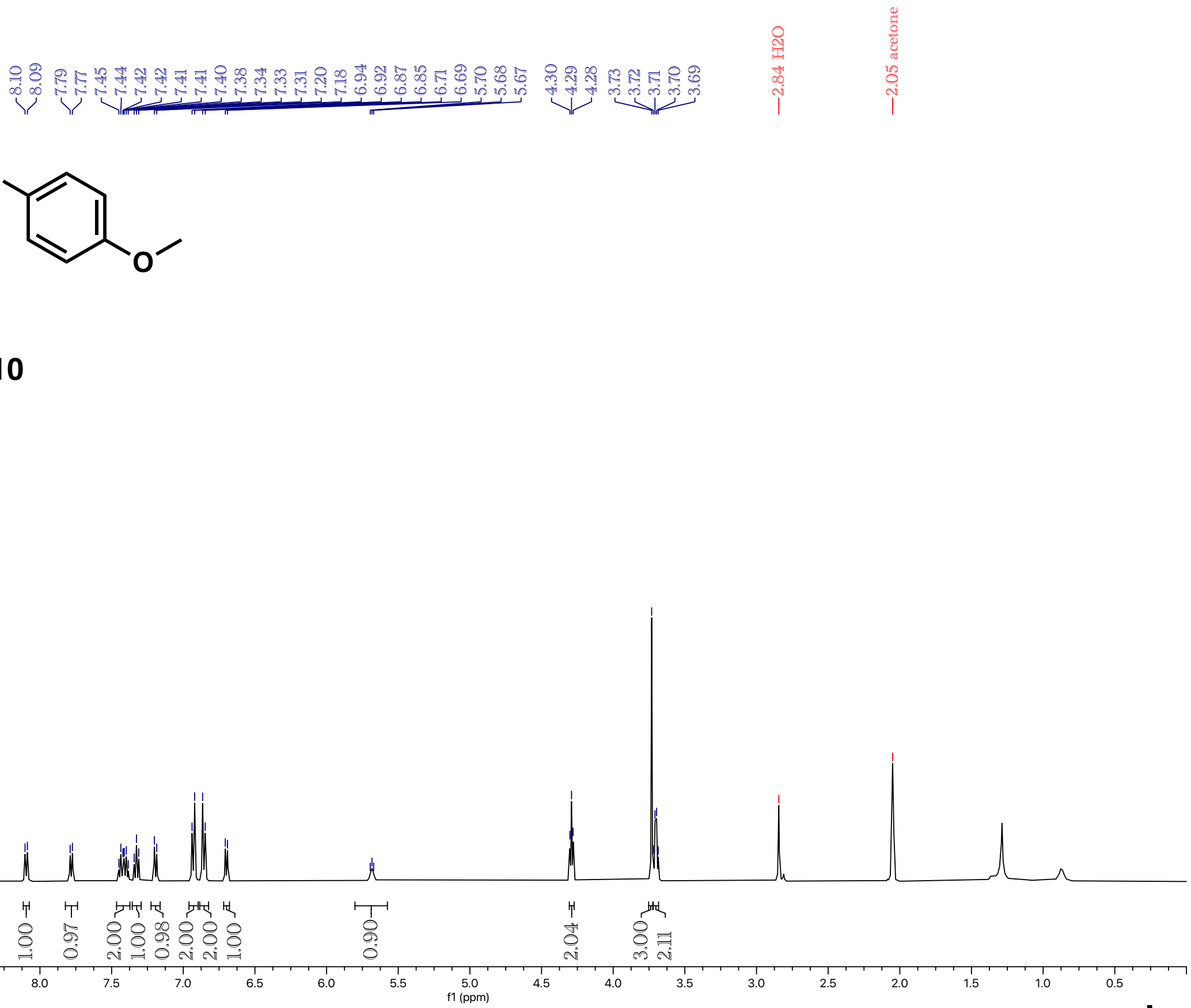
8.10
8.09
7.79
7.77
7.45
7.44
7.42
7.42
7.41
7.41
7.40
7.38
7.34
7.33
7.31
7.20
7.18
6.94
6.92
6.87
6.85
6.71
6.69
5.70
5.68
5.67
4.30
4.29
4.28
3.73
3.72
3.71
3.70
3.69
2.84 H2O
2.05 acetone
1.00
0.97
2.00
1.00
0.98
2.00
2.00
1.00
0.90
2.04
3.00
2.11
f1 (ppm)

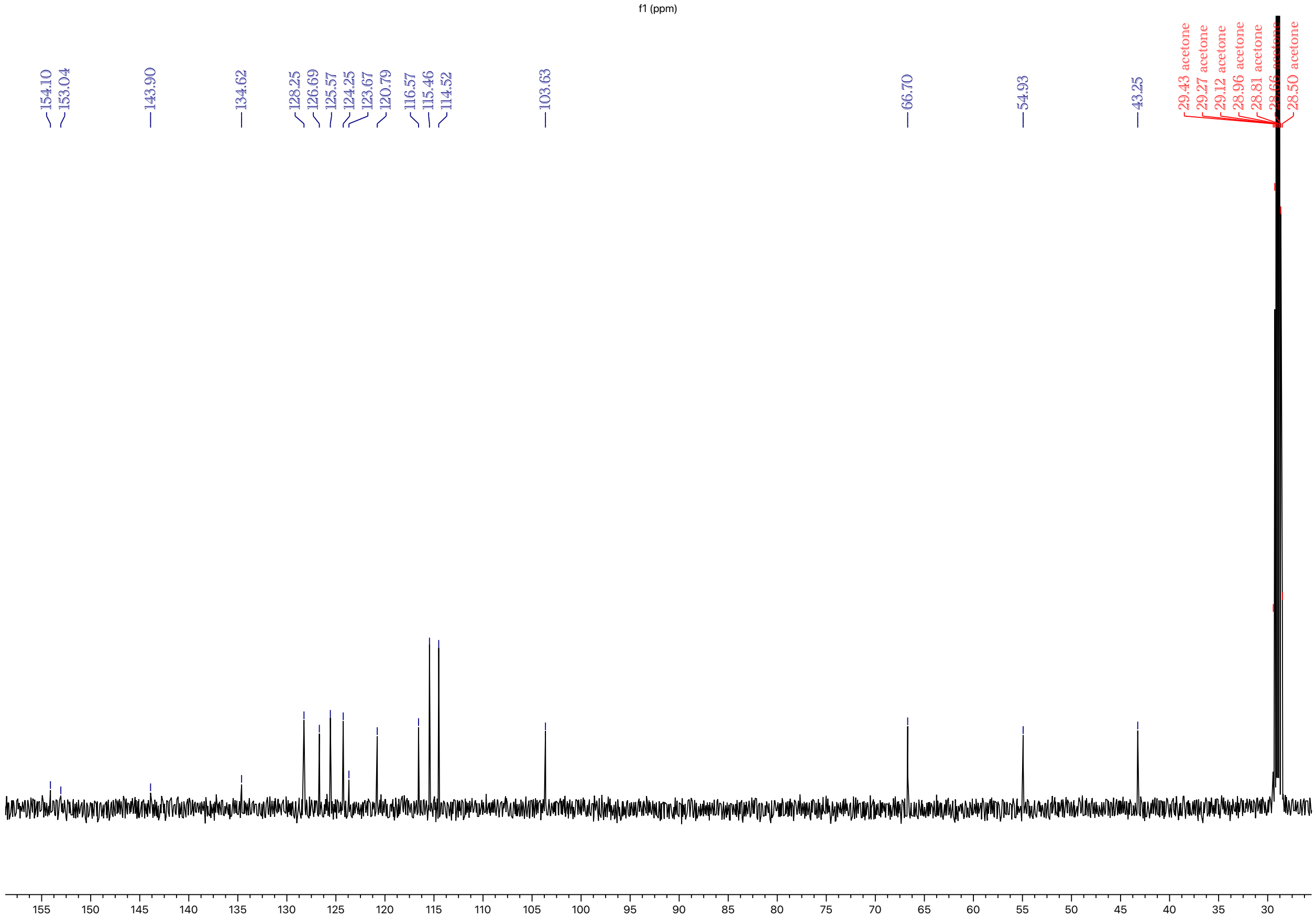
154.10
153.04
143.90
134.62
128.25
126.69
125.57
124.25
123.67
120.79
116.57
115.46
114.52
103.63
66.70
54.93
43.25
29.43 acetone
29.27 acetone
29.12 acetone
28.96 acetone
28.81 acetone
28.50 acetone
f1 (ppm)

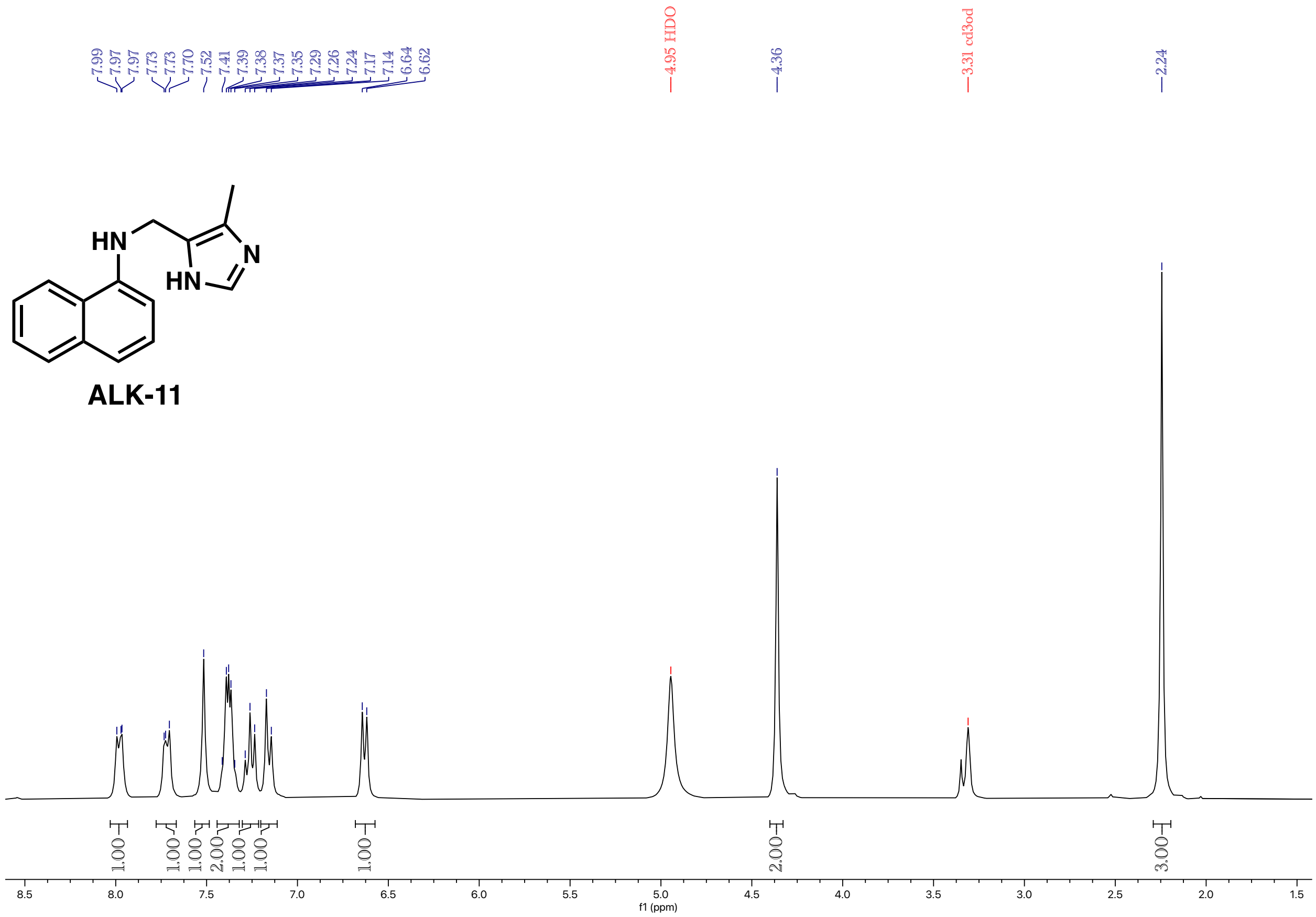

ALK-11

7.99
7.97
7.97
7.73
7.73
7.70
7.52
7.41
7.39
7.38
7.37
7.35
7.29
7.26
7.24
7.17
7.14
6.64
6.62
4.95 HDO
4.36
3.31 cd3od
2.24
1.00
1.00
1.00
2.00
1.00
1.00
1.00
2.00
3.00
f1 (ppm)

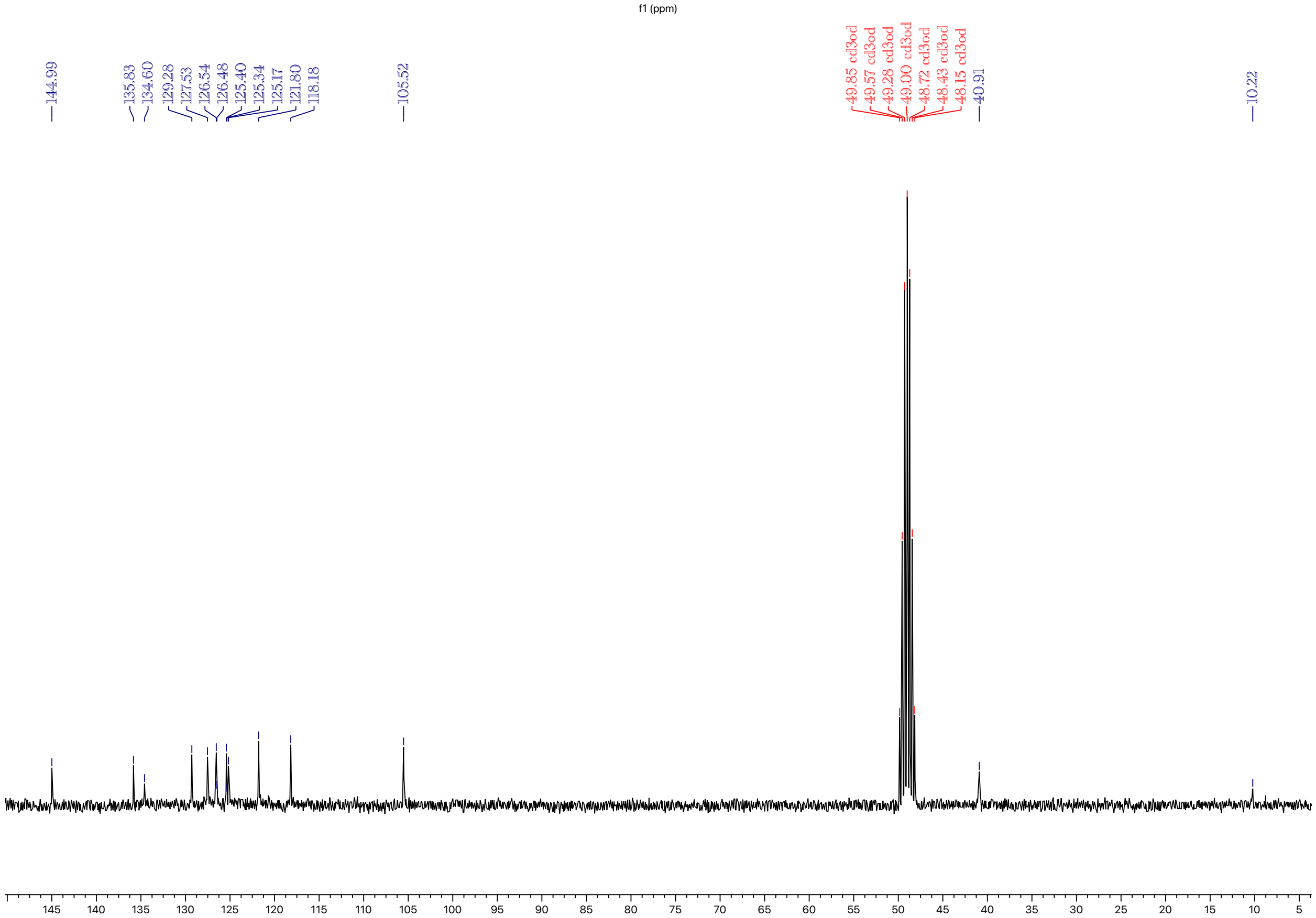

144.99
135.83
134.60
129.28
127.53
126.54
126.48
125.40
125.34
125.17
121.80
118.18
105.52
49.85 cd3od
49.57 cd3od
49.28 cd3od
49.00 cd3od
48.72 cd3od
48.43 cd3od
48.15 cd3od
40.91
10.22
f1 (ppm)

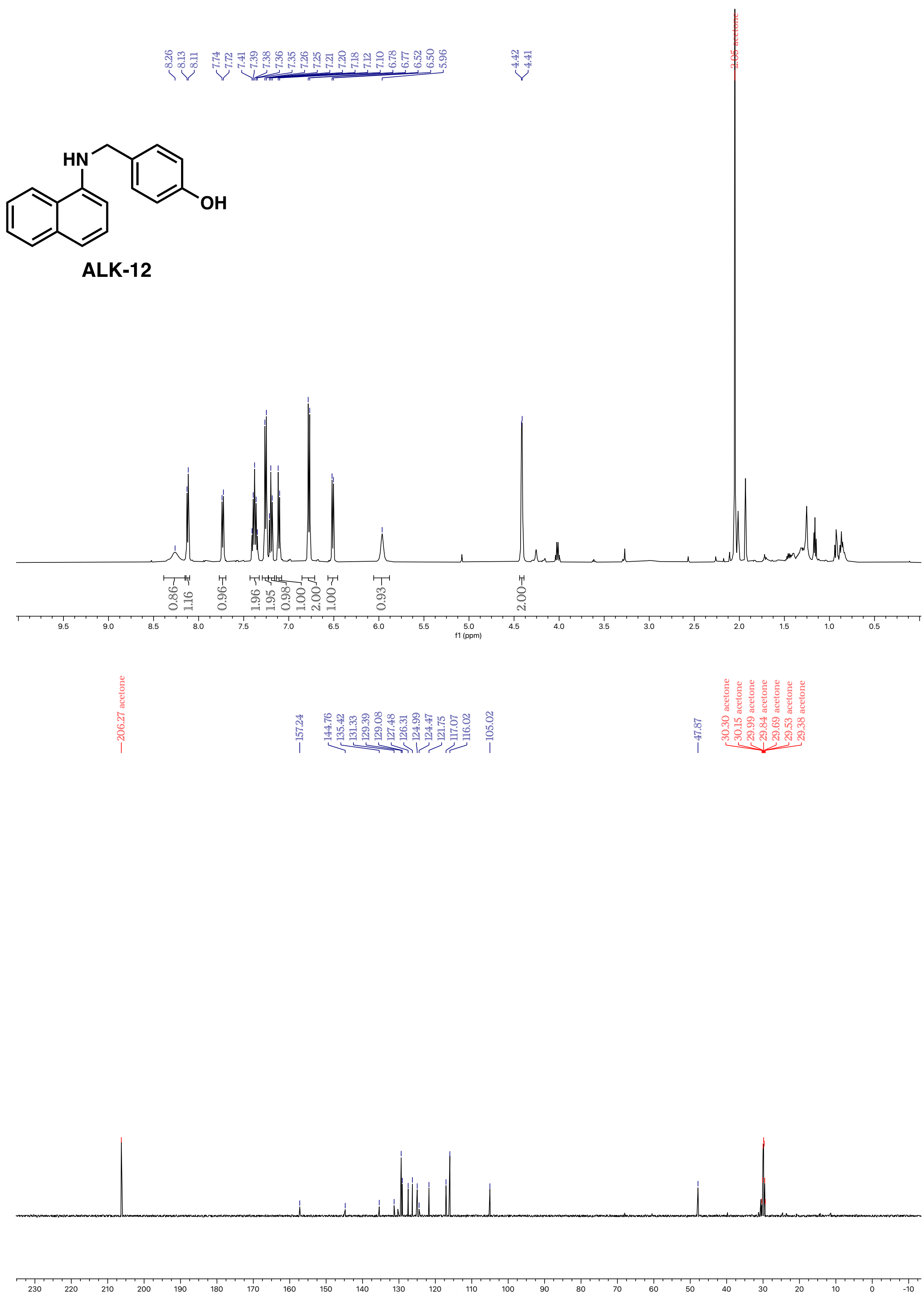
HN
OH
ALK-12
8.26
8.13
8.11
7.74
7.72
7.41
7.39
7.38
7.36
7.35
7.26
7.25
7.21
7.20
7.18
7.12
7.10
6.78
6.77
6.52
6.50
5.96
4.42
4.41
2.05 acetone
0.86
1.16
0.96
1.96
1.95
0.98
1.00
2.00
1.00
0.93
2.00
f1 (ppm)
206.27 acetone
157.24
144.76
135.42
131.33
129.39
129.08
127.48
126.31
124.99
124.47
121.75
117.07
116.02
105.02
47.87
30.30 acetone
30.15 acetone
29.99 acetone
29.84 acetone
29.69 acetone
29.53 acetone
29.38 acetone
f1 (ppm)

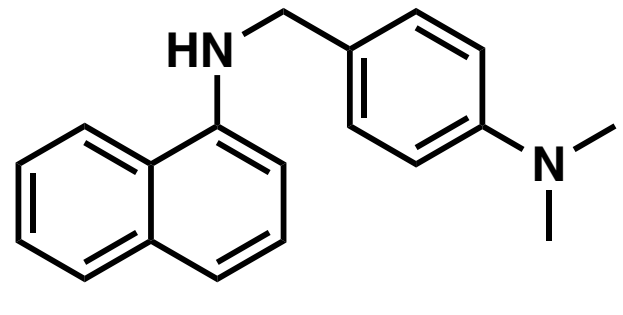

ALK-13

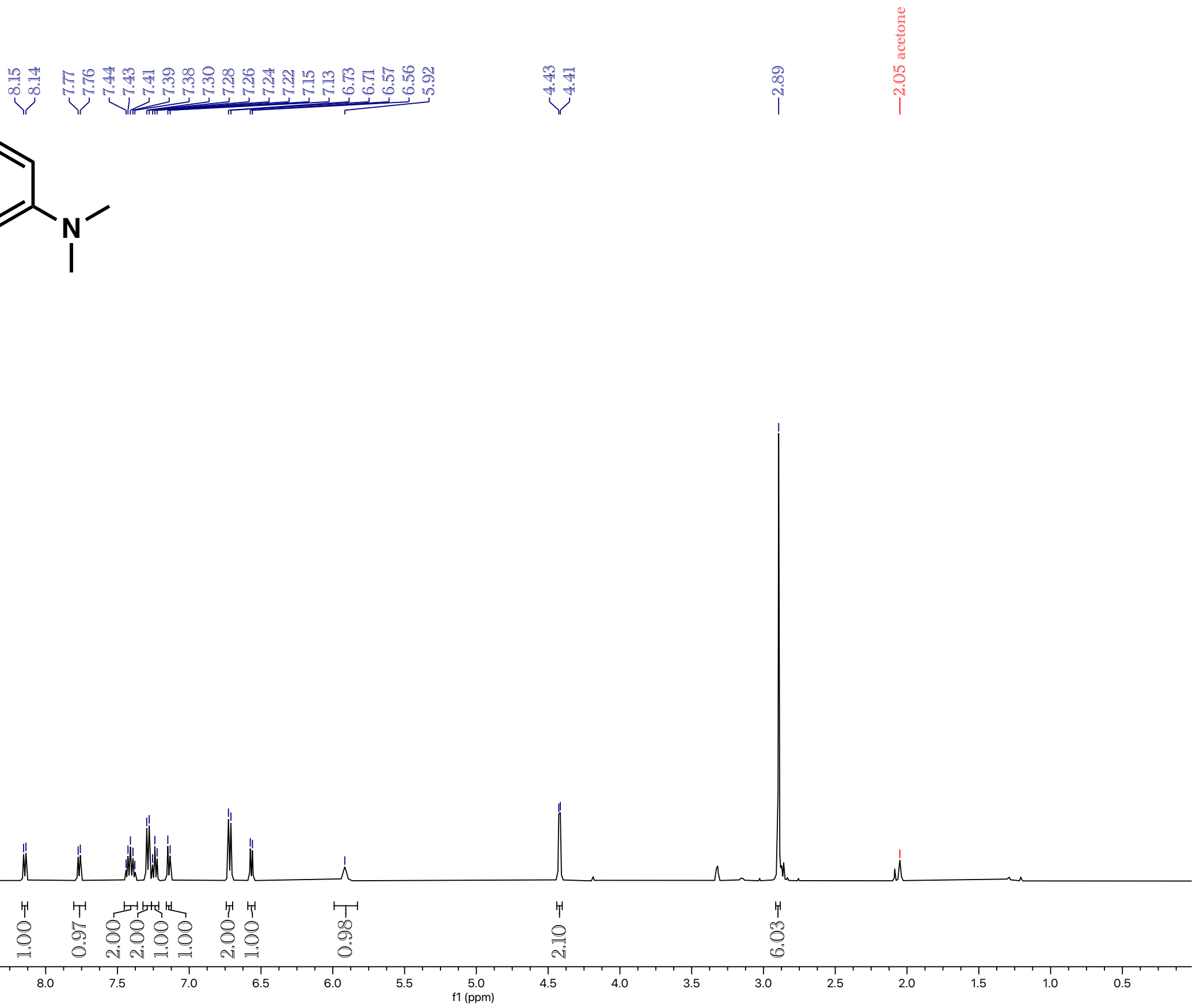
8.15
8.14
7.77
7.76
7.44
7.43
7.41
7.39
7.38
7.30
7.28
7.26
7.24
7.22
7.15
7.13
6.73
6.71
6.57
6.56
5.92
4.43
4.41
2.89
2.05 acetone
1.00
0.97
2.00
2.00
1.00
1.00
2.00
1.00
0.98
2.10
6.03
f1 (ppm)

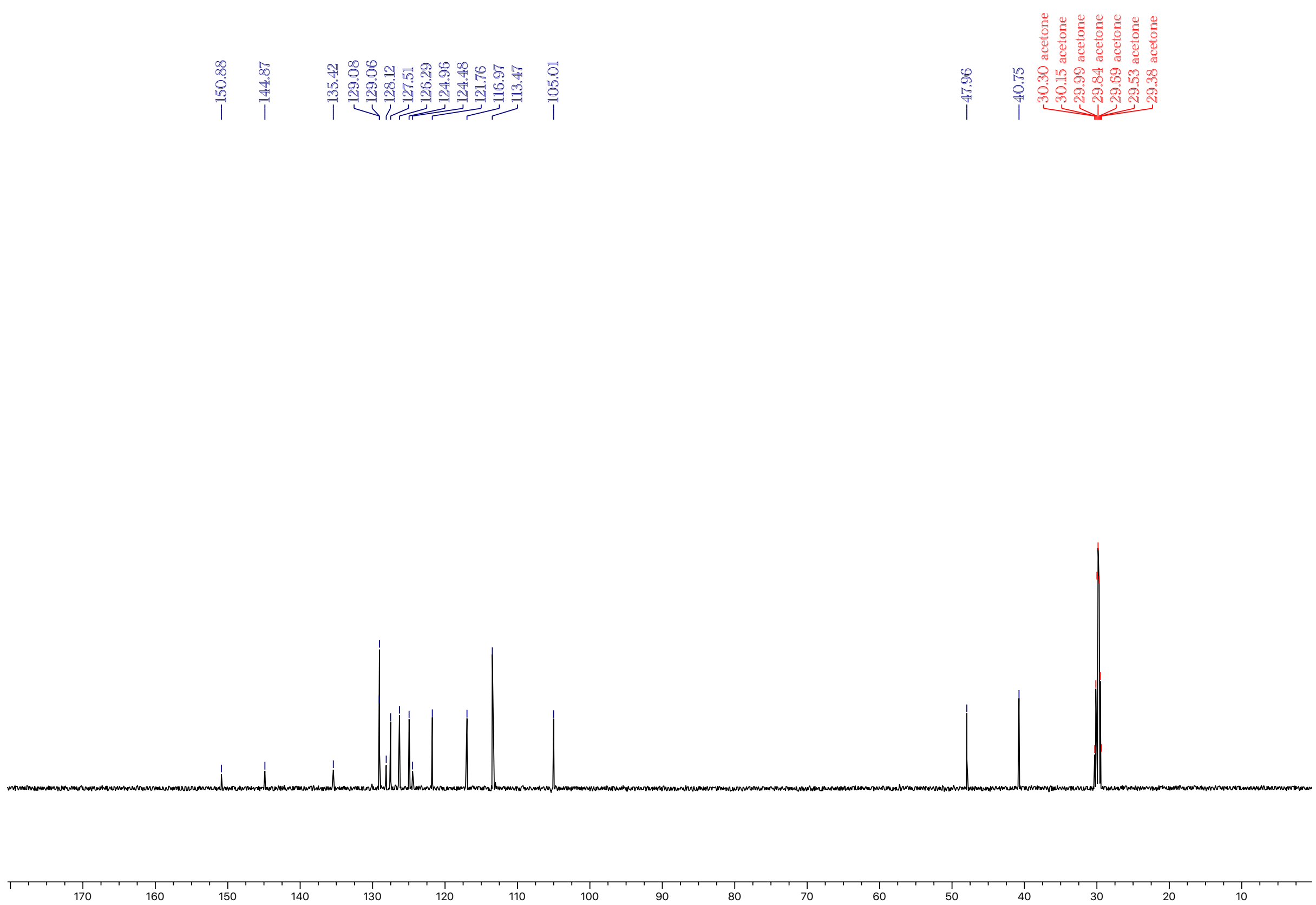
150.88
144.87
135.42
129.08
129.06
128.12
127.51
126.29
124.96
124.48
121.76
116.97
113.47
105.01
47.96
40.75
30.30 acetone
30.15 acetone
29.99 acetone
29.84 acetone
29.69 acetone
29.53 acetone
29.38 acetone
f1 (ppm)

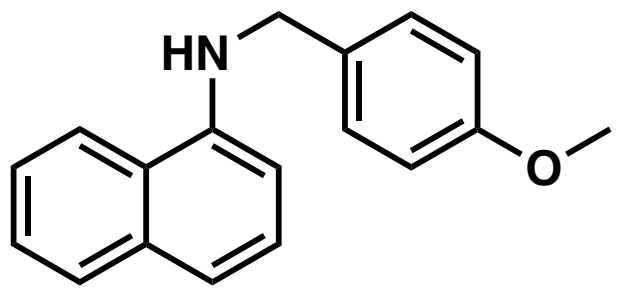
HN
O

ALK-14

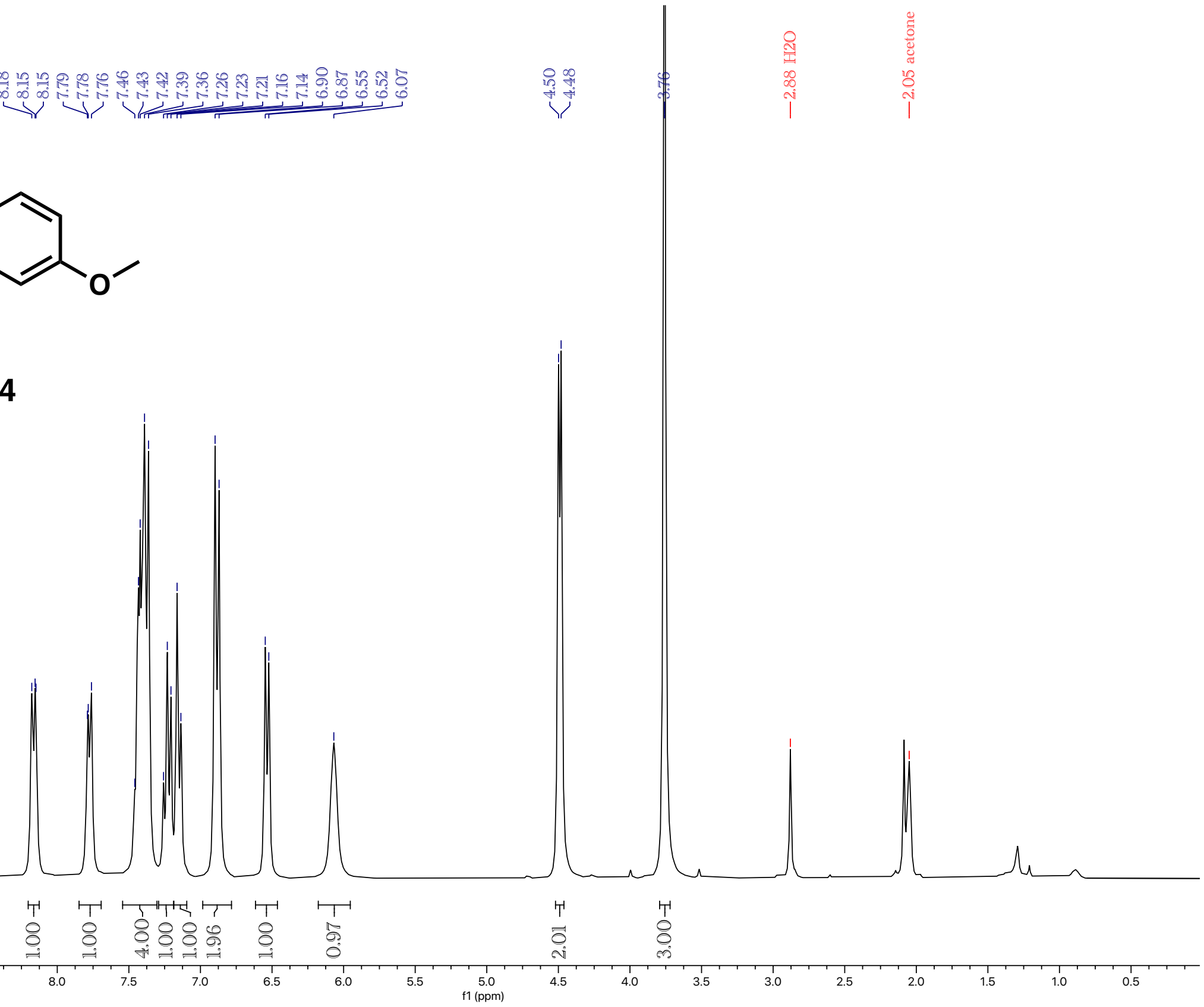
8.18
8.15
8.15
7.79
7.78
7.76
7.46
7.43
7.42
7.39
7.36
7.26
7.23
7.21
7.16
7.14
6.90
6.87
6.55
6.52
6.07
4.50
4.48
3.76
2.88 H2O
2.05 acetone
1.00
1.00
4.00
1.00
1.00
1.96
1.00
0.97
2.01
3.00
9.5
9.0
8.5
8.0
7.5
7.0
6.5
6.0
5.5
5.0
4.5
4.0
3.5
3.0
2.5
2.0
1.5
1.0
0.5
f1 (ppm)

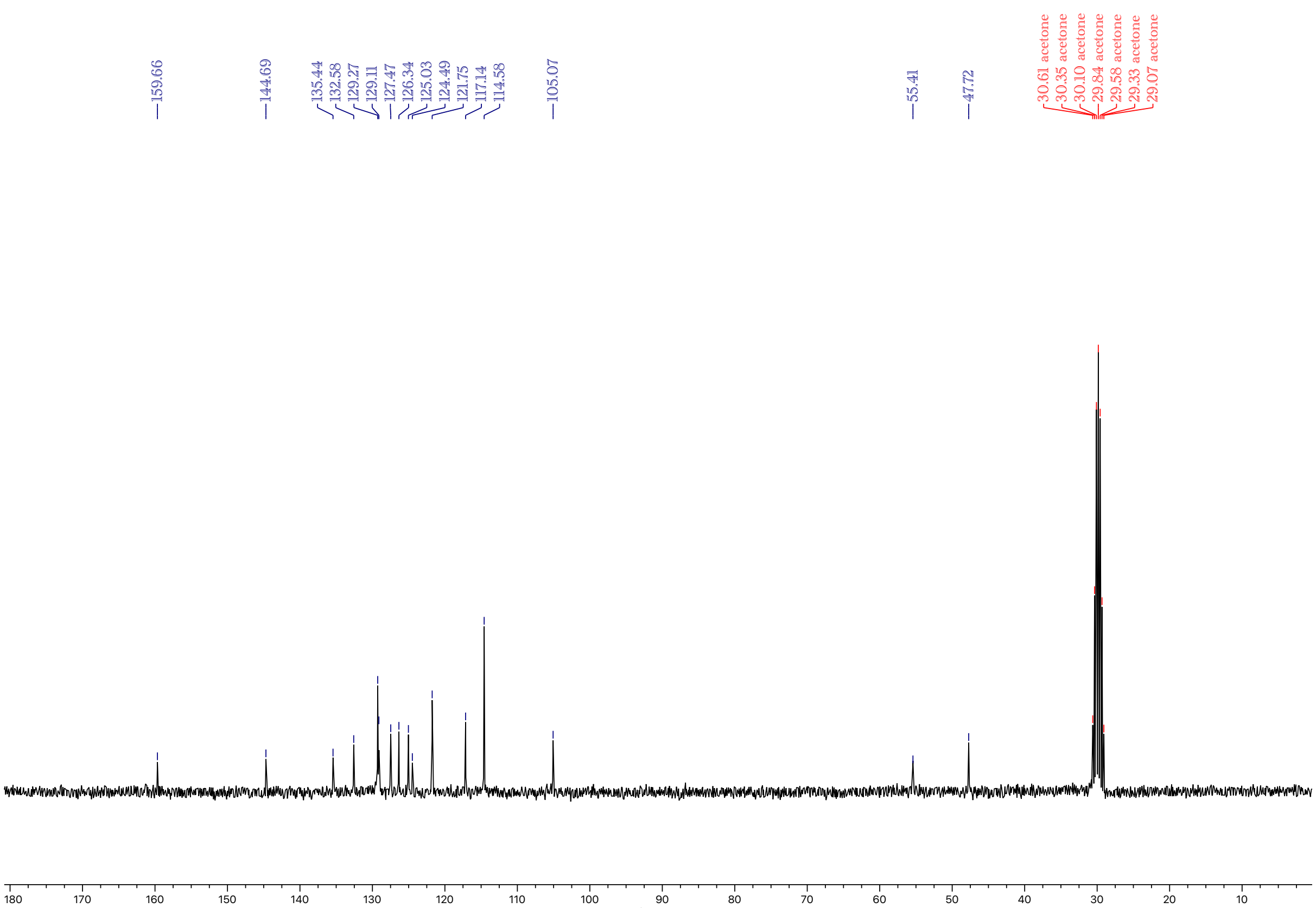
159.66
144.69
135.44
132.58
129.27
129.11
127.47
126.34
125.03
124.49
121.75
117.14
114.58
105.07
55.41
47.72
30.61 acetone
30.35 acetone
30.10 acetone
29.84 acetone
29.58 acetone
29.33 acetone
29.07 acetone
180
170
160
150
140
130
120
110
100
90
80
70
60
50
40
30
20
10
f1 (ppm)

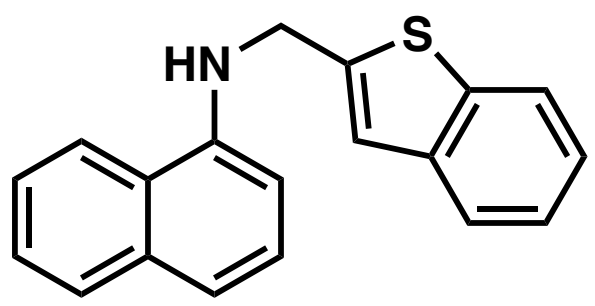

ALK-15

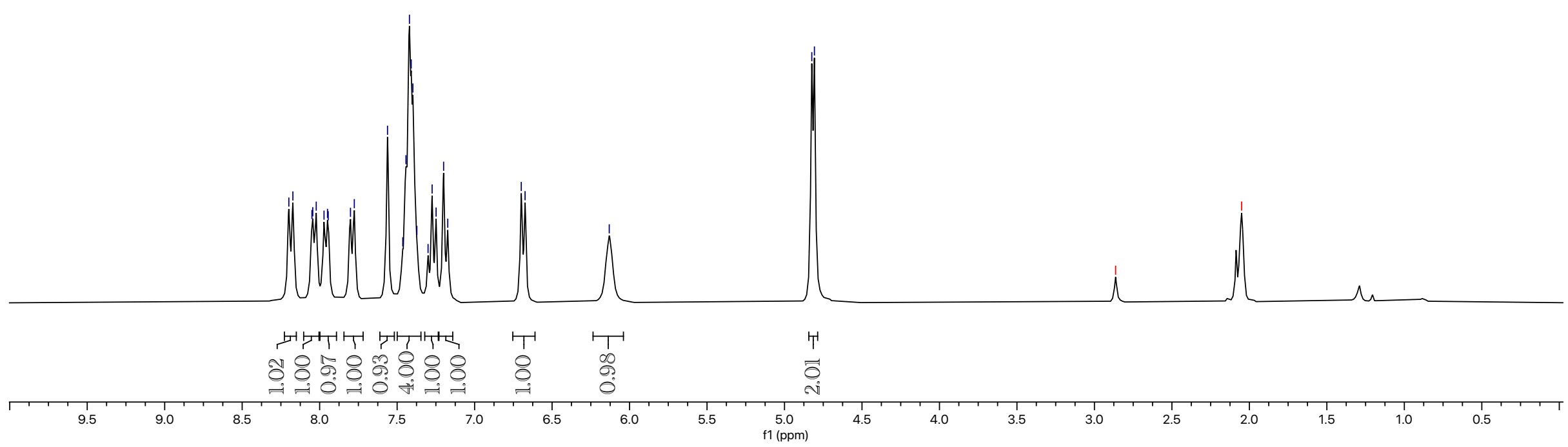

8.20
8.17
8.05
8.04
8.02
7.97
7.95
7.94
7.80
7.78
7.56
7.46
7.44
7.42
7.41
7.40
7.37
7.30
7.27
7.25
7.20
7.17
6.70
6.67
6.13
4.82
4.81
2.86 H2O
2.05 acetone
1.02
1.00
0.97
1.00
0.93
4.00
1.00
1.00
1.00
0.98
2.01
f1 (ppm)

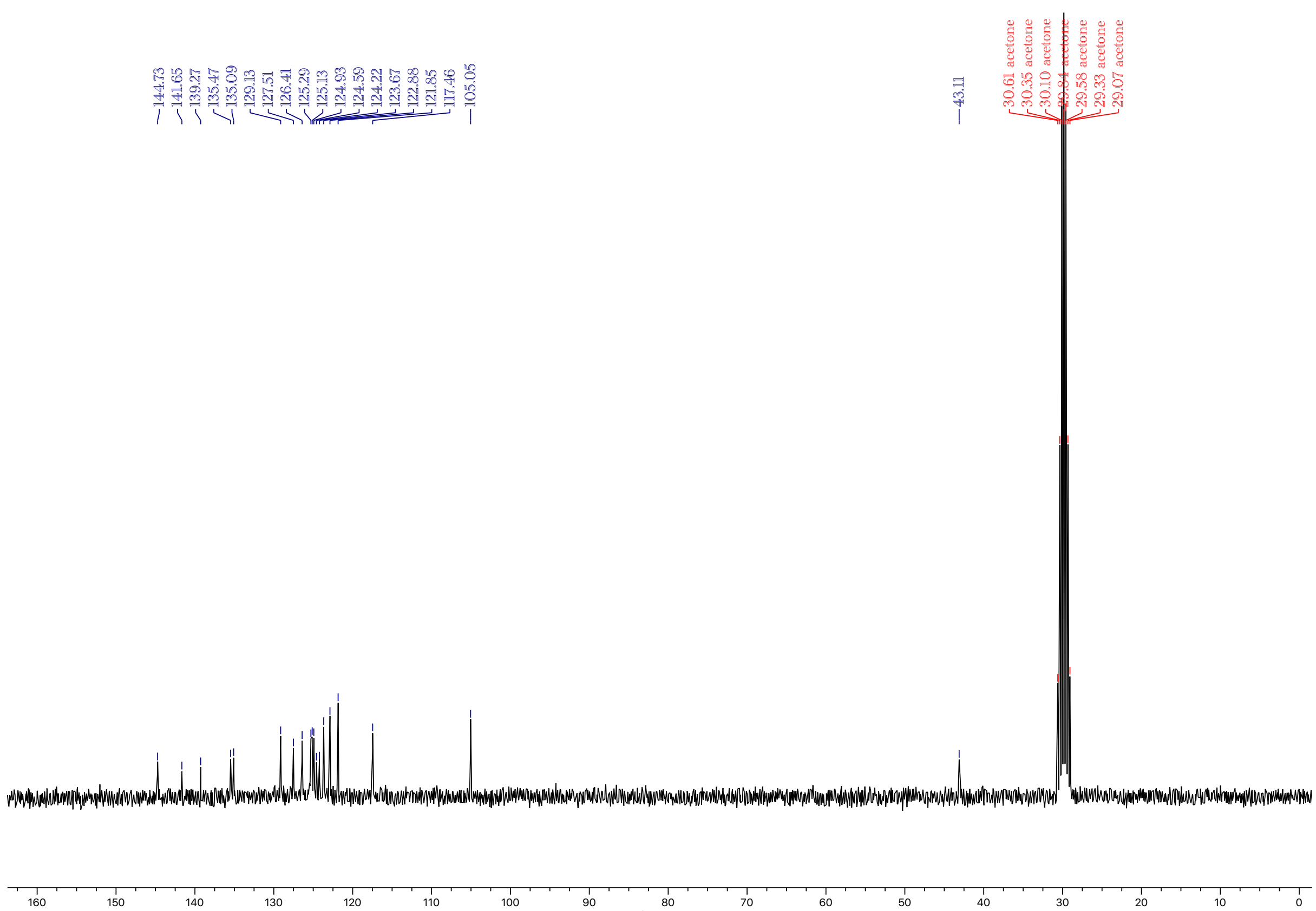

144.73
141.65
139.27
135.47
135.09
129.13
127.51
126.41
125.29
125.13
124.93
124.59
124.22
123.67
122.88
121.85
117.46
105.05
43.11
30.61 acetone
30.35 acetone
30.10 acetone
29.84 acetone
29.58 acetone
29.33 acetone
29.07 acetone
f1 (ppm)

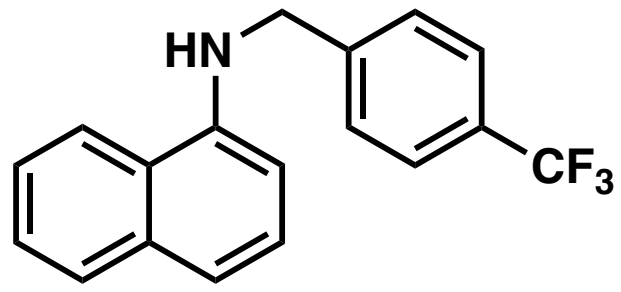
HN
CF_3

ALK-16

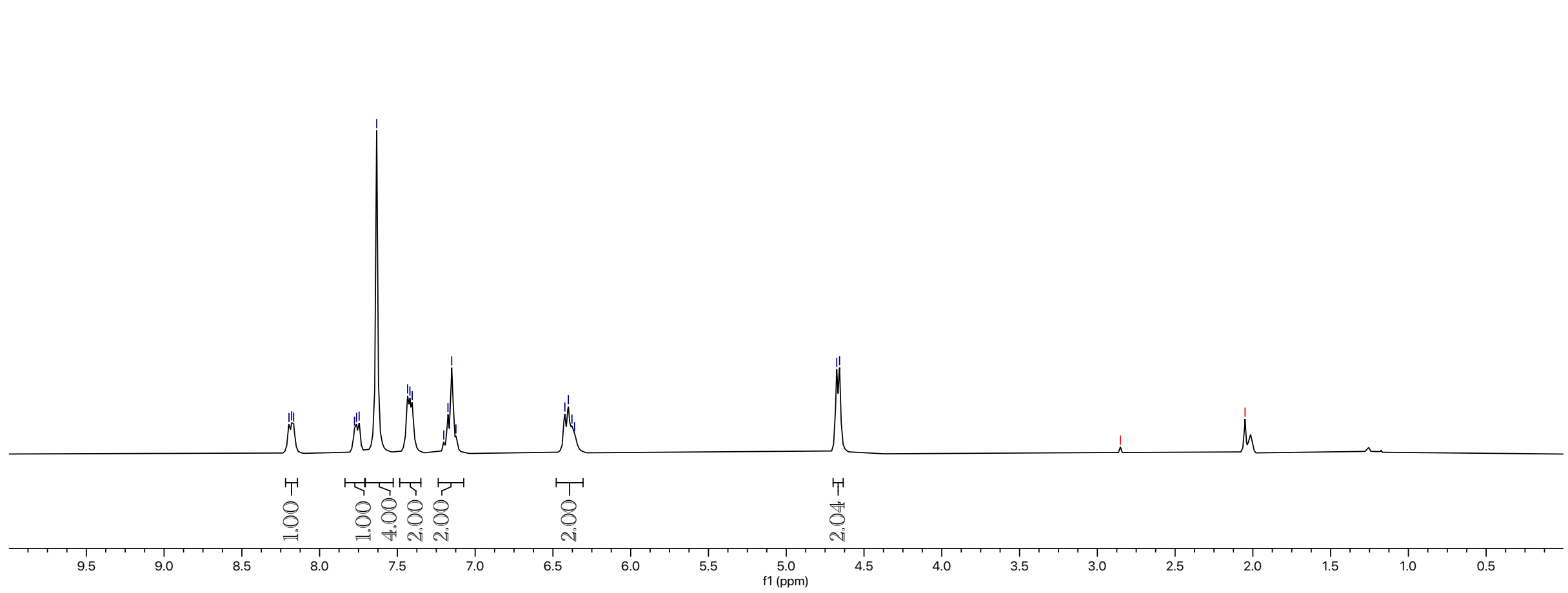
8.20
8.18
8.17
7.78
7.76
7.75
7.63
7.43
7.42
7.40
7.20
7.17
7.15
7.12
6.42
6.40
6.38
6.36
4.68
4.66
2.85 H2O
2.05 acetone
1.00
1.00
4.00
2.00
2.00
2.00
2.04
9.5
9.0
8.5
8.0
7.5
7.0
6.5
6.0
5.5
5.0
4.5
4.0
3.5
3.0
2.5
2.0
1.5
1.0
0.5
f1 (ppm)

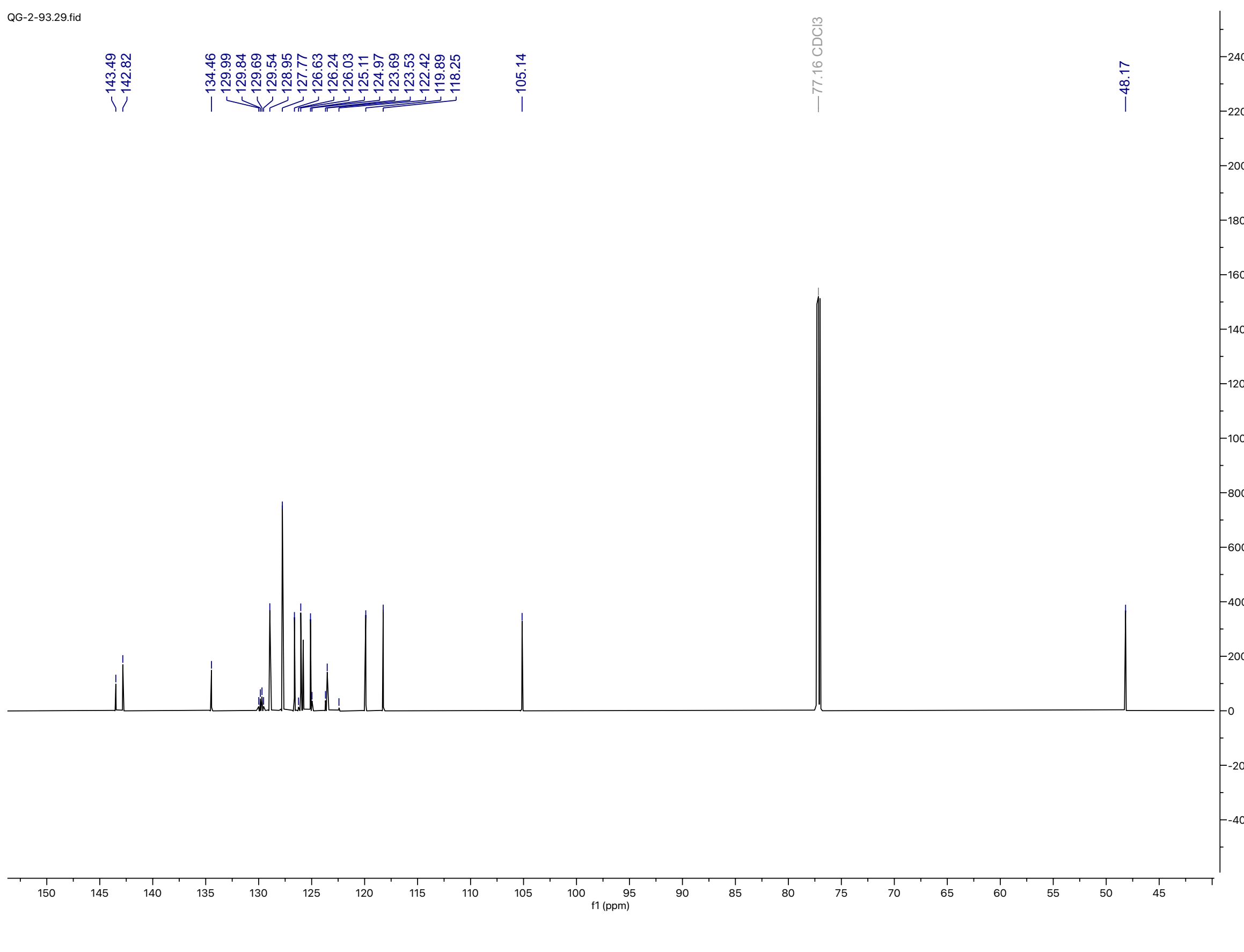
QG-2-93.29.fid
143.49
142.82
134.46
129.99
129.84
129.69
129.54
128.95
127.77
126.63
126.24
126.03
125.11
124.97
123.69
123.53
122.42
119.89
118.25
105.14
77.16 CDCl3
48.17
240000
220000
200000
180000
160000
140000
120000
100000
80000
60000
40000
20000
0
-20000
-40000
150
145
140
135
130
125
120
115
110
105
100
95
90
85
80
75
70
65
60
55
50
45
f1 (ppm)

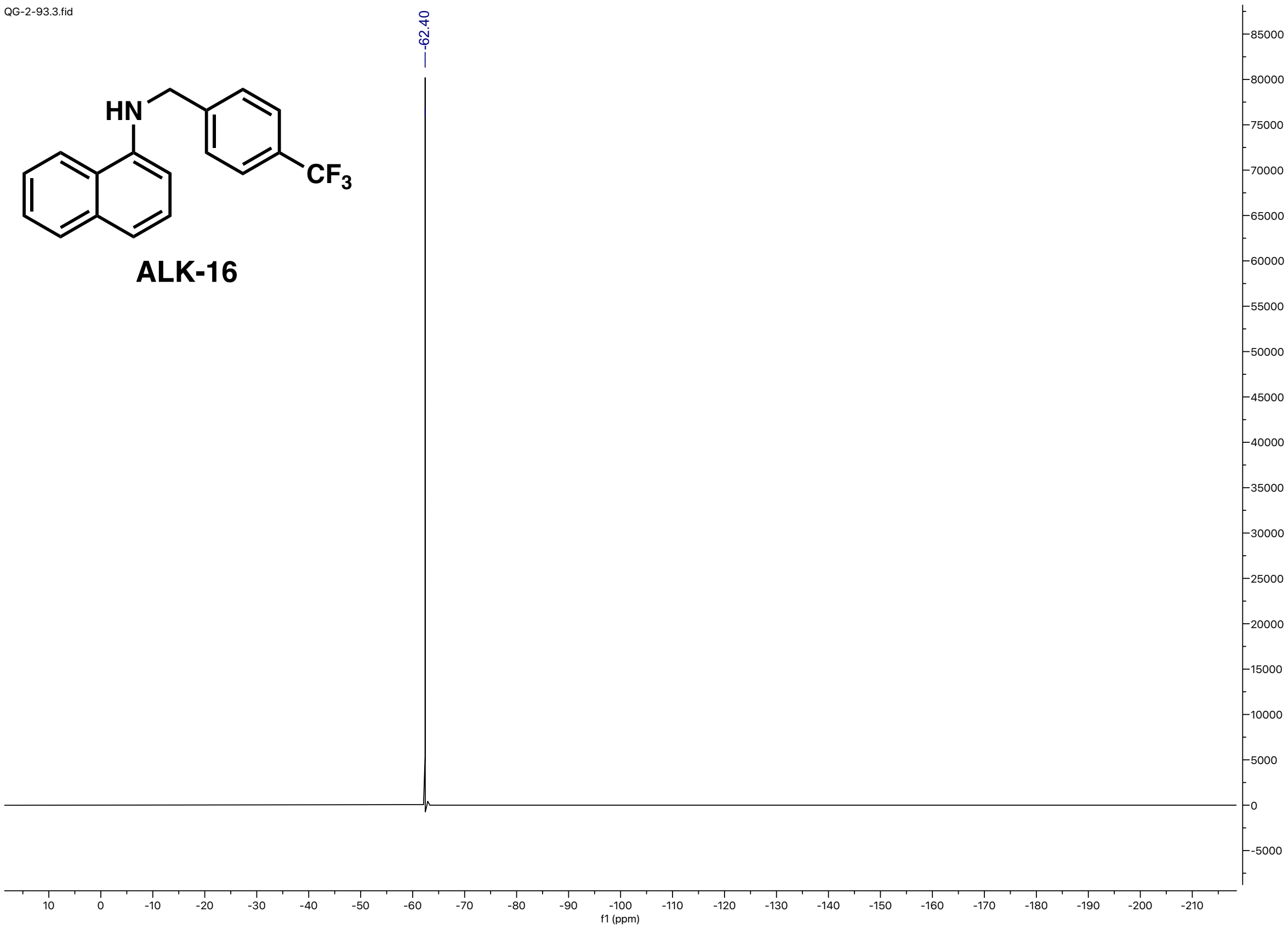
QG-2-93.3.fid
-62.40
HN
CF3
ALK-16
f1 (ppm)

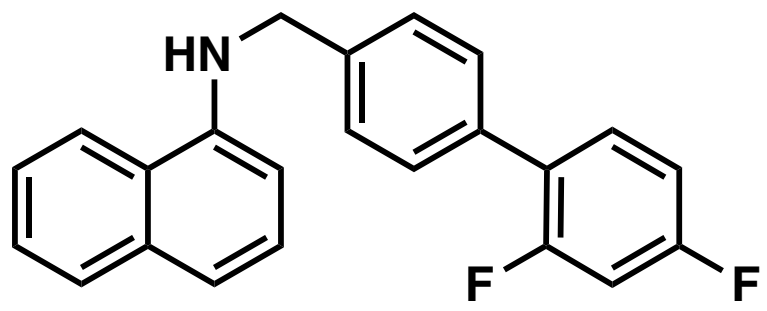

ALK-17

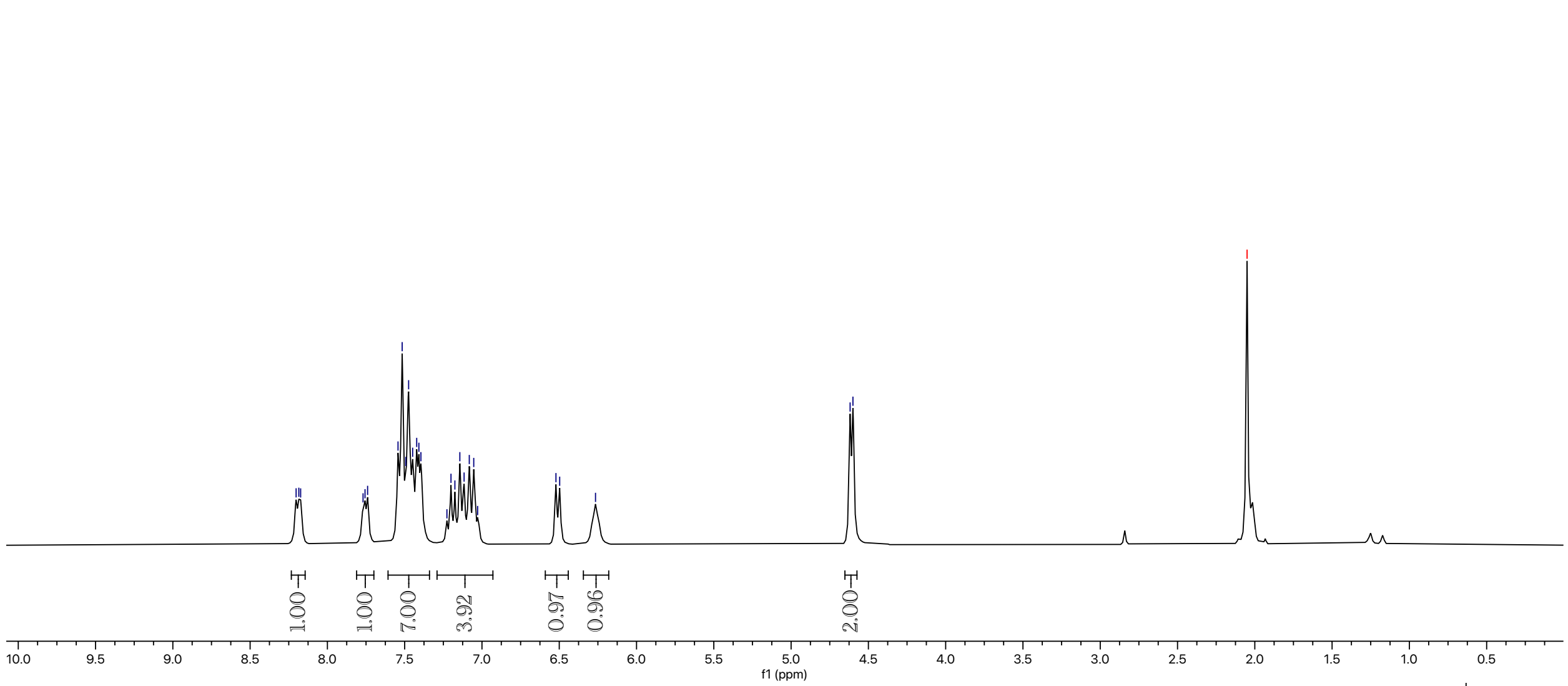

ALK-17.6.fid

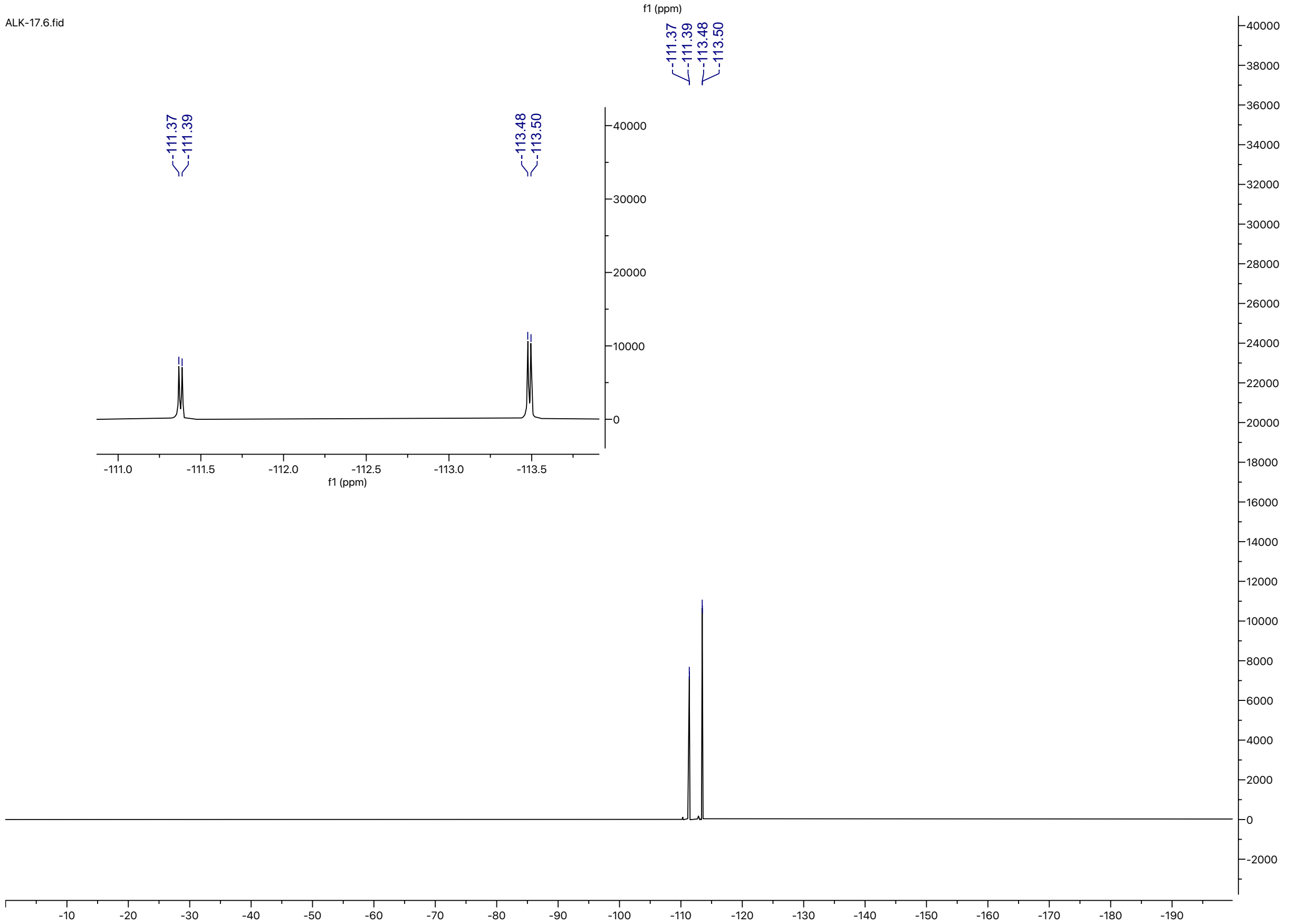

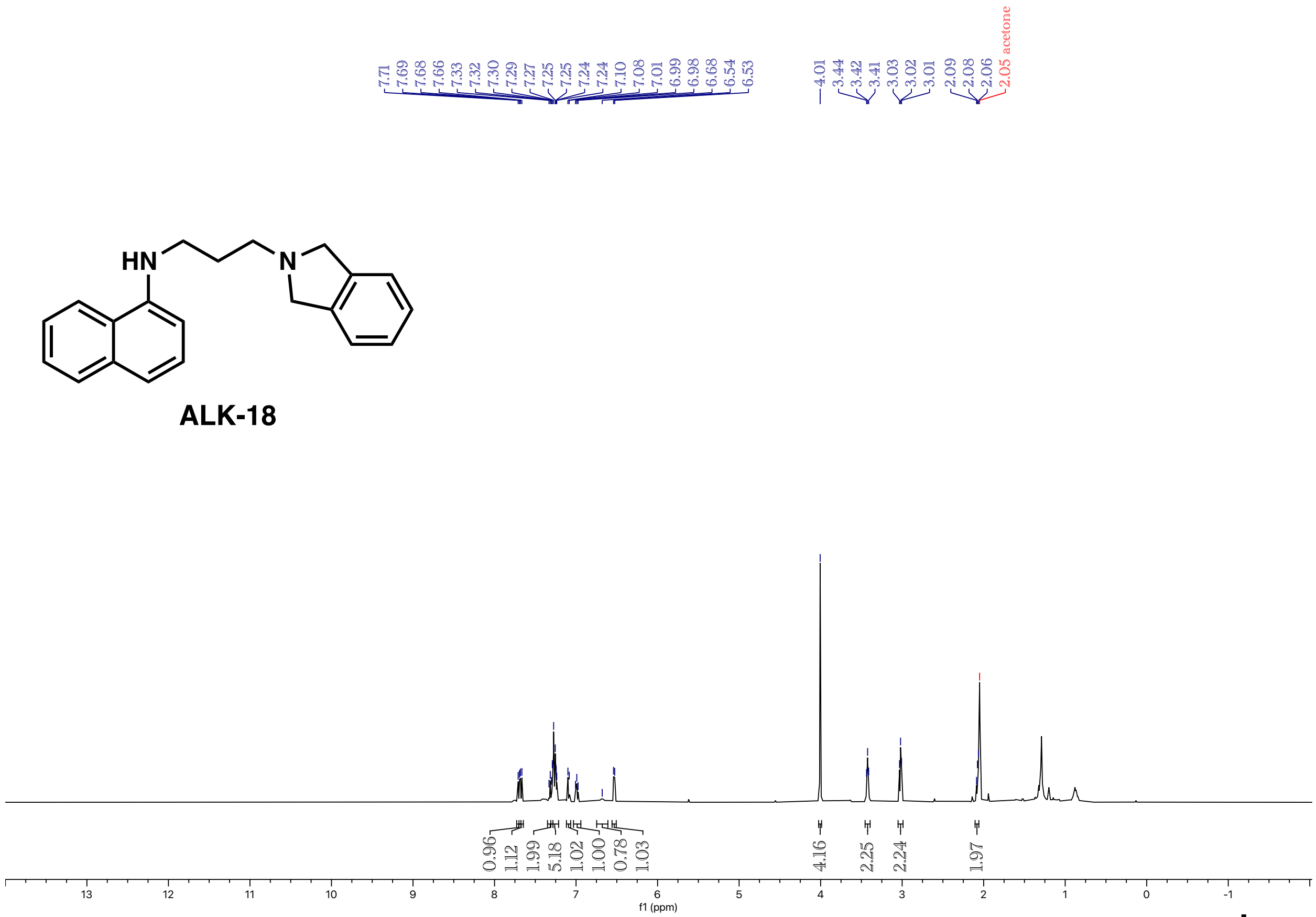

ALK-18

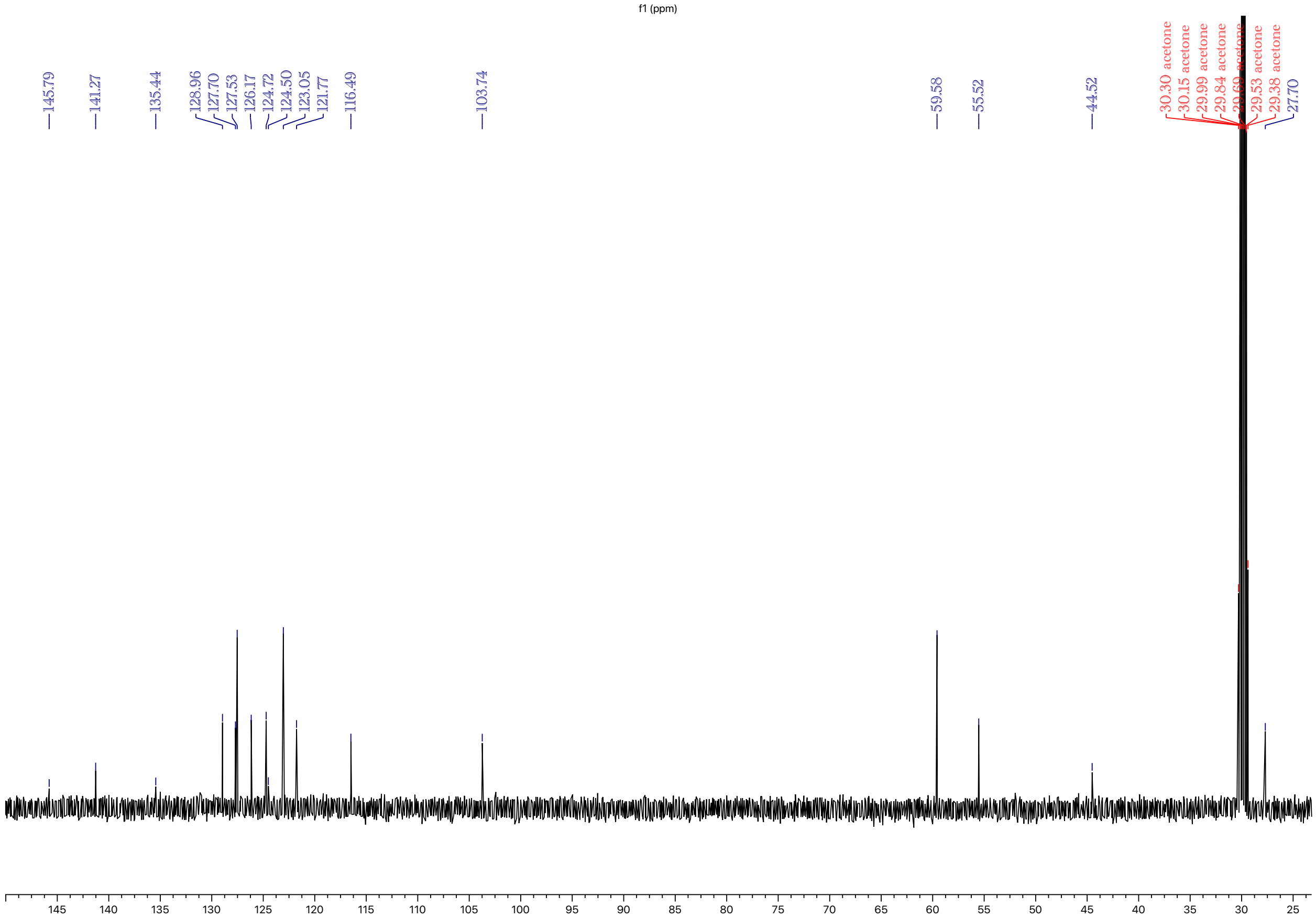

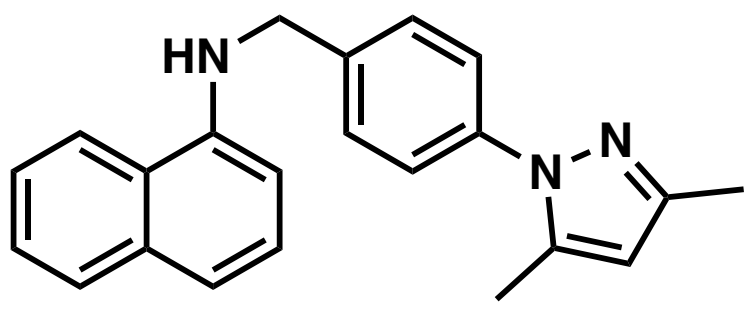

ALK-19

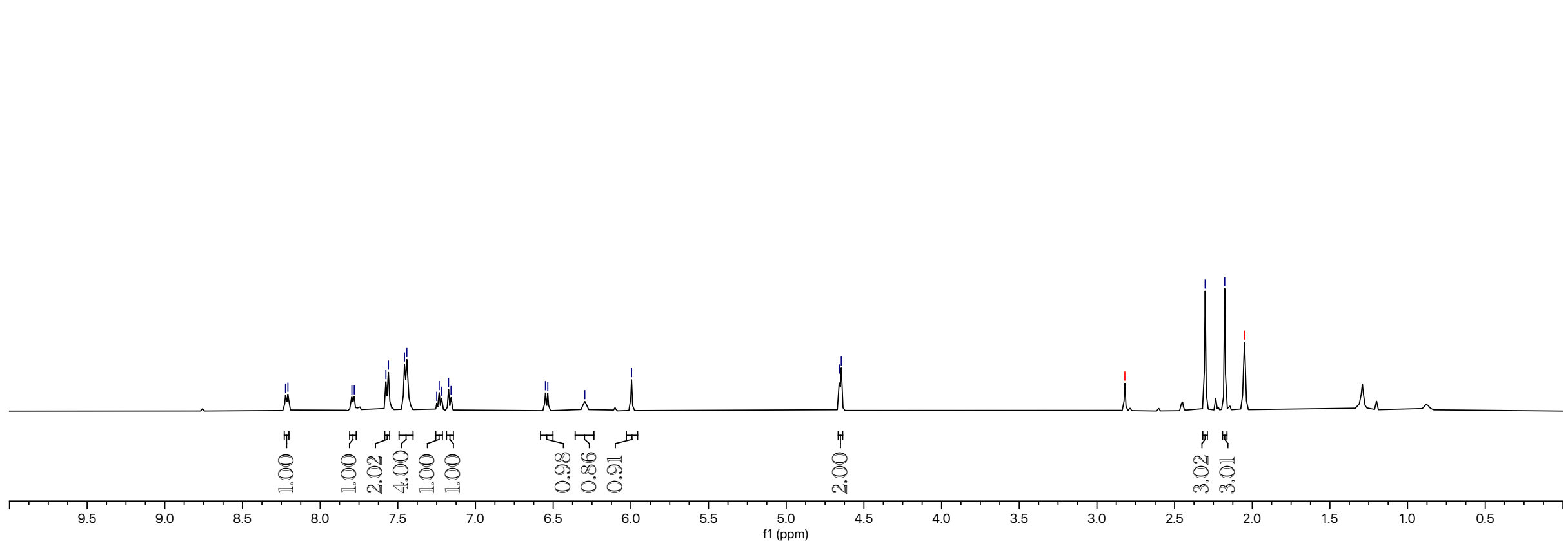
8.22
8.21
7.80
7.78
7.58
7.56
7.46
7.44
7.25
7.23
7.22
7.17
7.16
6.55
6.53
6.30
6.00
4.66
4.65
2.82 H2O
2.30
2.18
2.05 acetone
1.00
1.00
2.02
4.00
1.00
1.00
0.98
0.86
0.91
2.00
3.02
3.01
f1 (ppm)

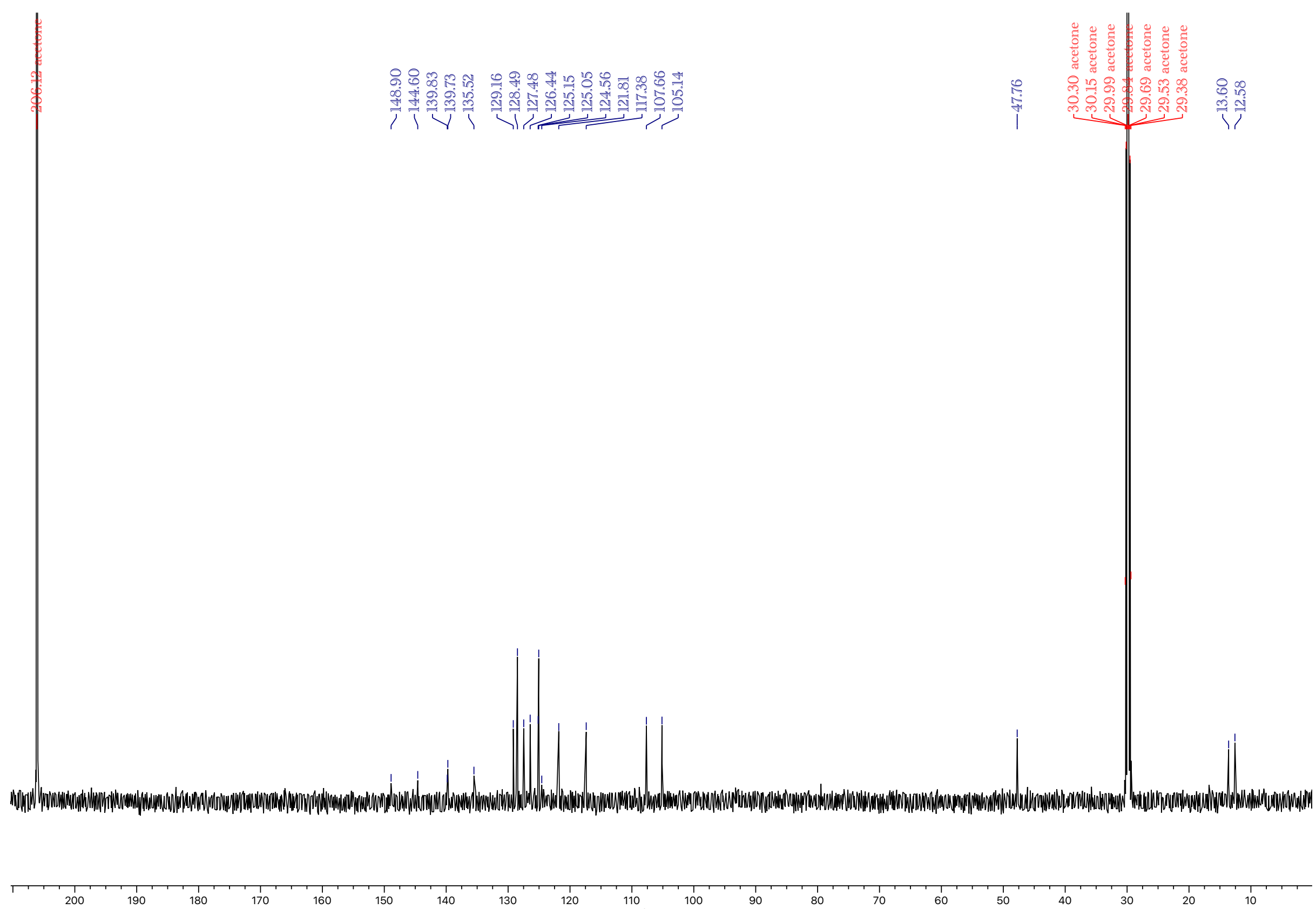
148.90
144.60
139.83
139.73
135.52
129.16
128.49
127.48
126.44
125.15
125.05
124.56
121.81
117.38
107.66
105.14
47.76
30.30 acetone
30.15 acetone
29.99 acetone
29.69 acetone
29.53 acetone
29.38 acetone
13.60
12.58
f1 (ppm)

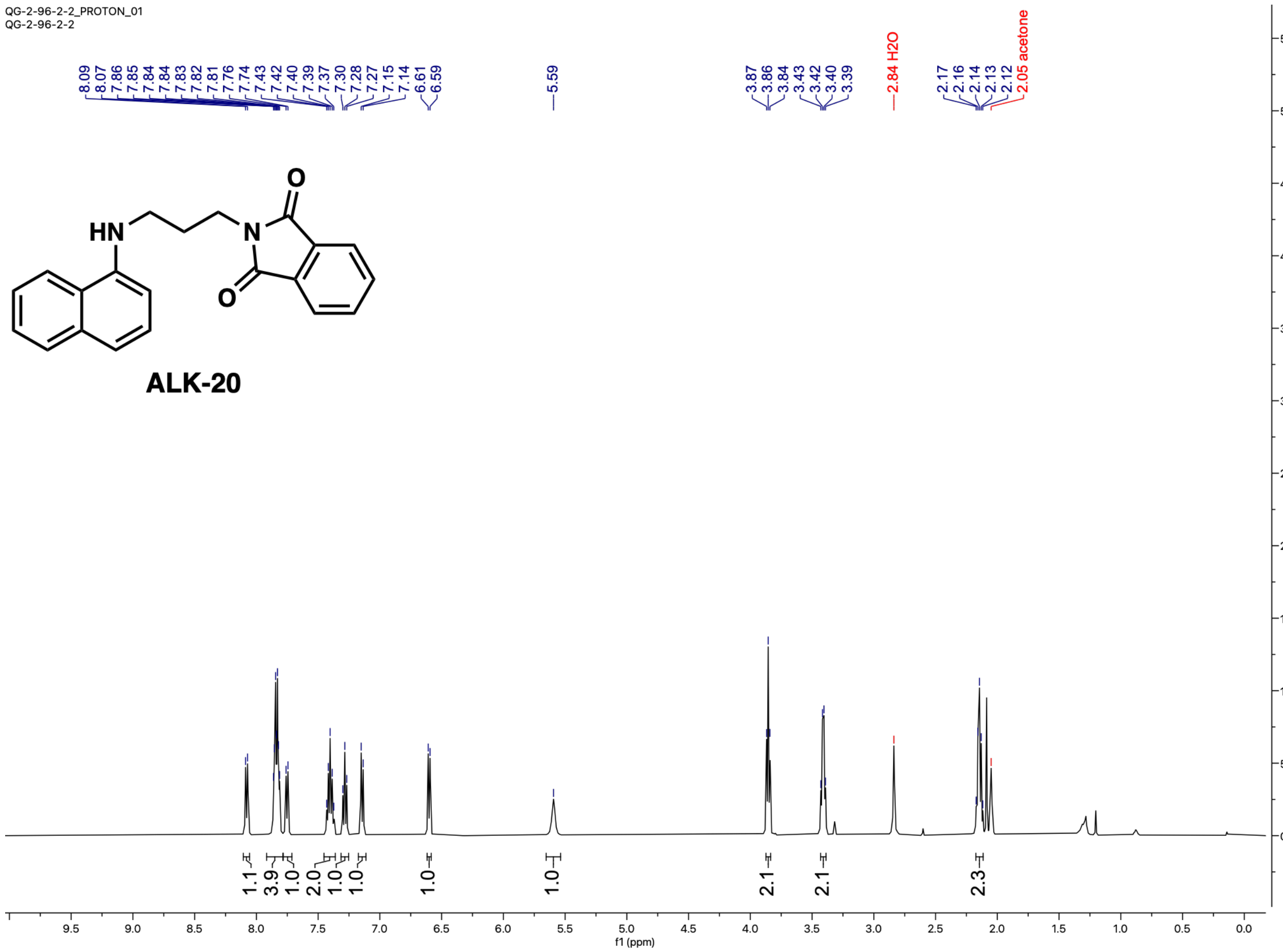

ALK-20
QG-2-96-2-2_PROTON_01
QG-2-96-2-2
8.09 8.07 7.86 7.85 7.84 7.84 7.83 7.82 7.81 7.76 7.74 7.43 7.42 7.40 7.39 7.37 7.30 7.28 7.27 7.15 7.14 6.61 6.59
5.59
3.87 3.86 3.84 3.43 3.42 3.40 3.39
2.84 H2O
2.17 2.16 2.14 2.13 2.12
2.05 acetone
1.1 3.9 1.0 2.0 1.0 1.0 1.0 1.0 2.1 2.1 2.3
f1 (ppm)

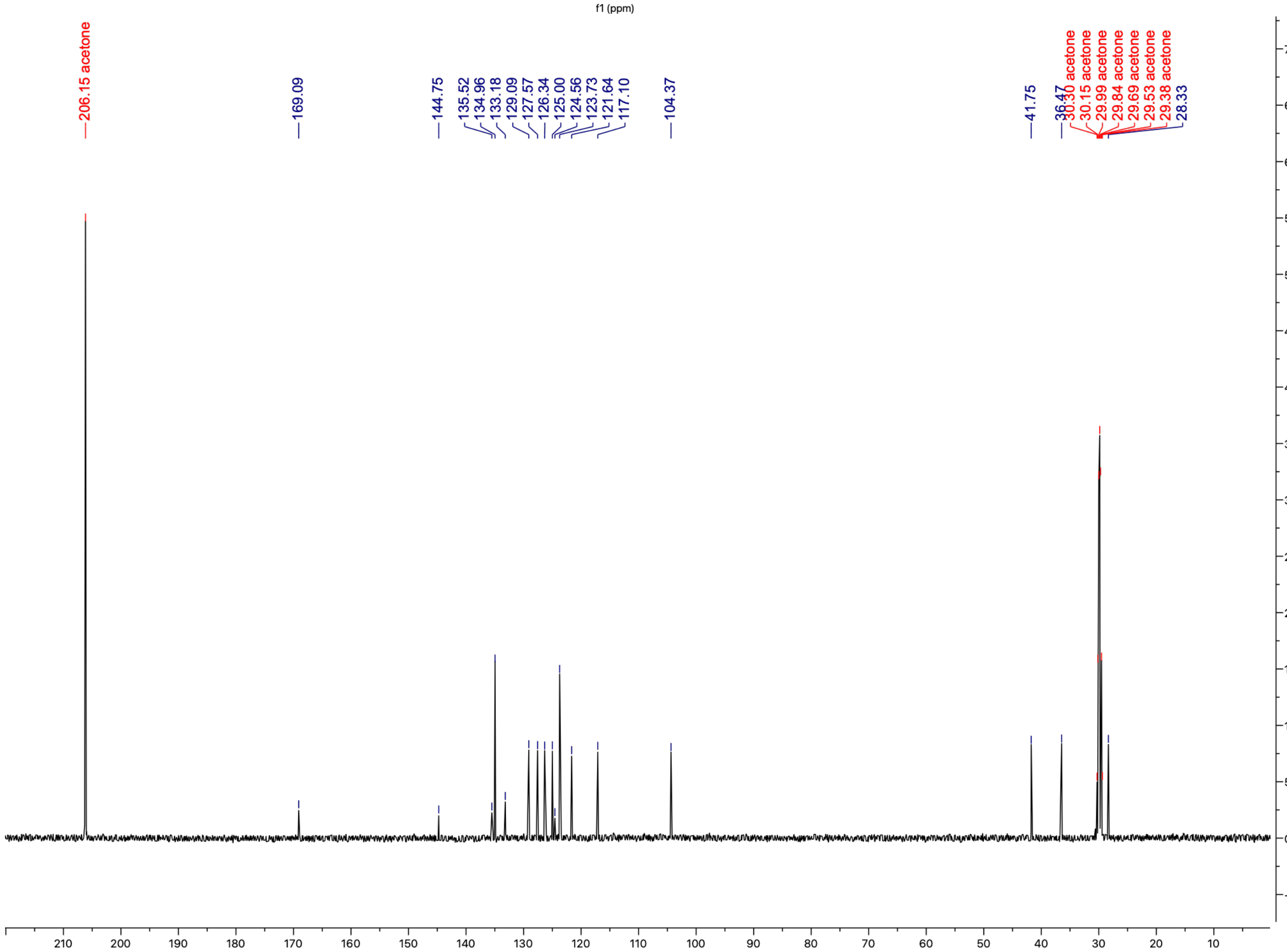
206.15 acetone
169.09
144.75
135.52 134.96 133.18 129.09 127.57 126.34 125.00 124.56 123.73 121.64 117.10
104.37
41.75
36.47
30.30 acetone
30.15 acetone
29.99 acetone
29.84 acetone
29.69 acetone
29.53 acetone
29.38 acetone
28.33
f1 (ppm)

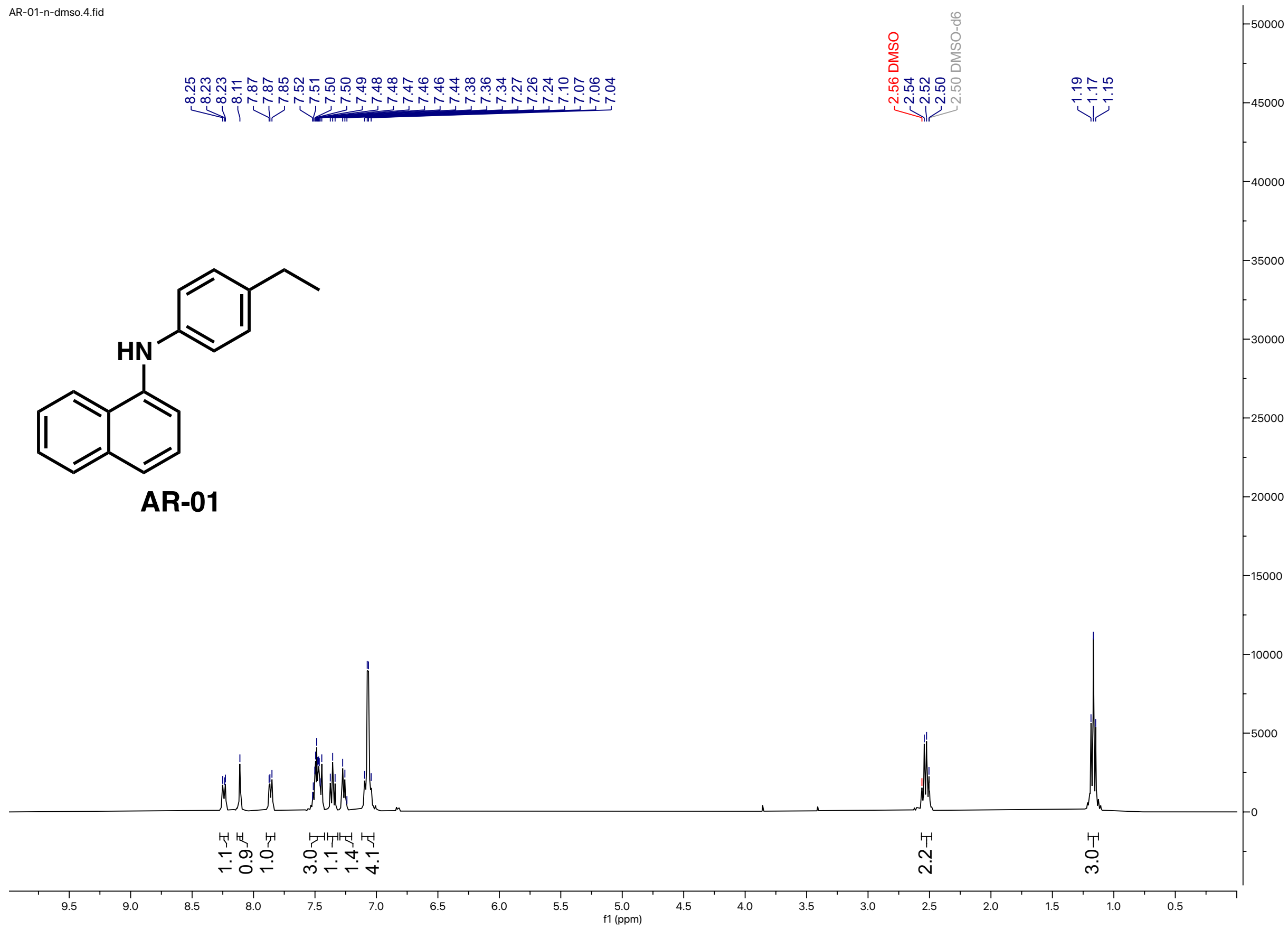
AR-01-n-dmso.4.fid
8.25 8.23 8.23 8.11 7.87 7.87 7.85 7.52 7.51 7.50 7.50 7.49 7.48 7.48 7.47 7.46 7.44 7.38 7.36 7.34 7.27 7.26 7.24 7.10 7.07 7.06 7.04
2.56 DMSO 2.54 2.52 2.50 2.50 DMSO-d6
1.19 1.17 1.15
HN
AR-01
1.1 0.9 1.0 3.0 1.1 1.4 4.1 2.2 3.0
f1 (ppm)

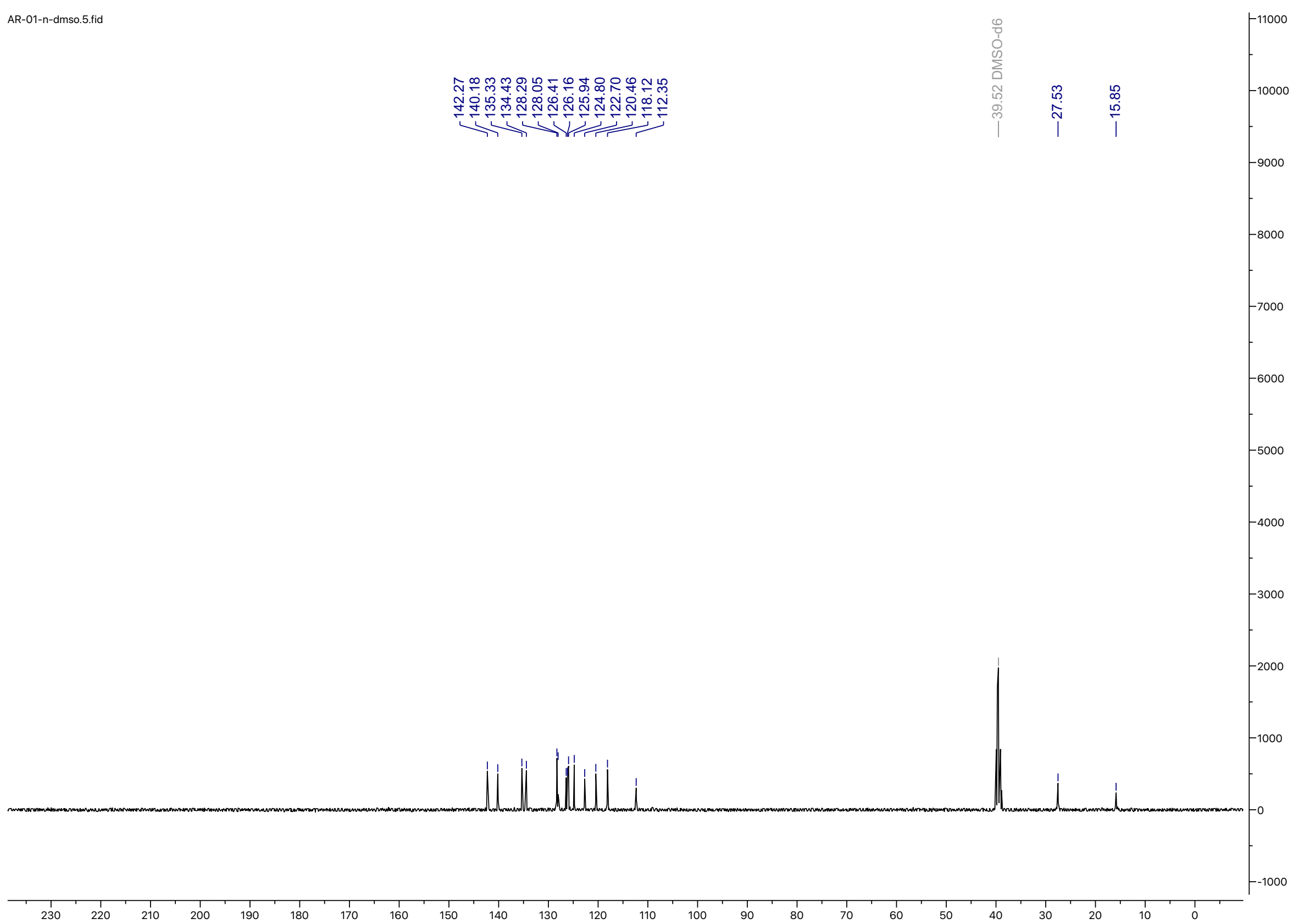
AR-01-n-dmso.5.fid
142.27 140.18 135.33 134.43 128.29 128.05 126.41 126.16 125.94 124.80 122.70 120.46 118.12 112.35
39.52 DMSO-d6
27.53
15.85
f1 (ppm)

AR-02

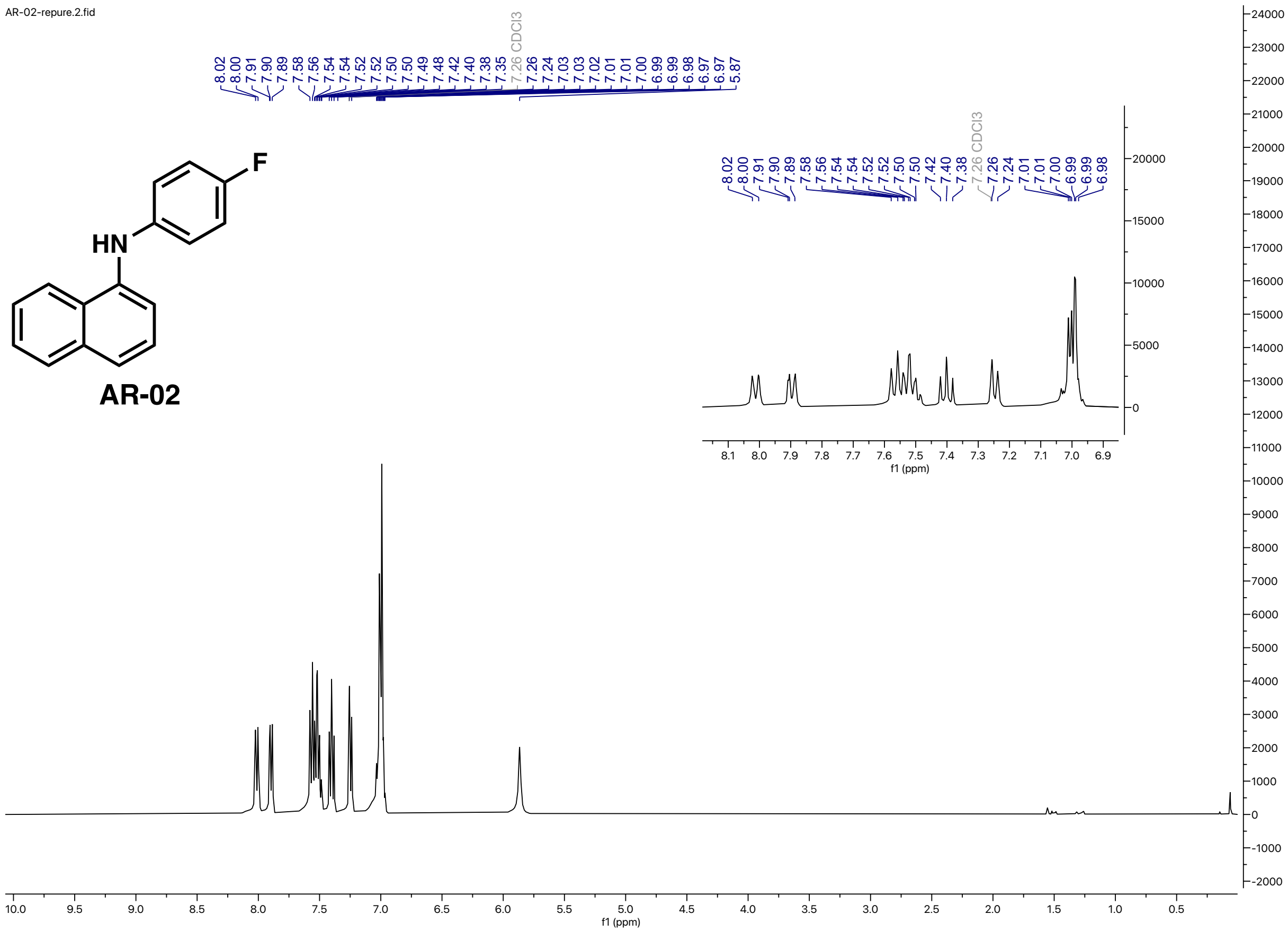
HN
F
AR-02-repure.2.fid
8.02
8.00
7.91
7.90
7.89
7.58
7.56
7.54
7.54
7.52
7.52
7.50
7.50
7.49
7.48
7.42
7.40
7.38
7.35
7.26 CDCl3
7.26
7.24
7.03
7.03
7.02
7.01
7.01
7.00
6.99
6.99
6.98
6.97
6.97
5.87
f1 (ppm)

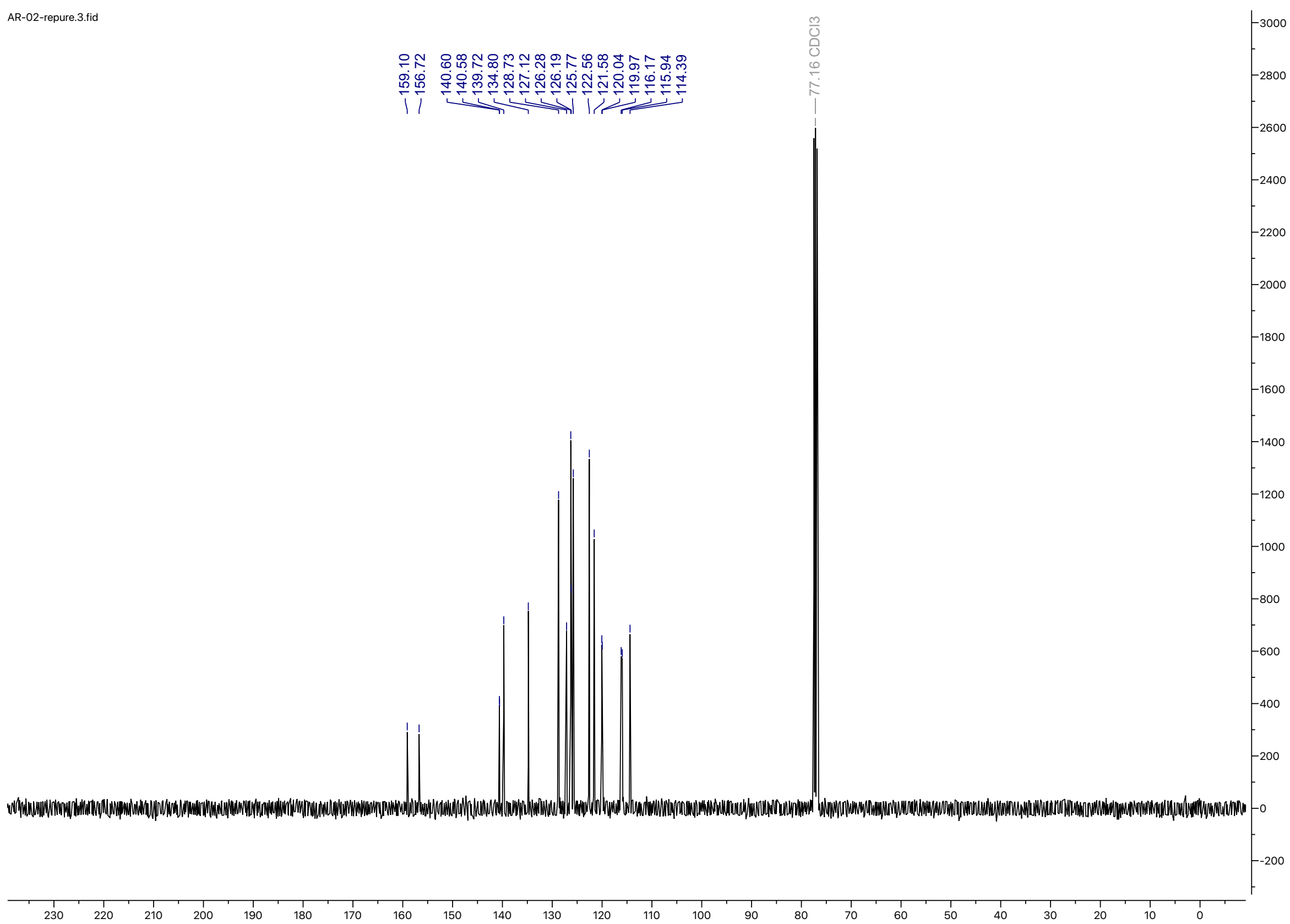
AR-02-repure.3.fid
159.10
156.72
140.60
140.58
139.72
134.80
128.73
127.12
126.28
126.19
125.77
122.56
121.58
120.04
119.97
116.17
115.94
114.39
77.16 CDCl3
f1 (ppm)

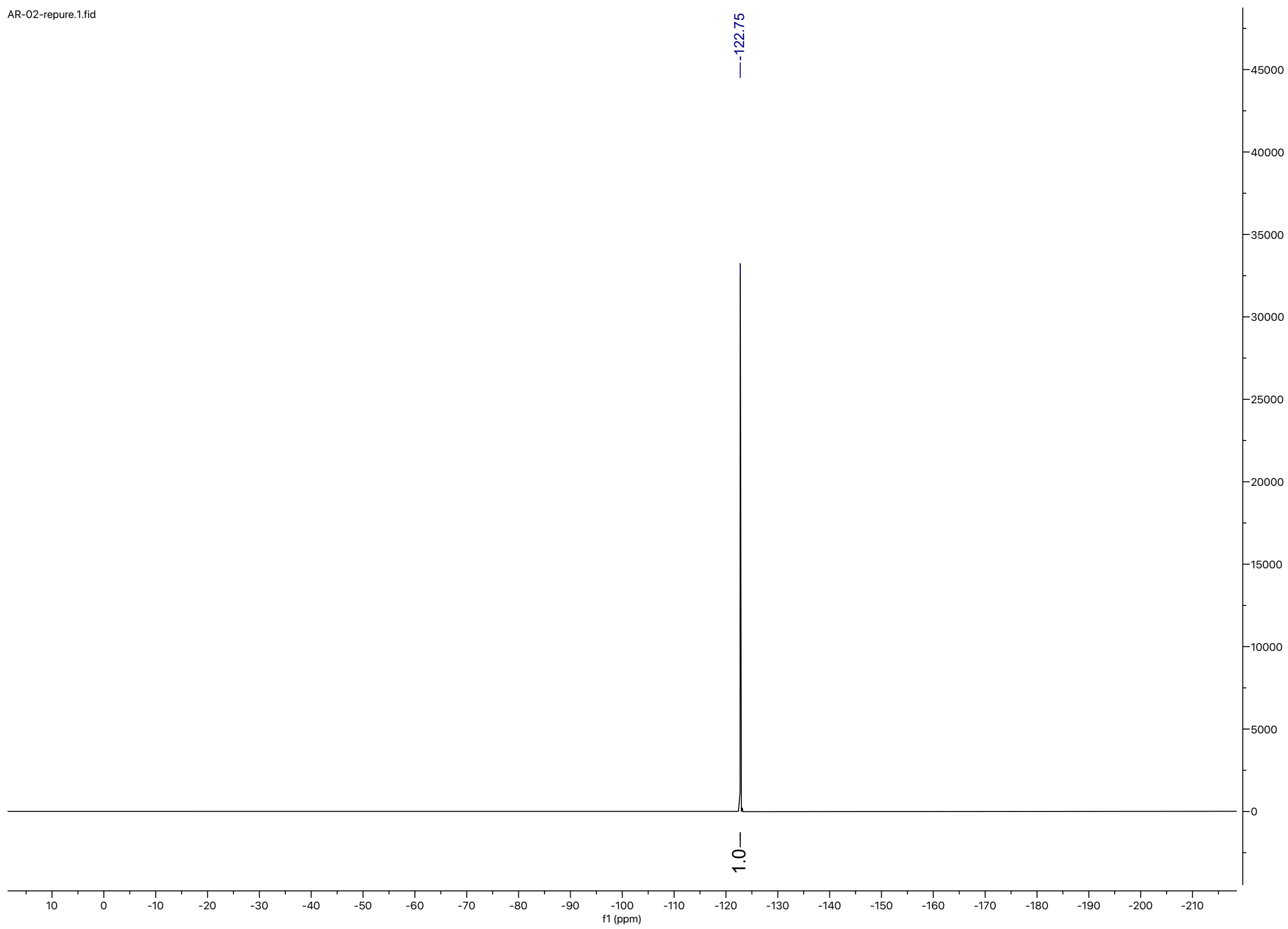
AR-02-repure.1.fid
-122.75
1.0
f1 (ppm)

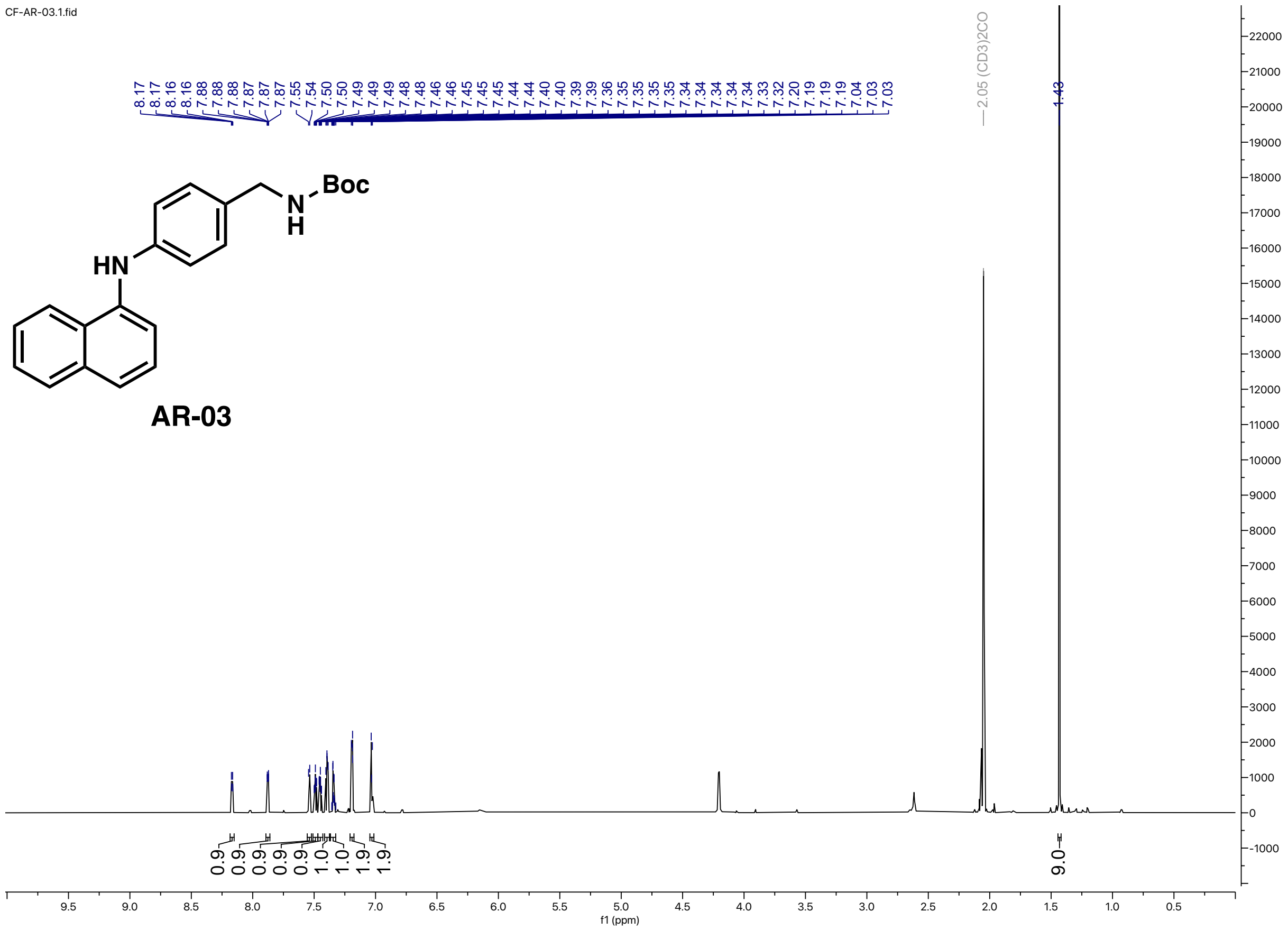

CF-AR-03.1.fid
8.17 8.17 8.16 8.16 7.88 7.88 7.88 7.87 7.87 7.87 7.55 7.54 7.50 7.50 7.49 7.49 7.48 7.48 7.46 7.46 7.45 7.45 7.45 7.44 7.40 7.40 7.39 7.39 7.36 7.35 7.35 7.35 7.35 7.34 7.34 7.34 7.34 7.33 7.32 7.20 7.19 7.19 7.04 7.03 7.03
2.05 (CD3)2CO
1.43
Boc
N
H
HN
AR-03
0.9 0.9 0.9 0.9 0.9 1.0 1.0 1.9 1.9
9.0
f1 (ppm)

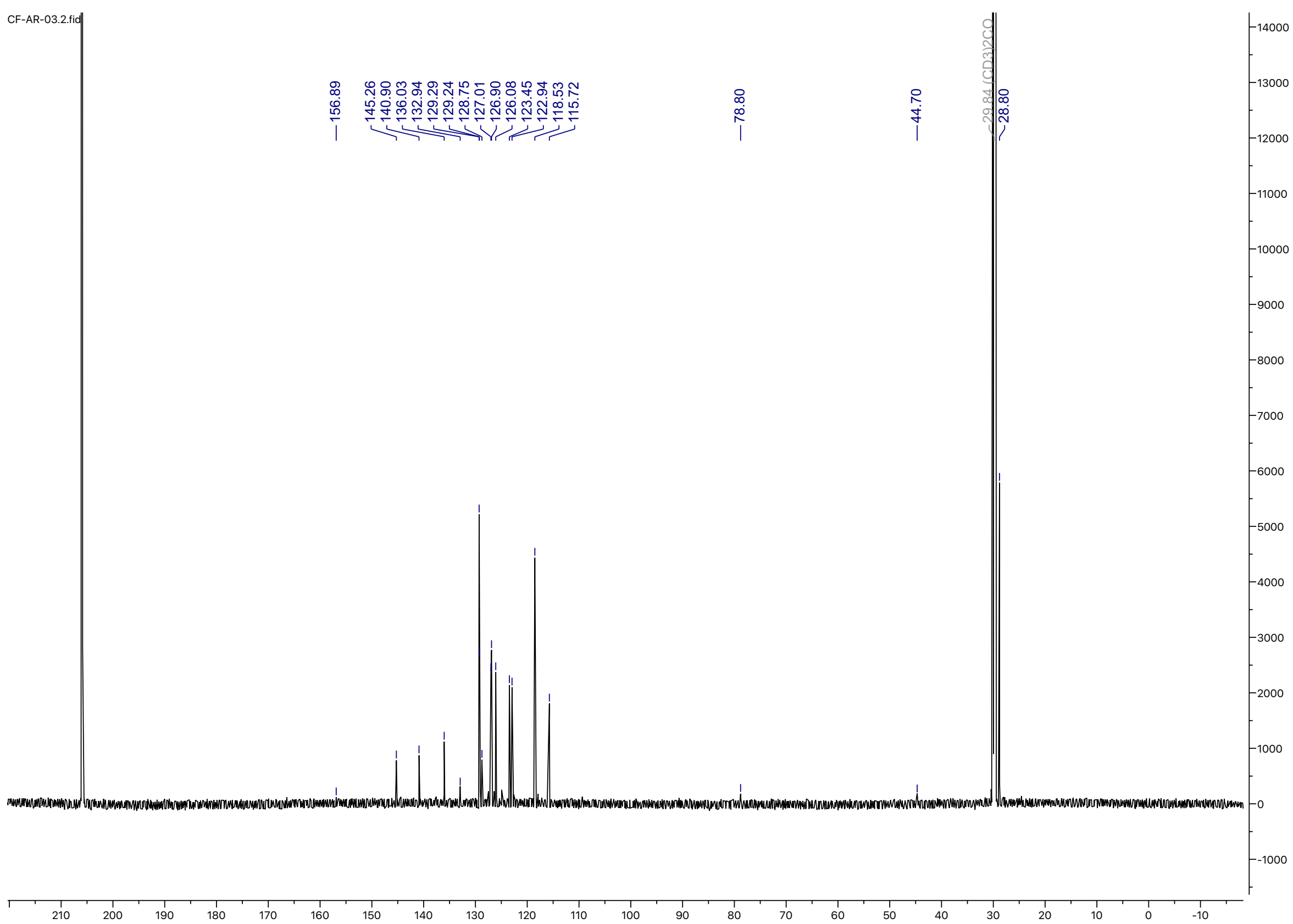

CF-AR-03.2.fid
156.89
145.26 140.90 136.03 132.94 129.29 129.24 128.75 127.01 126.90 126.08 123.45 122.94 118.53 115.72
78.80
44.70
29.84 (CD3)2CO
28.80
f1 (ppm)

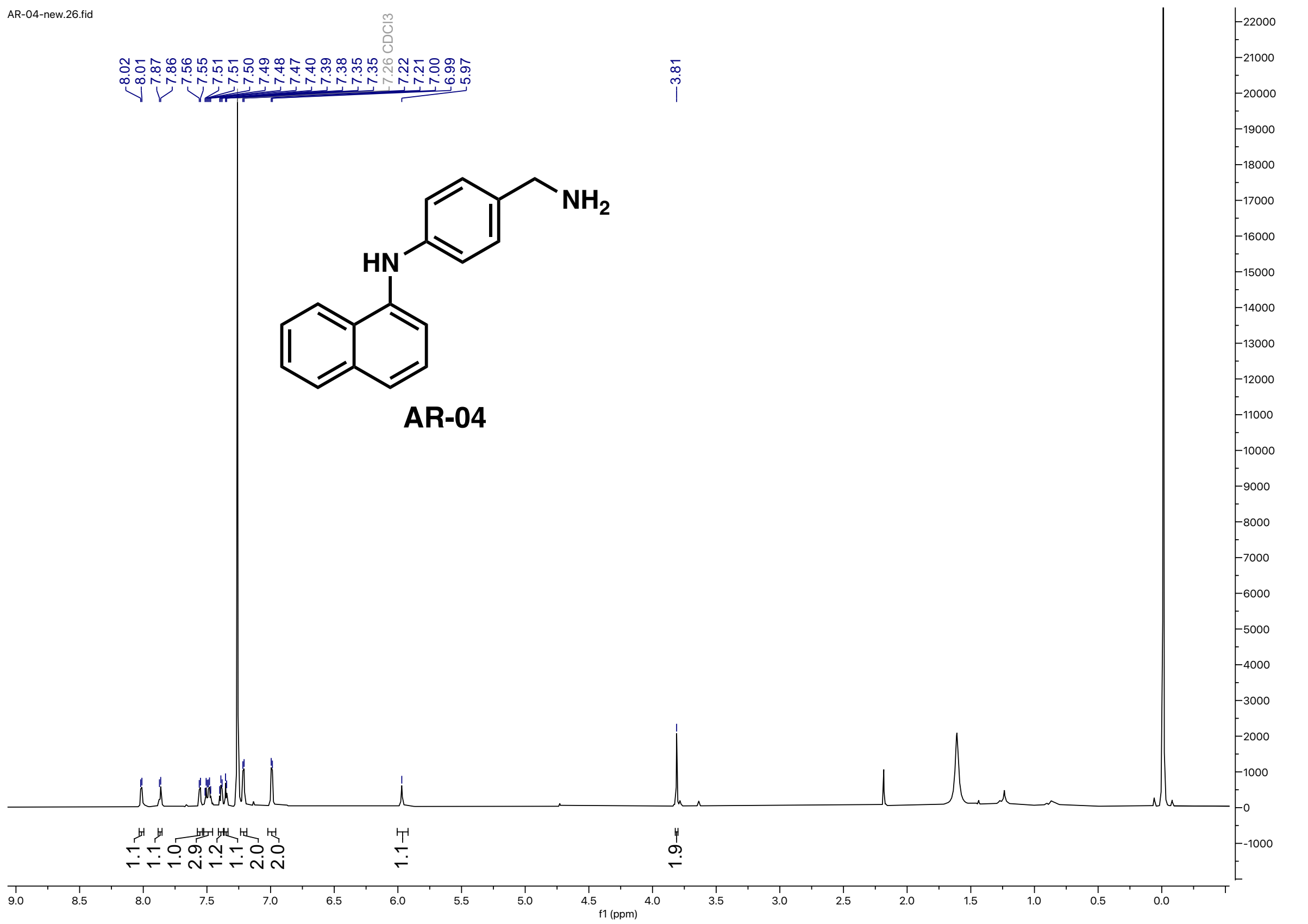
AR-04-new.26.fid
AR-04
f1 (ppm)

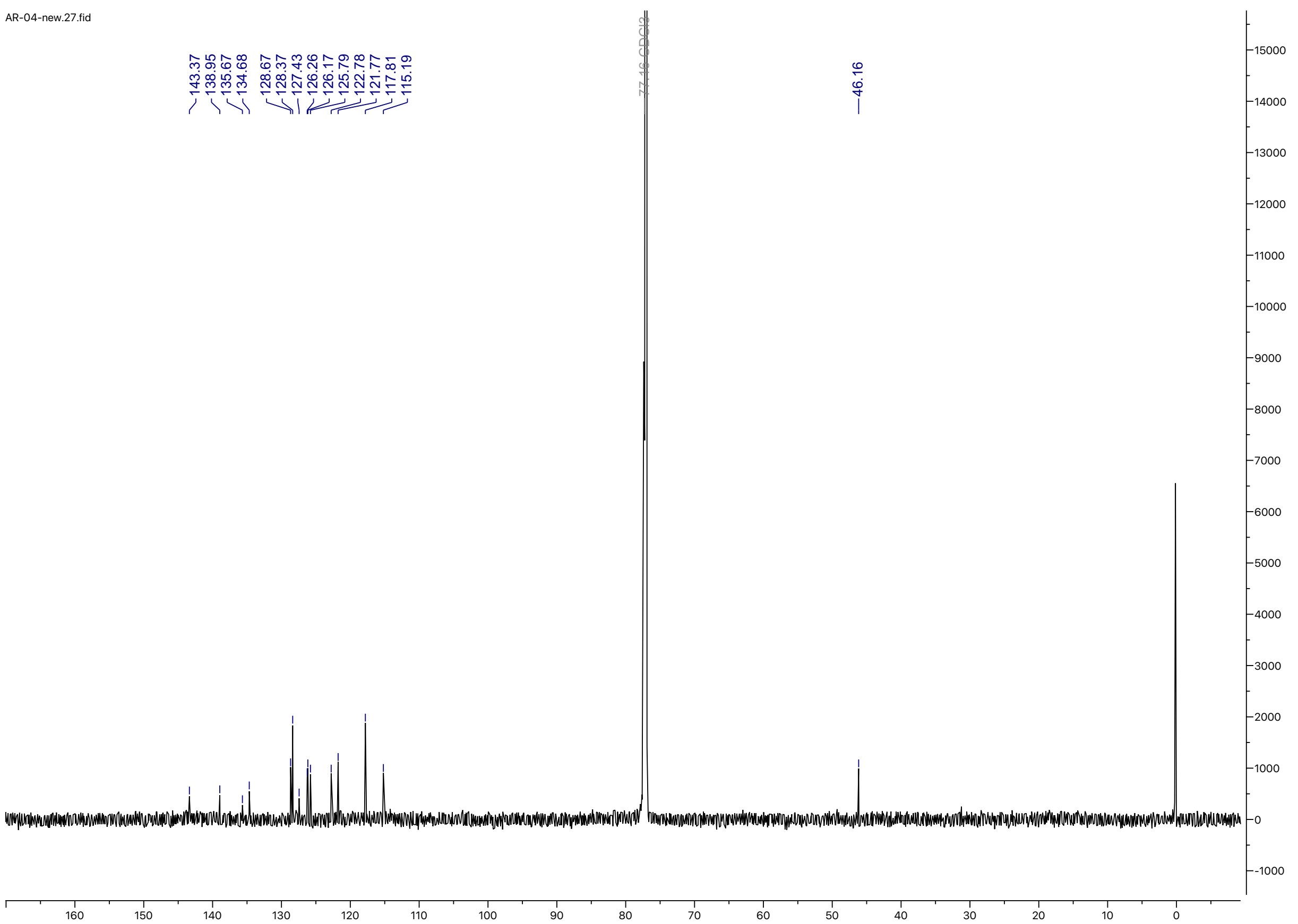
AR-04-new.27.fid
f1 (ppm)

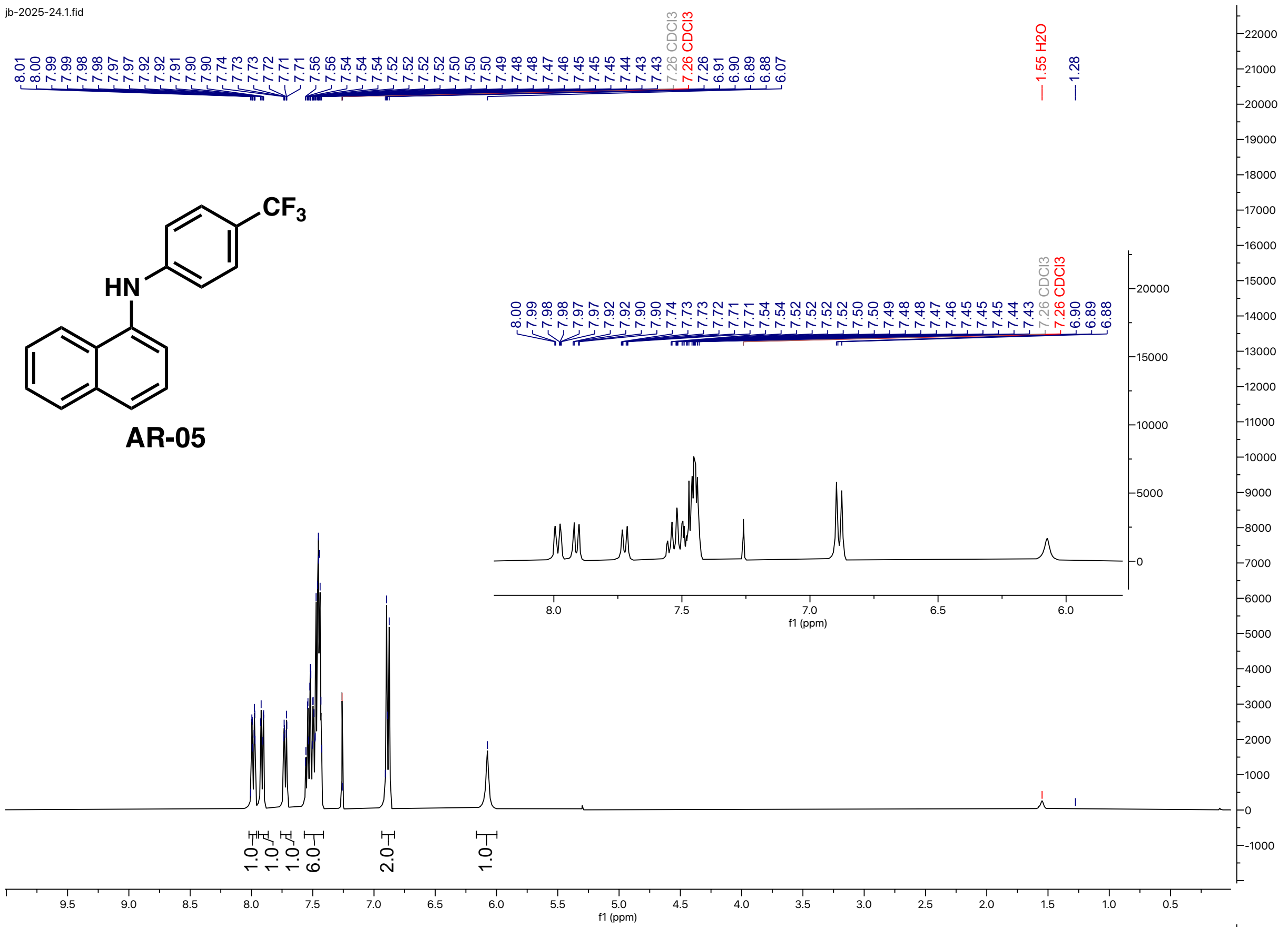
jb-2025-24.1.fid
HN
CF_3
AR-05
1.55 H2O
1.28
f1 (ppm)

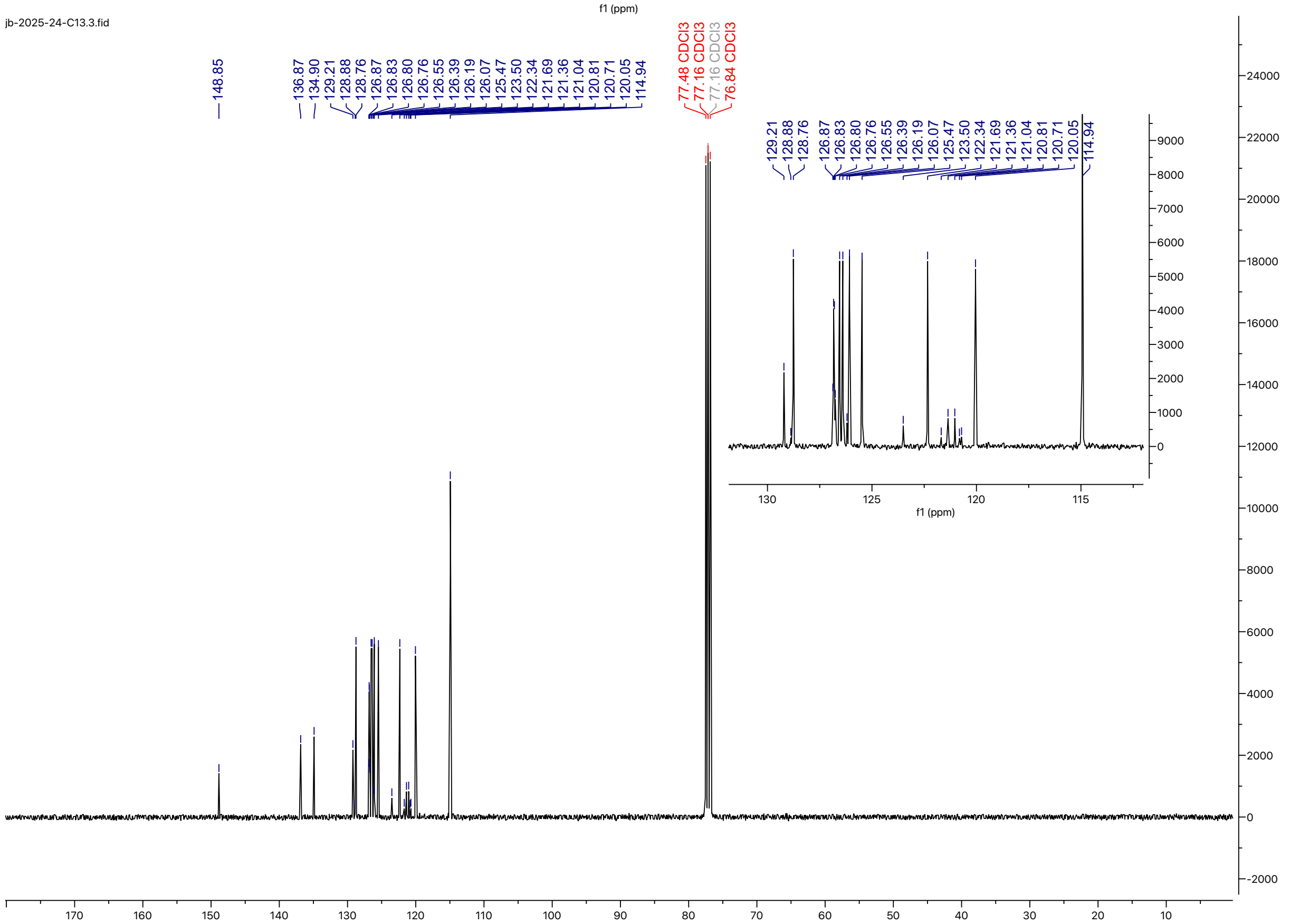
jb-2025-24-C13.3.fid
148.85
136.87
134.90
129.21
128.88
128.76
77.48 CDCl3
77.16 CDCl3
76.84 CDCl3
114.94
f1 (ppm)

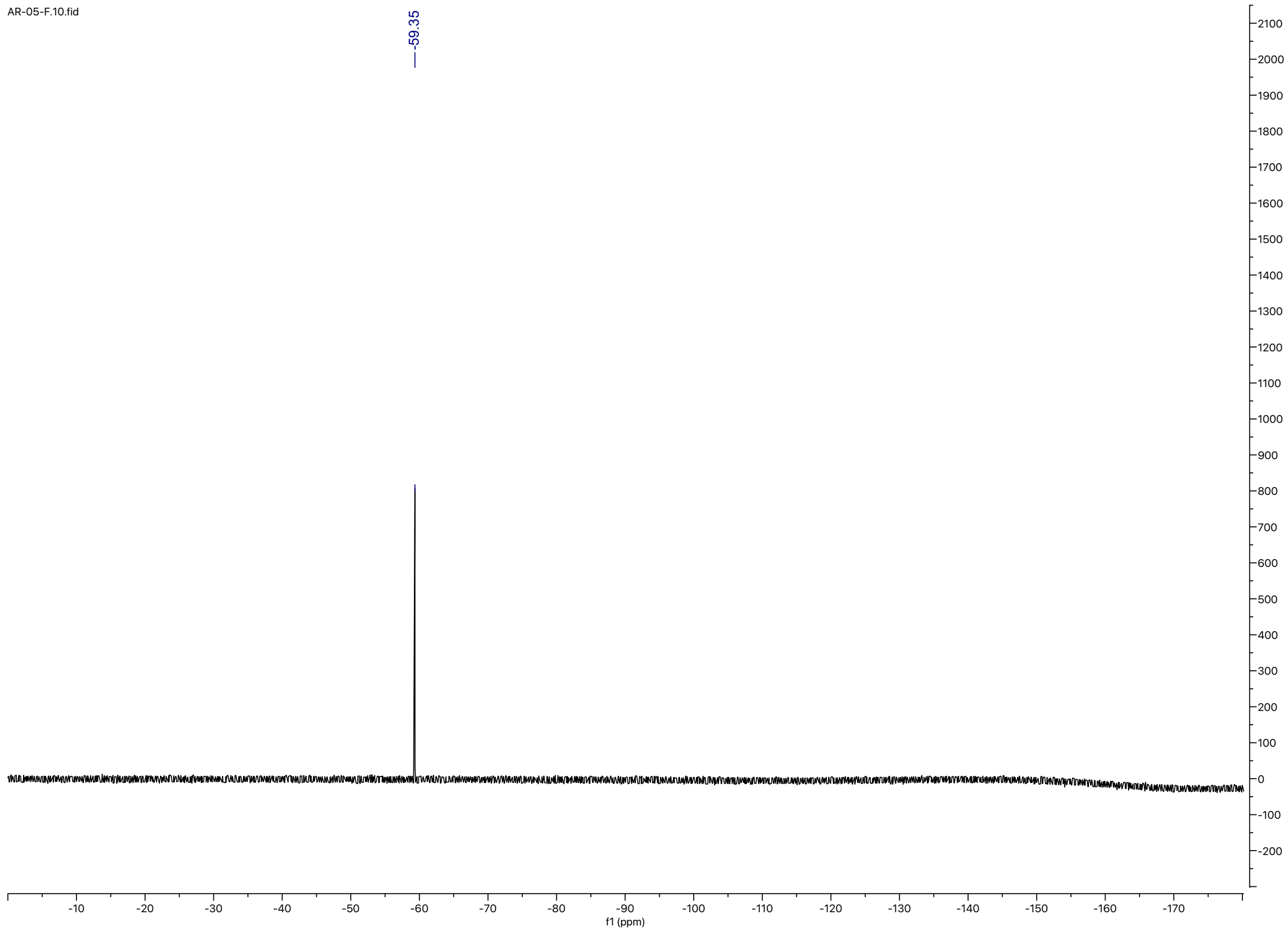

AR-05-F.10.fid
-59.35
f1 (ppm)

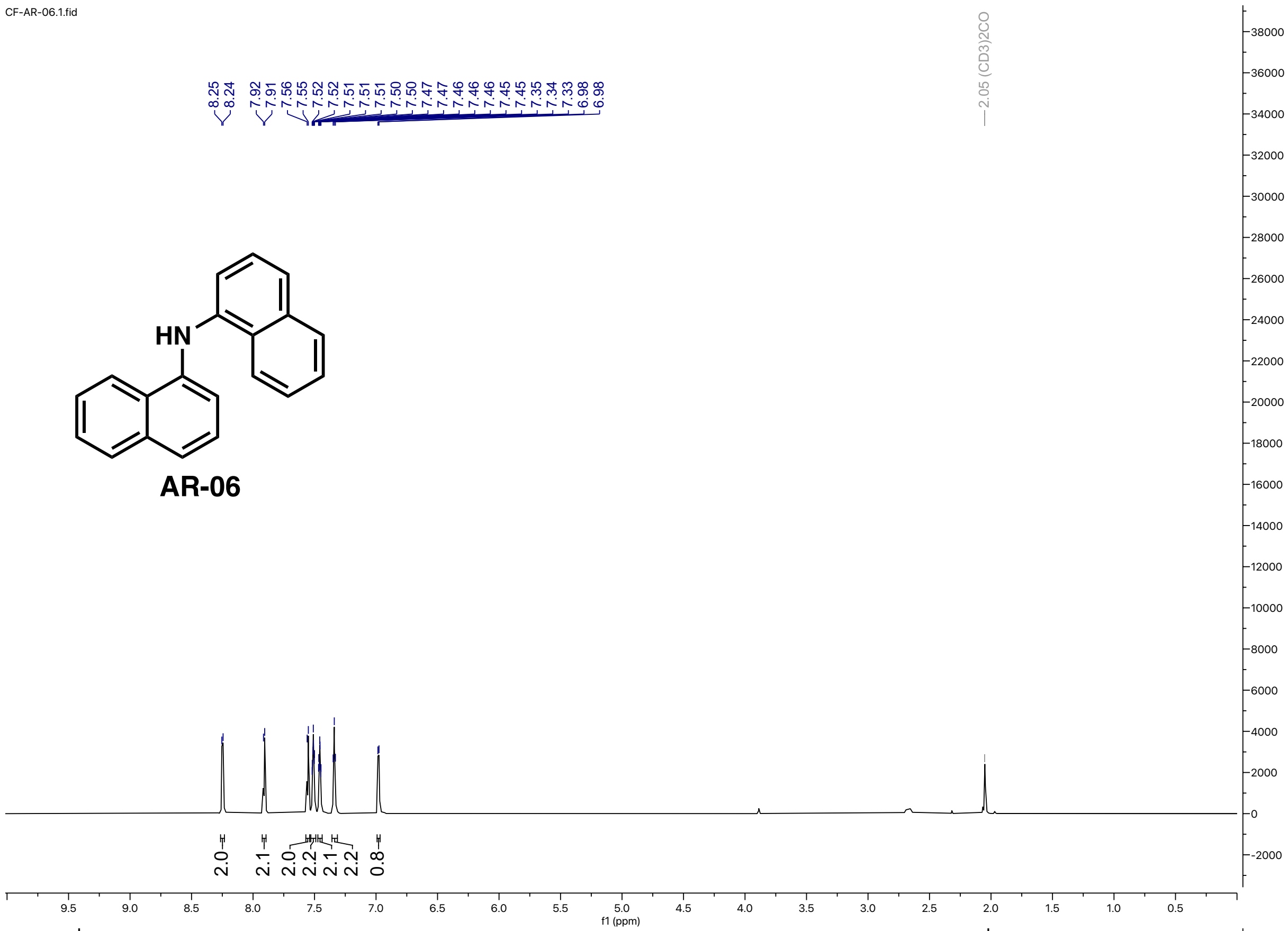
CF-AR-06.1.fid
8.25
8.24
7.92
7.91
7.56
7.55
7.52
7.52
7.51
7.51
7.51
7.50
7.50
7.47
7.47
7.46
7.46
7.46
7.45
7.45
7.35
7.34
7.33
6.98
6.98
2.05 (CD3)2CO
2.0
2.1
2.0
2.2
2.1
2.2
0.8
f1 (ppm)
HN

AR-06

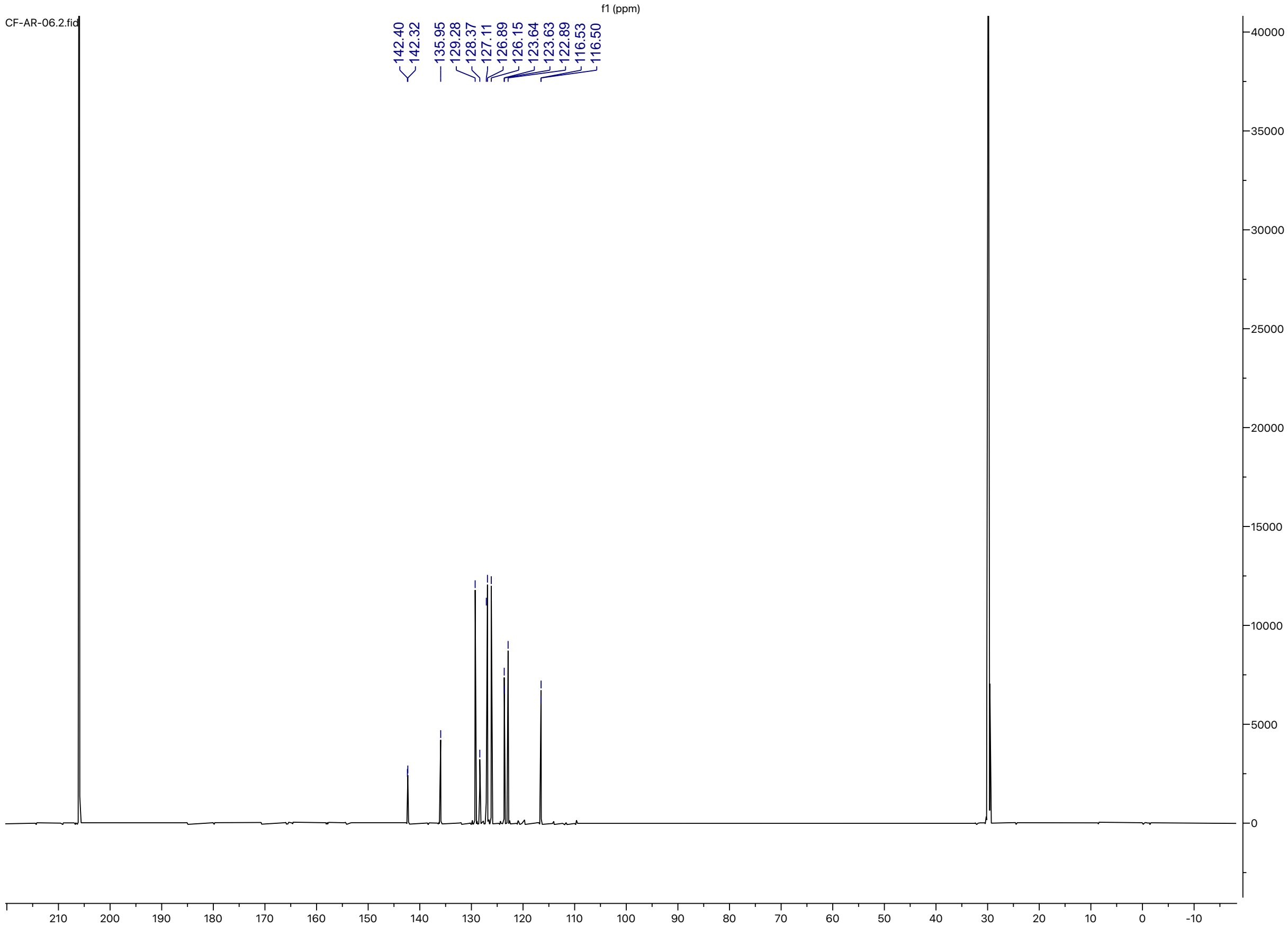
CF-AR-06.2.fid
142.40
142.32
135.95
129.28
128.37
127.11
126.89
126.15
123.64
123.63
122.89
116.53
116.50
f1 (ppm)

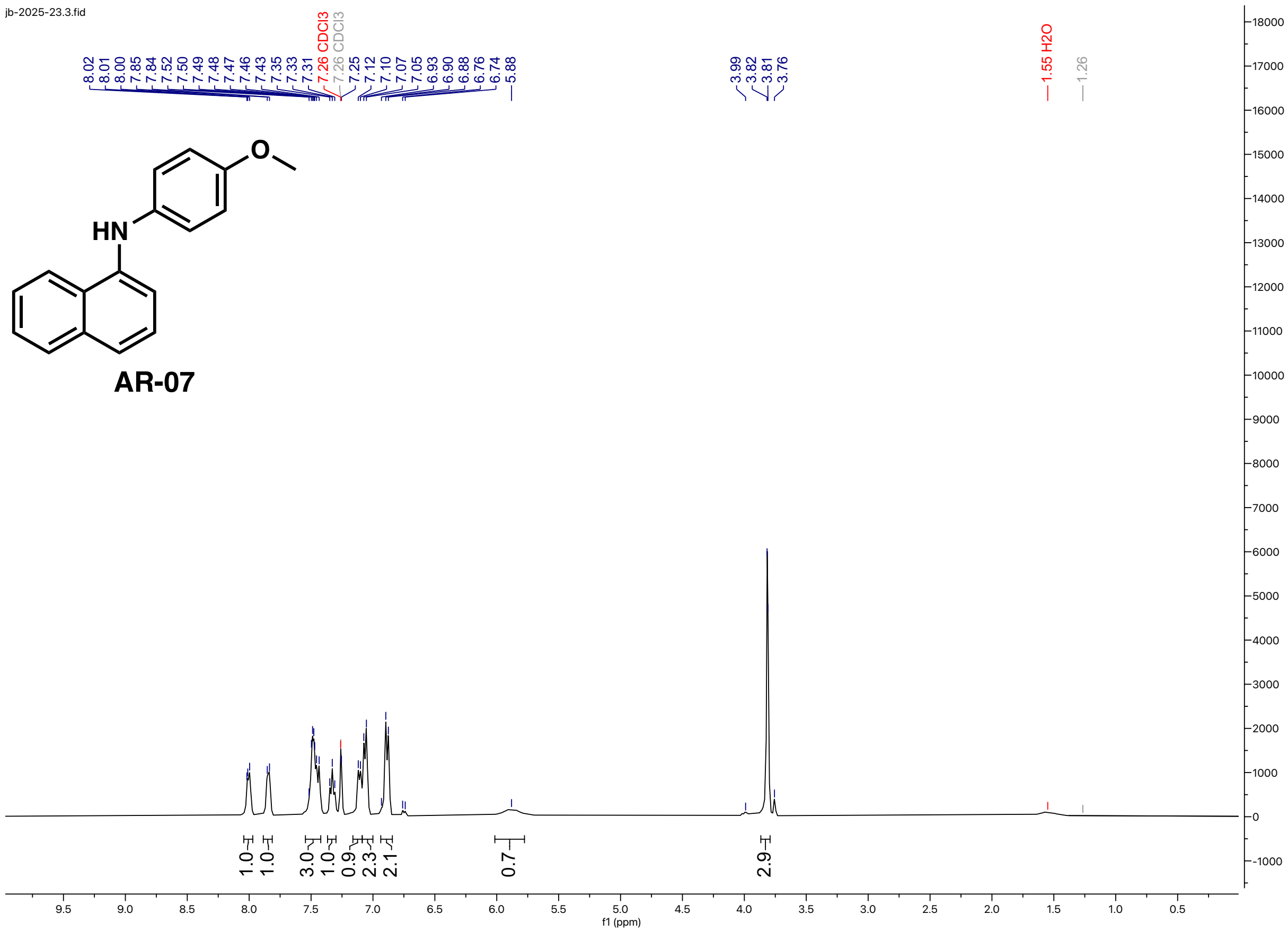
jb-2025-23.3.fid
8.02 8.01 8.00 7.85 7.84 7.52 7.50 7.49 7.48 7.47 7.46 7.43 7.35 7.33 7.31 7.26 CDCl3 7.26 CDCl3 7.25 7.12 7.10 7.07 7.05 6.93 6.90 6.88 6.76 6.74 5.88
3.99 3.82 3.81 3.76
1.55 H2O
1.26
HN
O
AR-07
1.0 1.0 3.0 1.0 0.9 2.3 2.1 0.7 2.9
f1 (ppm)

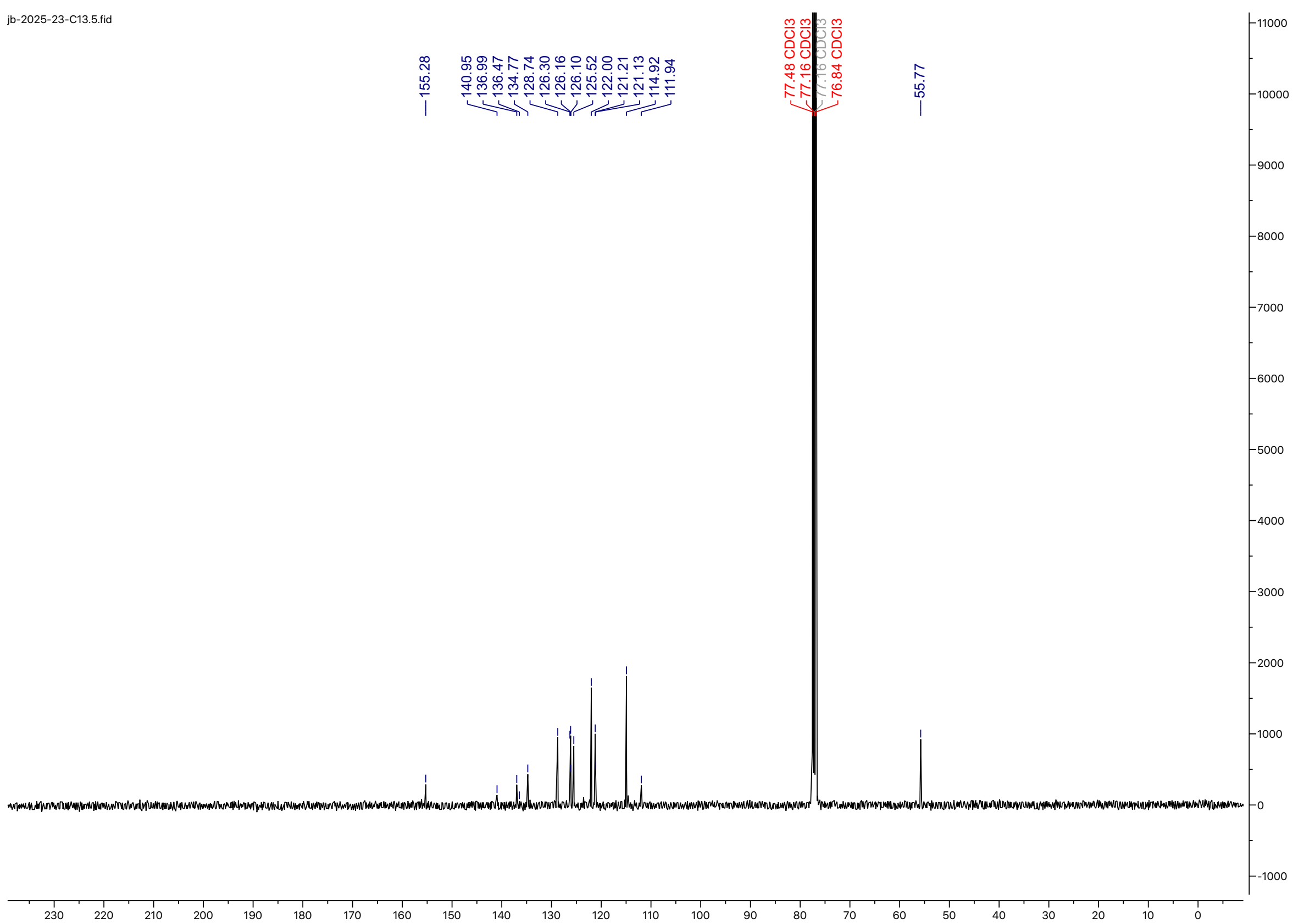
jb-2025-23-C13.5.fid
155.28
140.95 136.99 136.47 134.77 128.74 126.30 126.16 126.10 125.52 122.00 121.21 121.13 114.92 111.94
77.48 CDCl3 77.16 CDCl3 77.16 CDCl3 76.84 CDCl3
55.77
f1 (ppm)

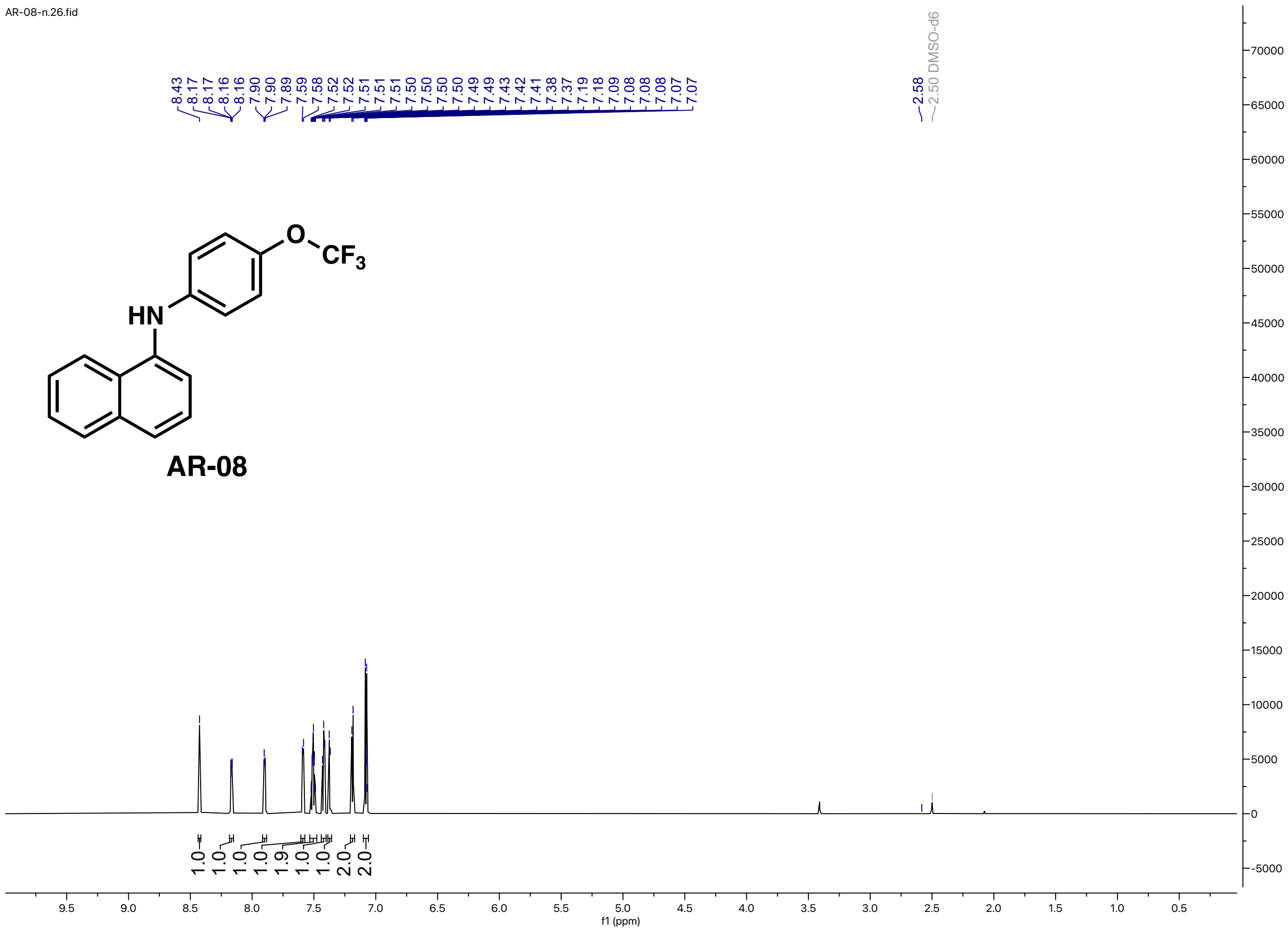
AR-08-n.26.fid
8.43
8.17
8.17
8.16
8.16
7.90
7.90
7.89
7.59
7.58
7.52
7.52
7.51
7.51
7.51
7.50
7.50
7.50
7.50
7.49
7.49
7.43
7.42
7.41
7.38
7.37
7.19
7.18
7.09
7.08
7.08
7.08
7.07
7.07
2.58
2.50 DMSO-d6
HN
O
CF3
AR-08
1.0
1.0
1.0
1.0
1.9
1.0
1.0
2.0
2.0
f1 (ppm)

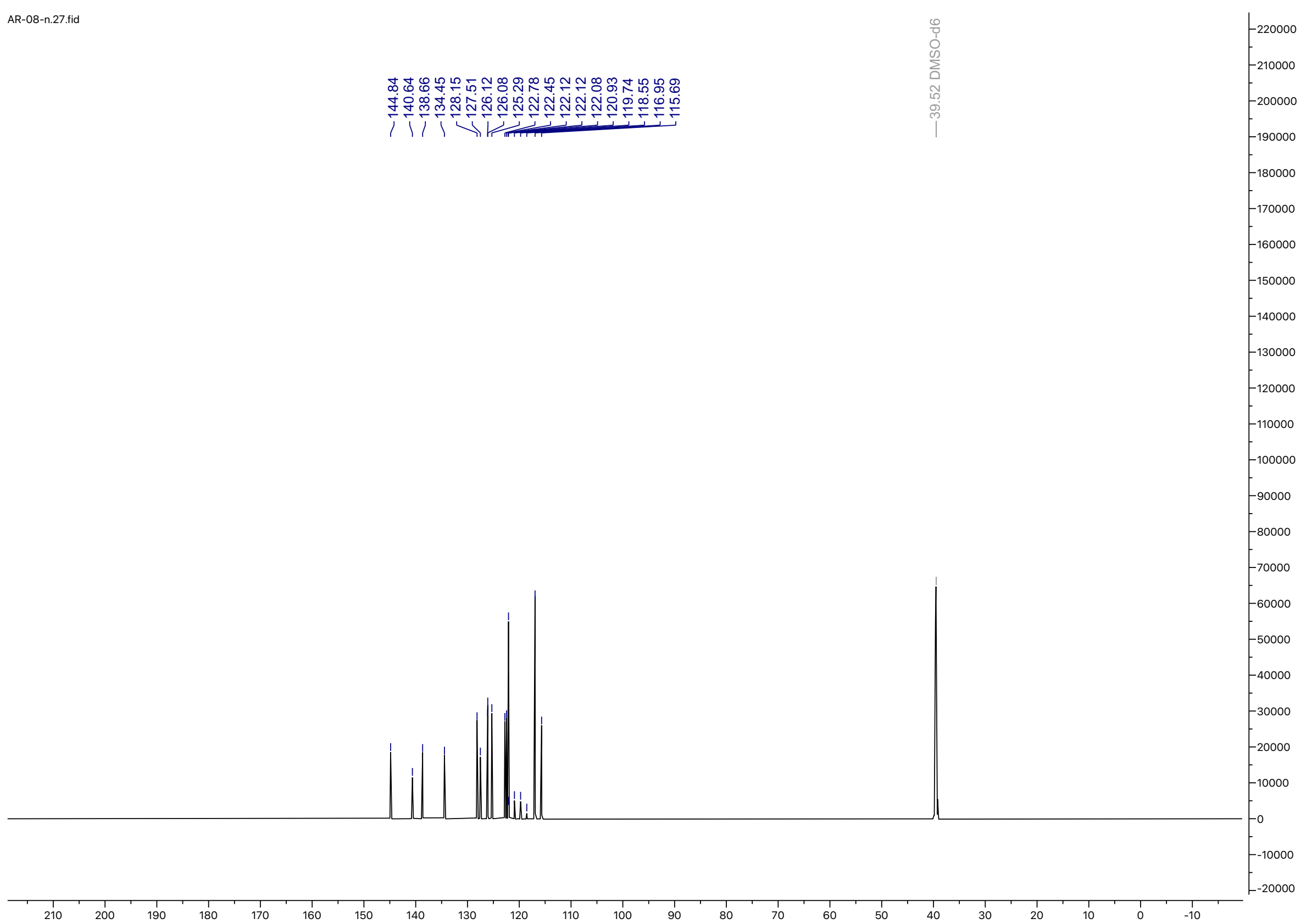
AR-08-n.27.fid
144.84
140.64
138.66
134.45
128.15
127.51
126.12
126.08
125.29
122.78
122.45
122.12
122.12
122.08
120.93
119.74
118.55
116.95
115.69
39.52 DMSO-d6
f1 (ppm)

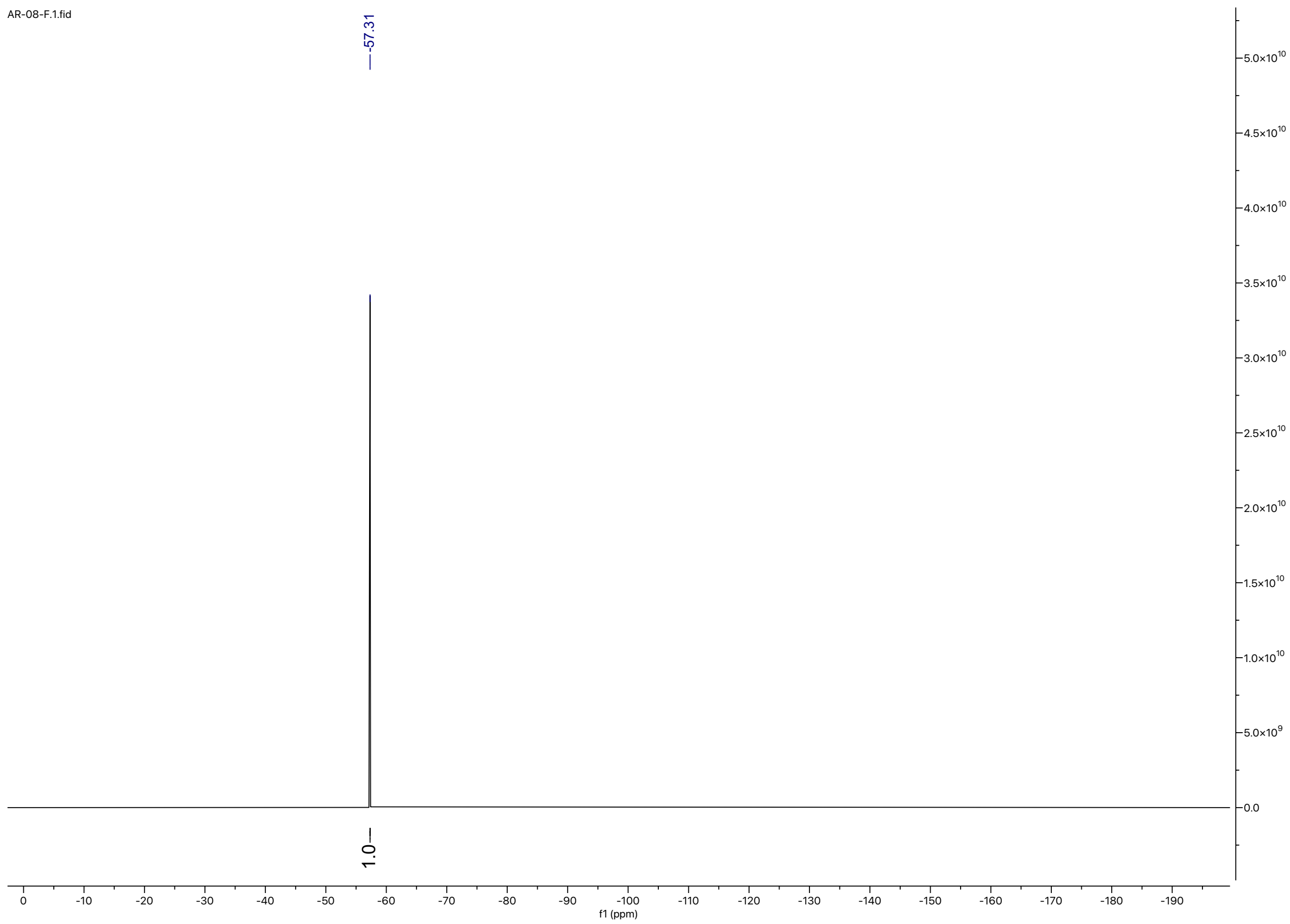
AR-08-F.1.fid
-57.31
1.0
f1 (ppm)

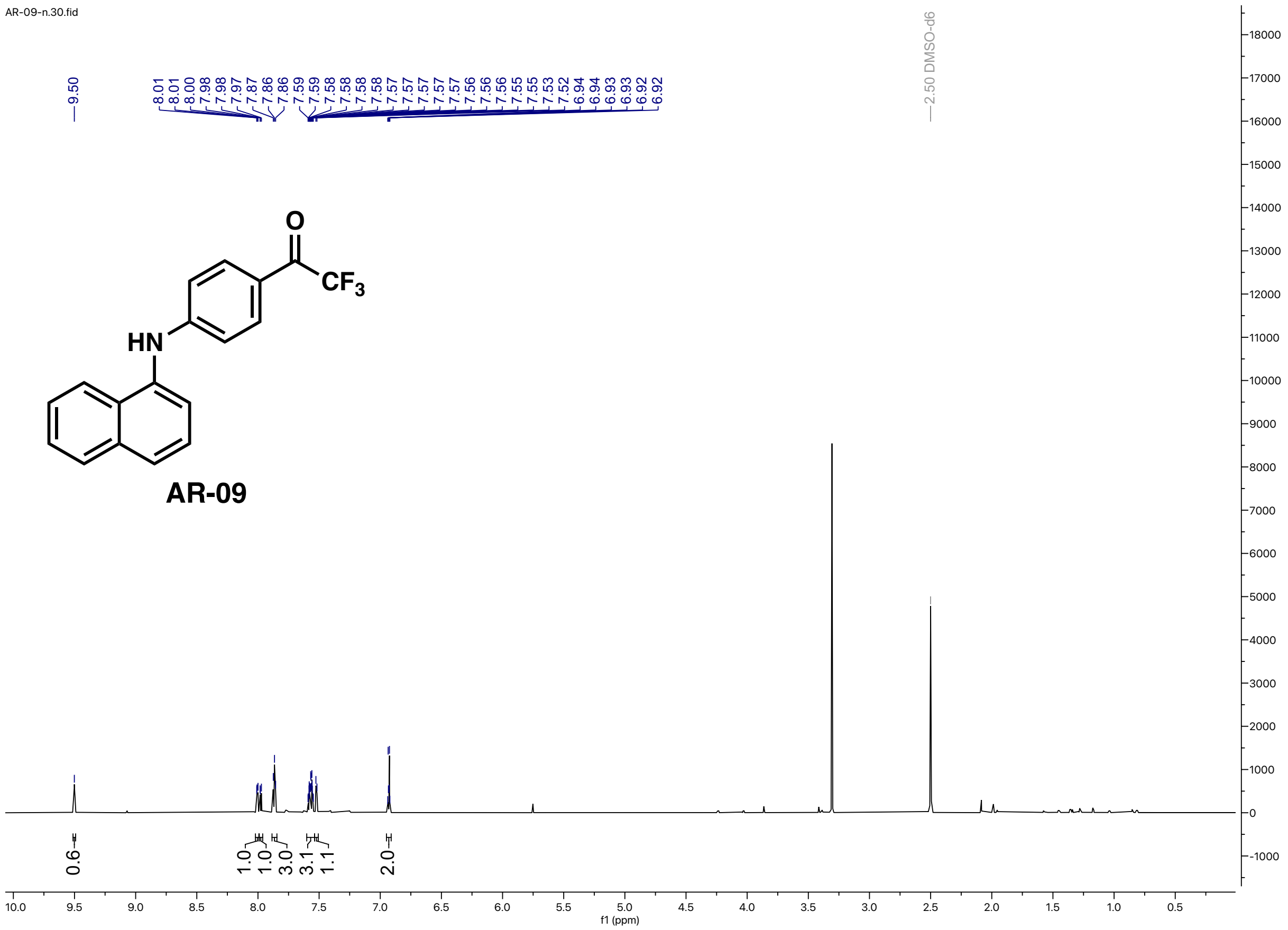
AR-09-n.30.fid
9.50
8.01
8.01
8.00
7.98
7.98
7.97
7.87
7.86
7.86
7.59
7.59
7.58
7.58
7.58
7.57
7.57
7.57
7.57
7.56
7.56
7.55
7.55
7.53
7.52
6.94
6.94
6.93
6.93
6.92
6.92
2.50 DMSO-d6
O
CF_3
HN
AR-09
0.6
1.0
1.0
3.0
3.1
1.1
2.0
f1 (ppm)

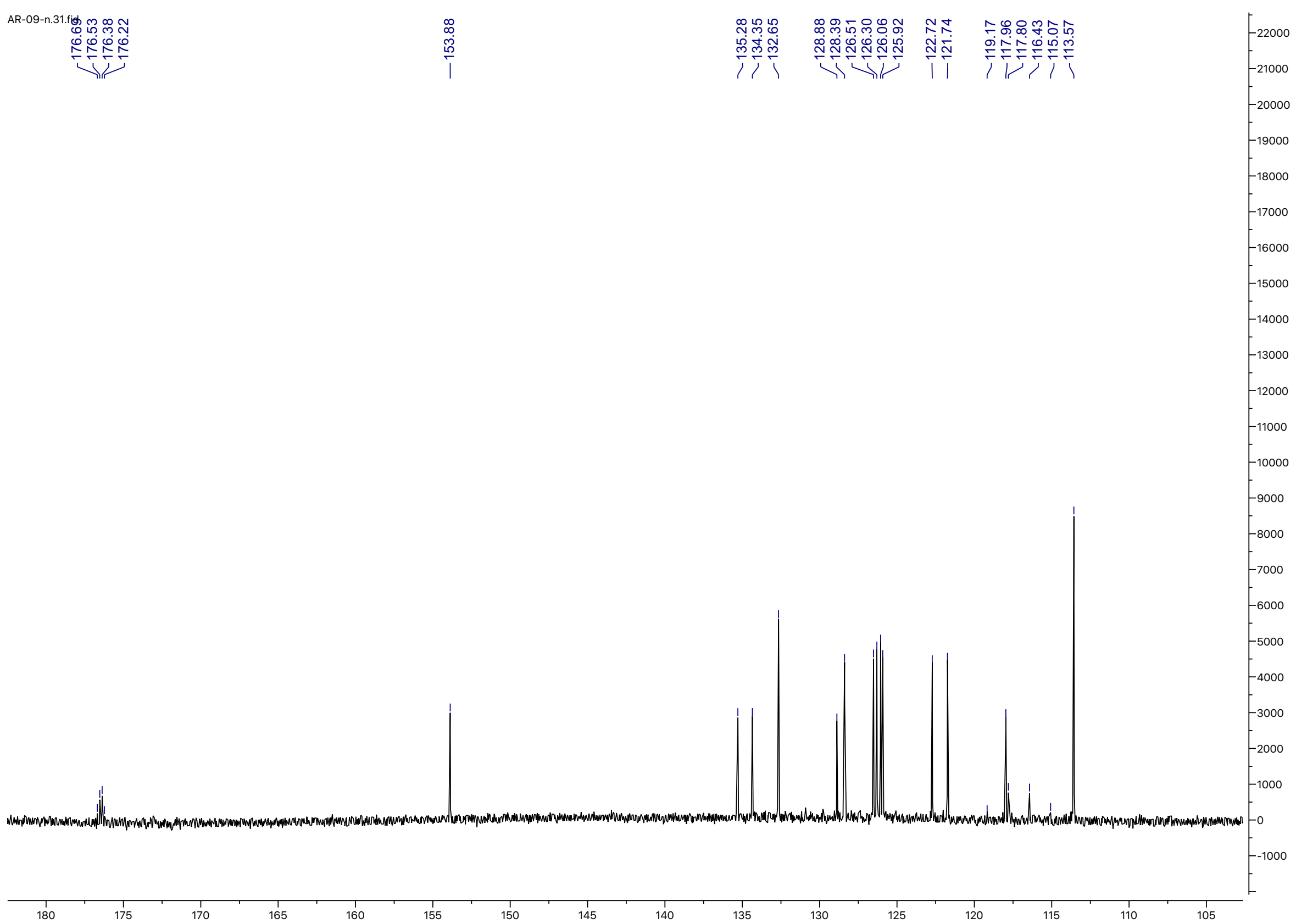
AR-09-n.31.fid
176.69
176.53
176.38
176.22
153.88
135.28
134.35
132.65
128.88
128.39
126.51
126.30
126.06
125.92
122.72
121.74
119.17
117.96
117.80
116.43
115.07
113.57
f1 (ppm)

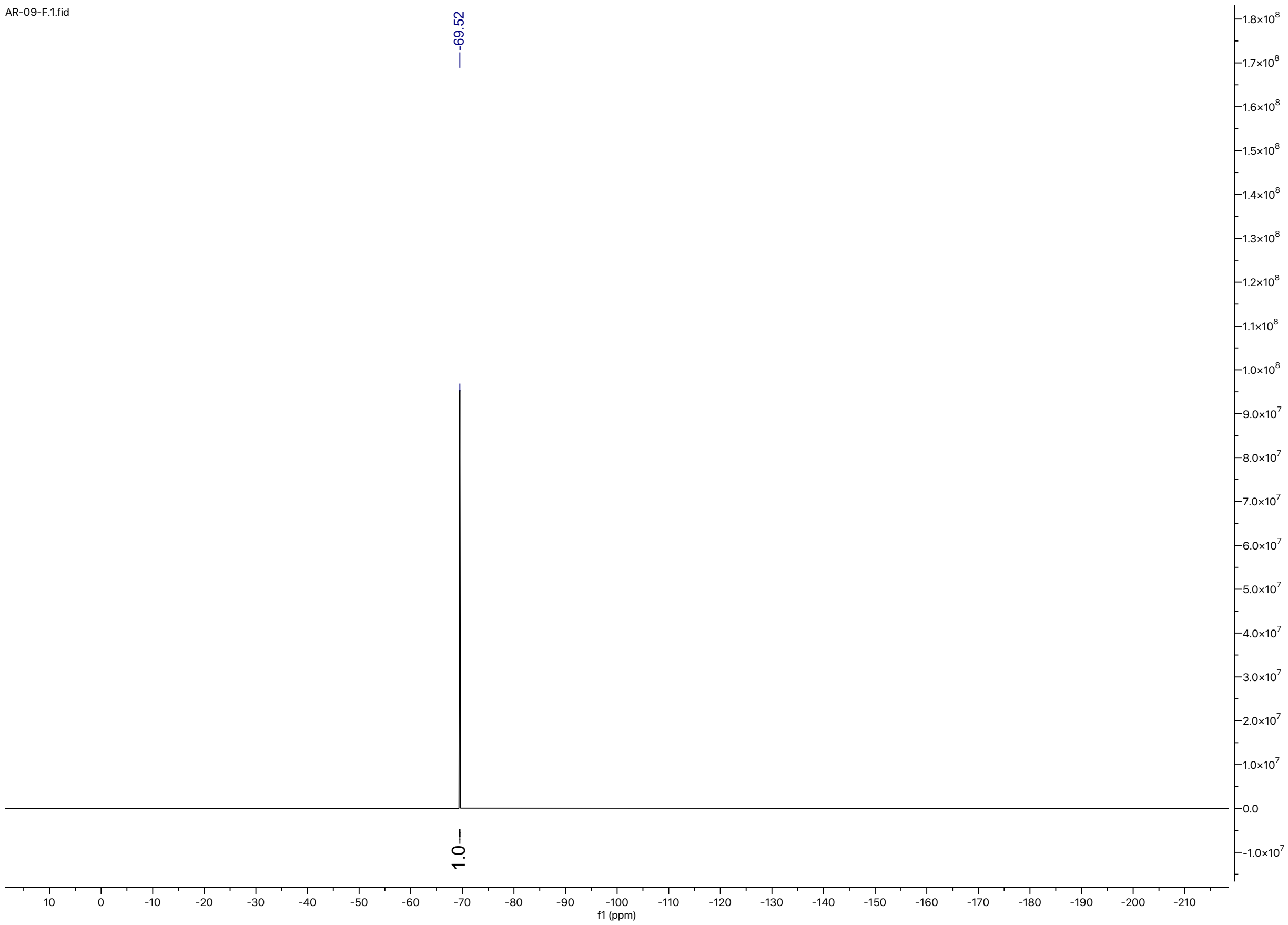
AR-09-F.1.fid
-69.52
1.0
f1 (ppm)

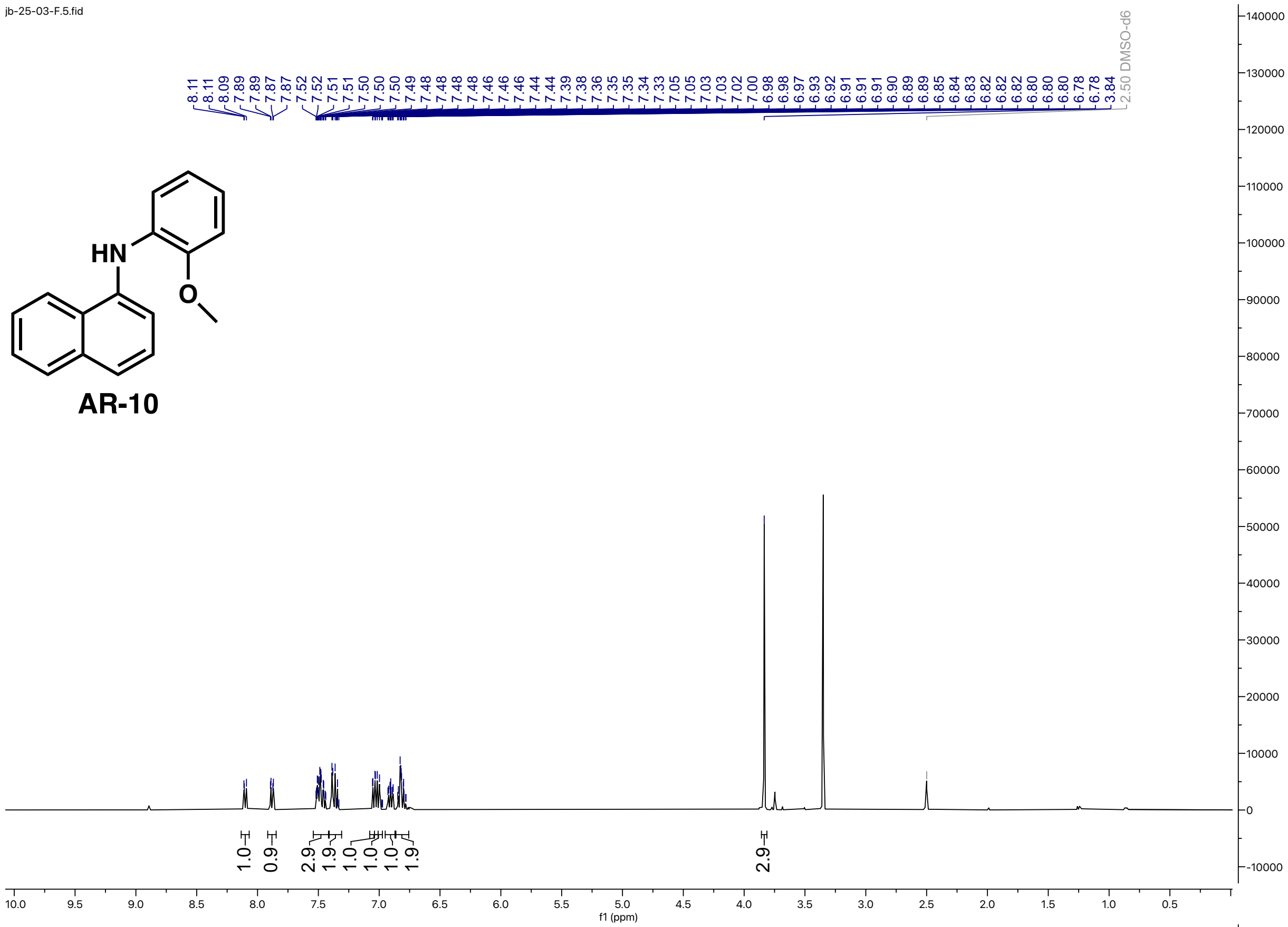

jb-25-03-F.5.fid
8.11 8.11 8.09 7.89 7.87 7.87 7.52 7.52 7.51 7.51 7.50 7.50 7.50 7.49 7.48 7.48 7.48 7.46 7.46 7.44 7.44 7.39 7.38 7.36 7.35 7.35 7.34 7.33 7.05 7.05 7.03 7.03 7.02 7.00 6.98 6.98 6.97 6.93 6.92 6.91 6.91 6.91 6.90 6.89 6.89 6.85 6.84 6.83 6.82 6.82 6.80 6.80 6.80 6.78 3.84
2.50 DMSO-d6
f1 (ppm)
HN
O
AR-10

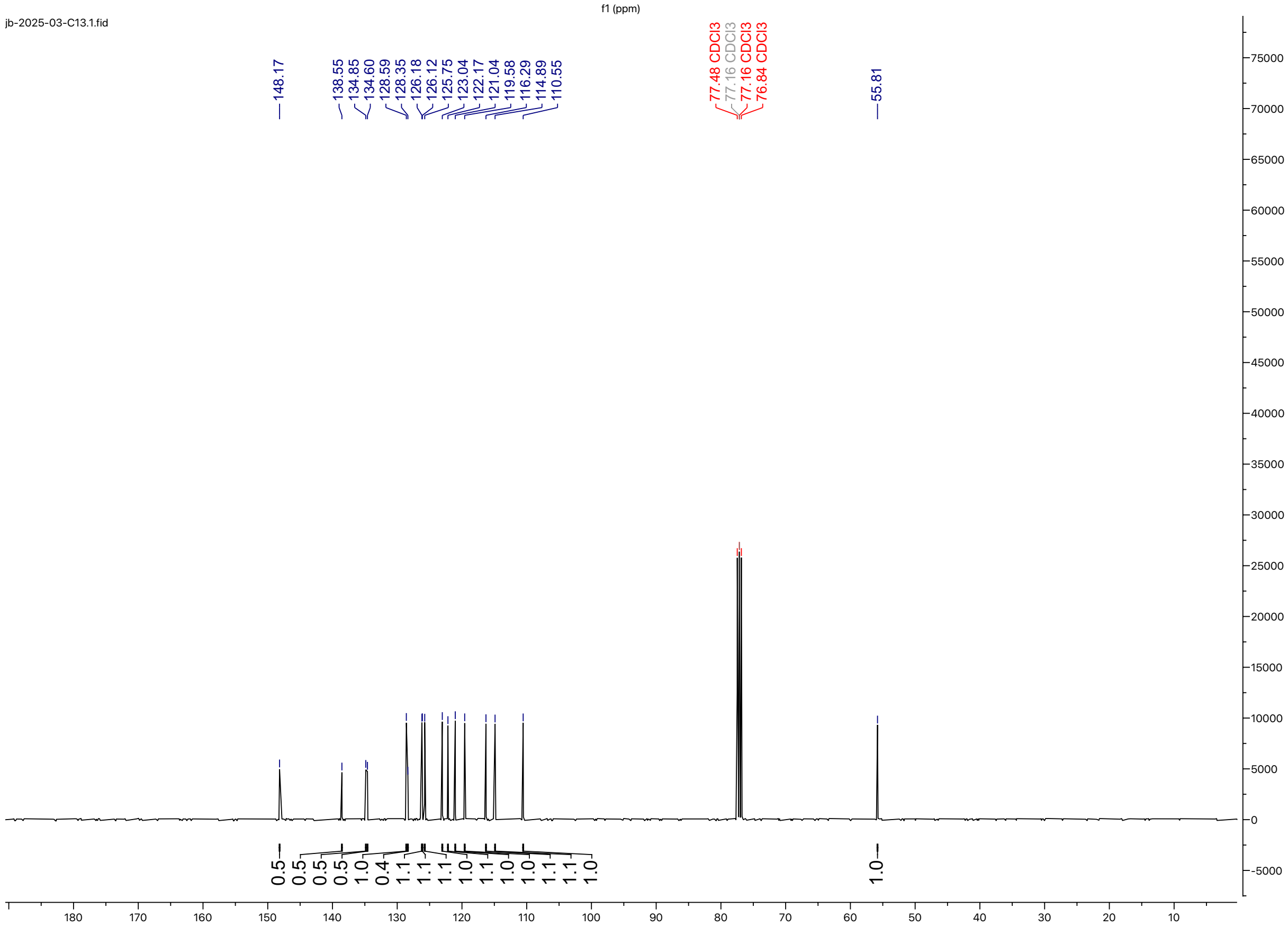

jb-2025-03-C13.1.fid
148.17 138.55 134.85 134.60 128.59 128.35 126.18 126.12 125.75 123.04 122.17 121.04 119.58 116.29 114.89 110.55
77.48 CDCl3 77.16 CDCl3 77.16 CDCl3 76.84 CDCl3
55.81
f1 (ppm)

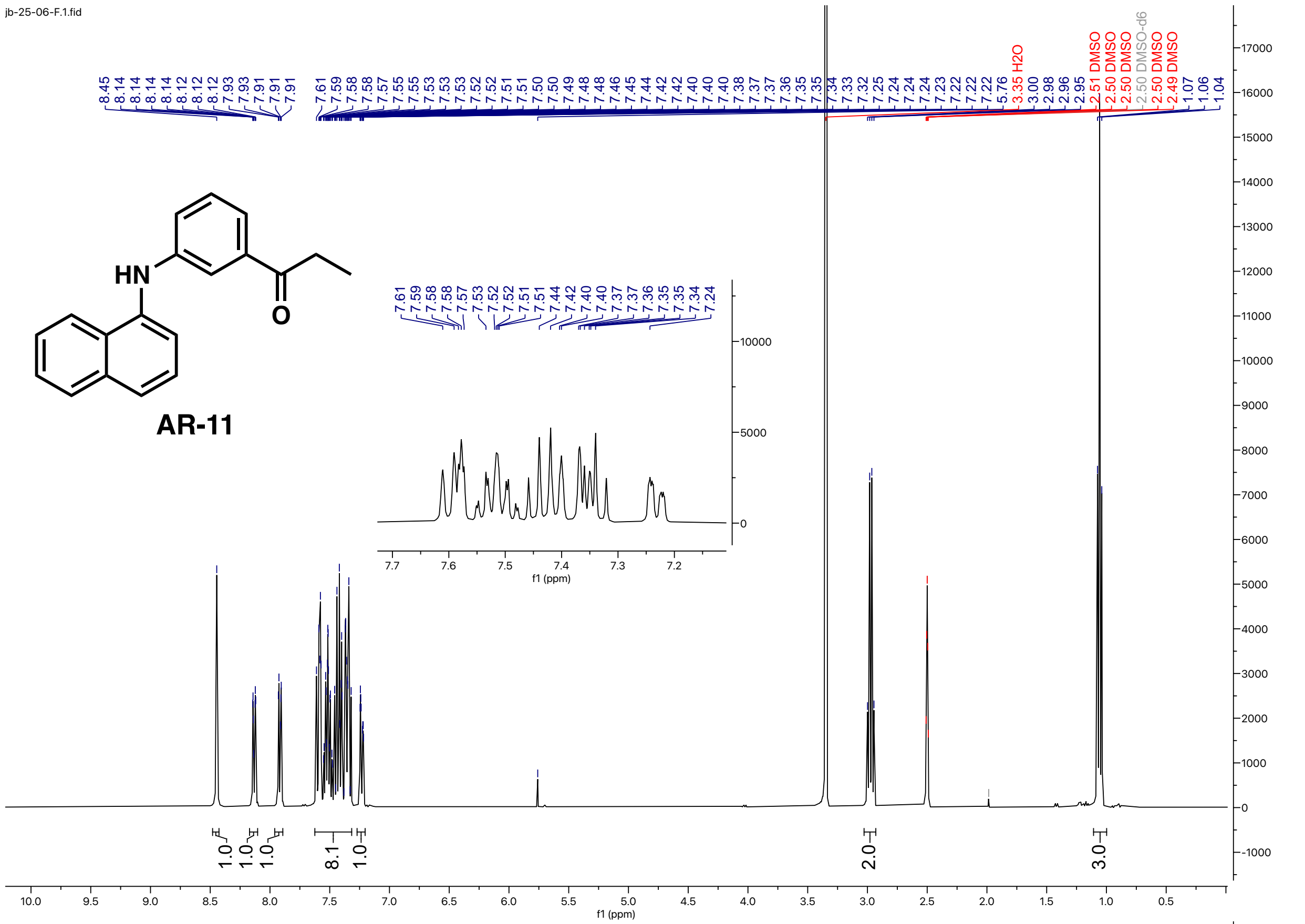
jb-25-06-F.1.fid
8.45 8.14 8.14 8.14 8.14 8.12 8.12 7.93 7.91 7.91 7.91 7.61 7.59 7.58 7.58 7.57 7.55 7.55 7.53 7.53 7.53 7.52 7.52 7.51 7.51 7.50 7.50 7.49 7.48 7.48 7.46 7.45 7.44 7.42 7.42 7.40 7.40 7.40 7.38 7.37 7.37 7.36 7.35 7.35 7.34 7.33 7.32 7.25 7.24 7.24 7.24 7.23 7.22 7.22 7.22 5.76
3.35 H2O
3.00 2.98 2.96 2.95
2.51 DMSO 2.50 DMSO 2.50 DMSO 2.50 DMSO-d6 2.50 DMSO 2.49 DMSO
1.07 1.06 1.04
HN
O
AR-11
7.61 7.59 7.58 7.58 7.57 7.53 7.52 7.52 7.51 7.51 7.44 7.42 7.40 7.40 7.37 7.37 7.36 7.35 7.35 7.34 7.24
f1 (ppm)
1.0 1.0 1.0 8.1 1.0 2.0 3.0
f1 (ppm)

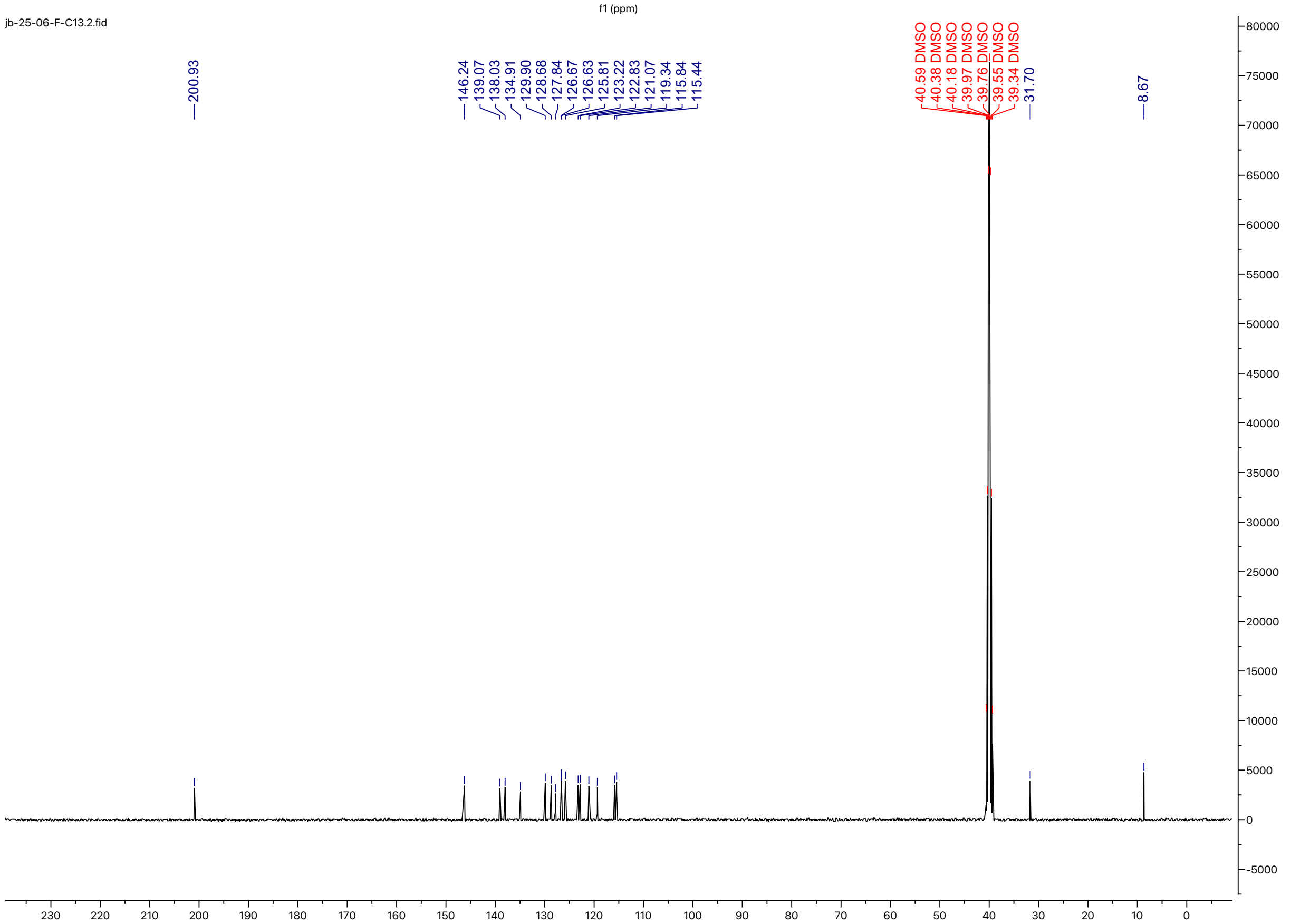
jb-25-06-F-C13.2.fid
200.93
146.24 139.07 138.03 134.91 129.90 128.68 127.84 126.67 126.63 125.81 123.22 122.83 121.07 119.34 115.84 115.44
40.59 DMSO 40.38 DMSO 40.18 DMSO 39.97 DMSO 39.76 DMSO 39.55 DMSO 39.34 DMSO
31.70
8.67
f1 (ppm)

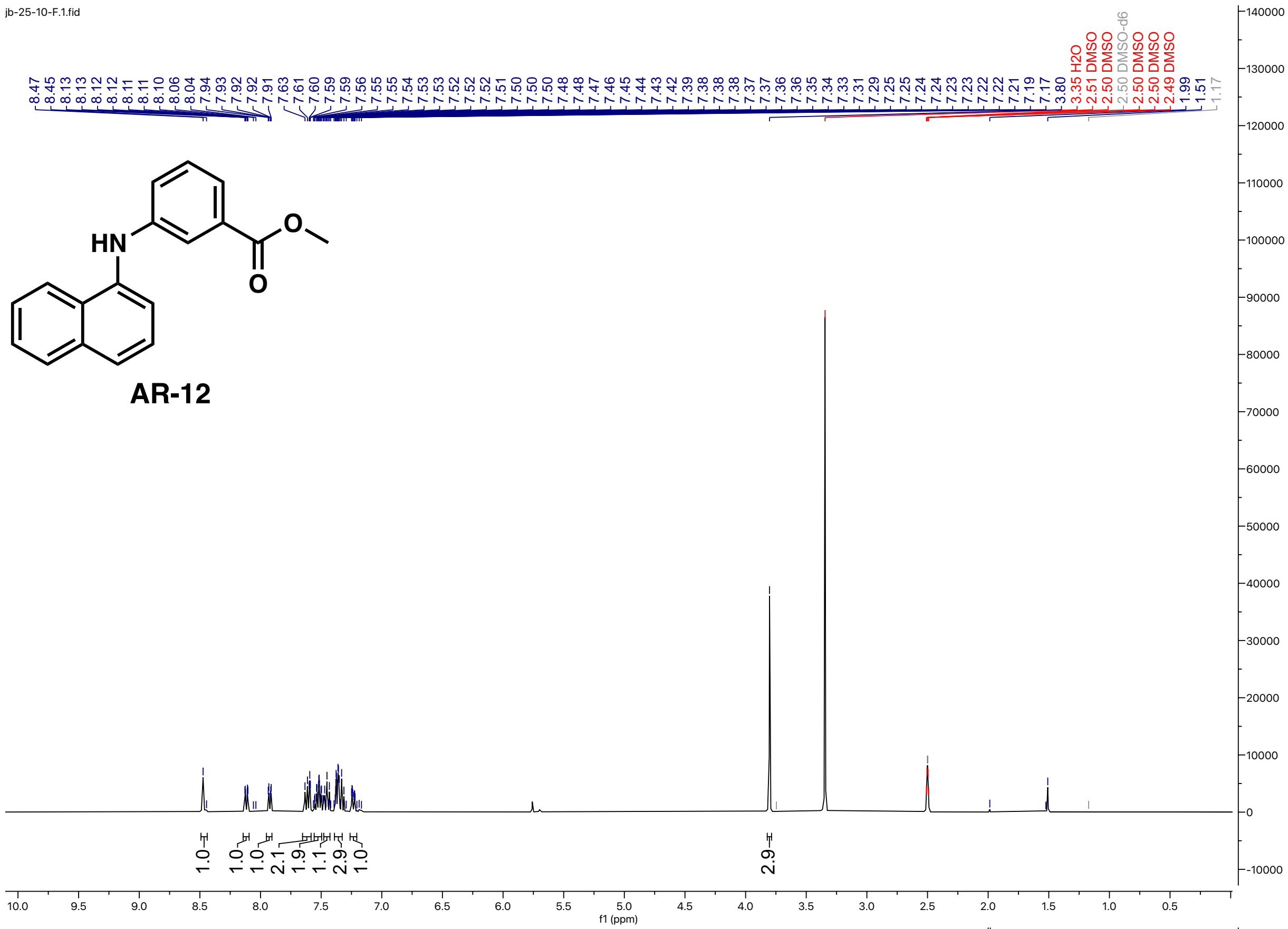

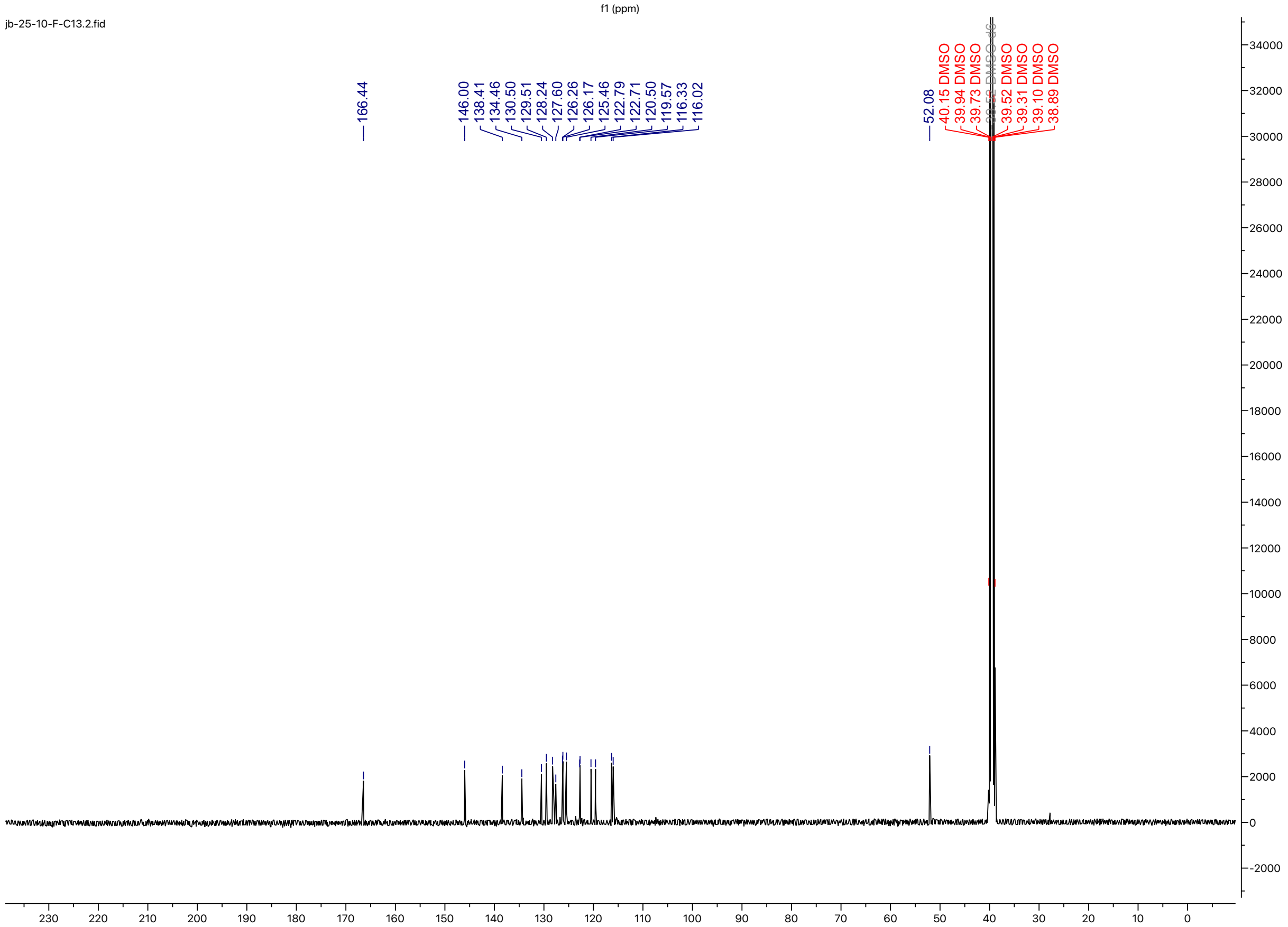

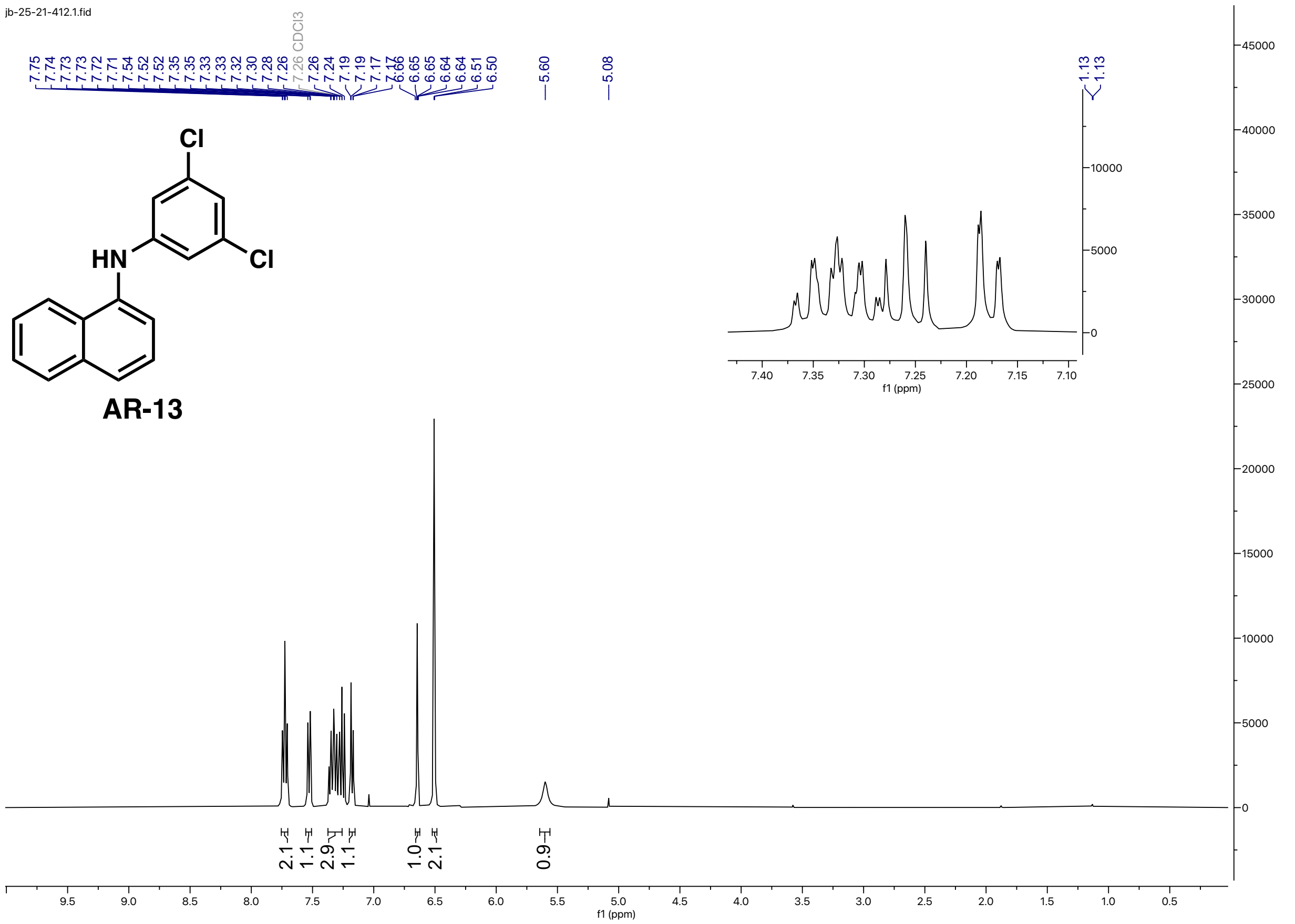
jb-25-21-412.1.fid
7.75
7.74
7.73
7.73
7.72
7.71
7.54
7.52
7.52
7.35
7.35
7.33
7.33
7.32
7.30
7.28
7.26
7.26 CDCl3
7.26
7.24
7.19
7.19
7.17
7.17
6.66
6.65
6.65
6.64
6.64
6.51
6.50
5.60
5.08
1.13
1.13
Cl
HN
Cl
AR-13
2.1
1.1
2.9
1.1
1.0
2.1
0.9
f1 (ppm)

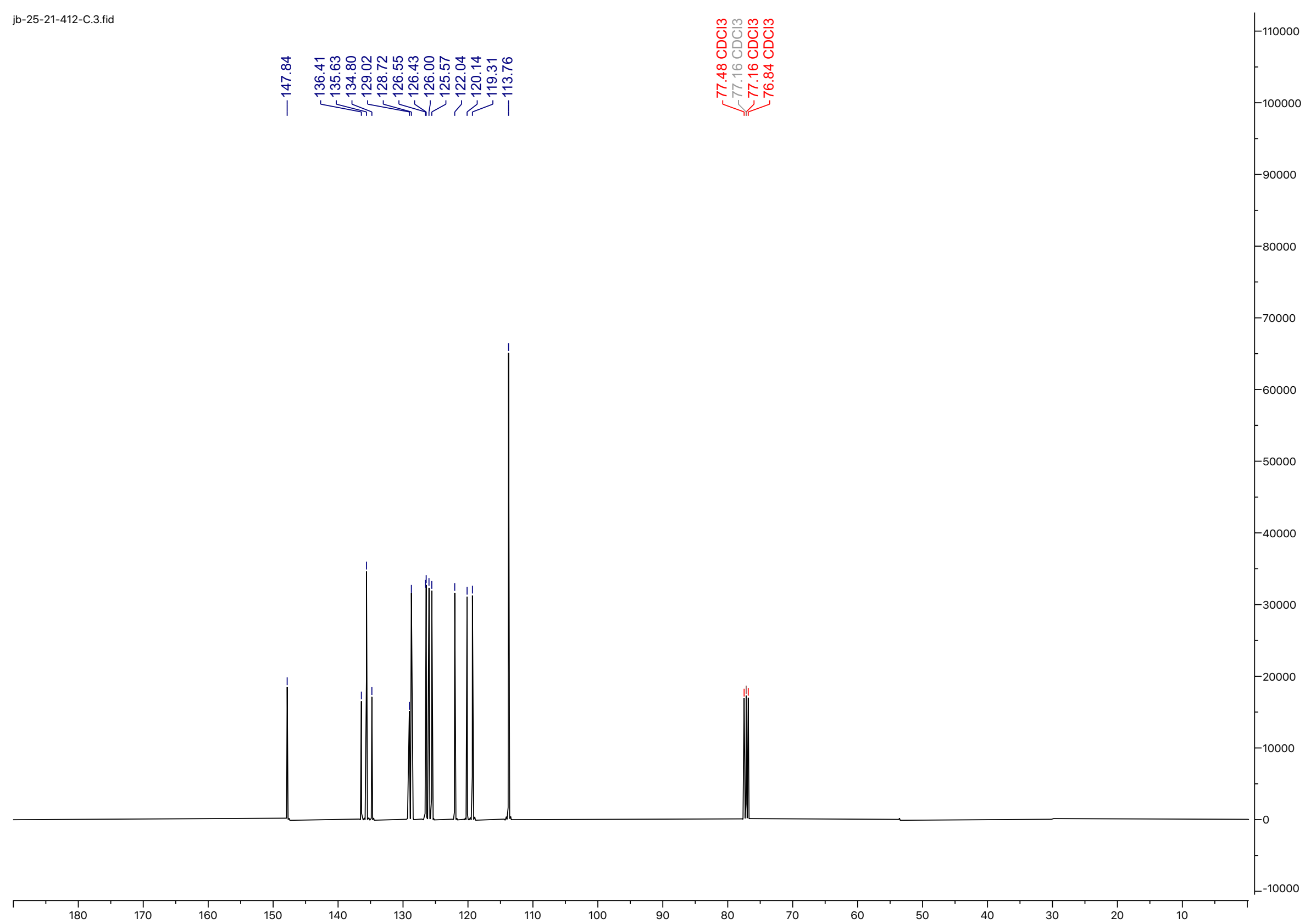
jb-25-21-412-C.3.fid
147.84
136.41
135.63
134.80
129.02
128.72
126.55
126.43
126.00
125.57
122.04
120.14
119.31
113.76
77.48 CDCl3
77.16 CDCl3
77.16 CDCl3
76.84 CDCl3
f1 (ppm)

AR-14

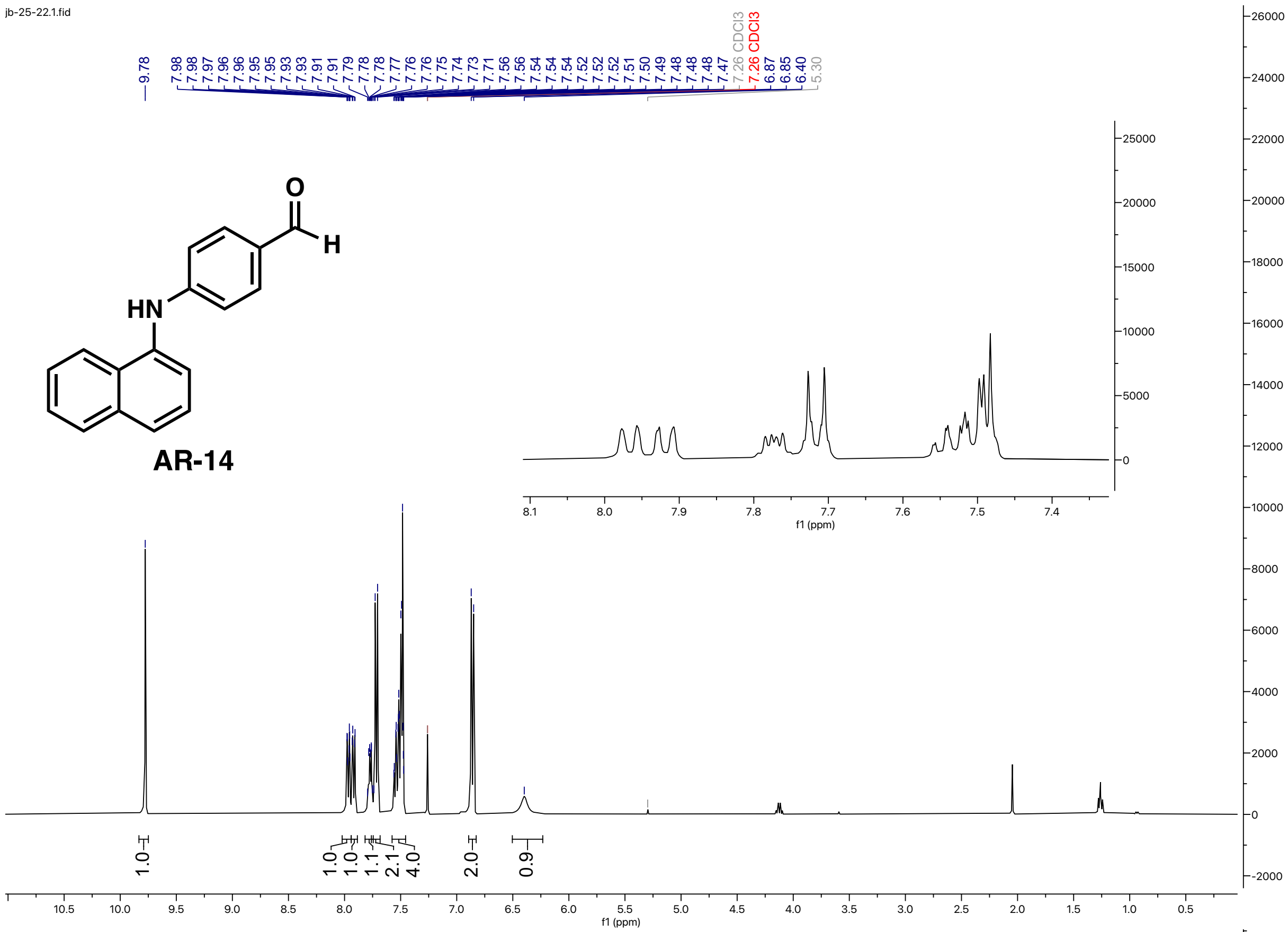
jb-25-22.1.fid
9.78
7.98 7.98 7.97 7.96 7.96 7.95 7.95 7.93 7.93 7.91 7.91 7.79 7.78 7.78 7.77 7.76 7.76 7.75 7.74 7.73 7.71 7.56 7.56 7.54 7.54 7.54 7.52 7.52 7.52 7.51 7.50 7.49 7.48 7.48 7.48 7.47
7.26 CDCl3
7.26 CDCl3
6.87 6.85 6.40
5.30
1.0
1.0 1.0 1.1 2.1 4.0
2.0
0.9
f1 (ppm)

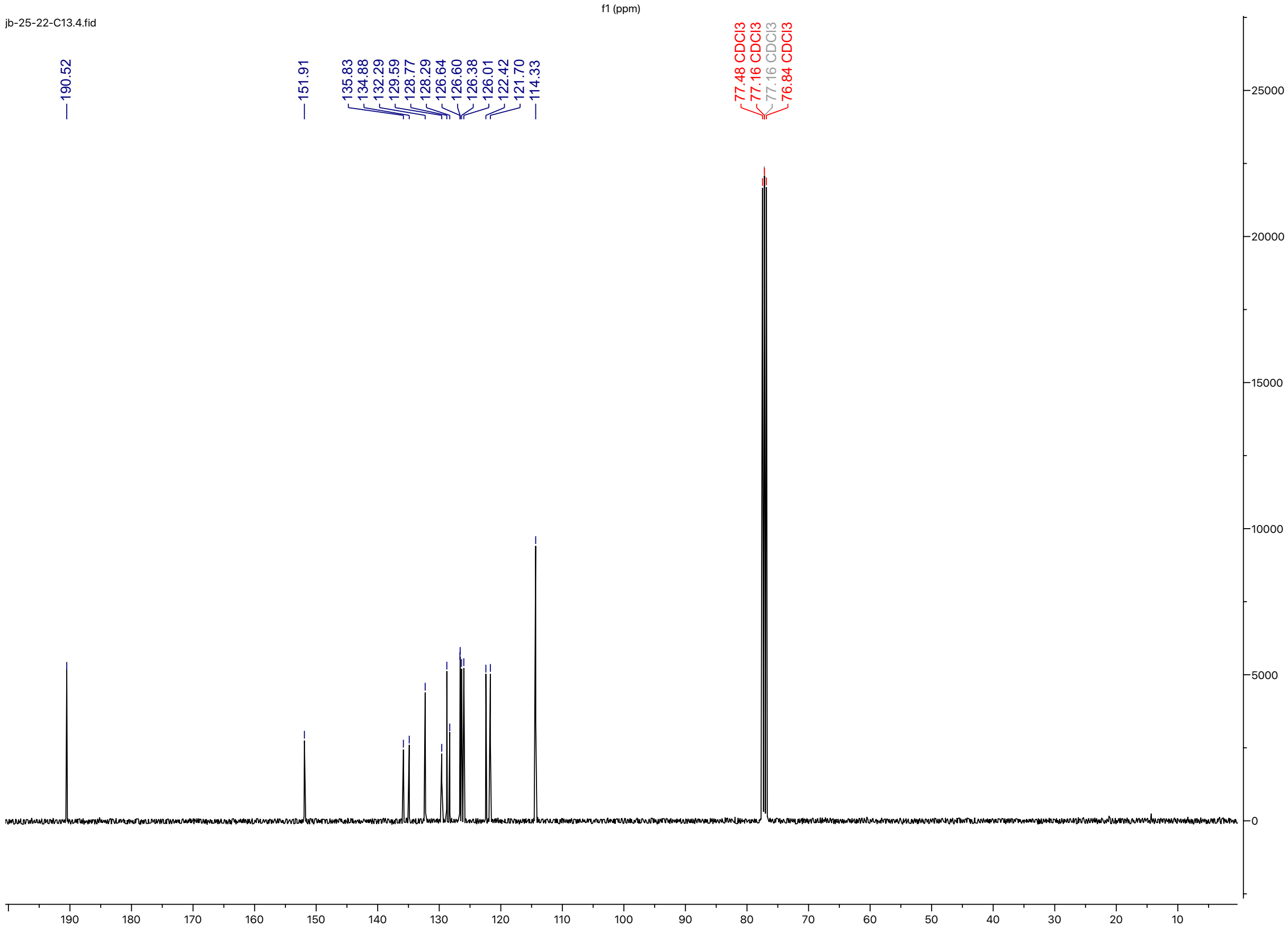
jb-25-22-C13.4.fid
190.52
151.91
135.83 134.88 132.29 129.59 128.77 128.29 126.64 126.60 126.38 126.01 122.42 121.70 114.33
77.48 CDCl3
77.16 CDCl3
77.16 CDCl3
76.84 CDCl3
f1 (ppm)

AR-15-n.28.fid

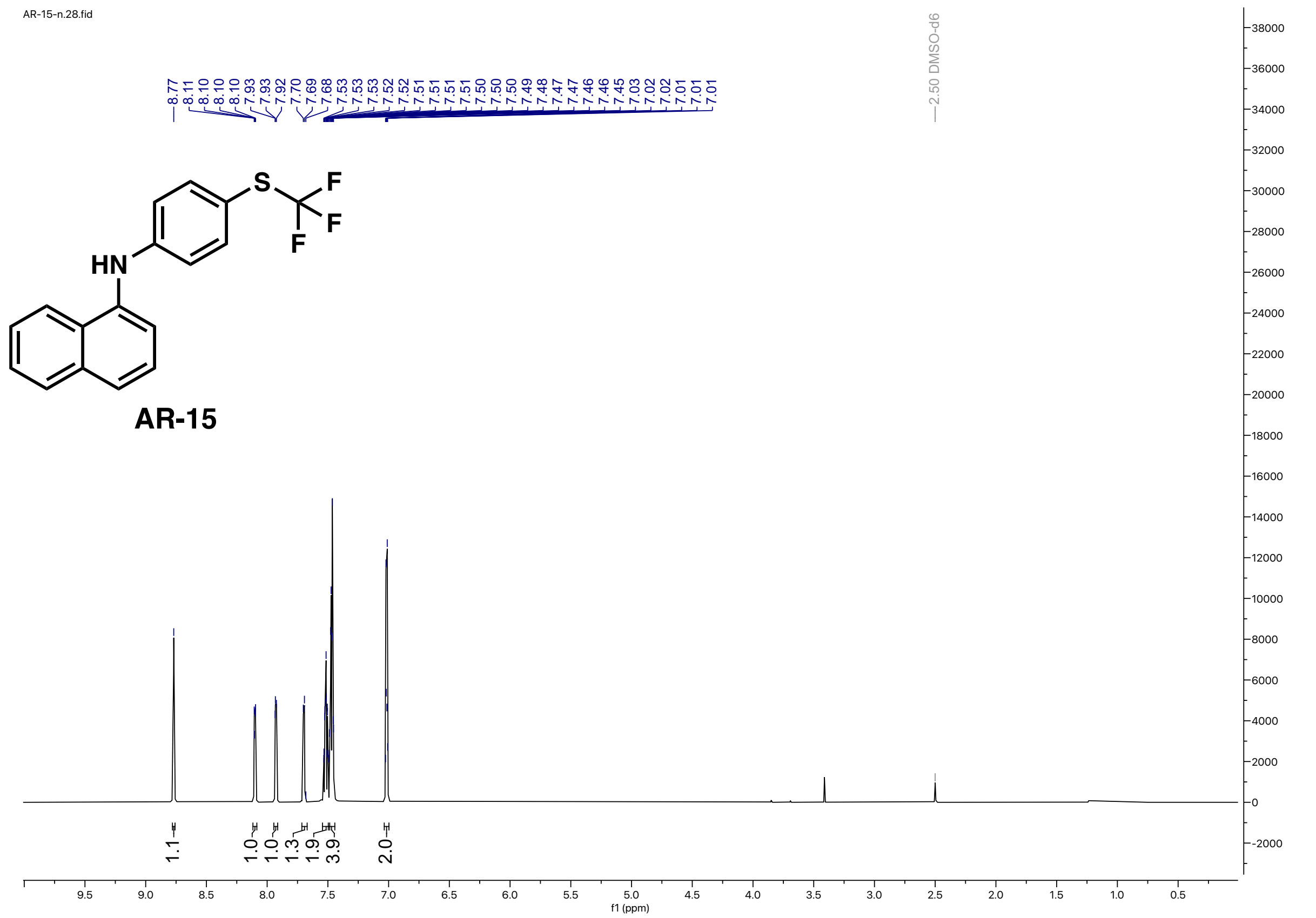

AR-15-n.29.fid

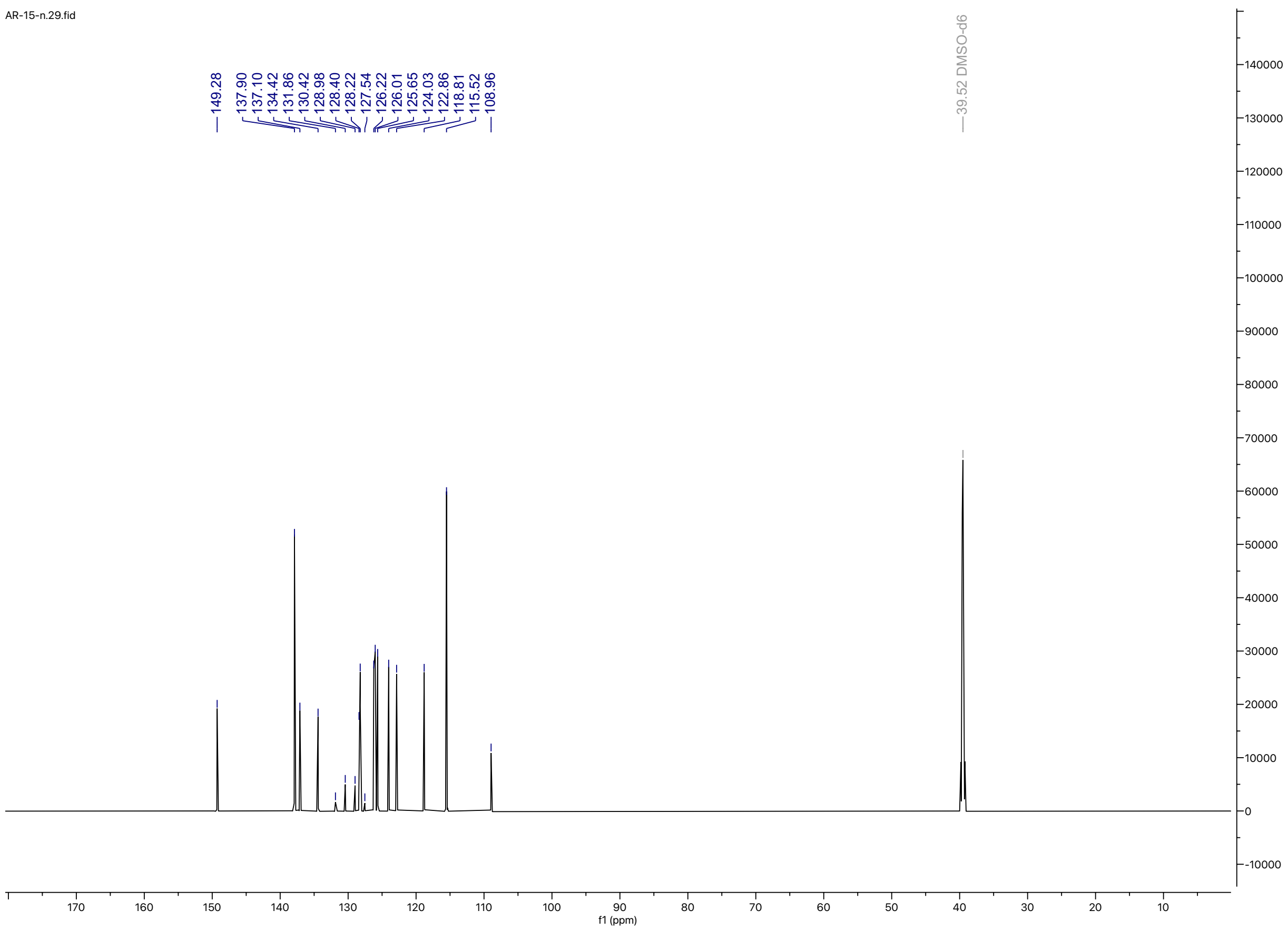

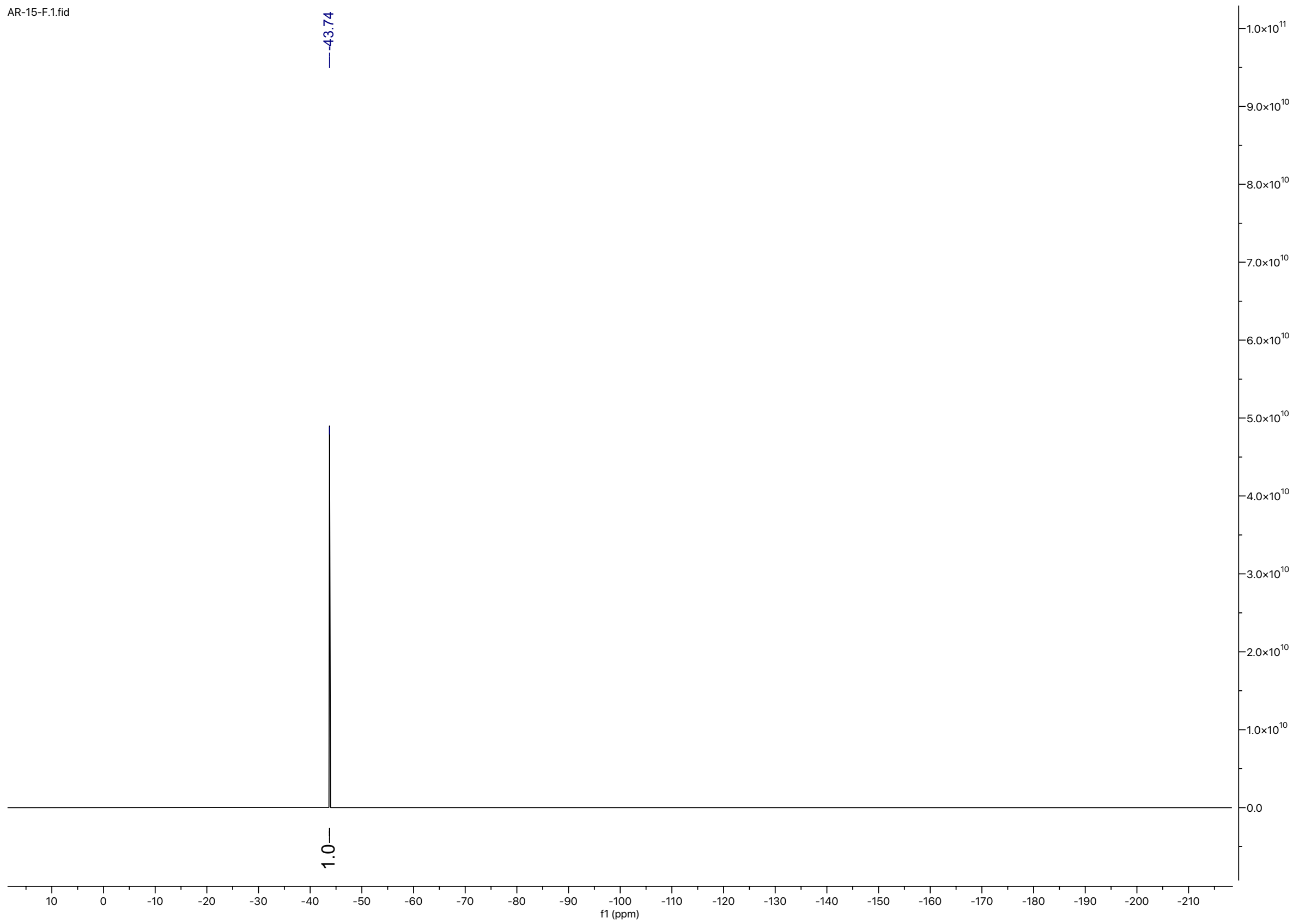
AR-15-F.1.fid
-43.74
1.0
f1 (ppm)

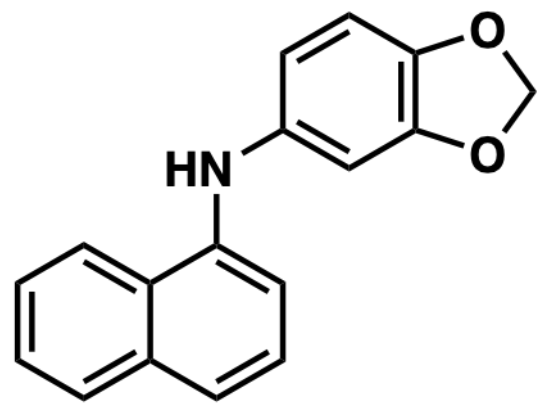

AR-16

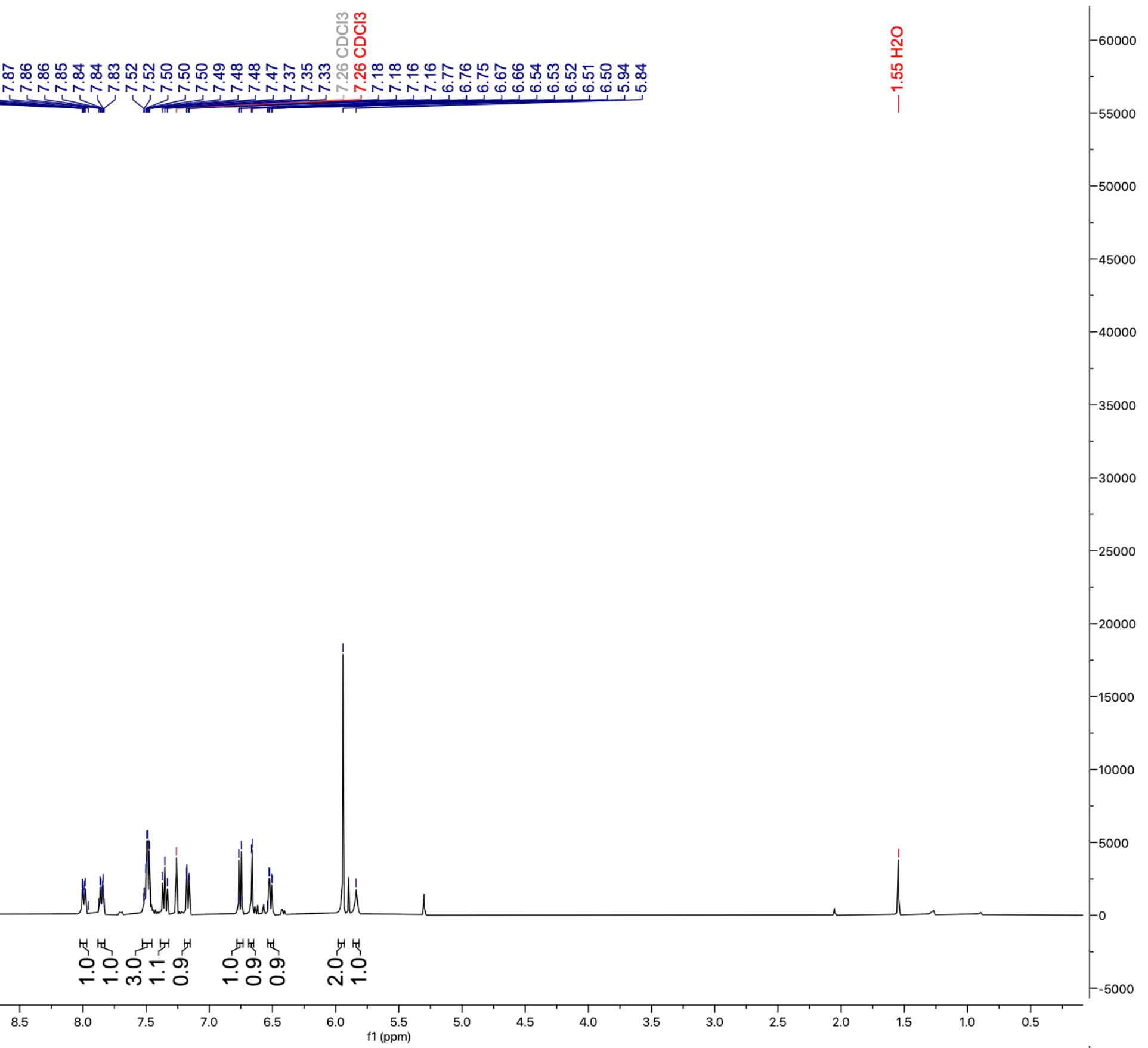
jb-2025-35.5.fid
8.00 8.00 8.00 8.00 7.99 7.98 7.96 7.87 7.86 7.86 7.85 7.84 7.84 7.83 7.52 7.52 7.50 7.50 7.50 7.49 7.48 7.48 7.47 7.37 7.35 7.33 7.26 CDCl3 7.26 CDCl3 7.18 7.18 7.16 7.16 6.77 6.76 6.75 6.67 6.66 6.66 6.54 6.53 6.52 6.51 6.50 5.94 5.84
1.55 H2O
1.0 1.0 3.0 1.1 0.9 1.0 0.9 0.9 2.0 1.0
f1 (ppm)

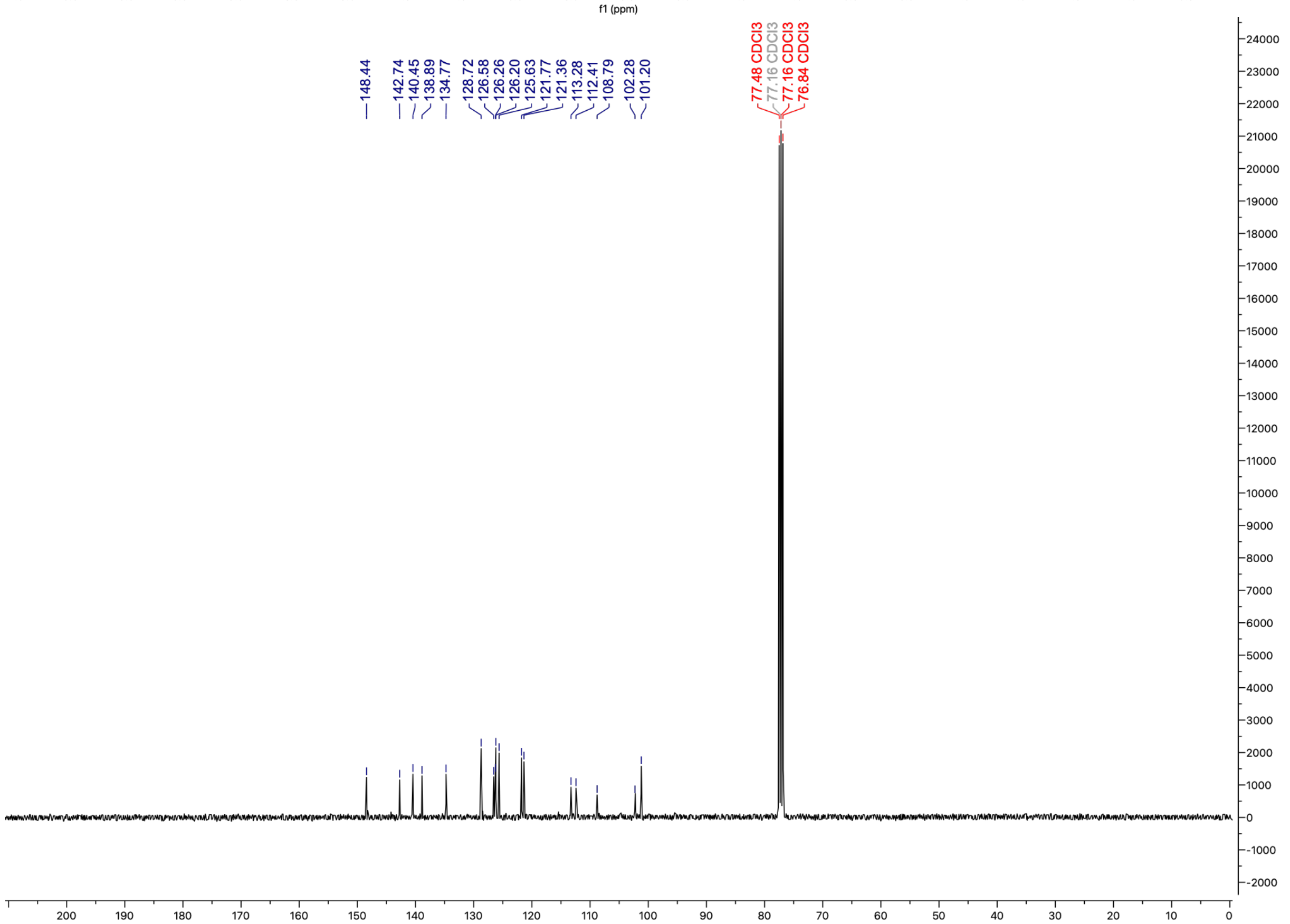
148.44 142.74 140.45 138.89 134.77 128.72 126.58 126.26 126.20 125.63 121.77 121.36 113.28 112.41 108.79 102.28 101.20
77.48 CDCl3 77.16 CDCl3 77.16 CDCl3 76.84 CDCl3
f1 (ppm)

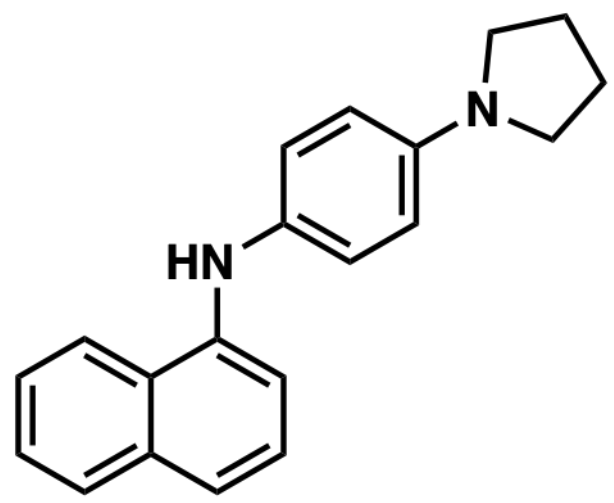
HN
N

AR-17

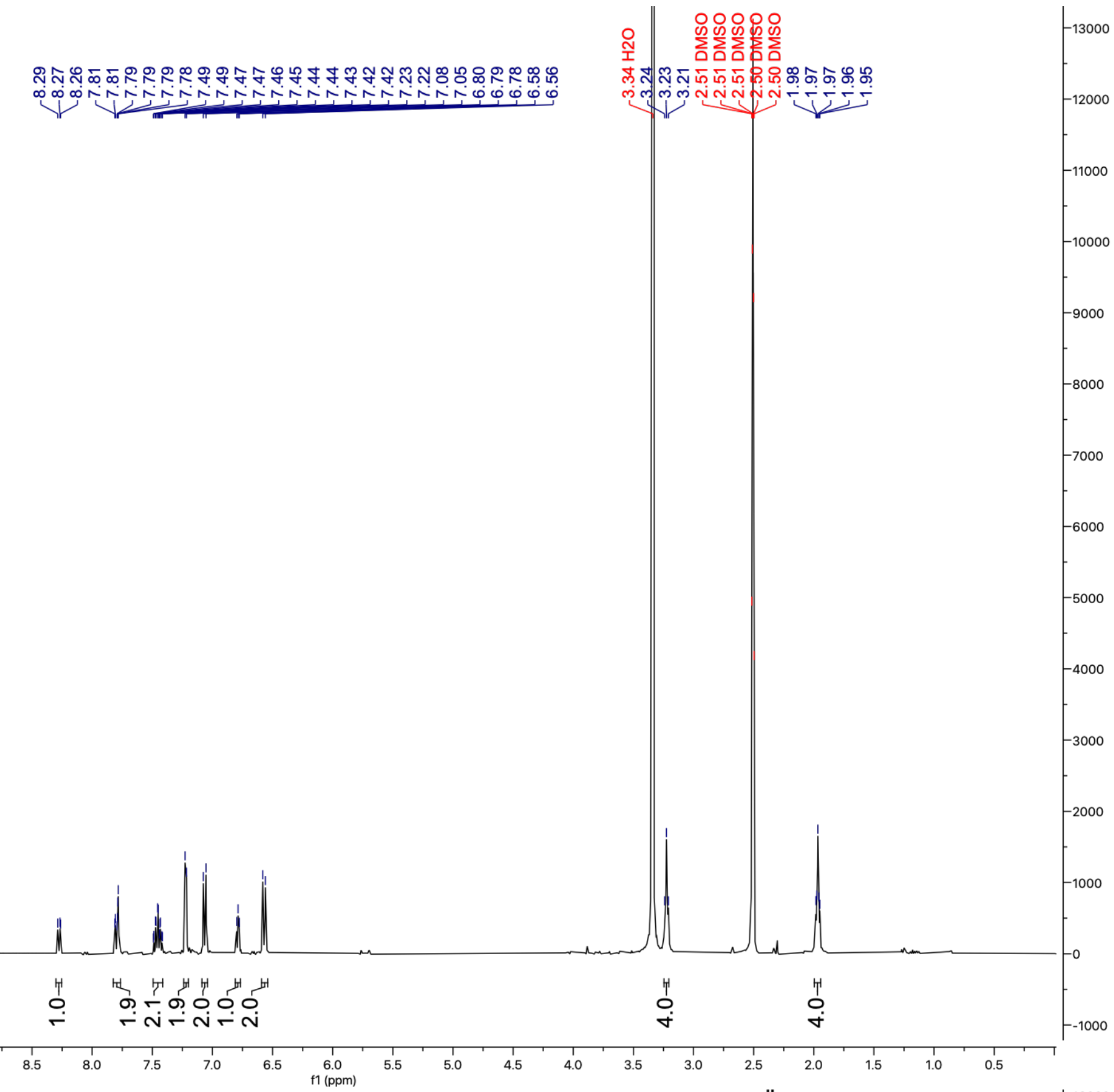
jb-2025-17-crude.1.fid
8.29
8.27
8.26
7.81
7.79
7.79
7.79
7.78
7.49
7.47
7.47
7.46
7.45
7.44
7.44
7.43
7.42
7.42
7.23
7.22
7.08
7.05
6.80
6.79
6.78
6.58
6.56
3.34 H2O
3.24
3.23
3.21
2.51 DMSO
2.51 DMSO
2.51 DMSO
2.50 DMSO
2.50 DMSO
1.98
1.97
1.97
1.96
1.95
1.0
1.9
2.1
1.9
2.0
1.0
2.0
4.0
4.0
f1 (ppm)

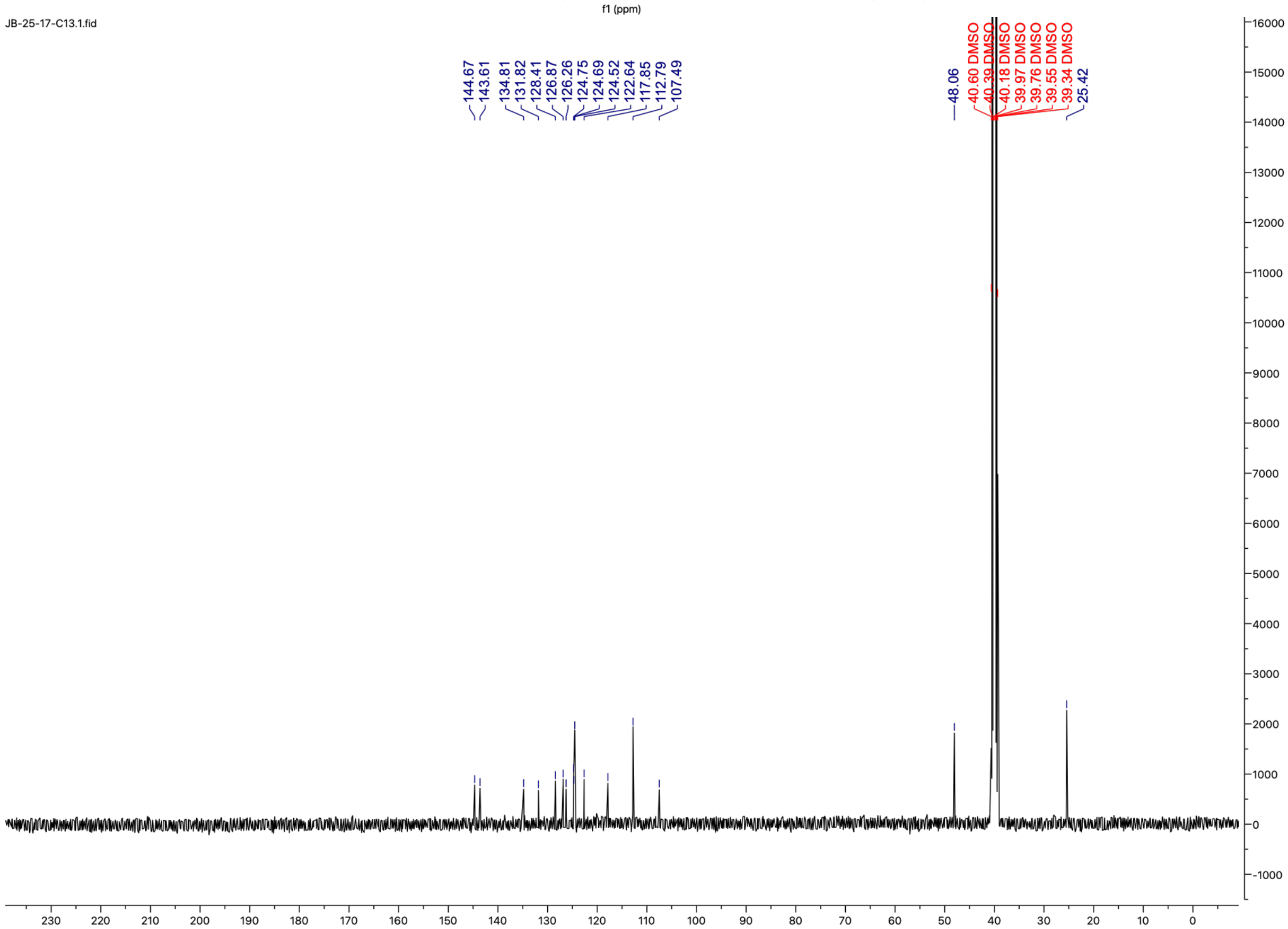
JB-25-17-C13.1.fid
144.67
143.61
134.81
131.82
128.41
126.87
126.26
124.75
124.69
124.52
122.64
117.85
112.79
107.49
48.06
40.60 DMSO
40.39 DMSO
40.18 DMSO
39.97 DMSO
39.76 DMSO
39.55 DMSO
39.34 DMSO
25.42
f1 (ppm)

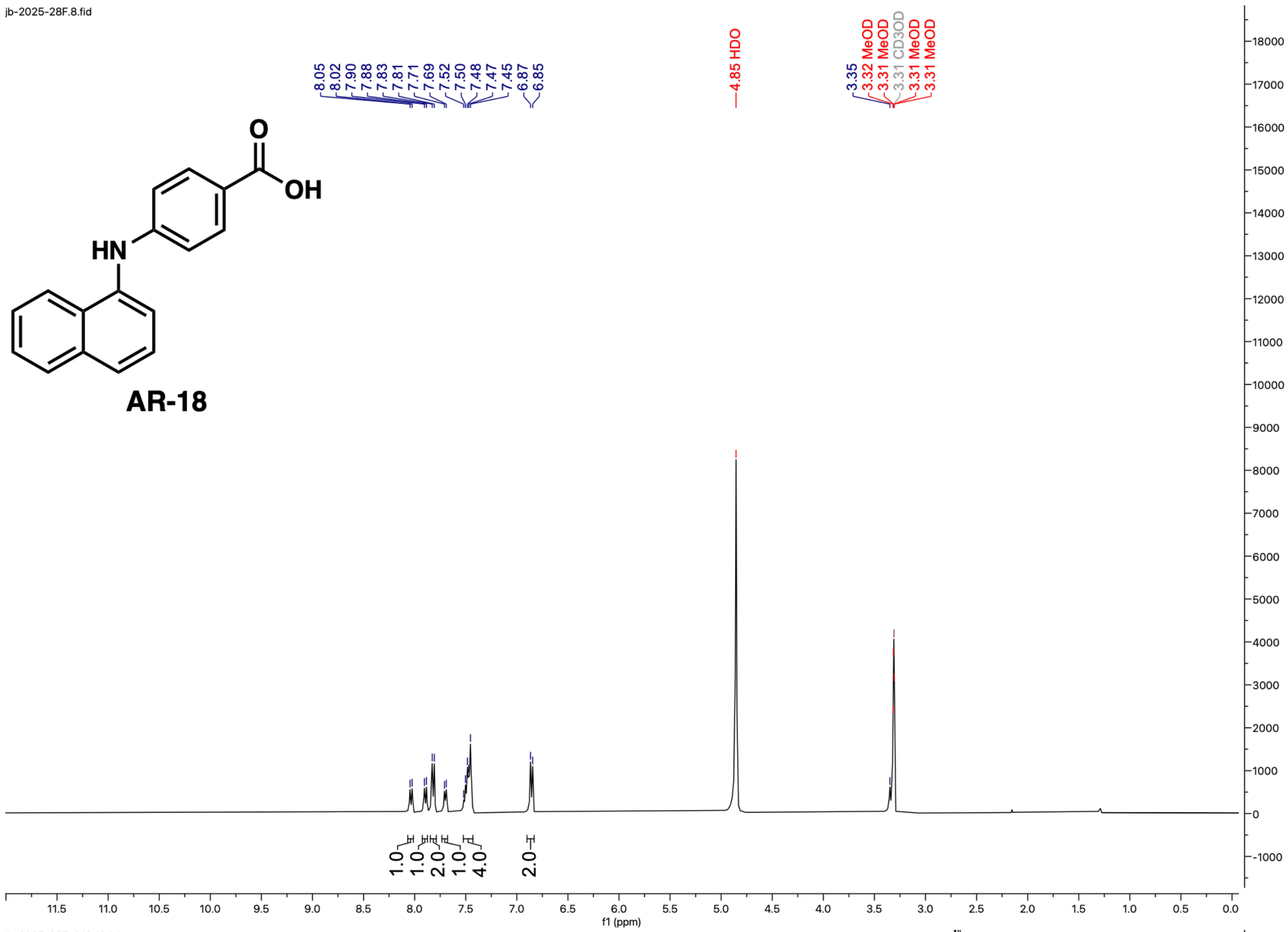
jb-2025-28F.8.fid
8.05
8.02
7.90
7.88
7.83
7.81
7.71
7.69
7.52
7.50
7.48
7.47
7.45
6.87
6.85
4.85 HDO
3.35
3.32 MeOD
3.31 CD3OD
3.31 MeOD
3.31 MeOD
O
OH
HN
AR-18
1.0
1.0
2.0
1.0
4.0
2.0
f1 (ppm)

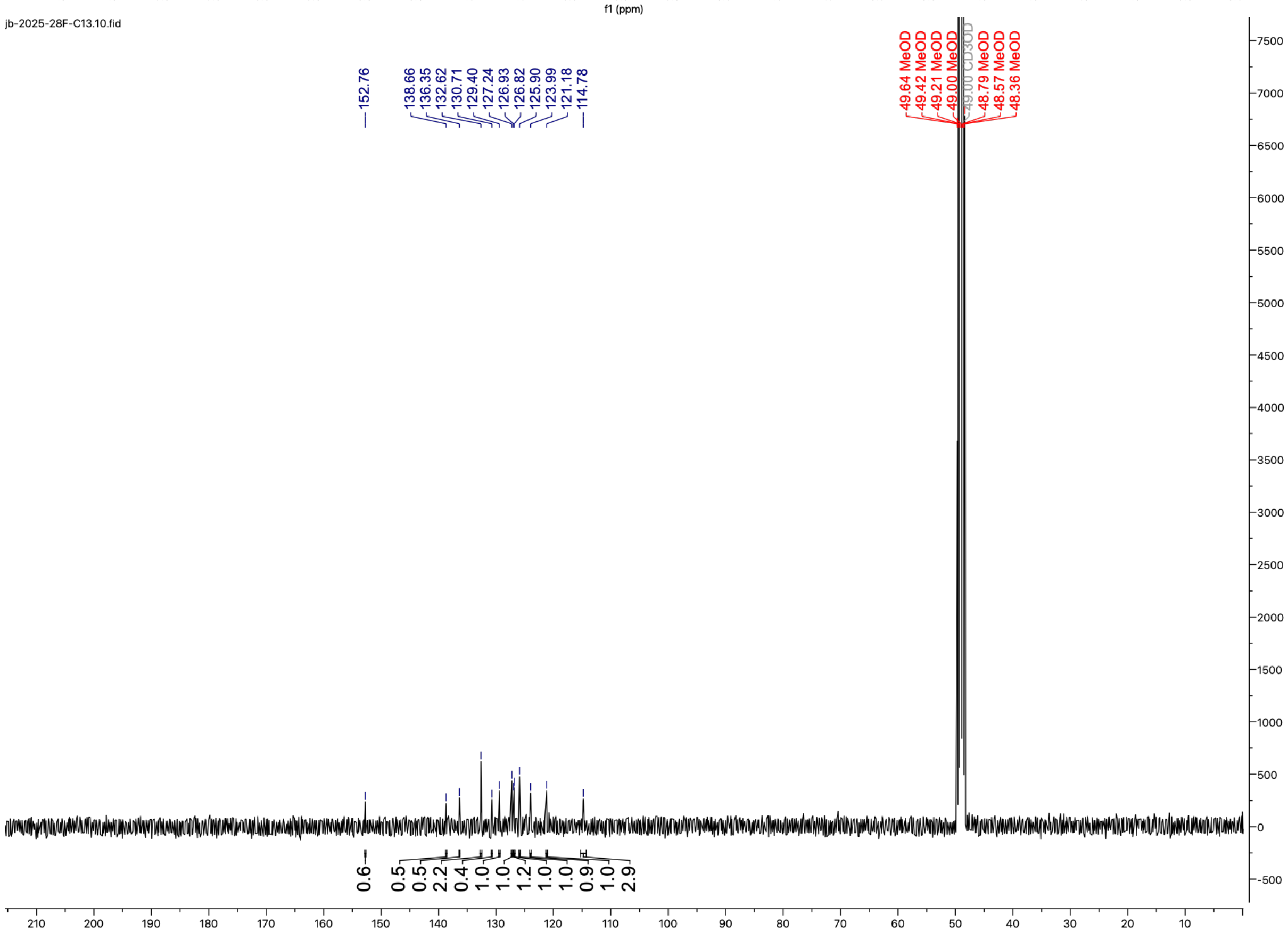
jb-2025-28F-C13.10.fid
152.76
138.66
136.35
132.62
130.71
129.40
127.24
126.93
126.82
125.90
123.99
121.18
114.78
49.64 MeOD
49.42 MeOD
49.21 MeOD
49.00 CD3OD
48.79 MeOD
48.57 MeOD
48.36 MeOD
0.6
0.5
0.5
2.2
0.4
1.0
1.0
1.2
1.0
1.0
0.9
1.0
2.9
f1 (ppm)

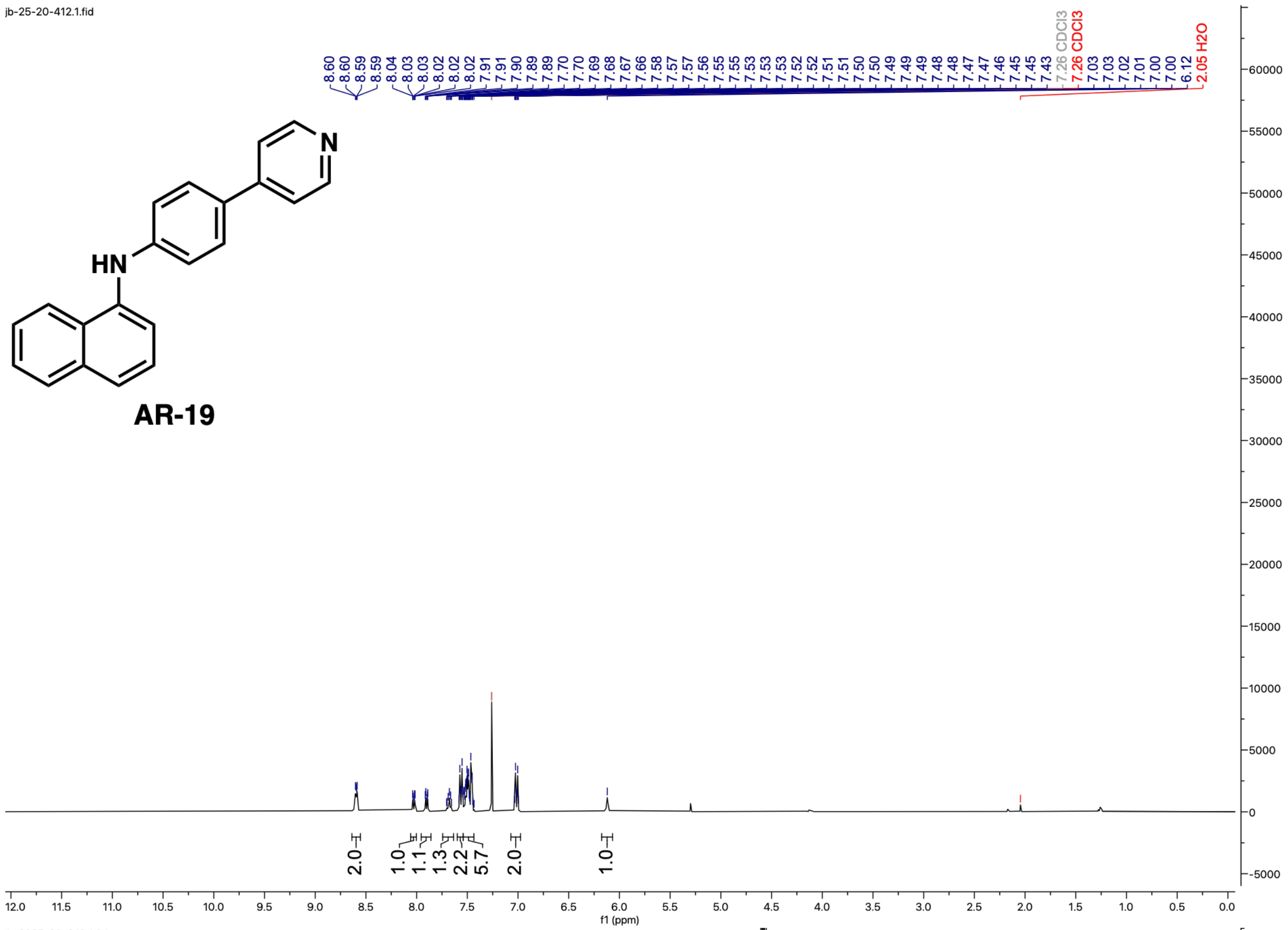
N
HN
AR-19
jb-25-20-412.1.fid
8.60
8.60
8.59
8.59
8.04
8.03
8.03
8.02
8.02
8.02
7.91
7.90
7.89
7.89
7.70
7.69
7.68
7.67
7.66
7.58
7.57
7.56
7.55
7.55
7.53
7.53
7.53
7.52
7.51
7.51
7.50
7.49
7.49
7.49
7.48
7.48
7.47
7.46
7.45
7.45
7.43
7.26 CDCl3
7.26 CDCl3
7.03
7.03
7.02
7.01
7.00
6.12
2.05 H2O
2.0
1.0
1.1
1.3
2.2
5.7
2.0
1.0
f1 (ppm)

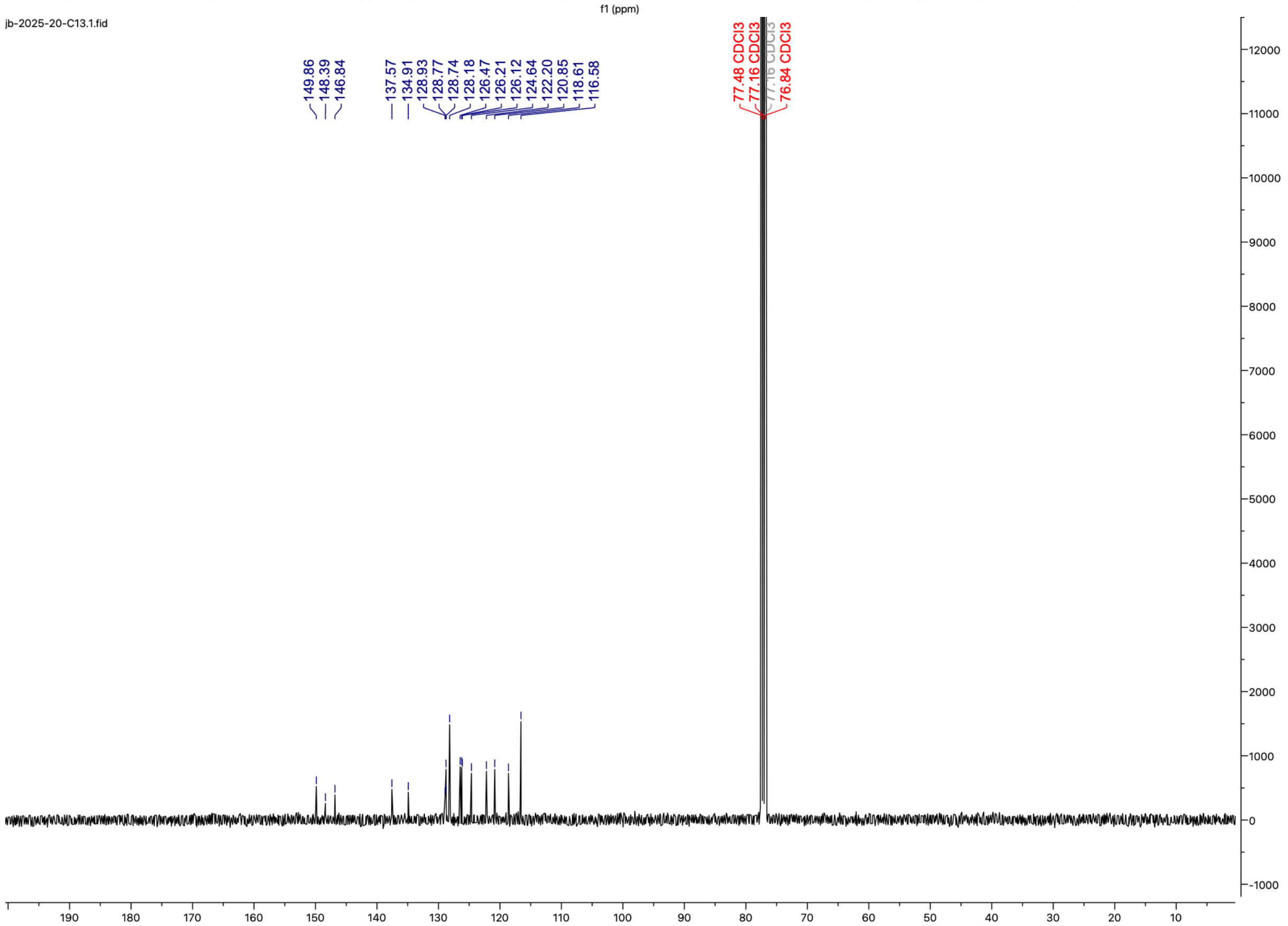
jb-2025-20-C13.1.fid
149.86
148.39
146.84
137.57
134.91
128.93
128.77
128.74
128.18
126.47
126.21
126.12
124.64
122.20
120.85
118.61
116.58
77.48 CDCl3
77.16 CDCl3
77.16 CDCl3
76.84 CDCl3
f1 (ppm)

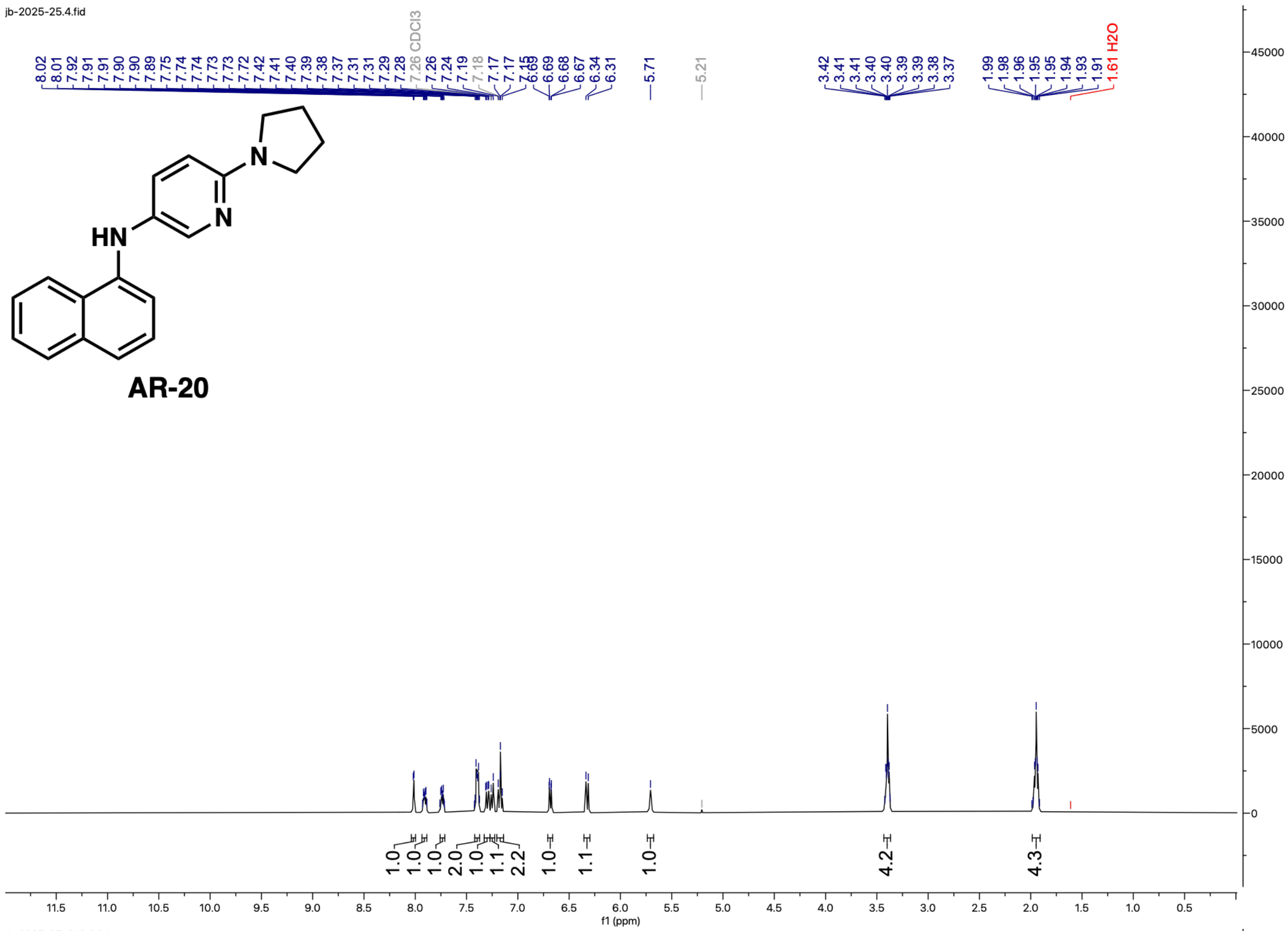
jb-2025-25.4.fid
8.02
8.01
7.92
7.91
7.91
7.90
7.90
7.89
7.75
7.74
7.74
7.73
7.73
7.72
7.42
7.41
7.40
7.39
7.38
7.37
7.31
7.31
7.29
7.28
7.26 CDCl3
7.26
7.24
7.19
7.18
7.17
7.17
7.15
6.69
6.69
6.68
6.67
6.34
6.31
5.71
5.21
3.42
3.41
3.41
3.40
3.40
3.39
3.39
3.38
3.37
1.99
1.98
1.96
1.95
1.95
1.94
1.93
1.91
1.61 H2O
HN
N
N
AR-20
1.0
1.0
1.0
2.0
1.0
1.1
2.2
1.0
1.1
1.0
4.2
4.3
f1 (ppm)

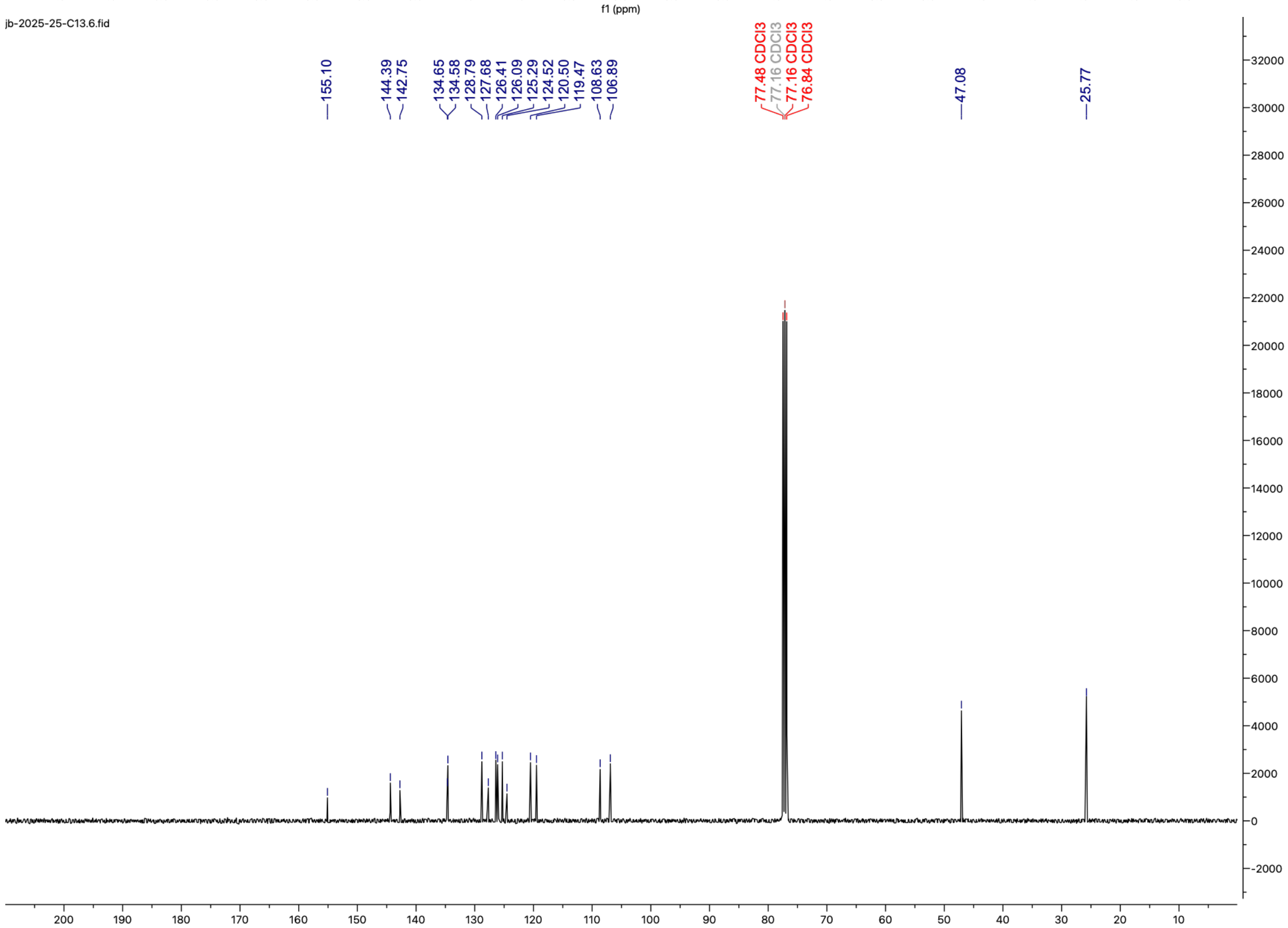
jb-2025-25-C13.6.fid
155.10
144.39
142.75
134.65
134.58
128.79
127.68
126.41
126.09
125.29
124.52
120.50
119.47
108.63
106.89
77.48 CDCl3
77.16 CDCl3
77.16 CDCl3
76.84 CDCl3
47.08
25.77
f1 (ppm)

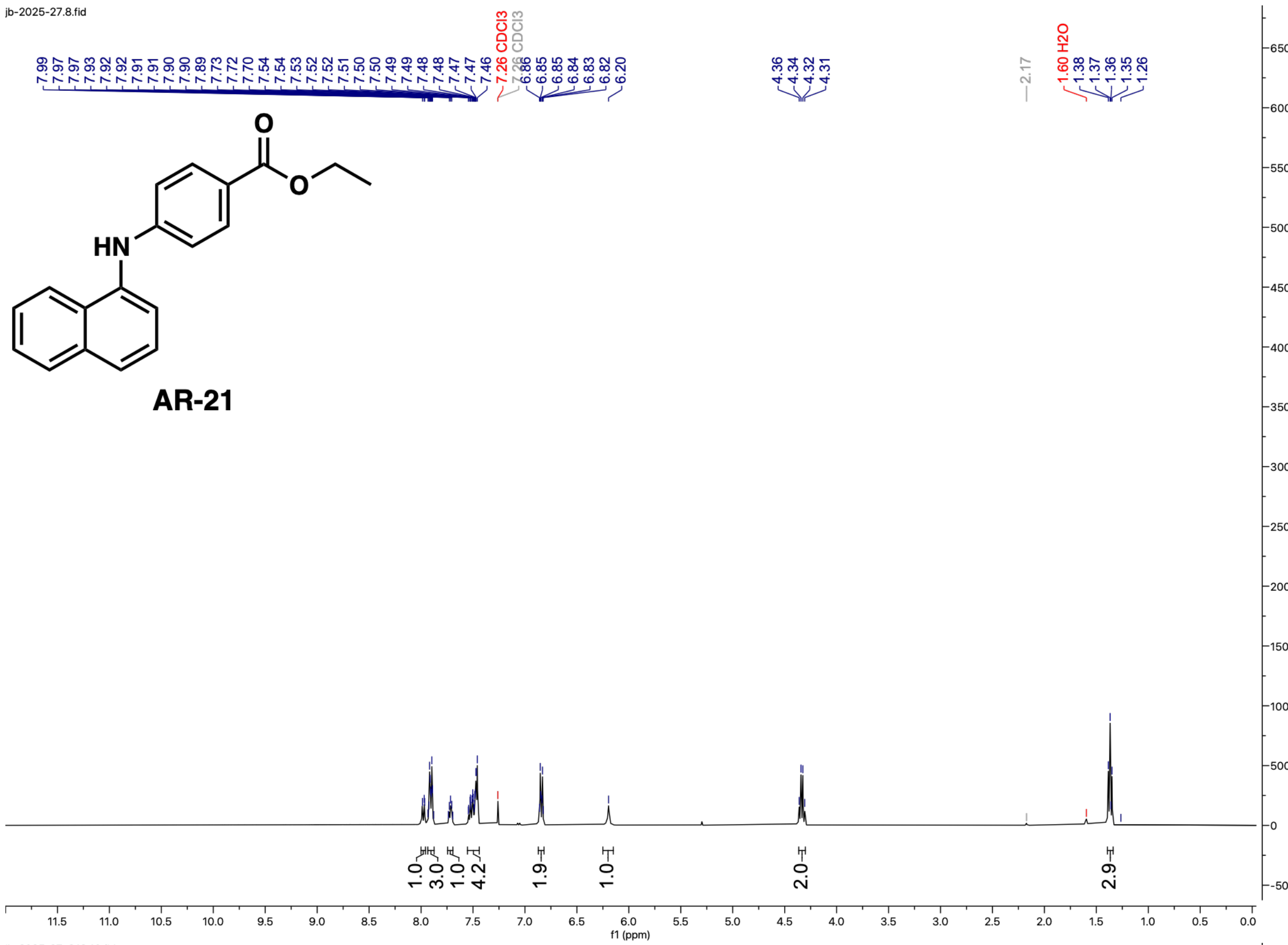

AR-21

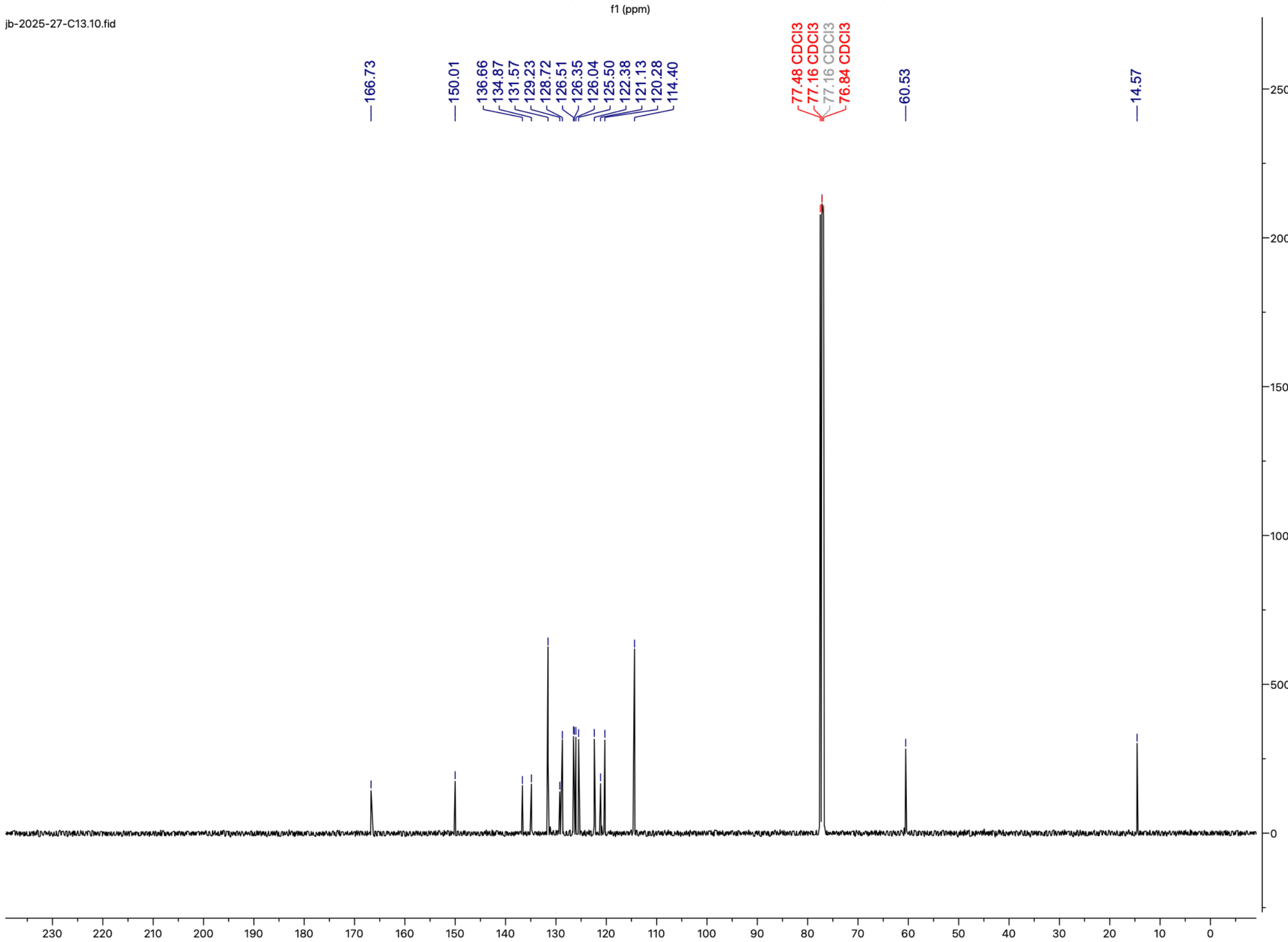

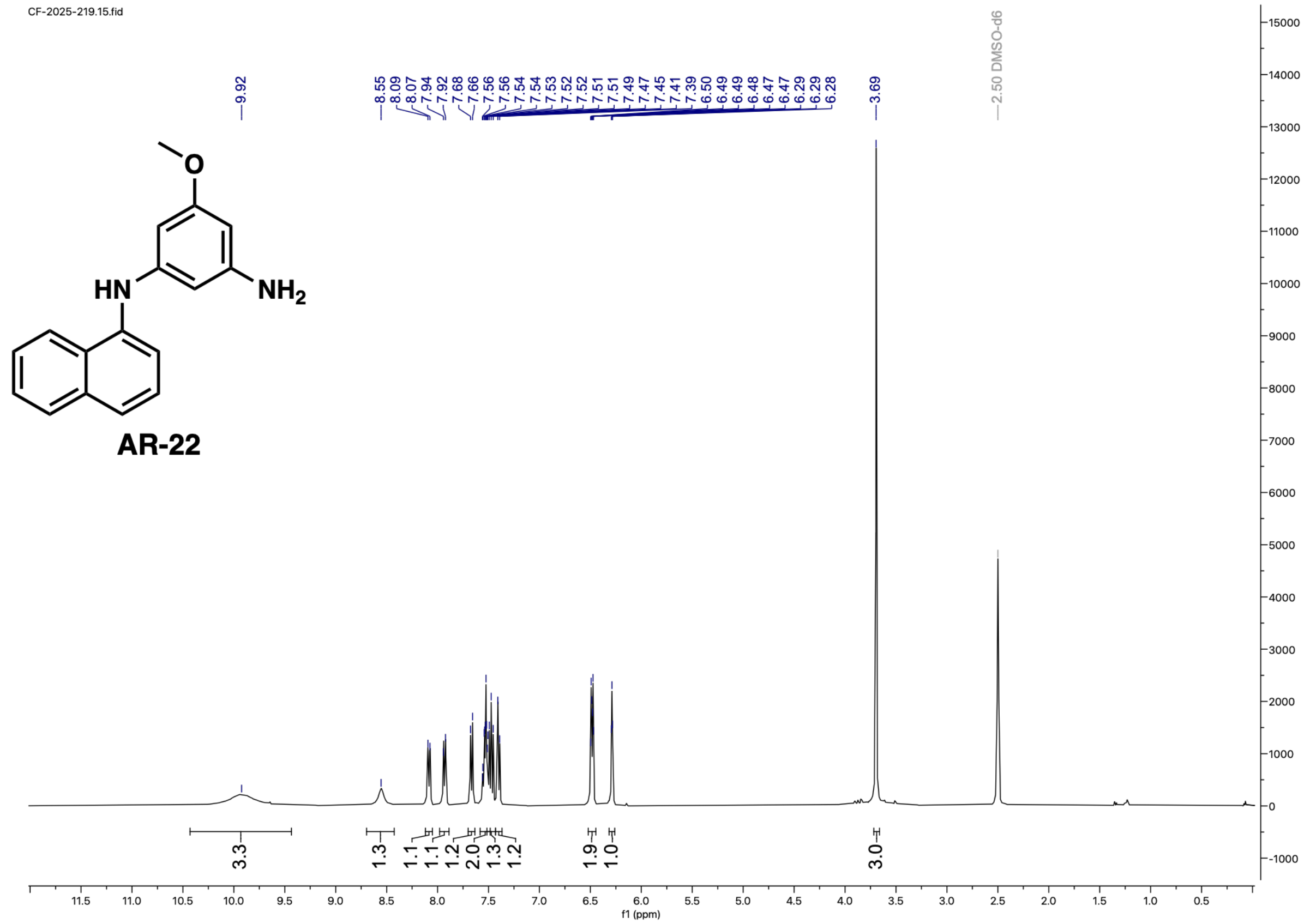
CF-2025-219.15.fid
9.92
8.55 8.09 8.07 7.94 7.92 7.68 7.66 7.56 7.56 7.54 7.54 7.53 7.52 7.52 7.51 7.51 7.49 7.47 7.45 7.41 7.39 6.50 6.49 6.49 6.48 6.47 6.47 6.29 6.29 6.28
3.69
2.50 DMSO-d6
O
HN
NH2
AR-22
3.3 1.3 1.1 1.1 1.2 2.0 1.3 1.2 1.9 1.0 3.0
f1 (ppm)

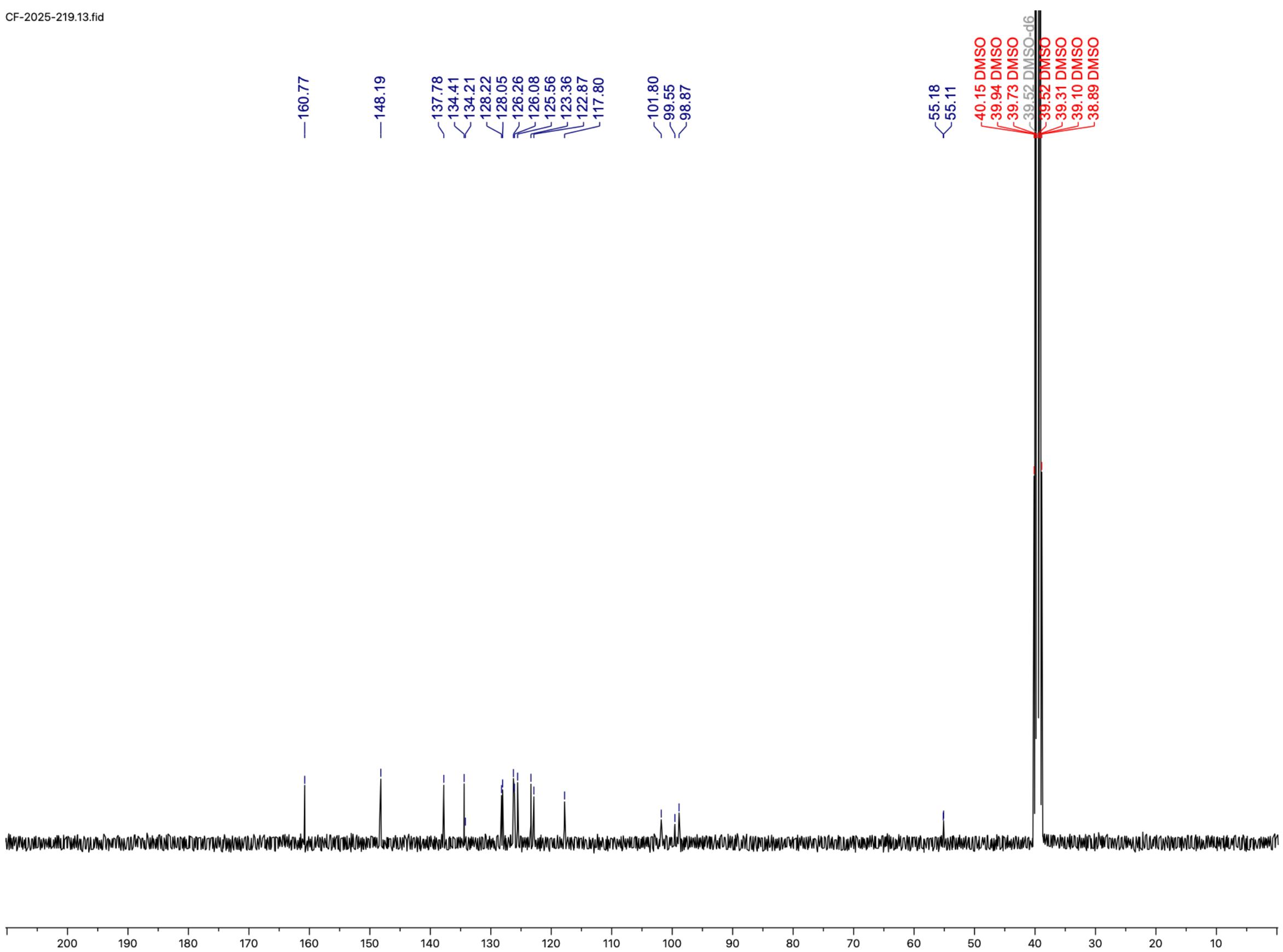
CF-2025-219.13.fid
160.77
148.19
137.78 134.41 134.21 128.22 128.05 126.26 126.08 125.56 123.36 122.87 117.80
101.80 99.55 98.87
55.18 55.11
40.15 DMSO 39.94 DMSO 39.73 DMSO 39.52 DMSO-d6 39.31 DMSO 39.10 DMSO 38.89 DMSO
f1 (ppm)

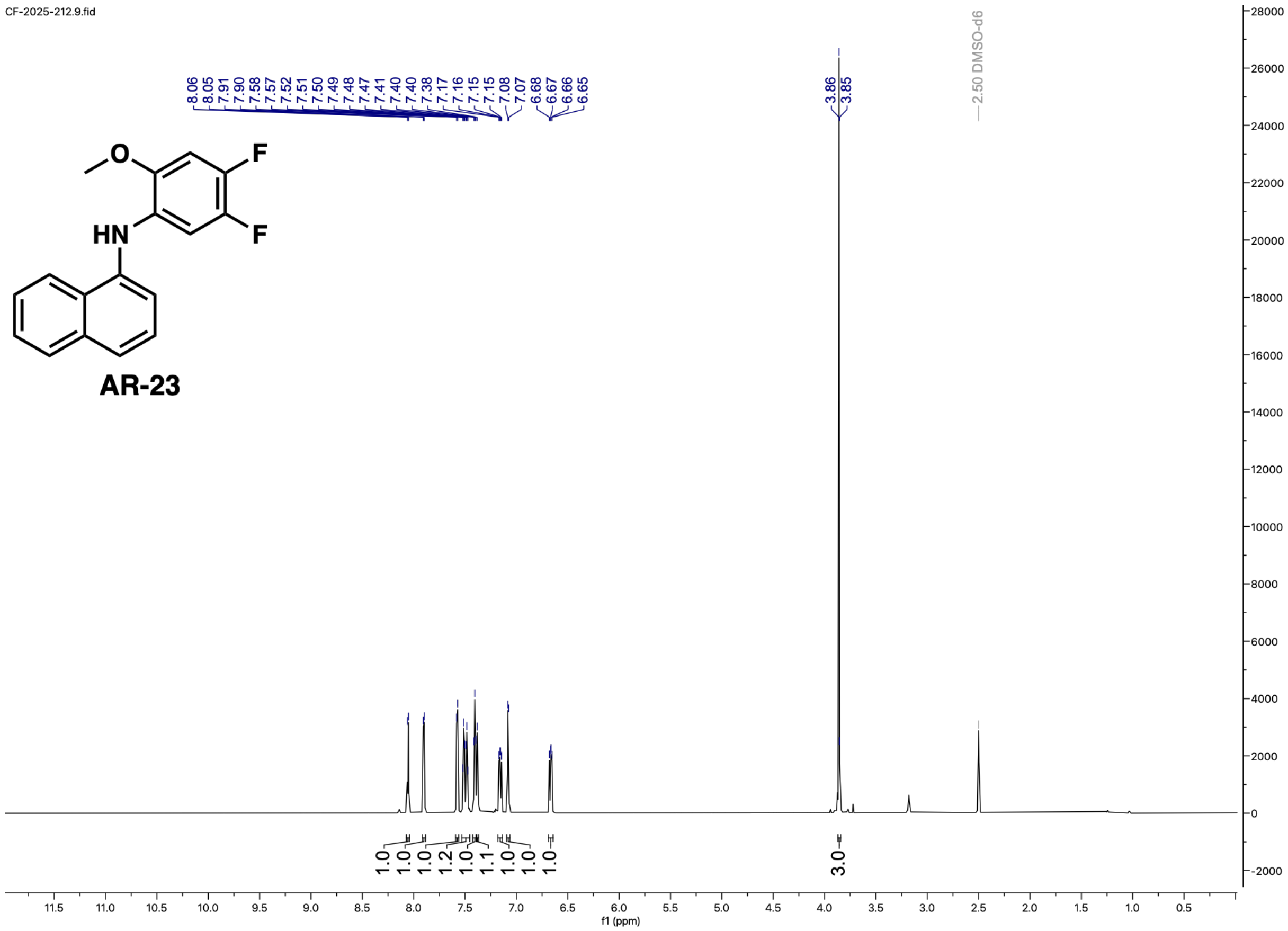

AR-23
CF-2025-212.9.fid
8.06
8.05
7.91
7.90
7.58
7.57
7.52
7.51
7.50
7.49
7.48
7.47
7.41
7.40
7.40
7.38
7.17
7.16
7.15
7.08
7.07
6.68
6.67
6.66
6.65
3.86
3.85
2.50 DMSO-d6
1.0
1.0
1.0
1.2
1.0
1.1
1.0
1.0
3.0
f1 (ppm)

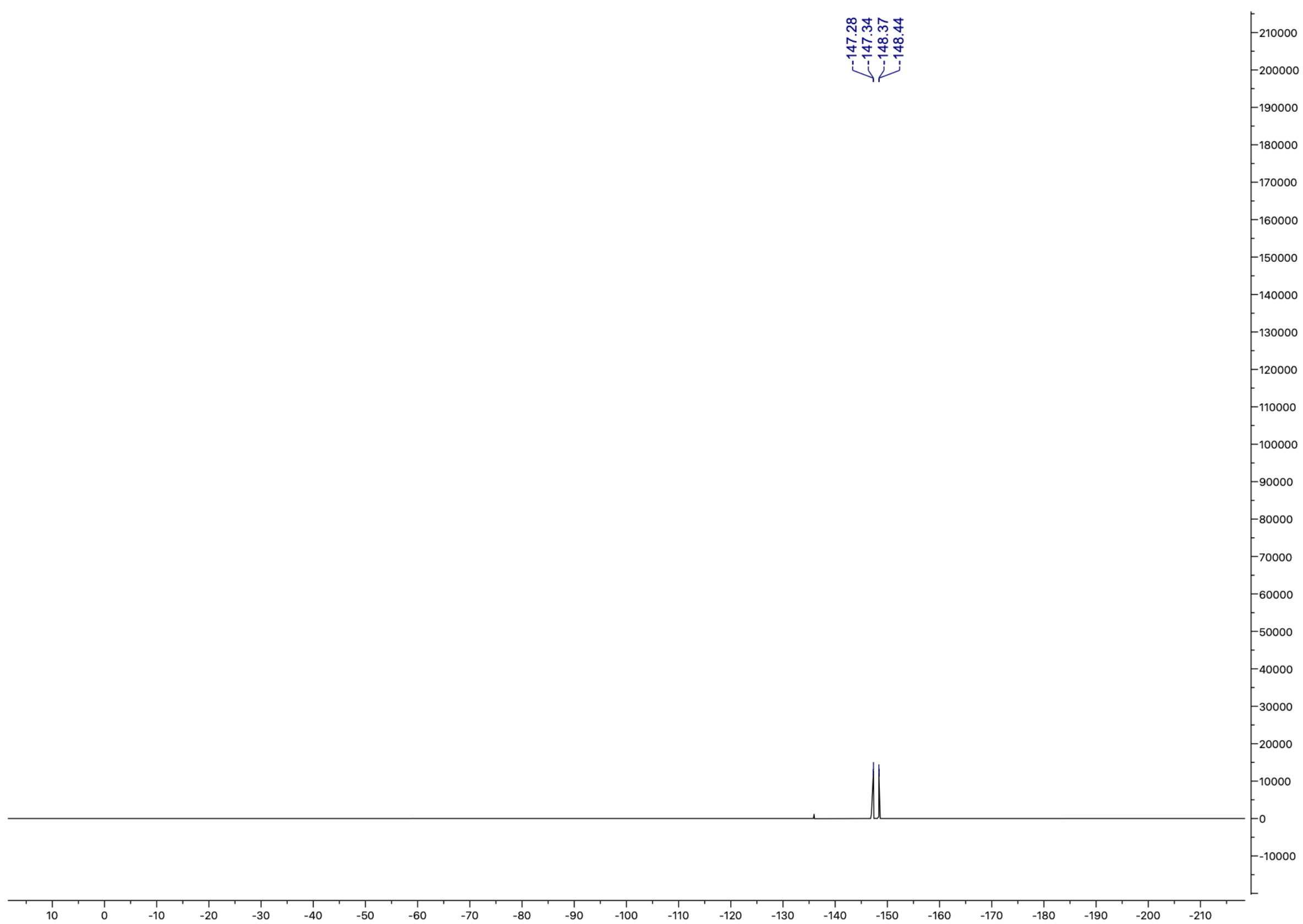

-147.28
-147.34
-148.37
-148.44
f1 (ppm)

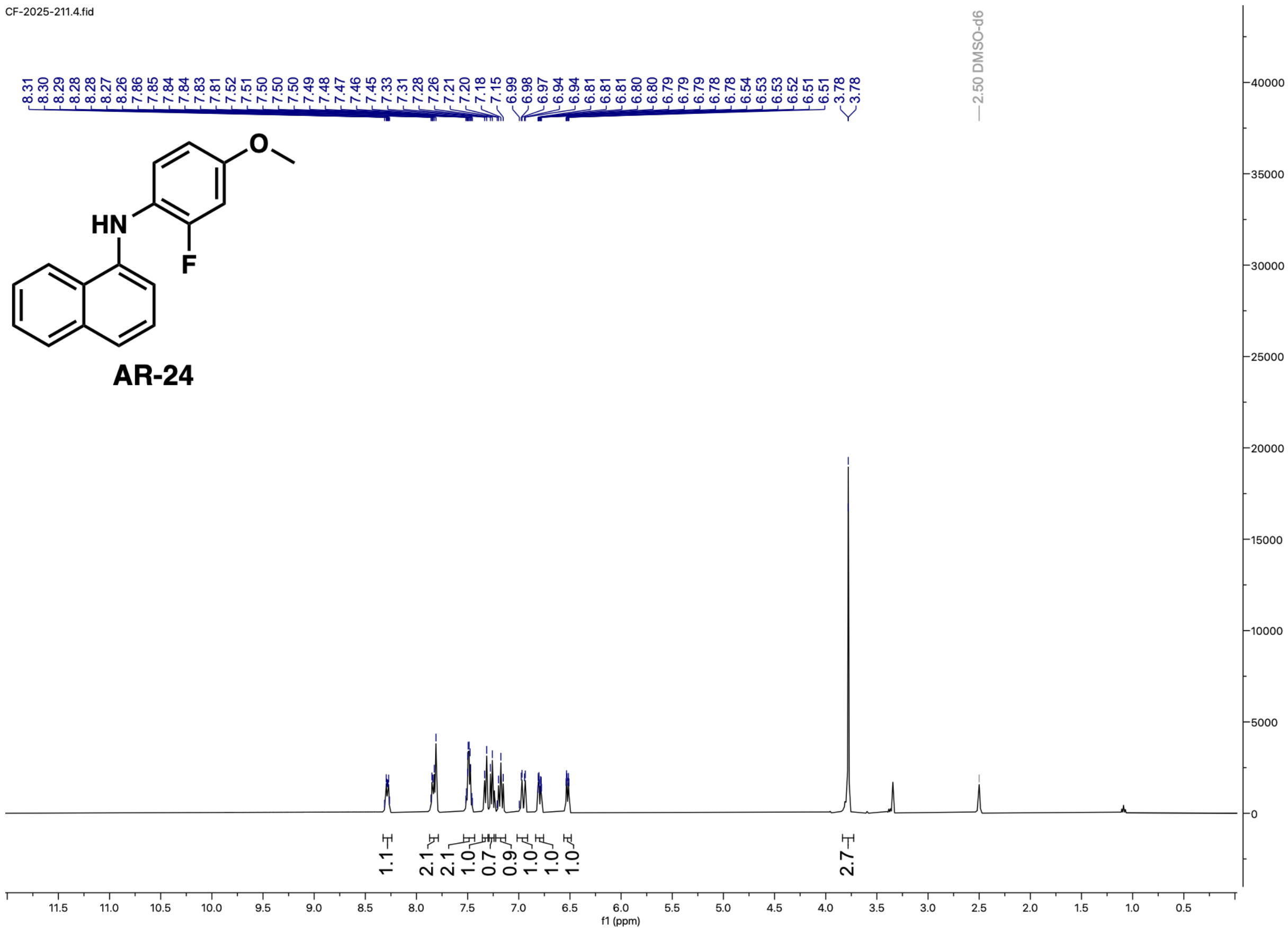
CF-2025-211.4.fid
AR-24
HN
F
O
8.31 8.30 8.29 8.28 8.28 8.27 8.26 7.86 7.85 7.84 7.84 7.83 7.81 7.52 7.51 7.50 7.50 7.50 7.49 7.48 7.47 7.46 7.45 7.33 7.31 7.28 7.26 7.21 7.20 7.18 7.15 6.99 6.98 6.97 6.94 6.94 6.81 6.81 6.81 6.80 6.80 6.79 6.79 6.79 6.78 6.54 6.53 6.52 6.51 6.51 3.78 3.78
2.50 DMSO-d6
1.1 2.1 2.1 1.0 0.7 0.9 1.0 1.0 1.0 2.7
f1 (ppm)

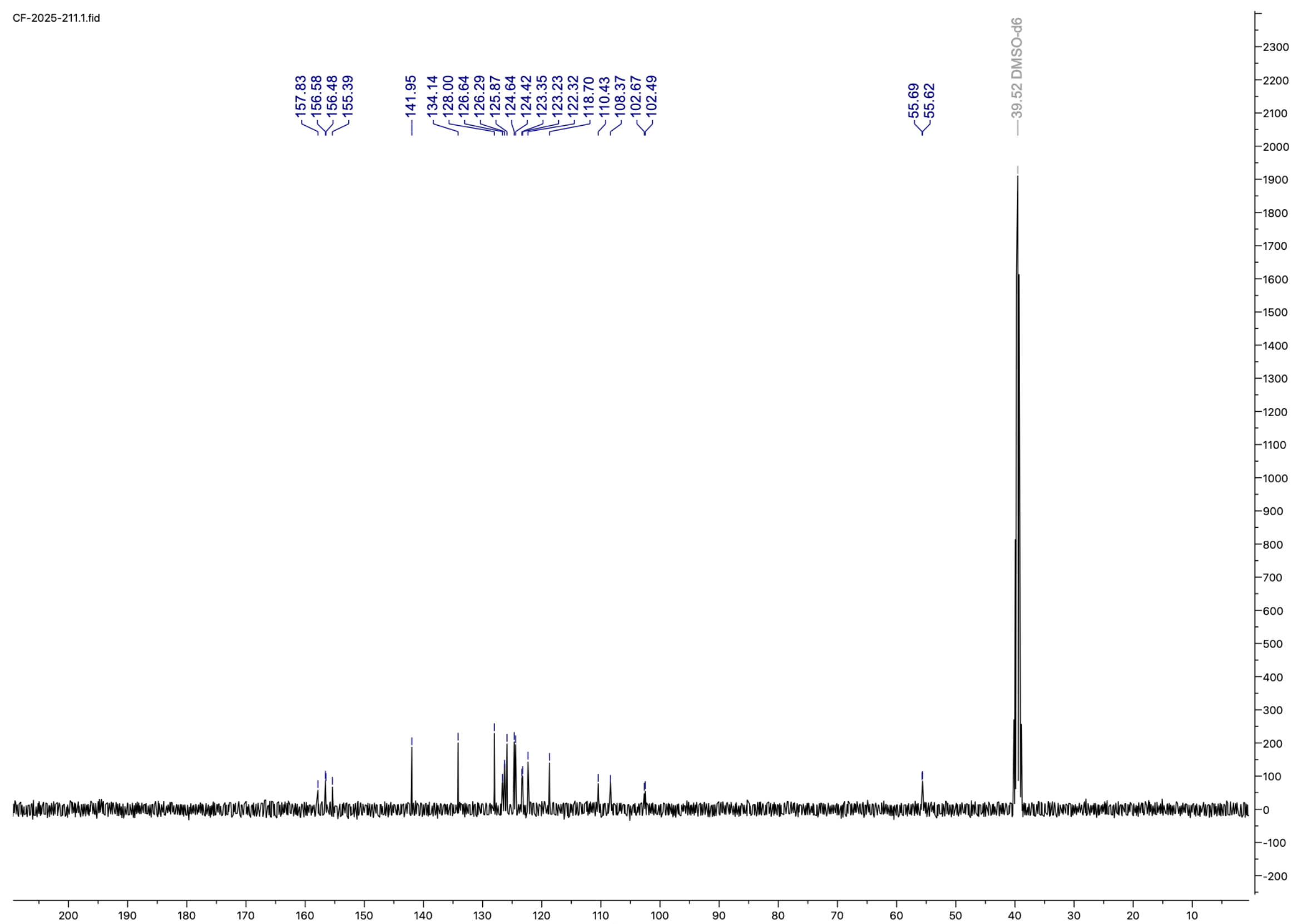
CF-2025-211.1.fid
157.83 156.58 156.48 155.39 141.95 134.14 128.00 126.64 126.29 125.87 124.64 124.42 123.35 123.23 122.32 118.70 110.43 108.37 102.67 102.49
55.69 55.62
39.52 DMSO-d6
f1 (ppm)

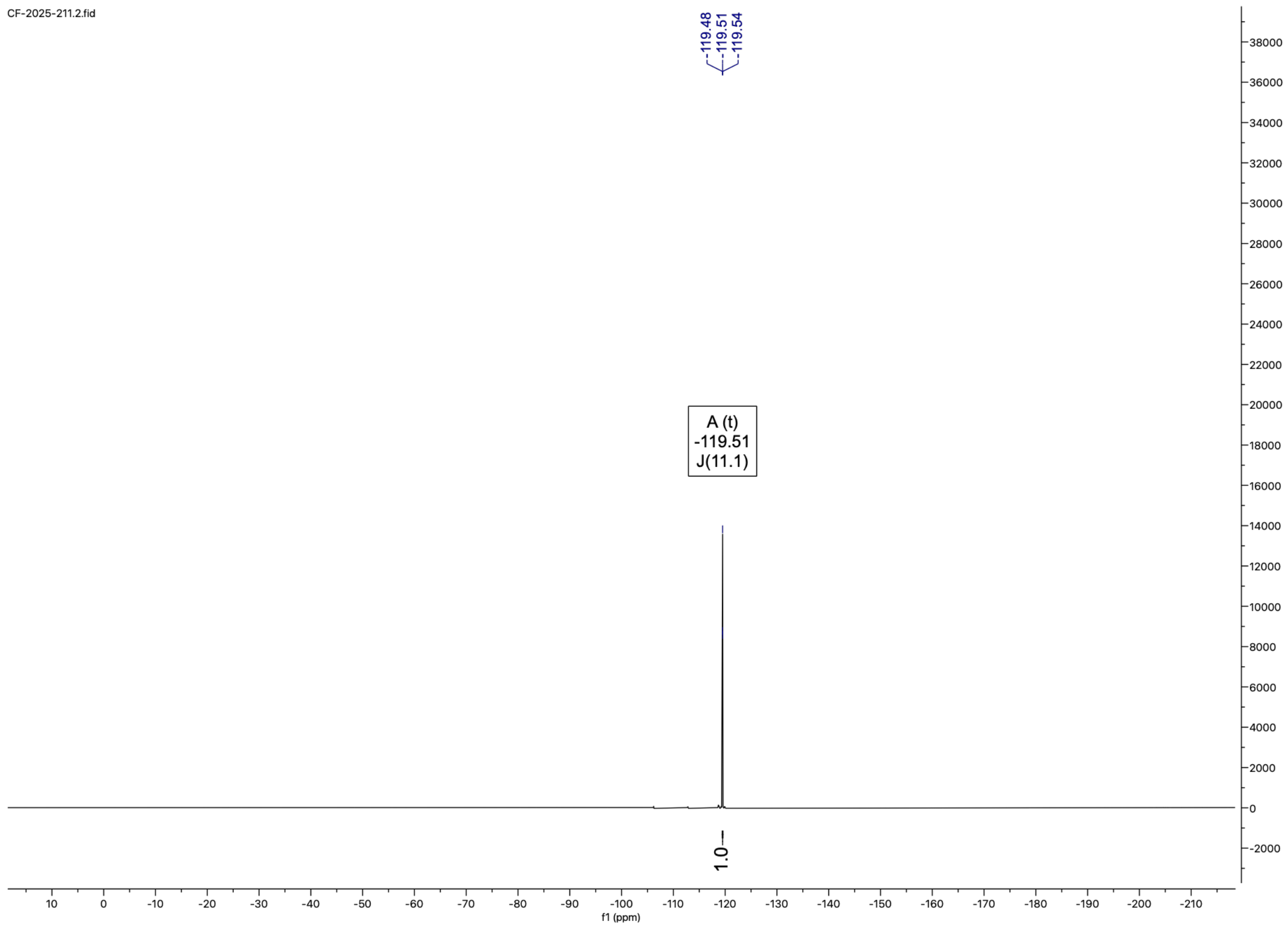
CF-2025-211.2.fid
-119.48
-119.51
-119.54
A (t)
-119.51
J(11.1)
1.0
f1 (ppm)

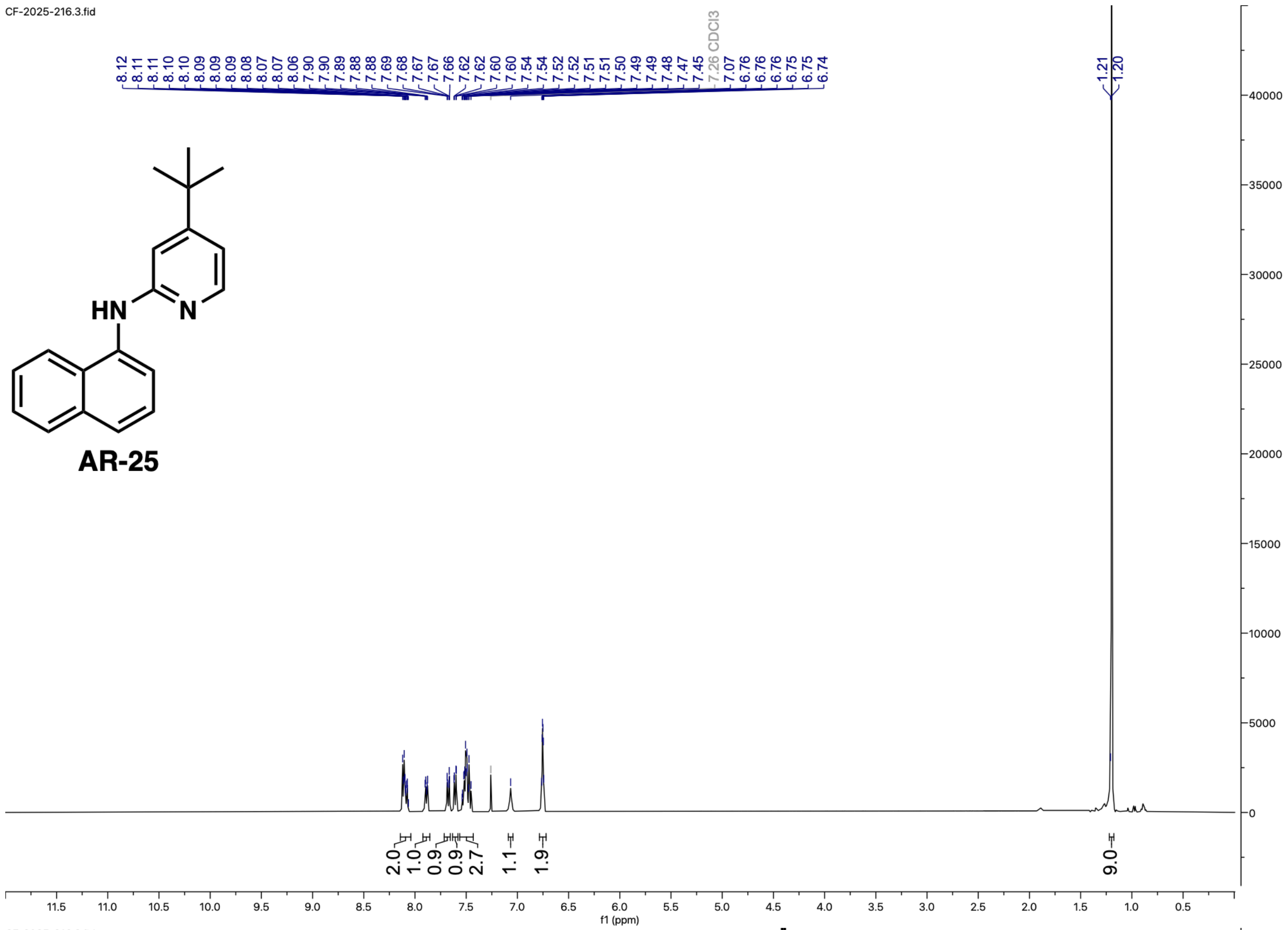
CF-2025-216.3.fid
8.12 8.11 8.11 8.10 8.09 8.09 8.08 8.07 8.07 8.06 7.90 7.90 7.89 7.88 7.88 7.69 7.68 7.67 7.67 7.66 7.62 7.62 7.60 7.60 7.54 7.54 7.52 7.52 7.51 7.50 7.49 7.49 7.48 7.47 7.45
7.26 CDCl3
7.07 6.76 6.76 6.76 6.75 6.75 6.74
1.21 1.20
HN
N
AR-25
2.0 1.0 0.9 0.9 2.7 1.1 1.9 9.0
f1 (ppm)

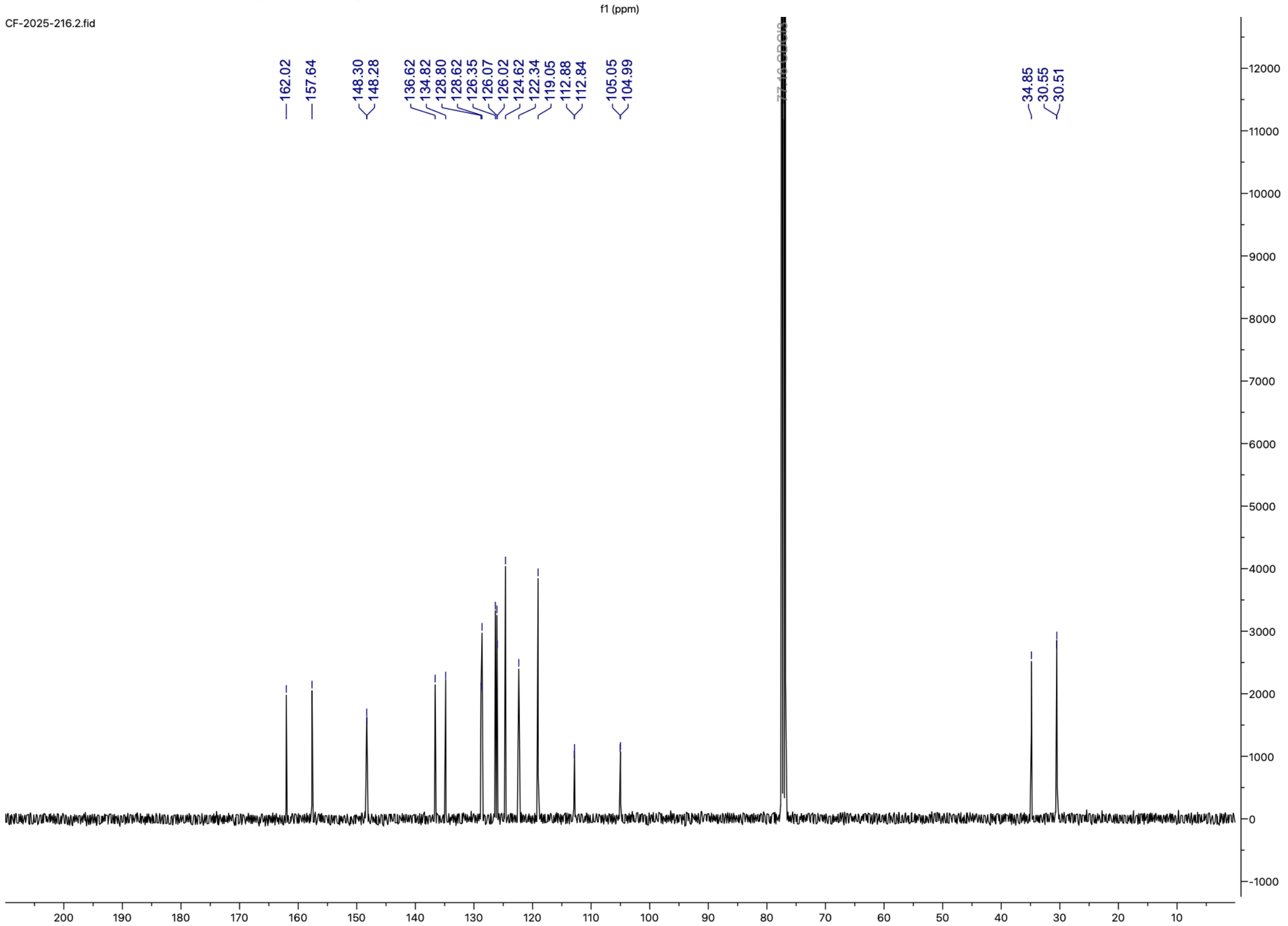
CF-2025-216.2.fid
162.02
157.64
148.30 148.28
136.62 134.82 128.80 128.62 126.35 126.07 126.02 124.62 122.34 119.05 112.88 112.84
105.05 104.99
34.85 30.55 30.51
f1 (ppm)

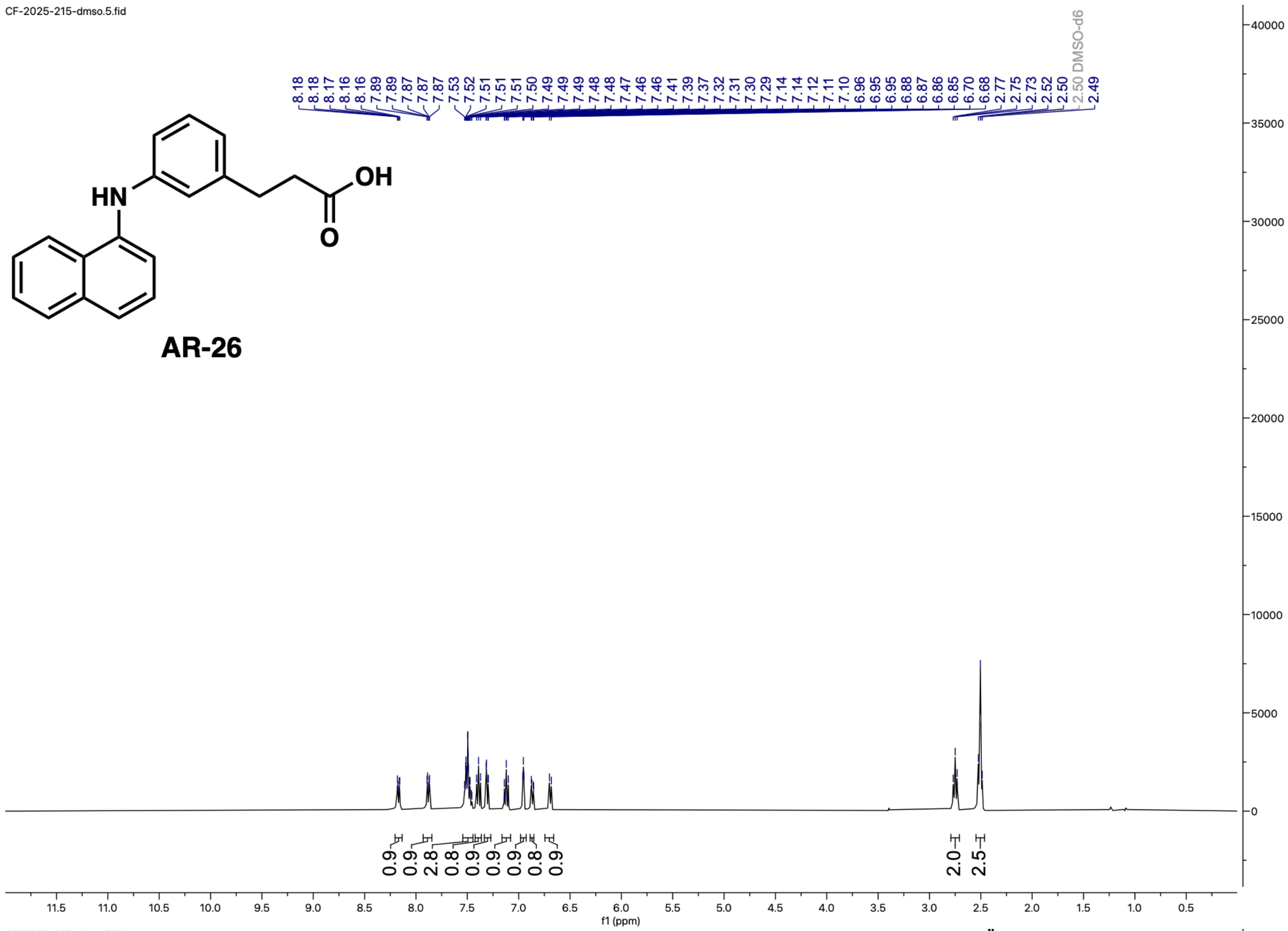
CF-2025-215-dmso.5.fid
HN
OH
O
AR-26
8.18
8.18
8.17
8.16
8.16
7.89
7.89
7.87
7.87
7.53
7.52
7.51
7.51
7.51
7.50
7.49
7.49
7.49
7.48
7.48
7.47
7.46
7.41
7.39
7.37
7.32
7.31
7.30
7.29
7.14
7.12
7.11
7.10
6.96
6.95
6.95
6.88
6.87
6.86
6.85
6.70
6.68
2.77
2.75
2.73
2.52
2.50 DMSO-d6
2.50
2.49
0.9
0.9
2.8
0.8
0.9
0.9
0.9
0.8
0.9
2.0
2.5
f1 (ppm)

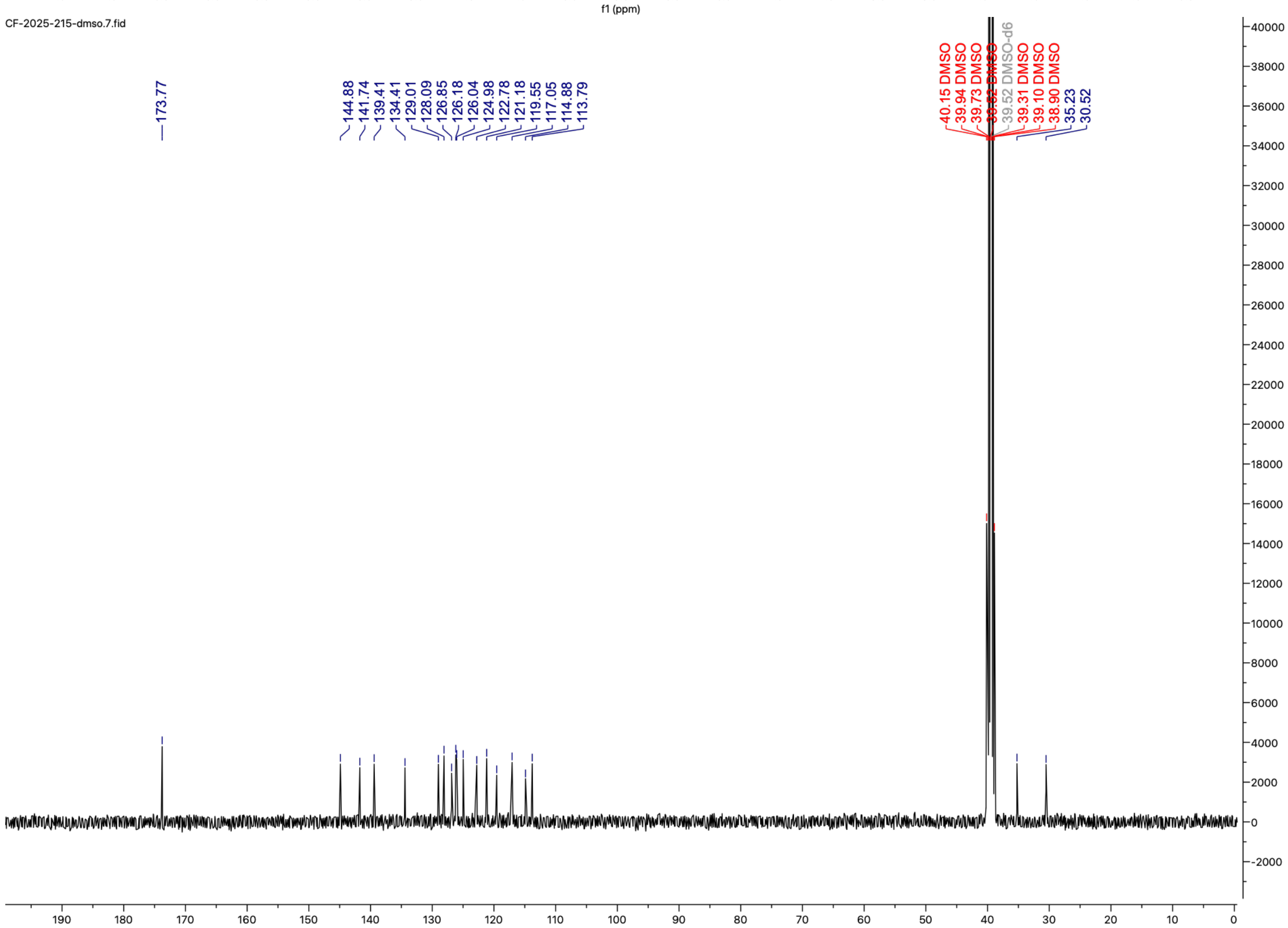
CF-2025-215-dmso.7.fid
173.77
144.88
141.74
139.41
134.41
129.01
128.09
126.85
126.18
126.04
124.98
122.78
121.18
119.55
117.05
114.88
113.79
40.15 DMSO
39.94 DMSO
39.73 DMSO
39.52 DMSO-d6
39.31 DMSO
39.10 DMSO
38.90 DMSO
35.23
30.52
f1 (ppm)

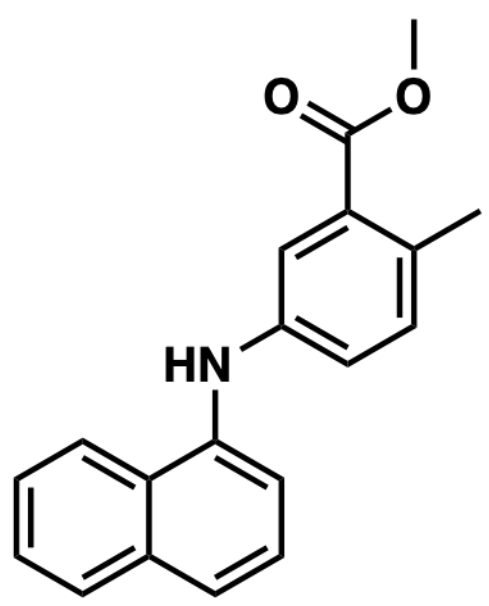

AR-27

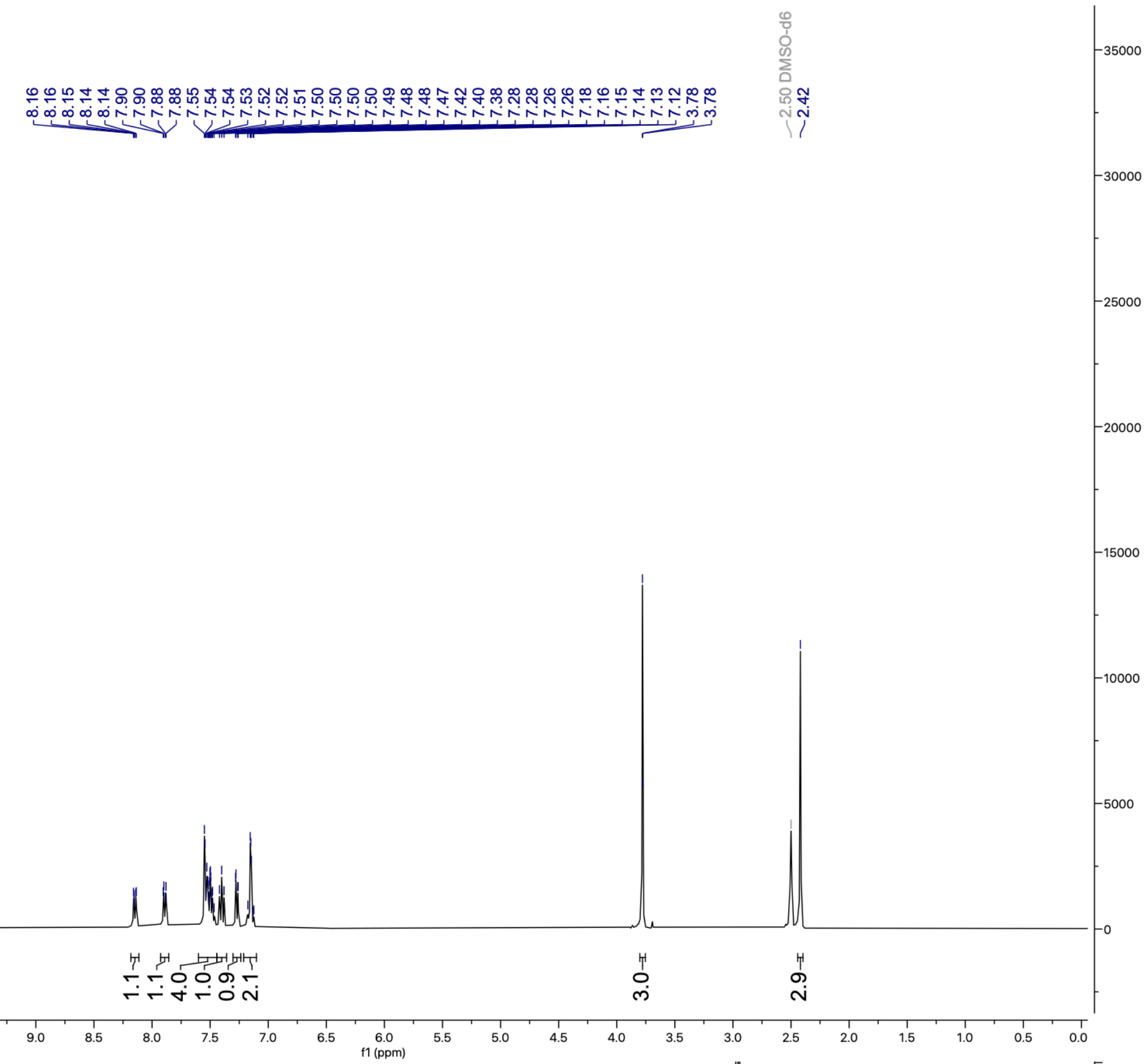
CF-2025-220-dmso.4.fid
8.16 8.16 8.15 8.14 8.14 7.90 7.88 7.88 7.55 7.54 7.54 7.53 7.52 7.52 7.51 7.50 7.50 7.50 7.50 7.49 7.48 7.48 7.47 7.42 7.40 7.38 7.28 7.28 7.26 7.26 7.18 7.16 7.15 7.14 7.13 7.12 3.78 3.78
2.50 DMSO-d6
2.42
1.1 1.1 4.0 1.0 0.9 2.1 3.0 2.9
f1 (ppm)

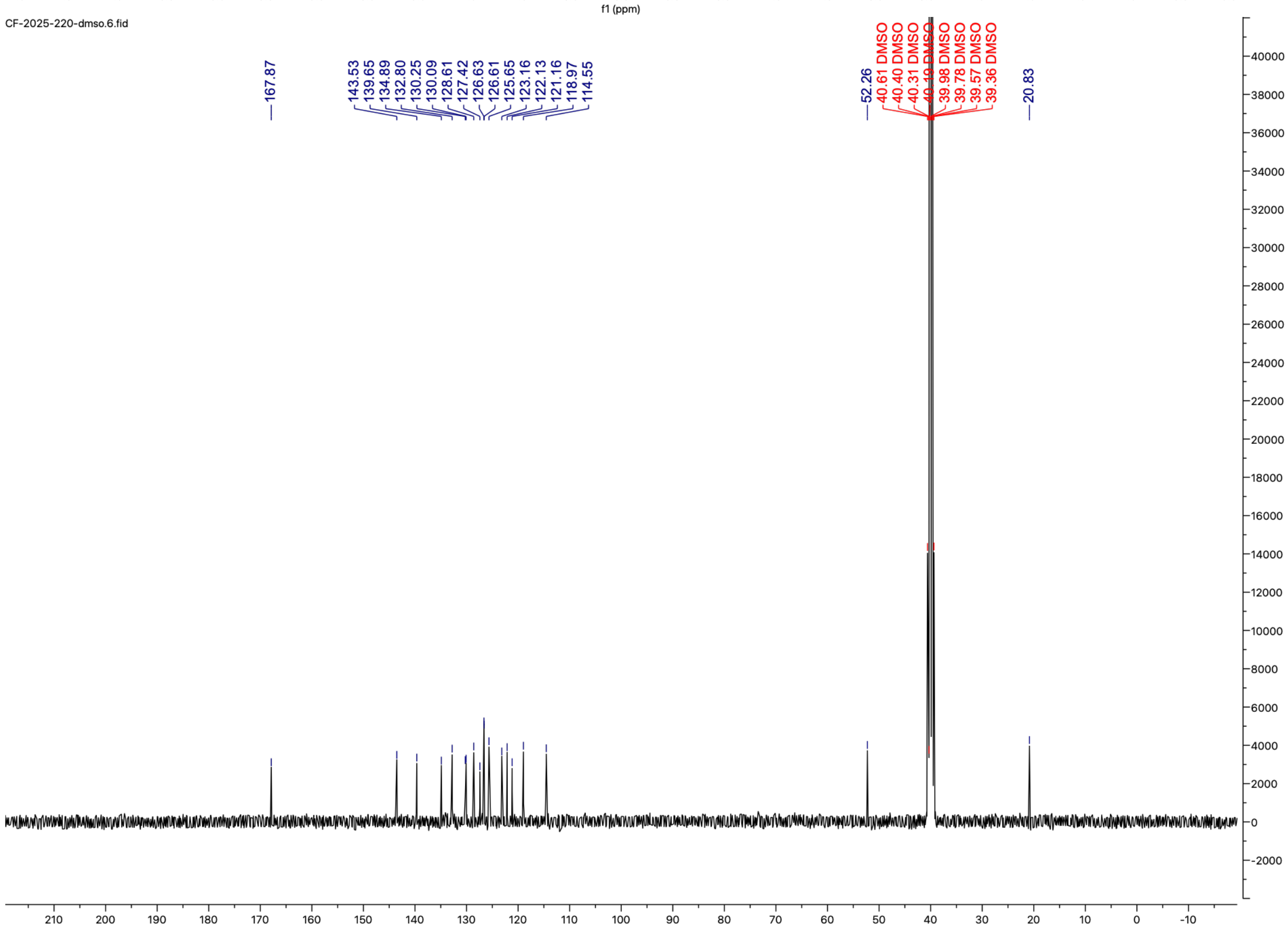
CF-2025-220-dmso.6.fid
167.87
143.53 139.65 134.89 132.80 130.25 130.09 128.61 127.42 126.63 126.61 125.65 123.16 122.13 121.16 118.97 114.55
52.26
40.61 DMSO 40.40 DMSO 40.31 DMSO 40.19 DMSO 39.98 DMSO 39.78 DMSO 39.57 DMSO 39.36 DMSO
20.83
f1 (ppm)

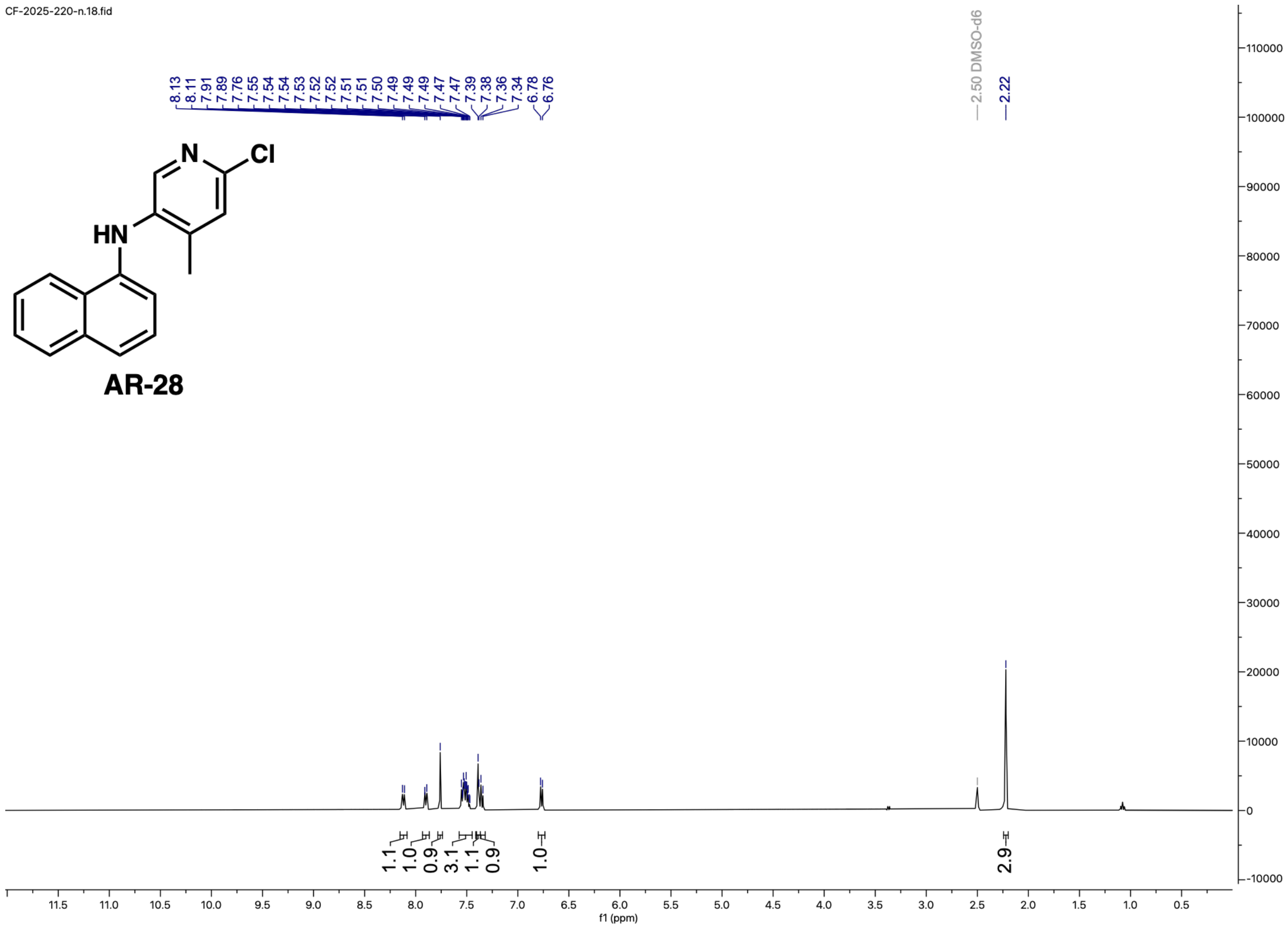
CF-2025-220-n.18.fid
8.13 8.11 7.91 7.89 7.76 7.55 7.54 7.54 7.53 7.52 7.52 7.51 7.51 7.50 7.49 7.49 7.49 7.47 7.47 7.39 7.38 7.36 7.34 6.78 6.76
2.50 DMSO-d6
2.22
N
Cl
HN
AR-28
1.1 1.0 0.9 3.1 1.1 0.9 1.0 2.9
f1 (ppm)

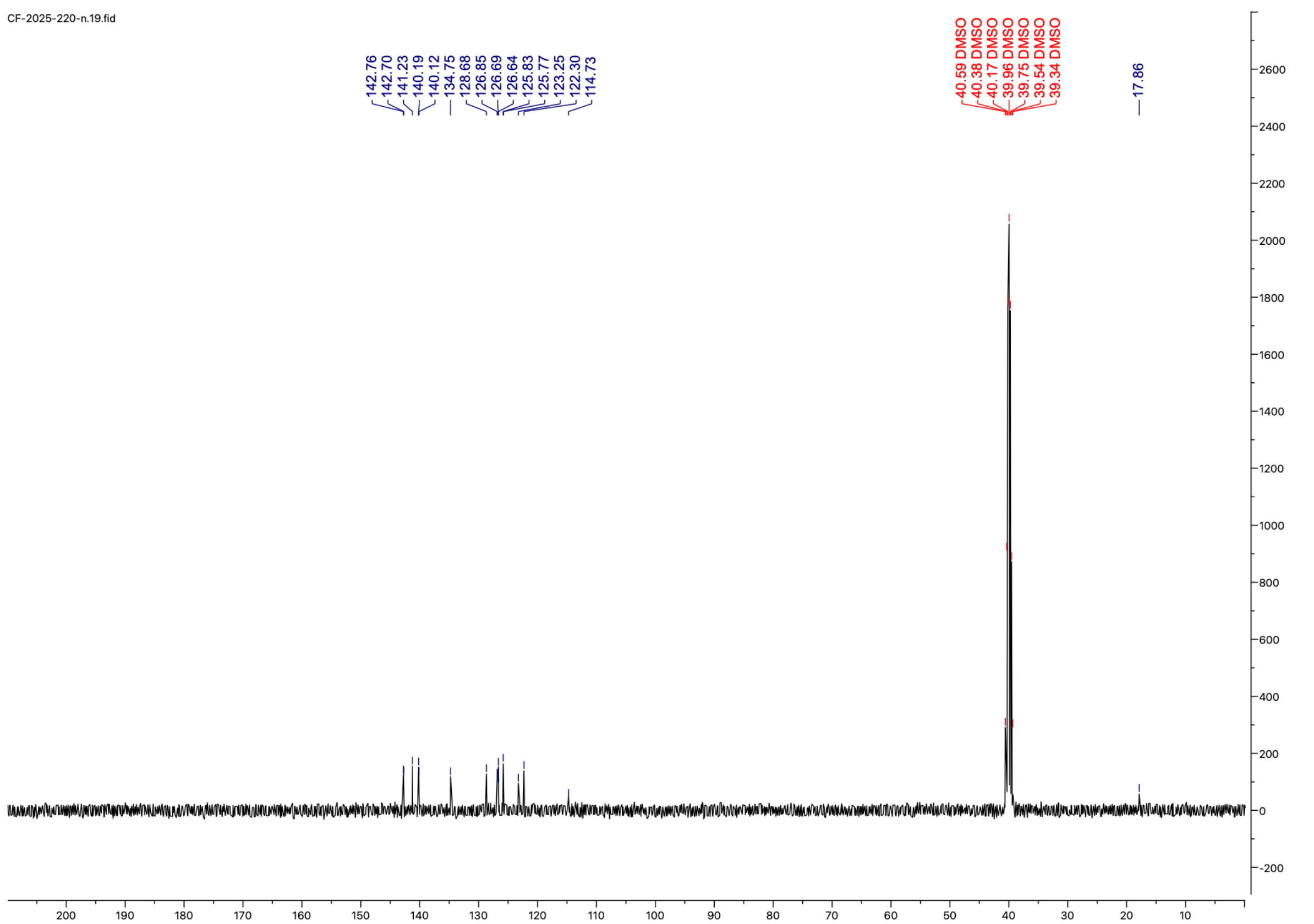
CF-2025-220-n.19.fid
142.76 142.70 141.23 140.19 140.12 134.75 128.68 126.85 126.69 126.64 125.83 125.77 123.25 122.30 114.73
40.59 DMSO 40.38 DMSO 40.17 DMSO 39.96 DMSO 39.75 DMSO 39.54 DMSO 39.34 DMSO
17.86
f1 (ppm)

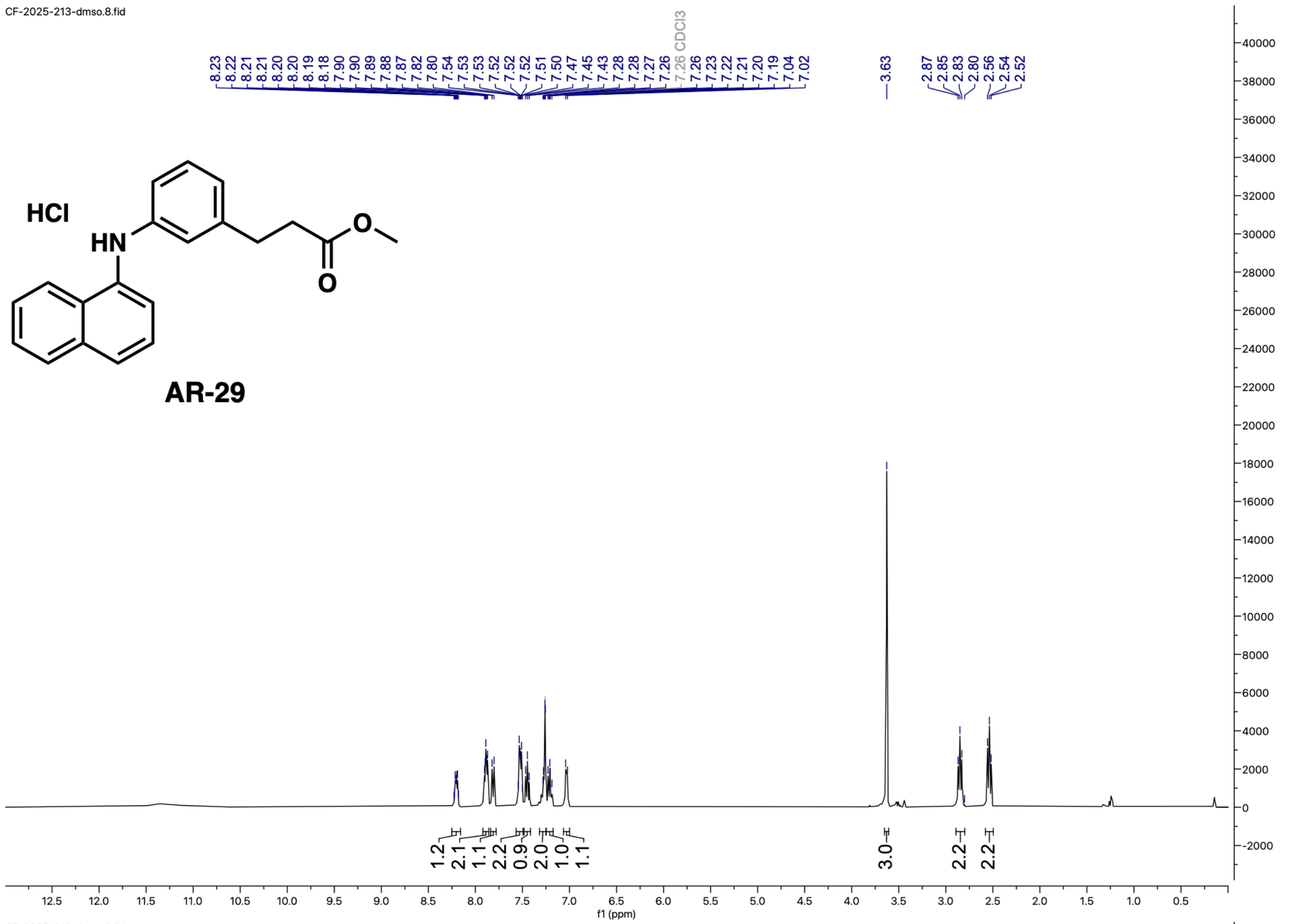
CF-2025-213-dmso.8.fid
HCl
HN
O
O
AR-29
8.23 8.22 8.21 8.21 8.20 8.20 8.19 8.18 7.90 7.90 7.89 7.88 7.87 7.82 7.80 7.54 7.53 7.53 7.52 7.52 7.52 7.51 7.50 7.47 7.45 7.43 7.28 7.28 7.27 7.26 7.26 CDCl3 7.26 7.23 7.22 7.21 7.20 7.19 7.04 7.02
3.63
2.87 2.85 2.83 2.80 2.56 2.54 2.52
1.2 2.1 1.1 2.2 0.9 2.0 1.0 1.1
3.0
2.2 2.2
f1 (ppm)

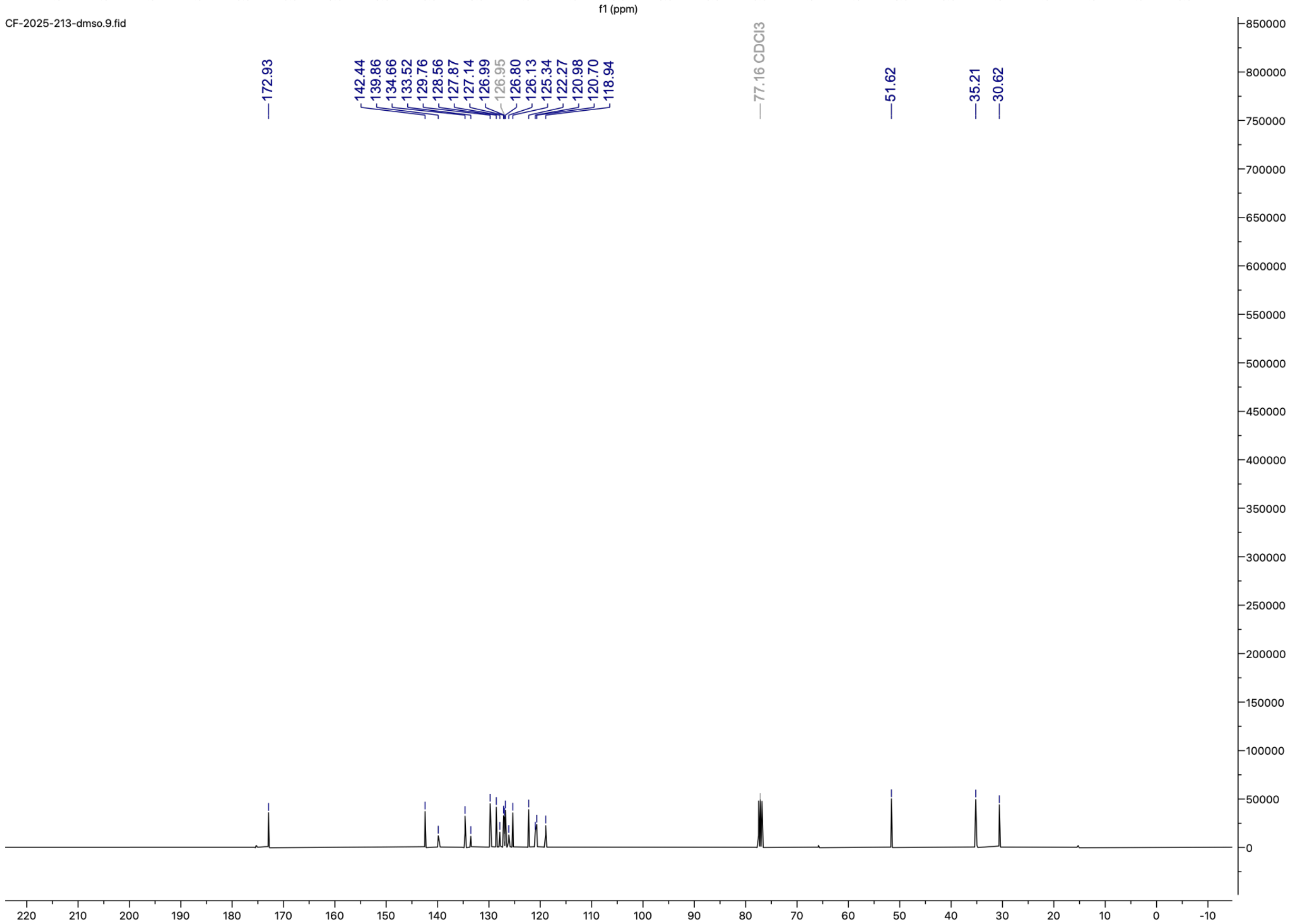
CF-2025-213-dmso.9.fid
172.93
142.44 139.86 134.66 133.52 129.76 128.56 127.87 127.14 126.99 126.95 126.80 126.13 125.34 122.27 120.98 120.70 118.94
77.16 CDCl3
51.62
35.21 30.62
f1 (ppm)

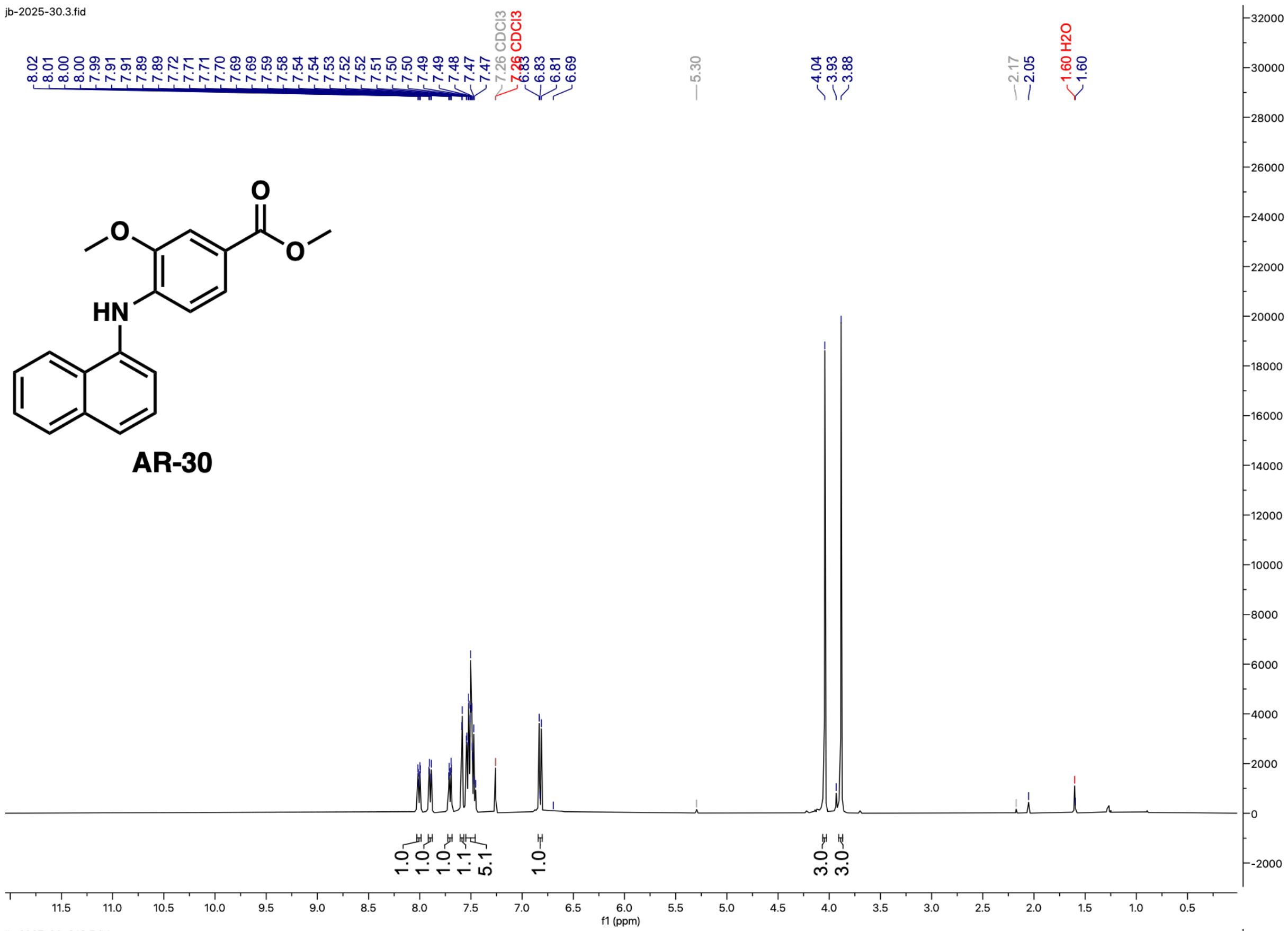
jb-2025-30.3.fid
8.02 8.01 8.00 8.00 7.99 7.91 7.91 7.89 7.72 7.71 7.71 7.70 7.69 7.69 7.59 7.58 7.54 7.54 7.53 7.52 7.52 7.51 7.50 7.49 7.49 7.48 7.47 7.47
7.26 CDCl3
7.26 CDCl3
6.83 6.83 6.81 6.69
5.30
4.04 3.93 3.88
2.17 2.05
1.60 H2O
1.60
AR-30
1.0 1.0 1.0 1.1 5.1 1.0 3.0 3.0
f1 (ppm)

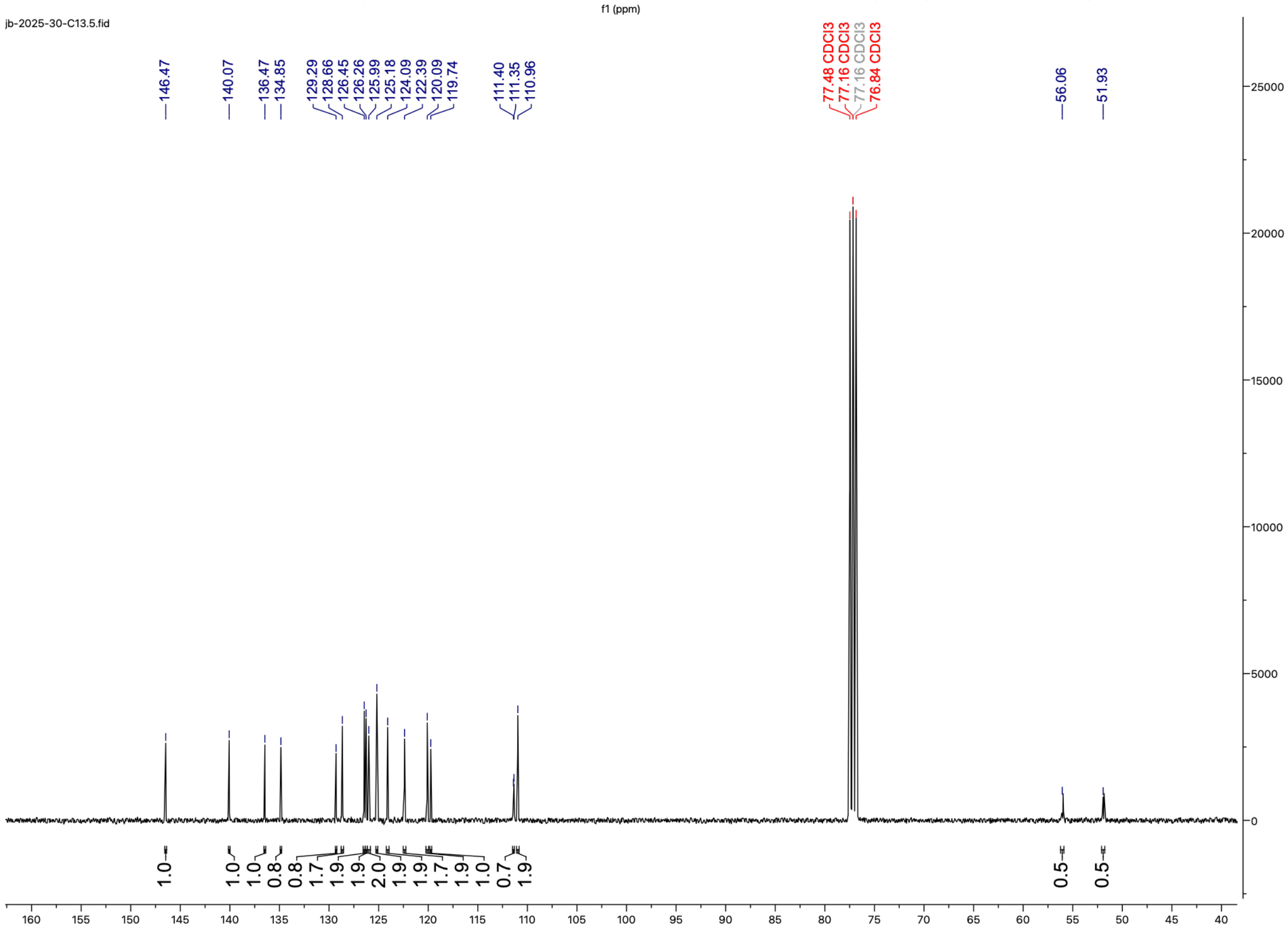
jb-2025-30-C13.5.fid
146.47 140.07 136.47 134.85 129.29 128.66 126.45 126.26 125.99 125.18 124.09 122.39 120.09 119.74 111.40 111.35 110.96
77.48 CDCl3
77.16 CDCl3
77.16 CDCl3
76.84 CDCl3
56.06 51.93
1.0 1.0 1.0 0.8 0.8 1.7 1.9 1.9 2.0 1.9 1.9 1.7 1.9 1.0 0.7 1.9 0.5 0.5
f1 (ppm)

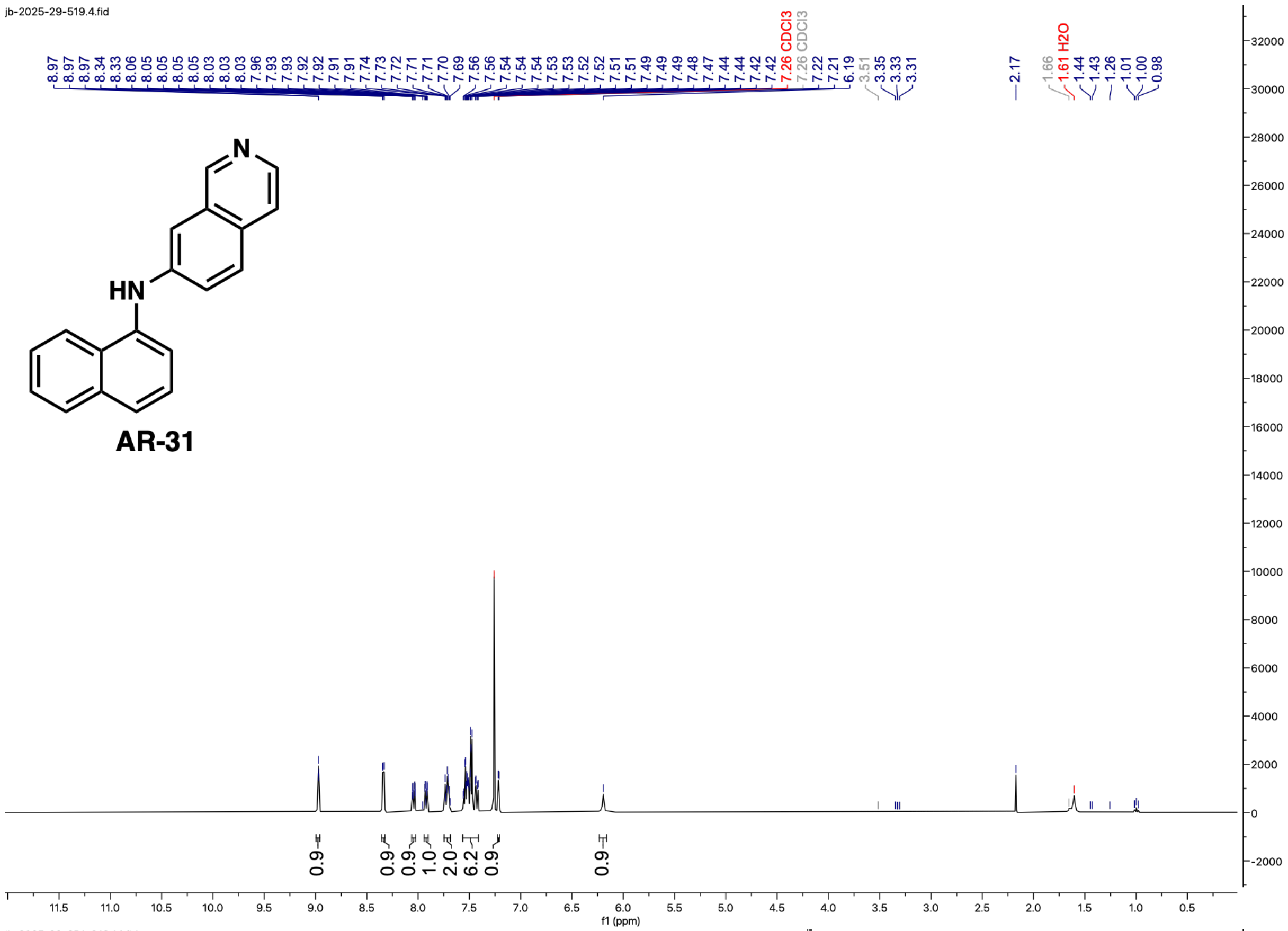
jb-2025-29-519.4.fid
8.97 8.97 8.97 8.34 8.33 8.06 8.05 8.05 8.05 8.05 8.03 8.03 8.03 7.96 7.93 7.93 7.92 7.92 7.91 7.91 7.74 7.73 7.72 7.71 7.71 7.70 7.69 7.56 7.56 7.54 7.54 7.53 7.53 7.52 7.52 7.51 7.51 7.49 7.49 7.48 7.47 7.44 7.44 7.42 7.42
7.26 CDCl3
7.26 CDCl3
7.22 7.21 6.19 3.51 3.35 3.33 3.31 2.17 1.66
1.61 H2O
1.44 1.43 1.26 1.01 1.00 0.98
N
HN
AR-31
0.9 0.9 0.9 1.0 2.0 6.2 0.9 0.9
f1 (ppm)

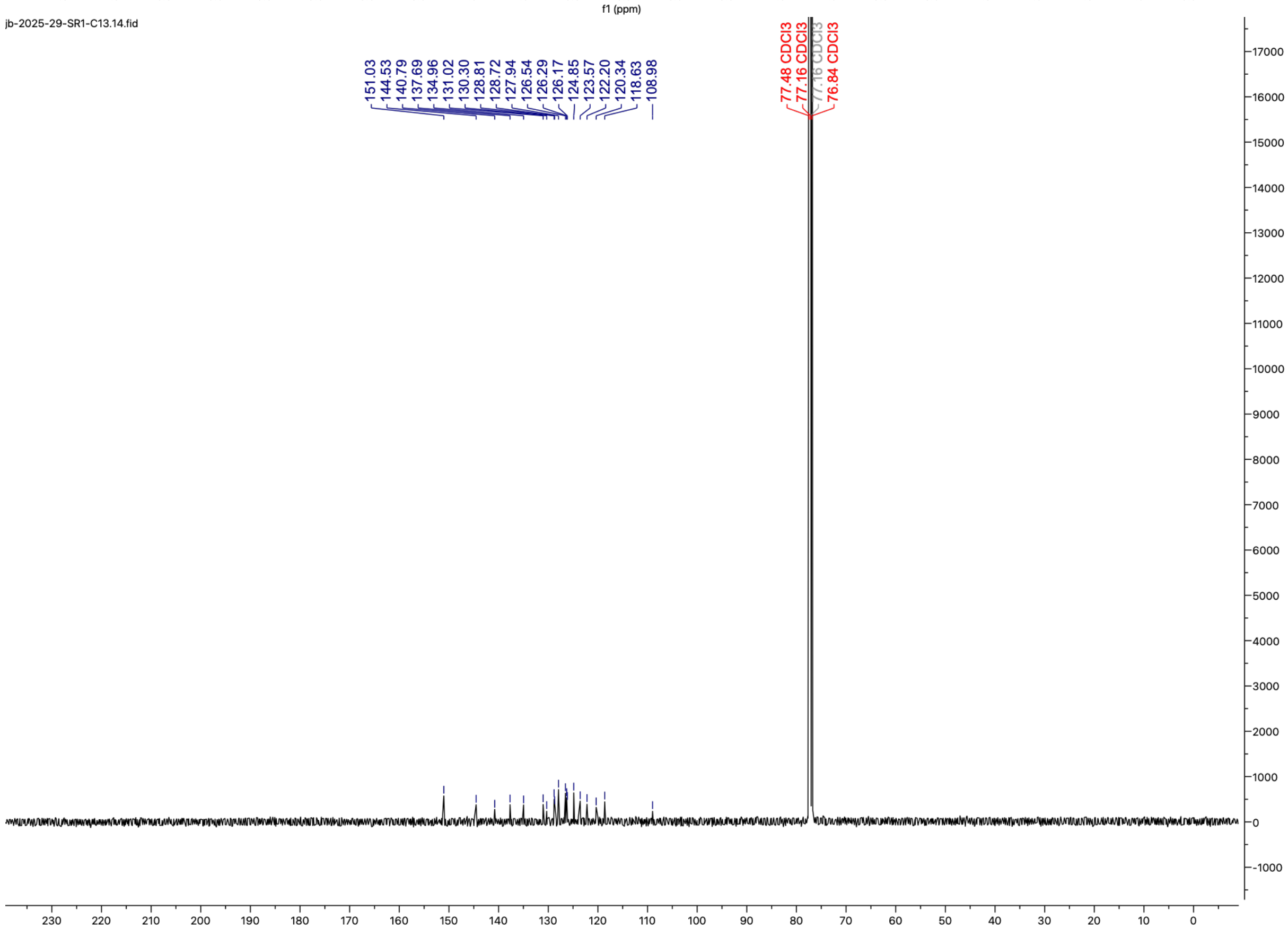
jb-2025-29-SR1-C13.14.fid
151.03 144.53 140.79 137.69 134.96 131.02 130.30 128.81 128.72 127.94 126.54 126.29 126.17 124.85 123.57 122.20 120.34 118.63 108.98
77.48 CDCl3
77.16 CDCl3
77.16 CDCl3
76.84 CDCl3
f1 (ppm)

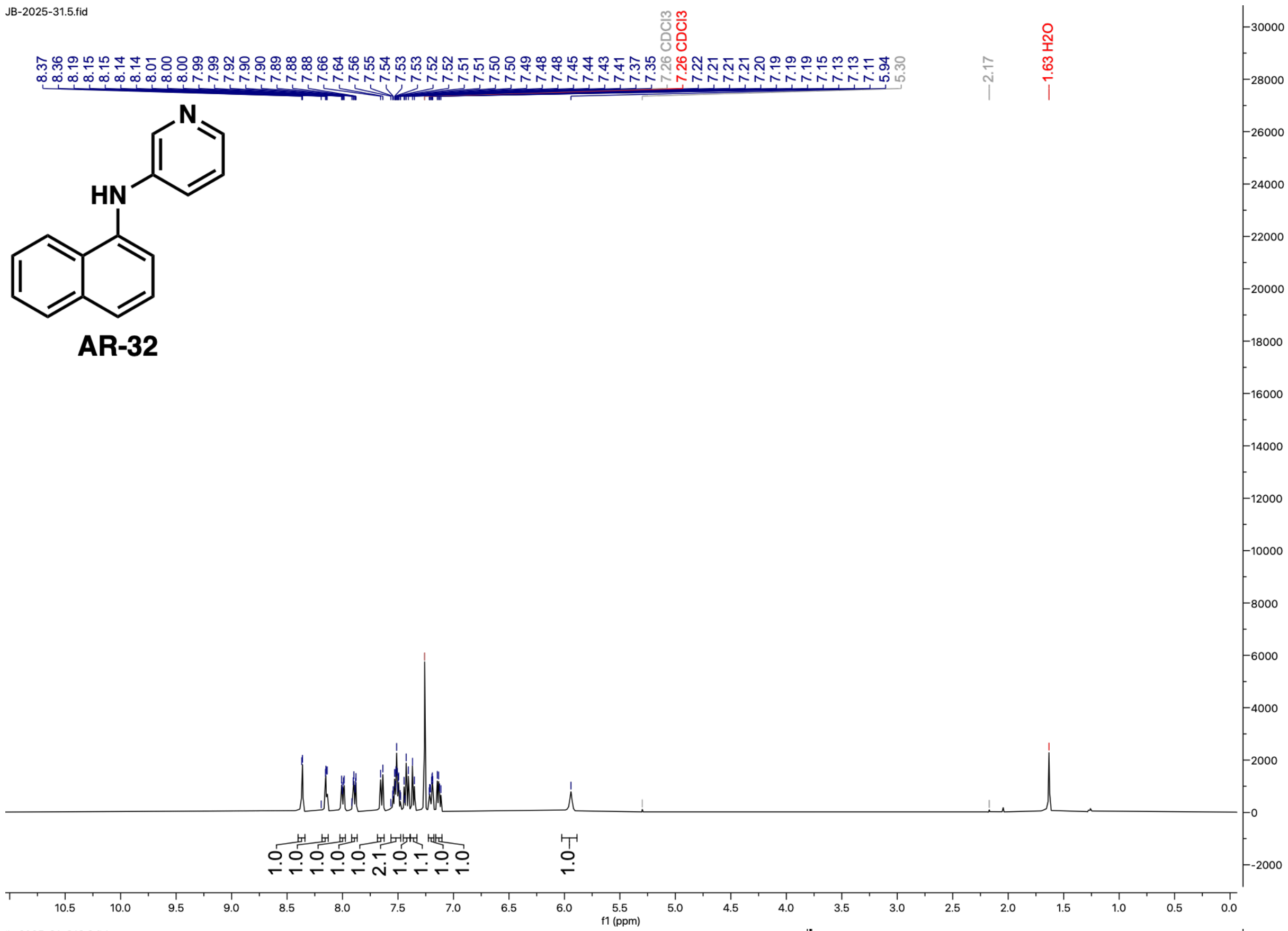
JB-2025-31.5.fid
8.37
8.36
8.19
8.15
8.15
8.14
8.14
8.01
8.00
8.00
7.99
7.92
7.90
7.90
7.89
7.88
7.88
7.66
7.64
7.56
7.55
7.54
7.53
7.53
7.52
7.52
7.51
7.51
7.50
7.50
7.49
7.48
7.48
7.45
7.44
7.43
7.41
7.37
7.35
7.26 CDCl3
7.26 CDCl3
7.22
7.21
7.21
7.21
7.20
7.19
7.19
7.19
7.15
7.13
7.13
7.11
5.94
5.30
2.17
1.63 H2O
HN
N
AR-32
1.0
1.0
1.0
1.0
1.0
2.1
1.0
1.1
1.0
1.0
1.0
f1 (ppm)

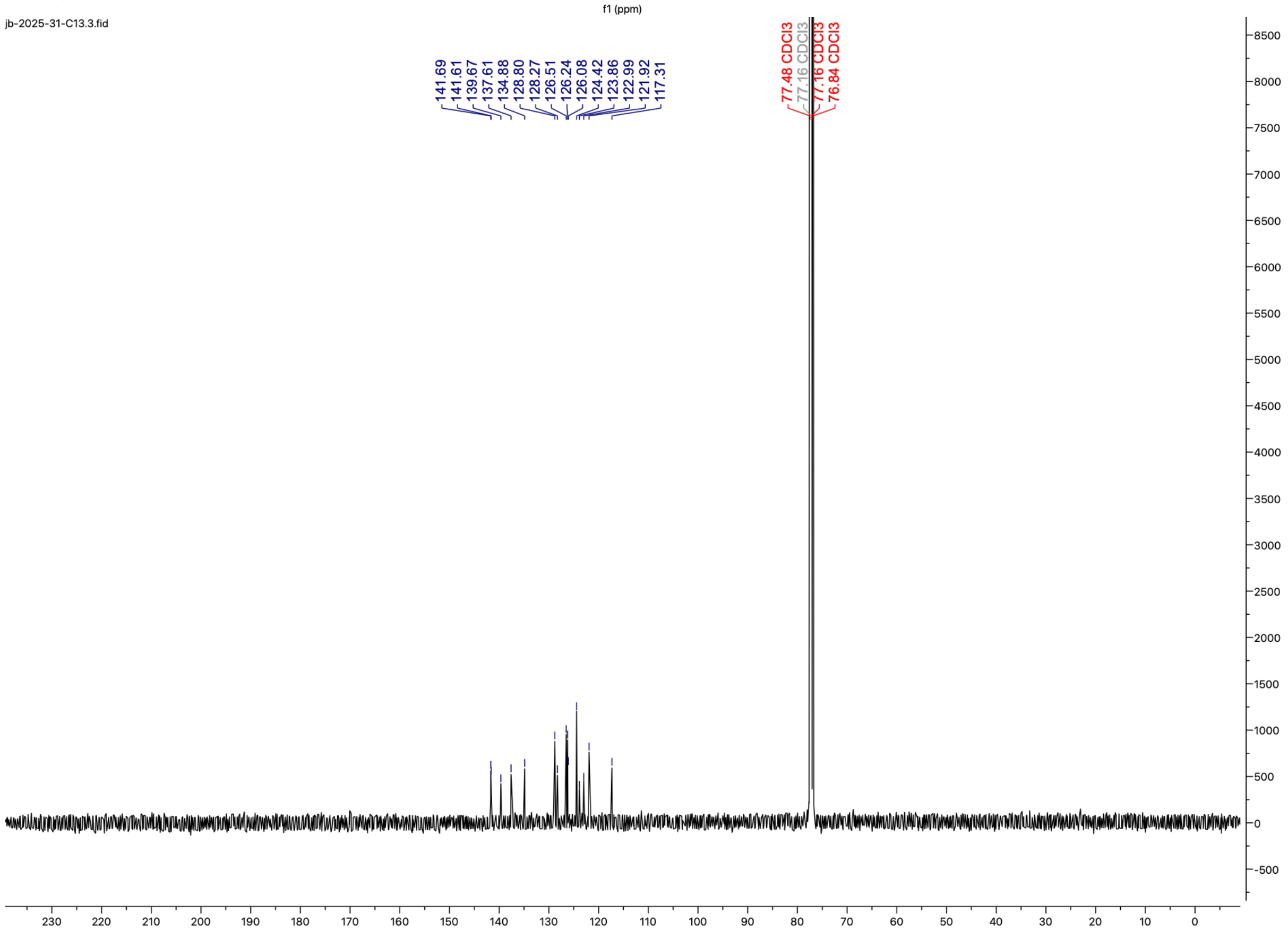
jb-2025-31-C13.3.fid
141.69
141.61
139.67
137.61
134.88
128.80
128.27
126.51
126.24
126.08
124.42
123.86
122.99
121.92
117.31
77.48 CDCl3
77.16 CDCl3
77.16 CDCl3
76.84 CDCl3
f1 (ppm)

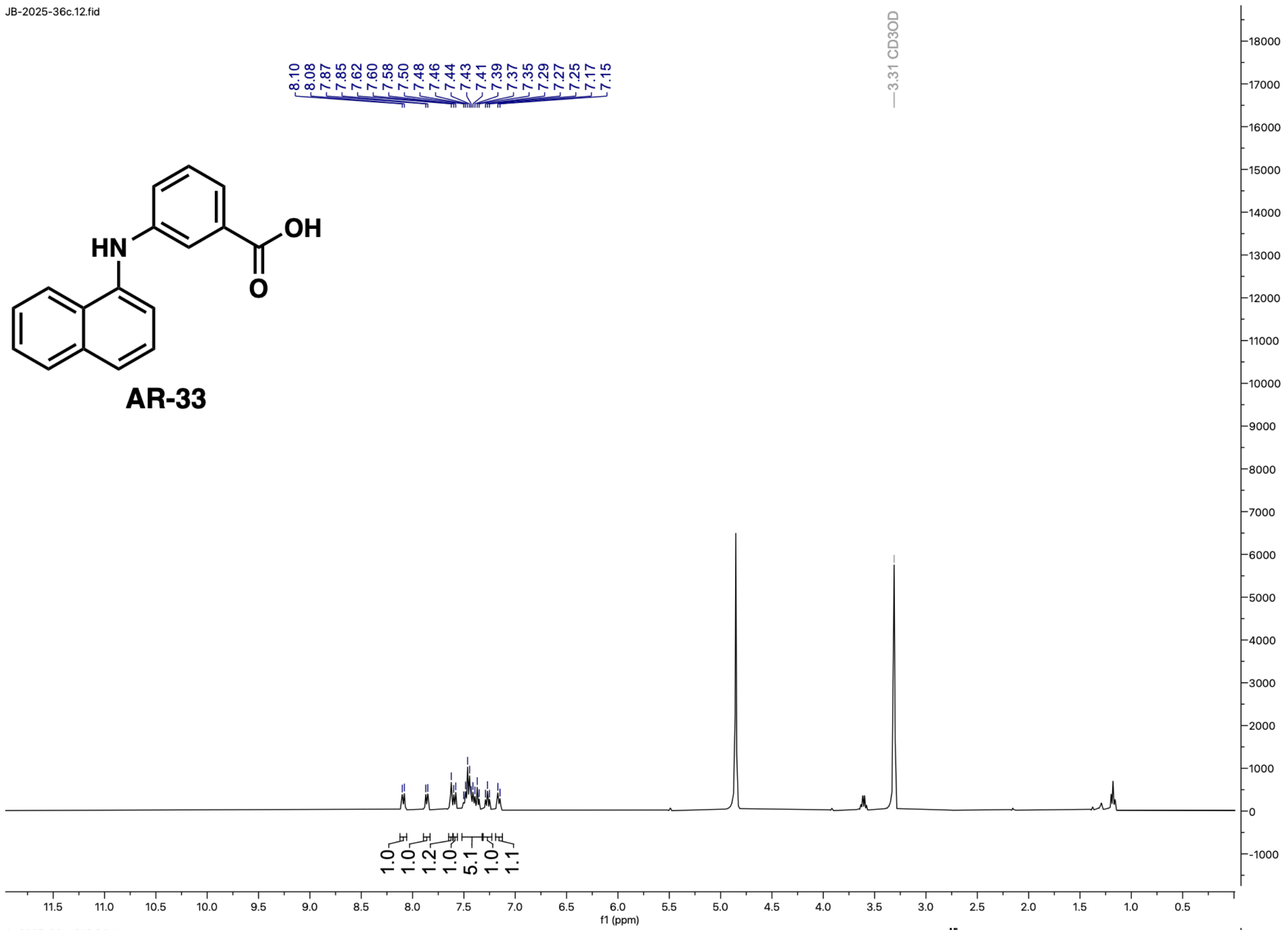
JB-2025-36c.12.fid
8.10 8.08 7.87 7.85 7.62 7.60 7.58 7.50 7.48 7.46 7.44 7.43 7.41 7.39 7.37 7.35 7.29 7.27 7.25 7.17 7.15
3.31 CD3OD
HN
OH
O
AR-33
1.0 1.0 1.2 1.0 5.1 1.0 1.1
f1 (ppm)

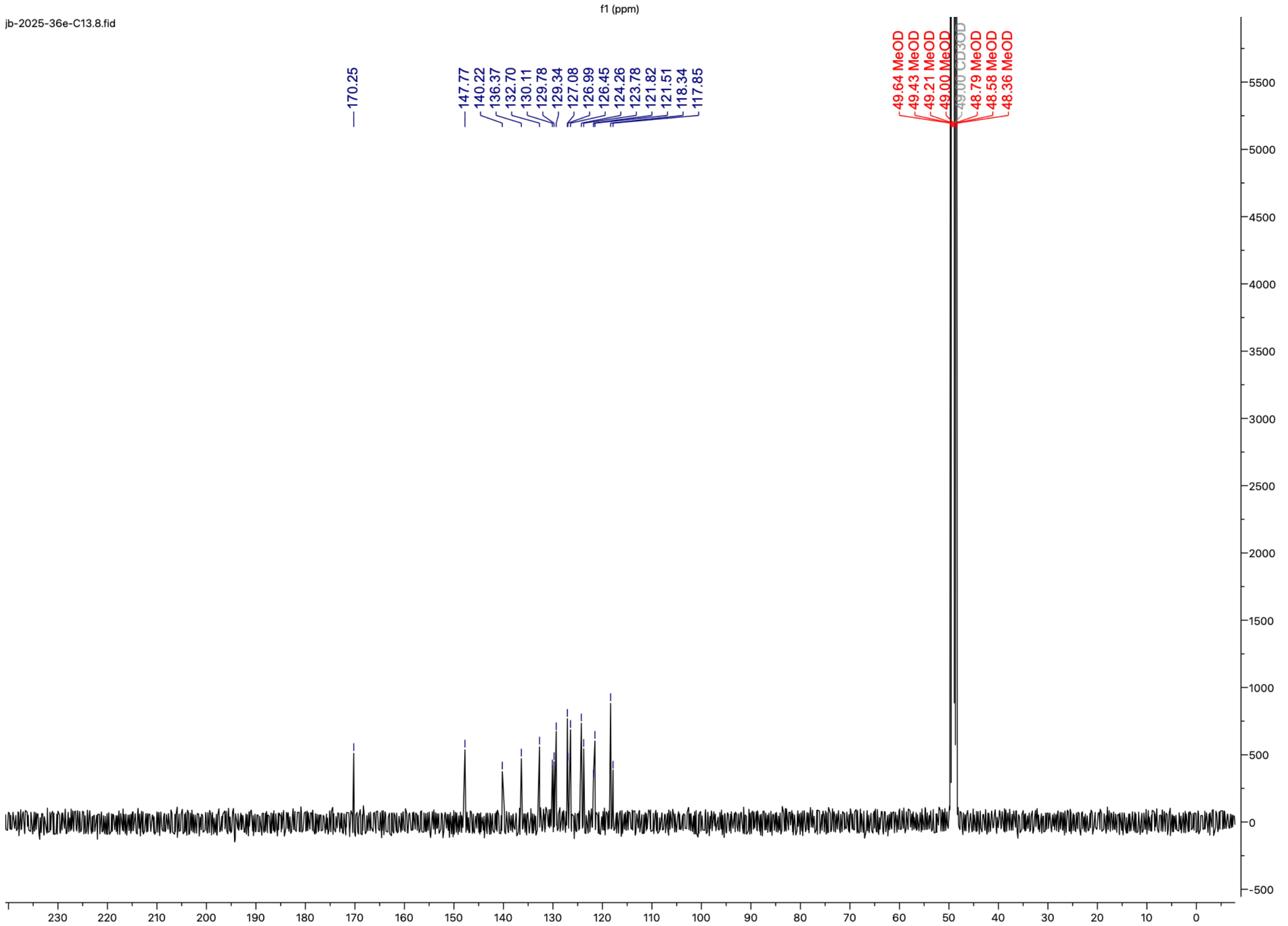
jb-2025-36e-C13.8.fid
170.25
147.77 140.22 136.37 132.70 130.11 129.78 129.34 127.08 126.99 126.45 124.26 123.78 121.82 121.51 118.34 117.85
49.64 MeOD 49.43 MeOD 49.21 MeOD 49.00 CD3OD 48.79 MeOD 48.58 MeOD 48.36 MeOD
f1 (ppm)

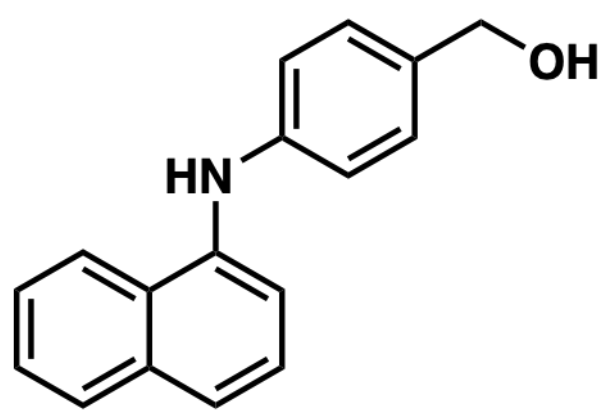

HN
OH

AR-34

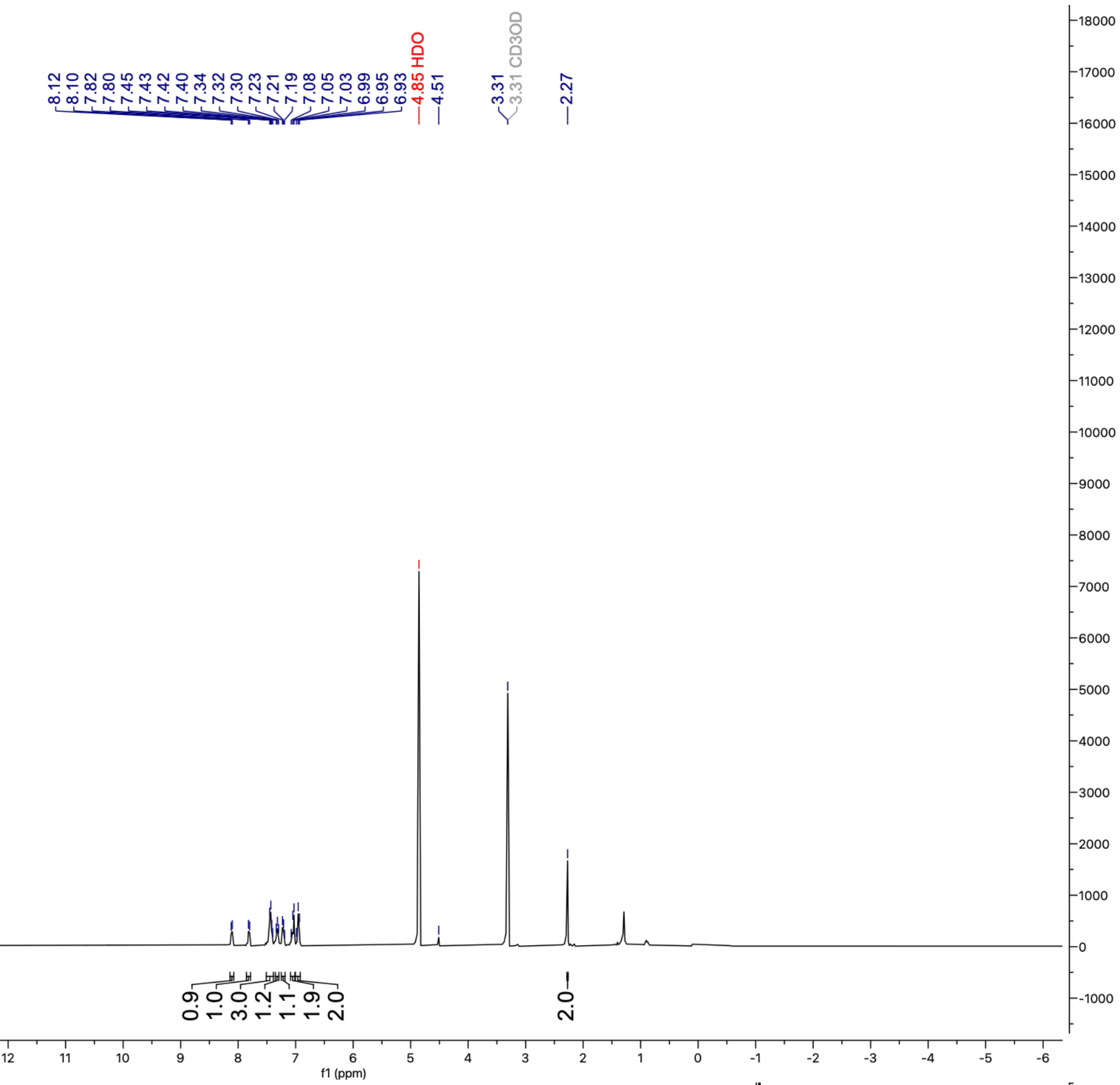

jb-2025-39b.1.fid
8.12
8.10
7.82
7.80
7.45
7.43
7.42
7.40
7.34
7.32
7.30
7.23
7.21
7.19
7.08
7.05
7.03
6.99
6.95
6.93
4.85 HDO
4.51
3.31
3.31 CD3OD
2.27
0.9
1.0
3.0
1.2
1.1
1.9
2.0
2.0
f1 (ppm)

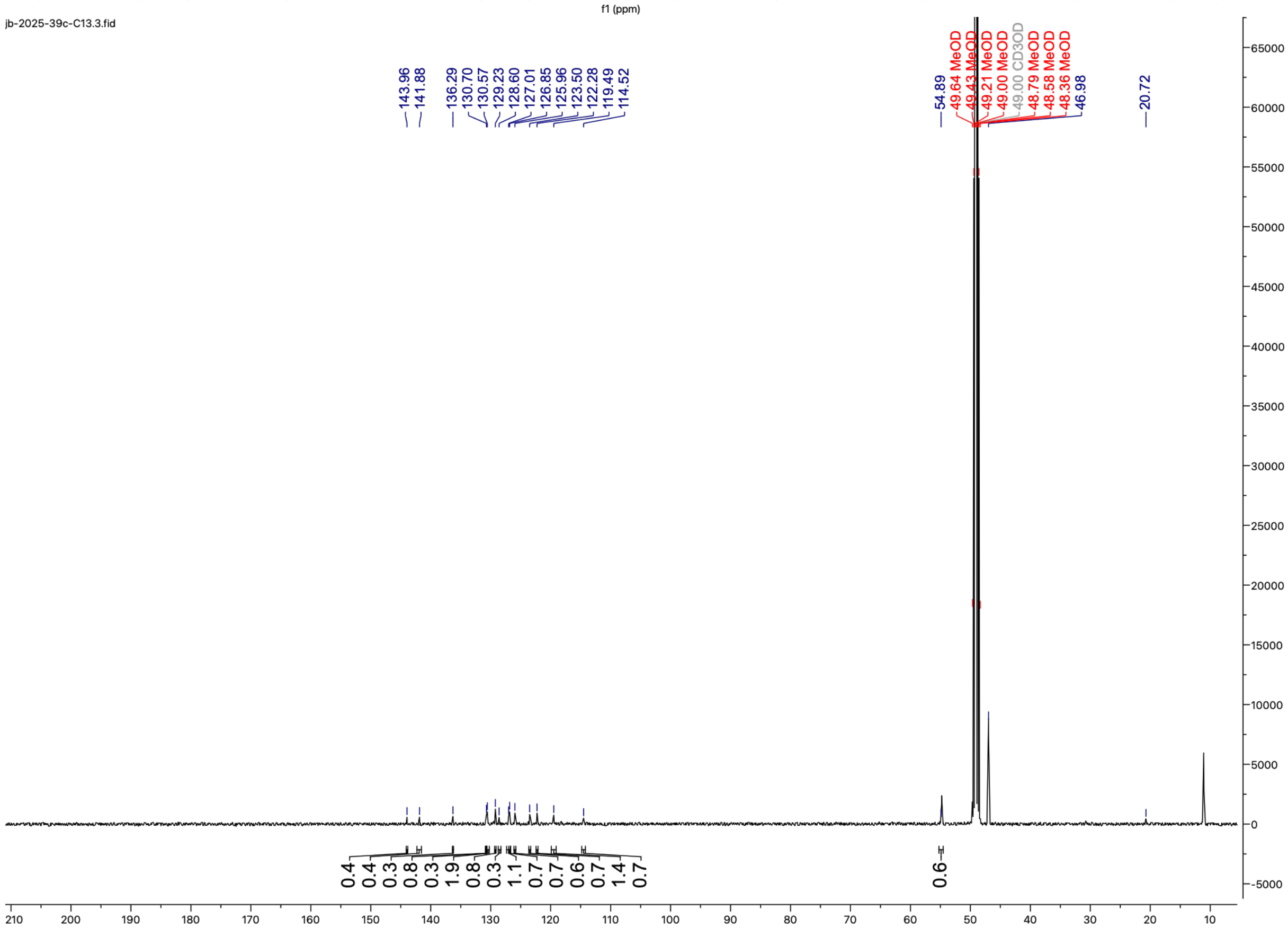

jb-2025-39c-C13.3.fid
143.96
141.88
136.29
130.70
130.57
129.23
128.60
127.01
126.85
125.96
123.50
122.28
119.49
114.52
54.89
49.64 MeOD
49.43 MeOD
49.21 MeOD
49.00 CD3OD
48.79 MeOD
48.58 MeOD
48.36 MeOD
46.98
20.72
0.4
0.4
0.3
0.8
0.3
1.9
0.8
0.3
1.1
0.7
0.7
0.6
0.7
1.4
0.7
0.6
f1 (ppm)

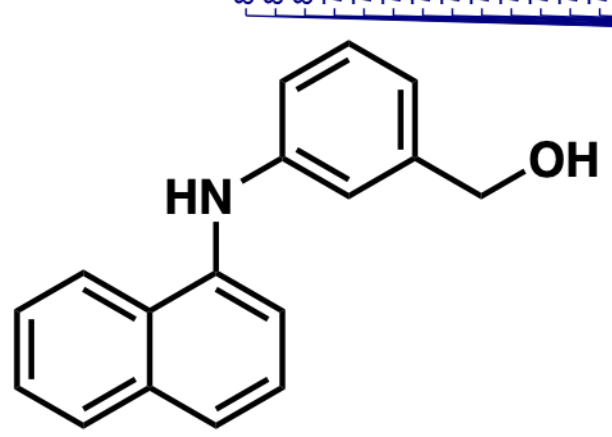

AR-35

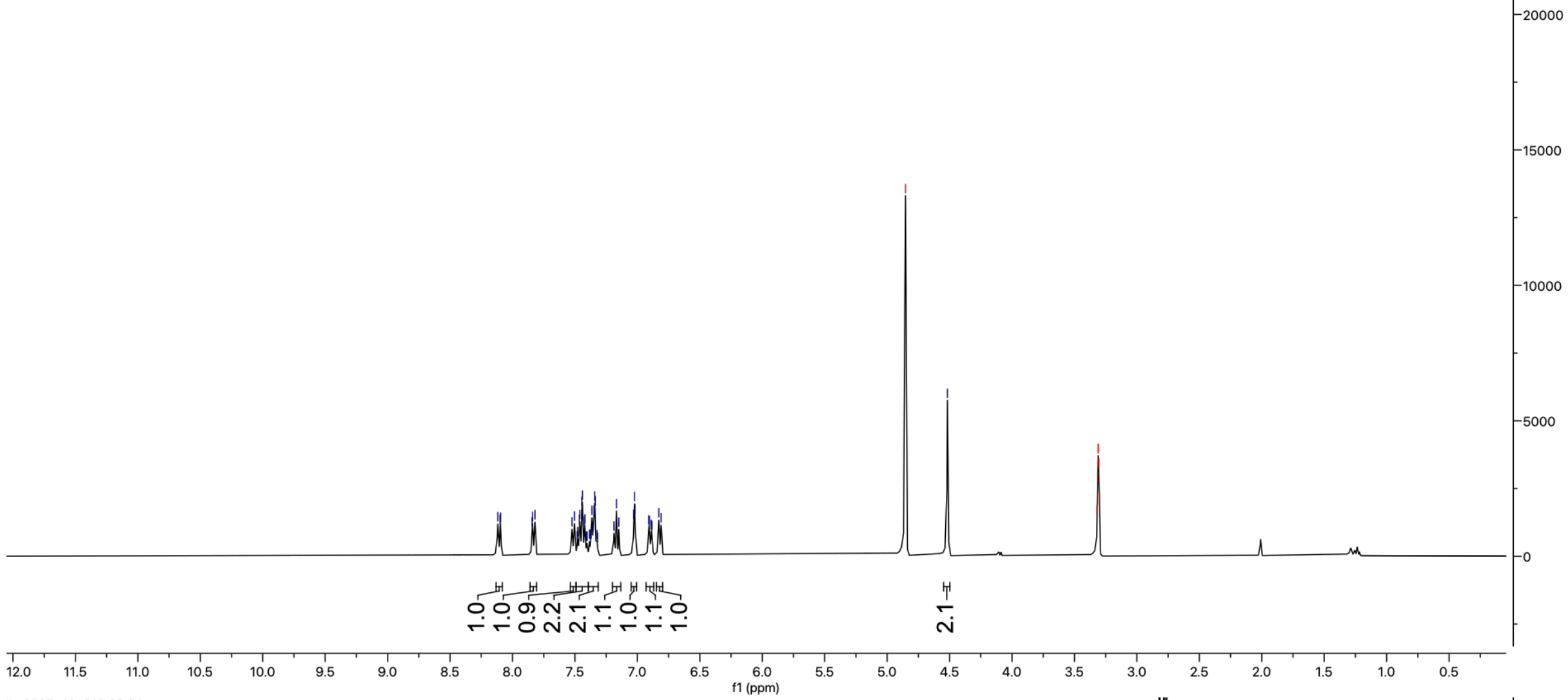

jb-2025-40.24.fid
8.12 8.10 8.10 7.84 7.84 7.82 7.52 7.50 7.48 7.48 7.46 7.46 7.44 7.44 7.42 7.42 7.41 7.40 7.38 7.37 7.36 7.35 7.35 7.34 7.34 7.32 7.32 7.19 7.17 7.15 7.03 7.02 7.02 6.91 6.90 6.89 6.88 6.83 6.81
4.85 HDO
4.52
3.32 MeOD
3.31 MeOD
3.31 MeOD
3.31 CD3OD
3.31 MeOD
3.30 MeOD
1.0 1.0 0.9 2.2 2.1 1.1 1.0 1.1 1.0
2.1
f1 (ppm)

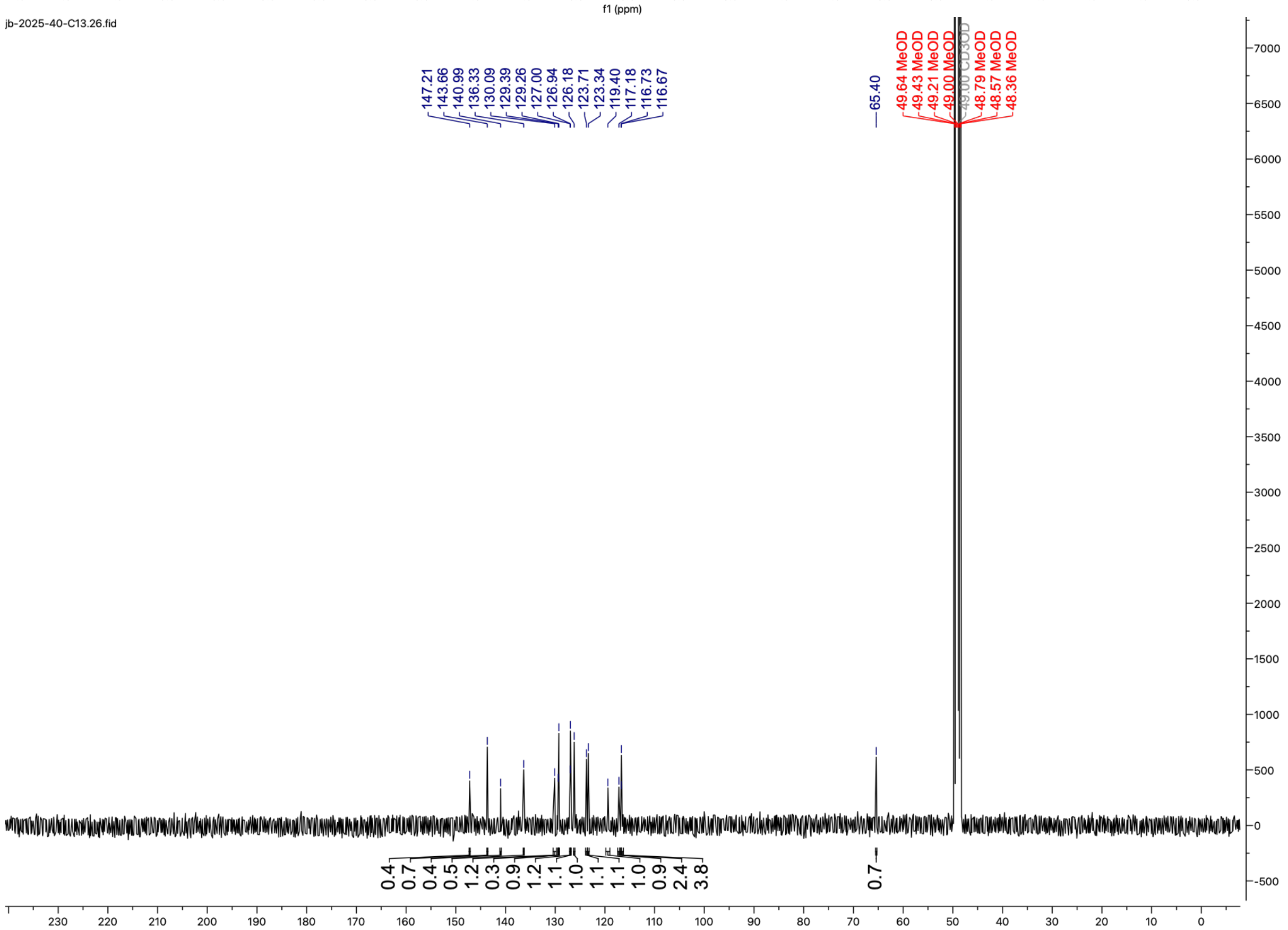

jb-2025-40-C13.26.fid
147.21 143.66 140.99 136.33 130.09 129.39 129.26 127.00 126.94 126.18 123.71 123.34 119.40 117.18 116.73 116.67
65.40
49.64 MeOD
49.43 MeOD
49.21 MeOD
49.00 CD3OD
48.79 MeOD
48.57 MeOD
48.36 MeOD
0.4 0.7 0.4 0.5 1.2 0.3 0.9 1.2 1.1 1.0 1.1 1.1 1.0 0.9 2.4 3.8
0.7
f1 (ppm)

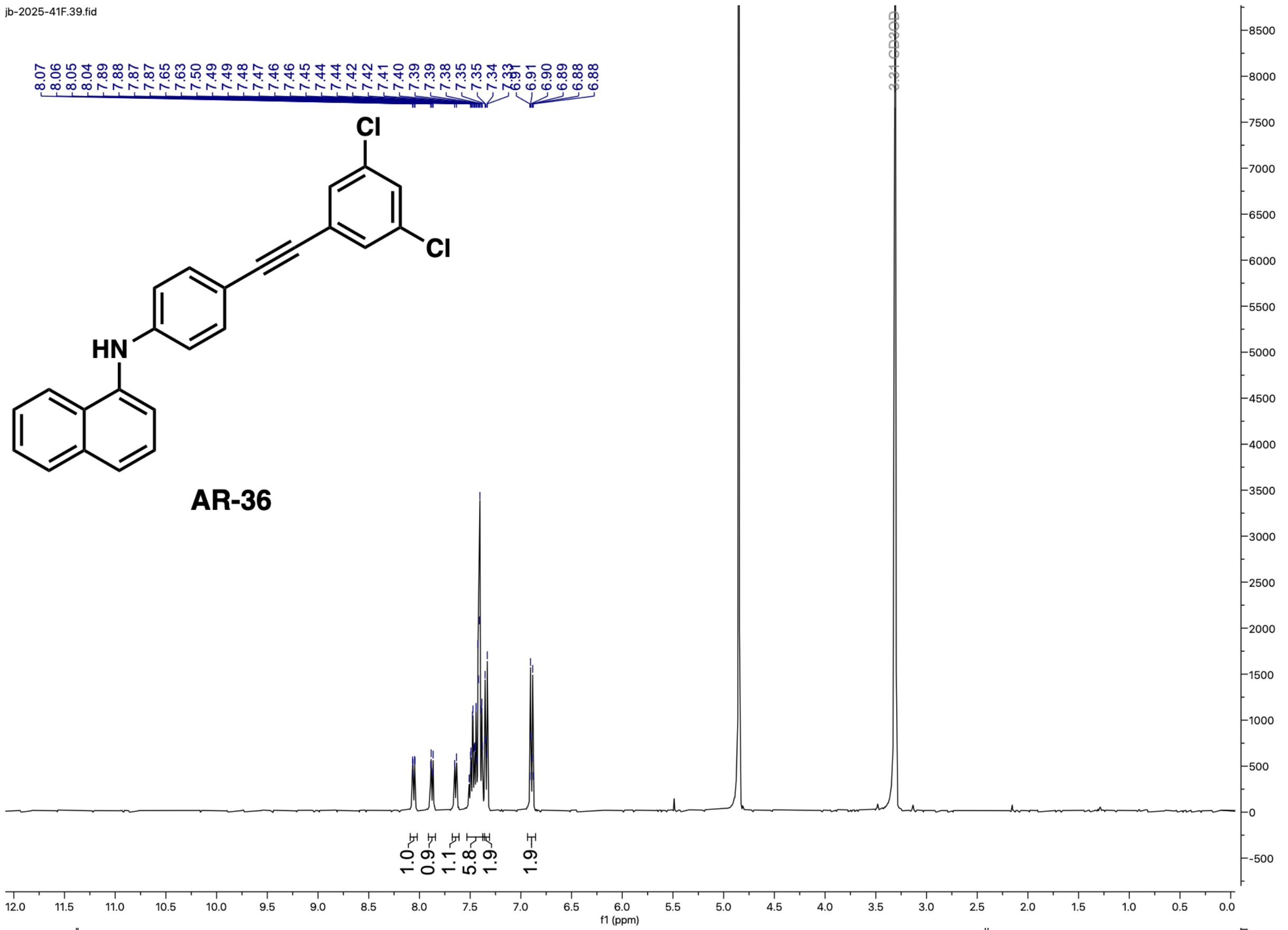
jb-2025-41F.39.fid
8.07 8.06 8.05 8.04 7.89 7.88 7.87 7.87 7.65 7.63 7.50 7.49 7.49 7.48 7.47 7.46 7.45 7.44 7.44 7.42 7.42 7.41 7.40 7.39 7.39 7.39 7.38 7.35 7.35 7.34 7.33 6.91 6.91 6.90 6.89 6.88 6.88
3.31 CD3OD
Cl
Cl
HN
AR-36
1.0 0.9 1.1 5.8 1.9 1.9
f1 (ppm)

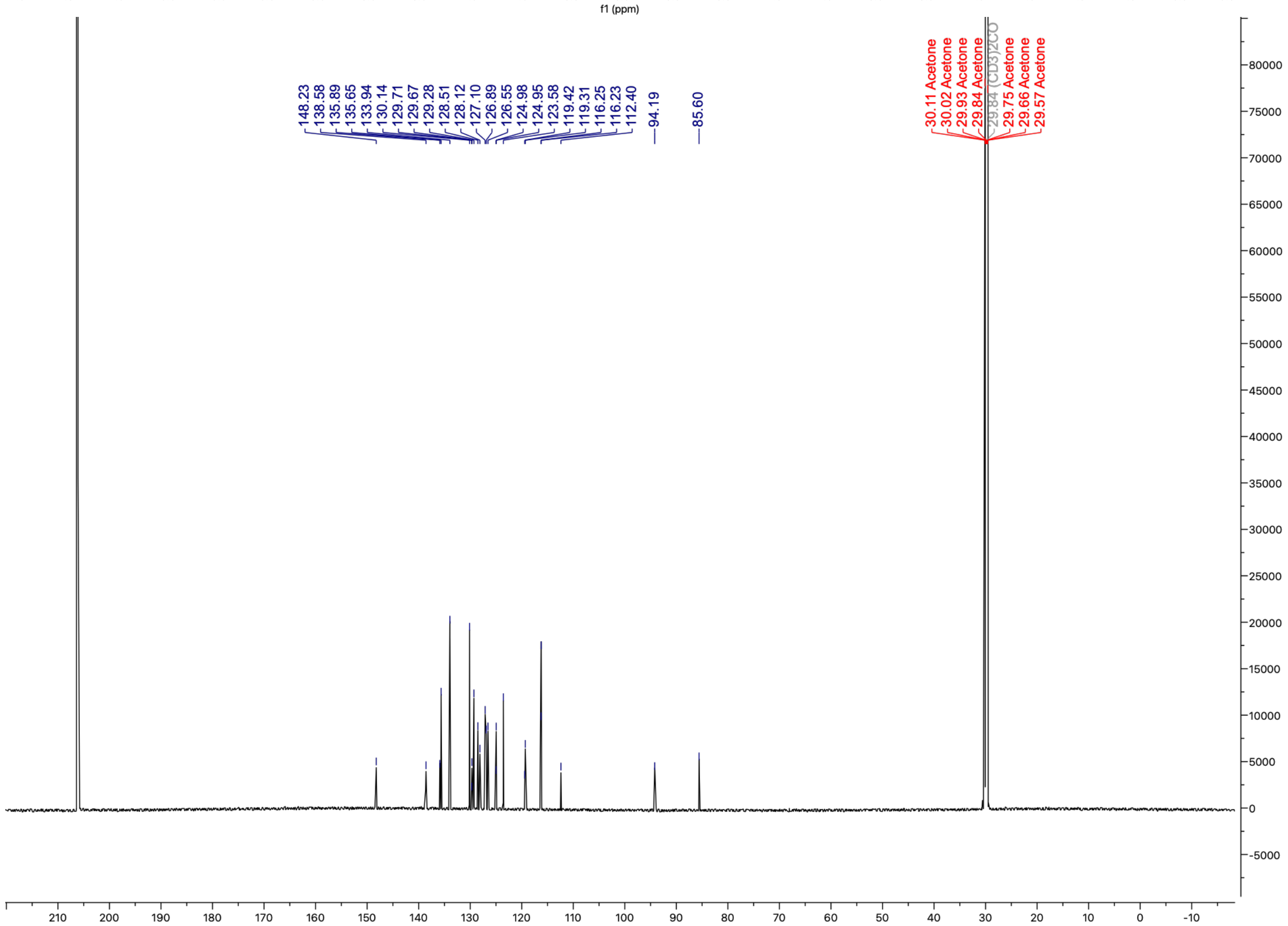
148.23 138.58 135.89 135.65 133.94 130.14 129.71 129.67 129.28 128.51 128.12 127.10 126.89 126.55 124.98 124.95 123.58 119.42 119.31 116.25 116.23 112.40
94.19
85.60
30.11 Acetone
30.02 Acetone
29.93 Acetone
29.84 Acetone
29.84 (CD3)2CO
29.75 Acetone
29.66 Acetone
29.57 Acetone
f1 (ppm)

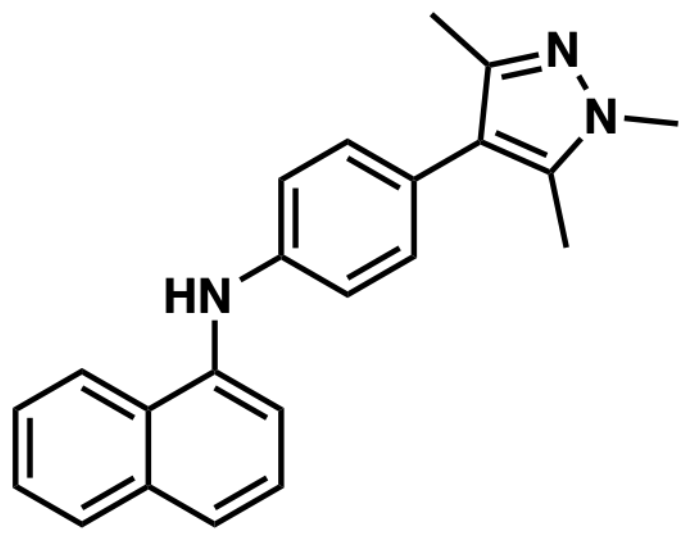

N
N
HN

AR-37

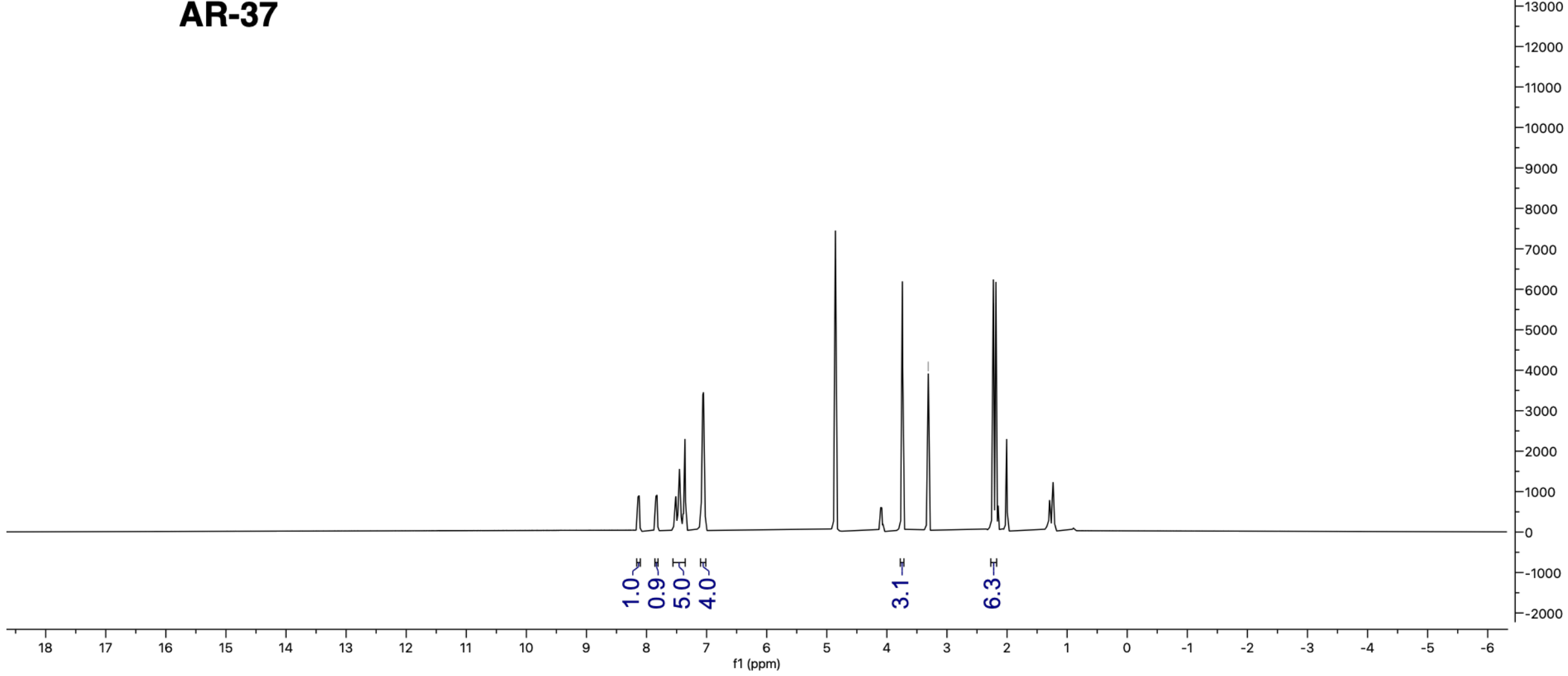

3.31 CD3OD
1.0
0.9
5.0
4.0
3.1
6.3
f1 (ppm)

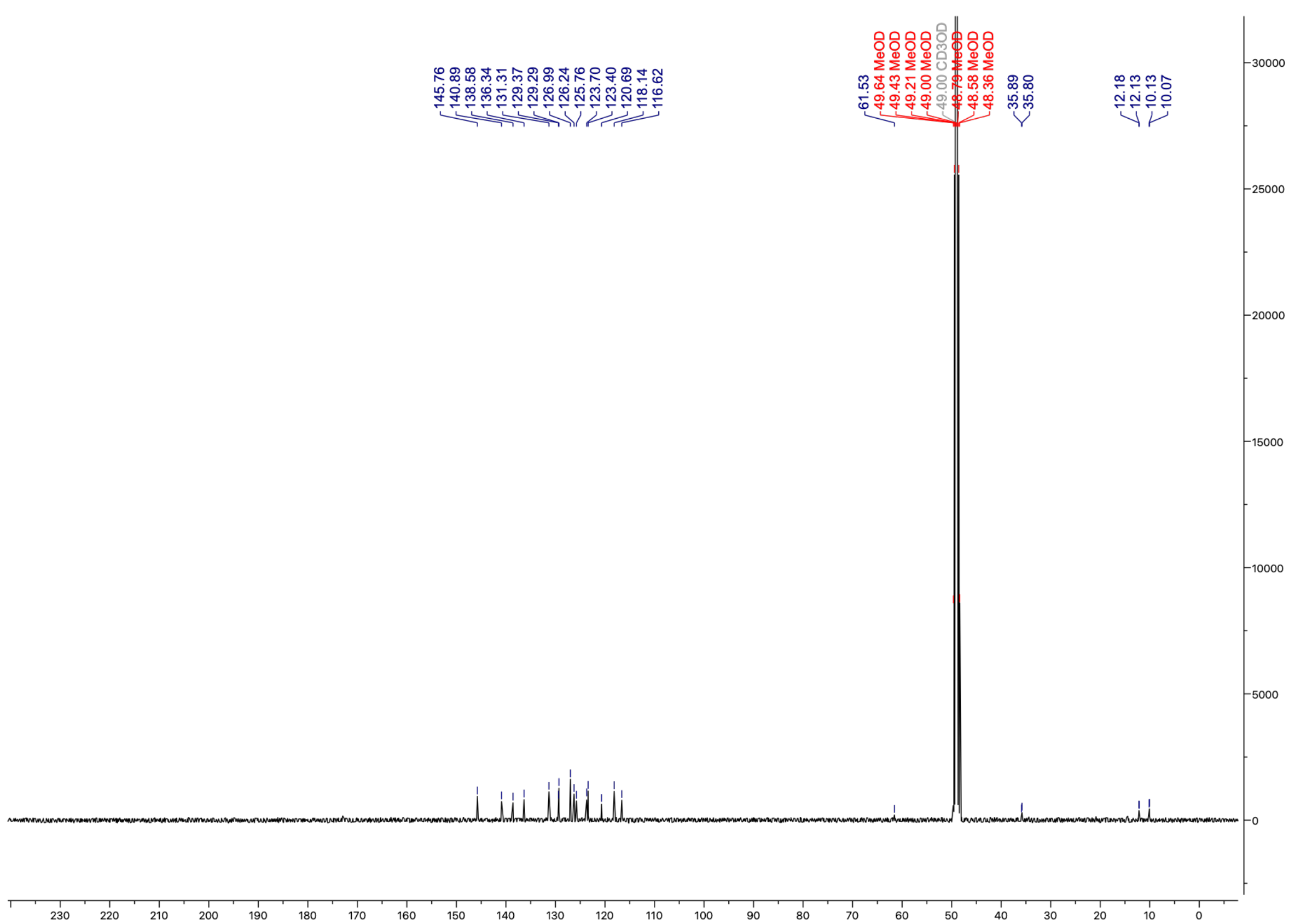

145.76
140.89
138.58
136.34
131.31
129.37
129.29
126.99
126.24
125.76
123.70
123.40
120.69
118.14
116.62
61.53
49.64 MeOD
49.43 MeOD
49.21 MeOD
49.00 CD3OD
48.79 MeOD
48.58 MeOD
48.36 MeOD
35.89
35.80
12.18
12.13
10.13
10.07
f1 (ppm)

AR-38

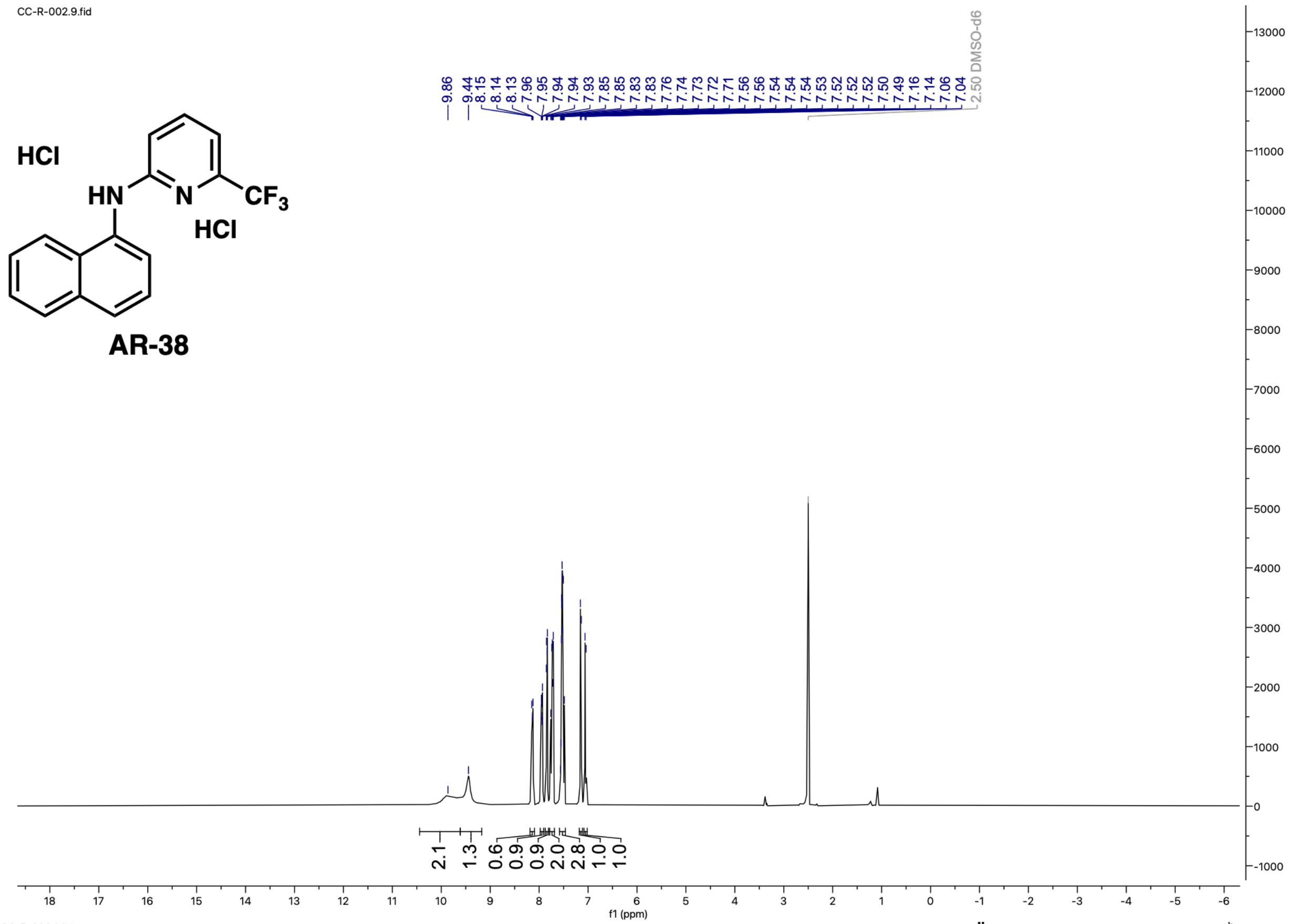
CC-R-002.9.fid
9.86
9.44
8.15
8.14
8.13
7.96
7.95
7.94
7.94
7.93
7.85
7.85
7.83
7.83
7.76
7.74
7.73
7.72
7.71
7.56
7.56
7.54
7.54
7.54
7.53
7.52
7.52
7.50
7.49
7.16
7.14
7.06
7.04
2.50 DMSO-d6
2.1
1.3
0.6
0.9
0.9
2.0
2.8
1.0
1.0
f1 (ppm)

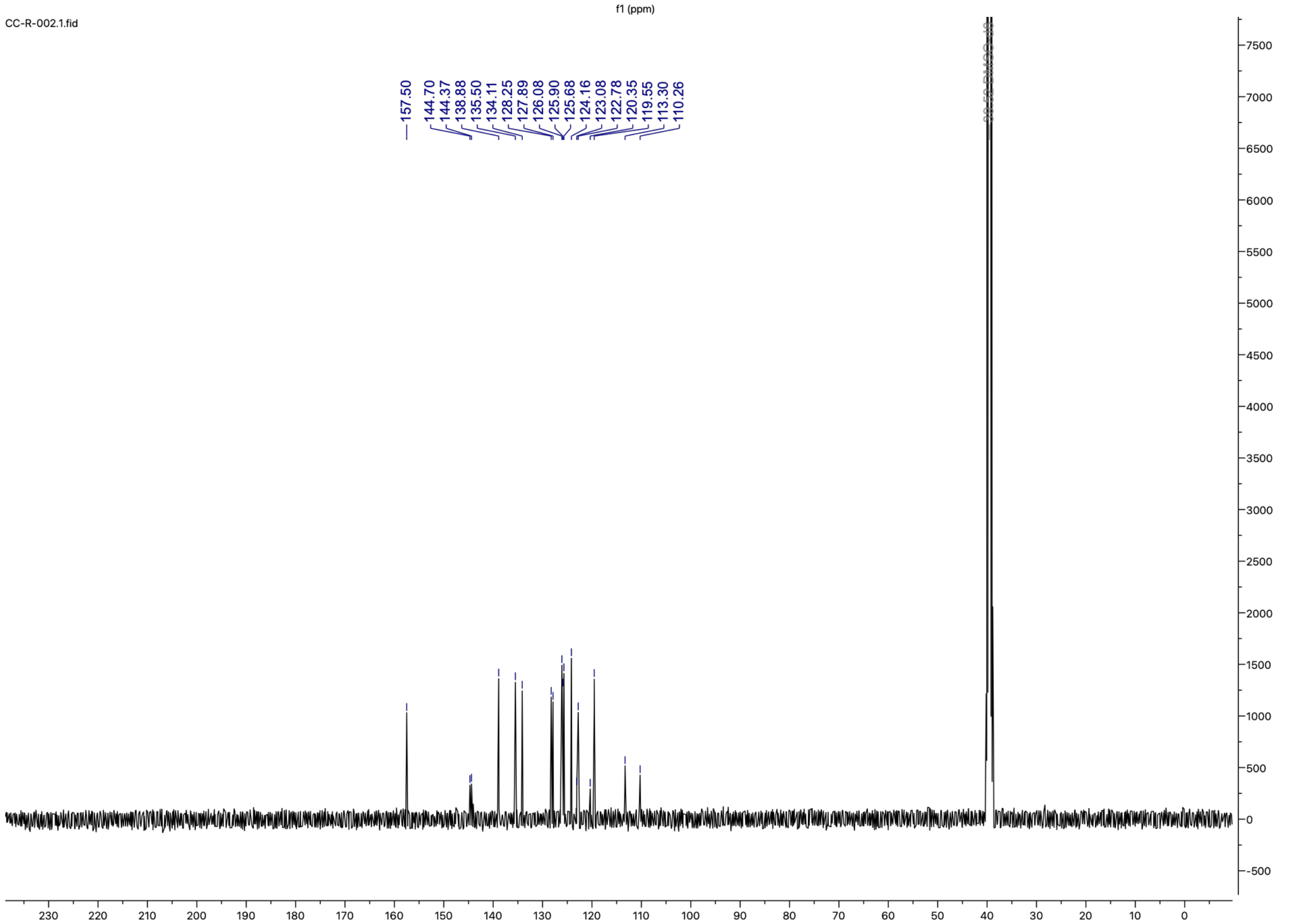
CC-R-002.1.fid
157.50
144.70
144.37
138.88
135.50
134.11
128.25
127.89
126.08
125.90
125.68
124.16
123.08
122.78
120.35
119.55
113.30
110.26
f1 (ppm)

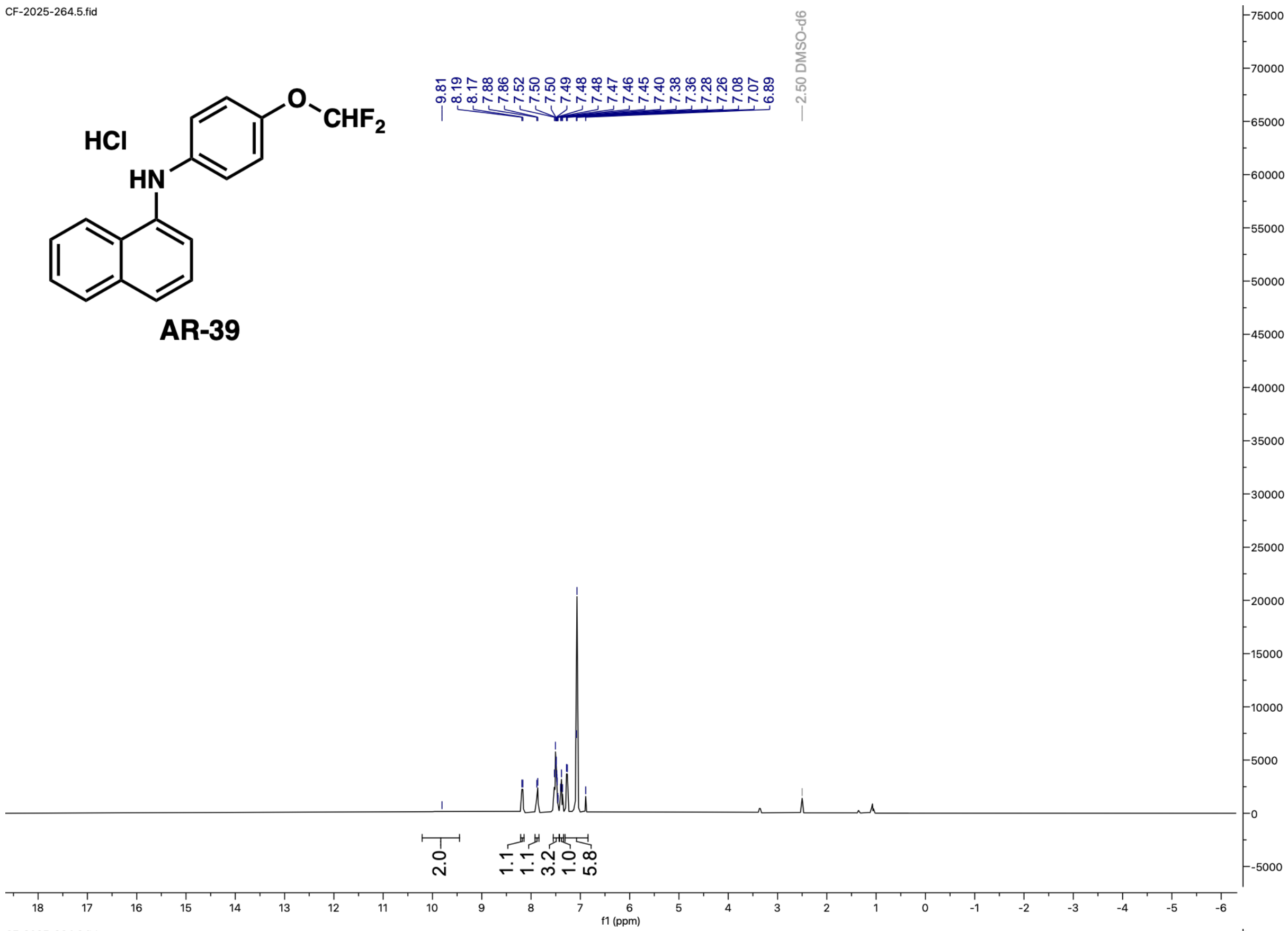
CF-2025-264.5.fid
HCl
HN
O
CHF2
AR-39
9.81
8.19
8.17
7.88
7.86
7.52
7.50
7.50
7.49
7.48
7.48
7.47
7.46
7.45
7.40
7.38
7.36
7.28
7.26
7.08
7.07
6.89
2.50 DMSO-d6
2.0
1.1
1.1
3.2
1.0
5.8
f1 (ppm)

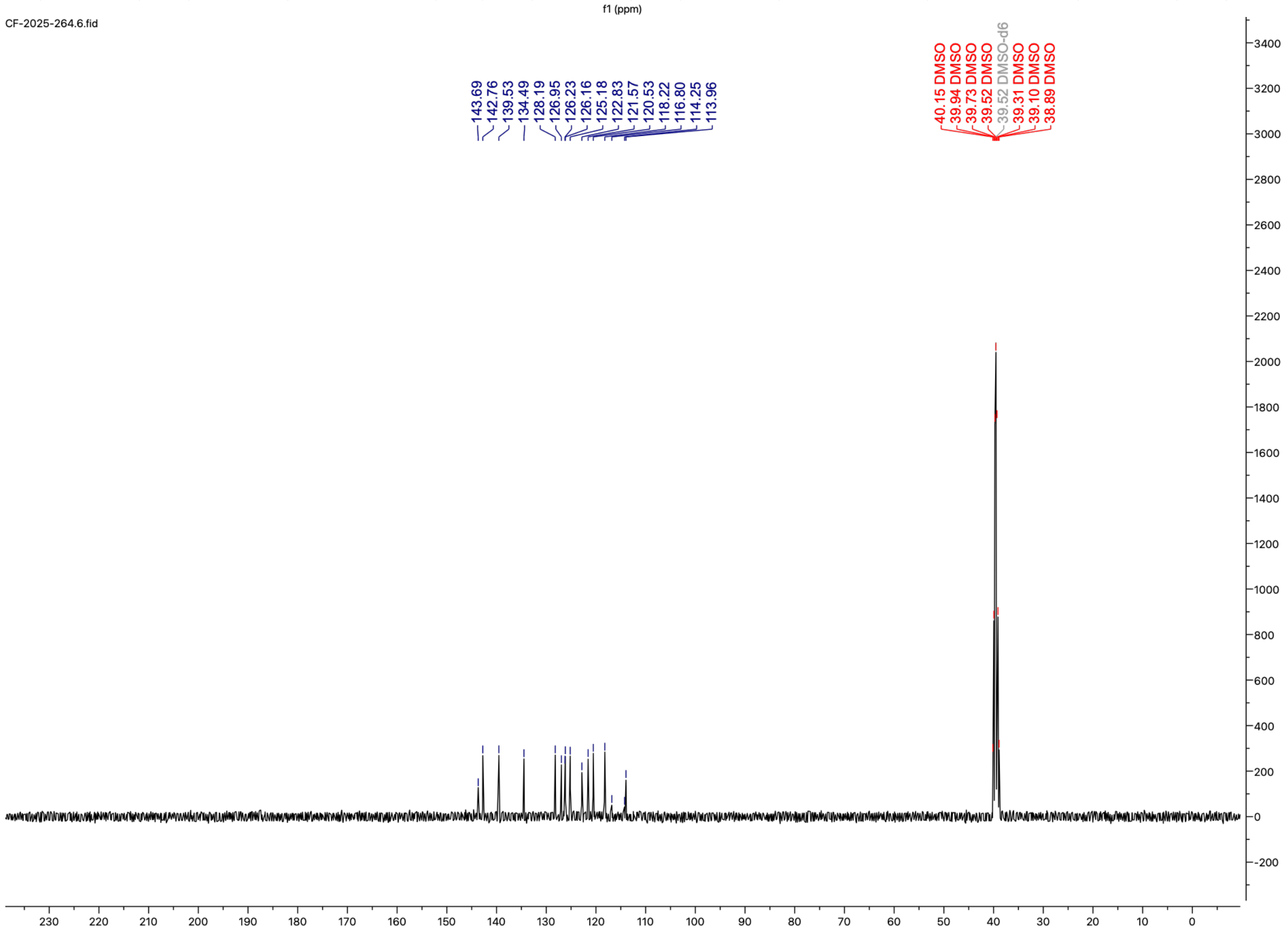
CF-2025-264.6.fid
143.69
142.76
139.53
134.49
128.19
126.95
126.23
126.16
125.18
122.83
121.57
120.53
118.22
116.80
114.25
113.96
40.15 DMSO
39.94 DMSO
39.73 DMSO
39.52 DMSO-d6
39.31 DMSO
39.10 DMSO
38.89 DMSO
f1 (ppm)

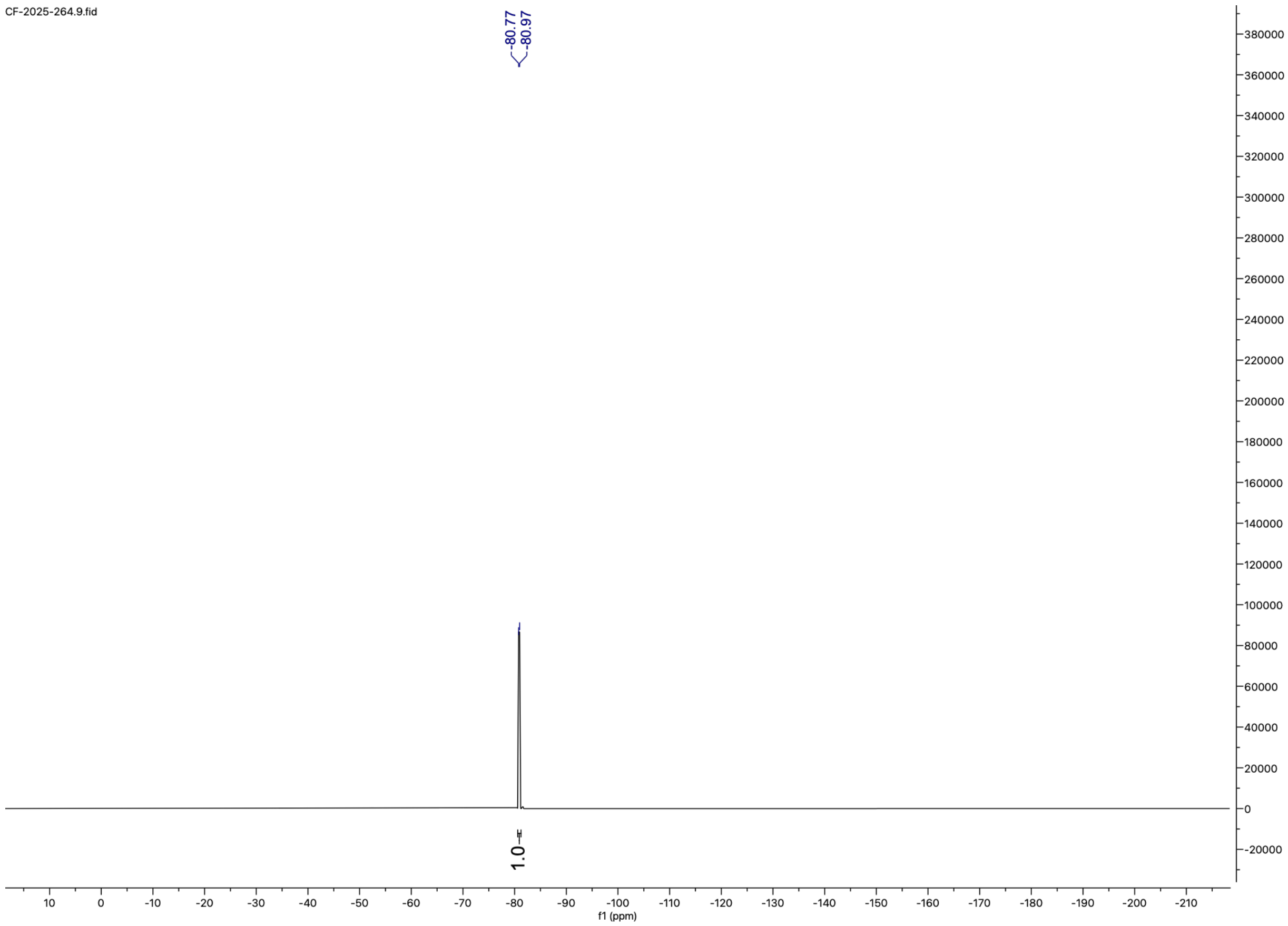
CF-2025-264.9.fid
-80.77
-80.97
1.0
f1 (ppm)

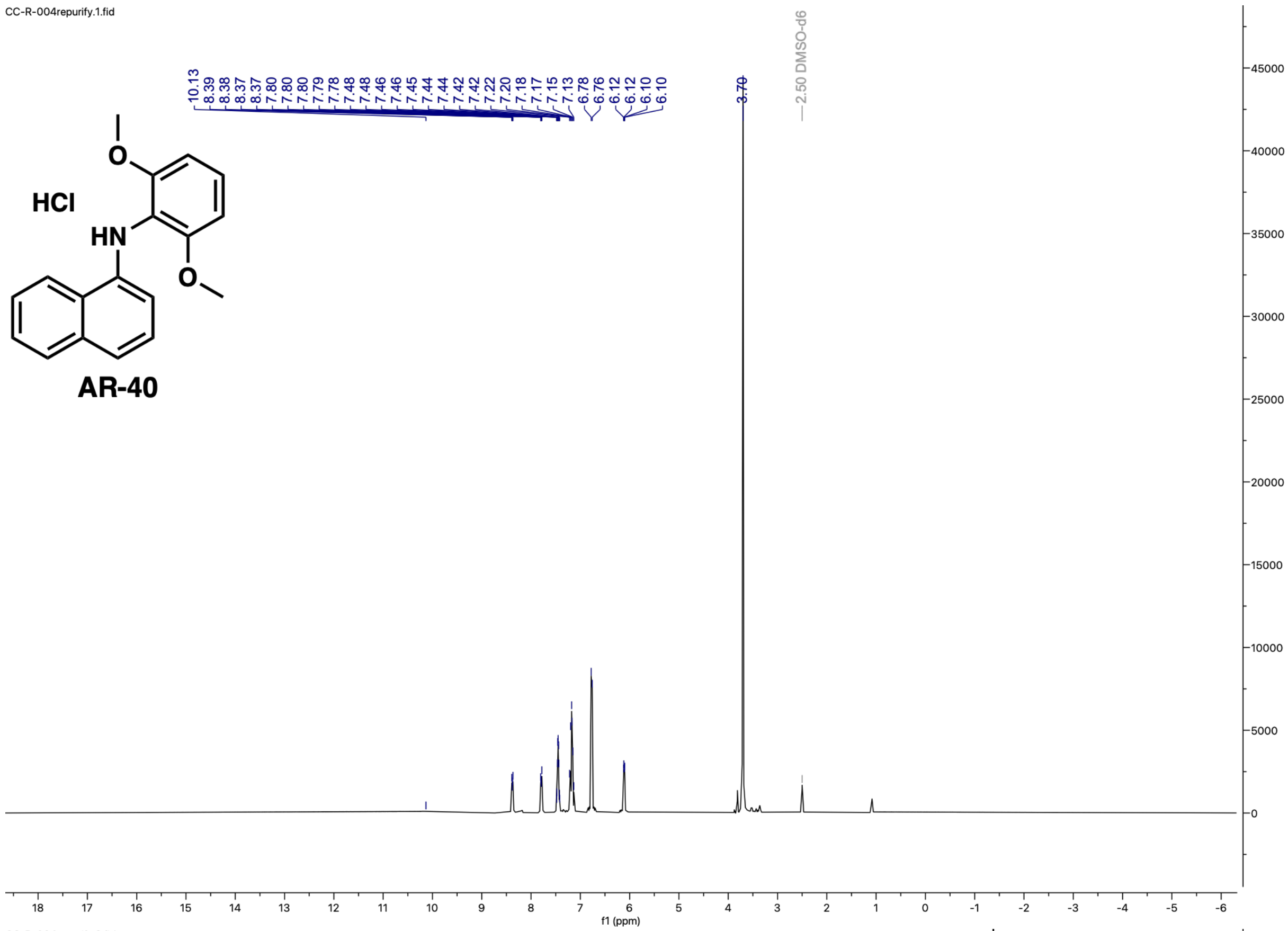

HCl
HN
AR-40
CC-R-004repurify.1.fid
10.13
8.39
8.38
8.37
8.37
7.80
7.80
7.80
7.79
7.78
7.48
7.48
7.46
7.46
7.45
7.44
7.44
7.42
7.42
7.22
7.20
7.18
7.17
7.15
7.13
6.78
6.76
6.12
6.10
6.10
3.70
2.50 DMSO-d6
f1 (ppm)

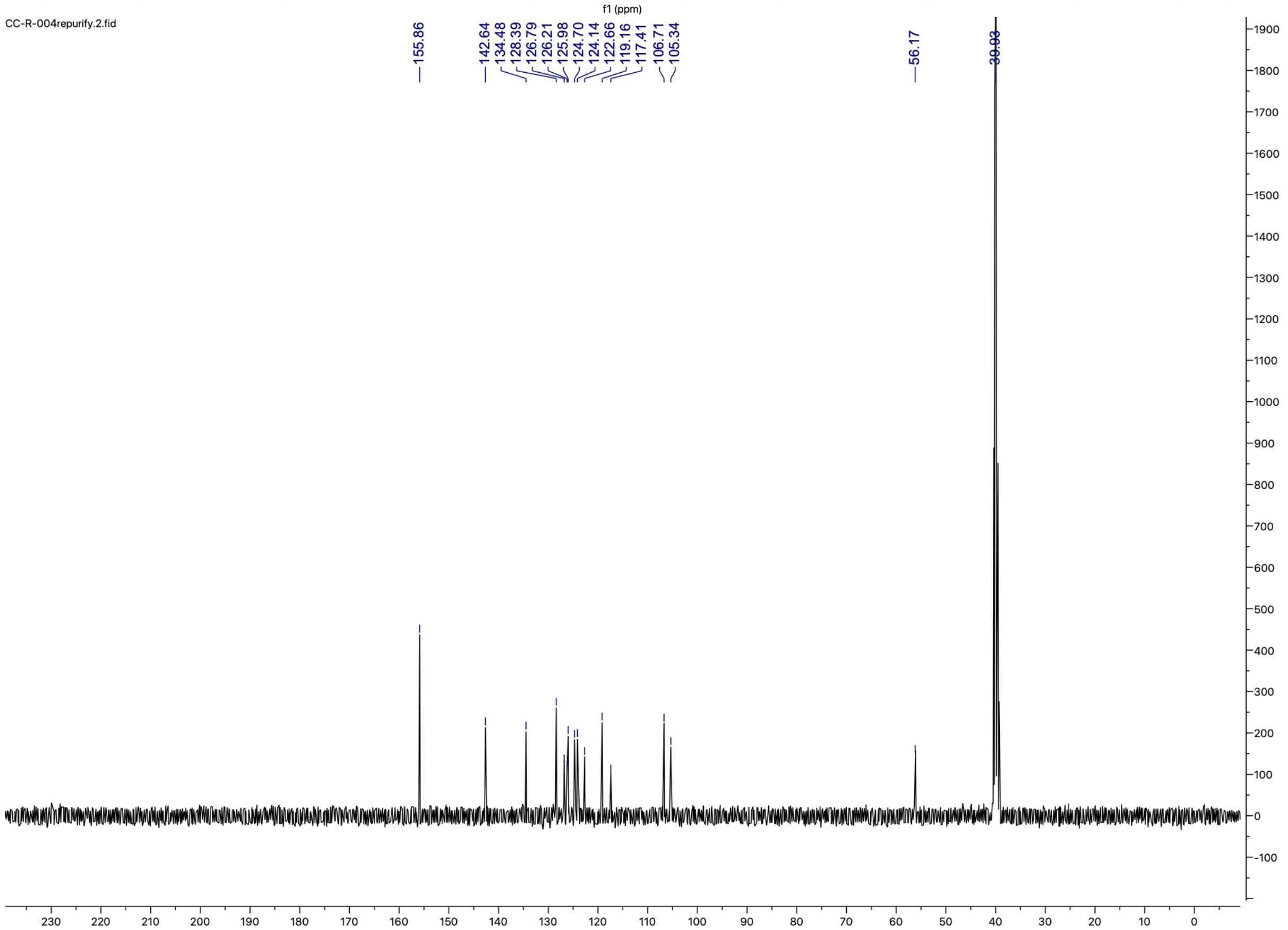

CC-R-004repurify.2.fid
155.86
142.64
134.48
128.39
126.79
126.21
125.98
124.70
124.14
122.66
119.16
117.41
106.71
105.34
56.17
39.93
f1 (ppm)

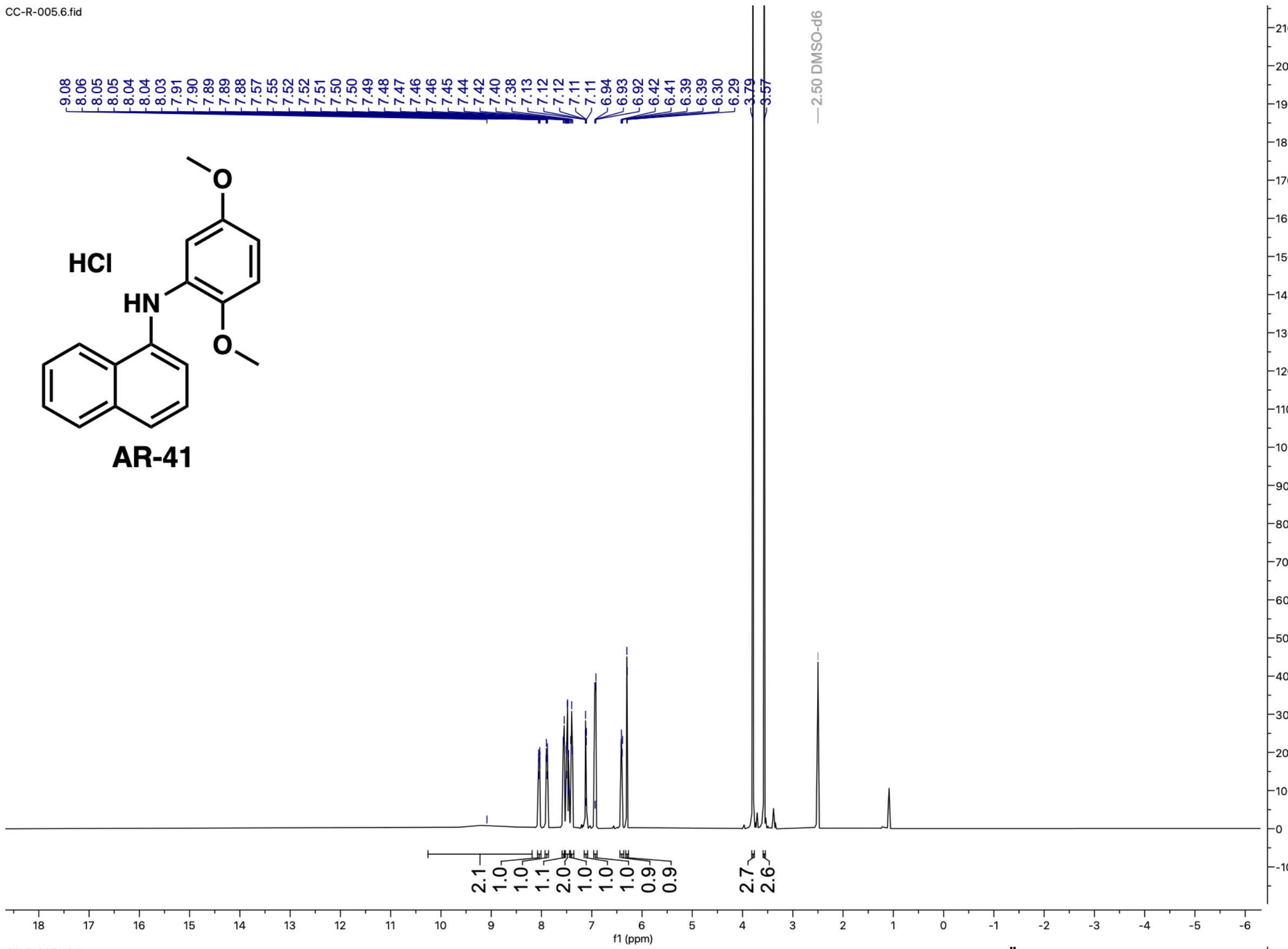
CC-R-005.6.fid
9.08 8.06 8.05 8.05 8.04 8.04 8.03 7.91 7.90 7.89 7.89 7.88 7.57 7.55 7.52 7.52 7.51 7.50 7.50 7.49 7.48 7.47 7.46 7.46 7.45 7.44 7.42 7.40 7.38 7.13 7.12 7.12 7.11 7.11 6.94 6.93 6.92 6.42 6.41 6.39 6.39 6.30 6.29 3.79 3.57
2.50 DMSO-d6
HCl
HN
O
AR-41
2.1 1.0 1.0 1.1 2.0 1.0 1.0 1.0 0.9 0.9 2.7 2.6
f1 (ppm)

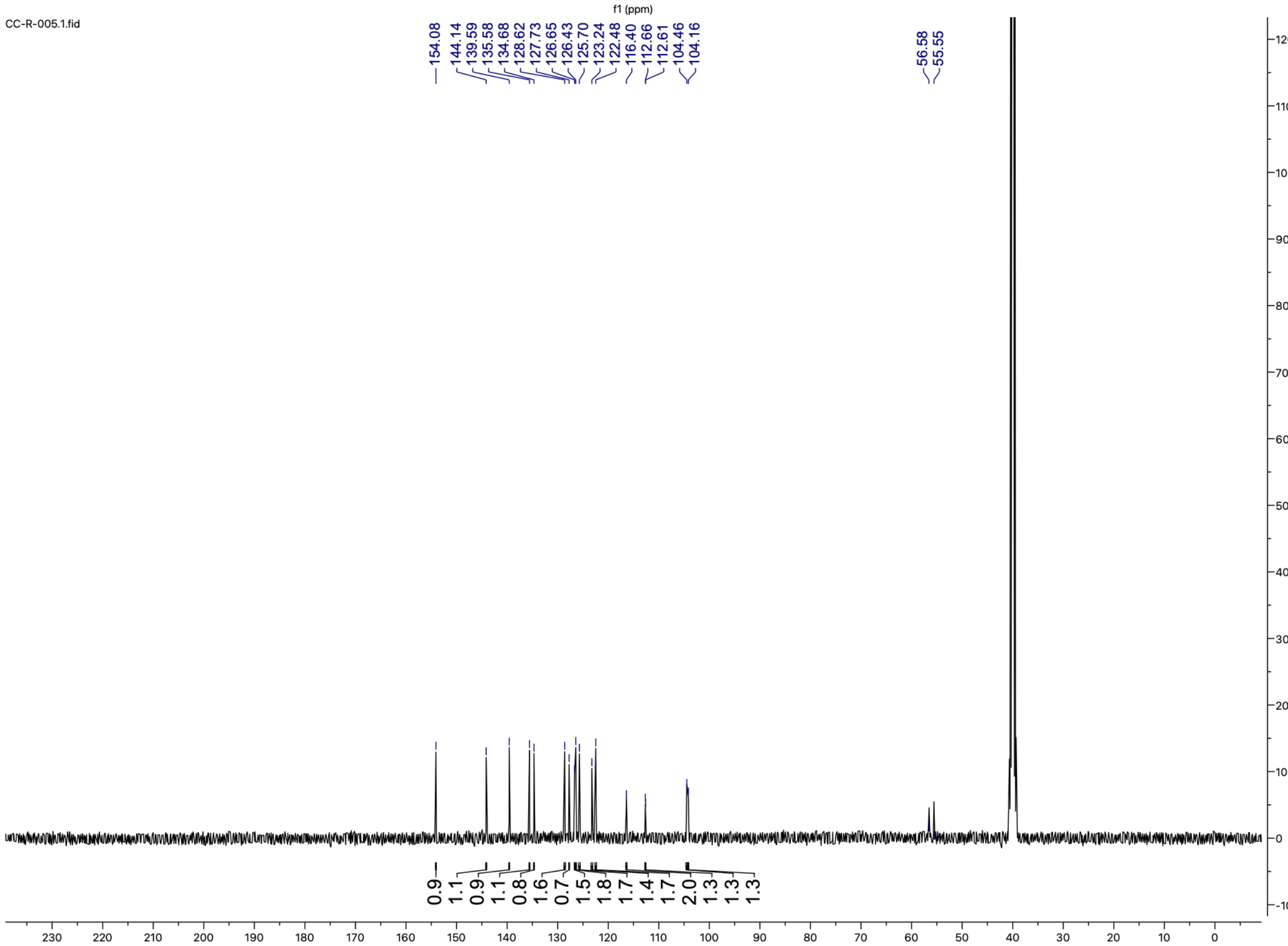
CC-R-005.1.fid
154.08 144.14 139.59 135.58 134.68 128.62 127.73 126.65 126.43 125.70 123.24 122.48 116.40 112.66 112.61 104.46 104.16
56.58 55.55
0.9 1.1 0.9 1.1 0.8 1.6 0.7 1.5 1.8 1.7 1.4 1.7 2.0 1.3 1.3 1.3
f1 (ppm)

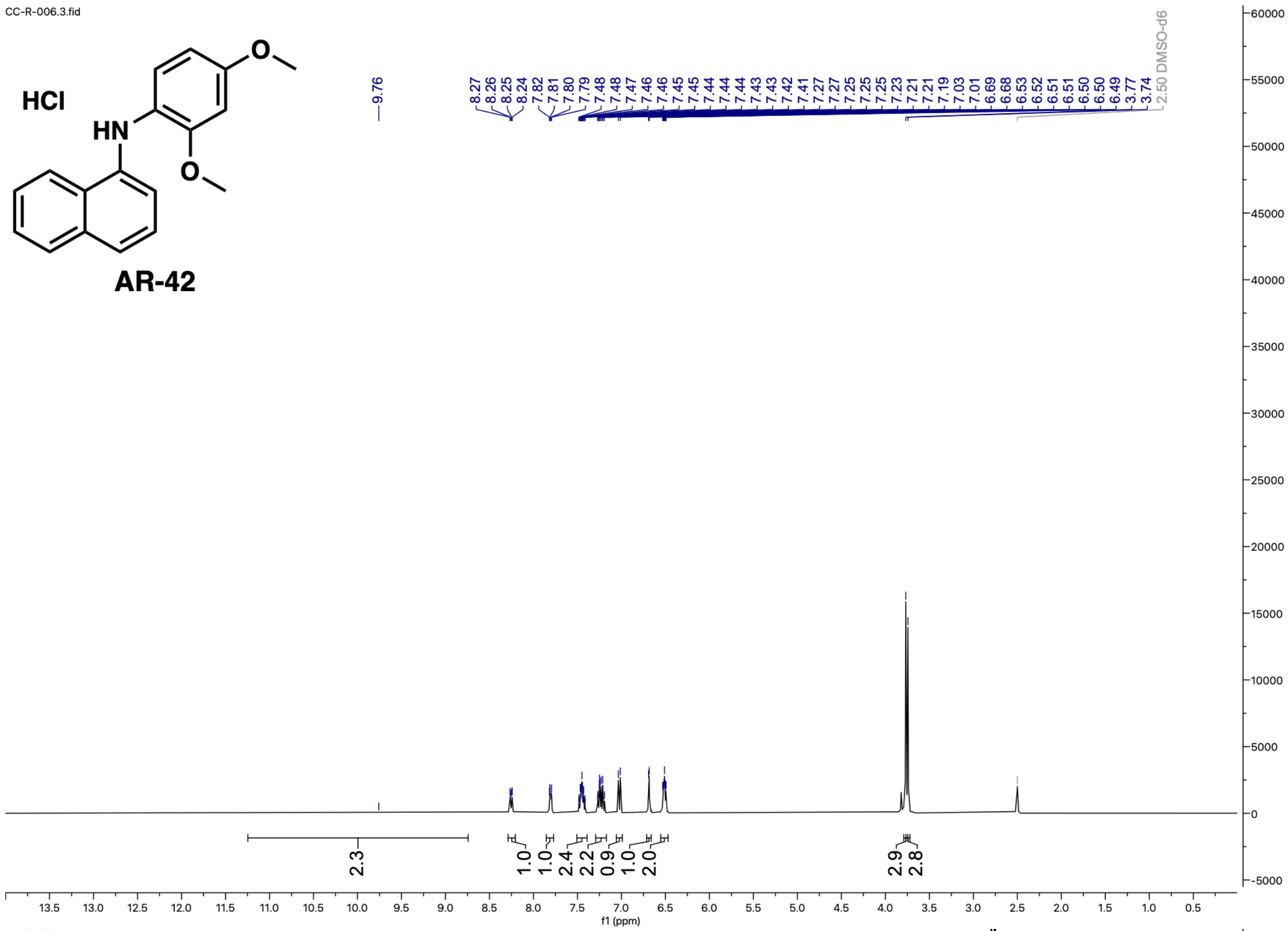
CC-R-006.3.fid
HCl
HN
O
AR-42
9.76
8.27
8.26
8.25
8.24
7.82
7.81
7.80
7.79
7.48
7.48
7.47
7.46
7.46
7.45
7.45
7.44
7.44
7.44
7.43
7.43
7.42
7.41
7.27
7.27
7.25
7.25
7.25
7.23
7.21
7.21
7.19
7.03
7.01
6.69
6.68
6.53
6.52
6.51
6.51
6.50
6.50
6.49
3.77
3.74
2.50 DMSO-d6
2.3
1.0
1.0
2.4
2.2
0.9
1.0
2.0
2.9
2.8
f1 (ppm)

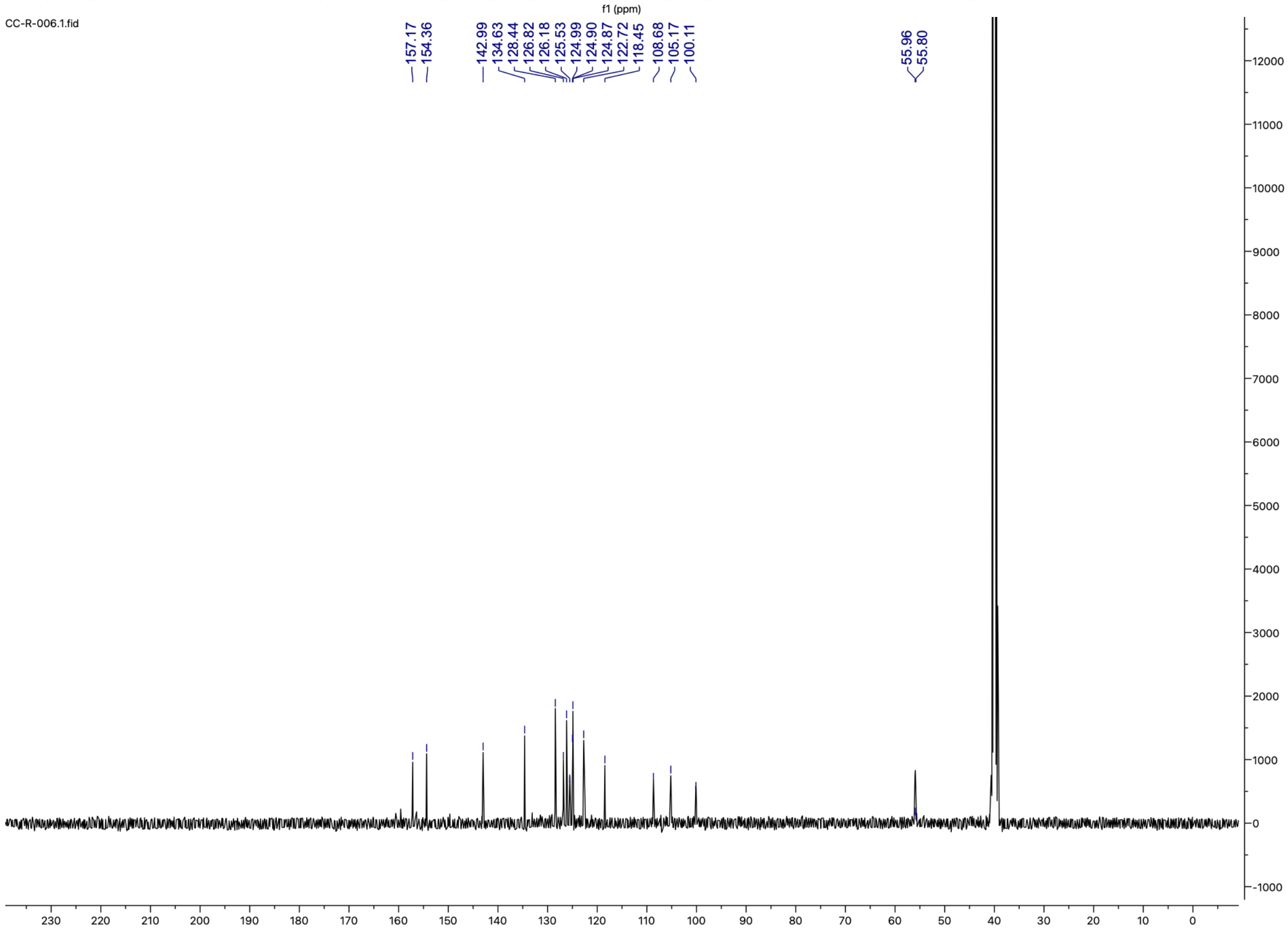
CC-R-006.1.fid
157.17
154.36
142.99
134.63
128.44
126.82
126.18
125.53
124.99
124.90
124.87
122.72
118.45
108.68
105.17
100.11
55.96
55.80
f1 (ppm)

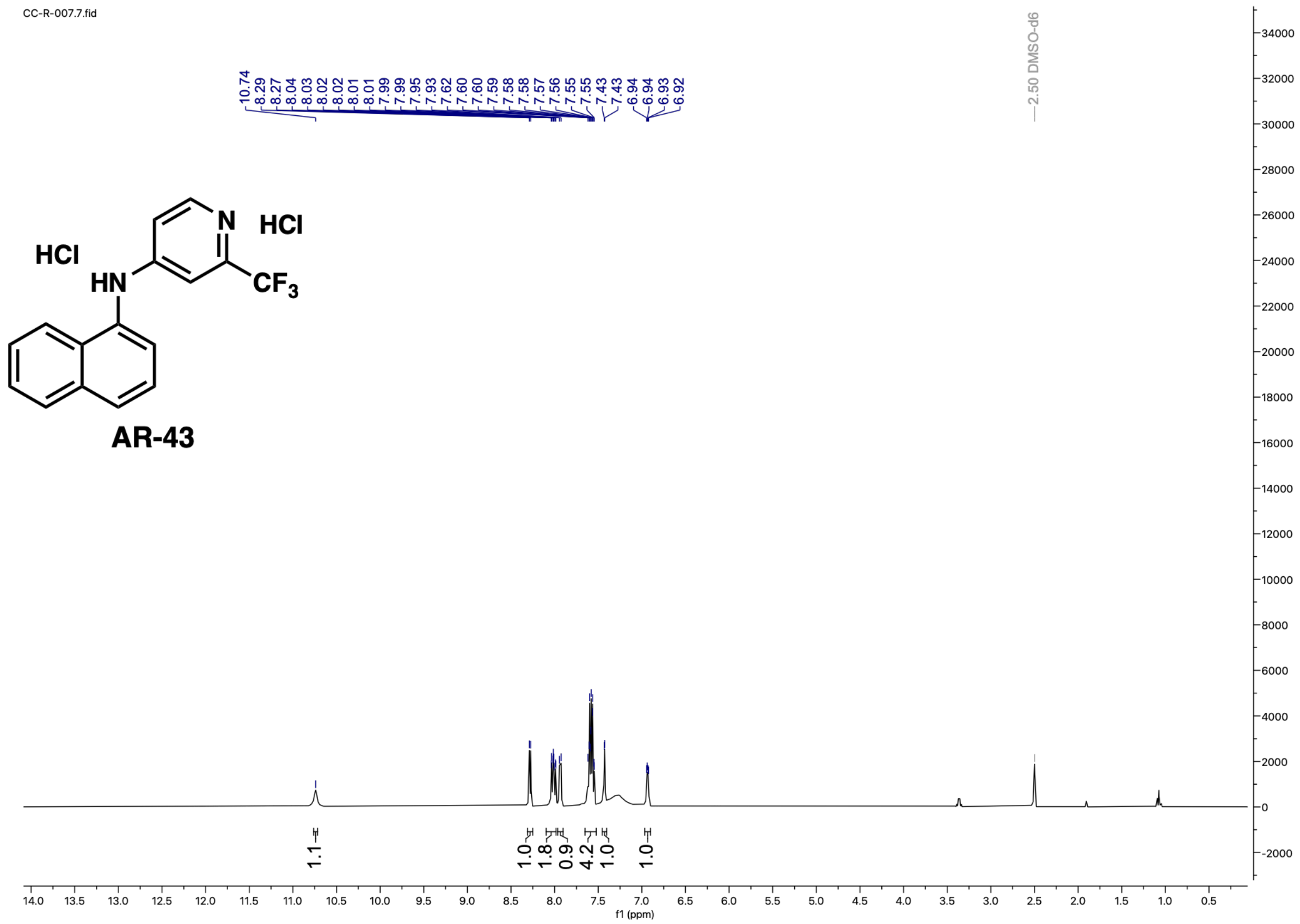
CC-R-007.7.fid
10.74
8.29
8.27
8.04
8.03
8.02
8.02
8.01
8.01
7.99
7.99
7.95
7.93
7.62
7.60
7.60
7.59
7.58
7.58
7.57
7.56
7.55
7.55
7.43
7.43
6.94
6.94
6.93
6.92
2.50 DMSO-d6
HCl
HN
N
HCl
CF3
1.1
1.0
1.8
0.9
4.2
1.0
1.0
f1 (ppm)

AR-43

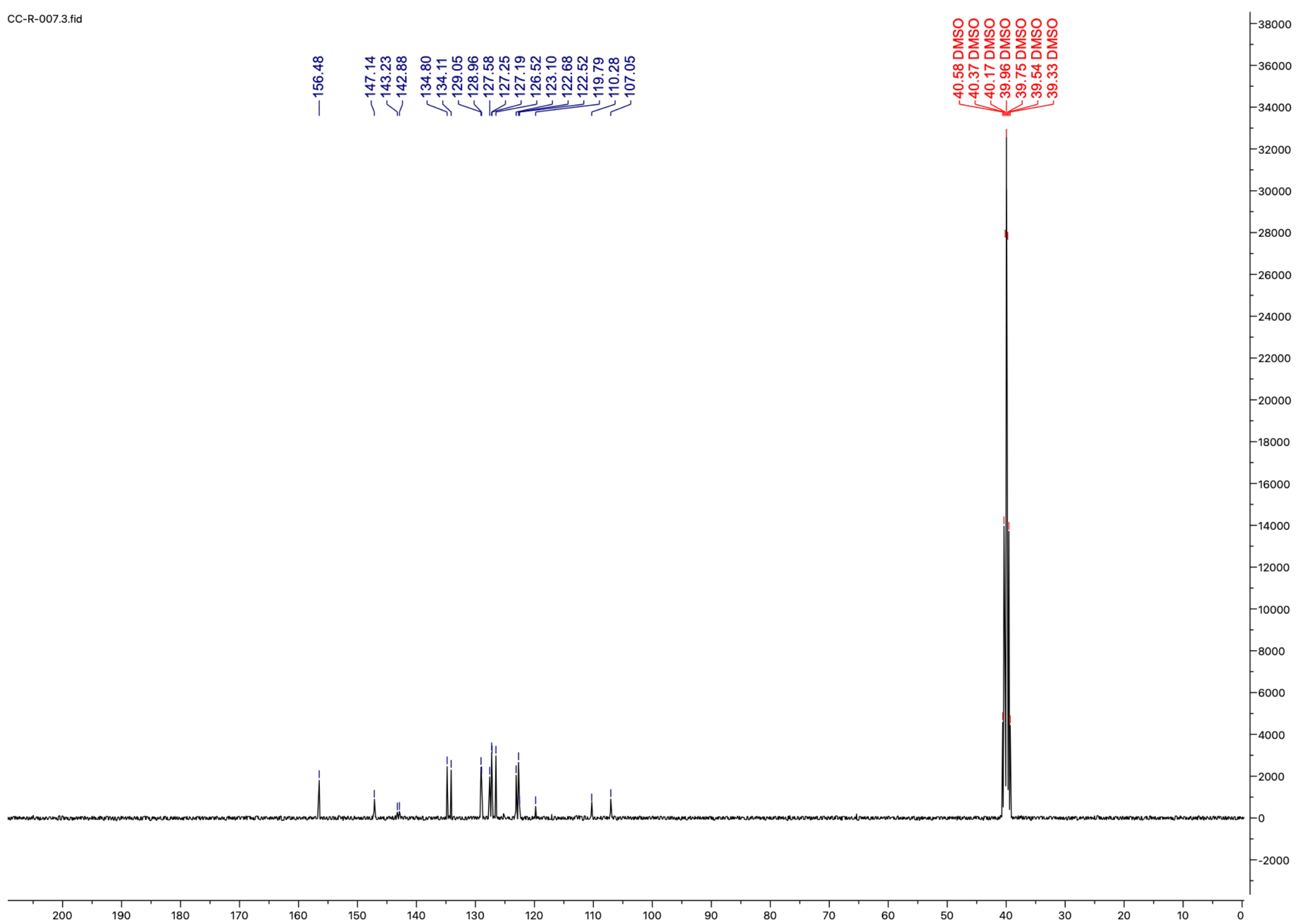
CC-R-007.3.fid
156.48
147.14
143.23
142.88
134.80
134.11
129.05
128.96
127.58
127.25
127.19
126.52
123.10
122.68
122.52
119.79
110.28
107.05
40.58 DMSO
40.37 DMSO
40.17 DMSO
39.96 DMSO
39.75 DMSO
39.54 DMSO
39.33 DMSO
f1 (ppm)

CC-R-007.1.fid

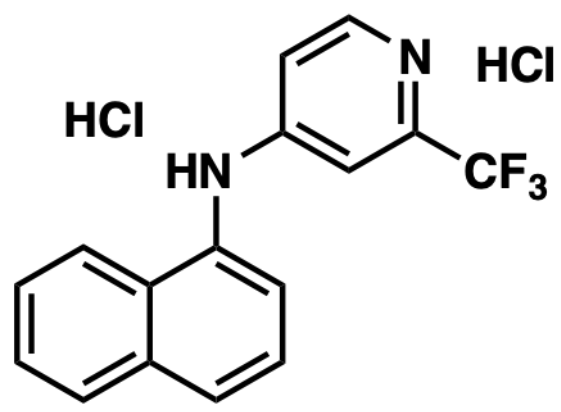

AR-43

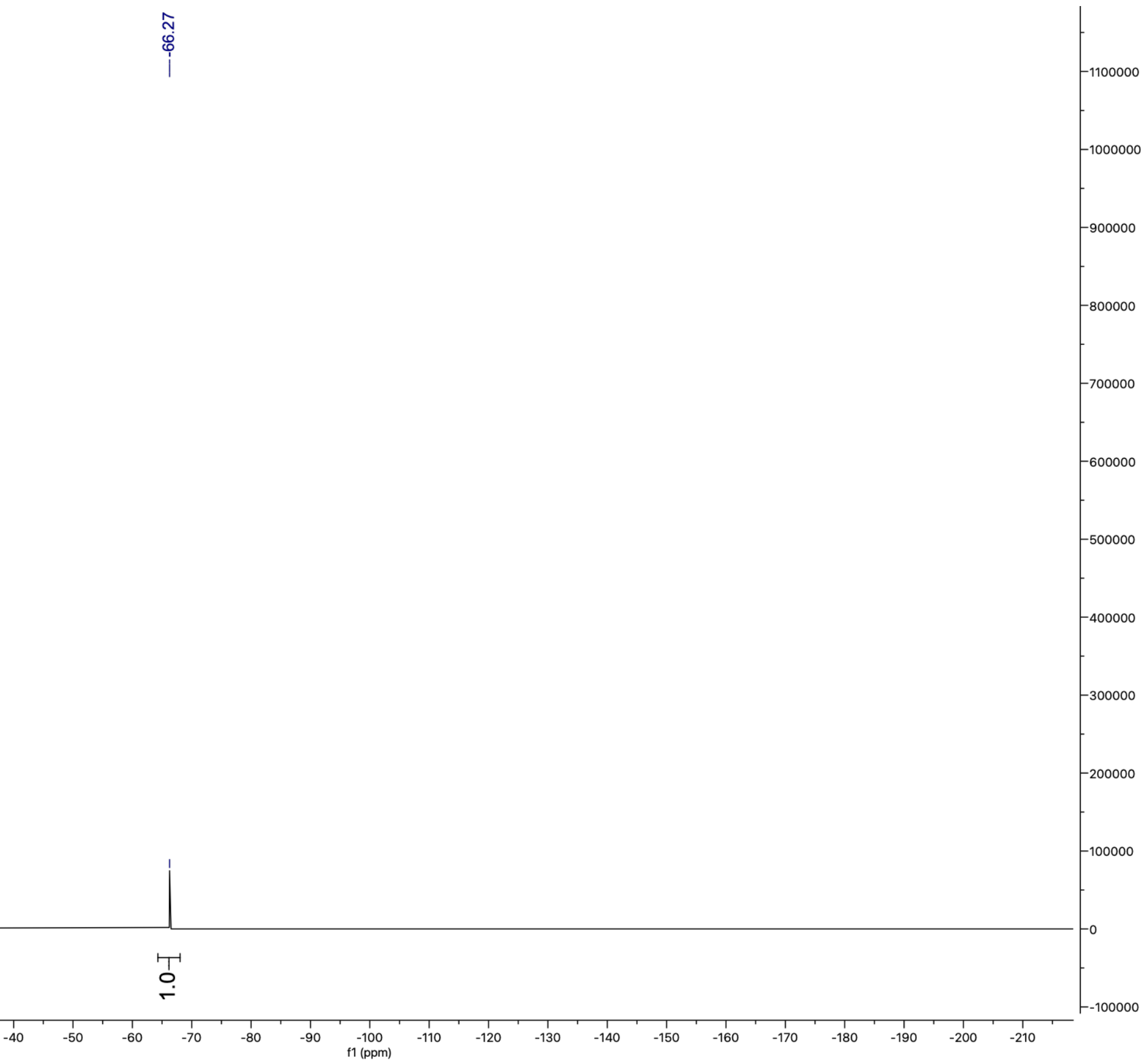

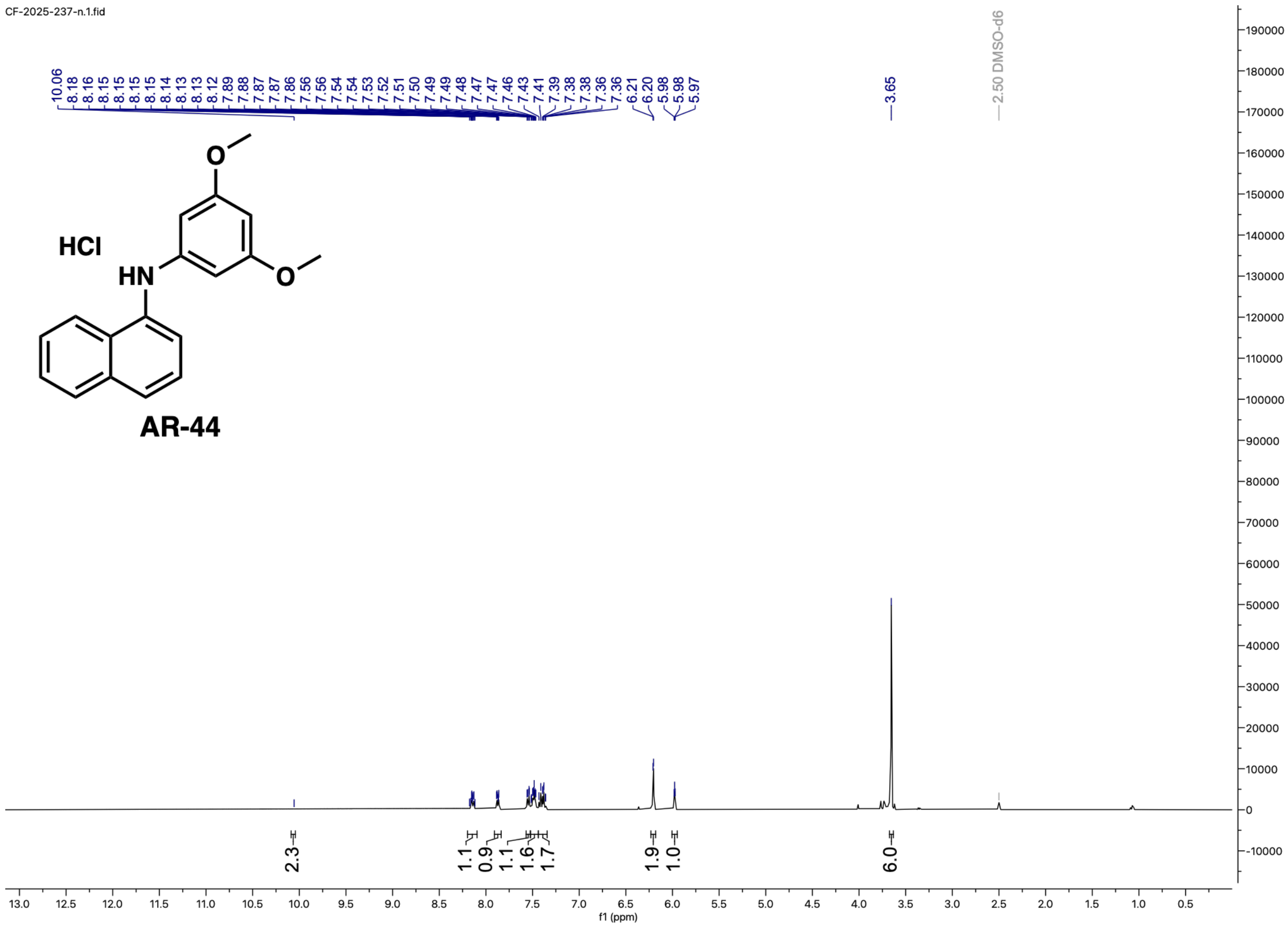
CF-2025-237-n.1.fid
HCl
HN
O
O
AR-44
10.06
3.65
2.50 DMSO-d6
2.3
1.1
0.9
1.1
1.6
1.7
1.9
1.0
6.0
f1 (ppm)

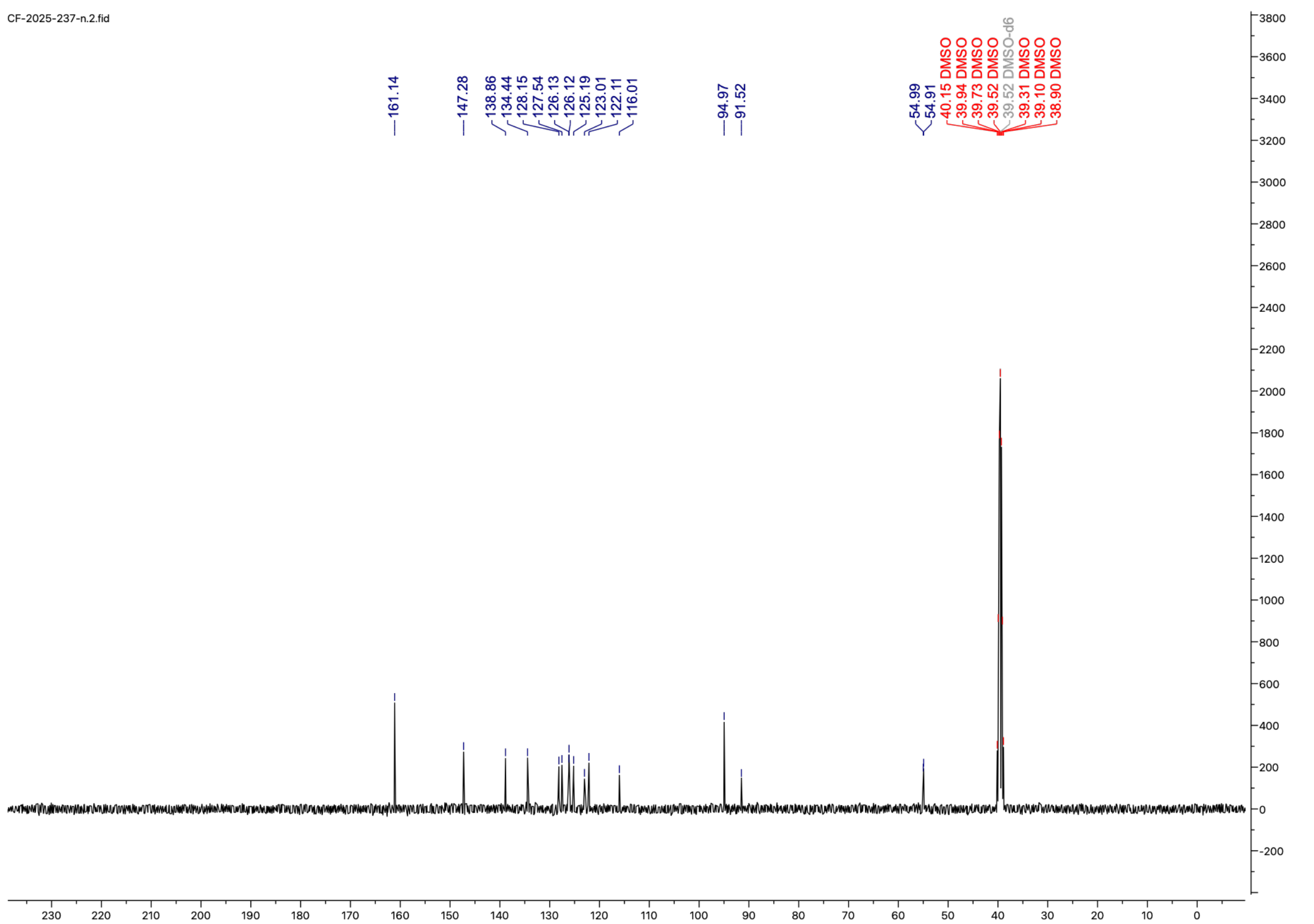
CF-2025-237-n.2.fid
161.14
147.28
138.86
134.44
128.15
127.54
126.13
126.12
125.19
123.01
122.11
116.01
94.97
91.52
54.99
54.91
40.15 DMSO
39.94 DMSO
39.73 DMSO
39.52 DMSO-d6
39.31 DMSO
39.10 DMSO
38.90 DMSO
f1 (ppm)

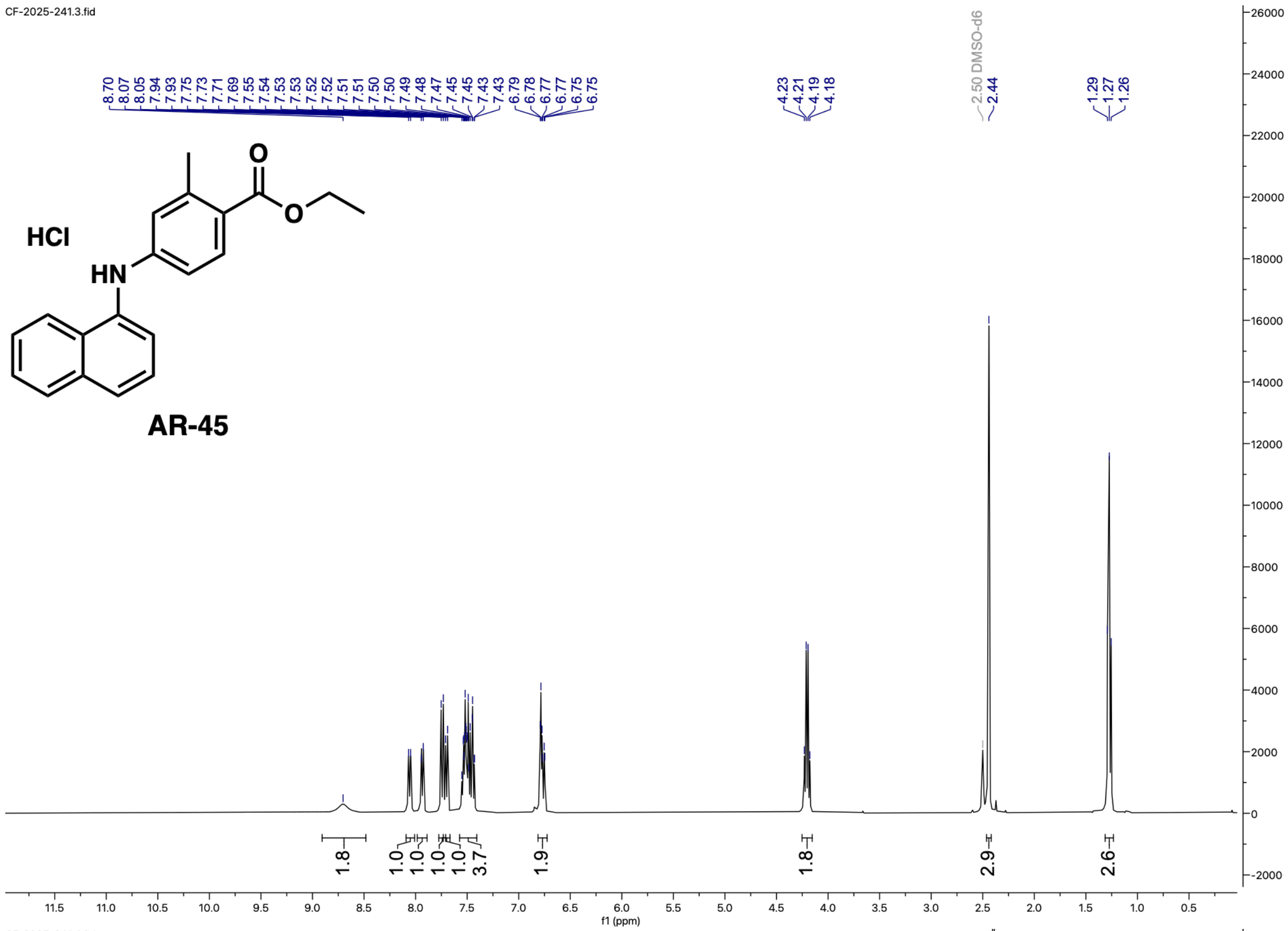
CF-2025-241.3.fid
8.70 8.07 8.05 7.94 7.93 7.75 7.73 7.71 7.69 7.55 7.54 7.53 7.53 7.52 7.52 7.51 7.51 7.50 7.50 7.49 7.48 7.47 7.45 7.45 7.43 7.43 6.79 6.78 6.77 6.77 6.75 6.75
4.23 4.21 4.19 4.18
2.50 DMSO-d6
2.44
1.29 1.27 1.26
HCl
HN
O
O
AR-45
1.8 1.0 1.0 1.0 1.0 3.7 1.9 1.8 2.9 2.6
f1 (ppm)

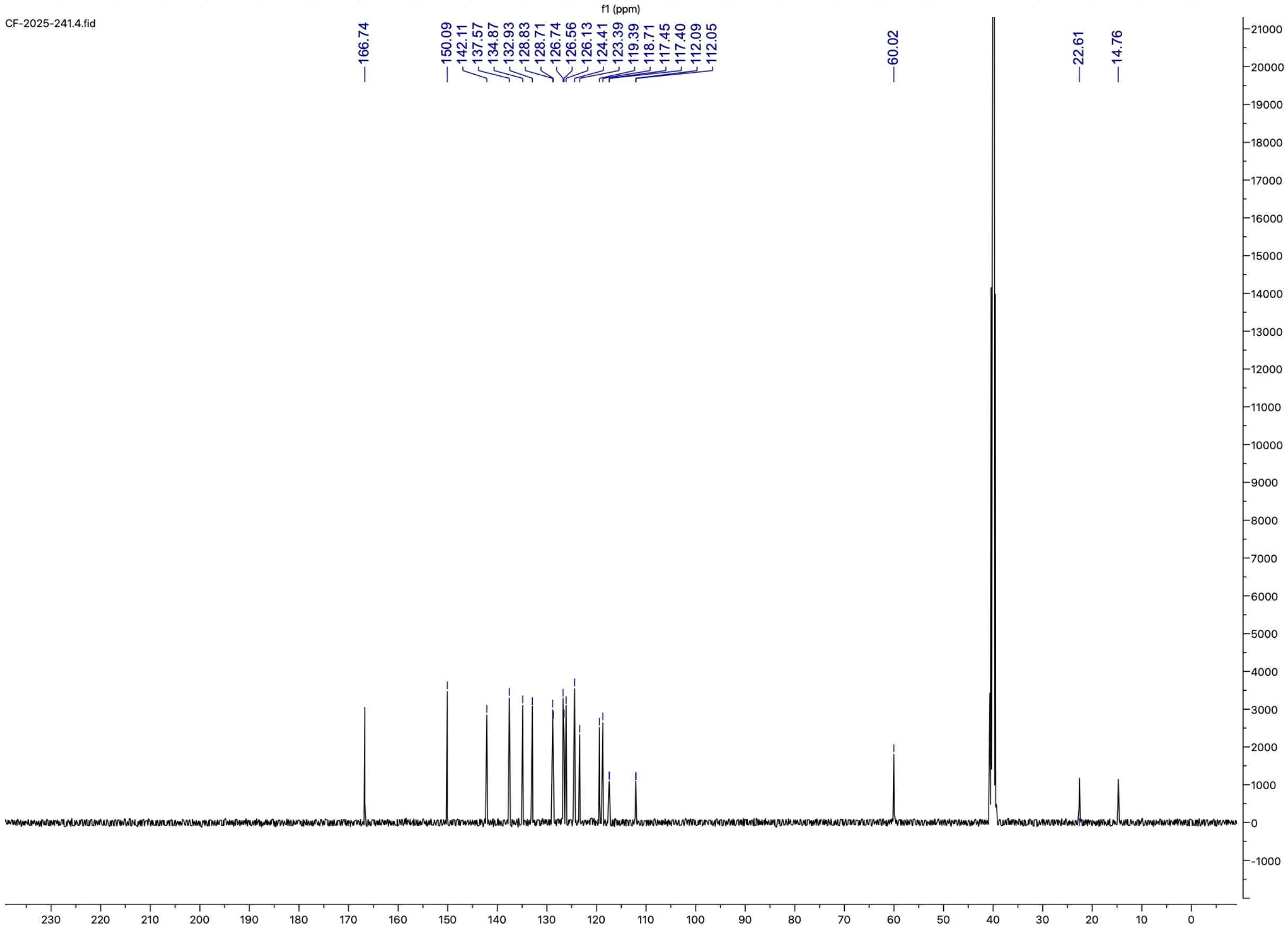
CF-2025-241.4.fid
166.74
150.09 142.11 137.57 134.87 132.93 128.83 128.71 126.74 126.56 126.13 124.41 123.39 119.39 118.71 117.45 117.40 112.09 112.05
60.02
22.61
14.76
f1 (ppm)

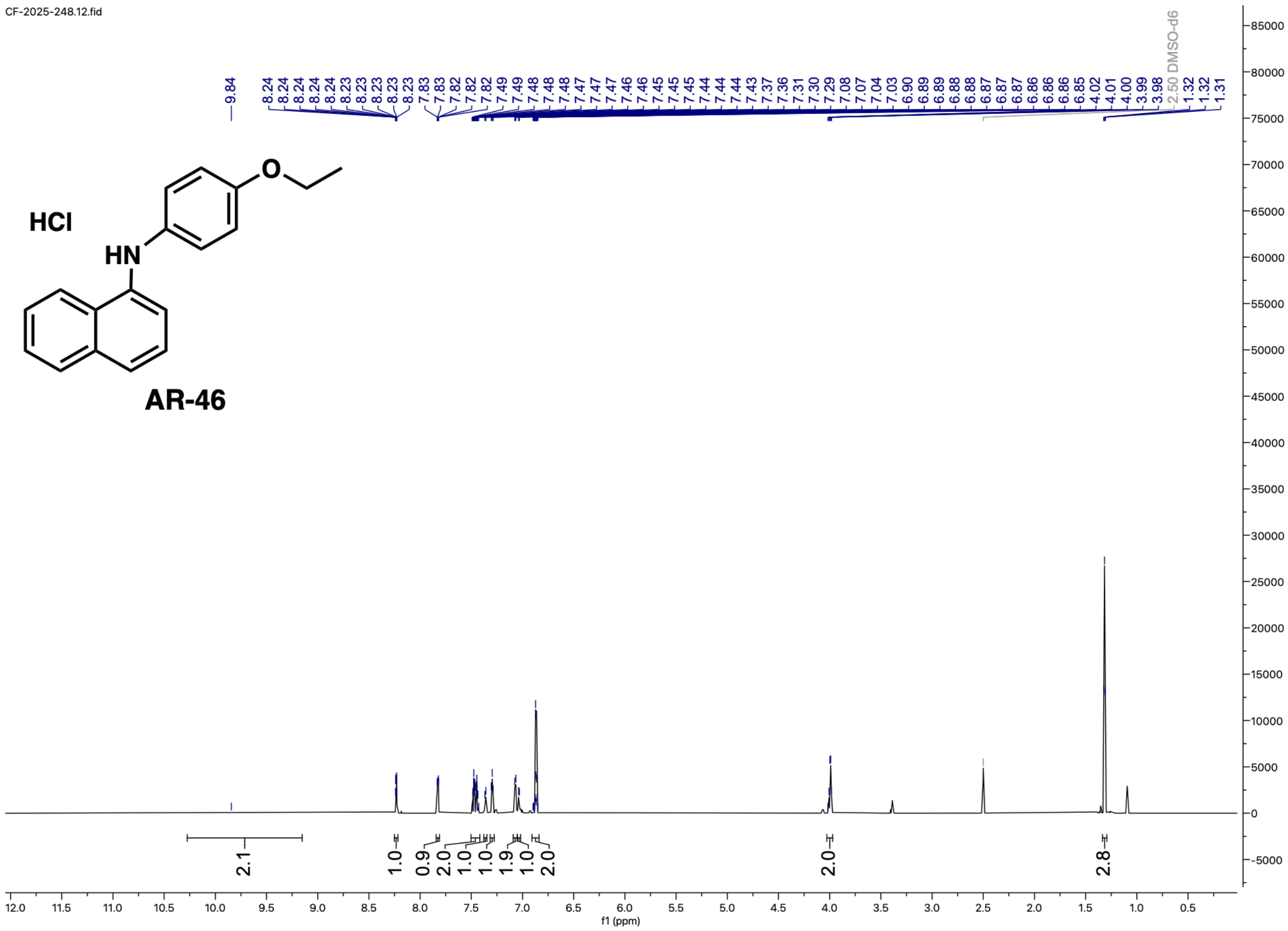
CF-2025-248.12.fid
9.84
8.24 8.24 8.24 8.24 8.24 8.23 8.23 8.23 8.23 7.83 7.83 7.82 7.82 7.82 7.49 7.48 7.48 7.47 7.47 7.46 7.46 7.45 7.45 7.44 7.44 7.44 7.43 7.37 7.36 7.31 7.30 7.29 7.08 7.07 7.04 7.03 6.90 6.89 6.89 6.88 6.88 6.87 6.87 6.86 6.86 6.86 6.85 4.02 4.01 4.00 3.99 3.98
2.50 DMSO-d6
1.32 1.32 1.31
HCl
HN
O
AR-46
2.1
1.0
0.9
2.0
1.0
1.0
1.9
1.0
2.0
2.0
2.8
f1 (ppm)

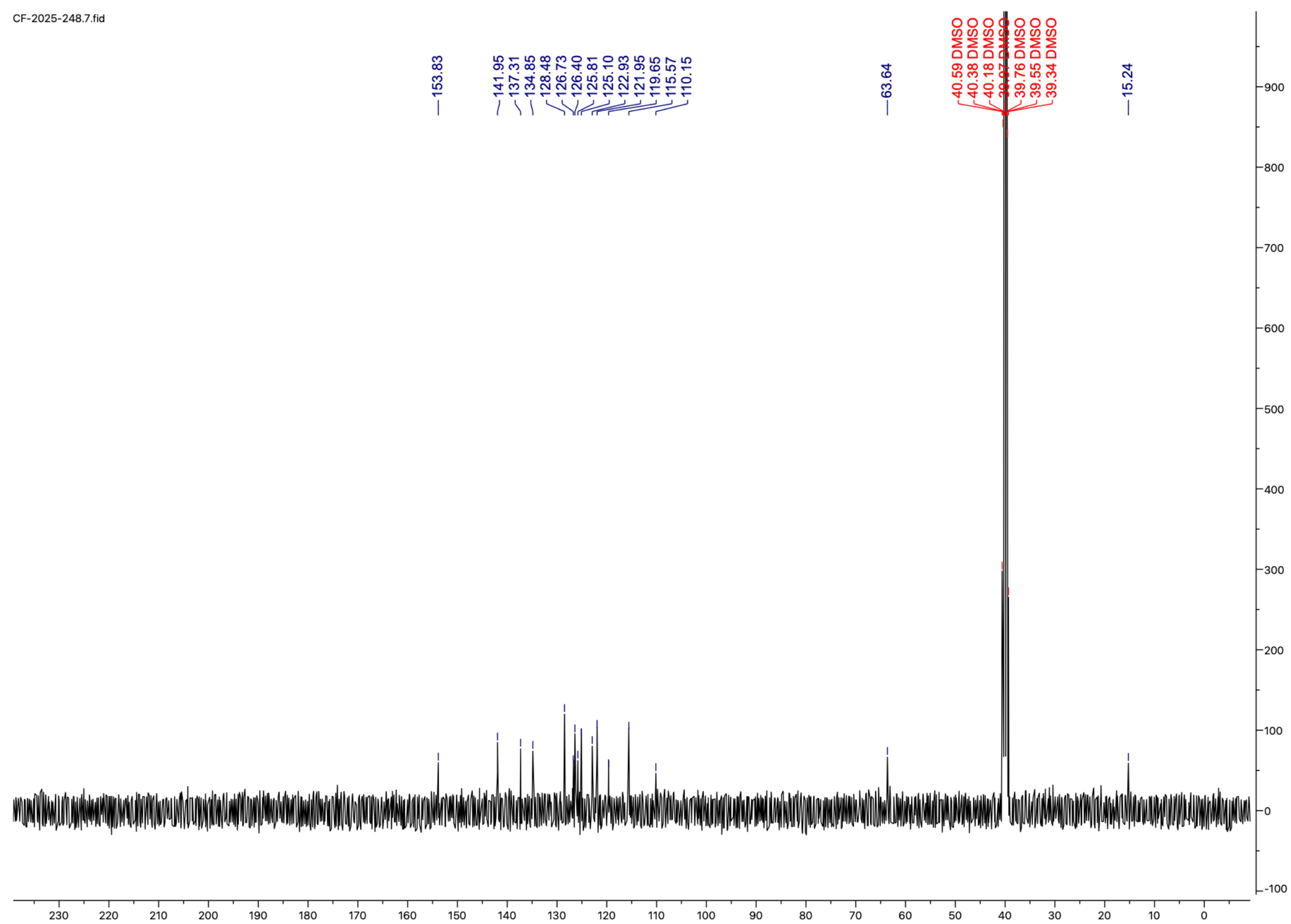
CF-2025-248.7.fid
153.83
141.95 137.31 134.85 128.48 126.73 126.40 125.81 125.10 122.93 121.95 119.65 115.57 110.15
63.64
40.59 DMSO 40.38 DMSO 40.18 DMSO 39.97 DMSO 39.76 DMSO 39.55 DMSO 39.34 DMSO
15.24
f1 (ppm)

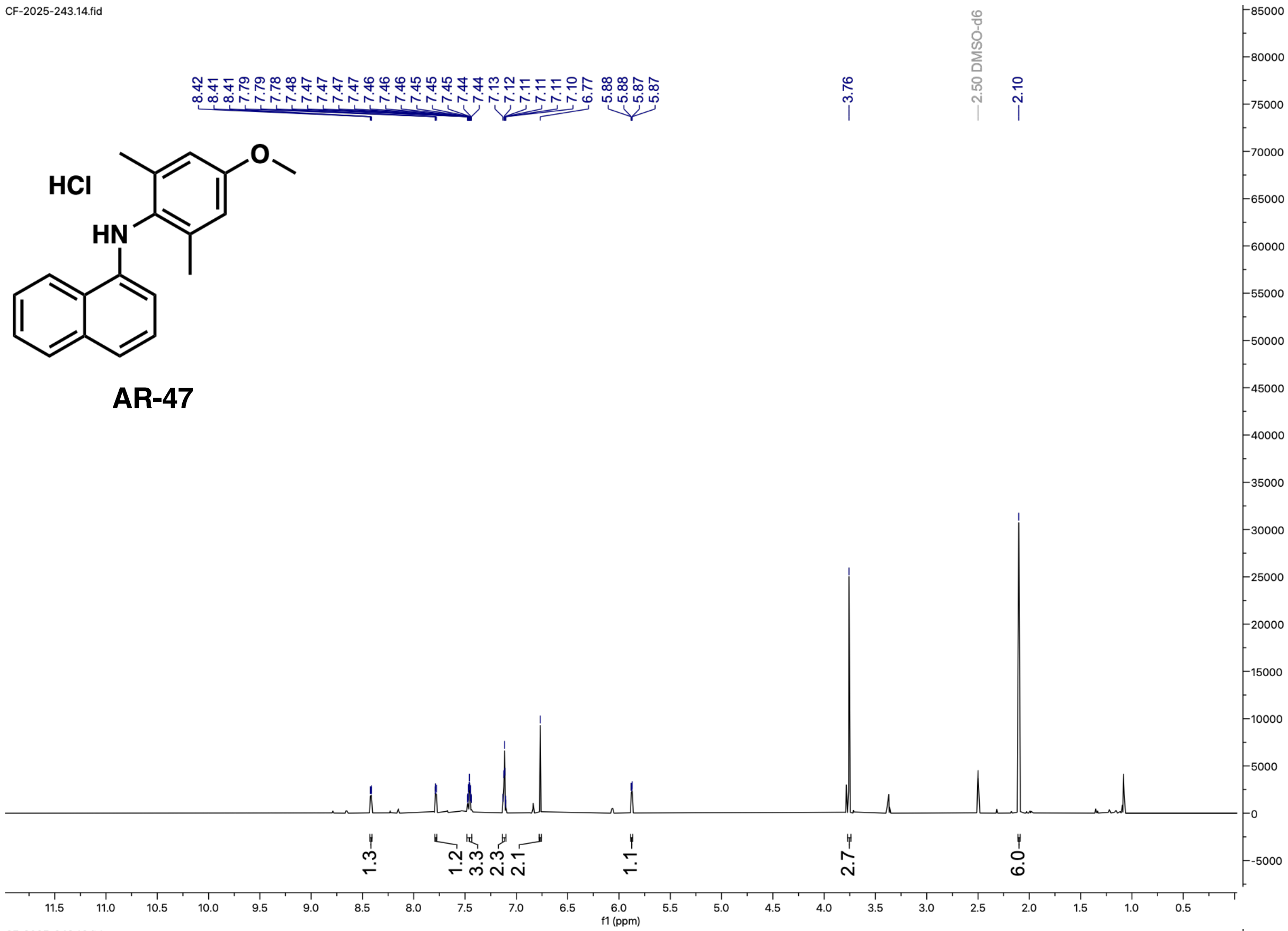

CF-2025-243.14.fid
8.42 8.41 8.41 7.79 7.79 7.78 7.48 7.47 7.47 7.47 7.46 7.46 7.46 7.45 7.45 7.45 7.44 7.44 7.13 7.12 7.11 7.11 7.11 7.10 6.77 5.88 5.88 5.87 5.87
3.76
2.50 DMSO-d6
2.10
HCl
HN
O
AR-47
1.3
1.2
3.3
2.3
2.1
1.1
2.7
6.0
f1 (ppm)

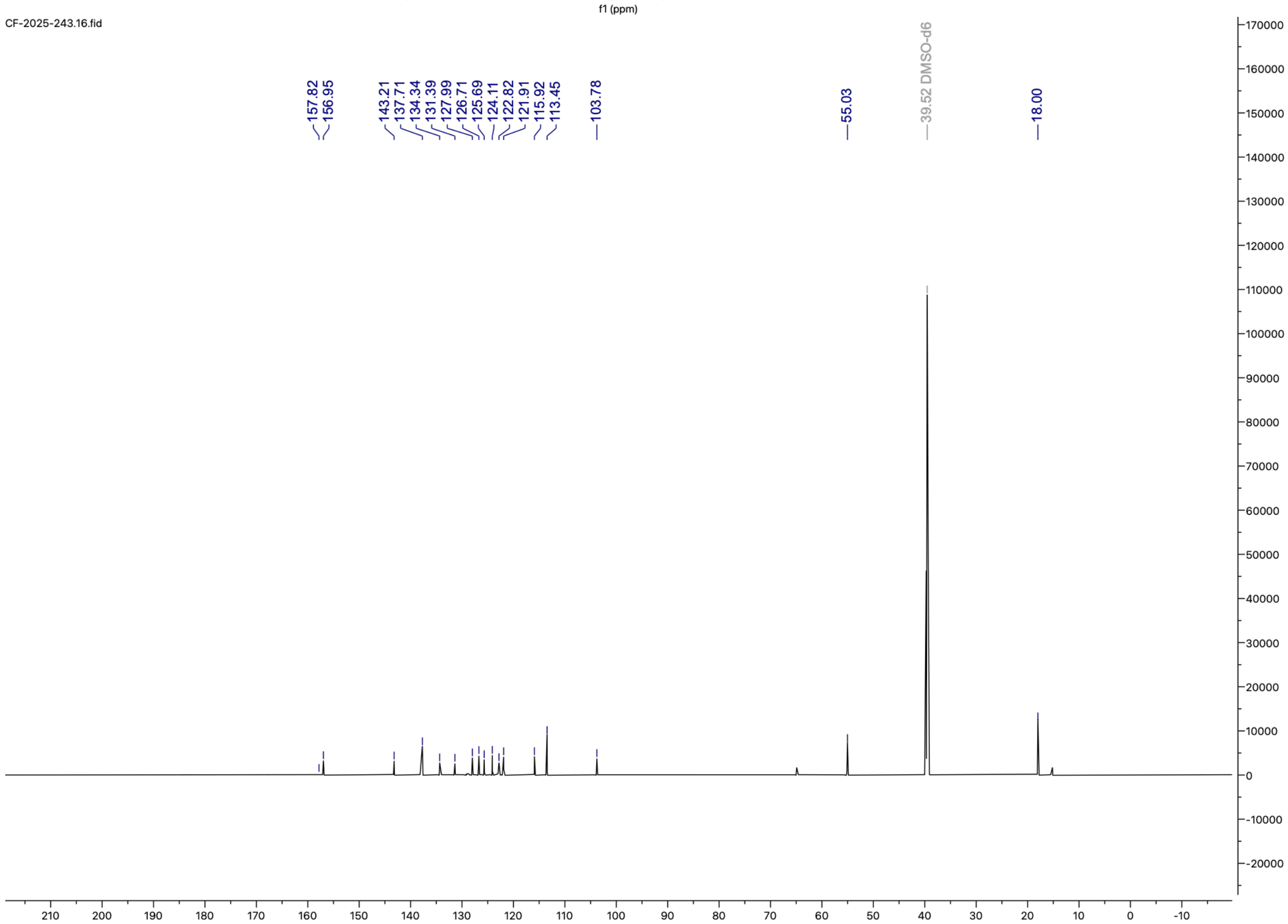

CF-2025-243.16.fid
157.82 156.95
143.21 137.71 134.34 131.39 127.99 126.71 125.69 124.11 122.82 121.91 115.92 113.45
103.78
55.03
39.52 DMSO-d6
18.00
f1 (ppm)

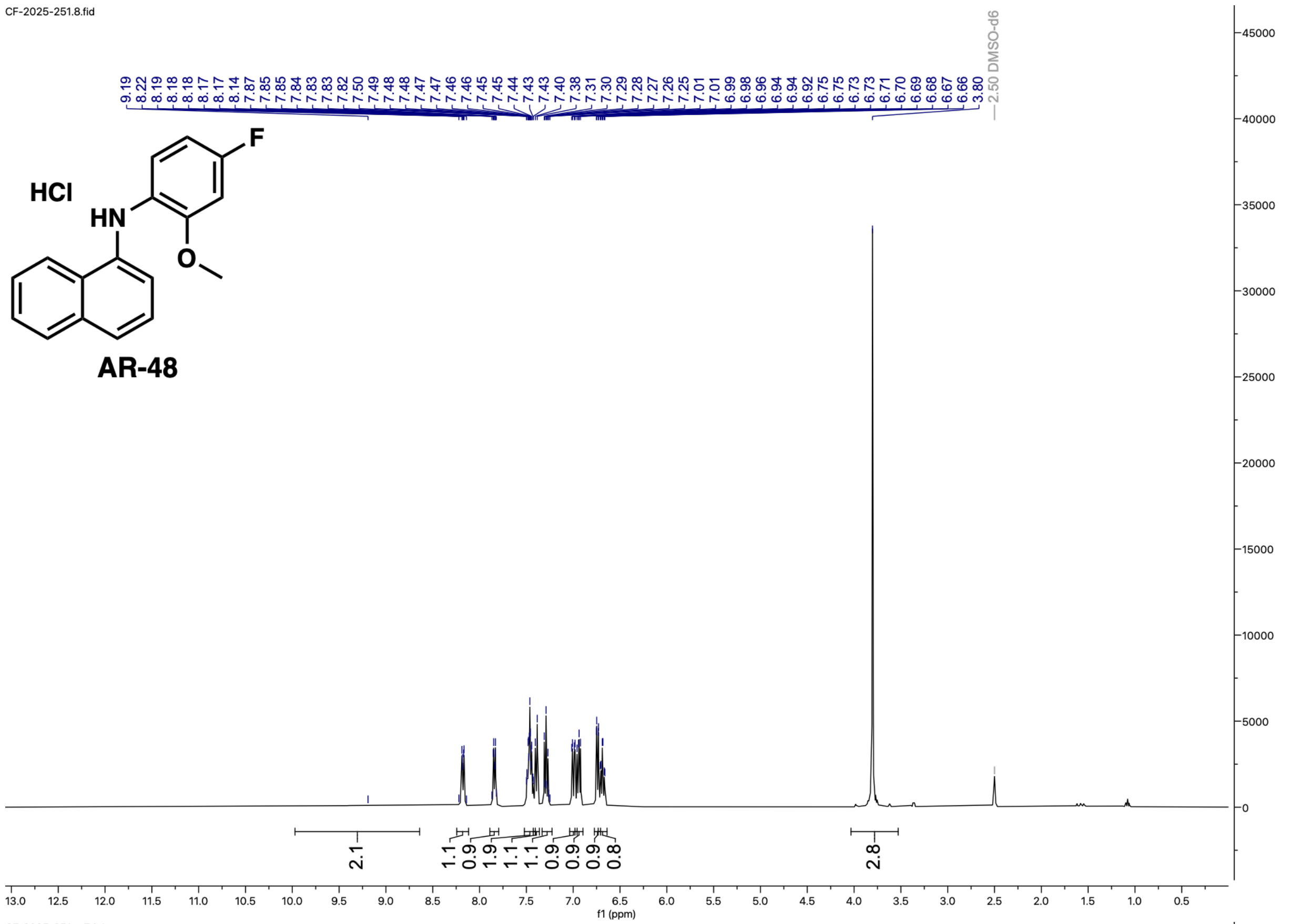
CF-2025-251.8.fid
9.19 8.22 8.19 8.18 8.18 8.17 8.17 8.14 7.87 7.85 7.85 7.84 7.83 7.83 7.82 7.50 7.49 7.48 7.48 7.47 7.47 7.46 7.46 7.45 7.45 7.44 7.43 7.43 7.40 7.38 7.31 7.30 7.29 7.28 7.27 7.26 7.25 7.01 7.01 6.99 6.98 6.96 6.94 6.94 6.92 6.92 6.75 6.75 6.73 6.73 6.71 6.70 6.69 6.68 6.67 6.66 3.80
2.50 DMSO-d6
HCl
HN
F
O
AR-48
2.1 1.1 0.9 1.9 1.1 1.1 0.9 0.9 0.9 0.8 2.8
f1 (ppm)

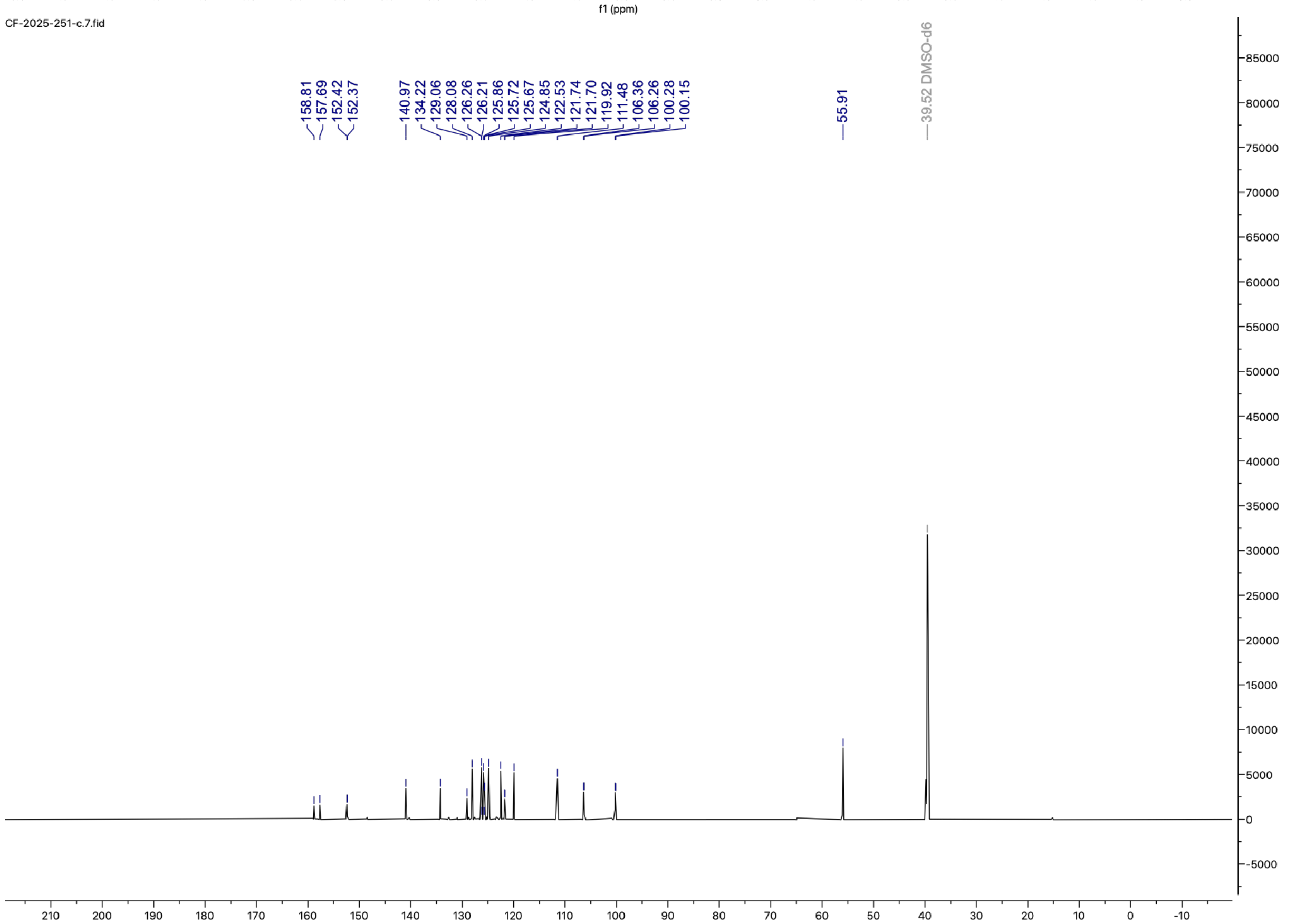
CF-2025-251-c.7.fid
158.81 157.69 152.42 152.37 140.97 134.22 129.06 128.08 126.26 126.21 125.86 125.72 125.67 124.85 122.53 121.74 121.70 119.92 111.48 106.36 106.26 100.28 100.15 55.91
39.52 DMSO-d6
f1 (ppm)

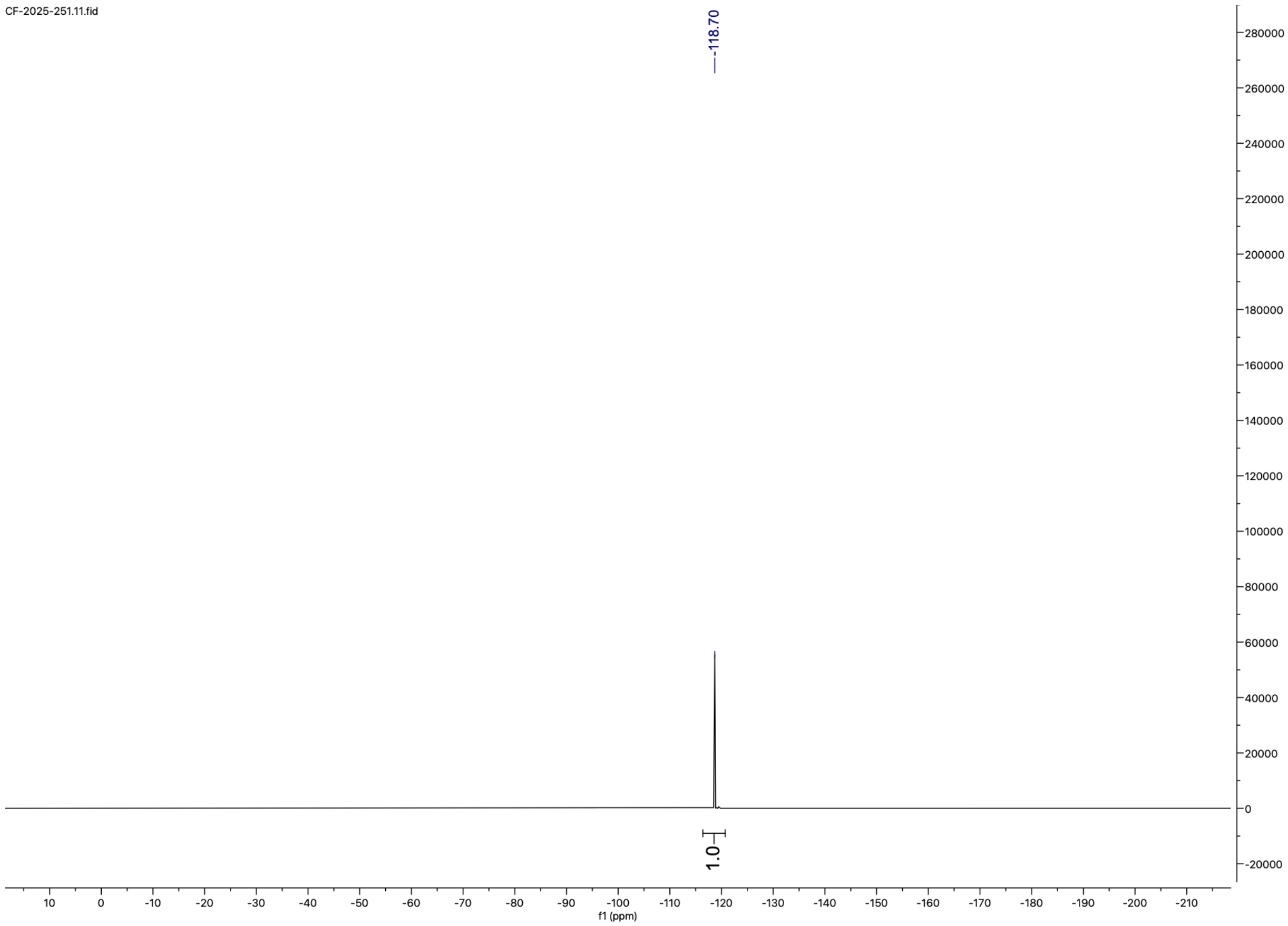
CF-2025-251.11.fid
-118.70
1.0
280000
260000
240000
220000
200000
180000
160000
140000
120000
100000
80000
60000
40000
20000
0
-20000
10
0
-10
-20
-30
-40
-50
-60
-70
-80
-90
-100
-110
-120
-130
-140
-150
-160
-170
-180
-190
-200
-210
f1 (ppm)

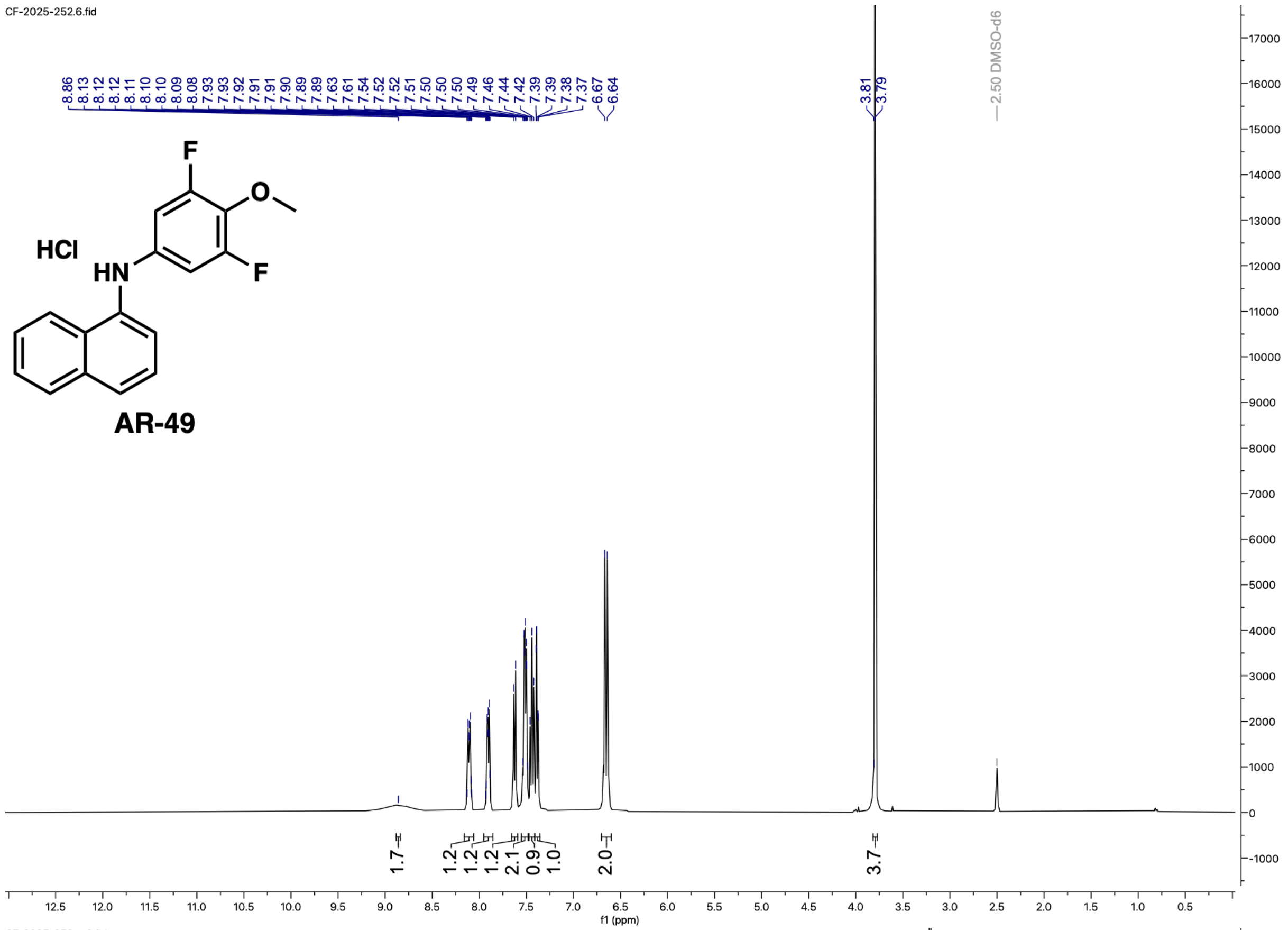

CF-2025-252.6.fid
8.86 8.13 8.12 8.12 8.11 8.10 8.09 8.08 7.93 7.93 7.92 7.91 7.91 7.90 7.89 7.89 7.63 7.61 7.54 7.52 7.52 7.51 7.50 7.50 7.50 7.49 7.46 7.44 7.42 7.39 7.39 7.38 7.37 6.67 6.64
3.81 3.79
2.50 DMSO-d6
HCl
HN
F
O
F
AR-49
1.7 1.2 1.2 1.2 2.1 0.9 1.0 2.0 3.7
f1 (ppm)

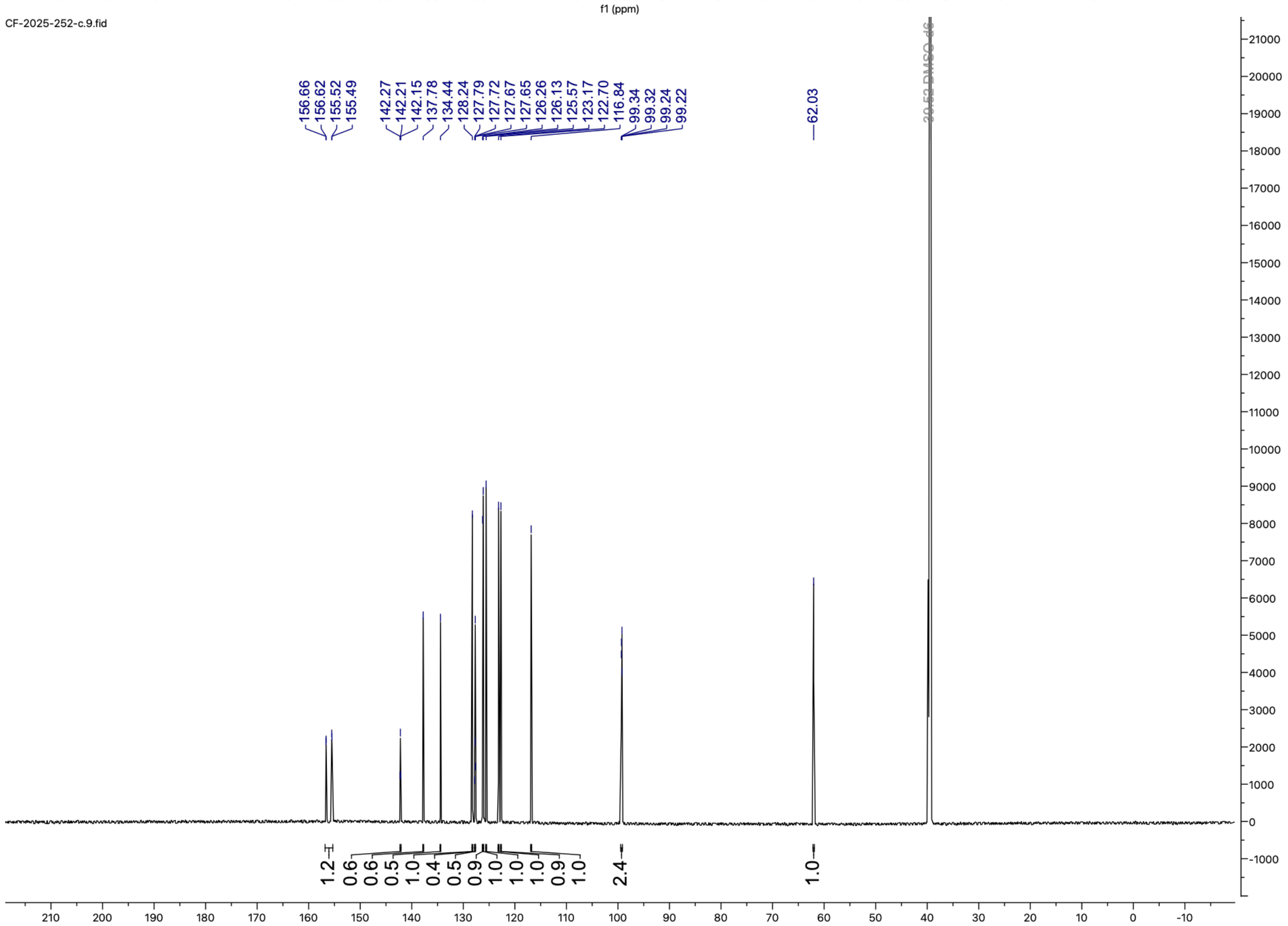

CF-2025-252-c.9.fid
156.66 156.62 155.52 155.49 142.27 142.21 142.15 137.78 134.44 128.24 127.79 127.72 127.67 127.65 126.26 126.13 125.57 123.17 122.70 116.84 99.34 99.32 99.24 99.22
62.03
39.52 DMSO-d6
1.2 0.6 0.6 0.5 1.0 0.4 0.5 0.9 1.0 1.0 1.0 0.9 1.0 2.4 1.0
f1 (ppm)

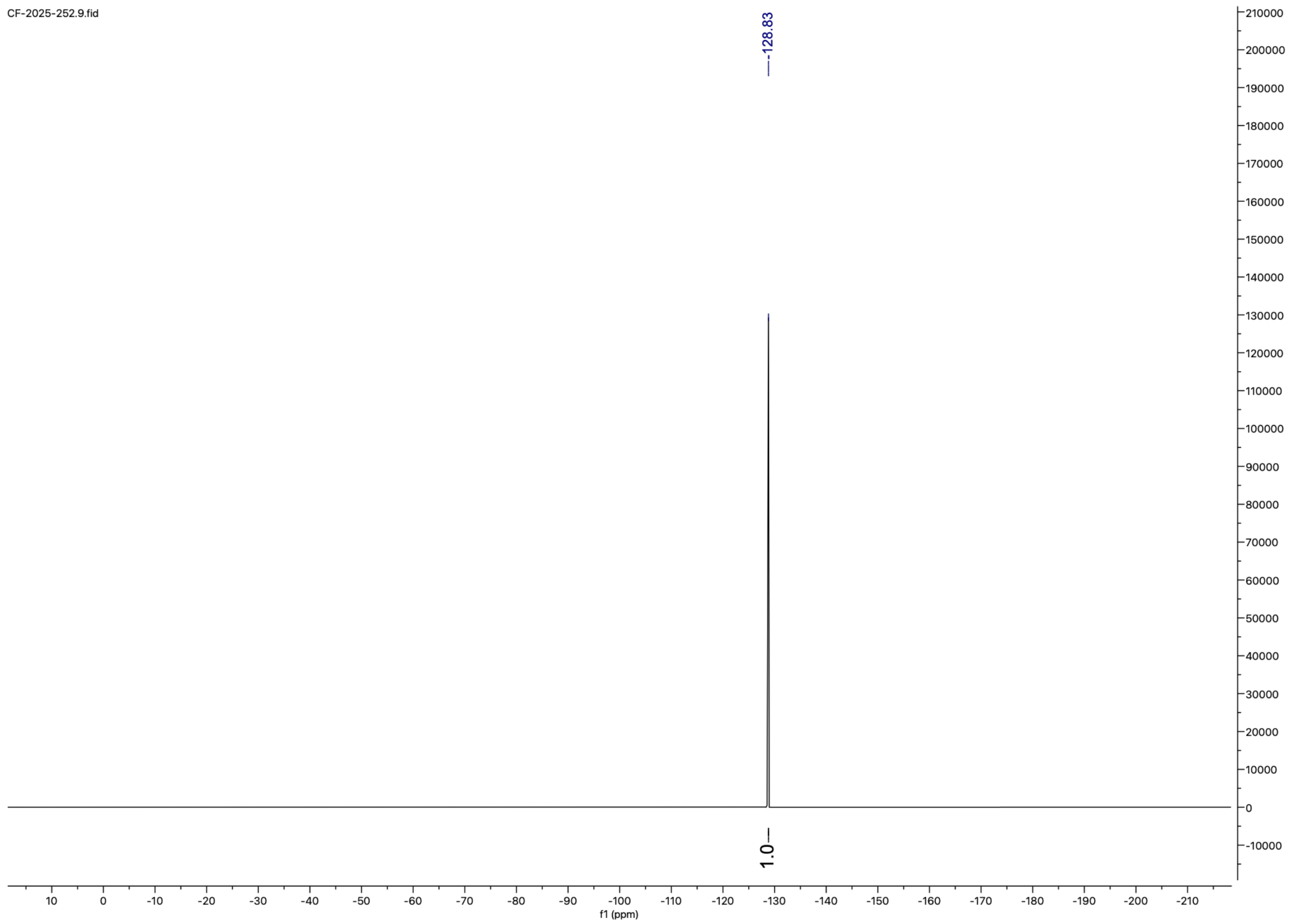

CF-2025-252.9.fid
-128.83
1.0
f1 (ppm)

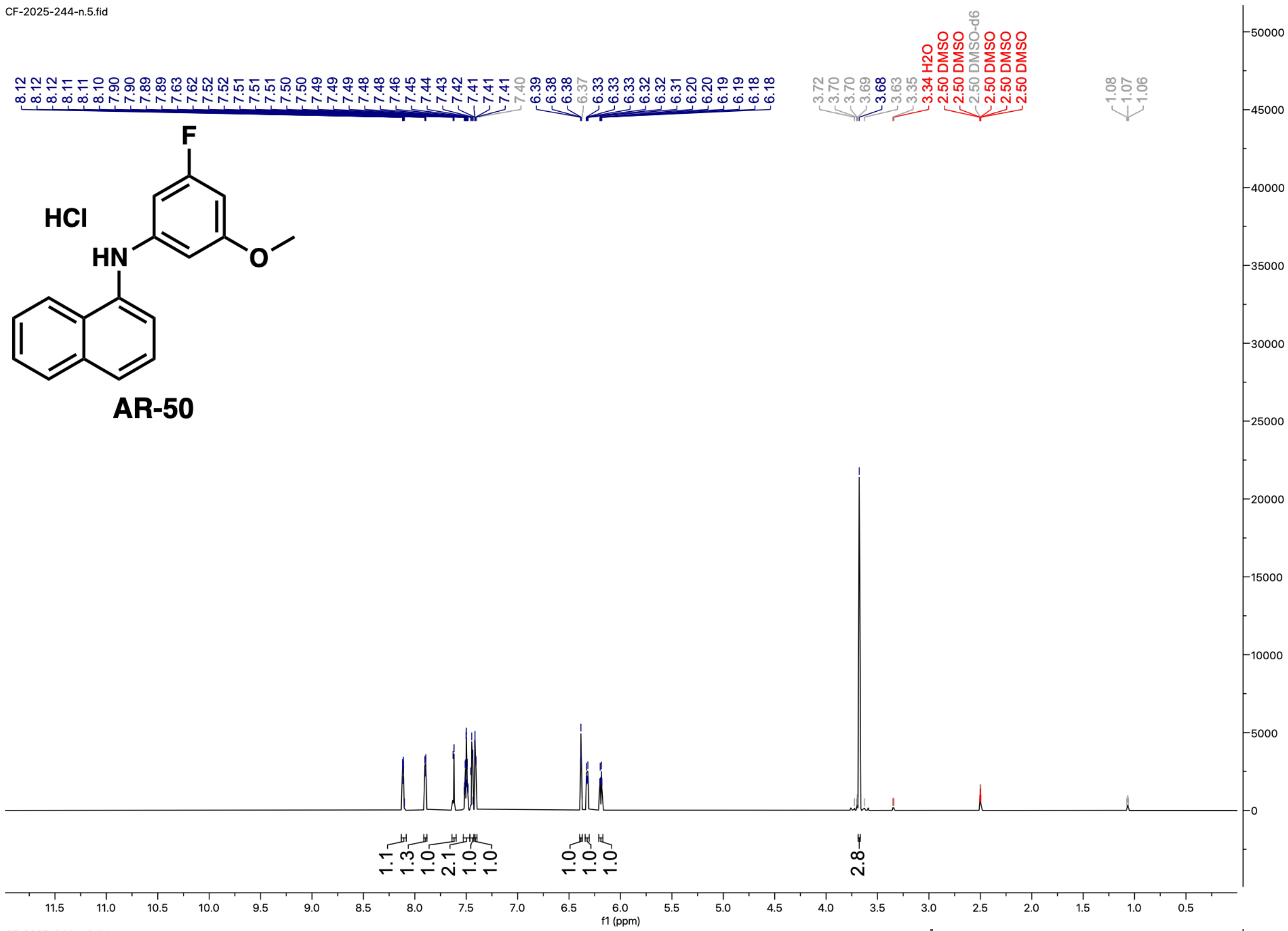

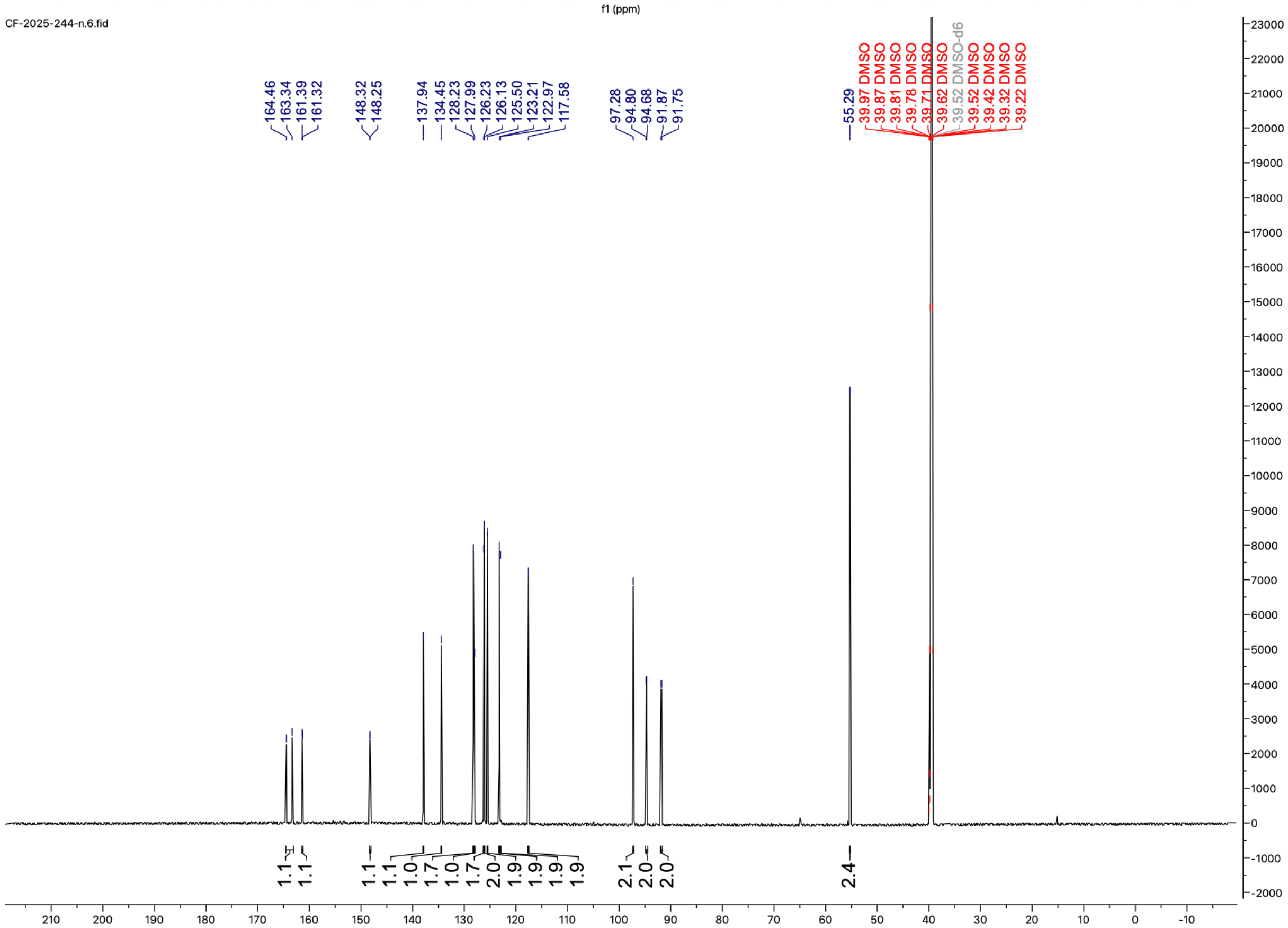

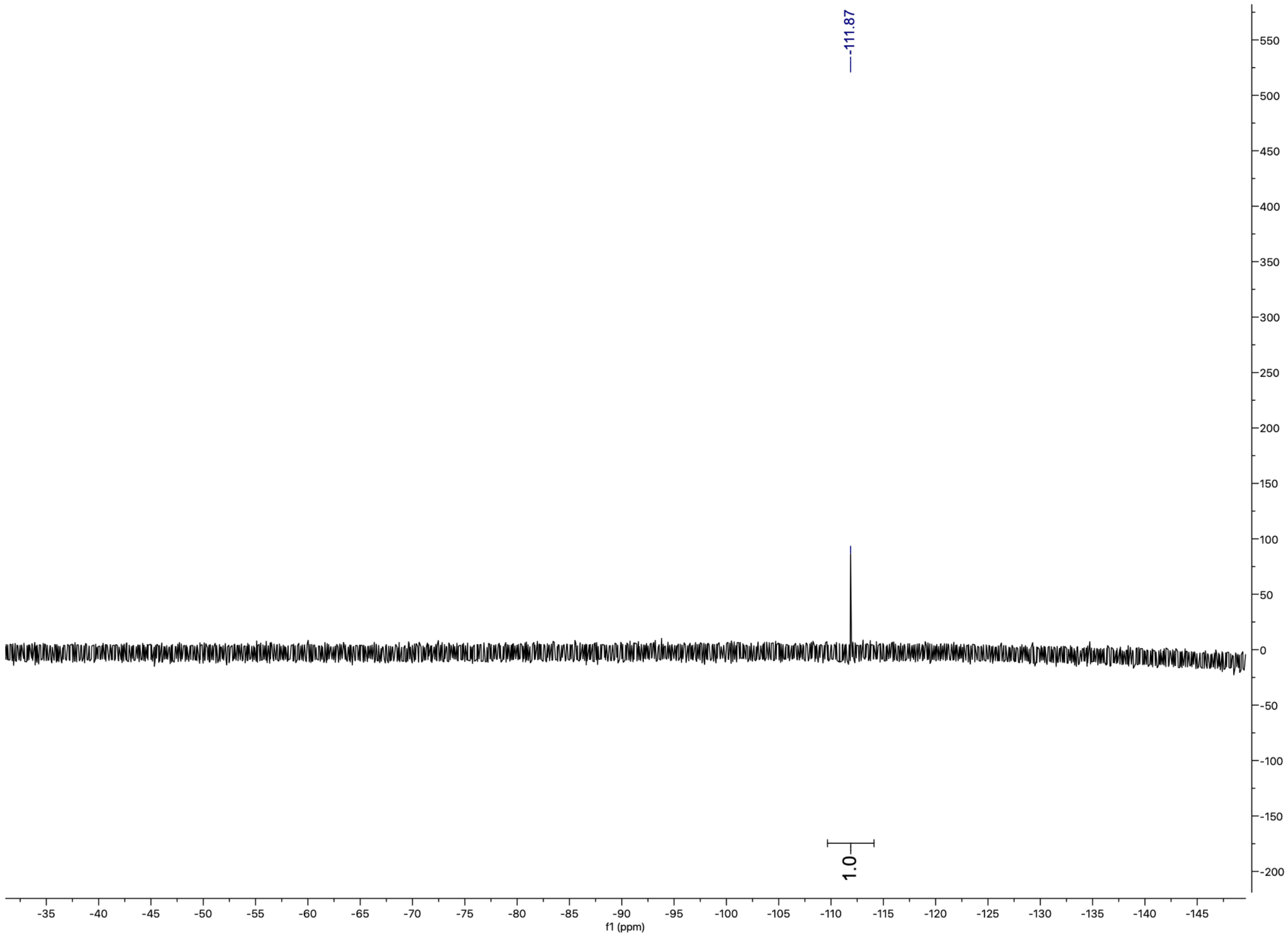

-111.87
1.0
f1 (ppm)

ALK-03

**Instrument:**	LCMS	**Location:**	P1-F10
		**Injection:**	1 of 1
**Acq. method:**	analytical-testing-NEW-NPN.amx	**Injection volume:**	5.000 µL
**Analysis method:**	*Purity-Cole.pmx	**Acq. operator:**	SYSTEM
**Column name:**	Poroshell_120_EC-C18	**Diameter:**	3
**Serial #:**	USCFW33961	**Length:**	150
		**Particle Size**	2.7

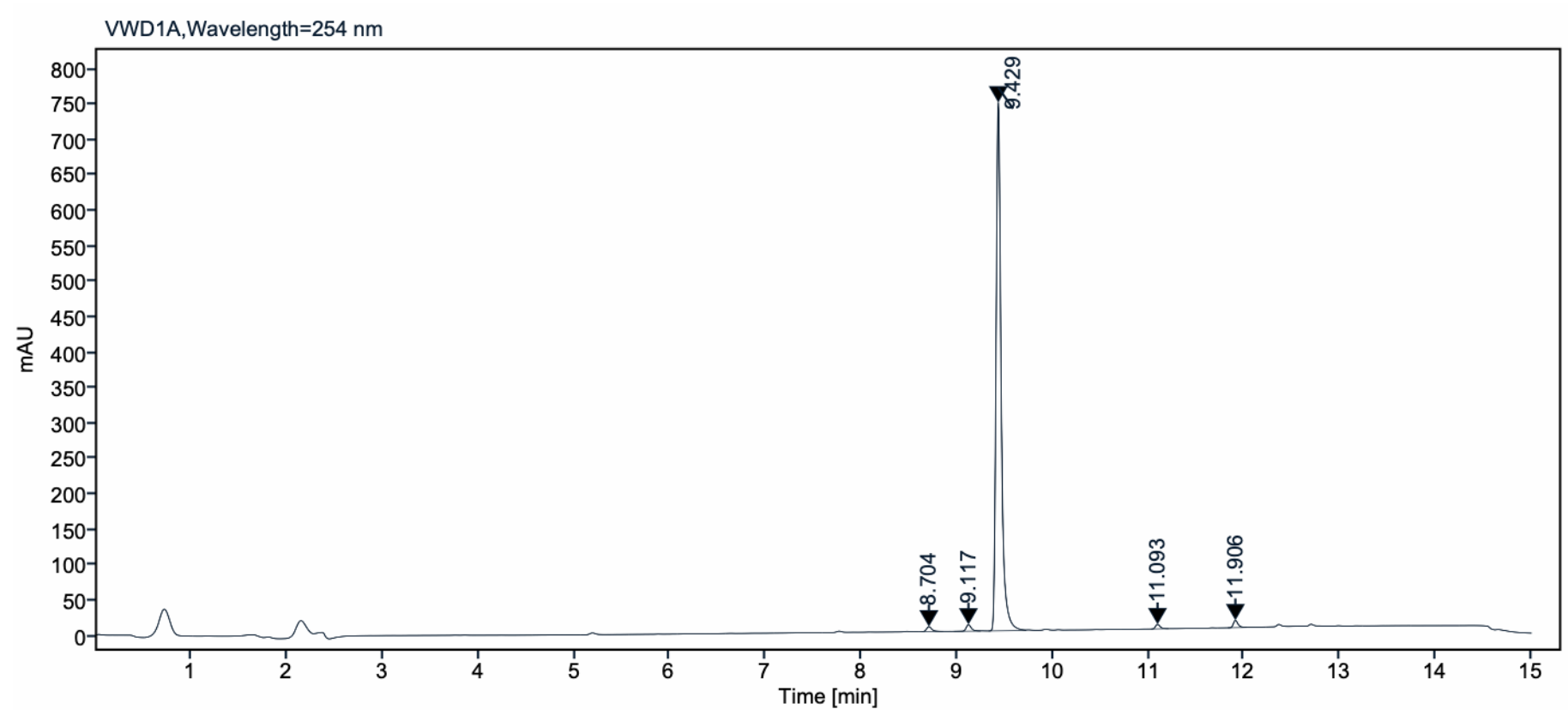

RT [min]	Width [min]	Area	Height	Area%
9.117	0.2764	34.5477	9.2698	1.2004
9.429	0.3885	2760.3749	745.8469	95.9139
11.906	0.1963	34.9644	10.2218	1.2149
	Sum	2829.8869		

ALK-15

**Instrument:**	LCMS	**Location:**	P1-F11
		**Injection:**	1 of 1
**Acq. method:**	analytical-testing-NEW-NPN.amx	**Injection volume:**	5.000 µL
**Analysis method:**	*Purity-Cole.pmx	**Acq. operator:**	SYSTEM
**Column name:**	Poroshell_120_EC-C18	**Diameter:**	3
**Serial #:**	USCFW33961	**Length:**	150
		**Particle Size**	2.7

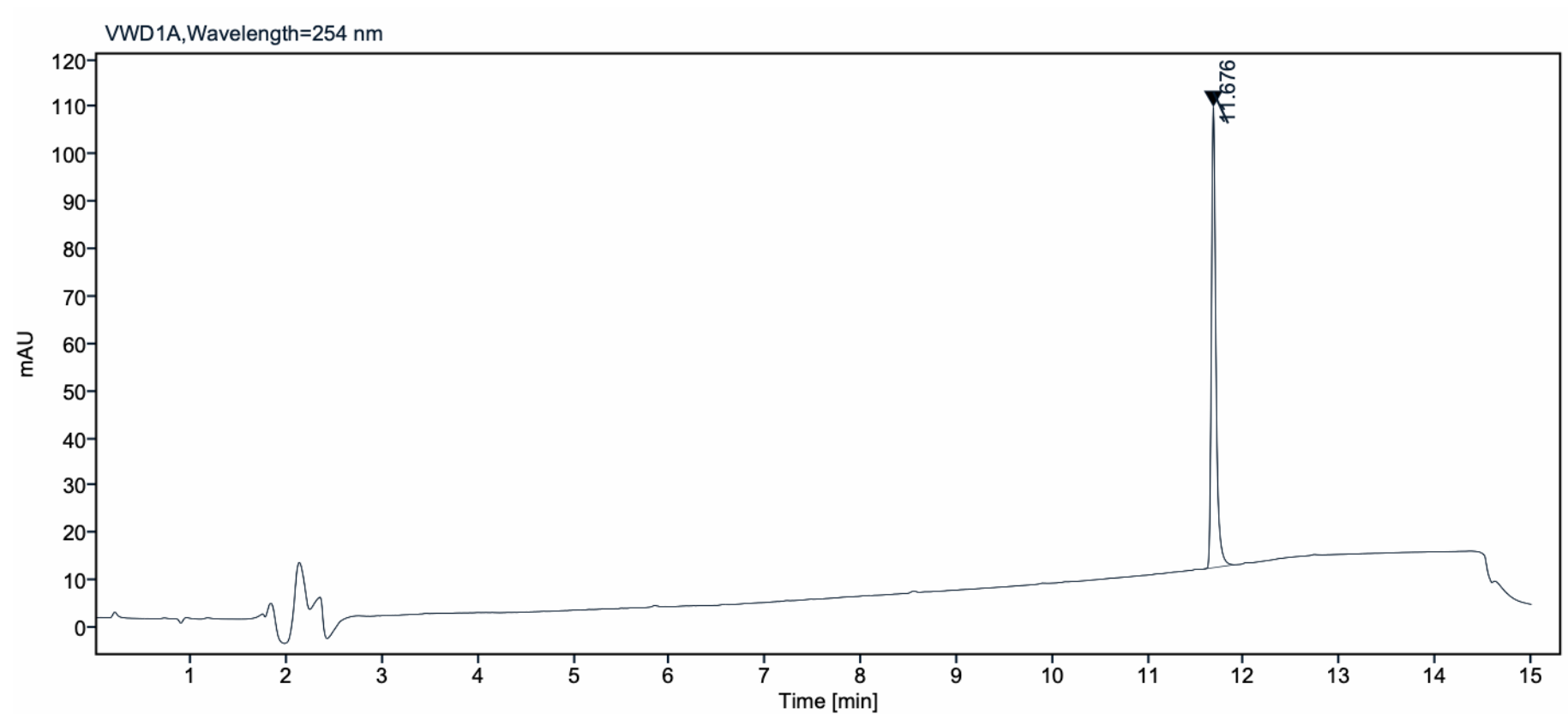

RT [min]	Width [min]	Area	Height	Area%
11.676	0.3201	318.1357	97.3288	100.0000
	Sum	318.1357		

AR-01

Instrument: LCMS
Acq. method: analytical-testing-NEW-NPN.amx
Analysis method: *Purity-Cole.pmx

Location: P1-F1
Injection: 1 of 1
Injection volume: 5.000 µL
Acq. operator: SYSTEM

Column name: Poroshell_120_EC-C18
Serial #: USCFW33961

Diameter: 3
Length: 150
Particle Size 2.7

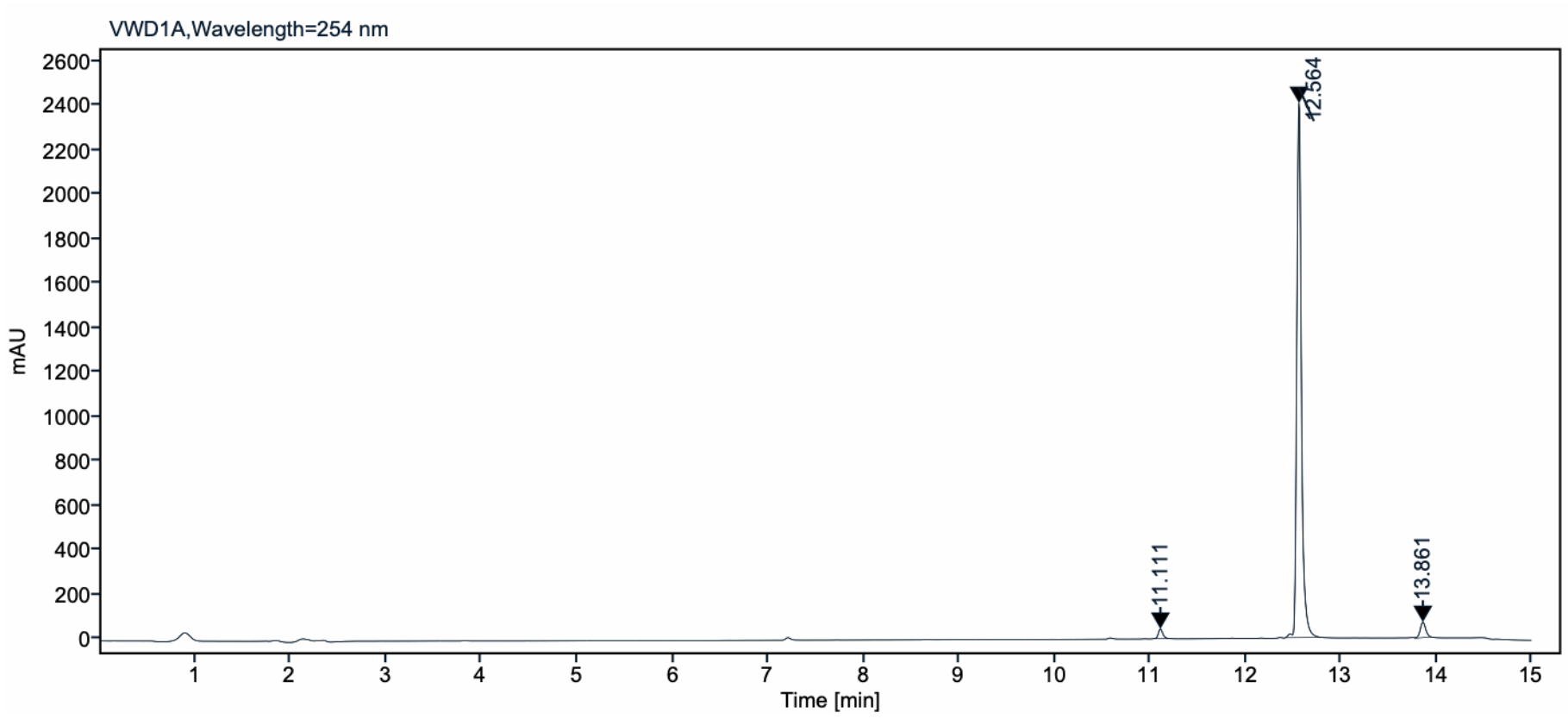

RT [min]	Width [min]	Area	Height	Area%
11.111	0.1467	136.3538	42.8635	1.5933
12.564	0.3744	8144.0251	2404.0461	95.1649
13.861	0.1705	277.4209	69.4628	3.2417
	Sum	8557.7997		

AR-04

Instrument: LCMS
Acq. method: analytical-testing-NEW.amx
Analysis method: Purity-Cole.pmx

Location: P1-E11
Injection: 1 of 1
Injection volume: 5.000 µL
Acq. operator: SYSTEM

Column name: Poroshell_120_EC-C18
Serial #: USCFW33961

Diameter: 3
Length: 150
Particle Size 2.7

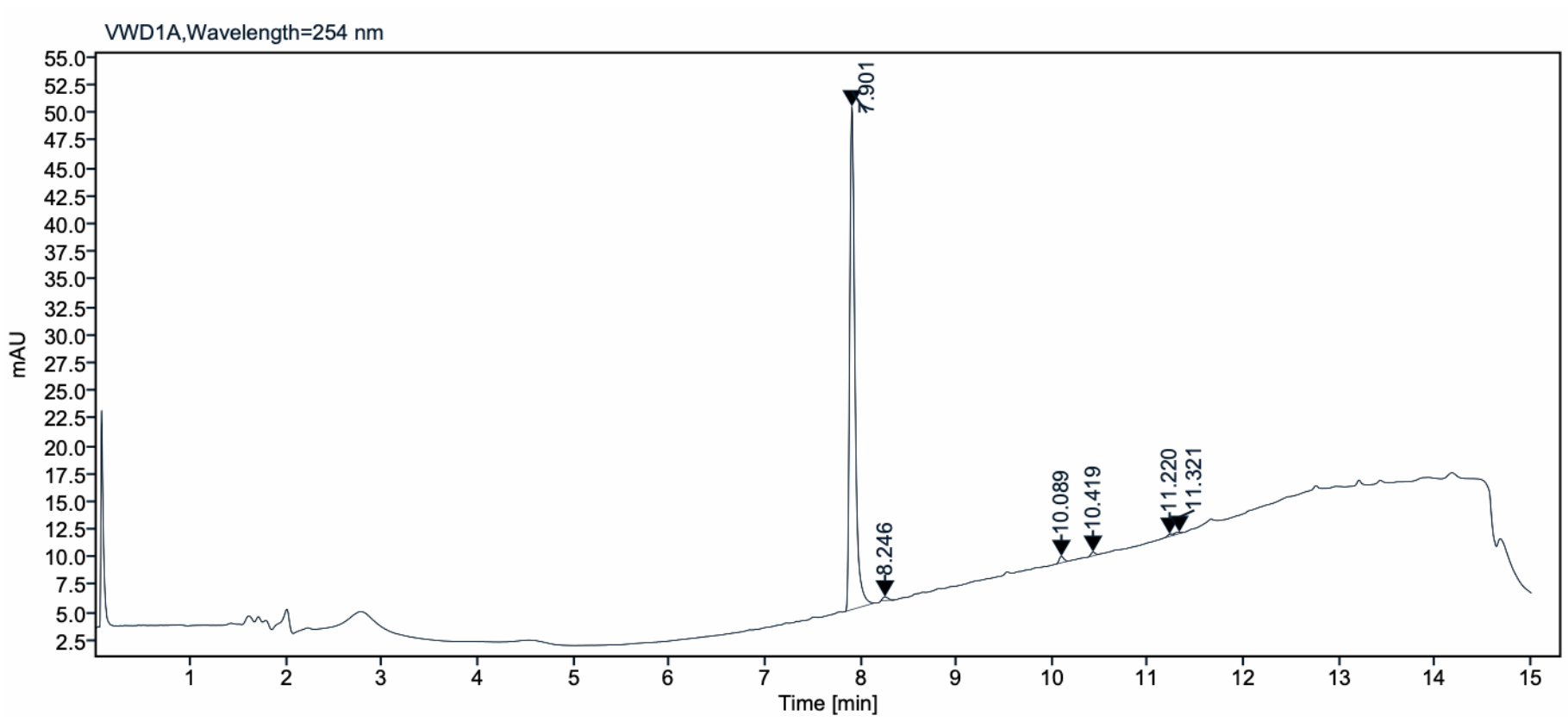

RT [min]	Width [min]	Area	Height	Area%
7.901	0.3216	174.3517	45.2799	96.8497
10.089	0.1457	1.9391	0.5736	1.0771
	Sum	176.2908		
